# DNA barcoding reveal patterns of species diversity among northwestern Pacific molluscs

**DOI:** 10.1038/srep33367

**Published:** 2016-09-19

**Authors:** Shao’e Sun, Qi Li, Lingfeng Kong, Hong Yu, Xiaodong Zheng, Ruihai Yu, Lina Dai, Yan Sun, Jun Chen, Jun Liu, Lehai Ni, Yanwei Feng, Zhenzhen Yu, Shanmei Zou, Jiping Lin

**Affiliations:** 1 Key Laboratory of Mariculture, Ministry of Education, Ocean University of China, Qingdao 266003, China

## Abstract

This study represents the first comprehensive molecular assessment of northwestern Pacific molluscs. In total, 2801 DNA barcodes belonging to 569 species from China, Japan and Korea were analyzed. An overlap between intra- and interspecific genetic distances was present in 71 species. We tested the efficacy of this library by simulating a sequence-based specimen identification scenario using Best Match (BM), Best Close Match (BCM) and All Species Barcode (ASB) criteria with three threshold values. BM approach returned 89.15% true identifications (95.27% when excluding singletons). The highest success rate of congruent identifications was obtained with BCM at 0.053 threshold. The analysis of our barcode library together with public data resulted in 582 Barcode Index Numbers (BINs), 72.2% of which was found to be concordantly with morphology-based identifications. The discrepancies were divided in two groups: sequences from different species clustered in a single BIN and conspecific sequences divided in one more BINs. In Neighbour-Joining phenogram, 2,320 (83.0%) queries fromed 355 (62.4%) species-specific barcode clusters allowing their successful identification. 33 species showed paraphyletic and haplotype sharing. 62 cases are represented by deeply diverged lineages. This study suggest an increased species diversity in this region, highlighting taxonomic revision and conservation strategy for the cryptic complexes.

DNA barcoding - sequencing a standard region of the mitochondrial cytochrome c oxidase 1 gene (COI) - has become a standardized and broadly used molecular approach for specimen identification and species discrimination^1^,^2^. Specimen identification is based on the evidence that selected DNA sequences are more variable among species than within species^3^. It involves assigning taxonomic names to a query sequence using a DNA reference library of taxonomically preidentified vouchers. Given this premises, the reliability of DNA barcoding is largely determined by the quality of the reference barcode libraries to which the unknown specimen is compared^4^. Generating rapid and accurate identifications of specimen with DNA barcodes can help to resolve distorted views of biodiversity^5^. DNA barcoding therefore represents a powerful tool for biodiversity assessment (species discovery), quickly sorting collections into species-like units^1^.

Many criticisms to DNA barcoding have been raised in the literature for the shortcomings of experimental design and analytical procedure^6^,^7^. For example, problems mostly occur when phylogenetic methods (e.g. neighbour joining) are used as the only analytical method, and identification success rates are not quantified^8^. However, a quantification of monophyly still remains a useful description of the data, when it was used in conjunction with other methods^6^. Thus, further barcoding studies should push forward improvements in data analysis, making more use of alternative methods. As explained by Collins and Cruickshank (2012), the sequence-based specimen identification criteria, such as ‘best close match’ criteria, make DNA barcoding a powerful tool in terms of established classification. When evaluating DNA barcoding as a biodiversity assessment tool (species discovery), a method is required that can estimate the number of species in mixed-organism sample directly from the barcode sequence data, and independently from the prior taxonomic species assignments preassigned taxonomic names (i.e. the data set used to subsequently measure consistency between the two approaches). The Barcode Index Numbers (BINs) analysis tool computed by the Barcode of Life Data system (BOLD)^9^ are able to use genetic information to generate an approximate of the number of operational taxonomic units that closely correspond to species.

The region of the northwestern Pacific comprising the countries of China, Japan, North Korea, South Korea, and Russia is characterized by distinct tectonic and geographical features, producing more than 75 percent of the marginal basins found on the Earth today^10^. The richest diversity of many marine taxa was found in these waters because of the complicated geological history and dramatic variations in local climates^11^,^12^,^13^. Therefore, biodiversity research and conservation efforts in this area are necessary. Marine molluscs are the most diverse phylum of marine life^14^. However, in recent years, increasingly violent and vigorous impacts of global climate change, coastal environment deterioration and anthropogenic activities have resulted in marked decline of biodiversity, and the number of endangered marine molluscs species have been distinctly increased. Moreover, the marine molluscs present a significant challenge for morphological approaches to specimen identification because they exhibit differences in life stage, frequently have morphologically cryptic taxa, and substantial phenotypic plasticity^15^,^16^, which hampered the conservation and management of the richest diversity of this taxa. In this sense, reliable specimen identification and biodiversity monitoring of organism in the field is quite necessary.

Many studies have validated the efficacy of DNA barcoding in specimen identification and species discovery for molluscs. Zou *et al.* (2011) demonstrates the effectiveness of the character-based barcoding method for specimen identification in Neogastropoda^17^. Aside from enabling identifications for whole specimens, barcode analysis opens up new possibilities - it can provide identifications during any stage of development. Puillandre *et al.* (2009b) clearly demonstrated the ability of barcodes to identify gastropod larvae, although barcode data are sparse and taxonomic coverage is biased toward shallow water species^18^. Teske *et al.* (2007) reported that the sympatric intertidal limpets (Siphonariidae) off coastal southeast Africa lacked barcode differences, suggesting they are morphotypes of a single species^19^. Two clams of the genus *Donax* showed no significant barcode variation and were found to represent one species^20^. Barcodes have also revealed lack of genetic differentiation among some species of molluscs, given that not all morphological differences are the result of cladogenesis^5^. Several prior studies have established the value of DNA barcoding in resolving morphologically cryptic species complexes in several molluscan families^22^,^23^. Despite the demonstrated utility of DNA barcoding in marine molluscs, these works focus either on restricted geographic areas and/or on a relatively restricted number of closely related species. No study has aimed to assemble a comprehensive barcode library for the entire Mollusca phylum of a large geographic area.

In this study, we establish a comprehensive barcode reference library for the marine molluscs of the northwestern Pacific (China, Japan and Korea), to test the efficacy of our DNA library for specimen identifications and shed new light on the northwestern Pacific molluscs diversity by employing different analytical approaches.

## Results

**Figure 1:**
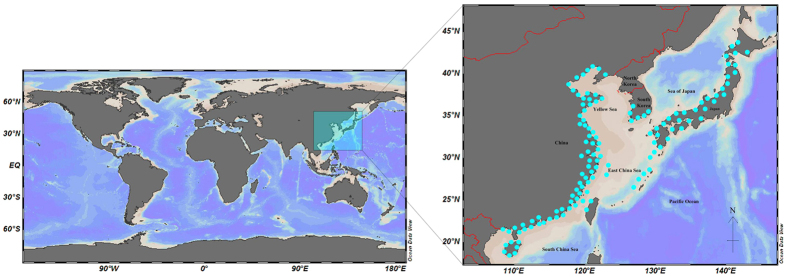
Distribution map for all sampling sites (magenta circles) in the region of the northwestern Pacific. The countries surrounding the study area: Greater China, Japan, and Korea. The location details and a list of the number of samples collected per site are available in the Supplementary Table S1. Both the map of World and the map of northwestern Pacific with Greater China, Japan, and Korea were rendered with ODV v4.7.3^63^ (available at http://odv.awi.de) and modified in Microsoft Office.

Surveys of three countries (Fig. 1) assemblages yielded a total of 2,801 sequences for the northwestern Pacific molluscs, belonging to 91 families, 240 genera, and 569 species. The taxonomy, accession numbers and the site of collection are available at Supplementary Table S1. For most species, multiple specimens (mean = 4.9 specimens per species) were analyzed to document intraspecific variability. 182 species were represented by a single specimen, and 1 species (*Cellana nigrolineata*) was represented by 62 specimens. The average nucleotide frequencies for all 573 species are as follows: A = 22.97%, T = 39.41%, G = 20.96% and C = 16.66%. Mean GC content averaged 37.62% (SE = 0.06), but showed considerable variation (range 29.94–52.02%). A chi-square test of homogeneity demonstrated significant variation in nucleotide frequencies among species in each of five molluscan classes (*P* < 0.001). Mean nearest neighbour distances between congeneric species showed a significant (*P* < 0.001; *R*^2^ = 0.167) positive correlation with mean GC content (Supplementary Fig. 1).

### Distance summary

**Figure 2:**
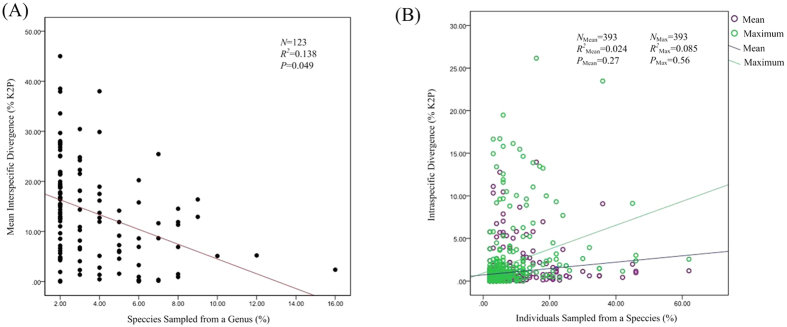
(**A**) The relationship between interspecific divergence and sample size within genera. Mean interspecific divergence (% K2P) at COI plotted against the number of species sampled from each genus of marine molluscs with ≥2 species (*N* = 123). The correlation was insignificant (*P* = 0.052; *R*^*2*^ = 0.08). (**B**) The relationship between intraspecific divergences and sample size within species. Mean and maximum intraspecific divergences (% K2P) at COI plotted against the number of individuals analyzed for 393 species of northwestern Pacific molluscs. The correlation between sample size and mean intraspecific divergence is insignificant (*P* = 0.27; *R*^*2*^ = 0.024) as well as the maximum intraspecific divergence (*P* = 0.56; *R*^*2*^ = 0.085).

We observed a hierarchical increase in mean divergence according to different taxonomic levels, within species (mean = 0.97%, SE = 0.023), within congeners (mean = 18.67%, SE = 0.004), within families (mean = 22.47%, SE = 0.003), within orders (mean = 25.3%, SE = 0.002) and within classes (mean = 30.60%, SE  =  0.012) (Table 1). Therefore, there was *ca* 19.25× more variation among congeneric species than among conspecific individuals. A regression analysis revealed that the mean interspecific divergence appeared to increase with the number of species analyzed from a genus, but the regression was not significant (Fig. 2A; *P* = 0.049; *R*^2^ = 0.138). And the intraspecific divergence did not significantly differ with the number of individuals analyzed per species (Fig. 2B; *P* = 0.27; *P* = 0.56).

### Barcode gap analysis

**Figure 3:**
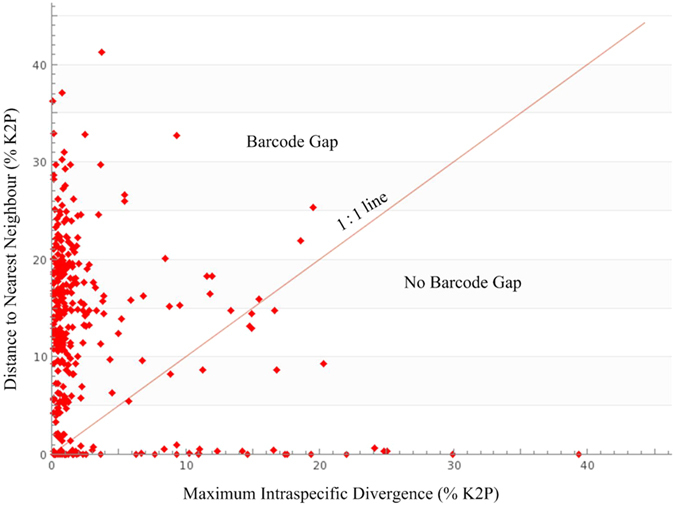
Statistical results of DNA barcoding performance. (**A**) Maximum intraspecific divergence compared with the nearest-neighbor distance for northwestern Pacific molluscs. Only species with multiple sequences are presented. Points above the line indicate species with a barcode gap. (**B**) Performance based on taxon clustering in Neighbor-joining analysis.

We counted how often the maximum sequence divergence among individuals of a species exceeded the minimum sequence divergence from another congeneric species. These situations, which may confound barcode-based taxonomic assignments, were encountered in 70 species (12.30%) (Fig. 3, Supplementary Table S2). In these species, the maximum intraspecific variation overlaps with the NN (nearest neighbour) distance, leading to the absence of a barcode gap and in 36 case, NN distances were zero. 91 species show low distance to the NN (<=2%), but still exceeded the maximum intraspecific value.

### Success of sequence-based specimen identification techniques

In the simulations, the BM approach returned 89.15% of true and 10.92% of false identifications (Table 2). When singletons were removed, false identifications decreased to 4.73%. Details of simulation results are available as Supplementary Table S3. With a threshold of 0.01, the BCM analysis provided 68.62% of true and 1.14% of false identifications. For 14.28% of the queries, the result is ambiguous (more than one equally close matches were found below the threshold of 0.01). 15.96% of the queries had no conspecific matches below the threshold of 0.01, and almost half of these (40.72%) were singletons with no conspecific sequence available. The threshold optimization method (‘threshVal’ function in SPIDER) reported a threshold between 0.0135 and 0.0260 (Supplementary Fig. 2). The average value of 0.02 was selected as the optimized threshold for the analyses. Under this threshold, the BCM approach provided 74.94% of true, 1.75% of false identifications, and the ambiguous queries were 15.42%. The remaining 7.89% queries were unidentified. The ‘localMinima’ function in SPIDER returned the threshold of 0.053 as possible transition between intra- and interspecific distances (Supplementary Fig. 3). With this threshold, the BCM approach provided 76.29% of true, 2.46% of false and 15.49% of ambiguous identifications, while 5.75% had no identification. When singletons were excluded, the false and unidentified queries decreased under each threshold. The ASB analysis returned the same results as BCM at the threshold value 0.01. While the BCM approach returned a slightly higher success rate than that of ASB approache from threshold value 0.021 and 0.053.

### BIN discordance report and the nearest neighbour analysis

The BIN analysis included 2591 of the 2801 records and generated 582 different BINs. A number of 387 BIN clusters was found to be taxonomically concordant with other barcode data on BOLD assigned to the same species name (Supplementary Table S4). Five records was indicated as singleton, which means that this BIN only refers to one specimen (Supplementary Table S5). BIN discordance analysis returned 190 BINs as discordant respect to our prior taxonomic assignments (Supplementary Table S6). The external (incl. BOLD data) incongruence occurred at different taxonomic levels: the highest rank of conflict was found at one phylum level, followed by five at order, as well as nine family level. At the genus level, 62 BINs were found to be discordant, which means that specimens belonging to different genera of the same family were grouped together in one BIN. Finally, 113 BINs incorporate specimens of at least two congeneric species. Within BNPM data, 72.2% of BINs was found to be concordantly with morphology-based identifications. The discrepancies include two groups: (i) 45 discordant BINs caused by haplotype sharing and low between-species divergence (Table 3), and (ii) 68 species clusters were assigned to two or more BINs (Table 4).

The nearest neighbour (NN) of each BIN according to the data available in BOLD is available as Supplementary Table S3. The NN comparison evidenced the under-representation of mollusc species on BOLD and the need for taxonomic reassessment of some species: 65% of BINs generated by our entries had a congeneric NN, 20% had a NN from the same family or a higher taxonomic rank, and 14% had a NN represented by an unidentified specimen.

### Neighbour-joining analysis

The neighbour joining (NJ) tree profile showed that sequence records for 2,320 (83.0%) queries representing 355 (62.4%) of all species formed distinct barcode clusters allowing their successful identification. 299 sequences involve 31 cases of paraphyly or shared barcodes between closely related species pairs, making their misidentification. Due to the lack of conspecific sequences in the data set, 31.9% of species are ambiguous and remain unidentified (Supplementary Fig. 4). Therefore, a large proportion of sequences (83.0%) and species (62.4%) were unambiguously distinguishable using the criterion of barcode clusters.

**Figure 4:**
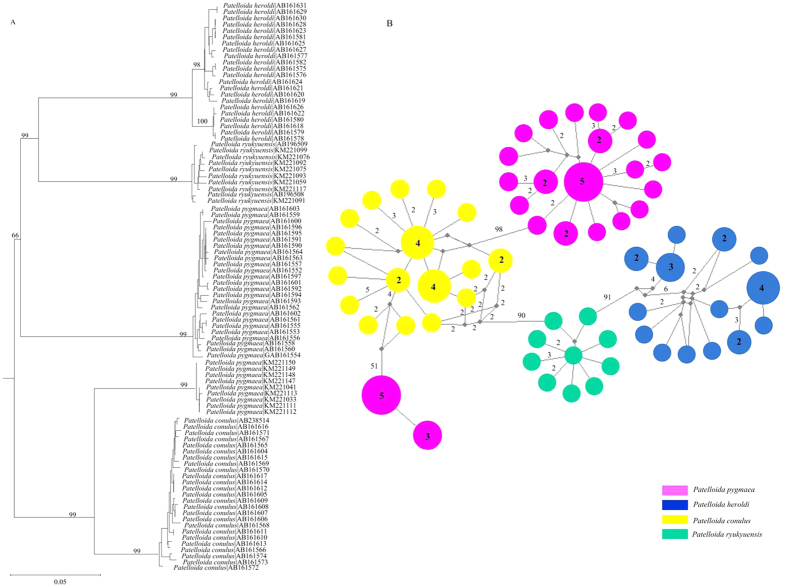
The phylogenetic analysis of four species of the genus *Patelloida*. (**A**) Neighbour-Joining (NJ) tree shows the relationships of the *Patelloida* spp. based on the K2P parameter model with bootstrap values more than 50% indicated. (**B**) The network connecting the haplotypes documented in the *Patelloida* spp. Haplotypes are represented by circles. The numbers on the internodes indicate mutation steps, and the other numbers are the frequencies of each haplotype. Color-coding represents distinct species. The black solid circle indicates missing intermediate steps between observed haplotypes.

Thirteen of these 31 problematic cases involved species that formed paraphyletic clusters (Supplementary Fig. 4, groups highlighted in yellow; e.g., *Patelloida pygmaea*; Fig. 4A). For *P. pygmaea*, some of the taxa exhibiting deep intraspecific divergence values were recovered as paraphyletic in phylogenetic trees; nevertheless, the haplotype networks of the paraphyletic species demonstrated that no shared haplotype was found between each pair of the species (e.g., *Patelloida* spp.; Fig. 4B).

**Figure 5:**
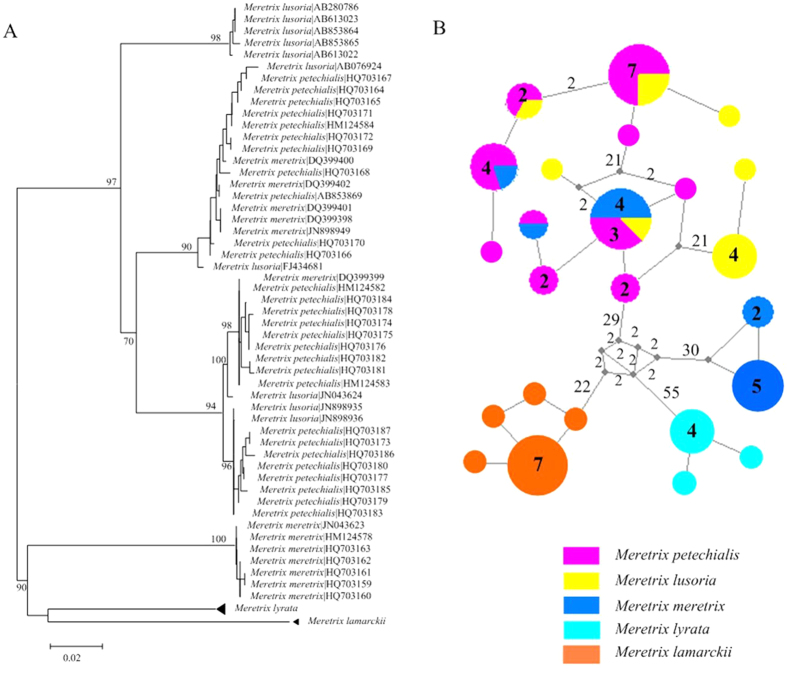
The phylogenetic analysis of five species of the genus *Meretrix*. (**A**) Neighbour-Joining (NJ) tree of barcodes from individuals of the genus *Meretrix* based on the K2P parameter model with bootstrap values more than 50% indicated. (**B**) Haplotype networks of *Meretrix* species. Haplotypes are represented by circles. The numbers on the internodes indicate mutation steps, and the other numbers are the frequencies of each haplotype. The haplotypes have a size proportional to the number of analyzed specimens with this haplotype. Color-coding represents distinct species. The black solid circle indicates missing intermediate steps between observed haplotypes.

Members of six species pairs and two species trioes showed cases of barcode sharing, producing a mixed-species cluster in the NJ tree (Supplementary Fig. 4, framed clusters; e.g., *Meretrix* spp; Fig. 5A). For *Meretrix* spp., the sharing of COI haplotypes was found in the haplotype networks of the closely related species (Fig. 5B). Overall, all these eighteen species with undifferentiated barcodes formed only fifteen clusters in phylogenetic trees.

**Figure 6:**
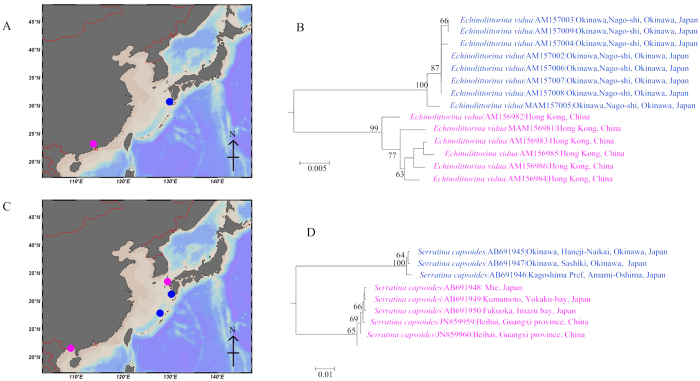
Examples of taxa with deep intraspecific divergence. (**A**) Sampling sites of the two COI lineages found in *Echinolittorina vidua*. The specimens of both lineages were present (scale bar, 400 km). (**B**) Neighbour-Joining (NJ) tree of COI barcodes of *E. vidua* with bootstrap values more than 50% indicated. (**C**) Sampling sites of the two COI lineages found in *Serratina capsoides.* The specimens of one of the lineages were allopatric (scale bar, 400 km). (**D**) Neighbour-Joining (NJ) tree of COI barcodes of *S. capsoides* with bootstrap values more than 50% indicated. The map of northwestern Pacific with Greater China, Japan, and Korea was rendered with ODV v4.7.3^63^ (available at http://odv.awi.de) and modified in Microsoft Office.

Deeply divergent intraspecific clusters were found within 62 of the 569 analyzed species (10.9%), indicating the occurrence of cryptic diversity (Table 5, Supplementary Fig. 4, groups highlighted in magenta). Those divergent intraspecific clusters, which correspond to divergent evolutionary lineages, were restricted to 32 of the 91 analyzed families (Table 5). The number of lineages by species varied from 2 to 4, for a total of 137 divergent lineages among 62 named species, which suggests a 13% increase in species diversity. Deeply divergent intraspecific lineages (>2%) were always (19/62) found in different geographical locations (e.g *Echinolittorina vidua* and *Serratina capsoides* Fig. 6A–D). Notably, the inflated geographical coverage changed the clustering pattern of conspecific individuals. In our data set, 3 species (*Patelloida pygmaea*, *Thais luteostoma* and *Conus sanguinolentus*) moved from monophyletic to paraphyletic after inclusion of additional populations. Consequently, we concentrated the study on how does the inclusion of geographically separated populations influence DNA barcoding. As expected, expansion of geographical coverage significantly increased intraspecific variation. The mean value of maximum intraspecific genetic distance increased eight-fold: from x ± S.E. = 1.02 ± 0.06% (when one population species was considered) to x ± S.E. = 8.77 ± 0.17% (when individuals from distinct populations were included).

## Discussion

The study represents the first comprehensive DNA barcode database for marine molluscs from the northwestern Pacific, including the collection and analysis of 569 species. It demonstrated the ability of DNA barcoding to identify species and shed a new light on their species diversity. The mean level of intraspecific divergence of 0.97% observed in northwestern Pacific molluscs was approximately two times higher than any other marine groups thoroughly surveyed with DNA barcodes, including the following: Australian marine fishes (0.39%)^24^, Australian decapods (0.46%)^25^, Australian echinoderms (0.62%)^24^, Canadian polychaetes (0.38%)^26^. Such a high level of intraspecific divergence may be explained by the limited dispersal capabilities of molluscs, which promote lineage divergence and enhanced speciation rates^27^.

No barcode sharing was detected among individuals of different species and a barcode gap was present for all but 70 cases. The NJ analysis demonstrated monophyletic clustering of haplotypes for 39 of these species. In the remaining 31 species, the distance to the NN was substantial (5.50–14.71%), but the level of the maximum intraspecific divergence was even higher (5.79–20.31%), producing the overlaps. The distance-based approach assumes that a species can be correctly identified when the mean distance to the most closely related species (nearest neighbor) is higher than the maximum intraspecific distance^28^. However, growing evidence suggests that the overlap between mean intra- and interspecific genetic distances is considerably greater with larger proportions of closely related taxa^29^,^30^. And, the extent of the barcoding gap tends to be overestimated when mean intraspecific distances are used, while smallest intraspecific distances yield more consistent results^31^. Hence, although the identification success generally declined when the overlap between intra- and interspecific distances increased, the lack of a barcoding gap is not necessarily influencing specimens identification^32^,^33^,^34^. Based on this hypothesis, in our data, the extent of the barcoding gap was not considered as a necessary predictor for the identification success.

In the BM approach, 2,497 nonsingleton queries had a conspecific sequence as closest match. Best match would perform much better if it was applied to a data set from which single-sequence species have been removed. When expand this approach to the entire BOLD database, 177 of the 182 singletons were clustered in BINs with conspecific or congeneric sequences from other projects, suggesting the 182 singletons were reduced to 5 once in the BOLD database. Fifteen BINs had a nearest neighbour from the same family or a higher taxonomic group, revealing the lack of barcode data for several molluscs^4^.

In our simulations, BCM approach returned a slightly higher success rate than that of ASB approach at the threshold value 0.021 and 0.053 used for identification. The ASB criterion is more restrictive than BCM. The BCM criterion looks only at the closest match below a defined threshold, while ASB assigns a match according to all sequences under that threshold. Thus, when sequences from different species have distances values falling below the threshold, ASB criterion returned a misidentification^4^. For this data set, BCM and ASB approaches don’t outperform tree-based specimen identification. For example, the success rate of tree-based specimen identification reached 83%, whereas BCM and ASB approaches yield lower success rate (68.62–76.29%) and has a relatively high incidence of ambiguous (14.28–19.53%). This high proportion of ambiguous identification could be due to the increasing geographic scale of sampling, the chances of encountering closely related species increase, while interspecific divergence decreases significantly^35^. With a higher chance of sequences from closely related species to fall under the threshold, more ambiguous identifications appeared.

The taxonomic reliability of DNA barcodes can be evaluated by analysing new data together with already published sequences. A taxonomic species assignment is more likely to be correct if congruent results were produced by several taxonomists. In implementing our BNPM data in the BOLD database, the majority of BINs (66.5%) was found to be taxonomically concordant with other barcode data on BOLD. For these cases, specimens analysed by at least two BOLD users were assigned the same species name and BIN. 33.4% of the BINs were found to be discordant. The highest rank of conflict was found at Phylum level at *Crassostrea gigas* (BIN:AAB2297). This discordance is probably to be caused by a typo or data-base error, as confusions between Mollusca and Arthropoda seem to be implausible. The conflict of seven species at order level and fourteen species at family level is unlikely to be caused misidentifications, as long as the data refers to adult specimens. The congruence problems at genus and particularly at species level can casused by misidentifications, because congeneric species usually be difficult to distinguish. Most of the discordances between our data and that already incorporated in the BIN pipeline were caused by the use of synonymies, inadequate taxonomy and misidentifications. This result highlights the need for an accurate taxonomic review of already published DNA barcode data, which will be one of the most relevant issues to increase the reliability of international barcode reference libraries like BOLD^36^.

In this study, the NJ phenogram derived from the complete barcode data set, resulted in thirteen paraphyly species. According to our current data, all haplotypes are species-specific, so that specimens could be attributed to the correct taxon. We emphasize that cases of paraphyly may not prevent the identification of species as they share no haplotypes. Cases of paraphyly in Central Asian butterflies were also treated as identification successes because the species involved were never found to share haplotypes^37^. Taking these cases into account, the identification success rate of DNA barcoding for northwestern Pacific molluscs specimens rises to 87.1%. However, considering only several cases involved, more sampling is required to verify the robustness of this conclusion. Simultaneously, these cases also highlight the importance of comprehensive sampling (across different populations and geographic regions) without which these species in our dataset may have appeared as reciprocally monophyletic, leading to misinterpretations of DNA barcoding performance. The BIN analysis failed to detect the concordance between identifications and genetic clustering for these paraphyly species. Seven of the species divided in more than one BIN and six cases share BINs with a nearest neighbour.

In the NJ analysis, the cases of low genetic divergence or haplotype sharing involved 18 species. In all of these species, the congeners shared the same BIN as well. In general, interspecific haplotype sharing has four possible explanations: hybridization, incomplete lineage sorting, inadequate taxonomy or misidentification^38^,^39^. Detailed analysis of such cases can provide a better understanding of the evolutionary history of the species involved. First, the identification of marine mollusks is often difficult due to the phenotypic plasticity and environment effects. They may exhibit morphological variations in different life stage, and some species have the shell reduced or (rarely) lost^40^. Most Cephalopoda species are composed of soft tissues, the measurement of which is difficult to standardize among researchers, and their growth patterns are highly responsive to environmental variables^41^. Thus, it may result in a lack of consensus regarding their taxonomy and lead to misidentification, producing an apparent case of haplotype sharing. Second, the pattern may also be attributed to hybridization or incomplete lineage sorting. The taxa share mtDNA haplotypes because of hybridization or incomplete lineage sorting of ancestral polymorphisms have been reported in Caenogastropoda, Mollusca, such as the sibling species of rough periwinkles, *Littorina arcana* and *L. saxatilis*^42^. However, this investigation is really sparse, and little is known regarding the other cases and further studies are needed to interpret the pattern. Thus, it seems inadequate to explain the cases of haplotype sharing encountered by us with hybridization pattern. In order to disentangle the relationships among the closely related species, more detailed studies (e.g., more detailed morphological analyses and population-level analyses with larger sample size) should be employed to these species in the future^43^.

Detecting cryptic and potentially new species from molecular biodiversity inventories is for many classical biologists the most appealing application of DNA barcoding^36^. Large genetic distances within traditionally recognized species accompanied by morphological, geographical and other subtle differences, have revealed cryptic species in most types of organism and habitat, from deep-sea clams to freshwater fish, and from tropical butterflies to arctic plants^44^,^45^,^46^,^47^,^48^. The proportion of species with deeply diverged lineages (>2%) among northwestern Pacific molluscs is relatively high (ca. 10.9%), revealed a significant amount of previously unrecognized cryptic diversity. This may unsurprising, given that molluscs represent a taxonomically weak-studied group of organisms. For the 569 species analyzed, our survey flagged 137 candidate species represented by 62 named species, which suggests a 13% increase in species diversity. Perhaps, this high cryptic diversity within northwestern Pacific molluscs is unsurprising, considering the fact that molluscs are the most diverse phylum of marine life, with more than 50,000 described species, coupled with a high degree of phenotypic plasticity and a shortage of taxonomists^49^. Furthermore, the marine habitats might be breeding grounds of cryptic speciation because they are the most species-rich habitats on Earth^50^ and because many of those organisms are involved in specialized interspecific interactions^48^. The highest proportion of cryptic diversity was found among family Cavoliniidae, Cultellidae, Haminoeidae, Pharidae, Pinnidae [an increase of 100% (1 of 1)], followed by family Batillariidae and Solenidae [an increase of 50% (1 of 2)] (Table 2). Nonetheless, 85% of all cryptic diversity occurs in the two most diversified classes, Bivalvia and Gastropoda. It appears that, just like for other components of biodiversity, the distribution of cryptic diversity among marine molluscs is not uniform, prompting several questions about possible taxonomic biases in the estimates of diversity. For example, do large, varied groups such as Bivalvia and Gastropoda hide unknown numbers of new species? However, sixteen of the 62 cryptic complexes are still represented by a single BIN each. Analysing the BNPM data set, 68 species were assigned to two or more BINs, because of the relatively high intraspecific divergences. It is worth noting that these species always has a conspecific sequence as their nearest neighbour, reflecting congruence between the simulations of sequence-based identifications scenario and independent clustering on BOLD. The presence of multiple BINs caused by divergent mitochondrial lineages for a single taxonomically identified species also gives some evidence for the existence of putative cryptic species^51^.

High genetic variability within a species can result from phylogeographic processes or geographically incomplete sampling^4^,^52^. In our data, 19 species are likely to exhibit notable intraspecific diversification among lineages from different geographical regions, and in 21 cases, more than one BINs were observed among different geographically population. The historical separation of the sea basins was reported to have dramatically influenced the current genetic distribution of various marine species^53^,^54^,^55^,^56^. This is particularly important when dealing with northwestern Pacific species, whose genetic structure was influenced by Pleistocene climatic fluctuations. During Pleistocene glaciations, three marginal seas (South China Sea, East China Sea and Japan Sea) of northwestern Pacific separated from each other owning to the declined sea level^57^,^58^. These three marginal seas had served as separate refugia and dramatically promoted the diversification of various marine species^53^,^54^,^55^,^57^. This geographically correlated population differentiation demonstrates that individuals from some taxa can be identified not only according to species but linked to a particular watershed^5^.

Perhaps, the genetically dissimilar taxa investigated in present study represent new species. Our calibration highlights a careful taxonomic revisionary work for these taxa, as well as the reproductive biology and ecology of the taxa involved. Because it is possible that some of the newly identified species is always accompanied by slight morphological changes that have simply been ignored, and the true number of biological species is likely to be greater than the current tally of nominal species^5^,^55^. Therefore, the current northwestern Pacific molluscs taxonomy at the species level conceals the species diversity in some groups. A good estimate of cryptic species diversity have important implications for conservation and natural resource protection and management^53^. Molecular evidence has revealed that species already considered endangered or threatened might be composed of cryptic species complexes that are even more rare than previously supposed^59^,^60^. This taxonomic shift renders one already threatened species into one more evolutionary lineages, each of which is substantially more endangered than was previously considered^61^. Moreover, species are lost at an alarming rate and looking for reproductive isolation is time-consuming, and once lost, an evolutionary lineage can never be recovered^62^. Thus, these results indicated that our DNA-based distinct evolutionary lineages highlighted in this study should be considered prioritized conservation units that need to be taken into account in protection strategies.

## Material and Methods

### Sampling and collection data

COI sequence data used for this analysis came from two sources: (i) specimens were collected from the coast of China for the purposes of DNA barcoding, and (ii) public data from China, Japan and Korea in GenBank, downloaded using the Barcode of Life Database (BOLD, www.barcodinglife.org). 1156 specimens were collected from the coast of China during 2004–2014. These samples were stored in 95% ethanol and deposited as voucher specimens in Fisheries College, Ocean University of China. The species-level identification was based on morphological characteristics according to the current literature and was conducted by taxonomists specialized in this fauna. Detailed specimen data (taxonomy, collection sites, and voucher catalogue numbers) are available via BOLD’s project ‘Barcoding of Molluscs along Coastal of China’ (BMCC). The 1645 sequences which were taken from the BOLD database are available in the BOLD project ‘Barcoding of Molluscs along Coastal of China, Japan and Korea (BMCCJK). All records used for this study were tagged with the unique identifier ‘Barcoding of Northwestern Pacific Molluscs’ (BNPM). Both the map of World and the map of northwestern Pacific with Greater China, Japan, and Korea were rendered with Ocean Data View (ODV) software, version 4.7.3 (available at http://odv.awi.de)^63^.

### Molecular Data Collection

The muscle tissue of each specimen was removed and used for DNA extraction following a CTAB method that has been modified from^64^ and a modification of standard phenol-chloroform procedure that has been described by^65^. A partial region of mitochondrial COI gene was amplified using universal primers (LCO1490 5′-GGT CAA CAA ATC ATA AAG ATA TTG G-3′ and HCO2198 5′-TAA ACT TCA GGG TGA CCA AAA AAT CA-3′) designed by^66^. For the species that were not successfully amplified by the universal COI primers, the other primers (COXAF 5′-CWA ATC AYA AAG ATA TTG GAA C-3′ and COXAR 5′-AAT ATA WAC TTC WGG GTG ACC-3′) designed by Colgan *et al.* (2001) were used^67^. PCR was carried out in a 50-μl reaction volume containing 2 U *Taq* DNA polymerase (Takara), about 100 ng of template DNA, 1 μM of forward and reverse primers, 200 μM of each dNTP, 1× PCR buffer and 2 mM MgCl_2_. The PCR reaction was carried out under the following conditions: 94 °C for 3 min, 35 cycles of 94 °C for 30 s, 48–52 °C for 1 min and 72 °C for 1 min, with a final extension period of 7 min at 72 °C. The amplified DNA was fractionated by electrophoresis in 1.5% low-melting-temperature agarose gels. PCR products were purified with EZ Spin Column DNA Gel Extraction Kit (Sangon BioTechnologies) following the manufacturer’ s protocol. The purified products were used as the template DNA for cycle sequencing reactions performed using BigDye Terminator Cycle Sequencing Kit (Applied Biosystems), and sequencing was conducted on an ABI PRISM 3730 (Applied Biosystems) automatic sequencer. Both DNA strands were sequenced to ensure accuracy.

### DNA Barcoding Analyses

Sequences were viewed and manually edited conducting with SEQMAN software (DNA-Star 7.2.1). Sequence alignment was performed using the BOLD Management and Analysis System^9^ and Clustal X software^68^. Overall data were compared using the ‘Distance Summary’ and ‘Barcode Gap Analysis’ tools on BOLD. Maximum intraspecific divergence was plotted against nearest neighbour distance to determine how often nearest neighbour distances were greater than intraspecific divergences, indicating the presence of a barcode gap. In addition, the ‘Sequence Composition’ tool on BOLD was used to examine variation in GC content among species. The Picante and VEGAN packages in Revolution R were used to perform linear regressions to determine if the number of individuals sampled within a species impacted estimates of intraspecific divergence and if the number of species sampled from a genus impacted the mean nearest neighbour distances^69^,^70^. The boot and Hmisc packages in Revolution R were used to test whether mean nearest neighbour distance was correlated with mean GC content^71^.

Genetic distances were calculated with the BOLD Management and Analysis System, employing the Kimura-2-Parameter (K2P) distance metric^72^. We analysed the quality of our data set by simulating a sequence sequence-based specimen identification scenario using R (www.r-project.org) with the libraries APE^73^ and SPIDER^74^, see also refs 4, 6 and 75. Every sequence was used as a query against the entire data set of identified sequences, and a species name was assigned based on three criteria: Best Match (BM), Best Close Match (BCM) and All Species Barcode (ASB). In BM, each query sequence was found according to its closest barcode match regardless of its distance. In BCM, the query sequence was identified by the closest barcode match with a distance below a defined threshold. In ASB, we assembled for each query a list of all barcodes sorted by similarity to the query using the same threshold as for best close match. The query sequence was identified when all matches below the threshold were conspecific.

In BM, if both sequences were from the same species, the results were “true”, whereas mismatched names were counted as “false”. Several equally good best matches from different species were considered ambiguous. In BCM and ASB, all queries without barcode match below the threshold value remained unidentified. The query was considered ‘ambiguous’ when several equally good best matches were found that belonged to a minimum of two species below the threshold (in BCM) or sequences from multiple species were found below the threshold (in ASB). Queries were labelled as ‘true’ or ‘false’ according to the respective congruence or incongruence between query identifications and prior taxonomic assignments.

Three different thresholds were used in BCM and ASB criteria. The first threshold was set to 0.01, which is the standard used by BOLD’s ID engine^9^. The second threshold was generated by the function ‘threshVal’ in SPIDER which minimizes the cumulative identification failure incorporating false-positive error (no conspecific matches within threshold but conspecific samples available) and false-negative error (more than one species recorded within threshold). The third threshold was obtained from the minimum of a density plot of genetic distances, which represents the transition between intra- and interspecifc distances, which was calculated by the function ‘localMinima’ in SPIDER.

We also compared the results of our simulations with the analysis tools provided by BOLD. In particular, we analysed the Barcode Index Numbers (BINs) assigned to our sequences according to the sequence-based clustering method implemented in BOLD^9^ and the nearest neighbour to each BIN.We used BIN assignments to (i) verify a priory species identification, (ii) to identify cases of haplotype sharing between species or low levels of interspecific distances, (iii) and to get hints on cryptic diversity (species with more than one BIN). The ‘BIN Discordance Report’ analysis tool was applied to analyse our data set together with public sequences on BOLD. BINs were identified as taxonomically discordant, if species clusters shared a BIN, or those were assigned to two or more BINs. The concordant BINs mean taht the sequences provided by at least two BOLD users were assigned the same species name and BIN.

The neighbor-joining tree^76^ of the whole data set was performed on the BOLD database. The number of divergent lineages within recognized species was calculated as the number of haplotypes, or clusters of haplotypes, with a mean divergence of over 2% from any other haplotypes or clusters of haplotypes.

Further phylogenetic analysis was performed on some species that represented, respectively, examples of paraphyletic clusters, cryptic diversity and distinct recognized species that potentially represent single evolutionary lineages. For those groups, we performed neighbor-joining analyses based on the K2P model using MEGA v. 5^77^. Branch support was estimated by bootstrapping with 1,000 replicates. In our study, the haplotype networks of the closely related species were constructed using the default 95% connection limit in the TCS software^78^.

## Additional Information

**How to cite this article**: Sun, S. *et al.* DNA barcoding reveal patterns of species diversity among northwestern Pacific molluscs. *Sci. Rep.* **6**, 33367; doi: 10.1038/srep33367 (2016).

## Supplementary Material

Supplementary Information

## Figures and Tables

**Table 1:** COI genetic divergences according to different taxonomic levels within the the northwestern Pacific molluscs.

Comparison	Min Dist(%)	Mean Dist(%)	Max Dist(%)	SE Dist(%)
Within species	0.00	0.96	26.16	0.002
Within genus,between species	0.04	18.67	36.98	0.011
Within family,between genera	6.52	22.47	40.28	0.019
Within order,between families	17.20	25.30	45.32	0.022
Within class,between order	19.33	30.60	50.59	0.026

**Table 2:** Identification success based on Best Match (BM), Best Close Match (BCM) and All Species Barcodes (ASB).

	BM	BCM (%)			ASB (%)		
		0.01	0.021	0.053	0.01	0.021	0.053
True	89.15 (95.34)	68.62 (73.27)	74.94 (79.99)	76.29 (81.41)	68.62 (73.27)	72.47 (77.36)	72.69 (77.70)
False	10.92 (4.73)	1.14 (0.88)	1.75 (1.07)	2.46 (1.11)	1.14 (0.88)	1.43 (0.73)	2.03 (0.65)
Ambiguous	—	14.28 (15.27)	15.42 (16.49)	15.49 (16.57)	14.28 (15.27)	18.21 (19.47)	19.53 (20.73)
No id	—	15.96 (10.58)	7.89 (2.44)	5.75 (0.92)	15.96 (10.58)	7.89 (2.44)	5.75 (0.92)

Three threshold values of 0.01, 0.02 (optimized threshold) and 0.053 (local minima) were used. Values in brackets represent the same analysis after the exclusion of singletons.

**Table 3:** Cases of BIN sharing involving 42 pairs and three triplets of species of northwestern Pacific molluscs.

Family	Species 1	Species 2	Species 3	Shared BIN
Acanthochitonidae	*Acanthochitona achates*	*Acanthochitona rubrolineata*	*Acanthochitona defilippi*	BOLD:ACB8074
Calliostomatidae	*Calliostoma aculeatum*	*Calliostoma sakashitai*		BOLD:AAW8762
Trochidae	*Cantharidus bisbalteatus*	*Cantharidus jessoensis*		BOLD:ACB7697
Nacellidae	*Cellana grata*	*Cellana nigrolineata*		BOLD:AAW6225
Nacellidae	*Cellana toreuma*	*Notoacmea schrenckii*		BOLD:AAI7335
Potamididae	*Cerithidea cingulata*	*Cerithidea djadjariensis*		BOLD:AAA7612
Trochidae	*Omphalius rusticus*	*Omphalius rusticus rusticus*	*Chlorostoma turbinatum*	BOLD:AAI1477
Octopodidae	*Cistopus indicus*	*Cistopus taiwanicus*		BOLD:ABA3763
Cerithiidae/Planaxidae	*Clypeomorus humilis*	*Planaxis sulcatus*		BOLD:AAO8512
Conidae	*Conus lividus*	*Conus sanguinolentus*		BOLD:AAO6206
Corbiculidae	*Corbicula fluminea*	*Corbicula leana*		BOLD:ACF5867
Veneridae	*Dosinia biscocta*	*Dosinia fibula*		BOLD:AAO9163
Plakobranchidae	*Elysia abei*	*Elysia amakusana*		BOLD:ACI2275
Plakobranchidae	*Elysia atroviridis*	*Elysia setoensis*		BOLD:ACI2277
Columbellidae/Conidae	*Euplica scripta*	*Conus aristophanes*		BOLD:AAJ7375
Fasciolariidae	*Fusinus forceps*	*Fusinus longicaudus*		BOLD:ACB7195
Idiosepiidae	*Idiosepius biserialis*	*Idiosepius paradoxus*		BOLD:AAW9588
Isognomonidae	*Isognomon acutirostris*	*Isognomon nucleus*		BOLD:AAW9229
Turbinidae	*Lunella coreensis*	*Lunella moniliformis*		BOLD:AAE3868
Turbinidae	*Lunella coronata*	*Lunella granulata*		BOLD:AAD3503
Veneridae	*Macridiscus multifarius*	*Macridiscus aequilatera*		BOLD:AAO8015
Veneridae	*Macridiscus aequilatera*	*Macridiscus semicancellata*		BOLD:AAO8016
Veneridae	*Meretrix lusoria*	*Meretrix meretrix*	*Meretrix petechialis*	BOLD:AAC6198
Veneridae	*Meretrix meretrix*	*Meretrix petechialis*		BOLD:AAC6197
Veneridae	*Mitrella bicincta*	*Mitrella burchardi*		BOLD:ACB6970
Mytilidae	*Modiolus comptus*	*Modiolus nipponicus*		BOLD:AAX4596
Mytilidae	*Mytilus coruscus*	*Mytilus galloprovincialis*		BOLD:AAB1503
Mytilidae	*Mytilus galloprovincialis*	*Mytilus edulis*		BOLD:AAA2184
Muricidae/Buccinidae	*Ocinebrellus inornatus*	*Neptunea cumingi*		BOLD:ACF4243
Buccinidae	*Neptunea kuroshio*	*Neptunea frater*		BOLD:AAF4517
Lottiidae	*Nipponacmea concinna*	*Nipponacmea nigrans*		BOLD:ACS5305
Lottiidae	*Nipponacmea radula*	*Nipponacmea schrenckii*		BOLD:AAX6432
Octopodidae	*Octopus incella*	*Octopus longispadiceus*		BOLD:AAD5241
Veneridae	*Paphia textile*	*Paphia undulata*		BOLD:AAO8673
Veneridae	*Pelecyora isocardia*	*Pelecyora trigona*		BOLD:AAO7896
Veneridae	*Periglypta puerpera*	*Periglypta compressa*		BOLD:AAL2655
Pteriidae	*Pinctada fucata*	*Pinctada martensi*		BOLD:AAY3639
Veneridae/Mactridae	*Protothaca jedoensis*	*Mactra veneriformis*		BOLD:AAB4298
Veneridae	*Ruditapes philippinarum*	*Ruditapes variegata*		BOLD:AAA3922
Sepiidae	*Sepiella inermis*	*Sepiella japonica*		BOLD:AAD8673
Strombidae	*Strombus lentiginosu*	*Strombus mutabiis*		BOLD:ACB7576
Muricidae	*Thais clavigera*	*Thais luteostoma*		BOLD:AAW6905
Muricidae	*Reishia bronni*	*Thais luteostoma*		BOLD:ACB7390
Octopodidae	*Octopus vulgaris*	*Octopus oshimai*		BOLD:AAB0289
Nacellidae	*Cellana radiata*	*Cellana radiata enneagona*		BOLD:AAC0533

The BIN for each pair or triad is shown.

**Table 4:** 68 cases in which high intraspecific divergence led to the assignment of conspecific individuals to two or more BINs.

Family	Species	Country	BINs
Acanthochitonidae	*Acanthochitona defilippi*	Korea	BOLD:ACB8074
		Korea	BOLD:AAE6153
		Korea	BOLD:AAE6152
Octopodidae	*Amphioctopus fangsiao*	China/Japan	BOLD:AAE5989
		China	BOLD:ABX6367
Pinnidae	*Atrina pectinata*	Japan	BOLD:AAD9827
		Japan	BOLD:AAD9828
Batillariidae	*Batillaria cumingii*	China/Japan	BOLD:ACY9200
		Japan	BOLD:ACB7408
Veneridae	*Callista chinensis*	China	BOLD:AAO9335
		China	BOLD:AAO9336
Trochidae	*Cantharidus callichroa*	Japan	BOLD:AAF7716
		Japan	BOLD:AAF7715
Nacellidae	*Cellana grata*	China	BOLD:ACQ5849
		Japan	BOLD:AAW6225
Nacellidae	*Cellana nigrolineata*	Japan	BOLD:AAW6225
		Japan	BOLD:AAI7331
		Japan	BOLD:ACQ2208
Potamididae	*Cerithidea djadjariensis*	Japan	BOLD:AAA7612
		Japan	BOLD:AAB1673
Trochidae	*Chlorostoma turbinatum*	Korea	BOLD:ACB8508
		Korea	BOLD:AAI1477
Veneridae	*Circe scripta*	China	BOLD:AAO5747
		China	BOLD:AAO5746
Cerithiidae	*Clypeomorus humilis*	China	BOLD:ACB8597
		China	BOLD:AAO8512
Mactridae	*Coelomactra antiquata*	China	BOLD:ACH4893
		China	BOLD:ACH4894
Conidae	*Conus sanguinolentus*	China	BOLD:AAO6206
		Japan	BOLD:ACB8444
Corbiculidae	*Corbicula leana*	Japan	BOLD:ACF5867
		Japan	BOLD:AAC2296
Personidae	*Distorsio reticularis*	China	BOLD:ACB8328
		China	BOLD:ACX3726
Muricidae	*Drupella margariticola*	China/Japan	BOLD:AAD8264
		Japan	BOLD:AAD8263
Littorinidae	*Echinolittorina vidua*	China	BOLD:AAA4229
		Japan	BOLD:ABY6936
Plakobranchidae	*Elysia ornata*	Japan	BOLD:ACI0075
		Japan	BOLD:ACI0076
		Japan	BOLD:AAM5939
Cypraeidae	*Erronea errones*	China	BOLD:AAF2702
		China	BOLD:AAB7225
Trochidae	*Ethaliella floccata*	Japan	BOLD:ACY9621
		Japan	BOLD:AAX7800
Columbellidae	*Euplica scripta*	China	BOLD:ACX3948
		China	BOLD:AAJ7375
Fasciolariidae	*Fusinus longicaudus*	Korea	BOLD:ACB7195
		China	BOLD:ACX3667
Veneridae	*Gafrarium dispar*	China	BOLD:AAO5706
		China	BOLD:AAO5707
Haminoeidae	*Haminoea japonica*	Japan	BOLD:ACH4492
		Japan	BOLD:ACH4494
		Japan	BOLD:ACH5215
		Japan	BOLD:ACI2127
Mytilidae	*Brachidontes mutalilis*	China	BOLD:ACQ6976
		China	BOLD:AAD4589
Idiosepiidae	*Idiosepius paradoxus*	Japan	BOLD:AAW9588
		Japan	BOLD:ACH3045
Littorinidae	*Littoraria intermedia*	Japan	BOLD:ACH3623
		China	BOLD:ACB7473
Littorinidae	*Littoraria scabra*	Japan	BOLD:AAK6714
		China	BOLD:ACB7955
Sepiolidae	*Loliolus beka*	China	BOLD:ABA8796
		China	BOLD:ABA8797
Lottiidae	*Lottia luchuana*	China/Japan	BOLD:AAJ2353
		China	BOLD:ACX3578
Veneridae	*Macridiscus aequilatera*	China	BOLD:AAO8015
		China	BOLD:AAO8016
Fissurellidae	*Macroschisma dilatata*	Japan	BOLD:AAJ1495
		Japan	BOLD:AAJ1496
Veneridae	*Meretrix lusoria*	China/Japan	BOLD:AAC6197
		Japan	BOLD:AAD4072
		China	BOLD:AAC6198
Veneridae	*Meretrix meretrix*	China	BOLD:AAC6198
		China	BOLD:AAO5535
		China	BOLD:AAC6197
Veneridae	*Meretrix petechialis*	China	BOLD:AAC6197
		China	BOLD:AAC6198
Columbellidae	*Mitrella bicincta*	China/Korea	BOLD:ACB6968
		Korea	BOLD:ACB6970
Trochidae	*Monodonta australis*	Korea	BOLD:ACB7447
		Korea	BOLD:ACB7257
Muricidae	*Morula striata*	Japan	BOLD:ACY9406
		Japan	BOLD:ACH4892
Mytilidae	*Mytilus galloprovincialis*	Korea	BOLD:AAB1503
		China	BOLD:AAA2184
Nassariidae	*Nassarius livescens*	China	BOLD:ACH4907
		China	BOLD:ACH4906
Nassariidae	*Nassarius siquijorensis*	China	BOLD:AAE0953
		China	BOLD:AAE0952
Buccinidae	*Neptunea cumingi*	China	BOLD:ACF4243
		Korea	BOLD:ACF4244
Neritidae	*Nerita helicinoides*	Japan	BOLD:AAH0946
		Japan	BOLD:AAH0947
Neritidae	*Nerita undata*	China	BOLD:ABY4809
		Japan	BOLD:ABY9761
Lottiidae	*Nipponacmea nigrans*	China	BOLD:ACS5305
		Japan	BOLD:AAX6433
Ostreidae	*Ostrea stentina*	Japan	BOLD:AAD3640
		Japan	BOLD:AAD5609
Veneridae	*Paphia semirugata*	China	BOLD:AAO8677
		China	BOLD:AAO8678
Veneridae	*Paphia sinuosa*	China	BOLD:AAO8671
		China	BOLD:ABA7706
Veneridae	*Paphia undulata*	China	BOLD:AAO8673
		China	BOLD:AAO8675
Lottiidae	*Patelloida pygmaea*	China	BOLD:ACB8437
		Japan	BOLD:AAB1669
Veneridae	*Periglypta puerpera*	China	BOLD:AAL2654
		China	BOLD:AAL2655
Veneridae	*Pitarina japonica*	China	BOLD:AAO6833
		China	BOLD:ACH3330
Plakobranchidae	*Plakobranchus ocellatus*	Japan	BOLD:ACH4499
		Japan	BOLD:ACB7131
		Japan	BOLD:ACH4500
		Japan	BOLD:ACH4501
Onchidiidae	*Platevindex mortoni*	China	BOLD:AAM1753
		China	BOLD:AAM4035
Veneridae	*Protothaca jedoensis*	China	BOLD:AAO5902
		China	BOLD:AAB4298
Mactridae	*Pseudocardium sachalinensis*	China	BOLD:ACX7097
		China	BOLD:ACI1599
Veneridae	*Ruditapes variegata*	China	BOLD:AAA3922
		China	BOLD:AAH7873
Sepiidae	*Sepia esculenta*	China	BOLD:AAE9622
		Japan	BOLD:AAE9621
Loliginidae	*Sepioteuthis lessoniana*	Japan	BOLD:AAA9505
		China/Japan	BOLD:AAA9503
Solenidae	*Solen grandis*	China	BOLD:ACQ3780
		China	BOLD:ACQ3781
Solenidae	*Solen strictus*	China	BOLD:ACQ5937
		China	BOLD:ACH5588
Skeneidae	*Stomatella planulata*	Japan	BOLD:ACY9511
		Japan	BOLD:AAF3287
Trochidae	*Strombus vittatus*	China	BOLD:ACX3385
		China	BOLD:ACB8333
Littorinidae	*Tectarius spinulosus*	Japan	BOLD:AAK0770
		Japan	BOLD:ACY9257
Potamididae	*Terebralia sulcata*	Japan	BOLD:AAE4101
		China/Japan	BOLD:ACQ3189
Muricidae	*Thais luteostoma*	Korea	BOLD:AAW6905
		China/Korea	BOLD:ACB7390
Cardiidae	*Vasticardium flavum*	China	BOLD:ACQ2883
		China	BOLD:ACQ2882
		China	BOLD:ACX4007

The BIN for each pair or triad is shown.

**Table 5:** Summary of the northwestern Pacific molluscs taxa analyzed.

Family	Species		
	Barcoded	Indistinguishable using barcodes	Deep intraspecific divergence (no. of Candidate species)
Veneridae	60	9	10 (21)
Trochidae	42	0	3 (8)
Muricidae	35	2	5 (11)
Mytilidae	26	3	3 (9)
Octopodidae	26	0	1 (2)
Lottiidae	23	3	2 (5)
Turbinidae	22	3	0
Littorinidae	19	0	3(6)
Buccinidae	19	0	0
Sepiidae	19	0	1 (2)
Arcidae	19	0	5 (11)
Neritidae	14	0	2 (4)
Plakobranchidae	9	0	3 (9)
Gonatidae	9	0	0
Loliginidae	9	0	1 (2)
Mactridae	9	1	1 (2)
Nassariidae	9	1	1 (2)
Conidae	8	2	0
Ostreidae	8	0	2 (4)
Potamididae	8	0	2 (5)
Pectinidae	7	0	0
Nacellidae	7	0	2 (4)
Pteriidae	6	0	0
Polyceridae	6	0	0
Strombidae	6	0	0
Calliostomatidae	6	0	0
Corbiculidae	6	2	0
Psammobiidae	5	0	0
Isognomonidae	5	0	0
Sepiolidae	5	0	1 (2)
Tellinidae	4	0	1 (2)
Pholadidae	4	0	0
Cypraeidae	4	0	1 (2)
Acanthochitonidae	3	1	1 (2)
Aglajidae	3	0	0
Calliotropidae	3	0	0
Cardiidae	3	0	1 (2)
Cerithiidae	3	0	0
Colloniidae	3	0	0
Columbellidae	3	2	1 (2)
Ficidae	3	0	0
Fissurellidae	3	0	1 (2)
Lepetidae	3	0	0
Melongenidae	3	0	0
Neritiliidae	3	0	0
Onchidiidae	3	0	1 (2)
Tonnidae	3	0	0
Vesicomyidae	3	0	0
Naticidae	2	0	0
Solenidae	2	1	1 (2)
Aeolidiidae	2	0	0
Batillariidae	2	0	1 (2)
Bursidae	2	0	0
Cassidae	2	0	0
Corbulidae	2	0	0
Elysiidae	2	0	0
Glycymerididae	2	0	0
Idiosepiidae	2	1	0
Limapontiidae	2	0	0
Noetiidae	2	0	0
Patellidae	2	0	0
Semelidae	2	0	0
Skeneidae	2	0	0
Solecurtidae	2	0	0
Stomatellidae	2	0	0
Fasciolariidae	2	0	1 (2)
Turritellidae	1	0	0
Acmaeidae	1	0	0
Aplysiidae	1	0	0
Architeuthidae	1	0	0
Cavoliniidae	1	0	1 (2)
Cocculinidae	1	0	0
Clavatulidae	1	0	0
Cultellidae	1	0	1 (2)
Donacidae	1	0	0
Dorididae	1	0	0
Haminoeidae	1	0	0
Lepetodrilidae	1	0	0
Myidae	1	0	0
Personidae	1	0	0
Pharidae	1	0	1 (2)
Pinnidae	1	0	1 (2)
Planaxidae	1	0	0
Pleurotomariidae	1	0	0
Ranellidae	1	0	0
Siphonariidae	1	0	0
Terebridae	1	0	0
Turbinellidae	1	0	0
Turridae	1	0	0
Volutidae	1	0	0
Total	569	31	62 (137)

The list includes the number of indistinguishable species and the number of species with deep intraspecific divergence (represented by lineages that diverge by over 2%), along with the total number of candidate species (Supplementary Fig. 4).
