# Supplementary material for: DNA barcoding reveal patterns of species diversity among northwestern Pacific molluscs
Source: Sci Rep. 2016 Sep 19;6:33367. doi: 10.1038/srep33367 (PMC5027561; doi:10.1038/srep33367)
Supplement: Supplementary Information [file srep33367-s1.pdf]

DNA barcoding reveal patterns of species diversity among northwestern Pacific molluscs

Shao’e Sun, Qi Li*, Lingfeng Kong, Hong Yu, Xiaodong Zheng, Ruihai Yu, Lina Dai, Yan Sun, Jun Chen, Jun Liu, Lehai Ni, Yanwei Feng, Zhenzhen Yu, Shanmei Zou, Jiping Lin

Key Laboratory of Mariculture, Ministry of Education, Ocean University of China, Qingdao 266003, China

*Corresponding author: Tel: +8653282031622. Fax: +8653282032773.
E-mail: qili66@ouc.edu.cn

Supplementary Table 1 List of analyzed specimens with detail data (taxonomy, voucher accession numbers, and collection sites).

Class	Order	Family	Genus	Species	Accession No.	Site of collection
Bivalvia	Arcoida	Arcidae	*Anadara*	*Anadara antiquata*	KU341897	Lingao, Hainan province, China
				Anadara antiquata	KU341898	Lingao, Hainan province, China
				Anadara antiquata	KU341899	Lingao, Hainan province, China
				Anadara antiquata	KU341900	Lingao, Hainan province, China
				Anadara antiquata	KU341901	Sanya, Hainan province, China
				Anadara antiquata	KU341902	Sanya, Hainan province, China
				Anadara antiquata	KU341903	Weizhou Island, Guangxi province, China
				Anadara antiquata	KU341904	Qionghai, Hainan province, China
				Anadara antiquata	KU341905	Qionghai, Hainan province, China
				Anadara crebricostata	KU341891	Beibhai, Guangxi province, China
				Anadara crebricostata	KU341892	Beibhai, Guangxi province, China
				Anadara crebricostata	KU341893	Beibhai, Guangxi province, China
				Anadara crebricostata	KU341894	Beibhai, Guangxi province, China
				Anadara crebricostata	KU341895	Beibhai, Guangxi province, China
				Anadara crebricostata	KU341896	Beibhai, Guangxi province, China
				Anadara vellicata	KU341881	Beibhai, Guangxi province, China
				Anadara vellicata	KU341882	Beibhai, Guangxi province, China
				Anadara vellicata	KU341883	Beibhai, Guangxi province, China
				Anadara vellicata	KU341884	Beibhai, Guangxi province, China
				Anadara vellicata	KU341885	Beibhai, Guangxi province, China
				Anadara vellicata	KU341886	Beibhai, Guangxi province, China
				Anadara vellicata	KU341887	Beibhai, Guangxi province, China
				Anadara vellicata	KU341888	Beibhai, Guangxi province, China
				Anadara vellicata	KU341889	Beibhai, Guangxi province, China
				Anadara vellicata	KU341890	Beibhai, Guangxi province, China
			Arca	*Arca avellana*	HM180481	Korea
				Arca avellana	HM180482	Korea

	Arca avellana	HM180483	Korea
	Arca navicularis	HQ258822	Weizhou Island, Guangxi province, China
	Arca navicularis	HQ258823	Beibhai, Guangxi province, China
	Arca navicularis	HQ258824	Beibhai, Guangxi province, China
	Arca navicularis	KU341928	Beibhai, Guangxi province, China
	Arca navicularis	KU341929	Beibhai, Guangxi province, China
Barbatia	*Barbatia fusca*	AB050899	Okinawa, nago, Okinawa, Japan
	Barbatia lacerata	HQ258828	Weizhou, Guangxi province, China
	Barbatia lacerata	HQ258829	Weizhou, Guangxi province, China
	Barbatia lacerata	HQ258830	Weizhou, Guangxi province, China
	Barbatia lacerata	HQ258831	Weizhou, Guangxi province, China
	Barbatia lacerata	HQ258832	Weizhou, Guangxi province, China
	Barbatia lacerata	HQ258833	Weizhou, Guangxi province, China
	Barbatia lacerata	HQ258834	Weizhou, Guangxi province, China
	Barbatia lacerata	HQ258835	Weizhou, Guangxi province, China
	Barbatia lacerata	HQ258826	Weizhou, Guangxi province, China
	Barbatia lacerata	HQ258827	Weizhou, Guangxi province, China
	Barbatia lacerata	HQ258836	Weizhou, Guangxi province, China
	Barbatia lacerata	AB076932	Okinawa, Iriomote Island, Okinawa, Japan
	Barbatia lima	AB076931	Kanagawa, Manazuru, Shiraiso, Japan
	Barbatia virescens	KU341920	Nanji, Zhejiang province, China
	Barbatia virescens	KU341921	Shengsi, Zhejiang province, China
	Barbatia virescens	KU341922	Lingao, Hainan province, China
	Barbatia virescens	KU341923	Shenzhen, Guangdong province, China
	Barbatia virescens	KU341924	Pingtan, Fujian province, China
	Barbatia virescens	KU341925	Xiapu, Fujian province, China
	Barbatia virescens	KU341926	Xiapu, Fujian province, China
	Barbatia virescens	KU341927	Fangchenggang, Guangxi province, China
Scapharca	*Scapharca cornea*	KU341858	Lingao, Hainan province, China
	Scapharca cornea	KU341859	Lingao, Hainan province, China
	Scapharca cornea	KU341860	Lingao, Hainan province, China

Scapharca cornea	KU341861	Lingao, Hainan province, China
Scapharca cornea	KU341862	Lingao, Hainan province, China
Scapharca cornea	KU341863	Lingao, Hainan province, China
Scapharca cornea	KU341864	Lingao, Hainan province, China
Scapharca globosa	KU341846	Sanya, Hainan province, China
Scapharca globosa	KU341847	Sanya, Hainan province, China
Scapharca globosa	KU341848	Sanya, Hainan province, China
Scapharca globosa	KU341849	Sanya, Hainan province, China
Scapharca globosa	KU341850	Sanya, Hainan province, China
Scapharca globosa	KU341851	Sanya, Hainan province, China
Scapharca globosa	KU341852	Sanya, Hainan province, China
Scapharca globosa	KU341853	Sanya, Hainan province, China
Scapharca globosa	AB254194	Saga, Japan
Scapharca gubernaculum	KU341854	Lingao, Hainan province, China
Scapharca gubernaculum	KU341855	Lingao, Hainan province, China
Scapharca gubernaculum	KU341856	Lingao, Hainan province, China
Scapharca gubernaculum	KU341857	Lingao, Hainan province, China
Scapharca inaequivalvis	KU341865	Sanya, Hainan province, China
Scapharca inaequivalvis	KU341866	Beibhai, Guangxi province, China
Scapharca inaequivalvis	KU341867	Beibhai, Guangxi province, China
Scapharca inaequivalvis	KU341868	Beibhai, Guangxi province, China
Scapharca inaequivalvis	AB076937	Osaka, Japan
Scapharca broughtonii	KU341869	Lianyungang, Jiangsu province, China
Scapharca broughtonii	KU341870	Panjin, Liaoning province, China
Scapharca broughtonii	KU341871	Rongcheng, Shandong province, China
Scapharca broughtonii	KU341872	Haiyang, Shandong province, China
Scapharca broughtonii	KU341873	Lianyungang, Jiangsu province, China
Scapharca broughtonii	AB050894	Kanagawa, Yokohama (market), Japan
Scapharca broughtonii	AB729113	Sendai Bay, Japan
Scapharca kagoshimensis	KU341874	Beibhai, Guangxi province, China
Scapharca kagoshimensis	KU341875	Beibhai, Guangxi province, China

	Scapharca kagoshimensis	KU341876	Beibhai, Guangxi province, China
	Scapharca kagoshimensis	KU341877	Ganyu, Jiangsu province, China
	Scapharca kagoshimensis	KU341878	Dandong, Liaoning province, China
	Scapharca kagoshimensis	KU341879	Lianyungang, Jiangsu province, China
	Scapharca kagoshimensis	KU341880	Qinhuangdao, Hebei province, China
	Scapharca kagoshimensis	AB854392	Japan
	Scapharca kagoshimensis	AB854393	Japan
	Scapharca kagoshimensis	AB854394	Japan
	Scapharca kagoshimensis	AB854395	Japan
	Scapharca kagoshimensis	AB854396	Japan
	Scapharca kagoshimensis	AB854397	Japan
	Scapharca kagoshimensis	AB854398	Japan
	Scapharca kagoshimensis	AB854399	Japan
	Scapharca kagoshimensis	AB854400	Japan
	Scapharca kagoshimensis	AB854401	Japan
	Scapharca kagoshimensis	AB854402	Japan
	Scapharca kagoshimensis	AB854403	Japan
	Scapharca kagoshimensis	AB854404	Japan
	Scapharca kagoshimensis	AB854405	Japan
	Scapharca satowi	AB050898	Chiba, Japan
Tegillarca	*Tegillarca granosa*	KU341910	Wenchang, Hainan province, China
	Tegillarca granosa	KU341911	Wenchang, Hainan province, China
	Tegillarca granosa	KU341912	Wenchang, Hainan province, China
	Tegillarca granosa	KU341913	Wenchang, Hainan province, China
	Tegillarca granosa	KU341914	Wenchang, Hainan province, China
	Tegillarca granosa	KU341915	Wenchang, Hainan province, China
	Tegillarca granosa	KU341916	Yueqing, Zhejiang province, China
	Tegillarca granosa	KU341917	Zhanjiang, Guangdong province, China
	Tegillarca granosa	KU341918	Shengsi, Zhejiang province, China
	Tegillarca granosa	KU341919	Rongcheng, Shandong province, China
	Tegillarca granosa	HQ258868	Xiamen, Fujian province, China

			Tegillarca nodifera	KU341906	Ganyu, Jiangsu province, China
			Tegillarca nodifera	KU341907	Ganyu, Jiangsu province, China
			Tegillarca nodifera	KU341908	Ganyu, Jiangsu province, China
			Tegillarca nodifera	KU341909	Ganyu, Jiangsu province, China
		Trisidos	*Trisidos kiyonoi*	HQ258842	Wenchang, Hainan province, China
			Trisidos kiyonoi	HQ258843	Wenchang, Hainan province, China
			Trisidos kiyonoi	KU341930	Wenchang, Hainan province, China
			Trisidos kiyonoi	KU341931	Wenchang, Hainan province, China
			Trisidos kiyonoi	HQ258845	Wenchang, Hainan province, China
			Trisidos kiyonoi	HQ258846	Beibhai, Guangxi province, China
	Glycymerididae	*Glycymeris*	*Glycymeris reevei*	AB076933	Okinawa, Okinawa, Japan
			Glycymeris rotunda	AB076934	Kanagawa, Misaki, Japan
	Noetiidae	*Arcopsis*	*Arcopsis interplicata*	HQ258875	Rizhao, Shandong province, China
			Arcopsis interplicata	HQ258876	Rizhao, Shandong province, China
			Arcopsis interplicata	HQ258877	Rizhao, Shandong province, China
			Arcopsis interplicata	HQ258878	Rizhao, Shandong province, China
			Arcopsis interplicata	HQ258879	Rizhao, Shandong province, China
		Didimacar	*Didimacar tenebrica*	KU341932	Beibhai, Guangxi province, China
			Didimacar tenebrica	KU341933	Fangchenggang, Guangxi province, China
			Didimacar tenebrica	KU341934	Nanji, Zhejiang province, China
			Didimacar tenebrica	KU341935	Nanji, Zhejiang province, China
			Didimacar tenebrica	KU341936	Nanji, Zhejiang province, China
			Didimacar tenebrica	HQ258871	Nanji, Zhejiang province, China
Myoida	Corbulidae	*Corbula*	*Corbula amurensis*	KJ028746	Fenshui, Shandong province, China
			Corbula amurensis	KJ028747	Panjin, Liaoning province, China
			Corbula amurensis	KJ028748	Panjin, Liaoning province, China
			Corbula amurensis	KJ028749	Panjin, Liaoning province, China
			Corbula amurensis	KJ028750	Panjin, Liaoning province, China
			Corbula amurensis	KJ028751	Fenshui, Shandong province, China
			Corbula amurensis	KJ028752	Fenshui, Shandong province, China
			Corbula amurensis	KJ028753	Zhoushan, Zhejiang province, China

			Corbula amurensis	KJ028754	Zhoushan, Zhejiang province, China
			Corbula amurensis	KJ028755	Zhoushan, Zhejiang province, China
			Corbula amurensis	KJ028756	Shengsi, Zhejiang province, China
			Corbula amurensis	KJ028757	Shengsi, Zhejiang province, China
			Corbula erythrodon	KJ125419	Haishantang, Guangxi province, China
	Myidae	*Mya*	*Mya arenaria*	KJ125420	Rongcheng, Shandong province, China
			Mya arenaria	KJ125421	Rongcheng, Shandong province, China
	Pholadidae	*Barnea*	*Barnea davidi*	KJ125426	China
			Barnea dilatata	KJ125414	China
			Barnea dilatata	KJ125415	China
		Martesia	*Martesia striata*	KJ125424	China
			Martesia striata	KJ125425	China
		Pholas	*Pholas orientalis*	KJ125422	Sanya, Hainan province, China
			Pholas orientalis	KJ125423	Sanya, Hainan province, China
Mytiloida	Mytilidae	*Adipicola*	*Adipicola crypta*	AB539004	Kagoshima, Japan
			Adipicola crypta	AB257519	Kagoshima, Japan
			Adipicola crypta	AB257518	Kagoshima, Japan
			Adipicola crypta	AB257517	Kagoshima, Japan
			Adipicola crypta	AB257516	Kagoshima, Japan
			Adipicola crypta	AB257515	Kagoshima, Japan
			Adipicola iwaotakii	AB257523	Ibaraki, Japan
			Adipicola iwaotakii	AB257521	Ibaraki, Japan
			Adipicola iwaotakii	AB257520	Ibaraki, Japan
			Adipicola iwaotakii	EU702325	Japan
			Adipicola iwaotakii	EU702324	Japan
			Adipicola iwaotakii	EU702323	Japan
			Adipicola pacifica	AB539005	Kagoshima, Japan
			Adipicola pacifica	HF545115	Japan
			Adipicola pacifica	AB170040	Kagoshima, Japan
			Adipicola pacifica	AB257528	Kagoshima, Japan
			Adipicola pacifica	AB257527	Kagoshima, Japan

	Adipicola pacifica	AB257526	Kagoshima, Japan
Bathymodiolus	*Bathymodiolus aduloides*	AB170054	Iheya Ridge, Mid-Okinawa Trough, Okinawa, Japan
	Bathymodiolus aduloides	AB170055	Off Kikaijima Island, Japan
	Bathymodiolus aduloides	AB170056	Off Kikaijima Island, Japan
	Bathymodiolus aduloides	AB170057	Off Kikaijima Island, Japan
	Bathymodiolus aduloides	AB170058	Off Kikaijima Island, Japan
	Bathymodiolus aduloides	AB170059	Off Kikaijima Island, Japan
	Bathymodiolus aduloides	HF545118	Japan
	Bathymodiolus aduloides	AB597557	Myojinsho,Izu-Ogasawara area, Japan
	Bathymodiolus hirtus	AB250694	Off-ishigaki Island, Kuroshima Knoll, Japan
	Bathymodiolus hirtus	AB170047	Kuroshima Knoll, Off Yaeyama Islands, Japan
	Bathymodiolus japonicus	HF545108	Japan
	Bathymodiolus japonicus	AB101422	Okinawa, Minami-Ensei Knoll, Okinawa, Japan
	Bathymodiolus japonicus	AB101423	Off Hatsushima and Okinawa, Minami-Ensei Knoll, Okinawa, Japan
	Bathymodiolus platifrons	HF545106	Japan
	Bathymodiolus platifrons	AB101419	Kanagawa, Off Hatsushima, Japan
	Bathymodiolus platifrons	AB101420	Kanagawa, Off Hatsushima, Japan
	Bathymodiolus platifrons	AB101421	Off Hatsushima and Okinawa, Iheya Ridge, Okinawa, Japan
	Bathymodiolus platifrons	AB250695	Okinawa Trough, Hatoma Knoll, Okinawa, Japan
	Bathymodiolus securiformis	AB170048	Kuroshima Knoll, Off Yaeyama Islands, Japan
	Bathymodiolus securiformis	AB170051	Kuroshima Knoll, Off Yaeyama Islands, Japan
	Bathymodiolus securiformis	AB170052	Dai-ni (no. 2) Atsumi Knoll, Nankai Trough, Japan
	Bathymodiolus securiformis	AB170053	Dai-ni (no. 3) Atsumi Knoll, Nankai Trough, Japan
	Bathymodiolus septemdierum	AB101424	Izu-Ogasawara Island-arc, Myojin Knoll, Japan
	Bathymodiolus	AB101425	Izu-Ogasawara Island-arc, Myojin Knoll, Japan

	septemdierum		
	Bathymodiolus septemdierum	AB101426	Izu-Ogasawara Island-arc, Myojin Knoll, Japan
	Bathymodiolus septemdierum	AB101427	Izu-Ogasawara Island-arc, Myojin Knoll, Japan
	Bathymodiolus septemdierum	AB101428	Izu-Ogasawara Island-arc, Suiyo Seamount, Japan
	Bathymodiolus septemdierum	AB101429	Izu-Ogasawara Island-arc, Suiyo Seamount, Japan
	Bathymodiolus septemdierum	AB101430	Izu-Ogasawara Island-arc, Suiyo Seamount, Japan
	Bathymodiolus septemdierum	AB170041	Izu-Ogsawara Island-arc, Japan
Benthomodiolus	*Benthomodiolus geikotsucola*	AB679346	Tokyo,Torishima seamount, Japan
	Benthomodiolus geikotsucola	HF545103	Japan
Gigantidas	*Gigantidas horikoshii*	HF545113	Japan
	Gigantidas horikoshii	AB257538	Kaikata Seamount, Japan
Hormomya	*Brachidontes mutalilis*	GQ480306	Fangchenggang, Guangxi province, China
	Brachidontes mutalilis	GQ480307	Fangchenggang, Guangxi province, China
	Brachidontes mutalilis	GQ480308	Fangchenggang, Guangxi province, China
	Brachidontes mutalilis	GQ480309	Lingao, Hainan province, China
	Brachidontes mutalilis	GQ480310	Lingao, Hainan province, China
Idasola	*Idasola japonica*	AB257537	Kagoshima, Japan
	Idasola japonica	AB257536	Kagoshima, Japan
Limnoperna	*Limnoperna fortunei*	AB828682	Toride, Japan
	Limnoperna fortunei	AB828681	Teganuma, Japan
	Limnoperna fortunei	AB828680	Teganuma, Japan
	Limnoperna fortunei	AB828679	Japan
	Limnoperna fortunei	AB520627	Ibaraki, Japan

	Limnoperna fortunei	AB520626	Ibaraki, Japan
	Limnoperna fortunei	AB520625	Ibaraki, Japan
	Limnoperna fortunei	AB520624	Ibaraki, Japan
	Limnoperna fortunei	AB520623	Ibaraki, Japan
	Limnoperna fortunei	AB520622	Chiba, Japan
	Limnoperna fortunei	AB520621	Chiba, Japan
	Limnoperna fortunei	AB520620	Chiba, Japan
	Limnoperna fortunei	AB520619	Chiba, Japan
	Limnoperna fortunei	AB520618	Chiba, Japan
	Limnoperna fortunei	AB520617	Chiba, Japan
	Limnoperna fortunei	AB520616	Chiba, Japan
	Limnoperna fortunei	AB520615	Chiba, Japan
	Limnoperna fortunei	AB520614	Chiba, Japan
	Limnoperna fortunei	AB520613	Kanto, Lake Ohshio, Kanto, Japan
	Limnoperna fortunei	AB520612	Kanto, Lake Ohshio, Kanto, Japan
Modiolus	*Modiolus auriculatus*	GQ480317	Sanya, Hainan province, China
	Modiolus comptus	GQ480313	Nanji, Zhejiang province, China
	Modiolus comptus	GQ480314	Nanji, Zhejiang province, China
	Modiolus comptus	GQ480315	Shengshan, Zhejiang province, China
	Modiolus comptus	GQ480316	Shengshan, Zhejiang province, China
	Modiolus elongatus	GQ480318	Fenshui, Shandong province, China
	Modiolus kurilensis	KP243079	China
	Modiolus kurilensis	KP243078	China
	Modiolus kurilensis	KP243077	China
	Modiolus kurilensis	KP243076	China
	Modiolus kurilensis	KP243075	China
	Modiolus kurilensis	KP243074	China
	Modiolus metcalfei	GQ480319	Beibhai, Guangxi province, China
	Modiolus metcalfei	GQ480320	Beibhai, Guangxi province, China
	Modiolus metcalfei	GQ480321	Fangchenggang, Guangxi province, China
	Modiolus metcalfei	GQ480322	Fangchenggang, Guangxi province, China

	Modiolus nipponicus	AB076912	Kanagawa, Manazuru, Siraiso, Japan
Musculista	*Musculista senhousia*	AB076942	Kanagawa, Ooiso, Japan
	Musculista senhousia	AB498016	Tokyo, Daiba, Japan
Mytilus	*Mytilus coruscus*	GQ480287	Zhoushan, Zhejiang province, China
	Mytilus coruscus	GQ480288	Zhoushan, Zhejiang province, China
	Mytilus coruscus	GQ480289	Zhoushan, Zhejiang province, China
	Mytilus coruscus	GQ480290	Zhoushan, Zhejiang province, China
	Mytilus coruscus	GQ480291	Zhoushan, Zhejiang province, China
	Mytilus coruscus	GQ480295	Zhoushan, Zhejiang province, China
	Mytilus coruscus	GQ480283	Zhoushan, Zhejiang province, China
	Mytilus edulis	HM180704	Korea
	Mytilus galloprovincialis	HM180705	Korea
	Mytilus galloprovincialis	HM180706	Korea
	Mytilus galloprovincialis	HM180707	Korea
	Mytilus galloprovincialis	HM180708	Korea
	Mytilus galloprovincialis	HM180709	Korea
	Mytilus galloprovincialis	HM180710	Korea
	Mytilus galloprovincialis	HM180711	Korea
	Mytilus galloprovincialis	HM180712	Korea
	Mytilus galloprovincialis	GQ480281	Haiyang, Shandong province, China
	Mytilus galloprovincialis	GQ480282	Lianyungang, Jiangsu province, China
	Mytilus galloprovincialis	GQ480284	Qingdao, Shandong province, China
	Mytilus galloprovincialis	GQ480285	Qingdao, Shandong province, China
	Mytilus galloprovincialis	GQ480286	Qingdao, Shandong province, China
	Mytilus galloprovincialis	GQ480292	Lianyungang, Jiangsu province, China
	Mytilus galloprovincialis	GQ480293	Lianyungang, Jiangsu province, China
	Mytilus galloprovincialis	GQ480294	Lianyungang, Jiangsu province, China
Perna	*Perna viridis*	GQ480296	Lingao, Hainan province, China
	Perna viridis	GQ480297	Lingao, Hainan province, China
	Perna viridis	GQ480298	Lingao, Hainan province, China
	Perna viridis	GQ480299	Lingao, Hainan province, China

			Perna viridis	GQ480300	Lingao, Hainan province, China
			Perna viridis	GQ480301	Yangjiang, Guangdong province, China
			Perna viridis	GQ480302	Yangjiang, Guangdong province, China
			Perna viridis	GQ480303	Yangjiang, Guangdong province, China
			Perna viridis	GQ480304	Yangjiang, Guangdong province, China
		Xenostrobus	*Xenostrobus atratus*	GQ480323	Beihai, Guangxi province, China
			Xenostrobus atratus	GQ480324	Beihai, Guangxi province, China
			Xenostrobus atratus	GQ480325	Beihai, Guangxi province, China
			Xenostrobus atratus	GQ480326	Beihai, Guangxi province, China
			Xenostrobus atratus	GQ480327	Beihai, Guangxi province, China
Ostreoida	Ostreidae	*Crassostrea*	*Crassostrea angulata*	HQ661008	Yangjiang, Guangdong province, China
			Crassostrea angulata	HQ661009	Pingtan, Fujian province, China
			Crassostrea angulata	EU672832	Taiwan, China
			Crassostrea angulata	AB904879	Kagoshima, Japan
			Crassostrea angulata	AB904880	Kagoshima, Japan
			Crassostrea angulata	AB904881	Kagoshima, Japan
			Crassostrea angulata	AB904882	Kagoshima, Japan
			Crassostrea angulata	AB904883	Kagoshima, Japan
			Crassostrea angulata	AB904885	Kagoshima, Japan
			Crassostrea angulata	AB904886	Kagoshima, Japan
			Crassostrea angulata	AB904887	Kagoshima, Japan
			Crassostrea angulata	AB904888	Kagoshima, Japan
			Crassostrea angulata	AB904890	Kagoshima, Japan
			Crassostrea ariakensis	HQ661020	Nantong, Jiangsu province, China
			Crassostrea ariakensis	HQ661021	Nantong, Jiangsu province, China
			Crassostrea ariakensis	EU672835	Yingkou, Liaoning province, China
			Crassostrea gigas	HQ661002	Rongcheng, Shandong province, China
			Crassostrea gigas	HQ661003	Rongcheng, Shandong province, China
			Crassostrea gigas	HQ661004	Qingdao, Shandong province, China
			Crassostrea gigas	HQ661005	Yantai, Shandong province, China
			Crassostrea gigas	HQ661006	Lianyungang, Jiangsu province, China

Crassostrea gigas	HQ661007	Lianyungang, Jiangsu province, China
Crassostrea gigas	AB636176	Japan
Crassostrea gigas	AB636174	Japan
Crassostrea gigas	AB636172	Japan
Crassostrea gigas	AB636169	Japan
Crassostrea gigas	AB636184	Japan
Crassostrea gigas	AB636183	Japan
Crassostrea gigas	AB636182	Japan
Crassostrea gigas	AB636181	Japan
Crassostrea gigas	AB636178	Japan
Crassostrea gigas	AB636171	Japan
Crassostrea gigas	AB636167	Japan
Crassostrea gigas	AB636166	Japan
Crassostrea gigas	AB904884	Kagoshima, Japan
Crassostrea gigas	AB904889	Kagoshima, Japan
Crassostrea gigas	KJ855241	Komaru, Japan
Crassostrea hongkongensis	FJ593172	Beihai, Guangxi Province, China
Crassostrea hongkongensis	EU118008	China
Crassostrea hongkongensis	EU672834	Haikou, Hainan province, China
Crassostrea hongkongensis	FJ593173	Xiamen, Fujiang province, China
Crassostrea iredalei	HQ661022	Sanya, Hainan province, China
Crassostrea iredalei	HQ661023	Sanya, Hainan province, China
Crassostrea sikamea	HQ661010	Shengsi, Zhejiang province, China
Crassostrea sikamea	HQ661011	Fuqing, Fujian province, China
Crassostrea sikamea	HQ661012	Fuqing, Fujian province, China
Crassostrea sikamea	HQ661013	Beibhai, Guangxi province, China
Crassostrea sikamea	HQ661014	Beibhai, Guangxi province, China

	Crassostrea sikamea	HQ661015	Beibhai, Guangxi province, China
	Crassostrea sikamea	HQ661016	Wenchang, Hainan province, China
	Crassostrea sikamea	HQ661017	Nantong, Jiangsu province, China
	Crassostrea sikamea	HQ661018	Nantong, Jiangsu province, China
	Crassostrea sikamea	HQ661019	Yangjiang, Guangdong province, China
	Crassostrea sikamea	AB904872	Wakayama, Japan
	Crassostrea sikamea	AB904873	Wakayama, Japan
	Crassostrea sikamea	AB904874	Wakayama, Japan
	Crassostrea sikamea	AB904875	Wakayama, Japan
	Crassostrea sikamea	AB904876	Wakayama, Japan
	Crassostrea sikamea	AB904877	Wakayama, Japan
	Crassostrea sikamea	AB904878	Wakayama, Japan
	Crassostrea sikamea	AB675954	Oita, Japan
	Crassostrea sikamea	AB675952	Oita, Japan
	Crassostrea sikamea	AB675950	Oita, Japan
	Crassostrea sikamea	AB675948	Oita, Japan
	Crassostrea sikamea	AB675955	Oita, Japan
	Crassostrea sikamea	AB675953	Oita, Japan
	Crassostrea sikamea	AB675951	Oita, Japan
	Crassostrea sikamea	AB675949	Oita, Japan
	Crassostrea sikamea	AB675947	Oita, Japan
Ostrea	*Ostrea stentina*	LC051588	Kagoshima, Ibusuki, Japan
	Ostrea stentina	LC051586	Kagoshima, Ibusuki, Japan
	Ostrea stentina	LC051585	Kagoshima, Ibusuki, Japan
	Ostrea stentina	LC051589	Kagoshima, Ibusuki, Japan
	Ostrea stentina	LC051591	Kagoshima, Ibusuki, Japan
	Ostrea stentina	LC051587	Kagoshima, Ibusuki, Japan
	Ostrea stentina	LC051590	Kagoshima, Ibusuki, Japan
	Ostrea stentina	LC051583	Wakayama, Kemi, Japan
	Ostrea stentina	LC051584	Wakayama, Kemi, Japan
	Ostrea stentina	LC051582	Wakayama, Kemi, Japan

		Saccostrea	*Saccostrea mordax*	HQ661025	Sanya, Hainan province, China
			Saccostrea mordax	HQ661026	Sanya, Hainan province, China
			Saccostrea mordax	HQ661027	Sanya, Hainan province, China
			Saccostrea mordax	HQ661028	Sanya, Hainan province, China
			Saccostrea mordax	HQ661029	Weizhou Island, Guangxi province, China
Pectinoida	Pectinidae	*Amusium*	*Amusium pleuronectes*	GU120012	Lingao, Hainan province, China
			Amusium pleuronectes	GU120013	Lingao, Hainan province, China
			Amusium pleuronectes	GU120014	Lingao, Hainan province, China
			Amusium pleuronectes	GU120015	Lingao, Hainan province, China
			Amusium pleuronectes	GU120016	Lingao, Hainan province, China
			Amusium pleuronectes	GU120017	Lingao, Hainan province, China
			Amusium pleuronectes	GU120018	Lingao, Hainan province, China
			Amusium pleuronectes	GU120019	Lingao, Hainan province, China
		Argopecten	*Argopecten irradians*	GU120020	Zhoushan, Zhejiang province, China
			Argopecten irradians	GU120021	Zhoushan, Zhejiang province, China
			Argopecten irradians	GU120022	Zhoushan, Zhejiang province, China
			Argopecten irradians	GU120023	Zhoushan, Zhejiang province, China
			Argopecten irradians	GU120024	Beibhai, Guangxi province, China
			Argopecten irradians	GU120025	Beibhai, Guangxi province, China
		Chlamys	*Chlamys farreri*	GU119998	Dalian, Liaoning province, China
			Chlamys farreri	GU119999	Dalian, Liaoning province, China
			Chlamys farreri	GU120000	Dalian, Liaoning province, China
			Chlamys farreri	FJ595957	Qingdao, Shandong province, China
		Decatopecten	*Decatopecten pilica*	GU120026	Beibhai, Guangxi province, China
			Decatopecten pilica	GU120027	Beibhai, Guangxi province, China
			Decatopecten pilica	GU120029	Beibhai, Guangxi province, China
			Decatopecten pilica	GU120030	Beibhai, Guangxi province, China
		Mimachlamys	*Mimachlamys nobilis*	GU119985	Sanya, Hainan province, China
			Mimachlamys nobilis	GU119986	Sanya, Hainan province, China
			Mimachlamys nobilis	GU119987	Sanya, Hainan province, China
			Mimachlamys nobilis	GU119988	Sanya, Hainan province, China

			Mimachlamys nobilis	GU119989	Sanya, Hainan province, China
		Mizuhopecten	*Mizuhopecten yessoensis*	GU119990	Weihai, Shandong province, China
			Mizuhopecten yessoensis	GU119991	Weihai, Shandong province, China
			Mizuhopecten yessoensis	GU119992	Weihai, Shandong province, China
			Mizuhopecten yessoensis	GU119993	Weihai, Shandong province, China
			Mizuhopecten yessoensis	GU119994	Changdao, Shandong province, China
			Mizuhopecten yessoensis	GU119995	Dalian, Liaoning province, China
			Mizuhopecten yessoensis	GU119996	Dalian, Liaoning province, China
			Mizuhopecten yessoensis	GU119997	Dalian, Liaoning province, China
		Volachlamys	*Volachlamys hirasei*	GU120001	Panjin, Liaoning province, China
			Volachlamys hirasei	GU120002	Panjin, Liaoning province, China
			Volachlamys hirasei	GU120003	Panjin, Liaoning province, China
			Volachlamys hirasei	GU120004	Panjin, Liaoning province, China
			Volachlamys hirasei	GU120005	Panjin, Liaoning province, China
			Volachlamys hirasei	GU120006	Panjin, Liaoning province, China
			Volachlamys hirasei	GU120007	Panjin, Liaoning province, China
			Volachlamys hirasei	GU120008	Panjin, Liaoning province, China
			Volachlamys hirasei	GU120009	Panjin, Liaoning province, China
			Volachlamys hirasei	GU120010	Panjin, Liaoning province, China
Pterioida	Isognomonidae	*Isognomon*	*Isognomon acutirostris*	AB076926	Okinawa, Iriomote Island, Okinawa, Japan
			Isognomon ephippium	KU341971	Shenzhen, Guangdong province, China
			Isognomon ephippium	KU341972	Shenzhen, Guangdong province, China
			Isognomon ephippium	KU341973	Shenzhen, Guangdong province, China
			Isognomon ephippium	KU341974	Shenzhen, Guangdong province, China
			Isognomon ephippium	KU341975	Shenzhen, Guangdong province, China
			Isognomon legumen	KU341965	Weizhou Island, Guangxi province, China
			Isognomon legumen	KU341966	Weizhou Island, Guangxi province, China
			Isognomon legumen	KU341967	Shenzhen, Guangdong province, China
			Isognomon legumen	KU341968	Shenzhen, Guangdong province, China
			Isognomon legumen	KU341969	Shenzhen, Guangdong province, China
			Isognomon nucleus	KU341970	Lingao, Hainan province, China

		Isognomon perna	KU341963	Sanya, Hainan province, China
		Isognomon perna	KU341964	Sanya, Hainan province, China
		Isognomon perna	AB076918	Okinawa, Gushikami, Okinawa, Japan
Pinnidae	*Atrina*	*Atrina pectinata*	AB059421	Ariake Bay, Japan
		Atrina pectinata	AB059422	Ariake Bay, Japan
		Atrina pectinata	AB059423	Yamaguchi, Ohmi Bay, Japan
		Atrina pectinata	AB059424	Yamaguchi, Ohmi Bay, Japan
		Atrina pectinata	AB076914	Kanagawa, Yokohama, Central market, Japan
Pteriidae	*Pinctada*	*Pinctada albina*	AB261165	Kagoshima, Japan
		Pinctada chemnitzi	KU341955	Pingtan, Fujian province, China
		Pinctada chemnitzi	KU341956	Haikou, Hainan province, China
		Pinctada chemnitzi	KU341957	Caotan, Guangdong province, China
		Pinctada chemnitzi	KU341958	Shankou, Guangxi province, China
		Pinctada fucata	KU341939	Beibhai, Guangxi province, China
		Pinctada fucata	KU341940	Beibhai, Guangxi province, China
		Pinctada fucata	KU341941	Beibhai, Guangxi province, China
		Pinctada fucata	KU341942	Beibhai, Guangxi province, China
		Pinctada fucata	KU341943	Beibhai, Guangxi province, China
		Pinctada fucata	KU341944	Wenchang, Hainan province, China
		Pinctada fucata	GQ355871	Japan
		Pinctada margaritifera	KU341945	Beibhai, Guangxi province, China
		Pinctada margaritifera	KU341946	Beibhai, Guangxi province, China
		Pinctada margaritifera	KU341947	Beibhai, Guangxi province, China
		Pinctada margaritifera	KU341948	Beibhai, Guangxi province, China
		Pinctada margaritifera	KU341949	Weizhou Island, Guangxi province, China
		Pinctada margaritifera	KU341950	Weizhou Island, Guangxi province, China
		Pinctada margaritifera	KU341951	Weizhou Island, Guangxi province, China
		Pinctada margaritifera	KU341952	Weizhou Island, Guangxi province, China
		Pinctada margaritifera	KU341953	Weizhou Island, Guangxi province, China
		Pinctada margaritifera	KU341954	Weizhou Island, Guangxi province, China
		Pinctada margaritifera	AB259166	Okinawa, Okinawa, Japan

			Pinctada martensi	GQ355882	Japan
			Pinctada martensi	AB076915	Kanagawa, Manazuru, Siraiso, Japan
			Pinctada maxima	KU341937	Weizhou Island, Guangxi province, China
			Pinctada maxima	KU341938	Hainan province, China
Veneroida	Cardiidae	*Clinocardium*	*Clinocardium californiense*	JN860022	Dalian, Liaoning province, China
			Clinocardium californiense	JN860023	Qingdao, Shandong province, China
			Clinocardium californiense	JN860024	Dalian, Liaoning province, China
			Clinocardium californiense	JN860025	Wenchang, Hainan province, China
			Clinocardium californiense	JN860026	Beibhai, Guangxi province, China
			Clinocardium californiense	JN860027	Rongcheng, Shandong province, China
		Vasticardium	*Vasticardium flavum*	JN860016	Lingshui, Hainan province, China
			Vasticardium flavum	JN860017	Lingshui, Hainan province, China
			Vasticardium flavum	JN860018	Sanya, Hainan province, China
			Vasticardium flavum	JN860019	Lingao, Hainan province, China
			Vasticardium flavum	JN860020	Wenchang, Hainan province, China
			Vasticardium flavum	JN860021	Lingao, Hainan province, China
		Vepricardium	*Vepricardium coronatum*	JN860013	Sanya, Hainan province, China
			Vepricardium coronatum	JN860014	Sanya, Hainan province, China
			Vepricardium coronatum	JN860015	Sanya, Hainan province, China
	Corbiculidae	*Corbicula*	*Corbicula fluminea*	KC211267	Japan
			Corbicula fluminea	KC211281	Japan
			Corbicula fluminea	KC211282	Japan
			Corbicula fluminea	KC211283	Japan
			Corbicula fluminea	KC211285	Japan
			Corbicula fluminea	KC211286	Japan

Corbicula japonica	AB498808	Niigata, Japan	
Corbicula japonica	AB498018	Ibaraki, Japan	
Corbicula japonica	AB845593	Fukuoka, Fukuoka,	Zuibaiji Riv, Japan
Corbicula japonica	AB971384	Japan	
Corbicula japonica	AB971385	Japan	
Corbicula japonica	AB971386	Japan	
Corbicula japonica	AB971387	Japan	
Corbicula japonica	AB971388	Japan	
Corbicula japonica	AB971389	Japan	
Corbicula japonica	AB971390	Japan	
Corbicula japonica	AB971391	Japan	
Corbicula japonica	AB971392	Japan	
Corbicula japonica	AB971393	Japan	
Corbicula japonica	AB971394	Japan	
Corbicula japonica	AB971395	Japan	
Corbicula japonica	AB971396	Japan	
Corbicula japonica	AB971397	Japan	
Corbicula japonica	AB971398	Japan	
Corbicula japonica	AB971399	Japan	
Corbicula japonica	AB971400	Japan	
Corbicula japonica	AB971401	Japan	
Corbicula japonica	AB971402	Japan	
Corbicula japonica	AB971403	Japan	
Corbicula japonica	AB971404	Japan	
Corbicula japonica	AB971405	Japan	
Corbicula japonica	AB971406	Japan	
Corbicula japonica	AB971407	Japan	
Corbicula japonica	AB971408	Japan	
Corbicula japonica	KC211253	Japan	
Corbicula japonica	KC211252	Japan	
Corbicula japonica	KC211254	Japan	

		Corbicula japonica	KC211255	Japan
		Corbicula japonica	KC211256	Japan
		Corbicula japonica	KC211257	Japan
		Corbicula japonica	KC211258	Japan
		Corbicula japonica	KC211259	Japan
		Corbicula japonica	KC211271	Japan
		Corbicula japonica	KC211272	Japan
		Corbicula japonica	KC211273	Japan
		Corbicula japonica	KC211274	Japan
		Corbicula japonica	KC211275	Japan
		Corbicula japonica	KC211276	Japan
		Corbicula leana	KC211268	Japan
		Corbicula leana	KC211269	Japan
		Corbicula leana	KC211270	Japan
		Corbicula leana	KC211287	Japan
		Corbicula leana	KC211288	Japan
		Corbicula leana	KC211289	Japan
		Corbicula leana	AB498810	Fukuoka, Chikugo River, Japan
		Corbicula leana	AB845591	Hyogo, minami-awaji, Hatsuo Riv, Japan
		Corbicula sandai	AB845590	Shiga, Hikone, Lake Biwa, Japan
		Corbicula sandai	KC211277	Japan
		Corbicula sandai	KC211278	Japan
		Corbicula sandai	KC211279	Japan
		Corbicula sandai	KC211280	Japan
		Corbicula sandai	AB498811	Shiga, Lake Biwa, Japan
	Geloina	*Geloina erosa*	AB076927	Okinawa, Iriomote, Okinawa, Japan
		Geloina expansa	AB498812	Okinawa, Iriomote Island, Okinawa, Japan
Cultellidae	*Cultellus*	*Cultellus attenuatus*	JN859998	Shengsi, Zhejiang province, China
		Cultellus attenuatus	JN859999	Qingdao, Shandong province, China
		Cultellus attenuatus	JN860000	Changzhou, Fujian province, China
		Cultellus attenuatus	JN860001	Qingdao, Shandong province, China

Donacidae	*Donax*	*Donax dysoni*	JN859976	Wenchang, Hainan province, China
		Donax dysoni	JN859977	Wenchang, Hainan province, China
		Donax dysoni	JN859978	Beibhai, Guangxi province, China
		Donax dysoni	JN859979	Beibhai, Guangxi province, China
		Donax dysoni	JN859980	Wenchang, Hainan province, China
		Donax dysoni	JN859981	Fangchenggang, Guangxi province, China
Mactridae	*Coelomactra*	*Coelomactra antiquata*	JN674607	Changzhou, Fujian province, China
		Coelomactra antiquata	JN674608	Pingtan, Fujian province, China
		Coelomactra antiquata	JN674609	Lianyungang, Jiangsu province, China
	Lutraria	*Lutraria arcuata*	JN674601	Beibhai, Guangxi province, China
		Lutraria arcuata	JN674602	Changzhou, Fujian province, China
		Lutraria arcuata	JN674603	Beibhai, Guangxi province, China
		Lutraria australis	JN674600	Beibhai, Guangxi province, China
	Mactra	*Mactra alta*	JN674615	Beibhai, Guangxi province, China
		Mactra alta	JN674616	Beibhai, Guangxi province, China
		Mactra alta	JN674617	Beibhai, Guangxi province, China
		Mactra alta	JN674618	Beibhai, Guangxi province, China
		Mactra alta	JN674619	Beibhai, Guangxi province, China
		Mactra alta	JN674620	Beibhai, Guangxi province, China
		Mactra chinensis	JN674630	Pingtan, Fujian province, China
		Mactra chinensis	JN674631	Lianyungang, Jiangsu province, China
		Mactra chinensis	JN674632	Wendeng, Shandong province, China
		Mactra chinensis	JN674633	Qinhuangdao, Hebei province, China
		Mactra chinensis	JN674634	Dandong, Liaoning province, China
		Mactra chinensis	JN674635	Nanji, Zhejiang province, China
		Mactra chinensis	KC205870	China
		Mactra chinensis	KC205871	China
		Mactra chinensis	KC205872	China
		Mactra chinensis	KC205873	China
		Mactra chinensis	KC205874	China
		Mactra chinensis	KC205875	China

		Mactra chinensis	KC205876	China
		Mactra chinensis	KC205877	China
		Mactra mauclata	JN674613	Wenchang, Hainan province, China
		Mactra mauclata	JN674614	Wenchang, Hainan province, China
		Mactra veneriformis	JN674621	Lianyungang, Jiangsu province, China
		Mactra veneriformis	JN674622	Zhoushan, Zhejiang province, China
		Mactra veneriformis	JN674623	Beibhai, Guangxi province, China
		Mactra veneriformis	JN674624	Dalian, Liaoning province, China
		Mactra veneriformis	JN674625	Dongying, Shandong province, China
		Mactra veneriformis	GQ864250	Lianyungang, Jiangsu province, China
		Mactra veneriformis	FJ851355	Qidong, Jiangsu province, China
		Mactra veneriformis	GQ864249	Lianyungang, Jiangsu province, China
		Mactra veneriformis	GQ864248	Lianyungang, Jiangsu province, China
		Mactra veneriformis	GQ864247	Lianyungang, Jiangsu province, China
		Mactra veneriformis	GQ864246	Lianyungang, Jiangsu province, China
		Mactra veneriformis	GQ864245	Lianyungang, Jiangsu province, China
		Mactra veneriformis	GQ864244	Lianyungang, Jiangsu province, China
		Mactra veneriformis	GQ864243	Lianyungang, Jiangsu province, China
		Mactra veneriformis	GQ864242	Lianyungang, Jiangsu province, China
		Mactra veneriformis	GQ864241	Lianyungang, Jiangsu province, China
		Mactra veneriformis	GQ864240	Lianyungang, Jiangsu province, China
		Mactra veneriformis	GQ864239	Lianyungang, Jiangsu province, China
		Mactra veneriformis	GQ864238	Lianyungang, Jiangsu province, China
		Mactra cumingii	JN674610	Sanya, Hainan province, China
		Mactra cumingii	JN674611	Sanya, Hainan province, China
		Mactra cumingii	JN674612	Sanya, Hainan province, China
	Pseudocardium	*Pseudocardium sachalinensis*	JN674604	Beibhai, Guangxi province, China
		Pseudocardium sachalinensis	JN674605	Beibhai, Guangxi province, China
Pharidae	*Siliqua*	*Siliqua radiata*	JN860002	Changzhou, Fujian province, China

		Siliqua radiata	JN860003	Changzhou, Fujian province, China
		Siliqua radiata	JN860004	Changzhou, Fujian province, China
		Siliqua radiata	JN860005	Changzhou, Fujian province, China
Psammobiidae	*Sanguinolaria*	*Sanguinolaria tchangsii*	JN859945	Lingao, Hainan province, China
		Sanguinolaria tchangsii	JN859947	Lingao, Hainan province, China
	Nuttallia	*Nuttallia olivacea*	JN859954	NanjiZhejiang, ProvinceChina
		Nuttallia olivacea	JN859955	NanjiZhejiang, ProvinceChina
	Soletellina	*Soletellina virescens*	JN859943	Beihai, Guangxi province, China
		Soletellina diphos	JN859948	Lingao, Hainan province, China
	Psammotaea	*Psammotaea elongata*	JN859936	Beihai, Guangxi Province, China
		Psammotaea elongata	JN859938	Beihai, Guangxi province, China
		Psammotaea elongata	JN859940	Wenchang, Hainan province, China
		Psammotaea elongata	JN859941	Beihai, Guangxi province, China
Semelidae	*Semele*	*Semele cf amabilis*	JN859975	Lingao, Hainan province, China
		Semele scaba	JN859973	Lingshui, Hainan province, China
		Semele scaba	JN859974	Lingshui, Hainan province, China
Solecurtidae	*Sinonovacula*	*Sinonovacula constricta*	JN859986	Beibhai, Guangxi province, China
		Sinonovacula constricta	JN859987	Lianyungang, Jiangsu province, China
		Sinonovacula constricta	JN859988	Panjin, Liaoning province, China
		Sinonovacula constricta	JN859989	Xiapu, Fujian province, China
		Sinonovacula constricta	JN859991	Shantou, Guangdong province, China
		Sinonovacula constricta	JN859993	Zhoushan, Zhejiang province, China
		Sinonovacula constricta	HM180885	Korea
		Sinonovacula constricta	HM180886	Korea
	Solecurtus	*Solecurtus divaricatus*	JN859983	Yangjiang, Guangdong province, China
Solenidae	*Solen*	*Solen grandis*	JN860010	Changzhou, Fujian province, China
		Solen grandis	JN860011	Qingdao, Shandong province, China
		Solen grandis	JN860012	Fenshui, Shandong province, China
		Solen strictus	JN860006	Fangchenggang, Guangxi province, China
		Solen strictus	JN860007	Beibhai, Guangxi province, China
		Solen strictus	JN860008	Dandong, Liaoning province, China

		Solen strictus	JN860009	Weihai, Shandong province, China
Tellinidae	*Macoma*	*Macoma candida*	JN859965	Beibhai, Guangxi province, China
		Macoma tokyoensis	JN859962	Rongcheng, Shandong province, China
		Macoma tokyoensis	JN859963	Rongcheng, Shandong province, China
	Moerella	*Moerella iridescens*	JN859970	Beihai, Guangxi province, China
	Serratina	*Serratina capsoides*	AB691950	Fukuoka, Imazu bay, Japan
		Serratina capsoides	AB691949	Kumamoto,Yokaku-bay, Japan
		Serratina capsoides	AB691948	Mie, Japan
		Serratina capsoides	AB691947	Okinawa, Sashiki, Okinawa, Japan
		Serratina capsoides	AB691946	Kagoshima Pref, Amami-Oshima, Japan
		Serratina capsoides	AB691945	Okinawa, Haneji-Naikai, Okinawa, Japan
		Serratina capsoides	JN859959	Beibhai, Guangxi province, China
		Serratina capsoides	JN859960	Beibhai, Guangxi province, China
Veneridae	*Anomalocardia*	*Anomalocardia producta*	HQ703052	Wenchang, Hainan province, China
		Anomalocardia producta	HQ703053	Wenchang, Hainan province, China
		Anomalocardia producta	HQ703054	Beibhai, Guangxi province, China
		Anomalocardia producta	HQ703055	Sanya, Hainan province, China
		Anomalocardia producta	HQ703056	Beibhai, Guangxi province, China
	Anomalodiscus	*Anomalodiscus squamosus*	HQ703048	Wenchang, Hainan province, China
		Anomalodiscus squamosus	HQ703049	Wenchang, Hainan province, China
		Anomalodiscus squamosus	HQ703050	Lingao, Hainan province, China
		Anomalodiscus squamosus	HQ703051	Sanya, Hainan province, China
		Anomalodiscus squamosus	HM124611	China
	Bonartemis	*Bonartemis histrio*	HQ703155	Beibhai, Guangxi province, China
		Bonartemis histrio	HQ703156	Beibhai, Guangxi province, China
	Callista	*Callista brevisiphonata*	HQ703037	Qingdao, Shandong province, China
		Callista brevisiphonata	HQ703038	Qingdao, Shandong province, China
		Callista brevisiphonata	HQ703039	Qingdao, Shandong province, China
		Callista brevisiphonata	HQ703040	Qingdao, Shandong province, China
		Callista brevisiphonata	JN898931	China
		Callista brevisiphonata	HM124569	China

	Callista chinensis	HQ703035	Pingtan, Fujian province, China
	Callista chinensis	HQ703036	Pingtan, Fujian province, China
Circe	*Circe scripta*	HQ703107	Beibhai, Guangxi province, China
	Circe scripta	HQ703108	Lingao, Hainan province, China
	Circe scripta	HQ703109	Lingao, Hainan province, China
	Circe scripta	HQ703110	Lingao, Hainan province, China
	Circe scripta	HQ703111	Beibhai, Guangxi province, China
	Circe scripta	HQ703112	Lingao, Hainan province, China
	Circe scripta	HQ703113	Sanya, Hainan province, China
	Circe scripta	HQ703114	Lingshui, Hainan province, China
Placamen	*Placamen calophylla*	HQ703062	Beibhai, Guangxi province, China
	Placamen calophylla	HQ703063	Sanya, Hainan province, China
	Placamen calophylla	HQ703064	Fangchenggang, Guangxi province, China
	Placamen isabellina	HQ703057	Beibu Bay, Guangxi province, China
	Placamen isabellina	HQ703058	Beibu Bay, Guangxi province, China
	Placamen isabellina	HQ703059	Beibu Bay, Guangxi province, China
	Placamen isabellina	HQ703060	Beibhai, Guangxi province, China
	Placamen isabellina	HQ703061	Beibhai, Guangxi province, China
Costacallista	*Costacallista erycina*	HQ703031	Beibhai, Guangxi province, China
	Costacallista erycina	HQ703032	Beibhai, Guangxi province, China
	Costacallista erycina	HQ703033	Beibhai, Guangxi province, China
	Costacallista erycina	HQ703034	Beibhai, Guangxi province, China
	Costacallista erycina	JN898943	China
	Costacallista erycina	HM124570	China
	Costacallista erycina	EU117993	China
Cyclina	*Cyclina sinensis*	HM021149	Japan
	Cyclina sinensis	HM021148	Japan
	Cyclina sinensis	HM021147	Japan
	Cyclina sinensis	HQ703115	Zhoushan, Zhejiang province, China
	Cyclina sinensis	HQ703116	Shengsi, Zhejiang province, China
	Cyclina sinensis	HQ703117	Yueqing, Zhejiang province, China

	Cyclina sinensis	HQ703118	Dandong, Liaoning province, China
	Cyclina sinensis	HQ703119	Qinhuangdao, Hebei province, China
	Cyclina sinensis	HQ703120	Panjin, Liaoning province, China
	Cyclina sinensis	HQ703121	Dongxing, Guangxi province, China
	Cyclina sinensis	HQ703122	Jimo, Shandong province, China
	Cyclina sinensis	HQ703123	Lianyungang, Jiangsu province, China
	Cyclina sinensis	HQ703124	Lvshun, Liaoning province, China
	Cyclina sinensis	HQ703125	Maoming, Guangdong province, China
	Cyclina sinensis	HQ703126	Qidong, Jiangsu province, China
	Cyclina sinensis	HQ703127	Sanya, Hainan province, China
	Cyclina sinensis	HQ703128	Tanggu, Tianjin province, China
	Cyclina sinensis	HQ703129	Changyi, Shandong province, China
	Cyclina sinensis	HQ703130	Xiamen, Fujian province, China
	Cyclina sinensis	HQ703131	Xiangshui, Jiangsu province, China
Sunetta	*Sunetta concinna*	JN898937	China
	Sunetta menstrualis	HQ703213	Fenshui, Shandong province, China
	Sunetta menstrualis	HQ703214	Fenshui, Shandong province, China
	Sunetta menstrualis	HQ703215	Fenshui, Shandong province, China
	Sunetta menstrualis	HQ703216	Fenshui, Shandong province, China
	Sunetta menstrualis	HM124589	China
	Sunetta menstrualis	HM124588	China
Dosinia	*Dosinia angulosa*	HQ703148	Haikou, Hainan province, China
	Dosinia angulosa	HQ703149	Haikou, Hainan province, China
	Dosinia angulosa	HQ703150	Haikou, Hainan province, China
	Dosinia angulosa	HQ703151	Haikou, Hainan province, China
	Dosinia biscocta	HM124577	China
	Dosinia biscocta	HM124572	China
	Dosinia corrugata	HQ703141	Ganyu, Jiangsu province, China
	Dosinia corrugata	HQ703142	Ganyu, Jiangsu province, China
	Dosinia corrugata	HQ703143	Dalian, Liaoning province, China
	Dosinia corrugata	HQ703144	Dalian, Liaoning province, China

	Dosinia corrugata	HQ703145	Dalian, Liaoning province, China
	Dosinia corrugata	HQ703146	Dalian, Liaoning province, China
	Dosinia corrugata	HQ703147	Weihai, Shandong province, China
	Dosinia corrugata	JN898932	China
	Dosinia corrugata	HM124573	China
	Dosinia fibula	HQ703132	Fenshui, Shandong province, China
	Dosinia fibula	HQ703133	Changzhou, Fujian province, China
	Dosinia fibula	HQ703134	Ganyu, Jiangsu province, China
	Dosinia fibula	HQ703135	Beibhai, Guangxi province, China
	Dosinia fibula	HQ703136	Beibhai, Guangxi province, China
	Dosinia japonicum	HQ703137	Qingdao, Shandong province, China
	Dosinia japonicum	HQ703138	Qingdao, Shandong province, China
	Dosinia troscheli	HQ703139	Sanya, Hainan province, China
	Dosinia troscheli	HQ703140	Changzhou, Fujian province, China
Gafrarium	*Gafrarium dispar*	HQ703086	Lingao, Hainan province, China
	Gafrarium dispar	HQ703087	Lingao, Hainan province, China
	Gafrarium dispar	HQ703088	Lingao, Hainan province, China
	Gafrarium dispar	HQ703089	Lingao, Hainan province, China
	Gafrarium dispar	HQ703090	Lingao, Hainan province, China
	Gafrarium dispar	HQ703091	Wenchang, Hainan province, China
	Gafrarium dispar	HQ703092	Weizhou, Guangxi province, China
	Gafrarium dispar	HQ703093	Sanya, Hainan province, China
	Gafrarium dispar	HQ703094	Weizhou, Guangxi province, China
	Gafrarium divaricatum	HQ703095	Lingao, Hainan province, China
	Gafrarium divaricatum	HQ703096	Lingao, Hainan province, China
	Gafrarium divaricatum	HQ703097	Zhanjiang, Guangdong province, China
	Gafrarium divaricatum	HQ703098	Zhanjiang, Guangdong province, China
	Gafrarium divaricatum	HQ703099	Zhanjiang, Guangdong province, China
	Gafrarium divaricatum	HQ703100	Zhanjiang, Guangdong province, China
	Gafrarium divaricatum	HQ703101	Beibhai, Guangxi province, China
	Gafrarium divaricatum	HQ703102	Beibhai, Guangxi province, China

	Gafrarium divaricatum	HQ703103	Pingtan, Fujian province, China
	Gafrarium divaricatum	HQ703104	Beibhai, Guangxi province, China
	Gafrarium divaricatum	HQ703105	Beibhai, Guangxi province, China
	Gafrarium divaricatum	HQ703106	Maoming, Guangdong province, China
	Gafrarium pectinatum	HQ703077	Wenchang, Hainan province, China
	Gafrarium pectinatum	HQ703078	Wenchang, Hainan province, China
	Gafrarium pectinatum	HQ703079	Sanya, Hainan province, China
	Gafrarium pectinatum	HQ703080	Qionghai, Hainan province, China
	Gafrarium tumidum	HQ703081	Wenchang, Hainan province, China
	Gafrarium tumidum	HQ703082	Wenchang, Hainan province, China
	Gafrarium tumidum	HQ703083	Lingao, Hainan province, China
	Gafrarium tumidum	HQ703084	Sanya, Hainan province, China
	Gafrarium tumidum	HQ703085	Qionghai, Hainan province, China
Antigona	*Antigona lamellaris*	HQ703334	Sanya, Hainan province, China
	Antigona lamellaris	HQ703335	Sanya, Hainan province, China
	Antigona lamellaris	HQ703336	Sanya, Hainan province, China
	Antigona lamellaris	HQ703337	Beibhai, Guangxi province, China
	Antigona lamellaris	HQ703338	Beibhai, Guangxi province, China
	Antigona lamellaris	HQ703339	Beibhai, Guangxi province, China
	Antigona lamellaris	HQ703340	Beibhai, Guangxi province, China
	Antigona lamellaris	HQ703341	Beibhai, Guangxi province, China
	Antigona lamellaris	HM124608	China
Globivenus	*Globivenus toreuma*	HQ703342	Lingao, Hainan province, China
Macridiscus	*Macridiscus aequilatera*	HQ703266	Haiyang, Shandong province, China
	Macridiscus aequilatera	HQ703267	Haiyang, Shandong province, China
	Macridiscus aequilatera	HQ703268	Haiyang, Shandong province, China
	Macridiscus aequilatera	HQ703269	Haiyang, Shandong province, China
	Macridiscus aequilatera	HQ703270	Haiyang, Shandong province, China
	Macridiscus aequilatera	HQ703271	Haiyang, Shandong province, China
	Macridiscus aequilatera	HQ703272	Haiyang, Shandong province, China
	Macridiscus aequilatera	GQ855272	Yangjiang, Guangdong province, China

Macridiscus aequilatera	GQ855273	Beibhai, Guangxi province, China
Macridiscus aequilatera	GQ855274	Beibhai, Guangxi province, China
Macridiscus aequilatera	GQ855275	Beibhai, Guangxi province, China
Macridiscus aequilatera	GQ855276	Zhoushan, Zhejiang province, China
Macridiscus melanaegis	HQ703273	Weihai, Shandong province, China
Macridiscus melanaegis	HQ703274	Weihai, Shandong province, China
Macridiscus multifarius	HQ224672	China
Macridiscus multifarius	HQ224673	China
Macridiscus multifarius	HQ224674	China
Macridiscus multifarius	HQ224675	China
Macridiscus multifarius	HQ224676	China
Macridiscus multifarius	HQ224677	China
Macridiscus multifarius	HQ224678	China
Macridiscus multifarius	HQ224679	China
Macridiscus semicancellata	HQ703275	Yangjiang, Guangdong province, China
Macridiscus semicancellata	HQ703276	Beibhai, Guangxi province, China
Macridiscus semicancellata	HQ703277	Beibhai, Guangxi province, China
Macridiscus semicancellata	HQ703278	Beibhai, Guangxi province, China
Macridiscus semicancellata	HQ703279	Beibhai, Guangxi province, China
Macridiscus semicancellata	HQ703280	Beibhai, Guangxi province, China
Macridiscus semicancellata	HQ703281	Pingtan, Fujian province, China
Macridiscus semicancellata	HQ224686	China
Macridiscus	HQ224687	China

	semicancellata		
	Macridiscus semicancellata	HQ224688	China
	Macridiscus semicancellata	HQ224689	China
	Macridiscus semicancellata	HQ224690	China
	Macridiscus semicancellata	HQ224692	China
	Macridiscus semicancellata	HQ224693	China
	Macridiscus semicancellata	HQ224694	China
Katelysia	*Katelysia hiantina*	JN898939	China
	Katelysia hiantina	HM124599	China
	Katelysia hiantina	HQ703287	Lingshui, Hainan province, China
	Katelysia hiantina	HQ703288	Zhanjiang, Guangdong province, China
	Katelysia hiantina	HQ703289	Maoming, Guangdong province, China
	Katelysia hiantina	HQ703290	Beibhai, Guangxi province, China
	Katelysia hiantina	HQ703291	Beibhai, Guangxi province, China
	Katelysia hiantina	HQ703292	Sanya, Hainan province, China
	Katelysia hiantina	HQ703293	Sanya, Hainan province, China
	Katelysia hiantina	HQ703294	Sanya, Hainan province, China
	Katelysia hiantina	GQ855255	Beibhai, Guangxi province, China
	Katelysia hiantina	GQ855256	Sanya, Hainan province, China
	Katelysia hiantina	GQ855257	Sanya, Hainan province, China
Marcia	*Marcia japonica*	HQ703282	Sanya, Hainan province, China
	Marcia japonica	HQ703283	Sanya, Hainan province, China
	Marcia japonica	HQ703284	Sanya, Hainan province, China
	Marcia japonica	HQ703285	Wenchang, Hainan province, China
	Marcia japonica	HQ703286	Wenchang, Hainan province, China

	Marcia japonica	GQ855258	Sanya, Hainan province, China
	Marcia japonica	GQ855259	Sanya, Hainan province, China
	Marcia japonica	GQ855260	Sanya, Hainan province, China
	Marcia japonica	GQ855261	Wenchang, Hainan province, China
	Marcia japonica	GQ855262	Wenchang, Hainan province, China
	Marcia marmorata	HQ703296	Sanya, Hainan province, China
	Marcia marmorata	HQ703297	Sanya, Hainan province, China
	Marcia marmorata	HQ703298	Beibhai, Guangxi province, China
	Marcia marmorata	HQ703299	Beibhai, Guangxi province, China
	Marcia marmorata	HQ703300	Beibhai, Guangxi province, China
	Marcia marmorata	HQ703301	Beibhai, Guangxi province, China
	Marcia marmorata	HQ703302	Beibhai, Guangxi province, China
	Marcia marmorata	HQ703303	Baimajing, Hainan province, China
Mercenaria	*Mercenaria mercenaria*	AB685212	Tokyo Bay, Japan
	Mercenaria mercenaria	AB685211	Tokyo Bay, Japan
	Mercenaria mercenaria	AB685210	Tokyo Bay, Japan
	Mercenaria mercenaria	AB685209	Tokyo Bay, Japan
	Mercenaria mercenaria	AB685208	Tokyo Bay, Japan
	Mercenaria mercenaria	AB685207	Tokyo Bay, Japan
	Mercenaria mercenaria	AB685206	Tokyo Bay, Japan
	Mercenaria mercenaria	AB685205	Tokyo Bay, Japan
	Mercenaria mercenaria	AB685204	Tokyo Bay, Japan
	Mercenaria mercenaria	AB685203	Tokyo Bay, Japan
	Mercenaria mercenaria	AB685202	Tokyo Bay, Japan
	Mercenaria mercenaria	AB685201	Tokyo Bay, Japan
	Mercenaria mercenaria	AB685200	Tokyo Bay, Japan
	Mercenaria mercenaria	AB685199	Tokyo Bay, Japan
	Mercenaria mercenaria	AB685198	Tokyo Bay, Japan
	Mercenaria mercenaria	AB685197	Tokyo Bay, Japan
	Mercenaria mercenaria	AB685196	Tokyo Bay, Japan
	Mercenaria mercenaria	AB685195	Tokyo Bay, Japan

	Mercenaria mercenaria	AB685194	Tokyo Bay, Japan
	Mercenaria mercenaria	AB685193	Tokyo Bay, Japan
	Mercenaria mercenaria	AB670734	Tokyo Bay, Japan
	Mercenaria mercenaria	AB670733	Tokyo Bay, Japan
	Mercenaria mercenaria	AB670732	Tokyo Bay, Japan
	Mercenaria mercenaria	AB670731	Tokyo Bay, Japan
	Mercenaria mercenaria	AB670730	Tokyo Bay, Japan
	Mercenaria mercenaria	AB670729	Tokyo Bay, Japan
	Mercenaria mercenaria	AB670728	Tokyo Bay, Japan
	Mercenaria mercenaria	AB670727	Tokyo Bay, Japan
	Mercenaria mercenaria	AB670726	Tokyo Bay, Japan
	Mercenaria mercenaria	AB670725	Tokyo Bay, Japan
	Mercenaria mercenaria	AB670724	Tokyo Bay, Japan
	Mercenaria mercenaria	AB670723	Tokyo Bay, Japan
	Mercenaria mercenaria	AB670722	Tokyo Bay, Japan
	Mercenaria mercenaria	AB670721	Tokyo Bay, Japan
	Mercenaria mercenaria	AB670720	Tokyo Bay, Japan
	Mercenaria mercenaria	AB670719	Tokyo Bay, Japan
	Mercenaria mercenaria	HQ703071	Lianyungang, Jiangsu province, China
	Mercenaria mercenaria	HQ703072	Lianyungang, Jiangsu province, China
	Mercenaria mercenaria	HQ703073	Lianyungang, Jiangsu province, China
	Mercenaria mercenaria	HQ703074	Lianyungang, Jiangsu province, China
	Mercenaria mercenaria	HQ703075	Lianyungang, Jiangsu province, China
	Mercenaria mercenaria	HQ703076	Lianyungang, Jiangsu province, China
	Mercenaria mercenaria	JN898950	China
	Mercenaria mercenaria	HM124619	China
	Mercenaria mercenaria	HM124618	China
	Mercenaria mercenaria	DQ399403	China
Meretrix	*Meretrix lamarckii*	HQ703188	Haikou, Hainan province, China
	Meretrix lamarckii	HQ703189	Haikou, Hainan province, China
	Meretrix lamarckii	HQ703190	Haikou, Hainan province, China

Meretrix lamarckii	HQ703191	Sanya, Hainan province, China
Meretrix lamarckii	HM124579	China
Meretrix lamarckii	JN043625	China
Meretrix lusoria	JN043624	China
Meretrix lusoria	JN898936	China
Meretrix lusoria	FJ434681	China
Meretrix lusoria	JN898935	China
Meretrix lusoria	AB853864	Aomori, Mutsu, Japan
Meretrix lusoria	AB853865	Kumamoto, Midorikawa, Japan
Meretrix lusoria	AB613023	Kyoto, Asokai Lagoon, Japan
Meretrix lusoria	AB613022	Kyoto, Asokai Lagoon, Japan
Meretrix lusoria	AB076924	Aich, Yokkaichi, Japan
Meretrix lusoria	AB280786	Oita, Japan
Meretrix lyrata	HQ703192	Zhangpu, Fujian province, China
Meretrix lyrata	HQ703193	Zhangpu, Fujian province, China
Meretrix lyrata	HQ703194	Zhanjiang, Guangdong province, China
Meretrix lyrata	HQ703195	Sanya, Hainan province, China
Meretrix lyrata	HQ703196	Sanya, Hainan province, China
Meretrix lyrata	HQ703197	Beibhai, Guangxi province, China
Meretrix lyrata	HQ703198	Wenchang, Hainan province, China
Meretrix lyrata	JN043622	China
Meretrix lyrata	JN898944	China
Meretrix lyrata	HM124581	China
Meretrix meretrix	HQ703159	Lingao, Hainan province, China
Meretrix meretrix	HQ703160	Lingao, Hainan province, China
Meretrix meretrix	HQ703161	Lingao, Hainan province, China
Meretrix meretrix	HQ703162	Beibhai, Guangxi province, China
Meretrix meretrix	HQ703163	Weizhou, Guangxi province, China
Meretrix meretrix	JN898949	China
Meretrix meretrix	HM124578	China
Meretrix meretrix	DQ399398	China

Meretrix meretrix	JN043623	China
Meretrix meretrix	DQ399399	China
Meretrix meretrix	DQ399400	China
Meretrix meretrix	DQ399401	China
Meretrix meretrix	DQ399402	China
Meretrix petechialis	HQ703164	Panjin, Liaoning province, China
Meretrix petechialis	HQ703165	Panjin, Liaoning province, China
Meretrix petechialis	HQ703166	Ganyu, Jiangsu province, China
Meretrix petechialis	HQ703167	Ganyu, Jiangsu province, China
Meretrix petechialis	HQ703168	Ganyu, Jiangsu province, China
Meretrix petechialis	HQ703169	Nanyang, Jiangsu province, China
Meretrix petechialis	HQ703170	Nanyang, Jiangsu province, China
Meretrix petechialis	HQ703171	Nanyang, Jiangsu province, China
Meretrix petechialis	HQ703172	Fenshui, Shandong province, China
Meretrix petechialis	HQ703173	Sanya, Hainan province, China
Meretrix petechialis	HQ703174	Xiamen, Fujian province, China
Meretrix petechialis	HQ703175	Xiamen, Fujian province, China
Meretrix petechialis	HQ703176	Zhanjiang, Guangdong province, China
Meretrix petechialis	HQ703177	Zhanjiang, Guangdong province, China
Meretrix petechialis	HQ703178	Beibhai, Guangxi province, China
Meretrix petechialis	HQ703179	Beibhai, Guangxi province, China
Meretrix petechialis	HQ703180	Shantou, Guangdong province, China
Meretrix petechialis	HQ703181	Haikou, Hainan province, China
Meretrix petechialis	HQ703182	Haikou, Hainan province, China
Meretrix petechialis	HQ703183	Lingao, Hainan province, China
Meretrix petechialis	HQ703184	Yueqing, Zhejiang province, China
Meretrix petechialis	HQ703185	Beibhai, Guangxi province, China
Meretrix petechialis	HQ703186	Beibhai, Guangxi province, China
Meretrix petechialis	HQ703187	Yangjiang, Guangdong province, China
Meretrix petechialis	HM124583	China
Meretrix petechialis	HM124582	China

	Meretrix petechialis	AB853869	China
	Meretrix petechialis	HM124584	China
Paphia	*Paphia amabilis*	HQ703249	Beibhai, Guangxi province, China
	Paphia amabilis	HQ703250	Beibhai, Guangxi province, China
	Paphia amabilis	HQ703251	Beibhai, Guangxi province, China
	Paphia amabilis	HQ703252	Beibhai, Guangxi province, China
	Paphia amabilis	HQ703253	Beibhai, Guangxi province, China
	Paphia amabilis	HQ703254	Beibhai, Guangxi province, China
	Paphia amabilis	HQ703255	Beibhai, Guangxi province, China
	Paphia amabilis	HQ703256	Wenchang, Hainan province, China
	Paphia amabilis	HQ703257	Sanya, Hainan province, China
	Paphia amabilis	HQ703258	Sanya, Hainan province, China
	Paphia amabilis	HQ703259	Sanya, Hainan province, China
	Paphia amabilis	HQ703260	Weizhou, Guangxi province, China
	Paphia amabilis	HQ703261	Weizhou, Guangxi province, China
	Paphia amabilis	HQ703262	Weizhou, Guangxi province, China
	Paphia amabilis	GQ855251	Beibhai, Guangxi province, China
	Paphia amabilis	GQ855252	Sanya, Hainan province, China
	Paphia amabilis	GQ855253	Beibhai, Guangxi province, China
	Paphia amabilis	GQ855254	Sanya, Hainan province, China
	Paphia euglypta	DQ184827	Ueshima Island, off Murozumi, Yamaguchi Prefecture, Japan
	Paphia gallus	HQ703233	Baimajing, Hainan province, China
	Paphia gallus	HQ703234	Beibhai, Guangxi province, China
	Paphia gallus	HQ703235	Beibhai, Guangxi province, China
	Paphia gallus	GQ855248	Beibhai, Guangxi province, China
	Paphia gallus	GQ855249	Danzhou, Hainan province, China
	Paphia gallus	GQ855250	Beibhai, Guangxi province, China
	Paphia gallus	JQ277814	China
	Paphia gallus	HM124597	China
	Paphia gallus	JQ277813	China

Paphia gallus	JQ277812	China
Paphia gallus	JQ277811	China
Paphia gallus	JQ277810	China
Paphia gallus	JQ277809	China
Paphia gallus	JQ277808	China
Paphia gallus	JQ277807	China
Paphia gallus	JQ277806	China
Paphia gallus	JQ277805	China
Paphia gallus	JQ277804	China
Paphia gallus	JQ277803	China
Paphia papilionacea	HQ703238	Weihai, Shandong province, China
Paphia papilionacea	HQ703239	Weihai, Shandong province, China
Paphia papilionacea	HQ703240	Weihai, Shandong province, China
Paphia papilionacea	HQ703241	Weihai, Shandong province, China
Paphia papilionacea	HQ703242	Qingdao, Shandong province, China
Paphia papilionacea	HQ703243	Qingdao, Shandong province, China
Paphia papilionacea	GQ855238	Weihai, Shandong province, China
Paphia papilionacea	GQ855239	Weihai, Shandong province, China
Paphia papilionacea	GQ855240	Weihai, Shandong province, China
Paphia papilionacea	GQ855241	Weihai, Shandong province, China
Paphia papilionacea	GQ855242	Qingdao, Shandong province, China
Paphia papilionacea	GQ855243	Qingdao, Shandong province, China
Paphia papilionacea	JN898946	China
Paphia papilionacea	HM124590	China
Paphia semirugata	HQ703263	Beibhai, Guangxi province, China
Paphia semirugata	HQ703264	Beibhai, Guangxi province, China
Paphia semirugata	HQ703265	Beibhai, Guangxi province, China
Paphia semirugata	HM124602	China
Paphia sinuosa	HQ703231	Pingtan, Fujian province, China
Paphia sinuosa	JQ277815	China
Paphia textile	HQ703236	Sanya, Hainan province, China

	Paphia textile	HQ703237	Beibhai, Guangxi province, China
	Paphia textile	JN898938	China
	Paphia undulata	HQ703244	Yangjiang, Guangdong province, China
	Paphia undulata	HQ703245	Fangchenggang, Guangxi province, China
	Paphia undulata	HQ703246	Fangchenggang, Guangxi province, China
	Paphia undulata	HQ703247	Beibhai, Guangxi province, China
	Paphia undulata	HQ703248	Shantou, Guangdong province, China
	Paphia undulata	GQ855244	Yangjiang, Guangdong province, China
	Paphia undulata	GQ855245	Fangchenggang, Guangxi province, China
	Paphia undulata	GQ855246	Beibhai, Guangxi province, China
	Paphia undulata	GQ855247	Fangchenggang, Guangxi province, China
	Paphia undulata	JN898933	China
	Paphia undulata	HM124591	China
	Paphia vernicosa	DQ184828	Fishing Nets, Iwaishima Island, Yamaguchi Prefecture, Japan
Pelecyora	*Pelecyora isocardia*	HQ703209	Qingdao, Shandong province, China
	Pelecyora isocardia	HQ703210	Fenshui, Shandong province, China
	Pelecyora trigona	HM124586	China
Periglypta	*Periglypta chemnitzii*	HQ703330	Beibhai, Guangxi province, China
	Periglypta chemnitzii	HQ703331	Beibhai, Guangxi province, China
	Periglypta chemnitzii	HQ703332	Beibhai, Guangxi province, China
	Periglypta chemnitzii	HQ703333	Beibhai, Guangxi province, China
	Periglypta chemnitzii	HM124614	China
	Periglypta compressa	HM124606	China
	Periglypta puerpera	HQ703318	Sanya, Hainan province, China
	Periglypta puerpera	HQ703319	Sanya, Hainan province, China
	Periglypta puerpera	HQ703320	Sanya, Hainan province, China
	Periglypta puerpera	HQ703321	Sanya, Hainan province, China
	Periglypta puerpera	HQ703322	Lingshui, Hainan province, China
	Periglypta puerpera	HQ703323	Wenchang, Hainan province, China
	Periglypta puerpera	HQ703324	Wenchang, Hainan province, China

	Periglypta puerpera	HQ703325	Qionghai, Hainan province, China
	Periglypta puerpera	HQ703326	Qionghai, Hainan province, China
	Periglypta puerpera	HQ703327	Qionghai, Hainan province, China
	Periglypta puerpera	HQ703328	Haikou, Hainan province, China
	Periglypta puerpera	HQ703329	Sanya, Hainan province, China
Pitarina	*Pitarina japonica*	HQ703206	Sanya, Hainan province, China
	Pitarina japonica	HQ703207	Lingao, Hainan province, China
	Pitarina striatum	HQ703203	Sanya, Hainan province, China
	Pitarina striatum	HQ703204	Sanya, Hainan province, China
	Pitarina striatum	HQ703205	Sanya, Hainan province, China
Protothaca	*Protothaca jedoensis*	HQ703065	Haiyang, Shandong province, China
	Protothaca jedoensis	HQ703066	Ganyu, Jiangsu province, China
	Protothaca jedoensis	HQ703067	Ganyu, Jiangsu province, China
	Protothaca jedoensis	HQ703068	Qingdao, Shandong province, China
	Protothaca jedoensis	HQ703069	Qingdao, Shandong province, China
	Protothaca jedoensis	HQ703070	Rushan, Shandong province, China
	Protothaca jedoensis	HM124607	China
	Protothaca jedoensis	HM124605	China
	Protothaca jedoensis	DQ399397	China
Ruditapes	*Ruditapes philippinarum*	HQ703304	Jimo, Shandong province, China
	Ruditapes philippinarum	HQ703305	Rongcheng, Shandong province, China
	Ruditapes philippinarum	HQ703306	Zhangpu, Fujian province, China
	Ruditapes philippinarum	HQ703307	Zhangpu, Fujian province, China
	Ruditapes philippinarum	HQ703308	Shenzhen, Guangdong province, China
	Ruditapes philippinarum	HQ703309	Ganyu, Jiangsu province, China
	Ruditapes philippinarum	HQ703310	Nanji, Zhejiang province, China
	Ruditapes philippinarum	HQ703311	Baimajing, Hainan province, China
	Ruditapes philippinarum	GQ855263	Qingdao, Shandong province, China
	Ruditapes philippinarum	GQ855264	Rongcheng, Shandong province, China
	Ruditapes philippinarum	GQ855265	Nanji, Zhejiang province, China
	Ruditapes philippinarum	GQ855266	Beibhai, Guangxi province, China

	Ruditapes philippinarum	GQ855267	Zhangpu, Fujian province, China
	Ruditapes philippinarum	JN898947	China
	Ruditapes philippinarum	HM124595	China
	Ruditapes philippinarum	AB244389	Kyoto, Miyazu Bay, Japan
	Ruditapes philippinarum	AB244390	Kyoto, Miyazu Bay, Japan
	Ruditapes philippinarum	AB244391	Kyoto, Miyazu Bay, Japan
	Ruditapes philippinarum	AB244392	Kumamoto, Ariake Sea, Japan
	Ruditapes philippinarum	AB244393	Kumamoto, Ariake Sea, Japan
	Ruditapes philippinarum	AB244394	Kumamoto, Ariake Sea, Japan
	Ruditapes philippinarum	AB244395	Kumamoto, Ariake Sea, Japan
	Ruditapes philippinarum	AB244396	Kumamoto, Ariake Sea, Japan
	Ruditapes philippinarum	AB244397	Hokkaido, Notsuke Bay, Hokkaido, Japan
	Ruditapes philippinarum	AB244398	Hokkaido, Notsuke Bay, Hokkaido, Japan
	Ruditapes philippinarum	AB244399	Hokkaido, Notsuke Bay, Hokkaido, Japan
	Ruditapes philippinarum	AB244400	Hokkaido, Notsuke Bay, Hokkaido, Japan
	Ruditapes philippinarum	AB244401	Hokkaido, Notsuke Bay, Hokkaido, Japan
	Ruditapes philippinarum	HM180833	Korea
	Ruditapes philippinarum	HM180834	Korea
	Ruditapes variegata	HQ703312	Beibhai, Guangxi province, China
	Ruditapes variegata	HQ703313	Beibhai, Guangxi province, China
	Ruditapes variegata	HQ703314	Weizhou, Guangxi province, China
	Ruditapes variegata	HQ703315	Sanya, Hainan province, China
	Ruditapes variegata	HQ703316	Sanya, Hainan province, China
	Ruditapes variegata	HQ703317	Weizhou, Guangxi province, China
	Ruditapes variegata	GQ855268	Beibhai, Guangxi province, China
	Ruditapes variegata	GQ855269	Weizhou, Guangxi province, China
	Ruditapes variegata	GQ855270	Danzhou, Hainan province, China
	Ruditapes variegata	GQ855271	Beibhai, Guangxi province, China
Saxidomus	*Saxidomus purpuratus*	HQ703041	Panjin, Liaoning province, China
	Saxidomus purpuratus	HQ703042	Panjin, Liaoning province, China
	Saxidomus purpuratus	HQ703043	Panjin, Liaoning province, China

	Saxidomus purpuratus	HQ703044	Panjin, Liaoning province, China
	Saxidomus purpuratus	HQ703045	Dalian, Liaoning province, China
	Saxidomus purpuratus	HQ703046	Dalian, Liaoning province, China
	Saxidomus purpuratus	HQ703047	Dalian, Liaoning province, China
	Saxidomus purpuratus	KP419933	China
	Saxidomus purpuratus	JN898951	China
	Saxidomus purpuratus	HM124571	China
	Saxidomus purpuratus	EU118007	China
Tapes	*Tapes dorsatus*	HQ703224	Hepu, Guangxi province, China
	Tapes dorsatus	HQ703225	Hepu, Guangxi province, China
	Tapes dorsatus	HQ703226	Hepu, Guangxi province, China
	Tapes dorsatus	HQ703227	Hepu, Guangxi province, China
	Tapes dorsatus	HQ703228	Beibhai, Guangxi province, China
	Tapes dorsatus	HQ703229	Beibhai, Guangxi province, China
	Tapes dorsatus	HQ703230	Beibhai, Guangxi province, China
	Tapes dorsatus	GQ855277	Beibhai, Guangxi province, China
	Tapes dorsatus	JN898942	China
	Tapes dorsatus	HM124594	China
	Tapes literatus	HQ703217	Sanya, Hainan province, China
	Tapes literatus	HQ703218	Lingshui, Hainan province, China
	Tapes literatus	HQ703219	Lingshui, Hainan province, China
	Tapes literatus	HQ703220	Wenchang, Hainan province, China
	Tapes literatus	HQ703221	Wenchang, Hainan province, China
	Tapes literatus	HQ703222	Sanya, Hainan province, China
	Tapes literatus	HQ703223	Sanya, Hainan province, China
	Tapes literatus	GQ855278	Sanya, Hainan province, China
	Tapes literatus	GQ855279	Lingshui, Hainan province, China
	Tapes literatus	GQ855280	Sanya, Hainan province, China
	Tapes literatus	JN898941	China
	Tapes literatus	HM124603	China
Venerupis	*Venerupis bruguieri*	DQ184829	Tanoura, Nagashima, Yamaguchi Prefecture, Japan

		Vesicomyidae	*Calyptogena*	*Calyptogena extenta*	AB479085	Kurile Trench, Japan
				Calyptogena kawamurai	AB479089	Suruga Bay, Nankai Trough, Okinawa Trough, Okinawa, Japan
				Calyptogena phaseoliformis	AB479088	Kurile Trench, Japan
Cephalopoda	Decapodiformes_incertae_sedis	Idiosepiidae	*Idiosepius*	*Idiosepius biserialis*	EU008955	Takasu, Japan
				Idiosepius biserialis	EU008954	Takasu, Japan
				Idiosepius biserialis	EU008953	Takasu, Japan
				Idiosepius paradoxus	EU008997	Ushimado, Japan
				Idiosepius paradoxus	EU008996	Ushimado, Japan
				Idiosepius paradoxus	EU008995	Ushimado, Japan
				Idiosepius paradoxus	EU008994	Ushimado, Japan
				Idiosepius paradoxus	EU008993	Ushimado, Japan
				Idiosepius paradoxus	EU008992	Ushimado, Japan
				Idiosepius paradoxus	EU008991	Ushimado, Japan
				Idiosepius paradoxus	EU008990	Ushimado, Japan
				Idiosepius paradoxus	EU008989	Seto Inland Sea, Japan
				Idiosepius paradoxus	EU008988	Seto Inland Sea, Japan
				Idiosepius paradoxus	EU008987	Okinawa Island, Okinawa, Japan
				Idiosepius paradoxus	EU008986	Okinawa Island, Okinawa, Japan
				Idiosepius paradoxus	EU008985	Nagoya, Japan
				Idiosepius paradoxus	EU008984	Nagoya, Japan
				Idiosepius paradoxus	EU008983	Nagoya, Japan
				Idiosepius paradoxus	EU008982	Nagoya, Japan
				Idiosepius paradoxus	EU008981	Nagoya, Japan
				Idiosepius paradoxus	EU008980	Nagoya, Japan
				Idiosepius paradoxus	EU008979	Nagoya, Japan
	Myopsida	Loliginidae	*Heterololigo*	*Heterololigo bleekeri*	AB573761	Japan
				Heterololigo bleekeri	AB573760	Japan
				Heterololigo bleekeri	AB573759	Japan
				Heterololigo bleekeri	AB573758	Japan

	Heterololigo bleekeri	AB573757	Japan
	Heterololigo bleekeri	AB573756	Japan
	Heterololigo bleekeri	AB573755	Japan
	Heterololigo bleekeri	AB573754	Japan
	Heterololigo bleekeri	AB441190	Japan
	Heterololigo bleekeri	AB441189	Japan
	Heterololigo bleekeri	AB441188	Japan
	Heterololigo bleekeri	AB441187	Japan
	Heterololigo bleekeri	AB441185	Japan
	Heterololigo bleekeri	AB441186	Japan
	Heterololigo bleekeri	AB441184	Japan
	Heterololigo bleekeri	AB441182	Japan
	Heterololigo bleekeri	AB441181	Japan
	Heterololigo bleekeri	AB441180	Japan
	Heterololigo bleekeri	AB441179	Japan
Sepioteuthis	*Sepioteuthis lessoniana*	HQ529540	Rizhao, Shandong province, China
	Sepioteuthis lessoniana	HQ529541	Rizhao, Shandong province, China
	Sepioteuthis lessoniana	HQ529542	Rizhao, Shandong province, China
	Sepioteuthis lessoniana	EU349466	Hong Kong, China
	Sepioteuthis lessoniana	AB986213	Kagoshima, Tanega-shima Island, Japan
	Sepioteuthis lessoniana	AB986214	Kagoshima, Tanega-shima Island, Japan
	Sepioteuthis lessoniana	AB986215	Kagoshima, Tanega-shima Island, Japan
	Sepioteuthis lessoniana	AB986216	Kagoshima, Tanega-shima Island, Japan
	Sepioteuthis lessoniana	AB986217	Kagoshima, Tanega-shima Island, Japan
	Sepioteuthis lessoniana	AB986218	Kagoshima, Tanega-shima Island, Japan
	Sepioteuthis lessoniana	AB986219	Kagoshima, Tanega-shima Island, Japan
	Sepioteuthis lessoniana	AB986220	Kagoshima, Tanega-shima Island, Japan
	Sepioteuthis lessoniana	AB986221	Tokushima, Mugi, Japan
	Sepioteuthis lessoniana	AB986222	Tokushima, Mugi, Japan
	Sepioteuthis lessoniana	AB986223	Tokushima, Mugi, Japan
	Sepioteuthis lessoniana	AB986224	Tokushima, Mugi, Japan

			Sepioteuthis lessoniana	AB986225	Tokushima, Mugi, Japan
			Sepioteuthis lessoniana	AB986226	Tokushima, Mugi, Japan
Octopoda	Octopodidae	*Amphioctopus*	*Amphioctopus aegina*	HQ846132	Lingao, Hainan province, China
			Amphioctopus aegina	HQ846133	Lingao, Hainan province, China
			Amphioctopus aegina	HQ846134	Lingao, Hainan province, China
			Amphioctopus aegina	HQ846135	Xiamen, Fujian province, China
			Amphioctopus aegina	HQ846136	Xiamen, Fujian province, China
			Amphioctopus aegina	HQ846137	Xiamen, Fujian province, China
			Amphioctopus aegina	JX456267	China
			Amphioctopus fangsiao	HQ846126	Xiamen, Fujian province, China
			Amphioctopus fangsiao	HQ846127	Xiamen, Fujian province, China
			Amphioctopus fangsiao	HQ846114	Lianyungang, Jiangsu province, China
			Amphioctopus fangsiao	HQ846155	Xiamen, Fujian province, China
			Amphioctopus fangsiao	AB430519	Hiroshima, Takehara, Japan
			Amphioctopus fangsiao	AB430518	East China Sea, China
			Amphioctopus kagoshimensis	HQ846122	Xiamen, Fujian province, China
			Amphioctopus kagoshimensis	HQ846123	Xiamen, Fujian province, China
			Amphioctopus kagoshimensis	HQ846124	Xiamen, Fujian province, China
			Amphioctopus kagoshimensis	HQ846125	Xiamen, Fujian province, China
			Amphioctopus kagoshimensis	AB430520	Kanagawa, Jogashima Island, Japan
			Amphioctopus marginatus	HQ846138	Xiamen, Fujian province, China
			Amphioctopus marginatus	HQ846139	Xiamen, Fujian province, China
			Amphioctopus marginatus	HQ846140	Xiamen, Fujian province, China
			Amphioctopus marginatus	HQ846141	Xiamen, Fujian province, China
			Amphioctopus marginatus	AB430522	Kanagawa, Ooiso, Japan
			Amphioctopus marginatus	AB430521	East China Sea, China

	Amphioctopus ovulum	AB430524	Kouchi, Japan
	Amphioctopus ovulum	AB430523	East China Sea, China
	Amphioctopus ovulum	HQ846156	Xiamen, Fujian province, China
	Amphioctopus ovulum	HQ846157	Xiamen, Fujian province, China
	Amphioctopus ovulum	HQ846158	Xiamen, Fujian province, China
	Amphioctopus ovulum	HQ846159	Xiamen, Fujian province, China
Callistoctopus	*Callistoctopus aspilosomatis*	AB430525	Okinawa, Miyagi Island, Okinawa, Japan
	Callistoctopus luteus	AB430526	Okinawa, Ohdo Beach, Okinawa, Japan
	Callistoctopus luteus	AB430527	Kanagawa, Miura, Japan
	Callistoctopus minor	AB430541	Ehime, Imabari, Miyakubo, Japan
	Callistoctopus minor	HQ846113	Rongcheng, Shandong province, China
	Callistoctopus minor	HQ846115	Rongcheng, Shandong province, China
	Callistoctopus minor	HQ846116	Rizhao, Shandong province, China
	Callistoctopus minor	HQ846117	Rizhao, Shandong province, China
	Callistoctopus minor	HQ846118	Dandong, Liaoning province, China
	Callistoctopus minor	HQ846119	Dandong, Liaoning province, China
	Callistoctopus ornatus	AB430528	Okinawa, Ohdo Beach, Okinawa, Japan
Cistopus	*Cistopus indicus*	JX456269	China
	Cistopus taiwanicus	HQ846142	Xiamen, Fujian province, China
	Cistopus taiwanicus	HQ846143	Xiamen, Fujian province, China
Hapalochlaena	*Hapalochlaena cf. maculosa*	HQ846163	Lingao, Hainan province, China
	Hapalochlaena fasciata	AB430529	Chiba, Japan
	Hapalochlaena lunulata	AB430530	Okinawa, Miyagi Island, Okinawa, Japan
Octopus	*Octopus tankahkeei*	JX456264	China
	Octopus vulgaris	HQ846110	Wenzhou, Zhejiang province, China
	Octopus vulgaris	HQ846154	Xiamen, Fujian province, China
	Octopus vulgaris	JX456270	China
	Octopus vulgaris	AB430547	Kanagawa, Misaki, Japan
	Octopus vulgaris	AB430546	Hyougo, Japan

			Octopus oshimai	JX456268	China
			Octopus conispadiceus	AB430533	Hokkaido, Kushiro, Hokkaido, Japan
			Octopus cyanea	AB430535	Okinawa, Nakagusuku, Okinawa, Japan
			Octopus cyanea	AB430534	Tokyo, Ogasawara Island, Japan
			Octopus incella	AB430542	Okinawa, Motobu, Hamamoto, Okinawa, Japan
			Octopus laqueus	AB430543	Okinawa, Kadena, Mizugama, Okinawa, Japan
			Octopus longispadiceus	AB430537	Hyogo, Kasumi, Shirosaki, Japan
			Octopus nanhaiensis	HQ846121	Sanya, Hainan province, China
			Octopus oliveri	AB430532	Tokyo, Ogasawara Island, Japan
			Octopus parvus	AB430544	Kanagawa, Misaki, Japan
			Octopus wolfi	AB430545	Okinawa, Kadena, Mizugama, Okinawa, Japan
Oegopsida	Architeuthidae	*Architeuthis*	*Architeuthis dux*	KC701762	Sea of Japan, Japan
			Architeuthis dux	KC701757	Off Chichijima,Ogasawara, Japan
			Architeuthis dux	KC701751	Sea of Japan, Japan
			Architeuthis dux	KC701741	Ohta City, Shimane Pref., Sea of Japan, Japan
			Architeuthis dux	KC701731	Sea of Japan, Japan
			Architeuthis dux	KC701730	Ogasawara, Japan
	Gonatidae	*Berryteuthis*	*Berryteuthis anonychus*	AB749277	Offshore Kushiro,western North Pacific, Japan
			Berryteuthis magister	AB749281	Offshore Kushiro,western North Pacific, Japan
		Gonatopsis	*Gonatopsis borealis*	AB749275	Offshore Kushiro,western North Pacific, Japan
			Gonatopsis octopedatus	AB749282	Offshore Aomori Pref. ,Sea of Japan
		Gonatus	*Gonatus berryi*	AB749280	Offshore Kushiro,western North Pacific, Japan
			Gonatus kamtschaticus	AB749274	Offshore Kushiro,western North Pacific, Japan
			Gonatus madokai	AB749284	Shore of Rausu, Hokkaido, Hokkaido, Japan
			Gonatus madokai	AB749283	Shore of Rausu, Hokkaido, Hokkaido, Japan
			Gonatus madokai	AB749278	Offshore Kushiro,western North Pacific, Japan
			Gonatus onyx	AB749279	Offshore Kushiro,western North Pacific, Japan
			Gonatus pyros	AB749273	Offshore Kushiro,western North Pacific, Japan
Sepiida	Sepiidae	*Sepia*	*Sepia aculeata*	HQ846106	Yangjiang, Guangdong province, China
			Sepia aculeata	HQ846107	Zhanjiang, Guangdong province, China
			Sepia aculeata	HQ846108	Qinzhou, Guangxi province, China

Sepia aculeata	HQ846083	Putian, Fujian province, China
Sepia andreana	AB430401	Osaka, Japan
Sepia aureomaculata	AB430402	Shizuoka, Japan
Sepia esculenta	HQ846084	Yangjiang, Guangdong province, China
Sepia esculenta	HQ846085	Zhoushan, Zhejiang province, China
Sepia esculenta	HQ846086	Zhoushan, Zhejiang province, China
Sepia esculenta	HQ846089	Qingdao, Shandong province, China
Sepia esculenta	HQ846087	Rizhao, Shandong province, China
Sepia esculenta	HQ846088	Dandong, Liaoning province, China
Sepia esculenta	HQ846091	Qinzhou, Guangxi province, China
Sepia esculenta	HQ846090	Beibhai, Guangxi province, China
Sepia esculenta	AB192335	Wakayama, Japan
Sepia kobiensis	AB193813	Kochi, Irino, Japan
Sepia latimanus	AB192338	Okinawa, Makishi, Okinawa, Japan
Sepia lorigera	AB193810	Mie, Japan
Sepia lycidas	HQ846109	Yangjiang, Guangdong province, China
Sepia lycidas	AB192337	Wakayama, Japan
Sepia lycidas	AB675088	Miyazaki, Japan
Sepia madokai	AB430407	Tottori, Karo, Japan
Sepia pardex	AB193809	Tottori, Sakaiminato, Japan
Sepia peterseni	AB192339	Kouchi, Japan
Sepia pharaonis	HQ846093	Yangjiang, Guangdong province, China
Sepia pharaonis	JN315869	Lingshui, Hainan province, China
Sepia pharaonis	JN315870	Lingshui, Hainan province, China
Sepia pharaonis	JN315871	Lingshui, Hainan province, China
Sepia pharaonis	JN315872	Lingshui, Hainan province, China
Sepia pharaonis	JN315873	Lingshui, Hainan province, China
Sepia pharaonis	JN315874	Lingshui, Hainan province, China
Sepia recurvirostra	HQ846092	Lingao, Hainan province, China
Sepia recurvirostra	HQ846161	Lingao, Hainan province, China
Sepia recurvirostra	HQ846162	Lingao, Hainan province, China

			Sepia subtenuipes	AB430414	Shizuoka, Japan
			Sepia tenuipes	AB430411	Shizuoka, Japan
			Sepia tokioensis	AB430412	Tottori, Karo, Japan
		Sepiella	*Sepiella maindroni*	AB192341	Osaka-bay, Japan
			Sepiella maindroni	AF346853	Tachibana Bay, Japan
			Sepiella inermis	HQ846080	Yangjiang, Guangdong province, China
			Sepiella inermis	HQ846081	Yangjiang, Guangdong province, China
			Sepiella japonica	HQ846078	Yangjiang, Guangdong province, China
			Sepiella japonica	HQ846079	Yangjiang, Guangdong province, China
			Sepiella japonica	HQ846082	Putian, Fujian province, China
Sepiolida	Sepiolidae	*Euprymna*	*Euprymna berryi*	HQ846099	Beibhai, Guangxi province, China
			Euprymna berryi	HQ846100	Beibhai, Guangxi province, China
			Euprymna berryi	HQ846101	Yangjiang, Guangdong province, China
			Euprymna berryi	HQ846102	Yangjiang, Guangdong province, China
			Euprymna morsei	HQ846103	Qingdao, Shandong province, China
			Euprymna morsei	HQ846104	Rizhao, Shandong province, China
			Euprymna morsei	HQ846105	Rizhao, Shandong province, China
		Sepiola	*Sepiola birostrata*	HQ846094	Rizhao, Shandong province, China
			Sepiola birostrata	HQ846095	Rizhao, Shandong province, China
			Sepiola birostrata	HQ846096	Rizhao, Shandong province, China
			Sepiola birostrata	HQ846097	Rizhao, Shandong province, China
			Sepiola birostrata	HQ846098	Rizhao, Shandong province, China
		Sepiolina	*Sepiolina nipponensis*	AB591073	Tosa Bay, Japan
			Sepiolina petasa	AB591071	Okinawa, off Kumeshima Island, Okinawa, Japan
Teuthida	Loliginidae	*Loliolus*	*Loliolus beka*	HQ529502	Rizhao, Shandong province, China
			Loliolus beka	HQ529503	Rizhao, Shandong province, China
			Loliolus beka	HQ529504	Rizhao, Shandong province, China
			Loliolus beka	HQ529505	Qingdao, Shandong province, China
			Loliolus beka	HQ529506	Qingdao, Shandong province, China
			Loliolus beka	HQ529507	Qingdao, Shandong province, China
			Loliolus beka	HQ529508	Qingdao, Shandong province, China

	Loliolus beka	HQ529509	Qingdao, Shandong province, China
	Loliolus beka	HQ529510	Rizhao, Shandong province, China
	Loliolus beka	HQ529511	Rizhao, Shandong province, China
	Loliolus beka	HQ529512	Rizhao, Shandong province, China
	Loliolus beka	HQ529513	Rizhao, Shandong province, China
	Loliolus beka	HQ529514	Rizhao, Shandong province, China
	Loliolus beka	HQ529515	Yangjiang, Guangdong province, China
	Loliolus beka	HQ529516	Yangjiang, Guangdong province, China
	Loliolus japonica	HQ529517	Xiamen, Fujian province, China
	Loliolus japonica	HQ529518	Xiamen, Fujian province, China
	Loliolus japonica	HQ529519	Xiamen, Fujian province, China
	Loliolus japonica	HQ529520	Xiamen, Fujian province, China
	Loliolus japonica	HQ529521	Qinzhou, Guangxi province, China
	Loliolus japonica	HQ529522	Qinzhou, Guangxi province, China
	Loliolus uyii	HQ529524	Yangjiang, Guangdong province, China
	Loliolus uyii	HQ529523	Sanya, Hainan province, China
	Loliolus uyii	HQ529525	Sanya, Hainan province, China
	Loliolus uyii	HQ529526	Sanya, Hainan province, China
	Loliolus uyii	HQ529527	Sanya, Hainan province, China
Uroteuthis	*Uroteuthis sibogae*	HQ529537	Xiamen, Fujian province, China
	Uroteuthis sibogae	HQ529538	Xiamen, Fujian province, China
	Uroteuthis sibogae	HQ529539	Xiamen, Fujian province, China
	Uroteuthis chinensis	HQ529528	Sanya, Hainan province, China
	Uroteuthis chinensis	EU349446	Fujian, Xiamen province, China
	Uroteuthis chinensis	EU349445	Fujian, Xiamen province, China
	Uroteuthis chinensis	EU349444	Fujian, Xiamen province, China
	Uroteuthis chinensis	EU349443	Fujian, Xiamen province, China
	Uroteuthis chinensis	EU349438	Hong Kong, China
	Uroteuthis chinensis	EU349437	Hong Kong, China
	Uroteuthis duvaucelii	HQ529529	Qinzhou, Guangxi province, China
	Uroteuthis duvaucelii	HQ529530	Qinzhou, Guangxi province, China

				Uroteuthis duvaucelii	HQ529531	Sanya, Hainan province, China
				Uroteuthis duvaucelii	HQ529532	Sanya, Hainan province, China
				Uroteuthis duvaucelii	HQ529533	Pingtan, Fujian province, China
				Uroteuthis duvaucelii	HQ529534	Pingtan, Fujian province, China
				Uroteuthis duvaucelii	HQ529535	Beibhai, Guangxi province, China
				Uroteuthis duvaucelii	EU349465	Shanghai, China
				Uroteuthis duvaucelii	EU349464	Hong Kong, China
				Uroteuthis duvaucelii	EU349463	Hong Kong, China
				Uroteuthis edulis	EU349462	Shanghai, China
				Uroteuthis edulis	EU349461	Shanghai, China
				Uroteuthis edulis	EU349460	Shanghai, China
				Uroteuthis edulis	EU349459	Shanghai, China
				Uroteuthis edulis	EU349458	Shanghai, China
				Uroteuthis edulis	AB675080	Yamaguchi, Japan
				Uroteuthis edulis	AB675081	Shimane, Japan
				Uroteuthis edulis	KF032040	Niigata, Japan
				Uroteuthis edulis	KF032039	Saga, Japan
				Uroteuthis edulis	KF032038	Saga, Japan
				Uroteuthis edulis	KF032037	Saga, Japan
				Uroteuthis edulis	EU349456	Japan
				Uroteuthis edulis	EU349455	Japan
				Uroteuthis edulis	EU349454	Japan
				Uroteuthis edulis	EU349453	Japan
				Uroteuthis edulis	EU349452	Japan
				Uroteuthis edulis	EU349451	Japan
				Uroteuthis edulis	EU349450	Japan
				Uroteuthis edulis	EU349449	Japan
				Uroteuthis edulis	EU349448	Japan
				Uroteuthis edulis	EU349447	Japan
Gastropoda	Anaspidea	Aplysiidae	*Aplysia*	*Aplysia kurodai*	JX560148	Japan
				Aplysia kurodai	JX560147	Japan

Archaeogastropoda	Calliostomatidae	*Calliostoma*	*Calliostoma aculeatum*	AB505271	Kanagawa, Miura, Off Zyogasima, Japan
			Calliostoma akoya	AB505272	Chiba, Japan
			Calliostoma consors	FN435323	Mie, Sugashima, Japan
			Calliostoma haliarchus	AB505273	Iwate, Off Yagi, Japan
			Calliostoma sakashitai	AB365225	Kanagawa, Misaki, Japan
			Calliostoma shinagawaensis	AB505275	Kanagawa, Miura, Off Zyogasima, Japan
	Lepetidae	*Lepeta*	*Lepeta caeca*	AB238458	Hokkaido, Akkeshi, Hokkaido, Japan
			Lepeta caeca	AB543978	Hokkaido, Akkeshi, Hokkaido, Japan
		Limalepeta	*Limalepeta lima*	AB543979	Hokkaido, Urakawa-cho, Hokkaido, Japan
			Limalepeta lima	AB543980	Hokkaido, Urakawa-cho, Hokkaido, Japan
		Sagamilepeta	*Sagamilepeta sagamiensis*	AB543981	Chiba, Japan
			Sagamilepeta sagamiensis	AB543982	Chiba, Japan
	Lepetodrilidae	*Lepetodrilus*	*Lepetodrilus nux*	AB820805	The Okinawa Trough, Okinawa, Japan
			Lepetodrilus nux	AB820806	The Okinawa Trough, Okinawa, Japan
			Lepetodrilus nux	AB820807	The Okinawa Trough, Okinawa, Japan
			Lepetodrilus nux	AB820808	The Okinawa Trough, Okinawa, Japan
			Lepetodrilus nux	AB820809	The Okinawa Trough, Okinawa, Japan
			Lepetodrilus nux	AB820810	The Okinawa Trough, Okinawa, Japan
			Lepetodrilus nux	AB820811	The Okinawa Trough, Okinawa, Japan
			Lepetodrilus nux	AB820812	The Okinawa Trough, Okinawa, Japan
			Lepetodrilus nux	AB820813	The Okinawa Trough, Okinawa, Japan
			Lepetodrilus nux	AB820814	The Okinawa Trough, Okinawa, Japan
			Lepetodrilus nux	AB820815	The Okinawa Trough, Okinawa, Japan
			Lepetodrilus nux	AB820816	The Okinawa Trough, Okinawa, Japan
			Lepetodrilus nux	AB820817	The Okinawa Trough, Okinawa, Japan
			Lepetodrilus nux	AB820818	The Okinawa Trough, Okinawa, Japan
			Lepetodrilus nux	AB820819	The Okinawa Trough, Okinawa, Japan
			Lepetodrilus nux	AB820820	The Okinawa Trough, Okinawa, Japan
			Lepetodrilus nux	AB820821	The Okinawa Trough, Okinawa, Japan
			Lepetodrilus nux	AB820822	The Okinawa Trough, Okinawa, Japan

		Lepetodrilus nux	AB820823	The Okinawa Trough, Okinawa, Japan
		Lepetodrilus nux	AB820824	The Okinawa Trough, Okinawa, Japan
		Lepetodrilus nux	AB820825	The Okinawa Trough, Okinawa, Japan
		Lepetodrilus nux	AB820826	The Okinawa Trough, Okinawa, Japan
		Lepetodrilus nux	AB820827	The Okinawa Trough, Okinawa, Japan
		Lepetodrilus nux	AB820828	The Okinawa Trough, Okinawa, Japan
		Lepetodrilus nux	AB820829	The Okinawa Trough, Okinawa, Japan
		Lepetodrilus nux	AB820830	The Okinawa Trough, Okinawa, Japan
		Lepetodrilus nux	AB820831	The Okinawa Trough, Okinawa, Japan
		Lepetodrilus nux	AB820832	The Okinawa Trough, Okinawa, Japan
		Lepetodrilus nux	AB820833	The Okinawa Trough, Okinawa, Japan
		Lepetodrilus nux	AB820834	The Okinawa Trough, Okinawa, Japan
		Lepetodrilus nux	AB820835	The Okinawa Trough, Okinawa, Japan
		Lepetodrilus nux	AB820836	The Okinawa Trough, Okinawa, Japan
		Lepetodrilus nux	AB820837	The Okinawa Trough, Okinawa, Japan
		Lepetodrilus nux	AB820838	The Okinawa Trough, Okinawa, Japan
		Lepetodrilus nux	AB820839	The Okinawa Trough, Okinawa, Japan
Pleurotomariidae	*Mikadotrochus*	*Mikadotrochus beyrichii*	AM049331	Boso Peninsula, Chiba Prefecture, Japan
		Mikadotrochus beyrichii	EU530109	Japan
Skeneidae	*Dillwynella*	*Dillwynella vitrea*	AM049336	Owase City, Mie Prefecture, Japan
		Dillwynella vitrea	EU530143	Japan
	Munditiella	*Munditiella ammonoceras*	AM049337	Okinawa, Aguni Island, Okinawa, Japan
		Munditiella ammonoceras	AB365244	Okinawa, Ishigaki, Okinawa, Japan
Stomatellidae	*Stomatella*	*Stomatella impertusa*	AB505292	Okinawa, Benoki, Okinawa, Japan
		Stomatella planulata	AB505293	Kagoshima, Japan
		Stomatella planulata	EU530132	Japan
		Stomatella planulata	EU530131	Japan
		Stomatella planulata	EU530130	Japan
Trochidae	*Alcyna*	*Alcyna ocellata*	AB505278	Kumamoto, Nogamazima, Japan
		Alcyna ocellata	AB505277	Kumamoto, Nogamazima, Japan
	Broderipia	*Broderipia iridescens*	AB505284	Kagoshima, Japan

	Broderipia iridescens	EU530139	Japan
Cantharidus	*Cantharidus bisbalteatus*	AB505279	Kumamoto, Nogamazima, Japan
	Cantharidus callichroa	AM049338	Omaezaki Town, Shizuoka Prefecture, Japan
	Cantharidus callichroa	EU530120	Japan
	Cantharidus callichroa	EU530119	Japan
	Cantharidus infuscatus	AB505282	Kagoshima, Japan
	Cantharidus jessoensis	AB505280	Iwate, Otsuchi, Japan
Chlorostoma	*Chlorostoma lischkei*	EU530145	Japan
	Chlorostoma lischkei	EU530144	Japan
	Chlorostoma turbinatum	HM180511	Korea
	Chlorostoma turbinatum	HM180512	Korea
	Chlorostoma turbinatum	HM180513	Korea
	Chlorostoma turbinatum	HM180514	Korea
	Chlorostoma turbinatum	HM180515	Korea
	Chlorostoma turbinatum	HM180516	Korea
	Chlorostoma turbinatum	HM180517	Korea
	Chlorostoma turbinatum	HM180518	Korea
	Chlorostoma turbinatum	HM180519	Korea
	Chlorostoma turbinatum	HM180520	Korea
	Chlorostoma turbinatum	HM180521	Korea
	Chlorostoma turbinatum	HM180522	Korea
	Chlorostoma turbinatum	HM180523	Korea
	Chlorostoma turbinatum	HM180524	Korea
	Chlorostoma turbinatum	HM180525	Korea
	Chlorostoma turbinatum	HM180526	Korea
Clanculus	*Clanculus bronni*	AB505296	Kumamoto, Amakusa, Tsuzizima, Japan
	Clanculus margaritarius	AB505297	Kagoshima, Japan
	Clanculus microdon	AB505298	Kumamoto, Nogamazima, Japan
Conotalopia	*Conotalopia mustelina*	AB505302	Kanagawa, Manaduru, Japan
	Conotalopia ornata	AB505303	Chiba, Japan
Diloma	*Diloma piperinus*	AB505288	Okinawa, Benoki, Okinawa, Japan

	Diloma radula	AY858090	Okinawa, Okinawa, Japan
	Diloma suavis	AB505281	Tokyo, Ogasawara Islands, Chichijima Suzaki, Japan
Ethaliella	*Ethaliella floccata*	AB505304	Okinawa, Kyoda, Okinawa, Japan
	Ethaliella floccata	AB505305	Okinawa, Kyoda, Okinawa, Japan
	Ethaliella floccata	EU530137	Japan
Ethminolia	*Ethminolia stearnsii*	AB505306	Chiba, Japan
	Ethminolia stearnsii	AB505307	Chiba, Japan
Eurytrochus	*Eurytrochus cognatus*	AB505299	Kumamoto, Amakusa, Tsuzizima, Japan
	Eurytrochus cognatus	EU530133	Japan
Hazuregyra	*Hazuregyra watanabei*	HF586245	Off Kinkazan, Miyagi, Honshu, Honshu, Japan
Lirularia	*Lirularia iridescens*	EU530125	Japan
	Lirularia iridescens	EU530124	Japan
	Lirularia pygmaea	AB505308	Chiba, Japan
	Lirularia pygmaea	AB505309	Chiba, Japan
Monilea	*Monilea smithi*	AB505310	Oita, Japan
	Monilea smithi	AB505311	Oita, Japan
Monodonta	*Monodonta perplexa*	DQ061096	Hokkaido, Hokkaido, Japan
	Monodonta canalifera	DQ061095	Okinawa, Okinawa, Japan
	Monodonta canalifera	EU530128	Japan
	Monodonta australis	HM180693	Korea
	Monodonta australis	HM180694	Korea
	Monodonta australis	HM180695	Korea
	Monodonta australis	HM180696	Korea
	Monodonta australis	HM180697	Korea
	Monodonta australis	HM180698	Korea
	Monodonta australis	HM180699	Korea
Omphalius	*Omphalius pfeifferi*	HM180730	Korea
	Omphalius pfeifferi	HM180731	Korea
	Omphalius rusticus rusticus	HM180733	Korea
	Omphalius rusticus	HM180734	Korea

			rusticus		
			Omphalius rusticus	HM180735	Korea
		Pseudostomatella	*Pseudostomatella decolorata*	AB505290	Okinawa, Benoki, Okinawa, Japan
			Pseudostomatella decolorata	AB505291	Okinawa, Benoki, Okinawa, Japan
		Pteria	*Pteria brevialata*	KU341962	Weizhou Island, Guangxi province, China
			Pteria penguin	KU341959	Weizhou Island, Guangxi province, China
			Pteria penguin	KU341960	Weizhou Island, Guangxi province, China
			Pteria penguin	KU341961	Weizhou Island, Guangxi province, China
		Rossiteria	*Rossiteria nuclea*	AB505312	Kagoshima, Japan
		Solariella	*Solariella nyssonus*	HF586294	E of Daiozaki, Mie, Honshu I., Honshu, Japan
			Solariella nyssonus	HF586295	Off Kanaya, Chiba, Honshu I., Honshu, Japan
			Stomatia obscura	AB505294	Kagoshima, Japan
			Stomatia phymotis	AB365223	Okinawa, Ishigaki, Okinawa, Japan
		Strombus	*Strombus vittatus*	JF693433	Weizhou Island, Guangxi province, China
			Strombus vittatus	JF693434	Beibhai, Guangxi province, China
			Strombus vittatus	JF693435	Beibhai, Guangxi province, China
		Trochus	*Trochus histrio*	AB505300	Kagoshima, Japan
			Trochus maculatus	EU530134	Japan
			Trochus maculatus	AB365224	Okinawa, Iriomote, Okinawa, Japan
			Trochus maculatus	AB505301	Kagoshima, Japan
			Trochus stellatus	EU530135	Japan
		Umbonium	*Umbonium giganteum*	AB505314	Shizuoka, Japan
			Umbonium giganteum	AB505313	Shizuoka, Japan
			Umbonium moniliferum	AB505315	Kumamoto,Yokakuwan, Japan
			Umbonium moniliferum	AB365227	Miyazaki, Japan
Cephalaspidea	Haminoeidae	*Haminoea*	*Haminoea japonica*	JN830658	Japan
			Haminoea japonica	JN830657	Japan
			Haminoea japonica	JN830656	Japan
			Haminoea japonica	JN830655	Japan

Haminoea japonica	JN830654	Japan
Haminoea japonica	JN830653	Japan
Haminoea japonica	JN830652	Japan
Haminoea japonica	JN830651	Japan
Haminoea japonica	JN830650	Japan
Haminoea japonica	JN830649	Japan
Haminoea japonica	JN830648	Japan
Haminoea japonica	KF572952	Japan
Haminoea japonica	KF572953	Japan
Haminoea japonica	KF572954	Japan
Haminoea japonica	KF572958	Japan
Haminoea japonica	KF572959	Japan
Haminoea japonica	KF572960	Japan
Haminoea japonica	KF572961	Japan
Haminoea japonica	KF572962	Japan
Haminoea japonica	KF572963	Japan
Haminoea japonica	KF572964	Japan
Haminoea japonica	KF572965	Japan
Haminoea japonica	KF572966	Japan
Haminoea japonica	KF572967	Japan
Haminoea japonica	KF572968	Japan
Haminoea japonica	KF572969	Japan
Haminoea japonica	KF572970	Japan
Haminoea japonica	KF572971	Japan
Haminoea japonica	KF572972	Japan
Haminoea japonica	KF572973	Japan
Haminoea japonica	KF572974	Japan
Haminoea japonica	KF572975	Japan
Haminoea japonica	KF572976	Japan
Haminoea japonica	KF572977	Japan
Haminoea japonica	KF572978	Japan

		Haminoea japonica	KF572979	Japan
		Haminoea japonica	KF572980	Japan
		Haminoea japonica	KF572981	Japan
		Haminoea japonica	KF572982	Japan
		Haminoea japonica	KF572983	Japan
		Haminoea japonica	KF572984	Japan
		Haminoea japonica	KF572985	Japan
		Haminoea japonica	KF572986	Japan
		Haminoea japonica	KF572987	Japan
		Haminoea japonica	KF572988	Japan
Aglajidae	*Melanochlamys*	*Melanochlamys ezoensis*	KJ704899	Japan
		Melanochlamys ezoensis	KJ704900	Japan
		Melanochlamys ezoensis	KJ704901	Japan
		Melanochlamys ezoensis	KJ704902	Japan
		Melanochlamys ezoensis	KJ704903	Japan
		Melanochlamys ezoensis	KJ704904	Japan
		Melanochlamys ezoensis	KJ704905	Japan
		Melanochlamys fukudai	KJ704906	Japan
		Melanochlamys fukudai	KJ704907	Japan
		Melanochlamys fukudai	KJ704908	Japan
		Melanochlamys fukudai	KJ704909	Japan
		Melanochlamys fukudai	KJ704910	Japan
		Melanochlamys fukudai	KJ704911	Japan
		Melanochlamys fukudai	KJ704912	Japan
		Melanochlamys fukudai	KJ704913	Japan
		Melanochlamys fukudai	KJ704914	Japan
		Melanochlamys fukudai	KJ704915	Japan
		Melanochlamys fukudai	KJ704916	Japan
		Melanochlamys fukudai	KJ704917	Japan
		Melanochlamys fukudai	KJ704918	Japan
		Melanochlamys fukudai	KJ704919	Japan

			Melanochlamys fukudai	KJ704920	Japan
			Melanochlamys fukudai	KJ704921	Japan
			Melanochlamys fukudai	KJ704922	Japan
			Melanochlamys fukudai	KJ704923	Japan
			Melanochlamys fukudai	KJ704924	Japan
			Melanochlamys fukudai	KJ704925	Japan
			Melanochlamys fukudai	KJ704926	Japan
			Melanochlamys fukudai	KJ704927	Japan
			Melanochlamys fukudai	KJ704928	Japan
			Melanochlamys fukudai	KJ704929	Japan
			Melanochlamys fukudai	KJ704930	Japan
			Melanochlamys kohi	KJ704933	Japan
			Melanochlamys kohi	KJ704934	Japan
			Melanochlamys kohi	KJ704935	Japan
Cocculiniformia	Cocculinidae	*Coccopigya*	*Coccopigya punctoradiata*	AB238590	Kochi, Japan
			Coccopigya punctoradiata	AB365259	Miyazaki, Japan
Cycloneritimorpha	Neritidae	*Nerita*	*Nerita albicilla*	AM049327	Okinawa Island, Okinawa Prefecture, Okinawa, Japan
			Nerita helicinoides	EU732252	South of Cape Hedo,Okinawa, Okinawa, Japan
			Nerita helicinoides	EU732251	Cape Zanpa, Okinawa, Okinawa, Japan
			Nerita japonica	EU732260	Misaki, Miura Peninsula, Japan
			Nerita japonica	EU732259	Misaki, Miura Peninsula, Japan
			Nerita ocellata	EU732280	South of Oku,Okinawa, Okinawa, Japan
			Nerita ocellata	EU732279	South of Oku,Okinawa, Okinawa, Japan
			Nerita planospira	EU732291	Yagaji Jima, Okinawa, Okinawa, Japan
			Nerita plicata	EU732293	South of Oku,Okinawa, Okinawa, Japan
			Nerita tristis	EU732324	South of Oku,Okinawa, Okinawa, Japan
			Nerita tristis	EU732323	South of Oku,Okinawa, Okinawa, Japan
			Nerita undata	EU732333	Hong Kong, China
			Nerita undata	EU732334	Hong Kong, China
			Nerita undata	EU732336	South of Oku,Okinawa, Okinawa, Japan
			Nerita undata	EU732335	South of Oku,Okinawa, Okinawa, Japan

			Nerita undulata	EU732352	Yagaji Jima, Okinawa, Okinawa, Japan
			Nerita undulata	EU732351	Yagaji Jima, Okinawa, Okinawa, Japan
			Nerita yoldii	EU732359	Hong Kong, China
			Nerita yoldii	EU732360	Hong Kong, China
		Neritina	*Neritina asperulata*	AB477474	Okinawa, Nago, Ooura, Okinawa, Japan
			Neritina asperulata	AB477473	Okinawa, Iriomote Takana, Okinawa, Japan
			Neritina asperulata	AB477472	Okinawa, Ishigaki Hoshino, Okinawa, Japan
			Neritina iris	AB477491	Okinawa, Iriomote Takana, Okinawa, Japan
			Neritina iris	AB477490	Kagoshima, Japan
			Neritina petitii	AB477497	Okinawa, Iriomote Takana, Okinawa, Japan
			Neritina petitii	AB477496	Okinawa, Ishigaki Hoshino, Okinawa, Japan
			Neritina petitii	AB477495	Okinawa, Ishigaki Hoshino, Okinawa, Japan
			Neritina pulligera	AB477502	Okinawa, Ishigaki Hoshino, Okinawa, Japan
			Neritina pulligera	AB477501	Kagoshima, Japan
Docoglossa	Patellidae	*Scutellastra*	*Scutellastra flexuosa*	AB238583	Wakayama, Japan
			Scutellastra optima	AB238585	Kagoshima, Japan
Gastropoda_order_incertae_sedis	Turbinidae	*Angaria*	*Angaria formosa*	AM049342	Sunabe, Okinawa Prefecture, Okinawa, Japan
			Angaria formosa	AM049343	Hinomisaki,Wakayama Prefecture, Japan
		Collonista	*Collonista amakusaensis*	AM049345	Minatogawa, Okinawa Prefecture, Okinawa, Japan
			Collonista costulosa	AM049346	Seragaki,Okinawa Prefecture, Okinawa, Japan
		Gabrielona	*Gabrielona pisinna*	AM049356	Okinawa, Aguni Island, Okinawa, Japan
			Gabrielona pisinna	AM049357	Okinawa, Aguni Island, Okinawa, Japan
		Guildfordia	*Guildfordia triumphans*	AM049375	Sakai, Wakayama Prefecture, Japan
			Guildfordia triumphans	AM049376	|Straits of Koshiki, Kagoshima Pref.Japan
			Guildfordia yoka	AM049377	Okinawa, I., Okinawa Prefecture, Okinawa, Japan
			Guildfordia yoka	AM049378	Okinawa, I., Okinawa Prefecture, Okinawa, Japan
			Guildfordia yoka	EU530156	Japan
		Lunella	*Lunella cinerea*	AB588872	Okinawa, Iriomote Island, Okinawa, Japan
			Lunella cinerea	AB297735	Okinawa, Iriomote Island, Okinawa, Japan
			Lunella cinerea	AB297734	Okinawa, Iriomote Island, Okinawa, Japan
			Lunella cinerea	AB297733	Okinawa, Iriomote Island, Okinawa, Japan

	Lunella coreensis	HQ681192	Japan
	Lunella coreensis	HQ681191	Japan
	Lunella coreensis	AM403861	Nakanohama,Hazu-cho, Aichi Pref., Japan
	Lunella coreensis	AM403860	Funagawaminato-kohama, Oga, Akita Pref, Japan
	Lunella coreensis	AB297732	Aich, Morozaki, Japan
	Lunella coreensis	AB297731	Kagawa, Japan
	Lunella coreensis	AB297730	Mie, Japan
	Lunella coreensis	HM180657	Korea
	Lunella coreensis	HM180658	Korea
	Lunella coreensis	HM180659	Korea
	Lunella coronata	AB297729	Okinawa, Yonashiro, Yakena, Japan
	Lunella coronata	AB297728	Okinawa, Yonashiro, Yakena, Japan
	Lunella coronata	AB297727	Okinawa, Yonashiro, Yakena, Japan
	Lunella granulata	AM403863	Hong Kong, China
	Lunella granulata	AB588892	Kagoshima, Japan
	Lunella granulata	AB588891	Japan
	Lunella granulata	AM403862	Teruma Beach Yonashiro, Okinawa Pref, Okinawa, Japan
	Lunella moniliformis	AB588878	Kagoshima, Japan
	Lunella moniliformis	AB588877	Kagoshima, Japan
	Lunella moniliformis	AB588876	Kagoshima, Japan
	Lunella ogasawarana	AB588883	Bonin Is, Chichizima, Japan
	Lunella ogasawarana	AB588882	Bonin Is, Chichizima, Japan
	Lunella ogasawarana	AB588881	Bonin Is, Chichizima, Japan
	Lunella ogasawarana	AB588880	Bonin Is, Chichizima, Japan
	Lunella ogasawarana	AB588879	Bonin Is, Chichizima, Japan
	Lunella ogasawarana	AB297723	Tokyo, Ogasawara Islands, Chichi-jima, Japan
	Lunella ogasawarana	AB297725	Tokyo, Ogasawara Islands, Chichi-jima, Japan
	Lunella ogasawarana	AB297724	Tokyo, Ogasawara Islands, Chichi-jima, Japan
	Lunella ogasawarana	AB297726	Tokyo, Ogasawara Islands, Chichi-jima, Japan
	Lunella ogasawarana	AB297722	Tokyo, Ogasawara Islands, Chichi-jima, Japan
Phasianella	*Phasianella solida*	AM049353	Chikura, Chiba Prefecture, Japan

			Phasianella solida	AM049354	Tsubaki Onsen, Wakayama Prefecture, Japan
		Pomaulax	*Pomaulax japonicus*	AM049380	KatsuuraChiba, Prefecture, Japan
			Pomaulax japonicus	AB297737	Chiba, Japan
		Turbo	*Turbo argyrostomus*	AM403899	Itoman,Okinawa Pref., Okinawa, Japan
			Turbo chrysostomus	AM403903	Iriomote I., Taketomi, Okinawa Pref. Japan
			Turbo cornutus	AM403882	Kii Nagashima, Mie Pref., Japan
			Turbo cornutus	AM403881	Morozaki, Minamichita-cho, Aichi Pref., Japan
			Turbo cornutus	HM180932	Korea
			Turbo cornutus	HM180933	Korea
			Turbo cornutus	HM180934	Korea
			Turbo marmoratus	AM403895	Chinen, Okinawa Pref., Okinawa, Japan
			Turbo marmoratus	AM403894	Kin, Okinawa Pref., Okinawa, Japan
			Turbo petholatus	AM049383	Seragaki,Okinawa Prefecture, Okinawa, Japan
			Turbo reevii	AM403878	Sakai, Minabe, Wakayama Pref., Japan
			Turbo setosus	AM403909	Higashizaki, Yonaguni I., Okinawa Pref., Japan
			Turbo stenogyrus	AM403916	Teruma Beach Yonashiro, Okinawa Pref, Okinawa, Japan
			Turbo stenogyrus	AM403915	HinomisakiHidaka, Wakayama, Pref, Japan
Littorinimorpha	Bursidae	*Bursa*	*Bursa granularis*	JF693344	Fuzhou, Fujian province, China
			Bursa granularis	JF693345	Rizhao, Shandong province, China
			Bursa granularis	JF693346	Weizhou Island, Guangxi province, China
			Bursa granularis	JF693347	Fangchenggang, Guangxi province, China
			Bursa granularis	JF693348	Fangchenggang, Guangxi province, China
			Bursa rana	JF693349	Zhanjiang, Guangdong province, China
			Bursa rana	JF693350	Beibhai, Guangxi province, China
			Bursa rana	JF693351	Beibhai, Guangxi province, China
			Bursa rana	JF693352	Pingtan, Fujian province, China
			Bursa rana	JF693353	Pingtan, Fujian province, China
	Cassidae	*Phalium*	*Phalium bisulcatum*	JF693409	Fuqing, Fujian province, China
			Phalium flammiferum	JF693410	Fuqing, Fujian province, China
	Cypraeidae	*Cypraea*	*Cypraea tigris*	JF693369	Paracel Islands, China
			Cypraea tigris	JF693370	Paracel Islands, China

		Cypraea vitellus	JF693371	Lingao, Hainan province, China
	Mauritia	*Mauritia arabica*	JF693392	Wenchang, Hainan province, China
		Mauritia arabica	JF693393	Lingao, Hainan province, China
		Mauritia arabica	JF693394	Lingao, Hainan province, China
		Mauritia arabica	JF693395	Lingao, Hainan province, China
	Erronea	*Erronea errones*	JF693396	Lingao, Hainan province, China
		Erronea errones	JF693397	Weizhou Island, Guangxi province, China
Ficidae	*Ficus*	*Ficus ficus*	JF693374	Zhanjiang, Guangdong province, China
		Ficus ficus	JF693375	Zhanjiang, Guangdong province, China
		Ficus ficus	JF693376	Zhanjiang, Guangdong province, China
		Ficus gracilis	JF693380	Sanya, Hainan province, China
		Ficus gracilis	JF693381	Sanya, Hainan province, China
		Ficus gracilis	JF693382	Beibu Bay, Guangxi province, China
		Ficus variegata	JF693377	Zhanjiang, Guangdong province, China
		Ficus variegata	JF693378	Zhanjiang, Guangdong province, China
		Ficus variegata	JF693379	Zhanjiang, Guangdong province, China
Littorinidae	*Echinolittorina*	*Echinolittorina cinerea*	AJ622991	Ogasawara Is, Chichijima I, Japan
		Echinolittorina radiata	JF693405	Shengshan, Zhejiang province, China
		Echinolittorina radiata	JF693406	Zhoushan, Zhejiang province, China
		Echinolittorina radiata	AJ623040	Tsubaki, Wakayama, Japan
		Echinolittorina reticulata	AJ623042	Ishigaki, Japan
		Echinolittorina reticulata	AM157057	Okinawa, Nago-shi, Okinawa, Japan
		Echinolittorina reticulata	AM157058	Okinawa, Nago-shi, Okinawa, Japan
		Echinolittorina reticulata	AM157059	Okinawa, Nago-shi, Okinawa, Japan
		Echinolittorina reticulata	AM157060	Okinawa, Nago-shi, Okinawa, Japan
		Echinolittorina reticulata	AM157061	Okinawa, Nago-shi, Okinawa, Japan
		Echinolittorina reticulata	AM157062	Okinawa, Nago-shi, Okinawa, Japan
		Echinolittorina reticulata	AM157063	Hachijo-jima, Mitsune, Japan
		Echinolittorina reticulata	AM157064	Ishigaki, Japan
		Echinolittorina reticulata	AM157065	Ishigaki, Japan
		Echinolittorina reticulata	AM157066	Ishigaki, Japan

Echinolittorina reticulata	AM157067	Ishigaki, Japan
Echinolittorina trochoides A	AM157086	Hong Kong, China
Echinolittorina trochoides E	AJ623057	Tsubaki, Wakayama, Japan
Echinolittorina trochoides E	AJ623058	Japan
Echinolittorina trochoides E	AM156817	Japan
Echinolittorina trochoides E	AM156818	Mitsuishi, Kanagawa, Japan
Echinolittorina trochoides E	AM156819	Mitsuishi, Kanagawa, Japan
Echinolittorina trochoides E	AM156820	Nago-shi,Okinawa, Okinawa, Japan
Echinolittorina trochoides E	AM156821	Nago-shi,Okinawa, Okinawa, Japan
Echinolittorina trochoides E	AM156822	Nago-shi,Okinawa, Okinawa, Japan
Echinolittorina trochoides E	AM156823	Nakazato, Kikaijima, Kagoshima, Japan
Echinolittorina trochoides E	AM156824	Nakazato, Kikaijima, Kagoshima, Japan
Echinolittorina trochoides E	AM156825	Nakazato, Kikaijima, Kagoshima, Japan
Echinolittorina trochoides E	AM156826	Shirahama, Wakayama, Japan
Echinolittorina trochoides E	AM156827	Shirahama, Wakayama, Japan
Echinolittorina trochoides E	AM156828	Mitsune, Hachijo-jima, Japan

	Echinolittorina trochoides E	AM156829	Mitsune, Hachijo-jima, Japan
	Echinolittorina trochoides E	AM156830	Mitsune, Hachijo-jima, Japan
	Echinolittorina trochoides E	AM156831	Tsuchi-hama, Amami-oshima, Japan
	Echinolittorina trochoides E	AM156832	Futami, Chichijima-hama, Amami-oshima, Japan
	Echinolittorina trochoides E	AM156833	Futami, Chichijima-hama, Amami-oshima, Japan
	Echinolittorina trochoides E	AM156834	Futami, Chichijima-hama, Amami-oshima, Japan
	Echinolittorina trochoides E	AM156835	Futami, Chichijima-hama, Amami-oshima, Japan
	Echinolittorina vidua	AM156986	Hong Kong, China
	Echinolittorina vidua	AM156985	Hong Kong, China
	Echinolittorina vidua	AM156984	Hong Kong, China
	Echinolittorina vidua	AM156983	Hong Kong, China
	Echinolittorina vidua	AM156982	Hong Kong, China
	Echinolittorina vidua	AM156981	Hong Kong, China
	Echinolittorina vidua	AM157002	Okinawa, Nago-shi, Okinawa, Japan
	Echinolittorina vidua	AM157003	Okinawa, Nago-shi, Okinawa, Japan
	Echinolittorina vidua	AM157004	Okinawa, Nago-shi, Okinawa, Japan
	Echinolittorina vidua	AM157005	Okinawa, Nago-shi, Okinawa, Japan
	Echinolittorina vidua	AM157006	Okinawa, Nago-shi, Okinawa, Japan
	Echinolittorina vidua	AM157007	Okinawa, Nago-shi, Okinawa, Japan
	Echinolittorina vidua	AM157008	Okinawa, Nago-shi, Okinawa, Japan
	Echinolittorina vidua	AM157009	Okinawa, Nago-shi, Okinawa, Japan
Littoraria	*Littoraria coccinea*	FN557093	Ishigaki I., Japan
	Littoraria intermedia	FN557104	Kyushu, Miyazaki, Kushima,Honjo R., Japan
	Littoraria intermedia	JF693389	Lingao, Hainan province, China

		Littoraria intermedia	JF693390	Fuqing, Fujian province, China
		Littoraria melanostoma	HE590830	Hong Kong, China
		Littoraria pallescens	FN557122	Makiya, Okinawa, Japan
		Littoraria pallescens	AB611831	Okinawa, Nago, Okinawa, Japan
		Littoraria pintado	AJ488634	Ishigaki Island, Japan
		Littoraria scabra	FN557135	Naha, Okinawa, Japan
		Littoraria scabra	JF693391	Rongcheng, Shandong province, China
		Littoraria sinensis	FN557140	Kyushu, Miyazaki, Kushima,Honjo R., Japan
	Littorina	*Littorina brevicula*	JF693387	Rizhao, Shandong province, China
		Littorina brevicula	JF693386	Fuqing, Fujian province, China
		Littorina brevicula	JF693388	Rongcheng, Shandong province, China
		Littorina brevicula	HE590833	Goura, , Japan
		Littorina horikawai	HE590836	Nejiko,Hirad I., Kyushu, Japan
		Littorina kasatka	HE590837	HokkaidoAkkeshi Marine Biological Station, Hokkaido, Japan
		Littorina mandshurica	HE590838	Abashiri, Hokkaido, Hokkaido, Japan
		Littorina squalida	HE590843	Abashiri, Hokkaido, Hokkaido, Japan
	Tectarius	*Tectarius spinulosus*	AJ488641	Ishigaki, Japan
		Tectarius spinulosus	AB611827	Kagoshima, Japan
Naticidae	*Natica*	*Natica lineata*	JF693401	Zhanjiang, Guangdong province, China
		Natica lineata	JF693402	Zhanjiang, Guangdong province, China
		Natica lineata	JF693404	Zhanjiang, Guangdong province, China
		Natica lineata	JF693403	Zhanjiang, Guangdong province, China
		Natica tigrina	JF693400	Beibhai, Guangxi province, China
Personidae	*Distorsio*	*Distorsio reticularis*	JF693372	Beibhai, Guangxi province, China
		Distorsio reticularis	JF693373	Beibhai, Guangxi province, China
Ranellidae	*Cymatium*	*Cymatium cingulatum*	JF693367	Sanya, Hainan province, China
		Cymatium cingulatum	JF693368	Sanya, Hainan province, China
Strombidae	*Lambis*	*Lambis lambis*	JF693383	Sanya, Hainan province, China
		Lambis lambis	JF693384	Sanya, Hainan province, China
		Lambis lambis	JF693385	Sanya, Hainan province, China

		Strombus	*Strombus lentiginosu*	JF693421	Lingshui, Hainan province, China
			Strombus lentiginosu	JF693422	Paracel Islands, China
			Strombus luhuanus	JF693429	Wenchang, Hainan province, China
			Strombus luhuanus	JF693430	Wenchang, Hainan province, China
			Strombus luhuanus	JF693431	Wenchang, Hainan province, China
			Strombus luhuanus	JF693432	Wenchang, Hainan province, China
			Strombus mutabiis	JF693420	Lingshui, Hainan province, China
			Strombus urceus	JF693423	Wenchang, Hainan province, China
			Strombus urceus	JF693424	Wenchang, Hainan province, China
			Strombus urceus	JF693425	Wenchang, Hainan province, China
		Margistrombus	*Margistrombus robustus*	JF693426	Beibhai, Guangxi province, China
			Margistrombus robustus	JF693427	Beibhai, Guangxi province, China
			Margistrombus robustus	JF693428	Beibhai, Guangxi province, China
			Margistrombus robustus	JF693436	Beibhai, Guangxi province, China
			Margistrombus robustus	JF693437	Beibhai, Guangxi province, China
	Tonnidae	*Tonna*	*Tonna dolium*	JF693441	Zhanjiang, Guangdong province, China
			Tonna dolium	JF693442	Zhanjiang, Guangdong province, China
			Tonna galea	JF693438	Haikou, Hainan province, China
			Tonna galea	JF693439	Haikou, Hainan province, China
			Tonna sulcosa	JF693440	Beibhai, Guangxi province, China
Neogastropoda	Buccinidae	*Babylonia*	*Babylonia areolata*	JN053011	Fuqing, Fujian province, China
			Babylonia areolata	HQ834066	Fuqing, Fujian province, China
			Babylonia areolata	JN053013	Fuqing, Fujian province, China
			Babylonia areolata	JN053012	Fuqing, Fujian province, China
			Babylonia lutosa	JN053010	Fuqing, Fujian province, China
		Buccinum	*Buccinum pemphigum*	JN052999	Dalian, Liaoning province, China
			Buccinum pemphigum	JN053000	Dalian, Liaoning province, China
			Buccinum pemphigum	JN053001	Dalian, Liaoning province, China
			Buccinum pemphigum	HQ834057	Dalian, Liaoning province, China
			Buccinum pemphigum	HQ834058	Dalian, Liaoning province, China
			Buccinum pemphigum	HQ834059	Dalian, Liaoning province, China

	Buccinum yokomaruae	JN052995	Dalian, Liaoning province, China
	Buccinum yokomaruae	JN052996	Dalian, Liaoning province, China
	Buccinum yokomaruae	JN052997	Dalian, Liaoning province, China
	Buccinum yokomaruae	JN052998	Dalian, Liaoning province, China
Cantharus	*Cantharus cecillei*	HQ834063	Fangchenggang, Guangxi province, China
	Cantharus cecillei	JN053007	Fangchenggang, Guangxi province, China
	Cantharus melanostomus	JN053038	Sanya, Hainan province, China
	Cantharus melanostomus	HQ834062	Sanya, Hainan province, China
Golikovia	*Golikovia ennae*	AB498779	Fukushima, Japan
	Golikovia fukueae	AB498780	Nagasaki,Hirazisone, Japan
	Neptunea arthritica	AB498778	Fukushima, Japan
	Neptunea arthritica	AB498777	Miyagi, Shitigahama, Japan
	Neptunea arthritica	AB498776	Miyagi, Shitigahama, Japan
Kelletia	*Kelletia lischkei*	HM180632	Korea
	Kelletia lischkei	HM180633	Korea
	Kelletia lischkei	HM180634	Korea
	Kelletia lischkei	HM180635	Korea
	Kelletia lischkei	HM180636	Korea
Neptunea	*Neptunea constricta*	AB498766	Fukushima, Japan
	Neptunea cumingi	HQ834061	Qingdao, Shandong province, China
	Neptunea cumingi	JN053006	Qingdao, Shandong province, China
	Neptunea cumingi	JN053005	Qingdao, Shandong province, China
	Neptunea cumingi	HM180715	Korea
	Neptunea cumingi	HM180716	Korea
	Neptunea frater	AB498769	Fukushima, Japan
	Neptunea frater	AB498768	Fukushima, Japan
	Neptunea frater	AB498767	Fukushima, Japan
	Neptunea intersculpta	AB498771	Fukushima, Japan
	Neptunea intersculpta	AB498770	Fukushima, Japan
	Neptunea kuroshio	AB498772	Shizuoka, Japan
	Neptunea mikawaensis	AB498773	Shizuoka, Japan

		Neptunea polycostata	AB498775	Hokkaido, Samani, Hokkaido, Japan
		Neptunea polycostata	AB498774	Hokkaido, Samani, Hokkaido, Japan
	Phos	*Phos senticosus*	JN053008	Beibhai, Guangxi province, China
		Phos senticosus	JN053009	Beibhai, Guangxi province, China
		Phos senticosus	HQ834064	Beibhai, Guangxi province, China
		Phos senticosus	HQ834065	Beibhai, Guangxi province, China
	Volutharpa	*Volutharpa ampullacea perryi*	JN053002	Rizhao, Shandong province, China
		Volutharpa ampullacea perryi	HQ834060	Rizhao, Shandong province, China
		Volutharpa ampullacea perryi	JN053004	Rizhao, Shandong province, China
		Volutharpa ampullacea perryi	JN053003	Rizhao, Shandong province, China
Clavatulidae	*Turricula*	*Turricula javana*	HQ834091	Lianyungang, Jiangsu province, China
Columbellidae	*Euplica*	*Euplica scripta*	HQ834054	Beibhai, Guangxi province, China
		Euplica scripta	JN052987	Beibhai, Guangxi province, China
		Euplica scripta	JN052986	Beibhai, Guangxi province, China
		Euplica scripta	JN052985	Beibhai, Guangxi province, China
	Mitrella	*Mitrella bicincta*	HQ834055	Yantai, Shandong province, China
		Mitrella bicincta	JN052991	Yantai, Shandong province, China
		Mitrella bicincta	JN052990	Yantai, Shandong province, China
		Mitrella bicincta	JN052989	Yantai, Shandong province, China
		Mitrella bicincta	JN052988	Yantai, Shandong province, China
		Mitrella bicincta	HM180683	Korea
		Mitrella bicincta	HM180684	Korea
		Mitrella bicincta	HM180685	Korea
		Mitrella bicincta	HM180686	Korea
		Mitrella bicincta	HM180687	Korea
		Mitrella bicincta	HM180688	Korea
		Mitrella bicincta	HM180689	Korea

		Mitrella bicincta	HM180690	Korea
		Mitrella bicincta	HM180691	Korea
		Mitrella bicincta	HM180692	Korea
		Mitrella burchardi	HQ834098	Zhoushan, Zhejiang province, China
		Mitrella burchardi	JN053028	Zhoushan, Zhejiang province, China
Conidae	*Conus*	*Conus aristophanes*	HQ834101	Beibhai, Guangxi province, China
		Conus betulinus	HQ834088	Zhanjiang, Guangdong province, China
		Conus betulinus	JN053043	Zhanjiang, Guangdong province, China
		Conus ebraeus	EF547576	Okinawa, Okinawa, Japan
		Conus ebraeus	EF547575	Okinawa, Okinawa, Japan
		Conus ebraeus	EF547574	Okinawa, Okinawa, Japan
		Conus ebraeus	EF547573	Okinawa, Okinawa, Japan
		Conus ebraeus	EF547572	Okinawa, Okinawa, Japan
		Conus ebraeus	EF547571	Okinawa, Okinawa, Japan
		Conus ebraeus	EF547570	Okinawa, Okinawa, Japan
		Conus ebraeus	EF547569	Okinawa, Okinawa, Japan
		Conus ebraeus	EF547568	Okinawa, Okinawa, Japan
		Conus ebraeus	EF547567	Okinawa, Okinawa, Japan
		Conus ebraeus	EF547566	Okinawa, Okinawa, Japan
		Conus ebraeus	EF547565	Okinawa, Okinawa, Japan
		Conus ebraeus	EF547564	Okinawa, Okinawa, Japan
		Conus ebraeus	EF547563	Okinawa, Okinawa, Japan
		Conus ebraeus	EF547562	Okinawa, Okinawa, Japan
		Conus ebraeus	EF547561	Okinawa, Okinawa, Japan
		Conus ebraeus	EF547560	Okinawa, Okinawa, Japan
		Conus ebraeus	EF547559	Okinawa, Okinawa, Japan
		Conus judaeus	EF108266	Japan
		Conus lividus	HQ852576	Okinawa, Okinawa, Japan
		Conus lividus	HQ852577	Okinawa, Okinawa, Japan
		Conus lividus	HQ852575	Okinawa, Okinawa, Japan
		Conus quercinus	HQ834087	Sanya, Hainan province, China

		Conus sanguinolentus	HQ834090	Qionghai, Hainan province, China
		Conus sanguinolentus	HQ852532	Okinawa, Okinawa, Japan
		Conus sanguinolentus	HQ852530	Okinawa, Okinawa, Japan
		Conus sanguinolentus	HQ852528	Okinawa, Okinawa, Japan
		Conus sanguinolentus	HQ852526	Okinawa, Okinawa, Japan
		Conus sanguinolentus	HQ852524	Okinawa, Okinawa, Japan
		Conus sanguinolentus	HQ852522	Okinawa, Okinawa, Japan
		Conus sanguinolentus	HQ852520	Okinawa, Okinawa, Japan
		Conus sanguinolentus	HQ852533	Okinawa, Okinawa, Japan
		Conus sanguinolentus	HQ852529	Okinawa, Okinawa, Japan
		Conus sanguinolentus	HQ852531	Okinawa, Okinawa, Japan
		Conus sanguinolentus	HQ852527	Okinawa, Okinawa, Japan
		Conus sanguinolentus	HQ852525	Okinawa, Okinawa, Japan
		Conus sanguinolentus	HQ852523	Okinawa, Okinawa, Japan
		Conus sanguinolentus	HQ852521	Okinawa, Okinawa, Japan
		Conus textile	HQ834089	Qionghai, Hainan province, China
Fasciolariidae	*Fusinus*	*Fusinus forceps*	HM180580	Korea
		Fusinus forceps	HM180581	Korea
		Fusinus forceps	HM180582	Korea
		Fusinus forceps	HM180583	Korea
		Fusinus forceps	HM180584	Korea
		Fusinus longicaudus	HQ834100	Zhangzhou, Fujian province, China
		Fusinus longicaudus	HQ834099	Lianyungang, Jiangsu province, China
		Fusinus longicaudus	HM180585	Korea
Melongenidae	*Hemifusus*	*Hemifusus colosseus*	HQ834068	Beibhai, Guangxi province, China
		Hemifusus colosseus	JN053018	Beibhai, Guangxi province, China
		Hemifusus ternatanus	HQ834067	Lianyungang, Jiangsu province, China
		Hemifusus ternatanus	JN053017	Lianyungang, Jiangsu province, China
		Hemifusus ternatanus	JN053016	Lianyungang, Jiangsu province, China
		Hemifusus ternatanus	JN053015	Lianyungang, Jiangsu province, China
		Hemifusus ternatanus	JN053014	Lianyungang, Jiangsu province, China

		Hemifusus ternatanus	HM180607	Korea
		Hemifusus ternatanus	HM180608	Korea
		Hemifusus ternatanus	HM180609	Korea
		Hemifusus tuba	HQ834070	Beibhai, Guangxi province, China
		Hemifusus tuba	HQ834069	Zhanjiang, Guangdong province, China
		Hemifusus tuba	JN053024	Zhanjiang, Guangdong province, China
		Hemifusus tuba	JN053023	Zhanjiang, Guangdong province, China
		Hemifusus tuba	JN053022	Zhanjiang, Guangdong province, China
		Hemifusus tuba	JN053021	Zhanjiang, Guangdong province, China
		Hemifusus tuba	JN053020	Zhanjiang, Guangdong province, China
		Hemifusus tuba	JN053019	Zhanjiang, Guangdong province, China
Muricidae	*Boreotrophon*	*Boreotrophon xestra*	HQ834056	Dalian, Liaoning province, China
		Boreotrophon xestra	JN052994	Dalian, Liaoning province, China
		Boreotrophon xestra	JN052993	Dalian, Liaoning province, China
		Boreotrophon xestra	JN052992	Dalian, Liaoning province, China
	Ceratostoma	*Ceratostoma rorifluum*	HM180413	Korea
		Ceratostoma rorifluum	HM180494	Korea
		Ceratostoma rorifluum	GU188257	Rongcheng, Shandong province, China
		Ceratostoma rorifluum	GU188258	Rongcheng, Shandong province, China
		Ceratostoma rorifluum	GU188259	Rongcheng, Shandong province, China
		Ceratostoma rorifluum	GU188260	Yantai, Shandong province, China
		Ceratostoma rorifluum	GU188261	Rongcheng, Shandong province, China
		Ceratostoma rorifluum	GU188262	Rongcheng, Shandong province, China
	Chicoreus	*Chicoreus asianus*	GU188200	Beibhai, Guangxi province, China
		Chicoreus asianus	GU188201	Beibhai, Guangxi province, China
		Chicoreus asianus	GU188202	Beibhai, Guangxi province, China
		Chicoreus asianus	GU188203	Beibhai, Guangxi province, China
		Chicoreus asianus	GU188204	Lingao, Hainan province, China
		Chicoreus asianus	GU188205	Pingtan, Fujian province, China
		Chicoreus torrefactus	GU188208	Beibhai, Guangxi province, China
		Chicoreus torrefactus	GU188209	Beibhai, Guangxi province, China

	Chicoreus torrefactus	GU188210	Beibhai, Guangxi province, China
	Chicoreus torrefactus	GU188211	Wenchang, Hainan province, China
Drupa	*Drupa albolabris*	HE584499	Tsuchihama, Kasari, Amami, Japan
	Drupa albolabris	HE584498	Chichijima, Ogasawara Is, Japan
	Drupa grossularia	HE584488	Amami-O-Shima, Ryukyu Is, Japan
	Drupa rubusidaeus	HE584527	Kakeroma, Ryukyu Is, Japan
	Drupa rubusidaeus	HE584526	Okinawa, Okinawa, Japan
Drupella	*Drupella cornus*	FR853820	Sakihara Is., Japan
	Drupella eburnea	FR853828	Sakaematsu, Kyushu Is, Japan
	Drupella fragum	FR853892	FukashimaOita Pref., Japan
	Drupella fragum	FR853890	FukashimaOita Pref., Japan
	Drupella fragum	FR853888	FukashimaOita Pref., Japan
	Drupella fragum	FR853886	FukashimaOita Pref., Japan
	Drupella fragum	FR853884	FukashimaOita Pref., Japan
	Drupella fragum	FR853846	FukashimaOita Pref., Japan
	Drupella fragum	FR853891	FukashimaOita Pref., Japan
	Drupella fragum	FR853889	FukashimaOita Pref., Japan
	Drupella fragum	FR853887	FukashimaOita Pref., Japan
	Drupella fragum	FR853885	FukashimaOita Pref., Japan
	Drupella fragum	FR853847	FukashimaOita Pref., Japan
	Drupella fragum	FR853845	FukashimaOita Pref., Japan
	Drupella margariticola	JN053037	Sanya, Hainan province, China
	Drupella margariticola	JN053036	Sanya, Hainan province, China
	Drupella margariticola	GU188268	Beibhai, Guangxi province, China
	Drupella margariticola	GU188269	Beibhai, Guangxi province, China
	Drupella margariticola	GU188270	Sanya, Hainan province, China
	Drupella margariticola	GU188271	Sanya, Hainan province, China
	Drupella margariticola	FR853858	Hong Kong, China
	Drupella margariticola	FR853859	Hong Kong, China
	Drupella margariticola	FR853857	Hong Kong, China
	Drupella margariticola	FR853823	Hong Kong, China

	Drupella margariticola	FR853862	Kyushu, Japan
	Drupella margariticola	FR853860	Kyushu Is., Japan
	Drupella margariticola	FR853861	Amami Is, Japan
	Drupella rugosa	FR853848	Hong Kong, China
	Drupella rugosa	FR853838	Hong Kong, China
	Drupella rugosa	FR853836	Hong Kong, China
	Drupella rugosa	FR853834	Hong Kong, China
	Drupella rugosa	FR853832	Hong Kong, China
	Drupella rugosa	FR853837	Hong Kong, China
	Drupella rugosa	FR853835	Hong Kong, China
	Drupella rugosa	FR853833	Hong Kong, China
	Drupella rugosa	FR853831	Hong Kong, China
	Drupella rugosa	FR853829	Kyushu Is., Japan
	Drupella rugosa	FR853827	Kyushu Is., Japan
Mancinella	*Mancinella echinata*	HE584343	Fukashima, Japan
	Mancinella siro	HE584344	Kagoshima, Japan
Morula	*Morula funiculata*	HE584047	Miyake Is., Japan
	Morula funiculata	HE584046	FukashimaOita Pref., Japan
	Morula funiculata	HE584045	Amami Is, Japan
	Morula granulata	JN053032	Sanya, Hainan province, China
	Morula granulata	JN053031	Sanya, Hainan province, China
	Morula granulata	JN053030	Sanya, Hainan province, China
	Morula granulata	JN053029	Sanya, Hainan province, China
	Morula japonica	HE584023	Sakihara Is. ,Amami Is, Japan
	Morula purpureocincta	HE584049	Kunigami, Okinawa, Japan
	Morula rugosa	JN053034	Sanya, Hainan province, China
	Morula rugosa	JN053033	Sanya, Hainan province, China
	Morula rumphiusi	HE584019	Yakushima I.,Kyushu, Japan
	Morula spinosa	HE584026	Miyazaki, Japan
	Morula spinosa	HE584025	Kagoshima, Japan
	Morula striata	HE584028	Yaeyama Is, Okinawa, Okinawa, Japan

	Morula striata	HE584027	Sakihara Is. ,Amami Is, Japan
	Morula zebrina	HE584033	Yakushima I.,Kyushu, Japan
Murex	*Murex trapa*	GU188195	Beibhai, Guangxi province, China
	Murex trapa	GU188196	Beibhai, Guangxi province, China
	Murex trapa	GU188197	Beibhai, Guangxi province, China
	Murex trapa	GU188198	Beibhai, Guangxi province, China
	Murex trapa	GU188199	Beibhai, Guangxi province, China
Nucella	*Nucella freycinetii*	AB743761	Erimo, Japan
	Nucella freycinetii	AB743760	Erimo, Japan
	Nucella freycinetii	AB743759	Erimo, Japan
	Nucella freycinetii	AB743758	Erimo, Japan
	Nucella freycinetii	AB743757	Erimo, Japan
	Nucella freycinetii	AB743756	Erimo, Japan
Ocinebrellus	*Ocinebrellus inornatus*	HM180491	Korea
	Ocinebrellus inornatus	HM180492	Korea
	Ocinebrellus inornatus	HM180493	Korea
Rapana	*Rapana bezoar*	FN677421	Tosa Bay, Japan
	Rapana bezoar	GU188166	Wenchang, Hainan province, China
	Rapana bezoar	GU188167	Zhanjiang, Guangdong province, China
	Rapana bezoar	GU188168	Zhanjiang, Guangdong province, China
	Rapana bezoar	GU188169	Zhanjiang, Guangdong province, China
	Rapana bezoar	GU188170	Yangjiang, Guangdong province, China
	Rapana rapiformis	GU188189	Beibhai, Guangxi province, China
	Rapana rapiformis	GU188190	Haikou, Hainan province, China
	Rapana rapiformis	GU188191	Haikou, Hainan province, China
	Rapana rapiformis	GU188192	Haikou, Hainan province, China
	Rapana rapiformis	GU188193	Haikou, Hainan province, China
	Rapana rapiformis	GU188194	Haikou, Hainan province, China
	Rapana venosa	HM180814	Korea
	Rapana venosa	HM180815	Korea
	Rapana venosa	HM180816	Korea

	Rapana venosa	GU188175	Ganyu, Jiangsu province, China
	Rapana venosa	GU188176	Ganyu, Jiangsu province, China
	Rapana venosa	GU188177	Lianyungang, Jiangsu province, China
	Rapana venosa	GU188178	Lianyungang, Jiangsu province, China
	Rapana venosa	GU188179	Lianyungang, Jiangsu province, China
	Rapana venosa	HE584367	Kumamoto, Kyushu, Japan
Reishia	*Reishia bronni*	HE584369	Miyazaki, Kyushu Is, Japan
	Reishia bronni	HE584368	Izu Is, Miyake I., Japan
	Reishia bronni	FR695722	Kanagawa Pier, Miura City, Aburatsubo, Japan
Thais	*Thais clavigera*	GU188218	Shengsi, Zhejiang province, China
	Thais clavigera	GU188213	Fangchenggang, Guangxi province, China
	Thais clavigera	GU188214	Rizhao, Shandong province, China
	Thais clavigera	GU188212	Zhanjiang, Guangdong province, China
	Thais clavigera	GU188216	Zhanjiang, Guangdong province, China
	Thais clavigera	GU188217	Qingdao, Shandong province, China
	Thais clavigera	HM180817	Korea
	Thais clavigera	HM180818	Korea
	Thais clavigera	HM180819	Korea
	Thais clavigera	HM180820	Korea
	Thais clavigera	HM180821	Korea
	Thais gradata	GU188228	Fangchenggang, Guangxi province, China
	Thais gradata	GU188229	Beibhai, Guangxi province, China
	Thais javanica	GU188230	Lingao, Hainan province, China
	Thais javanica	GU188231	Lingao, Hainan province, China
	Thais luteostoma	HM180822	Korea
	Thais luteostoma	HM180823	Korea
	Thais luteostoma	HM180824	Korea
	Thais luteostoma	HM180825	Korea
	Thais luteostoma	HM180826	Korea
	Thais luteostoma	HM180827	Korea
	Thais luteostoma	HM180828	Korea

		Thais luteostoma	HM180829	Korea
		Thais luteostoma	HM180830	Korea
		Thais luteostoma	HM180831	Korea
		Thais luteostoma	GU188232	Shengsi, Zhejiang province, China
		Thais luteostoma	GU188233	Shengsi, Zhejiang province, China
		Thais luteostoma	GU188234	Nanji, Zhejiang province, China
		Thais luteostoma	GU188235	Fangchenggang, Guangxi province, China
		Thais luteostoma	GU188236	Rizhao, Shandong province, China
		Thais luteostoma	GU188237	Rizhao, Shandong province, China
		Thais luteostoma	HE584372	Hong Kong, China
		Thais mutabilis	GU188248	Beibhai, Guangxi province, China
		Thais mutabilis	GU188249	Beibhai, Guangxi province, China
		Thais mutabilis	GU188250	Beibhai, Guangxi province, China
		Thais mutabilis	GU188251	Beibhai, Guangxi province, China
		Thais mutabilis	GU188252	Lianyungang, Jiangsu province, China
		Thais mutabilis	GU188253	Lianyungang, Jiangsu province, China
Nassariidae	*Nassarius*	*Nassarius conoidalis*	JQ975567	China
		Nassarius conoidalis	JQ975566	China
		Nassarius conoidalis	JQ975565	China
		Nassarius dorsatus	JQ975553	China
		Nassarius dorsatus	JQ975554	China
		Nassarius festivus	JQ975456	Qingdao, Shandong province, China
		Nassarius festivus	JQ975460	Qingdao, Shandong province, China
		Nassarius festivus	JQ975459	Qingdao, Shandong province, China
		Nassarius festivus	JQ975458	Qingdao, Shandong province, China
		Nassarius festivus	JQ975457	Qingdao, Shandong province, China
		Nassarius festivus	JQ975455	Qingdao, Shandong province, China
		Nassarius hepaticus	JQ975487	Zhanjiang, Guangdong province, China
		Nassarius hepaticus	JQ975491	Zhanjiang, Guangdong province, China
		Nassarius hepaticus	JQ975490	Zhanjiang, Guangdong province, China
		Nassarius hepaticus	JQ975489	Zhanjiang, Guangdong province, China

		Nassarius hepaticus	JQ975488	Zhanjiang, Guangdong province, China
		Nassarius hepaticus	JQ975486	Zhanjiang, Guangdong province, China
		Nassarius livescens	JQ975514	China
		Nassarius livescens	JQ975515	China
		Nassarius livescens	JQ975516	China
		Nassarius livescens	JQ975517	China
		Nassarius pullus	JQ975561	China
		Nassarius pullus	JQ975560	China
		Nassarius pullus	JQ975559	China
		Nassarius pullus	JQ975558	China
		Nassarius pullus	JQ975557	China
		Nassarius pullus	JQ975556	China
		Nassarius pullus	JQ975555	China
		Nassarius semiplicatus	JQ975564	China
		Nassarius semiplicatus	JQ975563	China
		Nassarius siquijorensis	JQ975552	Zhanjiang, Guangdong province, China
		Nassarius siquijorensis	HQ834076	Zhanjiang, Guangdong province, China
		Nassarius siquijorensis	JN053047	Zhanjiang, Guangdong province, China
	Varicinassa	*Varicinassa variciferus*	JQ975551	China
		Varicinassa variciferus	JQ975546	China
		Varicinassa variciferus	JQ975547	China
		Varicinassa variciferus	JQ975548	China
		Varicinassa variciferus	JQ975549	China
		Varicinassa variciferus	JQ975550	China
Terebridae	*Duplicaria*	*Duplicaria dussumieri*	HQ834094	Lianyungang, Jiangsu province, China
Turbinellidae	*Vasum*	*Vasum turbinellus*	JN053025	Sanya, Hainan province, China
		Vasum turbinellus	HQ834084	Sanya, Hainan province, China
Turridae	*Gemmula*	*Gemmula deshayesii*	HQ834092	Rizhao, Shandong province, China
Volutidae	*Melo*	*Melo melo*	JN053026	Fangchenggang, Guangxi province, China
		Melo melo	HQ834086	Fangchenggang, Guangxi province, China
		Melo melo	HQ834085	Fangchenggang, Guangxi province, China

			Melo melo	JN053027	Fangchenggang, Guangxi province, China
Neritoida	Neritiliidae	*Neritilia*	*Neritilia littoralis*	AB102710	Amami-ohshima, Hienhama, Japan
			Neritilia mimotoi	AB102711	Kagoshima, Japan
			Neritilia rubida	AB102712	Amami-ohshima, Yuwangama, Japan
Nudibranchia	Aeolidiidae	*Anteaeolidiella*	*Anteaeolidiella takanosimensis*	JX087530	Japan
			Anteaeolidiella takanosimensis	JX087529	Japan
		Protaeolidiella	*Protaeolidiella atra*	KP143676	Japan
			Protaeolidiella atra	KP143675	Japan
	Dorididae	*Cryptobranchia*	*Cryptobranchia kuragiensis*	AB543974	Hokkaido, Akkeshi, Hokkaido, Japan
			Cryptobranchia kuragiensis	AB238457	Hokkaido, Akkeshi, Hokkaido, Japan
	Polyceridae	*Roboastra*	*Roboastra gracilis*	EF142863	Okinawa, Okinawa, Japan
			Roboastra luteolineata	EF142861	Okinawa, Okinawa, Japan
		Tambja	*Tambja amakusana*	EF142877	Okinawa, Okinawa, Japan
			Tambja limaciformis	EF142878	Okinawa, Okinawa, Japan
			Tambja morosa	EF142867	Okinawa, Okinawa, Japan
			Tambja sagamiana	EF142870	Okinawa, Okinawa, Japan
Patellogastropoda	Acmaeidae	*Notoacmea*	*Notoacmea schrenckii*	HM180719	Korea
			Notoacmea schrenckii	HM180720	Korea
			Notoacmea schrenckii	HM180721	Korea
			Notoacmea schrenckii	HM180722	Korea
			Notoacmea schrenckii	HM180723	Korea
			Notoacmea schrenckii	HM180724	Korea
	Lottiidae	*Lottia*	*Lottia cassis*	KM221034	Rongcheng, Shandong province, China
			Lottia cassis	KM221036	Rongcheng, Shandong province, China
			Lottia cassis	KM221042	Rongcheng, Shandong province, China
			Lottia cassis	KM221043	Rongcheng, Shandong province, China
			Lottia cassis	KM221115	Rongcheng, Shandong province, China

	Lottia cassis	KM221116	Rongcheng, Shandong province, China
	Lottia dorsuosa	KM221054	Zhoushan, Zhejiang province, China
	Lottia dorsuosa	KM221109	Zhoushan, Zhejiang province, China
	Lottia dorsuosa	KM221108	Zhoushan, Zhejiang province, China
	Lottia kogamogai	AB238467	Ibaraki, Japan
	Lottia langfordi	AB238468	Kochi, Goshikinohama, Japan
	Lottia lindbergi	AB238470	Hokkaido, Akkeshi, Hokkaido, Japan
	Lottia luchuana	KM221056	Nanji, Zhejiang province, China
	Lottia luchuana	KM221094	Yangjiang, Guangdong province, China
	Lottia luchuana	KM221044	Beibu Bay, Guangxi province, China
	Lottia luchuana	KM221045	Beibu Bay, Guangxi province, China
	Lottia luchuana	KM221048	Beibu Bay, Guangxi province, China
	Lottia luchuana	KM221049	Beibu Bay, Guangxi province, China
	Lottia luchuana	KM221101	Wenchang, Hainan province, China
	Lottia luchuana	KM221100	Wenchang, Hainan province, China
	Lottia luchuana	AB238471	Okinawa, Ogimi, Okinawa, Japan
	Lottia tenuisculpta	AB238482	Mie, Japan
Nipponacmea	*Nipponacmea concinna*	KM221103	Fuqing, Fujian province, China
	Nipponacmea fuscoviridis	KF953455	China
	Nipponacmea fuscoviridis	KF953456	China
	Nipponacmea fuscoviridis	KF953457	China
	Nipponacmea fuscoviridis	KF953458	China
	Nipponacmea fuscoviridis	KF953459	China
	Nipponacmea gloriosa	AB238488	Shizuoka, Japan
	Nipponacmea habei	AB238489	Iwate, Yamada, Japan
	Nipponacmea nigrans	KF953202	China
	Nipponacmea nigrans	KF953204	China
	Nipponacmea nigrans	KF953205	China
	Nipponacmea nigrans	KF953206	China
	Nipponacmea nigrans	KF953207	China
	Nipponacmea nigrans	AB238490	Ibaraki, Japan

	Nipponacmea radula	KF953288	China
	Nipponacmea radula	KF953289	China
	Nipponacmea radula	KF953290	China
	Nipponacmea radula	KF953291	China
	Nipponacmea radula	KF953292	China
	Nipponacmea radula	AB238491	Kagoshima, Japan
	Nipponacmea schrenckii	KM221040	Rongcheng, Shandong province, China
	Nipponacmea schrenckii	KM221114	Rongcheng, Shandong province, China
	Nipponacmea schrenckii	KM221128	Qingdao, Shandong province, China
	Nipponacmea schrenckii	KM221130	Qingdao, Shandong province, China
	Nipponacmea schrenckii	KM221131	Qingdao, Shandong province, China
	Nipponacmea schrenckii	KM221123	Qingdao, Shandong province, China
	Nipponacmea teramachii	AB238493	Mie, Japan
Patelloida	*Patelloida conulus*	AB161565	Aich, Tahara, Shiokawa, Japan
	Patelloida conulus	AB161566	Aich, Isshiki, Japan
	Patelloida conulus	AB161567	Oita, Japan
	Patelloida conulus	AB161568	Hiroshima, Fukuyama, Japan
	Patelloida conulus	AB161569	Kumamoto, Matsushima, Japan
	Patelloida conulus	AB161570	Kumamoto, Hondo, Japan
	Patelloida conulus	AB161571	Nagasaki,Minamikushiyama, Japan
	Patelloida conulus	AB161572	Kumamoto, Reihoku, Tomioka, Japan
	Patelloida conulus	AB161573	Fukuoka, Tsuyazaki, Japan
	Patelloida conulus	AB161574	Fukuoka, Tsuyazaki, Japan
	Patelloida conulus	AB161604	Aich, Tahara, Shiokawa, Japan
	Patelloida conulus	AB161605	Aich, Tahara, Shiokawa, Japan
	Patelloida conulus	AB161606	Aich, Tahara, Shiokawa, Japan
	Patelloida conulus	AB161607	Aich, Tahara, Shiokawa, Japan
	Patelloida conulus	AB161608	Aich, Tahara, Shiokawa, Japan
	Patelloida conulus	AB161609	Aich, Tahara, Shiokawa, Japan
	Patelloida conulus	AB161610	Aich, Tahara, Shiokawa, Japan
	Patelloida conulus	AB161611	Aich, Tahara, Shiokawa, Japan

Patelloida conulus	AB161612	Aich, Tahara, Shiokawa, Japan
Patelloida conulus	AB161613	Aich, Tahara, Shiokawa, Japan
Patelloida conulus	AB161614	Aich, Tahara, Shiokawa, Japan
Patelloida conulus	AB161615	Aich, Tahara, Shiokawa, Japan
Patelloida conulus	AB161616	Aich, Tahara, Shiokawa, Japan
Patelloida conulus	AB161617	Aich, Tahara, Shiokawa, Japan
Patelloida conulus	AB238514	Aich, Tahara, Japan
Patelloida heroldi	AB161575	Mie, Japan
Patelloida heroldi	AB161576	Wakayama, Japan
Patelloida heroldi	AB161577	Kanagawa, Hayama, Japan
Patelloida heroldi	AB161578	Kochi, Tosa, Usa, Japan
Patelloida heroldi	AB161579	Kagawa, Japan
Patelloida heroldi	AB161580	Aich, Hazu, Nakanohama, Japan
Patelloida heroldi	AB161581	Kanagawa, Miura, Japan
Patelloida heroldi	AB161582	Wakayama, Japan
Patelloida heroldi	AB161618	Aich, Hazu, Nakanohama, Japan
Patelloida heroldi	AB161619	Aich, Hazu, Nakanohama, Japan
Patelloida heroldi	AB161620	Aich, Hazu, Nakanohama, Japan
Patelloida heroldi	AB161621	Aich, Hazu, Nakanohama, Japan
Patelloida heroldi	AB161622	Aich, Hazu, Nakanohama, Japan
Patelloida heroldi	AB161623	Aich, Hazu, Nakanohama, Japan
Patelloida heroldi	AB161624	Aich, Hazu, Nakanohama, Japan
Patelloida heroldi	AB161625	Aich, Hazu, Nakanohama, Japan
Patelloida heroldi	AB161626	Aich, Hazu, Nakanohama, Japan
Patelloida heroldi	AB161627	Aich, Hazu, Nakanohama, Japan
Patelloida heroldi	AB161628	Aich, Hazu, Nakanohama, Japan
Patelloida heroldi	AB161629	Aich, Hazu, Nakanohama, Japan
Patelloida heroldi	AB161630	Aich, Hazu, Nakanohama, Japan
Patelloida heroldi	AB161631	Aich, Hazu, Nakanohama, Japan
Patelloida lentiginosa	AB238517	Okinawa, Benoki, Okinawa, Japan
Patelloida pygmaea	AB161552	Aich, Tahara, Shiokawa, Japan

Patelloida pygmaea	AB161553	Yamaguchi, Esaki, Japan
Patelloida pygmaea	AB161554	Fukushima, Japan
Patelloida pygmaea	AB161555	Ehime, Misho, Japan
Patelloida pygmaea	AB161556	Nagasaki,Tsushima, Japan
Patelloida pygmaea	AB161557	Chiba, Japan
Patelloida pygmaea	AB161560	Wakayama, Japan
Patelloida pygmaea	AB161561	Fukuoka, Tsuyazaki, Japan
Patelloida pygmaea	AB161558	Okayama, Japan
Patelloida pygmaea	AB161559	Hiroshima, Fukuyama, Japan
Patelloida pygmaea	AB161562	Kumamoto, Hondo, Japan
Patelloida pygmaea	AB161563	Kumamoto, Kawaura, Japan
Patelloida pygmaea	AB161564	Kumamoto, Matsushima, Japan
Patelloida pygmaea	AB161590	Aich, Tahara, Shiokawa, Japan
Patelloida pygmaea	AB161591	Aich, Tahara, Shiokawa, Japan
Patelloida pygmaea	AB161592	Aich, Tahara, Shiokawa, Japan
Patelloida pygmaea	AB161593	Aich, Tahara, Shiokawa, Japan
Patelloida pygmaea	AB161594	Aich, Tahara, Shiokawa, Japan
Patelloida pygmaea	AB161595	Aich, Tahara, Shiokawa, Japan
Patelloida pygmaea	AB161596	Aich, Tahara, Shiokawa, Japan
Patelloida pygmaea	AB161599	Aich, Tahara, Shiokawa, Japan
Patelloida pygmaea	AB161600	Aich, Tahara, Shiokawa, Japan
Patelloida pygmaea	AB161601	Aich, Tahara, Shiokawa, Japan
Patelloida pygmaea	AB161602	Aich, Tahara, Shiokawa, Japan
Patelloida pygmaea	AB161603	Aich, Tahara, Shiokawa, Japan
Patelloida pygmaea	KM221033	Yantai, Shandong province, China
Patelloida pygmaea	KM221111	Yantai, Shandong province, China
Patelloida pygmaea	KM221112	Yantai, Shandong province, China
Patelloida pygmaea	KM221113	Yantai, Shandong province, China
Patelloida pygmaea	KM221041	Rongcheng, Shandong province, China
Patelloida pygmaea	KM221147	Qingdao, Shandong province, China
Patelloida pygmaea	KM221148	Qingdao, Shandong province, China

		Patelloida pygmaea	KM221149	Qingdao, Shandong province, China
		Patelloida pygmaea	KM221150	Qingdao, Shandong province, China
		Patelloida ryukyuensis	KM221059	Nanji, Zhejiang province, China
		Patelloida ryukyuensis	KM221075	Fuqing, Fujian province, China
		Patelloida ryukyuensis	KM221076	Fuqing, Fujian province, China
		Patelloida ryukyuensis	KM221091	Yangjiang, Guangdong province, China
		Patelloida ryukyuensis	KM221092	Yangjiang, Guangdong province, China
		Patelloida ryukyuensis	KM221093	Yangjiang, Guangdong province, China
		Patelloida ryukyuensis	KM221117	Zhoushan, Zhejiang province, China
		Patelloida ryukyuensis	KM221099	Zhoushan, Zhejiang province, China
		Patelloida ryukyuensis	AB196508	Hong Kong, China
		Patelloida ryukyuensis	AB196509	Hong Kong, China
		Patelloida saccharina form lanx	KM221083	Zhangzhou, Fujian province, China
		Patelloida saccharina form lanx	KM221084	Zhangzhou, Fujian province, China
		Patelloida saccharina form lanx	KM221085	Zhangzhou, Fujian province, China
		Patelloida saccharina form lanx	KM221086	Zhangzhou, Fujian province, China
		Patelloida saccharina form lanx	KM221087	Zhangzhou, Fujian province, China
		Patelloida saccharina form lanx	KM221090	Yangjiang, Guangdong province, China
		Patelloida saccharina form lanx	KM221102	Wenchang, Hainan province, China
		Patelloida signata	AB161632	Aich, Hazu, Nakanohama, Japan
		Patelloida striata	AB161589	Okinawa, Ogimi, Okinawa, Japan
Nacellidae	*Cellana*	*Cellana grata*	GQ455948	Japan
		Cellana grata	GQ455949	Japan
		Cellana grata	GQ455950	Japan

Cellana grata	AB238546	Mie, Japan
Cellana grata	KM221067	Xiapu, Fujian province, China
Cellana grata	KM221072	Xiapu, Fujian province, China
Cellana grata	KM221155	Xiapu, Fujian province, China
Cellana grata	KM221156	Xiapu, Fujian province, China
Cellana grata	KM221095	Nanji, Zhejiang province, China
Cellana grata	KM221105	Nanji, Zhejiang province, China
Cellana grata	GQ455945	Hong Kong, China
Cellana grata	GQ455946	Hong Kong, China
Cellana mazatlandica	GQ455951	Ogasawara, Japan
Cellana mazatlandica	GQ455952	Ogasawara, Japan
Cellana mazatlandica	GQ455953	Ogasawara, Japan
Cellana mazatlandica	GQ455954	Ogasawara, Japan
Cellana mazatlandica	GQ455955	Ogasawara, Japan
Cellana mazatlandica	AB433635	Tokyo, Ogasawara Islands, Anejima, Japan
Cellana mazatlandica	AB433636	Tokyo, Ogasawara Islands, Hirajima, Japan
Cellana mazatlandica	AB433637	Tokyo, Ogasawara Islands, Hahajima, Japan
Cellana mazatlandica	AB433638	Tokyo, Ogasawara Islands, Hahajima, Japan
Cellana mazatlandica	AB433639	Tokyo, Ogasawara Islands, Mukojima, Japan
Cellana mazatlandica	AB433640	Tokyo, Ogasawara Islands, Chichi-jima, Japan
Cellana mazatlandica	AB433641	Tokyo, Ogasawara Islands, Mukojima, Japan
Cellana mazatlandica	AB433642	Tokyo, Ogasawara Islands, Chichi-jima, Japan
Cellana nigrolineata	AB548176	Chiba, Japan
Cellana nigrolineata	AB548177	Chiba, Japan
Cellana nigrolineata	AB548181	Ehime, Hiraura, Japan
Cellana nigrolineata	AB548182	Ehime, Hiraura, Japan
Cellana nigrolineata	AB548213	Fukui, Japan
Cellana nigrolineata	AB548165	Fukuoka, Hazu, Japan
Cellana nigrolineata	AB548166	Hyogo, Yura, Japan
Cellana nigrolineata	AB548167	Hyogo, Yura, Japan
Cellana nigrolineata	AB548162	Kagawa, Japan

Cellana nigrolineata	|AB548199	Kagoshima, Japan
Cellana nigrolineata	AB548200	Kagoshima, Japan
Cellana nigrolineata	AB548185	Kagoshima, Japan
Cellana nigrolineata	AB548196	Kagoshima, Japan
Cellana nigrolineata	AB548197	Kagoshima, Japan
Cellana nigrolineata	AB548183	Kagoshima, Japan
Cellana nigrolineata	AB548206	Kagoshima, Japan
Cellana nigrolineata	AB548207	Kagoshima, Japan
Cellana nigrolineata	AB548208	Kagoshima, Japan
Cellana nigrolineata	AB548209	Kagoshima, Japan
Cellana nigrolineata	AB548210	Kagoshima, Japan
Cellana nigrolineata	AB548184	Kagoshima, Japan
Cellana nigrolineata	AB548201	Kagoshima, Japan
Cellana nigrolineata	AB548202	Kagoshima, Japan
Cellana nigrolineata	AB548203	Kagoshima, Japan
Cellana nigrolineata	AB548204	Kagoshima, Japan
Cellana nigrolineata	AB548205	Kagoshima, Japan
Cellana nigrolineata	AB548192	Kagoshima, Japan
Cellana nigrolineata	AB548193	Kagoshima, Japan
Cellana nigrolineata	AB548194	Kagoshima, Japan
Cellana nigrolineata	AB548195	Kagoshima, Japan
Cellana nigrolineata	AB548198	Kagoshima, Japan
Cellana nigrolineata	AB548156	Kagoshima, Japan
Cellana nigrolineata	AB548190	Kagoshima, Japan
Cellana nigrolineata	AB548191	Kagoshima, Japan
Cellana nigrolineata	AB548168	Kanagawa, Hayama, Japan
Cellana nigrolineata	AB548169	Kanagawa, Hayama, Japan
Cellana nigrolineata	AB548178	Kochi, Hanemisaki, Japan
Cellana nigrolineata	AB548170	Kumamoto, Karakizaki, Japan
Cellana nigrolineata	AB548171	Kumamoto, Karakizaki, Japan
Cellana nigrolineata	AB548160	Kumamoto, Reihoku, Japan

Cellana nigrolineata	AB548159	Kumamoto, Reihoku, Japan
Cellana nigrolineata	AB548174	Miyagi, Ayukawa, Japan
Cellana nigrolineata	AB548175	Miyagi, Ayukawa, Japan
Cellana nigrolineata	AB548179	Miyazaki, Kyushu Is, Japan
Cellana nigrolineata	AB548180	Miyazaki, Kyushu Is, Japan
Cellana nigrolineata	AB548211	Miyazaki, Kyushu Is, Japan
Cellana nigrolineata	AB548212	Miyazaki, Kyushu Is, Japan
Cellana nigrolineata	AB548187	Oita, Japan
Cellana nigrolineata	AB548188	Oita, Japan
Cellana nigrolineata	AB548189	Oita, Japan
Cellana nigrolineata	AB548161	Shizuoka, Japan
Cellana nigrolineata	AB548186	Shizuoka, Japan
Cellana nigrolineata	AB548172	Shizuoka, Japan
Cellana nigrolineata	AB548173	Shizuoka, Japan
Cellana nigrolineata	AB548163	Shizuoka, Japan
Cellana nigrolineata	AB548157	Wakayama, Japan
Cellana nigrolineata	AB548158	Wakayama, Japan
Cellana nigrolineata	AB548164	Wakayama, Japan
Cellana nigrolineata	AB548154	Yamaguchi, Hikari, Japan
Cellana nigrolineata	AB548155	Yamaguchi, Hikari, Japan
Cellana nigrolineata	GQ455956	Japan
Cellana nigrolineata	AB238548	Mie, Japan
Cellana radiata	AB263731	Ogasawara Islands, Chichijima, John Beach, Japan
Cellana radiata	AB263730	Ogasawara Islands, Chichijima, John Beach, Japan
Cellana radiata	AB263729	Ogasawara Islands, Chichijima, Miyanohama, Japan
Cellana radiata	AB263728	Ogasawara Islands, Chichijima, Miyanohama, Japan
Cellana radiata	AB263727	Ogasawara Islands, Chichijima, Sakaiura, Japan
Cellana radiata	AB263726	Ogasawara Islands, Chichijima, Sakaiura, Japan
Cellana radiata	AB263725	Ogasawara Islands, Chichijima, Sakaiura, Japan
Cellana radiata	AB263724	Ogasawara Islands, Chichijima, Sakaiura, Japan
Cellana radiata	AB433644	Tokyo, Ogasawara Islands, Chichijima Sakaiura, Japan

			Cellana radiata	AB433645	Tokyo, Ogasawara Islands, Chichijima Sakaiura, Japan
			Cellana radiata	AB433646	Tokyo, Minami Iwojima, Japan
			Cellana radiata enneagona	GQ455964	Ogasawara, Japan
			Cellana radiata enneagona	GQ455965	Ogasawara, Japan
			Cellana testudinaria	AB238563	Okinawa, Okinawa, Japan
			Cellana toreuma	KM221163	Rongcheng, Shandong province, China
			Cellana toreuma	KM221118	Qingdao, Shandong province, China
			Cellana toreuma	KM221055	Shengsi, Zhejiang province, China
			Cellana toreuma	KM221053	Zhoushan, Zhejiang province, China
			Cellana toreuma	KM221057	Nanji, Zhejiang province, China
			Cellana toreuma	KM221078	Pingtan, Fujian province, China
			Cellana toreuma	KM221079	Pingtan, Fujian province, China
			Cellana toreuma	KM221066	Sanya, Hainan province, China
			Cellana toreuma	KM221162	Sanya, Hainan province, China
			Cellana toreuma	AB445032	Kumamoto, Reihoku, Japan
			Cellana toreuma	AB445031	Wakayama, Japan
			Cellana toreuma	AB445030	Kagawa, Japan
			Cellana toreuma	AB445029	Miyazaki, Kyushu Is, Japan
			Cellana toreuma	AB445028	Aomori, Tappizaki, Japan
			Cellana toreuma	AB445027	Miyagi, Ayukawa, Japan
			Cellana toreuma	AB445026	Kanagawa, Hayama, Japan
			Cellana toreuma	AB445025	Mie, Japan
			Cellana toreuma	AB445024	Aich, Morozaki, Japan
			Cellana toreuma	AB445020	Fukui, Japan
			Cellana toreuma	AB445019	Iwate, Yamada, Japan
			Cellana toreuma	GQ455984	Japan
			Cellana toreuma	GQ455985	Japan
			Cellana toreuma	AB238564	AkitaOga, Japan
Pulmonata	Siphonariidae	*Siphonaria*	*Siphonaria japonica*	KF716648	Lianyungang, Jiangsu province, China

			Siphonaria japonica	KF716623	Haikou, Hainan province, China
			Siphonaria japonica	KF716594	Dongshan, Guangdong province, China
			Siphonaria japonica	KF716568	Xiamen, Fujiang province, China
			Siphonaria japonica	KF716536	Hong Kong, China
			Siphonaria japonica	KF716747	Weihai, Shandong province, China
			Siphonaria japonica	KF716745	Weihai, Shandong province, China
			Siphonaria japonica	KF716718	Rizhao, Shandong province, China
			Siphonaria japonica	KF716717	Rizhao, Shandong province, China
			Siphonaria japonica	KF716701	Qingdao, Shandong province, China
			Siphonaria japonica	KF716700	Qingdao, Shandong province, China
			Siphonaria japonica	KF716679	Ningbo, Zhejiang province, China
Sacoglossa	Plakobranchidae	*Elysia*	*Elysia abei*	AB758955	Chiba, Japan
			Elysia abei	AB758954	Niigata, Japan
			Elysia abei	AB758953	Niigata, Japan
			Elysia abei	KC573711	Shirahama, Japan
			Elysia abei	KC573712	Shirahama, Japan
			Elysia abei	KC573713	Shirahama, Japan
			Elysia amakusana	AB758956	Niigata, Japan
			Elysia atroviridis	AB758942	Kanagawa, Miura, Japan
			Elysia atroviridis	AB758941	Kanagawa, Miura, Japan
			Elysia atroviridis	AB758940	Kanagawa, Miura, Japan
			Elysia atroviridis	AB758939	Kanagawa, Miura, Japan
			Elysia atroviridis	AB758938	Kanagawa, Miura, Japan
			Elysia atroviridis	AB758937	Kanagawa, Miura, Japan
			Elysia atroviridis	AB758936	Chiba, Japan
			Elysia atroviridis	AB758935	Chiba, Japan
			Elysia atroviridis	AB758934	Okayama, Japan
			Elysia atroviridis	AB758933	Okayama, Japan
			Elysia atroviridis	AB758932	Okayama, Japan
			Elysia atroviridis	AB758931	Okayama, Japan
			Elysia atroviridis	AB758930	Hiroshima, Mukaishima, Japan

Elysia atroviridis	AB758929	Hiroshima, Mukaishima, Japan
Elysia atroviridis	AB758928	Hiroshima, Mukaishima, Japan
Elysia atroviridis	AB758927	Hiroshima, Mukaishima, Japan
Elysia atroviridis	AB758926	Hiroshima, Mukaishima, Japan
Elysia atroviridis	AB758925	Hiroshima, Mukaishima, Japan
Elysia atroviridis	AB758924	Hiroshima, Mukaishima, Japan
Elysia atroviridis	AB758923	Hiroshima, Mukaishima, Japan
Elysia atroviridis	AB758922	Hiroshima, Mukaishima, Japan
Elysia atroviridis	AB758921	Okayama, Japan
Elysia atroviridis	AB758920	Okayama, Japan
Elysia atroviridis	AB758919	Okayama, Japan
Elysia atroviridis	AB758918	Okayama, Japan
Elysia atroviridis	AB758917	Okayama, Japan
Elysia atroviridis	AB758916	Okayama, Japan
Elysia atroviridis	AB758915	Japan
Elysia atroviridis	AB758914	Oita, Japan
Elysia atroviridis	AB758913	Kanagawa, Miura, Japan
Elysia atroviridis	AB758912	Kanagawa, Miura, Japan
Elysia atroviridis	AB758911	Chiba, Japan
Elysia atroviridis	AB758910	Chiba, Japan
Elysia atroviridis	AB758909	Okayama, Japan
Elysia atroviridis	AB758908	Okayama, Japan
Elysia atroviridis	AB758907	Okayama, Japan
Elysia atroviridis	AB758906	Okayama, Japan
Elysia atroviridis	AB758905	Okayama, Japan
Elysia atroviridis	AB758949	Oita, Japan
Elysia atroviridis	AB758948	Oita, Japan
Elysia atroviridis	AB758947	Oita, Japan
Elysia atroviridis	AB758946	Oita, Japan
Elysia atroviridis	AB758945	Yamaguchi, Tsunoshima, Japan
Elysia atroviridis	AB758944	Yamaguchi, Tsunoshima, Japan

			Elysia atroviridis	AB758943	Kanagawa, Miura, Japan
			Elysia atroviridis	KC573760	Choshi, Japan
			Elysia ornata	AB758967	Okinawa, Sobe, Okinawa, Japan
			Elysia ornata	AB758966	Niigata, Japan
			Elysia ornata	AB758965	Niigata, Japan
			Elysia ornata	AB758964	Chiba, Japan
			Elysia ornata	AB758963	Yamaguchi, Tsunoshima, Japan
			Elysia ornata	AB758962	Mie, Japan
			Elysia pusilla	AB758959	Okinawa, Hamahiga, Okinawa, Japan
			Elysia pusilla	AB758958	Okinawa, Hamahiga, Okinawa, Japan
			Elysia pusilla	AB758957	Okinawa, Chinen, Okinawa, Japan
			Elysia rufescens	AB758961	Okinawa, Sobe, Okinawa, Japan
			Elysia rufescens	AB758960	Chiba, Japan
			Elysia setoensis	KC573761	Kanagawa, Japan
			Elysia trisinuata	AB758951	Niigata, Japan
		Plakobranchus	*Plakobranchus ocellatus*	AB758971	Okinawa, Toguchi, Okinawa, Japan
			Plakobranchus ocellatus	AB758970	Okinawa, Toguchi, Okinawa, Japan
			Plakobranchus ocellatus	AB758969	Okinawa, Kouri, Okinawa, Japan
			Plakobranchus ocellatus	AB758968	Okinawa, Sobe, Okinawa, Japan
			Plakobranchus ocellatus	AB501307	Okinawa, Okinawa, Japan
	Limapontiidae	*Stiliger*	*Stiliger ornatus*	AB501311	Okinawa, Okinawa, Japan
			Stiliger smaragdinus	AB501310	Kanagawa, Japan
	Elysiidae	*Thuridilla*	*Thuridilla gracilis*	AB758972	Okinawa, Hamahiga, Okinawa, Japan
			Thuridilla splendens	AB758973	Okinawa, Kouri, Okinawa, Japan
Sorbeoconcha	Batillariidae	*Batillaria*	*Batillaria cumingii*	JF693342	Fuqing, Fujian province, China
			Batillaria cumingii	JF693341	Rongcheng, Shandong province, China
			Batillaria cumingii	JF693343	Rongcheng, Shandong province, China
			Batillaria cumingii	AB535194	Miyagi, Nagazuraura Lagoon, Japan
			Batillaria cumingii	AB535195	Miyagi, Nagazuraura Lagoon, Japan
			Batillaria zonalis	JF693339	Qingdao, Shandong province, China
			Batillaria zonalis	JF693340	Qingdao, Shandong province, China

Cerithiidae	*Clypeomorus*	*Clypeomorus humilis*	JF693365	Qingdao, Shandong province, China
		Clypeomorus humilis	JF693366	Qingdao, Shandong province, China
	Cerithium	*Cerithium traillii*	JF693363	Beibhai, Guangxi province, China
		Cerithium traillii	JF693364	Beibhai, Guangxi province, China
	Rhinoclavis	*Rhinoclavis sinensis*	JF693360	Beibhai, Guangxi province, China
		Rhinoclavis sinensis	JF693361	Lingao, Hainan province, China
		Rhinoclavis sinensis	JF693362	Lingao, Hainan province, China
Planaxidae	*Planaxis*	*Planaxis sulcatus*	JF693413	Weizhou, Guangxi province, China
		Planaxis sulcatus	JF693411	Fangchenggang, Guangxi province, China
		Planaxis sulcatus	JF693414	Wenchang, Hainan province, China
		Planaxis sulcatus	JF693412	Sanya, Hainan province, China
		Planaxis sulcatus	JF693415	Sanya, Hainan province, China
Potamididae	*Cerithidea*	*Cerithidea alata*	HE680304	Yokaku Bay, Kumamoto Pref., Japan
		Cerithidea alata	HE680305	Yokaku Bay, Kumamoto Pref., Japan
		Cerithidea alata	HE680306	Yokaku Bay, Kumamoto Pref., Japan
		Cerithidea alata	HE680307	Yokaku Bay, Kumamoto Pref., Japan
		Cerithidea alata	HE680308	Yokaku Bay, Kumamoto Pref., Japan
		Cerithidea alata	HE680309	Yokaku Bay, Kumamoto Pref., Japan
		Cerithidea alata	HE680310	Yokaku Bay, Kumamoto Pref., Japan
		Cerithidea alata	HE680311	Yokaku Bay, Kumamoto Pref., Japan
		Cerithidea cingulata	HE680432	Shioya Bay,Okinawa, Okinawa, Japan
		Cerithidea cingulata	HE680433	Shioya Bay, Okinawa, Okinawa, Japan
		Cerithidea cingulata	HE680434	Tsuyazaki, Fukuoka Pref., Japan
		Cerithidea cingulata	HE680435	Tsuyazaki, Fukuoka Pref., Japan
		Cerithidea cingulata	HE680436	Tsuyazaki, Fukuoka Pref., Japan
		Cerithidea cingulata	HE680437	Tsuyazaki, Fukuoka Pref., Japan
		Cerithidea cingulata	HE680438	Tsuyazaki, Fukuoka Pref., Japan
		Cerithidea cingulata	HE680439	Tsuyazaki, Fukuoka Pref., Japan
		Cerithidea cingulata	HE680440	Tsuyazaki, Fukuoka Pref., Japan
		Cerithidea cingulata	HE680441	Tsuyazaki, Fukuoka Pref., Japan
		Cerithidea cingulata	HE680442	Tsuyazaki, Fukuoka Pref., Japan

Cerithidea cingulata	HE680443	Tsuyazaki, Fukuoka Pref., Japan
Cerithidea cingulata	HE680444	Tsuyazaki, Fukuoka Pref., Japan
Cerithidea cingulata	HE680445	Tsuyazaki, Fukuoka Pref., Japan
Cerithidea cingulata	HE680446	Tsuyazaki, Fukuoka Pref., Japan
Cerithidea cingulata	HE680447	Tsuyazaki, Fukuoka Pref., Japan
Cerithidea cingulata	HE680448	Tsuyazaki, Fukuoka Pref., Japan
Cerithidea cingulata	HE680449	Tsuyazaki, Fukuoka Pref., Japan
Cerithidea cingulata	HE680450	Tsuyazaki, Fukuoka Pref., Japan
Cerithidea cingulata	HE680451	Tsuyazaki, Fukuoka Pref., Japan
Cerithidea cingulata	HE680452	Tsuyazaki, Fukuoka Pref., Japan
Cerithidea cingulata	HE680454	Yokakuwan, Kawaura, Kumamoto Pref., Japan
Cerithidea cingulata	HE680455	Yokakuwan, Kawaura, Kumamoto Pref., Japan
Cerithidea djadjariensis	HE680559	Hikawa, Kumamoto Pref., Kyushu, Japan
Cerithidea djadjariensis	HE680560	Hikawa, Kumamoto Pref., Kyushu, Japan
Cerithidea djadjariensis	HE680561	Hikawa, Kumamoto Pref., Kyushu, Japan
Cerithidea djadjariensis	HE680562	Hikawa, Kumamoto Pref., Kyushu, Japan
Cerithidea djadjariensis	HE680563	Hikawa, Kumamoto Pref., Kyushu, Japan
Cerithidea djadjariensis	HE680564	Hikawa, Kumamoto Pref., Kyushu, Japan
Cerithidea djadjariensis	HE680565	Hikawa, Kumamoto Pref., Kyushu, Japan
Cerithidea djadjariensis	HE680566	Hikawa, Kumamoto Pref., Kyushu, Japan
Cerithidea djadjariensis	HE680567	Hikawa, Kumamoto Pref., Kyushu, Japan
Cerithidea djadjariensis	HE680568	Hikawa, Kumamoto Pref., Kyushu, Japan
Cerithidea djadjariensis	HE680569	Hikawa, Kumamoto Pref., Kyushu, Japan
Cerithidea djadjariensis	HE680557	Iriomote I., Ryukyu Is, Japan
Cerithidea djadjariensis	HE680558	Iriomote I., Ryukyu Is, Japan
Cerithidea djadjariensis	HE680556	Isahaya Bay, Nagasaki Pref., Japan
Cerithidea djadjariensis	HE680582	Isahaya Bay, Nagasaki Pref., Japan
Cerithidea djadjariensis	HE680583	Isahaya Bay, Nagasaki Pref., Japan
Cerithidea djadjariensis	HE680570	Kiire, Kagoshima Pref., Japan
Cerithidea djadjariensis	HE680571	Kiire, Kagoshima Pref., Japan
Cerithidea djadjariensis	HE680584	Kiire, Kagoshima Pref., Japan

Cerithidea djadjariensis	HE680572	Matsuzaka, Mie Pref., Japan
Cerithidea djadjariensis	HE680573	Matsuzaka, Mie Pref., Japan
Cerithidea djadjariensis	HE680574	Matsuzaka, Mie Pref., Japan
Cerithidea djadjariensis	HE680575	Matsuzaka, Mie Pref., Japan
Cerithidea djadjariensis	HE680576	Matsuzaka, Mie Pref., Japan
Cerithidea djadjariensis	HE680577	Matsuzaka, Mie Pref., Japan
Cerithidea djadjariensis	HE680578	Matsuzaka, Mie Pref., Japan
Cerithidea djadjariensis	HE680579	Matsuzaka, Mie Pref., Japan
Cerithidea djadjariensis	HE680580	Matsuzaka, Mie Pref., Japan
Cerithidea djadjariensis	HE680581	Matsuzaka, Mie Pref., Japan
Cerithidea djadjariensis	HE680553	Senaga-jima,Okinawa Pref., Okinawa, Japan
Cerithidea djadjariensis	HE680554	Senaga-jima,Okinawa Pref., Okinawa, Japan
Cerithidea largillierti	AM932789	Lower Reaches of Ushitsu River, Nagata, Ashikari Town, Kyushu, Japan
Cerithidea ornate	AM932767	Sandybyakken, Japan
Cerithidea ornate	AM932766	Mouth of Hikawa River, Hikawa Town, Kyushu, Japan
Cerithidea ornate	HE680222	Hikawa, Kumamoto Pref., Kyushu, Japan
Cerithidea ornate	HE680223	Hikawa, Kumamoto Pref., Kyushu, Japan
Cerithidea ornate	HE680224	Hikawa, Kumamoto Pref., Kyushu, Japan
Cerithidea rhizophorarum	JF694694	South China Sea
Cerithidea rhizophorarum	JF694695	Taiwan Strait, Hsinchu, Taiwan, China
Cerithidea rhizophorarum	AM932774	Makiya, Okinawa, Japan
Cerithidea rhizophorarum	HE680254	Japan, Funaura
Cerithidea rhizophorarum	HE680255	Funaura, Japan
Cerithidea rhizophorarum	HE680257	Funaura, Japan
Cerithidea rhizophorarum	HE680258	Haneji, Okinawa, Ryukyu Is, Okinawa, Japan
Cerithidea rhizophorarum	HE680259	Hikawa, Kumamoto Pref., Kyushu, Japan
Cerithidea rhizophorarum	HE680260	Hikawa, Kumamoto Pref., Kyushu, Japan
Cerithidea rhizophorarum	HE680261	Fukuoka Pref., Imazu Bay, Japan
Cerithidea rhizophorarum	HE680262	Fukuoka Pref., Imazu Bay, Japan
Cerithidea rhizophorarum	HE680263	Fukuoka Pref., Imazu Bay, Japan

	Cerithidea rhizophorarum	HE680264	Fukuoka Pref., Imazu Bay, Japan
	Cerithidea rhizophorarum	HE680265	Fukuoka Pref., Imazu Bay, Japan
	Cerithidea rhizophorarum	HE680276	Isahaya Bay, Nagasaki Pref., Japan
	Cerithidea rhizophorarum	HE680277	Isahaya Bay, Nagasaki Pref., Japan
	Cerithidea rhizophorarum	HE680278	Isahaya Bay, Nagasaki Pref., Japan
	Cerithidea rhizophorarum	HE680279	Isahaya Bay, Nagasaki Pref., Japan
	Cerithidea rhizophorarum	HE680280	Isahaya Bay, Nagasaki Pref., Japan
	Cerithidea rhizophorarum	HE680281	Isahaya Bay, Nagasaki Pref., Japan
	Cerithidea rhizophorarum	HE680282	Isahaya Bay, Nagasaki Pref., Japan
	Cerithidea rhizophorarum	HE680271	Hiroshima Pref., Kasaoka Bay, Japan
	Cerithidea rhizophorarum	HE680272	Hiroshima Pref., Kasaoka Bay, Japan
	Cerithidea rhizophorarum	HE680273	Hiroshima Pref., Kasaoka Bay, Japan
	Cerithidea rhizophorarum	HE680274	Hiroshima Pref., Kasaoka Bay, Japan
	Cerithidea rhizophorarum	HE680275	Hiroshima Pref., Kasaoka Bay, Japan
	Cerithidea rhizophorarum	HE680270	Kiire, Kagoshima, Japan
	Cerithidea rhizophorarum	HE680266	Kiire, Kagoshima Pref., Japan
	Cerithidea rhizophorarum	HE680267	Kiire, Kagoshima Pref., Japan
	Cerithidea rhizophorarum	HE680268	Kiire, Kagoshima Pref., Japan
	Cerithidea rhizophorarum	HE680269	Kiire, Kagoshima Pref., Japan
	Cerithidea rhizophorarum	HE680283	Shiokawa, Aichi Pref., Japan
	Cerithidea rhizophorarum	HE680284	Shiokawa, Aichi Pref., Japan
	Cerithidea rhizophorarum	HE680285	Shiokawa, Aichi Pref., Japan
Terebralia	*Terebralia palustris*	HE680652	Ryukyu Is, HunauraIriomote I., Japan
	Terebralia sulcata	HE680663	Hong Kong, China
	Terebralia sulcata	HE680670	Haneji, Okinawa, Okinawa, Japan
	Terebralia sulcata	HE680630	Okinawa Pref., Kunigami-gun, Ginoza, Kanna R., Okinawa, Japan
	Terebralia sulcata	HE680667	Okinawa Pref., Kunigami-gun, Ginoza, Kanna R., Okinawa, Japan
	Terebralia sulcata	HE680668	Okinawa Pref., Kunigami-gun, Ginoza, Kanna R., Okinawa, Japan

			Terebralia sulcata	HE680669	Okinawa Pref., Kunigami-gun, Ginoza, Kanna R., Okinawa, Japan
			Terebralia sulcata	HE680671	Okinawa, Nago, Ooura R., Okinawa, Japan
	Turritellidae	*Turritella*	*Turritella terebra*	JF693443	Beibhai, Guangxi province, China
			Turritella terebra	JF693444	Wenzhou, Zhejiang province, China
			Turritella terebra	JF693445	Ningde, Fujian province, China
			Turritella terebra	JF693446	Beibhai, Guangxi province, China
			Turritella terebra	JF693447	Lianyungang, Jiangsu province, China
Systellommatophora	Onchidiidae	*Paraoncidium*	*Paraoncidium reevesii*	JN543145	Zhanjiang, Guangdong province, China
			Paraoncidium reevesii	JN543146	Cangnan, Zhejiang province, China
			Paraoncidium reevesii	JN543147	Hong Kong, China
			Paraoncidium reevesii	JN543148	Qinzhou, Guangxi province, China
			Paraoncidium reevesii	JN543149	Ningde, Fujian province, China
			Paraoncidium reevesii	JN543150	Haikou, Hainan province, China
			Paraoncidium reevesii	JN543151	Xiamen, Fujiang province, China
		Peronia	*Peronia verruculata*	JN543152	Zhanjiang, Guangdong province, China
			Peronia verruculata	JN543153	Ningde, Fujian province, China
			Peronia verruculata	JN543154	Haikou, Hainan province, China
		Platevindex	*Platevindex mortoni*	JN543136	Zhanjiang, Guangdong province, China
			Platevindex mortoni	JN543137	Ningde, Fujian province, China
			Platevindex mortoni	JN543138	Haikou, Hainan province, China
			Platevindex mortoni	JN543139	Qinzhou, Guangxi province, China
			Platevindex mortoni	JN543140	Xiamen, Fujiang province, China
			Platevindex mortoni	JN543141	Hong Kong, China
Thecosomata	Cavoliniidae	*Cuvierina*	*Cuvierina pacifica*	KP292770	Japan
			Cuvierina pacifica	KP292769	Japan
			Cuvierina pacifica	KP292768	Japan
			Cuvierina pacifica	KP292767	Japan
			Cuvierina pacifica	KP292766	Japan
Vetigastropoda	Calliotropidae	*Ginebis*	*Ginebis argenteonitens*	AB365231	Kanagawa, Misaki, Japan
			Ginebis argenteonitens	EU530112	Japan

				Ginebis argenteonitens	EU530111	Japan
			Granata	*Granata lyrata*	AB365232	Kagoshima, Japan
				Granata lyrata	EU530114	Japan
			Herpetopoma	*Herpetopoma pauperculus*	AB365233	Ibaraki, Japan
		Colloniidae	*Homalopoma*	*Homalopoma granuliferum*	AM049347	Shionomisaki, Wakayama Prefecture, Japan
				Homalopoma granuliferum	AB365217	Kanagawa, Misaki, Japan
				Homalopoma nocturnum	AM049348	Mitsuishi, Kanagawa Prefecture, Japan
				Homalopoma sangarense	AM049350	Ohtsuchi Bay, Iwate Prefecture, Japan
		Fissurellidae	*Emarginula*	*Emarginula foveolata*	AM049333	nland Sea, Hiroshima Prefecture, Japan
				Emarginula foveolata	AB238593	Hiroshima, Japan
				Emarginula variegata	AB365213	Okinawa, Okinawa, Japan
				Emarginula variegata	AB238594	Okinawa, Okinawa, Japan
			Macroschisma	*Macroschisma dilatata*	AM049334	Sugashima Island, Mie Prefecture, Japan
				Macroschisma dilatata	AB365212	Miyazaki, Japan
Polyplacophora	Acanthochitonina	Acanthochitonidae	*Acanthochitona*	*Acanthochitona achates*	HM180415	Korea
				Acanthochitona achates	HM180416	Korea
				Acanthochitona defilippi	HM180417	Korea
				Acanthochitona defilippi	HM180419	Korea
				Acanthochitona defilippi	HM180421	Korea
				Acanthochitona defilippi	HM180422	Korea
				Acanthochitona defilippi	HM180418	Korea
				Acanthochitona rubrolineata	HM180427	Korea
				Acanthochitona rubrolineata	HM180428	Korea
				Acanthochitona rubrolineata	HM180429	Korea
				Acanthochitona rubrolineata	HM180430	Korea

Supplementary Table 2. Species without a barcode gap. For each species, the mean and maximum intra-specific values are compared to the nearest neighbour distance.

Family	Species	Mean Intra-Sp	Max Intra-Sp	Nearest Species	Distance to NN
Veneridae	*Ruditapes variegata*	6.11	29.9	*Ruditapes philippinarum*	0
Cerithiidae	*Clypeomorus humilis*	25.03	25.03	*Planaxis sulcatus*	0.33
Acanthochitonidae	*Acanthochitona defilippi*	12.29	24.84	*Acanthochitona achates*	0.29
Columbellidae	*Euplica scripta*	12.13	24.14	*Conus aristophanes*	0.63
Mytilidae	*Mytilus galloprovincialis*	11.91	22.03	*Mytilus coruscus*	0
Veneridae	*Meretrix meretrix*	10.3	19.35	*Meretrix petechialis*	0
Plakobranchidae	*Elysia atroviridis*	1.8	17.61	*Elysia ornata*	0
Veneridae	*Paphia undulata*	3.24	17.44	*Paphia textile*	0
Muricidae	*Rapana bezoar*	4.87	16.56	*Rapana venosa*	0.46
Veneridae	*Periglypta puerpera*	2.77	14.64	*Periglypta compressa*	0
Lottiidae	*Nipponacmea nigrans*	4.77	14.23	*Nipponacmea concinna*	0.3
Conidae	*Conus sanguinolentus*	1.92	12.38	*Conus lividus*	0.31
Lottiidae	*Patelloida pygmaea*	8.25	20.31	*Patelloida conulus*	9.26
Plakobranchidae	*Elysia ornata*	7.44	10.96	*Elysia atroviridis*	0
Muricidae	*Thais luteostoma*	1.94	10.99	*Reishia bronni*	0.15
Nacellidae	*Cellana nigrolineata*	1.15	11.05	*Cellana grata*	0.51
Columbellidae	*Mitrella bicincta*	5.52	10.26	*Mitrella cf. tuberosa*	0.15
Veneridae	*Meretrix lusoria*	5.56	9.32	*Meretrix petechialis*	0
Idiosepiidae	*Idiosepius paradoxus*	1.91	9.35	*Idiosepius biserialis*	0.94
Littorinidae	*Littoraria intermedia*	11.14	16.79	*Littoraria scabra*	8.7
Nacellidae	*Cellana grata*	3.93	8.43	*Cellana nigrolineata*	0.51
Veneridae	*Meretrix petechialis*	3.7	7.7	*Meretrix lusoria*	0
Fasciolariidae	*Fusinus longicaudus*	4.73	6.69	*Fusinus forceps*	0.15
Veneridae	*Macridiscus aequilatera*	3.26	6.26	*Macridiscus semicancellata*	0

Corbiculidae	*Corbicula leana*	1.82	3.62	*Corbicula fluminea*	0
Littorinidae	*Littoraria scabra*	11.31	11.31	*Littoraria intermedia*	8.7
Veneridae	*Ruditapes philippinarum*	1.12	2.6	*Ruditapes variegata*	0
Acanthochitonidae	*Acanthochitona rubrolineata*	1.91	3	*Acanthochitona defilippi*	0.44
Sepiidae	*Sepiella maindroni*	3.11	3.11	*Sepiella japonica*	0.77
Mactridae	*Mactra veneriformis*	0.74	2.34	*Protothaca jedoensis*	0
Mactridae	*Coelomactra antiquata*	10.33	14.95	*Mactra cumingii*	12.93
Solenidae	*Solen grandis*	11.1	16.66	*Solen strictus*	14.71
Veneridae	*Macridiscus semicancellata*	0.85	1.9	*Macridiscus aequilatera*	0
Muricidae	*Reishia bronni*	1.53	2.01	*Thais luteostoma*	0.15
Trochidae	*Chlorostoma turbinatum*	0.38	1.61	*Omphalius rusticus*	0
Veneridae	*Gafrarium dispar*	3.78	14.74	*Gafrarium divaricatum*	13.19
Mytilidae	*Mytilus coruscus*	0.65	1.54	*Mytilus galloprovincialis*	0
Planaxidae	*Planaxis sulcatus*	1.12	1.81	*Clypeomorus humilis*	0.33
Muricidae	*Thais clavigera*	0.72	2.17	*Thais luteostoma*	0.91
Buccinidae	*Neptunea cumingi*	1.01	1.54	*Ocinebrellus inornatus*	0.3
Trochidae	*Omphalius rusticus rusticus*	1.22	1.22	*Chlorostoma turbinatum*	0
Fasciolariidae	*Fusinus forceps*	0.56	1.31	*Fusinus longicaudus*	0.15
Mytilidae	*Modiolus comptus*	0.92	1.54	*Modiolus nipponicus*	0.43
Lottiidae	*Nipponacmea radula*	0.4	1.08	*Nipponacmea schrenckii*	0
Nacellidae	*Cellana toreuma*	0.18	1.07	*Notoacmea schrenckii*	0
Acmaeidae	*Notoacmea schrenckii*	0.43	1.02	*Cellana toreuma*	0
Corbiculidae	*Corbicula fluminea*	0.47	0.95	*Corbicula leana*	0
Pteriidae	*Pinctada fucata*	0.22	0.8	*Pinctada martensi*	0
Veneridae	*Dosinia biscocta*	0.76	0.76	*Dosinia fibula*	0
Veneridae	*Macridiscus multifarius*	0.45	0.66	*Macridiscus aequilatera*	0
Veneridae	*Dosinia fibula*	0.28	0.63	*Dosinia biscocta*	0
Veneridae	*Paphia textile*	0.63	0.63	*Paphia undulata*	0
Muricidae	*Drupella margariticola*	2.95	8.89	*Drupella rugosa*	8.28
Plakobranchidae	*Plakobranchus ocellatus*	11.57	14.92	*Elysia ornata*	14.45
Lottiidae	*Nipponacmea schrenckii*	0.27	0.47	*Nipponacmea radula*	0

Nacellidae	*Cellana radiata*	0.16	0.46	*Cellana radiata enneagona*	0
Conidae	*Conus lividus*	0.52	0.77	*Conus sanguinolentus*	0.31
Octopodidae	*Octopus vulgaris*	0.12	0.32	*Octopus oshimai*	0
Columbellidae	*Mitrella cf. tuberosa*	0.46	0.46	*Mitrella bicincta*	0.15
Turbinidae	*Lunella coreensis*	0.1	0.31	*Lunella moniliformis*	0
Nassariidae	*Nassarius siquijorensis*	3.87	5.79	*Nassarius hepaticus*	5.5
Muricidae	*Ocinebrellus inornatus*	0.39	0.59	*Neptunea cumingi*	0.3
Muricidae	*Rapana venosa*	0.4	0.73	*Rapana bezoar*	0.46
Pteriidae	*Pinctada martensi*	0.25	0.25	*Pinctada fucata*	0
Nacellidae	*Cellana radiata enneagona*	0.16	0.16	*Cellana radiata*	0
Turbinidae	*Lunella coronata*	0.1	0.15	*Lunella granulata*	0
Turbinidae	*Lunella granulata*	0.08	0.15	*Lunella coronata*	0
Turbinidae	*Lunella moniliformis*	0.1	0.15	*Lunella coreensis*	0
Plakobranchidae	*Elysia abei*	0.96	1.39	*Elysia amakusana*	1.39
Veneridae	*Pelecyora isocardia*	0	0	*Pelecyora trigona*	0

Supplementary Table 3. Results of SPIDER package analysis. BINs delimitation and BOLD nearest neighbour.

Species	GenBank NO.		BM	BCM 0.01	BCM 0.021	BCM 0.053	ASB 0.01	ASB 0.021	ASB 0.053	NN
Acanthochitona achates	HM180415		*Acanthochitona achates*	ambiguous	ambiguous	ambiguous	ambiguous	ambiguous	ambiguous	*Acanthochitona defilippi*
Acanthochitona achates	HM180416		*Acanthochitona achates*	ambiguous	ambiguous	ambiguous	ambiguous	ambiguous	ambiguous	*Acanthochitona defilippi*
Acanthochitona rubrolineata	HM180427		*Acanthochitona achates*	ambiguous	ambiguous	ambiguous	ambiguous	ambiguous	ambiguous	*Acanthochitona defilippi*
Acanthochitona rubrolineata	HM180428	BOLD:ACB8074	*Acanthochitona rubrolineata*	correct	correct	correct	correct	ambiguous	ambiguous	*Acanthochitona defilippi*
Acanthochitona rubrolineata	HM180429		*Acanthochitona achates*	ambiguous	ambiguous	ambiguous	ambiguous	ambiguous	ambiguous	*Acanthochitona defilippi*
Acanthochitona rubrolineata	HM180430		*Acanthochitona rubrolineata*	correct	correct	correct	correct	ambiguous	ambiguous	*Acanthochitona defilippi*
Acanthochitona defilippi	HM180422		*Acanthochitona defilippi*	incorrect	incorrect	incorrect	incorrect	incorrect	incorrect	*Acanthochitona defilippi*
Acanthochitona defilippi	HM180417		*Acanthochitona defilippi*	correct	correct	correct	correct	correct	correct	*Liolophura japonica*
Acanthochitona defilippi	HM180418	BOLD:AAE6153	*Acanthochitona defilippi*	correct	correct	correct	correct	correct	correct	*Liolophura japonica*
Acanthochitona defilippi	HM180421		*Acanthochitona defilippi*	correct	correct	correct	correct	correct	correct	*Liolophura japonica*
Acanthochitona defilippi	HM180419	BOLD:AAE6152	*Acanthochitona defilippi*	no id	no id	no id	no id	no id	no id	*Liolophura japonica*
Adipicola crypta	AB539004		*Adipicola crypta*	correct	correct	correct	correct	correct	correct	*Adipicola crypta*
Adipicola crypta	AB257519		*Adipicola crypta*	correct	correct	correct	correct	correct	correct	*Adipicola crypta*
Adipicola crypta	AB257518	BOLD:AAD3750	*Adipicola crypta*	correct	correct	correct	correct	correct	correct	*Adipicola crypta*
Adipicola crypta	AB257517		*Adipicola crypta*	correct	correct	correct	correct	correct	correct	*Adipicola crypta*
Adipicola crypta	AB257516		*Adipicola crypta*	correct	correct	correct	correct	correct	correct	*Adipicola crypta*
Adipicola crypta	AB257515		*Adipicola crypta*	correct	correct	correct	correct	correct	correct	*Adipicola crypta*
Adipicola iwaotakii	AB257523		*Adipicola iwaotakii*	correct	correct	correct	correct	correct	correct	*Idas sp.*
Adipicola iwaotakii	AB257521		*Adipicola iwaotakii*	correct	correct	correct	correct	correct	correct	*Idas sp.*
Adipicola iwaotakii	AB257520	BOLD:AAA7140	*Adipicola iwaotakii*	correct	correct	correct	correct	correct	correct	*Idas sp.*
Adipicola iwaotakii	EU702325		*Adipicola iwaotakii*	correct	correct	correct	correct	correct	correct	*Idas sp.*
Adipicola iwaotakii	EU702324		*Adipicola iwaotakii*	correct	correct	correct	correct	correct	correct	*Idas sp.*
Adipicola iwaotakii	EU702323		*Adipicola iwaotakii*	no id	correct	correct	no id	correct	correct	*Idas sp.*
Adipicola pacifica	AB539005	BOLD:ACH5470	*Adipicola pacifica*	correct	correct	correct	correct	correct	correct	*Bathymodiolus platifrons*

Adipicola pacifica	HF545115		*Adipicola pacifica*	correct	correct	correct	correct	correct	correct	*Bathymodiolus platifrons*
Adipicola pacifica	AB170040		*Adipicola pacifica*	no id	correct	correct	no id	correct	correct	*Bathymodiolus platifrons*
Adipicola pacifica	AB257528		*Adipicola pacifica*	correct	correct	correct	correct	correct	correct	*Bathymodiolus platifrons*
Adipicola pacifica	AB257527		*Adipicola pacifica*	correct	correct	correct	correct	correct	correct	*Bathymodiolus platifrons*
Adipicola pacifica	AB257526		*Adipicola pacifica*	correct	correct	correct	correct	correct	correct	*Bathymodiolus platifrons*
Alcyna ocellata	AB505278	BOLD:ACB8409	*Alcyna ocellata*	correct	correct	correct	correct	correct	correct	*Alcyna ocellata*
Alcyna ocellata	AB505277		*Alcyna ocellata*	correct	correct	correct	correct	correct	correct	*Alcyna ocellata*
Amphioctopus aegina	HQ846132		*Amphioctopus aegina*	correct	correct	correct	correct	correct	correct	*Amphioctopus aegina*
Amphioctopus aegina	HQ846133		*Amphioctopus aegina*	correct	correct	correct	correct	correct	correct	*Amphioctopus aegina*
Amphioctopus aegina	HQ846134		*Amphioctopus aegina*	correct	correct	correct	correct	correct	correct	*Amphioctopus aegina*
Amphioctopus aegina	HQ846135	BOLD:AAM5036	*Amphioctopus aegina*	correct	correct	correct	correct	correct	correct	*Amphioctopus aegina*
Amphioctopus aegina	HQ846136		*Amphioctopus aegina*	correct	correct	correct	correct	correct	correct	*Amphioctopus aegina*
Amphioctopus aegina	HQ846137		*Amphioctopus aegina*	correct	correct	correct	correct	correct	correct	*Amphioctopus aegina*
Amphioctopus aegina	JX456267		*Amphioctopus aegina*	correct	correct	correct	correct	correct	correct	*Amphioctopus aegina*
Amphioctopus fangsiao	HQ846126		*Amphioctopus fangsiao*	correct	correct	correct	correct	correct	correct	*Amphioctopus fangsiao*
Amphioctopus fangsiao	HQ846127		*Amphioctopus fangsiao*	correct	correct	correct	correct	correct	correct	*Amphioctopus fangsiao*
Amphioctopus fangsiao	HQ846114	BOLD:AAE5989	*Amphioctopus fangsiao*	correct	correct	correct	correct	correct	correct	*Amphioctopus fangsiao*
Amphioctopus fangsiao	HQ846155		*Amphioctopus fangsiao*	correct	correct	correct	correct	correct	correct	*Amphioctopus fangsiao*
Amphioctopus fangsiao	AB430519		*Amphioctopus fangsiao*	correct	correct	correct	correct	correct	correct	*Amphioctopus fangsiao*
Amphioctopus fangsiao	AB430518	BOLD:ABX6367	*Amphioctopus fangsiao*	no id	no id	no id	no id	no id	no id	*Amphioctopus fangsiao*
Amphioctopus kagoshimensis	HQ846122		*Amphioctopus kagoshimensis*	correct	correct	correct	correct	correct	correct	*Amphioctopus aegina*
Amphioctopus kagoshimensis	HQ846123		*Amphioctopus kagoshimensis*	correct	correct	correct	correct	correct	correct	*Amphioctopus aegina*
Amphioctopus kagoshimensis	HQ846124		*Amphioctopus kagoshimensis*	correct	correct	correct	correct	correct	correct	*Amphioctopus aegina*
Amphioctopus kagoshimensis	HQ846125		*Amphioctopus kagoshimensis*	correct	correct	correct	correct	correct	correct	*Amphioctopus aegina*
Amphioctopus kagoshimensis	AB430520		*Amphioctopus kagoshimensis*	correct	correct	correct	correct	correct	correct	*Amphioctopus aegina*
Amphioctopus marginatus	HQ846138	BOLD:ABA8783	*Amphioctopus marginatus*	correct	correct	correct	correct	correct	correct	*Amphioctopus sp.*
Amphioctopus marginatus	HQ846139		*Amphioctopus marginatus*	correct	correct	correct	correct	correct	correct	*Amphioctopus sp.*
Amphioctopus marginatus	HQ846140		*Amphioctopus marginatus*	correct	correct	correct	correct	correct	correct	*Amphioctopus sp.*
Amphioctopus marginatus	HQ846141		*Amphioctopus marginatus*	correct	correct	correct	correct	correct	correct	*Amphioctopus sp.*
Amphioctopus marginatus	AB430522		*Amphioctopus marginatus*	correct	correct	correct	correct	correct	correct	*Amphioctopus sp.*
Amphioctopus marginatus	AB430521		*Amphioctopus marginatus*	correct	correct	correct	correct	correct	correct	*Amphioctopus sp.*

Amphioctopus ovulum	HQ846156	*Amphioctopus ovulum*	correct	correct	correct	correct	correct	correct	*Amphioctopus fangsiao*
Amphioctopus ovulum	HQ846157	*Amphioctopus ovulum*	correct	correct	correct	correct	correct	correct	*Amphioctopus fangsiao*
Amphioctopus ovulum	HQ846158	*Amphioctopus ovulum*	correct	correct	correct	correct	correct	correct	*Amphioctopus fangsiao*
Amphioctopus ovulum	HQ846159	*Amphioctopus ovulum*	correct	correct	correct	correct	correct	correct	*Amphioctopus fangsiao*
Amphioctopus ovulum	AB430524	*Amphioctopus ovulum*	no id	correct	correct	no id	correct	correct	*Amphioctopus fangsiao*
Amphioctopus ovulum	AB430523	*Amphioctopus ovulum*	correct	correct	correct	correct	correct	correct	*Amphioctopus fangsiao*
Amusium pleuronectes	GU120012	*Amusium pleuronectes*	correct	correct	correct	correct	correct	correct	
Amusium pleuronectes	GU120013	*Amusium pleuronectes*	correct	correct	correct	correct	correct	correct	
Amusium pleuronectes	GU120014	*Amusium pleuronectes*	correct	correct	correct	correct	correct	correct	
Amusium pleuronectes	GU120015	*Amusium pleuronectes*	correct	correct	correct	correct	correct	correct	
Amusium pleuronectes	GU120016	*Amusium pleuronectes*	correct	correct	correct	correct	correct	correct	
Amusium pleuronectes	GU120017	*Amusium pleuronectes*	correct	correct	correct	correct	correct	correct	
Amusium pleuronectes	GU120018	*Amusium pleuronectes*	correct	correct	correct	correct	correct	correct	
Amusium pleuronectes	GU120019	*Amusium pleuronectes*	correct	correct	correct	correct	correct	correct	
Anadara antiquata	KU341897	*Anadara antiquata*	correct	correct	correct	correct	correct	correct	
Anadara antiquata	KU341898	*Anadara antiquata*	correct	correct	correct	correct	correct	correct	
Anadara antiquata	KU341899	*Anadara antiquata*	correct	correct	correct	correct	correct	correct	
Anadara antiquata	KU341900	*Anadara antiquata*	correct	correct	correct	correct	correct	correct	
Anadara antiquata	KU341901	*Anadara antiquata*	correct	correct	correct	correct	correct	correct	
Anadara antiquata	KU341902	*Anadara antiquata*	correct	correct	correct	correct	correct	correct	
Anadara antiquata	KU341903	*Anadara antiquata*	correct	correct	correct	correct	correct	correct	
Anadara antiquata	KU341904	*Anadara antiquata*	correct	correct	correct	correct	correct	correct	
Anadara antiquata	KU341905	*Anadara antiquata*	correct	correct	correct	correct	correct	correct	
Anadara crebricostata	KU341891	*Anadara crebricostata*	correct	correct	correct	correct	correct	correct	
Anadara crebricostata	KU341892	*Anadara crebricostata*	correct	correct	correct	correct	correct	correct	
Anadara crebricostata	KU341893	*Anadara crebricostata*	correct	correct	correct	correct	correct	correct	
Anadara crebricostata	KU341894	*Anadara crebricostata*	correct	correct	correct	correct	correct	correct	
Anadara crebricostata	KU341895	*Anadara crebricostata*	correct	correct	correct	correct	correct	correct	
Anadara crebricostata	KU341896	*Anadara crebricostata*	correct	correct	correct	correct	correct	correct	
Anadara vellicata	KU341881	*Anadara vellicata*	correct	correct	correct	correct	correct	correct	
Anadara vellicata	KU341882	*Anadara vellicata*	correct	correct	correct	correct	correct	correct	

Anadara vellicata	KU341883	*Anadara vellicata*	correct	correct	correct	correct	correct	correct	
Anadara vellicata	KU341884	*Anadara vellicata*	correct	correct	correct	correct	correct	correct	
Anadara vellicata	KU341885	*Anadara vellicata*	correct	correct	correct	correct	correct	correct	
Anadara vellicata	KU341886	*Anadara vellicata*	correct	correct	correct	correct	correct	correct	
Anadara vellicata	KU341887	*Anadara vellicata*	correct	correct	correct	correct	correct	correct	
Anadara vellicata	KU341888	*Anadara vellicata*	correct	correct	correct	correct	correct	correct	
Anadara vellicata	KU341889	*Anadara vellicata*	correct	correct	correct	correct	correct	correct	
Anadara vellicata	KU341890	*Anadara vellicata*	correct	correct	correct	correct	correct	correct	
Angaria formosa	AM049342	*Angaria formosa*	no id	correct	correct	no id	correct	correct	*Angaria sp.*
Angaria formosa	AM049343	*Angaria formosa*	no id	correct	correct	no id	correct	correct	*Angaria sp.*
Anomalodiscus squamosus	HQ703048	*Anomalodiscus squamosus*	correct	correct	correct	correct	correct	correct	*Clausinella isabellina*
Anomalodiscus squamosus	HQ703049	*Anomalodiscus squamosus*	correct	correct	correct	correct	correct	correct	*Clausinella isabellina*
Anomalodiscus squamosus	HQ703050	*Anomalodiscus squamosus*	correct	correct	correct	correct	correct	correct	*Clausinella isabellina*
Anomalodiscus squamosus	HQ703051	*Anomalodiscus squamosus*	correct	correct	correct	correct	correct	correct	*Clausinella isabellina*
Anomalodiscus squamosus	HM124611	*Anomalodiscus squamosus*	correct	correct	correct	correct	correct	correct	*Clausinella isabellina*
Anteaeolidiella takanosimensis	JX087530	*Anteaeolidiella takanosimensis*	no id	correct	correct	no id	correct	correct	*Anteaeolidiella poshitra*
Anteaeolidiella takanosimensis	JX087529	*Anteaeolidiella takanosimensis*	no id	correct	correct	no id	correct	correct	*Anteaeolidiella poshitra*
Antigona lamellaris	HQ703334	*Antigona lamellaris*	correct	correct	correct	correct	correct	correct	*Eurhomalea lenticularis*
Antigona lamellaris	HQ703335	*Antigona lamellaris*	correct	correct	correct	correct	correct	correct	*Eurhomalea lenticularis*
Antigona lamellaris	HQ703336	*Antigona lamellaris*	correct	correct	correct	correct	correct	correct	*Eurhomalea lenticularis*
Antigona lamellaris	HQ703337	*Antigona lamellaris*	correct	correct	correct	correct	correct	correct	*Eurhomalea lenticularis*
Antigona lamellaris	HQ703338	*Antigona lamellaris*	correct	correct	correct	correct	correct	correct	*Eurhomalea lenticularis*
Antigona lamellaris	HQ703339	*Antigona lamellaris*	correct	correct	correct	correct	correct	correct	*Eurhomalea lenticularis*
Antigona lamellaris	HQ703340	*Antigona lamellaris*	correct	correct	correct	correct	correct	correct	*Eurhomalea lenticularis*
Antigona lamellaris	HQ703341	*Antigona lamellaris*	correct	correct	correct	correct	correct	correct	*Eurhomalea lenticularis*
Antigona lamellaris	HM124608	*Antigona lamellaris*	correct	correct	correct	correct	correct	correct	*Eurhomalea lenticularis*
Aplysia kurodai	JX560148	*Aplysia kurodai*	correct	correct	correct	correct	correct	correct	*Aplysia fasciata*
Aplysia kurodai	JX560147	*Aplysia kurodai*	correct	correct	correct	correct	correct	correct	*Aplysia fasciata*
Arca avellana	HM180481	*Arca avellana*	correct	correct	correct	correct	correct	correct	*Humilaria kennerleyi*
Arca avellana	HM180482	*Arca avellana*	correct	correct	correct	correct	correct	correct	*Humilaria kennerleyi*
Arca avellana	HM180483	*Arca avellana*	no id	correct	correct	no id	correct	correct	*Humilaria kennerleyi*

Arca navicularis	HQ258822	*Arca navicularis*	correct	correct	correct	correct	correct	correct	
Arca navicularis	HQ258823	*Arca navicularis*	correct	correct	correct	correct	correct	correct	
Arca navicularis	HQ258824	*Arca navicularis*	no id	correct	correct	no id	correct	correct	
Arca navicularis	KU341928	*Arca navicularis*	correct	correct	correct	correct	correct	correct	
Arca navicularis	KU341929	*Arca navicularis*	correct	correct	correct	correct	correct	correct	
Architeuthis dux	KC701762	*Architeuthis dux*	correct	correct	correct	correct	correct	correct	*Todaropsis eblanae*
Architeuthis dux	KC701757	*Architeuthis dux*	correct	correct	correct	correct	correct	correct	*Todaropsis eblanae*
Architeuthis dux	KC701751	*Architeuthis dux*	correct	correct	correct	correct	correct	correct	*Todaropsis eblanae*
Architeuthis dux	KC701741	*Architeuthis dux*	correct	correct	correct	correct	correct	correct	*Todaropsis eblanae*
Architeuthis dux	KC701731	*Architeuthis dux*	correct	correct	correct	correct	correct	correct	*Todaropsis eblanae*
Architeuthis dux	KC701730	*Architeuthis dux*	correct	correct	correct	correct	correct	correct	*Todaropsis eblanae*
Arcopsis interplicata	HQ258875	*Arcopsis interplicata*	correct	correct	correct	correct	correct	correct	
Arcopsis interplicata	HQ258876	*Arcopsis interplicata*	correct	correct	correct	correct	correct	correct	
Arcopsis interplicata	HQ258877	*Arcopsis interplicata*	correct	correct	correct	correct	correct	correct	
Arcopsis interplicata	HQ258878	*Arcopsis interplicata*	correct	correct	correct	correct	correct	correct	
Arcopsis interplicata	HQ258879	*Arcopsis interplicata*	no id	no id	correct	no id	no id	correct	
Argopecten irradians	GU120020	*Argopecten irradians*	correct	correct	correct	correct	correct	correct	
Argopecten irradians	GU120021	*Argopecten irradians*	correct	correct	correct	correct	correct	correct	
Argopecten irradians	GU120022	*Argopecten irradians*	correct	correct	correct	correct	correct	correct	
Argopecten irradians	GU120023	*Argopecten irradians*	correct	correct	correct	correct	correct	correct	
Argopecten irradians	GU120024	*Argopecten irradians*	correct	correct	correct	correct	correct	correct	
Argopecten irradians	GU120025	*Argopecten irradians*	correct	correct	correct	correct	correct	correct	
Atrina pectinata	AB059421	*Atrina pectinata*	correct	correct	correct	correct	correct	correct	*Atrina pectinata*
Atrina pectinata	AB059423	*Atrina pectinata*	correct	correct	correct	correct	correct	correct	*Atrina pectinata*
Atrina pectinata	AB059422	*Atrina pectinata*	correct	correct	correct	correct	correct	correct	*Atrina pectinata*
Atrina pectinata	AB059424	*Atrina pectinata*	no id	correct	correct	no id	correct	correct	*Atrina pectinata*
Atrina pectinata	AB076914	*Atrina pectinata*	correct	correct	correct	correct	correct	correct	*Atrina pectinata*
Babylonia areolata	JN053011	*Babylonia areolata*	correct	correct	correct	correct	correct	correct	*Babylonia areolata*
Babylonia areolata	HQ834066	*Babylonia areolata*	correct	correct	correct	correct	correct	correct	*Babylonia areolata*
Babylonia areolata	JN053013	*Babylonia areolata*	correct	correct	correct	correct	correct	correct	*Babylonia areolata*
Babylonia areolata	JN053012	*Babylonia areolata*	correct	correct	correct	correct	correct	correct	*Babylonia areolata*

Babylonia lutosa	JN053010	*Babylonia areolata*	no id	no id	no id	no id	no id	no id	*Babylonia areolata*
Barbatia fusca	AB050899	*Barbatia lacerata*	no id	no id	no id	no id	no id	no id	*Babylonia areolata*
Barbatia lacerata	HQ258828	*Barbatia lacerata*	correct	correct	correct	correct	correct	correct	
Barbatia lacerata	HQ258829	*Barbatia lacerata*	correct	correct	correct	correct	correct	correct	
Barbatia lacerata	HQ258830	*Barbatia lacerata*	no id	correct	correct	no id	correct	correct	
Barbatia lacerata	HQ258831	*Barbatia lacerata*	correct	correct	correct	correct	correct	correct	
Barbatia lacerata	HQ258832	*Barbatia lacerata*	correct	correct	correct	correct	correct	correct	
Barbatia lacerata	HQ258833	*Barbatia lacerata*	correct	correct	correct	correct	correct	correct	
Barbatia lacerata	HQ258834	*Barbatia lacerata*	correct	correct	correct	correct	correct	correct	
Barbatia lacerata	HQ258835	*Barbatia lacerata*	correct	correct	correct	correct	correct	correct	
Barbatia lacerata	HQ258826	*Barbatia lacerata*	correct	correct	correct	correct	correct	correct	
Barbatia lacerata	HQ258827	*Barbatia lacerata*	correct	correct	correct	correct	correct	correct	
Barbatia lacerata	HQ258836	*Barbatia lacerata*	no id	correct	correct	no id	correct	correct	
Barbatia lacerata	AB076932	*Barbatia lacerata*	no id	correct	correct	no id	correct	correct	*Barbatia tenera*
Barbatia lima	AB076931	*Arcopsis interplicata*	no id	no id	no id	no id	no id	no id	*Cucullaea labiata*
Barbatia virescens	KU341920	*Barbatia virescens*	correct	correct	correct	correct	correct	correct	
Barbatia virescens	KU341921	*Barbatia virescens*	correct	correct	correct	correct	correct	correct	
Barbatia virescens	KU341922	*Barbatia virescens*	correct	correct	correct	correct	correct	correct	
Barbatia virescens	KU341923	*Barbatia virescens*	correct	correct	correct	correct	correct	correct	
Barbatia virescens	KU341924	*Barbatia virescens*	correct	correct	correct	correct	correct	correct	
Barbatia virescens	KU341925	*Barbatia virescens*	correct	correct	correct	correct	correct	correct	
Barbatia virescens	KU341926	*Barbatia virescens*	correct	correct	correct	correct	correct	correct	
Barbatia virescens	KU341927	*Barbatia virescens*	correct	correct	correct	correct	correct	correct	
Barnea davidi	KJ125426	*Barnea dilatata*	no id	no id	no id	no id	no id	no id	*Barnea dilatata*
Barnea dilatata	KJ125414	*Barnea dilatata*	correct	correct	correct	correct	correct	correct	*Barnea davidi*
Barnea dilatata	KJ125415	*Barnea dilatata*	correct	correct	correct	correct	correct	correct	*Barnea davidi*
Bathymodiolus aduloides	AB170054	*Bathymodiolus aduloides*	correct	correct	correct	correct	correct	correct	*Bathymodiolus manusensis*
Bathymodiolus aduloides	AB170055	*Bathymodiolus aduloides*	correct	correct	correct	correct	correct	correct	*Bathymodiolus manusensis*
Bathymodiolus aduloides	AB170056	*Bathymodiolus aduloides*	correct	correct	correct	correct	correct	correct	*Bathymodiolus manusensis*
Bathymodiolus aduloides	AB170057	*Bathymodiolus aduloides*	correct	correct	correct	correct	correct	correct	*Bathymodiolus manusensis*
Bathymodiolus aduloides	AB170058	*Bathymodiolus aduloides*	correct	correct	correct	correct	correct	correct	*Bathymodiolus manusensis*

Bathymodiolus aduloides	AB170059	*Bathymodiolus aduloides*	correct	correct	correct	correct	correct	correct	*Bathymodiolus manusensis*
Bathymodiolus aduloides	HF545118	*Bathymodiolus aduloides*	correct	correct	correct	correct	correct	correct	*Bathymodiolus manusensis*
Bathymodiolus aduloides	AB597557	*Bathymodiolus aduloides*	correct	correct	correct	correct	correct	correct	*Bathymodiolus manusensis*
Bathymodiolus hirtus	AB250694	*Bathymodiolus hirtus*	correct	correct	correct	correct	correct	correct	*Idas sp.*
Bathymodiolus hirtus	AB170047	*Bathymodiolus hirtus*	correct	correct	correct	correct	correct	correct	*Idas sp.*
Bathymodiolus japonicus	HF545108	*Bathymodiolus japonicus*	correct	correct	correct	correct	correct	correct	*Bathymodiolus tangaroa*
Bathymodiolus japonicus	AB101422	*Bathymodiolus japonicus*	correct	correct	correct	correct	correct	correct	*Bathymodiolus tangaroa*
Bathymodiolus japonicus	AB101423	*Bathymodiolus japonicus*	correct	correct	correct	correct	correct	correct	*Bathymodiolus tangaroa*
Bathymodiolus platifrons	HF545106	*Bathymodiolus platifrons*	correct	correct	correct	correct	correct	ambiguous	*Bathymodiolus mauritanicus*
Bathymodiolus platifrons	AB101419	*Bathymodiolus platifrons*	no id	no id	correct	no id	no id	correct	*Bathymodiolus mauritanicus*
Bathymodiolus platifrons	AB101420	*Bathymodiolus platifrons*	correct	correct	correct	correct	correct	correct	*Bathymodiolus mauritanicus*
Bathymodiolus platifrons	AB101421	*Bathymodiolus platifrons*	correct	correct	correct	correct	correct	correct	*Bathymodiolus mauritanicus*
Bathymodiolus platifrons	AB250695	*Bathymodiolus platifrons*	correct	correct	correct	correct	correct	correct	*Bathymodiolus mauritanicus*
Bathymodiolus securiformis	AB170048	*Bathymodiolus securiformis*	no id	correct	correct	no id	correct	correct	*Gigantidas tangaroa*
Bathymodiolus securiformis	AB170051	*Bathymodiolus securiformis*	correct	correct	correct	correct	correct	correct	*Gigantidas tangaroa*
Bathymodiolus securiformis	AB170052	*Bathymodiolus securiformis*	correct	correct	correct	correct	correct	correct	*Gigantidas tangaroa*
Bathymodiolus securiformis	AB170053	*Bathymodiolus securiformis*	correct	correct	correct	correct	correct	correct	*Gigantidas tangaroa*
Bathymodiolus septemdierum	AB101424	*Bathymodiolus septemdierum*	no id	no id	correct	no id	no id	correct	
Bathymodiolus septemdierum	AB101426	*Bathymodiolus septemdierum*	no id	no id	correct	no id	no id	correct	
Bathymodiolus septemdierum	AB101427	*Bathymodiolus septemdierum*	no id	no id	correct	no id	no id	correct	
Bathymodiolus septemdierum	AB101425	*Bathymodiolus septemdierum*	correct	correct	correct	correct	correct	correct	*Bathymodiolus sp.*
Bathymodiolus septemdierum	AB101428	*Bathymodiolus septemdierum*	correct	correct	correct	correct	correct	correct	*Bathymodiolus sp.*
Bathymodiolus septemdierum	AB101429	*Bathymodiolus septemdierum*	correct	correct	correct	correct	correct	correct	*Bathymodiolus sp.*
Bathymodiolus septemdierum	AB101430	*Bathymodiolus septemdierum*	correct	correct	correct	correct	correct	correct	*Bathymodiolus sp.*
Bathymodiolus septemdierum	AB170041	*Bathymodiolus septemdierum*	correct	correct	correct	correct	correct	correct	*Bathymodiolus sp.*
Batillaria cumingii	JF693342	*Batillaria cumingii*	ambiguous	ambiguous	ambiguous	ambiguous	ambiguous	ambiguous	*Batillaria attramentaria*
Batillaria cumingii	JF693341	*Batillaria cumingii*	ambiguous	ambiguous	ambiguous	ambiguous	ambiguous	ambiguous	*Batillaria attramentaria*
Batillaria cumingii	JF693343	*Batillaria cumingii*	no id	ambiguous	ambiguous	no id	ambiguous	ambiguous	*Batillaria attramentaria*
Batillaria cumingii	AB535194	*Batillaria cumingii*	incorrect	incorrect	incorrect	incorrect	ambiguous	ambiguous	*Batillaria attramentaria*
Batillaria cumingii	AB535195	*Batillaria cumingii*	no id	ambiguous	ambiguous	no id	ambiguous	ambiguous	*Batillaria cumingii*
Batillaria zonalis	JF693339	*Batillaria zonalis*	correct	correct	correct	correct	correct	correct	*Batillaria cumingii*

Batillaria zonalis	JF693340	*Batillaria zonalis*	correct	correct	correct	correct	correct	correct	*Batillaria cumingii*
Benthomodiolus geikotsucola	AB679346	*Benthomodiolus geikotsucola*	correct	correct	correct	correct	correct	correct	*Benthomodiolus sp.*
Benthomodiolus geikotsucola	HF545103	*Benthomodiolus geikotsucola*	correct	correct	correct	correct	correct	correct	*Benthomodiolus sp.*
Berryteuthis anonychus	AB749277	*Gonatus kamtschaticus*	no id	no id	no id	no id	no id	no id	*Berryteuthis anonychus*
Berryteuthis magister	AB749281	*Gonatus madokai*	no id	no id	no id	no id	no id	no id	*Gonatopsis borealis*
Bonartemis histrio	HQ703153	*Bonartemis histrio*	no id	correct	correct	no id	correct	correct	*Dosinia troscheli*
Bonartemis histrio	HQ703156	*Bonartemis histrio*	correct	correct	correct	correct	correct	correct	*Dosinia victoriae*
Boreotrophon xestra	HQ834056	*Boreotrophon xestra*	correct	correct	correct	correct	correct	correct	*Boreotrophon clathratus*
Boreotrophon xestra	JN052994	*Boreotrophon xestra*	correct	correct	correct	correct	correct	correct	*Boreotrophon clathratus*
Boreotrophon xestra	JN052993	*Boreotrophon xestra*	no id	correct	correct	no id	correct	correct	*Boreotrophon clathratus*
Boreotrophon xestra	JN052992	*Boreotrophon xestra*	correct	correct	correct	correct	correct	correct	*Boreotrophon clathratus*
Broderipia iridescens	AB505284	*Broderipia iridescens*	correct	correct	correct	correct	correct	correct	*Lirularia iridescens*
Broderipia iridescens	EU530139	*Broderipia iridescens*	correct	correct	correct	correct	correct	correct	*Lirularia iridescens*
Buccinum pemphigum	JN052999	*Buccinum pemphigum*	correct	correct	correct	correct	correct	correct	*Buccinum yokomaruae*
Buccinum pemphigum	JN053000	*Buccinum pemphigum*	correct	correct	correct	correct	correct	correct	*Buccinum yokomaruae*
Buccinum pemphigum	JN053001	*Buccinum pemphigum*	correct	correct	correct	correct	correct	correct	*Buccinum yokomaruae*
Buccinum pemphigum	HQ834057	*Buccinum pemphigum*	correct	correct	correct	correct	correct	correct	*Buccinum yokomaruae*
Buccinum pemphigum	HQ834058	*Buccinum pemphigum*	no id	no id	correct	no id	no id	correct	*Buccinum yokomaruae*
Buccinum pemphigum	HQ834059	*Buccinum pemphigum*	correct	correct	correct	correct	correct	correct	*Buccinum yokomaruae*
Buccinum yokomaruae	JN052995	*Buccinum yokomaruae*	correct	correct	correct	correct	correct	correct	*Buccinum pemphigum*
Buccinum yokomaruae	JN052996	*Buccinum yokomaruae*	correct	correct	correct	correct	correct	correct	*Buccinum pemphigum*
Buccinum yokomaruae	JN052997	*Buccinum yokomaruae*	correct	correct	correct	correct	correct	correct	*Buccinum pemphigum*
Buccinum yokomaruae	JN052998	*Buccinum yokomaruae*	correct	correct	correct	correct	correct	correct	*Buccinum pemphigum*
Bursa granularis	JF693344	*Bursa granularis*	correct	correct	correct	correct	correct	correct	*Naticidae sp.*
Bursa granularis	JF693345	*Bursa granularis*	correct	correct	correct	correct	correct	correct	*Naticidae sp.*
Bursa granularis	JF693346	*Bursa granularis*	correct	correct	correct	correct	correct	correct	*Naticidae sp.*
Bursa granularis	JF693347	*Bursa granularis*	correct	correct	correct	correct	correct	correct	*Naticidae sp.*
Bursa granularis	JF693348	*Bursa granularis*	no id	no id	correct	no id	no id	correct	*Naticidae sp.*
Bursa rana	JF693349	*Bursa rana*	correct	correct	correct	correct	correct	correct	*Bursa granularis*
Bursa rana	JF693350	*Bursa rana*	correct	correct	correct	correct	correct	correct	*Bursa granularis*
Bursa rana	JF693351	*Bursa rana*	correct	correct	correct	correct	correct	correct	*Bursa granularis*

Bursa rana	JF693352	*Bursa rana*	correct	correct	correct	correct	correct	correct	*Bursa granularis*
Bursa rana	JF693353	*Bursa rana*	correct	correct	correct	correct	correct	correct	*Bursa granularis*
Calliostoma aculeatum	AB505271	*Calliostoma sakashitai*	no id	incorrect	incorrect	no id	incorrect	incorrect	*Calliostoma unicum*
Calliostoma sakashitai	AB365225	*Calliostoma aculeatum*	no id	incorrect	incorrect	no id	incorrect	incorrect	*Calliostoma unicum*
Calliostoma akoya	AB505272	*Calliostoma consors*	no id	no id	no id	no id	no id	no id	*Otukaia kiheiziebisu*
Calliostoma consors	FN435323	*Calliostoma haliarchus*	no id	no id	incorrect	no id	no id	incorrect	*Calliostoma haliarchus*
Calliostoma haliarchus	AB505273	*Calliostoma consors*	no id	no id	incorrect	no id	no id	incorrect	*Calliostoma unicum*
Calliostoma shinagawaensis	AB505275	*Calliostoma consors*	no id	no id	no id	no id	no id	no id	*Calliostoma unicum*
Callista brevisiphonata	HQ703037	*Callista brevisiphonata*	no id	no id	correct	no id	no id	correct	*Saxidomus purpuratus*
Callista brevisiphonata	HQ703038	*Callista brevisiphonata*	correct	correct	correct	correct	correct	correct	*Saxidomus purpuratus*
Callista brevisiphonata	HQ703039	*Callista brevisiphonata*	no id	correct	correct	no id	correct	correct	*Saxidomus purpuratus*
Callista brevisiphonata	HQ703040	*Callista brevisiphonata*	correct	correct	correct	correct	correct	correct	*Saxidomus purpuratus*
Callista brevisiphonata	JN898931	*Callista brevisiphonata*	no id	correct	correct	no id	correct	correct	*Saxidomus purpuratus*
Callista brevisiphonata	HM124569	*Callista brevisiphonata*	correct	correct	correct	correct	correct	correct	*Saxidomus purpuratus*
Callista chinensis	HQ703035	*Callista chinensis*	correct	correct	correct	correct	correct	correct	*Callista chione*
Callista chinensis	HQ703036	*Callista chinensis*	no id	no id	correct	no id	no id	correct	*Callista chione*
Callistoctopus aspilosomatis	AB430525	*Callistoctopus ornatus*	no id	no id	no id	no id	no id	no id	*Callistoctopus sp.*
Callistoctopus luteus	AB430526	*Callistoctopus luteus*	correct	correct	correct	correct	correct	correct	*Callistoctopus luteus*
Callistoctopus luteus	AB430527	*Callistoctopus luteus*	correct	correct	correct	correct	correct	correct	*Callistoctopus luteus*
Callistoctopus minor	HQ846113	*Callistoctopus minor*	ambiguous	ambiguous	ambiguous	ambiguous	ambiguous	ambiguous	*Octopus sp.*
Callistoctopus minor	HQ846115	*Callistoctopus minor*	ambiguous	ambiguous	ambiguous	ambiguous	ambiguous	ambiguous	*Octopus sp.*
Callistoctopus minor	HQ846116	*Callistoctopus minor*	ambiguous	ambiguous	ambiguous	ambiguous	ambiguous	ambiguous	*Octopus sp.*
Callistoctopus minor	HQ846117	*Callistoctopus minor*	ambiguous	ambiguous	ambiguous	ambiguous	ambiguous	ambiguous	*Octopus sp.*
Callistoctopus minor	HQ846118	*Callistoctopus minor*	ambiguous	ambiguous	ambiguous	ambiguous	ambiguous	ambiguous	*Octopus sp.*
Callistoctopus minor	HQ846119	*Callistoctopus minor*	ambiguous	ambiguous	ambiguous	ambiguous	ambiguous	ambiguous	*Octopus sp.*
Callistoctopus minor	AB430541	*Callistoctopus minor*	incorrect	incorrect	incorrect	incorrect	incorrect	incorrect	*Octopus sp.*
Callistoctopus ornatus	AB430528	*Callistoctopus aspilosomatis*	no id	no id	no id	no id	no id	no id	*Macrotritopus defilippi*
Calyptogena extenta	AB479085	*Calyptogena kawamurai*	no id	no id	no id	no id	no id	no id	*Calyptogena okutanii*
Calyptogena kawamurai	AB479089	*Calyptogena extenta*	no id	no id	no id	no id	no id	no id	*Calyptogena okutanii*
Calyptogena phaseoliformis	AB479088	*Calyptogena kawamurai*	no id	no id	no id	no id	no id	no id	*Calyptogena phaseoliformis*
Cantharidus bisbalteatus	AB505279	*Cantharidus jessoensis*	incorrect	incorrect	incorrect	incorrect	incorrect	incorrect	*Cantharidus callichroa*

Cantharidus jessoensis	AB505282	*Diloma suavis*	no id	no id	no id	no id	no id	no id	*Cantharidus callichroa*
Cantharidus callichroa	AB505280	*Cantharidus bisbalteatus*	incorrect	incorrect	incorrect	incorrect	incorrect	incorrect	*Cantharidus callichroa*
Cantharidus callichroa	EU530120	*Cantharidus callichroa*	no id	correct	correct	no id	correct	correct	*Cantharidus callichroa*
Cantharidus callichroa	AM049338	*Cantharidus callichroa*	no id	correct	correct	no id	correct	correct	*Cantharidus callichroa*
Cantharidus infuscatus	EU530119	*Cantharidus callichroa*	no id	correct	correct	no id	correct	correct	*Phorcus lineatus*
Cantharus cecillei	HQ834063	*Cantharus cecillei*	correct	correct	correct	correct	correct	correct	*Buccinidae sp.*
Cantharus cecillei	JN053007	*Cantharus cecillei*	correct	correct	correct	correct	correct	correct	*Buccinidae sp.*
Cantharus melanostomus	HQ834062	*Cantharus melanostomus*	correct	correct	correct	correct	correct	correct	*Cantharus spiralis*
Cantharus melanostomus	JN053038	*Cantharus melanostomus*	correct	correct	correct	correct	correct	correct	*Cantharus spiralis*
Cellana mazatlandica	GQ455951	*Cellana mazatlandica*	correct	correct	correct	correct	correct	correct	*Cellana grata*
Cellana mazatlandica	GQ455952	*Cellana mazatlandica*	correct	correct	correct	correct	correct	correct	*Cellana grata*
Cellana mazatlandica	GQ455953	*Cellana mazatlandica*	correct	correct	correct	correct	correct	correct	*Cellana grata*
Cellana mazatlandica	GQ455954	*Cellana mazatlandica*	no id	correct	correct	no id	correct	correct	*Cellana grata*
Cellana mazatlandica	GQ455955	*Cellana mazatlandica*	correct	correct	correct	correct	correct	correct	*Cellana grata*
Cellana mazatlandica	AB433635	*Cellana mazatlandica*	correct	correct	correct	correct	correct	correct	*Cellana grata*
Cellana mazatlandica	AB433636	*Cellana mazatlandica*	correct	correct	correct	correct	correct	correct	*Cellana grata*
Cellana mazatlandica	AB433637	*Cellana mazatlandica*	correct	correct	correct	correct	correct	correct	*Cellana grata*
Cellana mazatlandica	AB433638	*Cellana mazatlandica*	correct	correct	correct	correct	correct	correct	*Cellana grata*
Cellana mazatlandica	AB433639	*Cellana mazatlandica*	correct	correct	correct	correct	correct	correct	*Cellana grata*
Cellana mazatlandica	AB433640	*Cellana mazatlandica*	correct	correct	correct	correct	correct	correct	*Cellana grata*
Cellana mazatlandica	AB433641	*Cellana mazatlandica*	correct	correct	correct	correct	correct	correct	*Cellana grata*
Cellana mazatlandica	AB433642	*Cellana mazatlandica*	correct	correct	correct	correct	correct	correct	*Cellana grata*
Cellana grata	KM221067	*Cellana grata*	correct	correct	correct	correct	correct	correct	*Cellana mazatlandica*
Cellana grata	KM221072	*Cellana grata*	correct	correct	correct	correct	correct	correct	*Cellana mazatlandica*
Cellana grata	KM221155	*Cellana grata*	correct	correct	correct	correct	correct	correct	*Cellana mazatlandica*
Cellana grata	KM221156	*Cellana grata*	correct	correct	correct	correct	correct	correct	*Cellana mazatlandica*
Cellana grata	KM221095	*Cellana grata*	correct	correct	correct	correct	correct	correct	*Cellana mazatlandica*
Cellana grata	KM221105	*Cellana grata*	correct	correct	correct	correct	correct	correct	*Cellana mazatlandica*
Cellana grata	GQ455945	*Cellana grata*	correct	correct	correct	correct	correct	correct	*Cellana mazatlandica*
Cellana grata	GQ455946	*Cellana grata*	correct	correct	correct	correct	correct	correct	*Cellana mazatlandica*
Cellana grata	GQ455948	*Cellana grata*	ambiguous	ambiguous	ambiguous	ambiguous	ambiguous	ambiguous	*Cellana mazatlandica*

Cellana grata	GQ455949	*Cellana grata*	ambiguous	ambiguous	ambiguous	ambiguous	ambiguous	ambiguous	*Cellana mazatlandica*
Cellana grata	GQ455950	*Cellana grata*	ambiguous	ambiguous	ambiguous	ambiguous	ambiguous	ambiguous	*Cellana mazatlandica*
Cellana grata	AB238546	*Cellana grata*	ambiguous	ambiguous	ambiguous	ambiguous	ambiguous	ambiguous	*Cellana mazatlandica*
Cellana nigrolineata	AB548213	*Cellana nigrolineata*	incorrect	incorrect	incorrect	incorrect	incorrect	incorrect	*Cellana mazatlandica*
Cellana nigrolineata	AB548176	*Cellana nigrolineata*	ambiguous	ambiguous	ambiguous	ambiguous	ambiguous	ambiguous	*Cellana nigrolineata*
Cellana nigrolineata	AB548177	*Cellana nigrolineata*	ambiguous	ambiguous	ambiguous	ambiguous	ambiguous	ambiguous	*Cellana nigrolineata*
Cellana nigrolineata	AB548181	*Cellana nigrolineata*	correct	correct	correct	correct	ambiguous	ambiguous	*Cellana nigrolineata*
Cellana nigrolineata	AB548182	*Cellana nigrolineata*	correct	correct	correct	correct	ambiguous	ambiguous	*Cellana nigrolineata*
Cellana nigrolineata	AB548166	*Cellana nigrolineata*	ambiguous	ambiguous	ambiguous	ambiguous	ambiguous	ambiguous	*Cellana nigrolineata*
Cellana nigrolineata	AB548167	*Cellana nigrolineata*	ambiguous	ambiguous	ambiguous	ambiguous	ambiguous	ambiguous	*Cellana nigrolineata*
Cellana nigrolineata	AB548162	*Cellana nigrolineata*	correct	correct	correct	correct	ambiguous	ambiguous	*Cellana nigrolineata*
Cellana nigrolineata	AB548168	*Cellana nigrolineata*	correct	correct	correct	correct	ambiguous	ambiguous	*Cellana nigrolineata*
Cellana nigrolineata	AB548169	*Cellana nigrolineata*	correct	correct	correct	correct	ambiguous	ambiguous	*Cellana nigrolineata*
Cellana nigrolineata	AB548178	*Cellana nigrolineata*	ambiguous	ambiguous	ambiguous	ambiguous	ambiguous	ambiguous	*Cellana nigrolineata*
Cellana nigrolineata	AB548174	*Cellana nigrolineata*	correct	correct	correct	correct	ambiguous	ambiguous	*Cellana nigrolineata*
Cellana nigrolineata	AB548175	*Cellana nigrolineata*	correct	correct	correct	correct	ambiguous	ambiguous	*Cellana nigrolineata*
Cellana nigrolineata	AB548187	*Cellana nigrolineata*	correct	correct	correct	correct	ambiguous	ambiguous	*Cellana nigrolineata*
Cellana nigrolineata	AB548188	*Cellana nigrolineata*	correct	correct	correct	correct	ambiguous	ambiguous	*Cellana nigrolineata*
Cellana nigrolineata	AB548189	*Cellana nigrolineata*	correct	correct	correct	correct	ambiguous	ambiguous	*Cellana nigrolineata*
Cellana nigrolineata	AB548161	*Cellana nigrolineata*	correct	correct	correct	correct	ambiguous	ambiguous	*Cellana nigrolineata*
Cellana nigrolineata	AB548186	*Cellana nigrolineata*	ambiguous	ambiguous	ambiguous	ambiguous	ambiguous	ambiguous	*Cellana nigrolineata*
Cellana nigrolineata	AB548172	*Cellana nigrolineata*	correct	correct	correct	correct	ambiguous	ambiguous	*Cellana nigrolineata*
Cellana nigrolineata	AB548173	*Cellana nigrolineata*	correct	correct	correct	correct	ambiguous	ambiguous	*Cellana nigrolineata*
Cellana nigrolineata	AB548163	*Cellana nigrolineata*	ambiguous	ambiguous	ambiguous	ambiguous	ambiguous	ambiguous	*Cellana nigrolineata*
Cellana nigrolineata	AB548157	*Cellana nigrolineata*	correct	correct	correct	correct	ambiguous	ambiguous	*Cellana nigrolineata*
Cellana nigrolineata	AB548158	*Cellana nigrolineata*	correct	correct	correct	correct	ambiguous	ambiguous	*Cellana nigrolineata*
Cellana nigrolineata	AB548164	*Cellana nigrolineata*	ambiguous	ambiguous	ambiguous	ambiguous	ambiguous	ambiguous	*Cellana nigrolineata*
Cellana nigrolineata	AB548154	*Cellana nigrolineata*	correct	correct	correct	correct	ambiguous	ambiguous	*Cellana nigrolineata*
Cellana nigrolineata	AB548155	*Cellana nigrolineata*	correct	correct	correct	correct	ambiguous	ambiguous	*Cellana nigrolineata*
Cellana nigrolineata	GQ455956	*Cellana nigrolineata*	correct	correct	correct	correct	ambiguous	ambiguous	*Cellana nigrolineata*
Cellana nigrolineata	AB238548	*Cellana nigrolineata*	correct	correct	correct	correct	ambiguous	ambiguous	*Cellana nigrolineata*

Cellana nigrolineata	AB548199	*Cellana nigrolineata*	ambiguous	ambiguous	ambiguous	ambiguous	ambiguous	ambiguous	*Cellana nigrolineata*
Cellana nigrolineata	AB548200	*Cellana nigrolineata*	ambiguous	ambiguous	ambiguous	ambiguous	ambiguous	ambiguous	*Cellana nigrolineata*
Cellana nigrolineata	AB548185	*Cellana nigrolineata*	ambiguous	ambiguous	ambiguous	ambiguous	ambiguous	ambiguous	*Cellana nigrolineata*
Cellana nigrolineata	AB548196	*Cellana nigrolineata*	ambiguous	ambiguous	ambiguous	ambiguous	ambiguous	ambiguous	*Cellana nigrolineata*
Cellana nigrolineata	AB548197	*Cellana nigrolineata*	ambiguous	ambiguous	ambiguous	ambiguous	ambiguous	ambiguous	*Cellana nigrolineata*
Cellana nigrolineata	AB548183	*Cellana nigrolineata*	ambiguous	ambiguous	ambiguous	ambiguous	ambiguous	ambiguous	*Cellana nigrolineata*
Cellana nigrolineata	AB548206	*Cellana nigrolineata*	ambiguous	ambiguous	ambiguous	ambiguous	ambiguous	ambiguous	*Cellana nigrolineata*
Cellana nigrolineata	AB548207	*Cellana nigrolineata*	ambiguous	ambiguous	ambiguous	ambiguous	ambiguous	ambiguous	*Cellana nigrolineata*
Cellana nigrolineata	AB548208	*Cellana nigrolineata*	ambiguous	ambiguous	ambiguous	ambiguous	ambiguous	ambiguous	*Cellana nigrolineata*
Cellana nigrolineata	AB548209	*Cellana nigrolineata*	ambiguous	ambiguous	ambiguous	ambiguous	ambiguous	ambiguous	*Cellana nigrolineata*
Cellana nigrolineata	AB548210	*Cellana nigrolineata*	ambiguous	ambiguous	ambiguous	ambiguous	ambiguous	ambiguous	*Cellana nigrolineata*
Cellana nigrolineata	AB548184	*Cellana nigrolineata*	ambiguous	ambiguous	ambiguous	ambiguous	ambiguous	ambiguous	*Cellana nigrolineata*
Cellana nigrolineata	AB548201	*Cellana nigrolineata*	ambiguous	ambiguous	ambiguous	ambiguous	ambiguous	ambiguous	*Cellana nigrolineata*
Cellana nigrolineata	AB548202	*Cellana nigrolineata*	ambiguous	ambiguous	ambiguous	ambiguous	ambiguous	ambiguous	*Cellana nigrolineata*
Cellana nigrolineata	AB548203	*Cellana nigrolineata*	ambiguous	ambiguous	ambiguous	ambiguous	ambiguous	ambiguous	*Cellana nigrolineata*
Cellana nigrolineata	AB548204	*Cellana nigrolineata*	ambiguous	ambiguous	ambiguous	ambiguous	ambiguous	ambiguous	*Cellana nigrolineata*
Cellana nigrolineata	AB548205	*Cellana nigrolineata*	ambiguous	ambiguous	ambiguous	ambiguous	ambiguous	ambiguous	*Cellana nigrolineata*
Cellana nigrolineata	AB548192	*Cellana nigrolineata*	ambiguous	ambiguous	ambiguous	ambiguous	ambiguous	ambiguous	*Cellana nigrolineata*
Cellana nigrolineata	AB548193	*Cellana nigrolineata*	ambiguous	ambiguous	ambiguous	ambiguous	ambiguous	ambiguous	*Cellana nigrolineata*
Cellana nigrolineata	AB548194	*Cellana nigrolineata*	ambiguous	ambiguous	ambiguous	ambiguous	ambiguous	ambiguous	*Cellana nigrolineata*
Cellana nigrolineata	AB548195	*Cellana nigrolineata*	ambiguous	ambiguous	ambiguous	ambiguous	ambiguous	ambiguous	*Cellana nigrolineata*
Cellana nigrolineata	AB548198	*Cellana nigrolineata*	ambiguous	ambiguous	ambiguous	ambiguous	ambiguous	ambiguous	*Cellana nigrolineata*
Cellana nigrolineata	AB548156	*Cellana nigrolineata*	ambiguous	ambiguous	ambiguous	ambiguous	ambiguous	ambiguous	*Cellana nigrolineata*
Cellana nigrolineata	AB548190	*Cellana nigrolineata*	ambiguous	ambiguous	ambiguous	ambiguous	ambiguous	ambiguous	*Cellana nigrolineata*
Cellana nigrolineata	AB548191	*Cellana nigrolineata*	ambiguous	ambiguous	ambiguous	ambiguous	ambiguous	ambiguous	*Cellana nigrolineata*
Cellana nigrolineata	AB548170	*Cellana nigrolineata*	ambiguous	ambiguous	ambiguous	ambiguous	ambiguous	ambiguous	*Cellana nigrolineata*
Cellana nigrolineata	AB548171	*Cellana nigrolineata*	ambiguous	ambiguous	ambiguous	ambiguous	ambiguous	ambiguous	*Cellana nigrolineata*
Cellana nigrolineata	AB548160	*Cellana nigrolineata*	ambiguous	ambiguous	ambiguous	ambiguous	ambiguous	ambiguous	*Cellana nigrolineata*
Cellana nigrolineata	AB548159	*Cellana nigrolineata*	ambiguous	ambiguous	ambiguous	ambiguous	ambiguous	ambiguous	*Cellana nigrolineata*
Cellana nigrolineata	AB548179	*Cellana nigrolineata*	ambiguous	ambiguous	ambiguous	ambiguous	ambiguous	ambiguous	*Cellana nigrolineata*
Cellana nigrolineata	AB548180	*Cellana nigrolineata*	ambiguous	ambiguous	ambiguous	ambiguous	ambiguous	ambiguous	*Cellana nigrolineata*

Cellana nigrolineata	AB548211	*Cellana nigrolineata*	ambiguous	ambiguous	ambiguous	ambiguous	ambiguous	ambiguous	*Cellana nigrolineata*
Cellana nigrolineata	AB548212	*Cellana nigrolineata*	ambiguous	ambiguous	ambiguous	ambiguous	ambiguous	ambiguous	*Cellana nigrolineata*
Cellana nigrolineata	AB548165	*Cellana nigrolineata*	incorrect	incorrect	incorrect	incorrect	incorrect	incorrect	*Cellana nigrolineata*
Cellana radiata	AB263731	*Cellana radiata*	correct	correct	correct	correct	correct	correct	*Cellana toreuma*
Cellana radiata	AB263730	*Cellana radiata*	correct	correct	correct	correct	correct	correct	*Cellana toreuma*
Cellana radiata	AB263729	*Cellana radiata*	correct	correct	correct	correct	correct	correct	*Cellana toreuma*
Cellana radiata	AB263728	*Cellana radiata*	correct	correct	correct	correct	correct	correct	*Cellana toreuma*
Cellana radiata	AB263727	*Cellana radiata*	correct	correct	correct	correct	correct	correct	*Cellana toreuma*
Cellana radiata	AB263726	*Cellana radiata*	correct	correct	correct	correct	correct	correct	*Cellana toreuma*
Cellana radiata	AB263725	*Cellana radiata*	correct	correct	correct	correct	correct	correct	*Cellana toreuma*
Cellana radiata	AB263724	*Cellana radiata*	correct	correct	correct	correct	correct	correct	*Cellana toreuma*
Cellana radiata	AB433644	*Cellana radiata*	correct	correct	correct	correct	correct	correct	*Cellana toreuma*
Cellana radiata	AB433645	*Cellana radiata*	correct	correct	correct	correct	correct	correct	*Cellana toreuma*
Cellana radiata	AB433646	*Cellana radiata*	correct	correct	correct	correct	correct	correct	*Cellana toreuma*
Cellana radiata enneagona	GQ455964	*Cellana radiata enneagona*	correct	correct	correct	correct	correct	correct	*Cellana toreuma*
Cellana radiata enneagona	GQ455965	*Cellana radiata enneagona*	correct	correct	correct	correct	correct	correct	*Cellana toreuma*
Cellana testudinaria	AB238563	*Cellana mazatlandica*	no id	no id	no id	no id	no id	no id	*Cellana solida*
Cellana toreuma	KM221163	*Cellana toreuma*	ambiguous	ambiguous	ambiguous	ambiguous	ambiguous	ambiguous	*Cellana radiata*
Cellana toreuma	KM221118	*Cellana toreuma*	ambiguous	ambiguous	ambiguous	ambiguous	ambiguous	ambiguous	*Cellana radiata*
Cellana toreuma	KM221055	*Cellana toreuma*	ambiguous	ambiguous	ambiguous	ambiguous	ambiguous	ambiguous	*Cellana radiata*
Cellana toreuma	KM221053	*Cellana toreuma*	ambiguous	ambiguous	ambiguous	ambiguous	ambiguous	ambiguous	*Cellana radiata*
Cellana toreuma	KM221057	*Cellana toreuma*	ambiguous	ambiguous	ambiguous	ambiguous	ambiguous	ambiguous	*Cellana radiata*
Cellana toreuma	KM221078	*Cellana toreuma*	ambiguous	ambiguous	ambiguous	ambiguous	ambiguous	ambiguous	*Cellana radiata*
Cellana toreuma	KM221079	*Cellana toreuma*	ambiguous	ambiguous	ambiguous	ambiguous	ambiguous	ambiguous	*Cellana radiata*
Cellana toreuma	KM221066	*Cellana toreuma*	ambiguous	ambiguous	ambiguous	ambiguous	ambiguous	ambiguous	*Cellana radiata*
Cellana toreuma	KM221162	*Cellana toreuma*	ambiguous	ambiguous	ambiguous	ambiguous	ambiguous	ambiguous	*Cellana radiata*
Cellana toreuma	AB445032	*Cellana toreuma*	ambiguous	ambiguous	ambiguous	ambiguous	ambiguous	ambiguous	*Cellana radiata*
Cellana toreuma	AB445031	*Cellana toreuma*	ambiguous	ambiguous	ambiguous	ambiguous	ambiguous	ambiguous	*Cellana radiata*
Cellana toreuma	AB445030	*Cellana toreuma*	ambiguous	ambiguous	ambiguous	ambiguous	ambiguous	ambiguous	*Cellana radiata*
Cellana toreuma	AB445029	*Cellana toreuma*	ambiguous	ambiguous	ambiguous	ambiguous	ambiguous	ambiguous	*Cellana radiata*
Cellana toreuma	AB445028	*Cellana toreuma*	ambiguous	ambiguous	ambiguous	ambiguous	ambiguous	ambiguous	*Cellana radiata*

Cellana toreuma	AB445027	*Cellana toreuma*	ambiguous	ambiguous	ambiguous	ambiguous	ambiguous	ambiguous	*Cellana radiata*
Cellana toreuma	AB445026	*Cellana toreuma*	ambiguous	ambiguous	ambiguous	ambiguous	ambiguous	ambiguous	*Cellana radiata*
Cellana toreuma	AB445025	*Cellana toreuma*	ambiguous	ambiguous	ambiguous	ambiguous	ambiguous	ambiguous	*Cellana radiata*
Cellana toreuma	AB445024	*Cellana toreuma*	ambiguous	ambiguous	ambiguous	ambiguous	ambiguous	ambiguous	*Cellana radiata*
Cellana toreuma	AB445020	*Cellana toreuma*	ambiguous	ambiguous	ambiguous	ambiguous	ambiguous	ambiguous	*Cellana radiata*
Cellana toreuma	AB445019	*Cellana toreuma*	ambiguous	ambiguous	ambiguous	ambiguous	ambiguous	ambiguous	*Cellana radiata*
Cellana toreuma	GQ455984	*Cellana toreuma*	ambiguous	ambiguous	ambiguous	ambiguous	ambiguous	ambiguous	*Cellana radiata*
Cellana toreuma	GQ455985	*Cellana toreuma*	ambiguous	ambiguous	ambiguous	ambiguous	ambiguous	ambiguous	*Cellana radiata*
Cellana toreuma	AB238564	*Cellana toreuma*	ambiguous	ambiguous	ambiguous	ambiguous	ambiguous	ambiguous	*Cellana radiata*
Notoacmea schrenckii	HM180719	*Cellana toreuma*	ambiguous	ambiguous	ambiguous	ambiguous	ambiguous	ambiguous	*Cellana radiata*
Notoacmea schrenckii	HM180720	*Cellana toreuma*	ambiguous	ambiguous	ambiguous	ambiguous	ambiguous	ambiguous	*Cellana radiata*
Notoacmea schrenckii	HM180721	*Cellana toreuma*	ambiguous	ambiguous	ambiguous	ambiguous	ambiguous	ambiguous	*Cellana radiata*
Notoacmea schrenckii	HM180722	*Cellana toreuma*	ambiguous	ambiguous	ambiguous	ambiguous	ambiguous	ambiguous	*Cellana radiata*
Notoacmea schrenckii	HM180723	*Cellana toreuma*	ambiguous	ambiguous	ambiguous	ambiguous	ambiguous	ambiguous	*Cellana radiata*
Notoacmea schrenckii	HM180724	*Cellana toreuma*	ambiguous	ambiguous	ambiguous	ambiguous	ambiguous	ambiguous	*Cellana radiata*
Ceratostoma rorifluum	GU188257	*Ceratostoma rorifluum*	correct	correct	correct	correct	correct	correct	*Ocinebrellus inornatus*
Ceratostoma rorifluum	GU188258	*Ceratostoma rorifluum*	correct	correct	correct	correct	correct	correct	*Ocinebrellus inornatus*
Ceratostoma rorifluum	GU188259	*Ceratostoma rorifluum*	correct	correct	correct	correct	correct	correct	*Ocinebrellus inornatus*
Ceratostoma rorifluum	GU188260	*Ceratostoma rorifluum*	correct	correct	correct	correct	correct	correct	*Ocinebrellus inornatus*
Ceratostoma rorifluum	GU188261	*Ceratostoma rorifluum*	correct	correct	correct	correct	correct	correct	*Ocinebrellus inornatus*
Ceratostoma rorifluum	GU188262	*Ceratostoma rorifluum*	correct	correct	correct	correct	correct	correct	*Ocinebrellus inornatus*
Ceratostoma rorifluum	HM180413	*Ceratostoma rorifluum*	correct	correct	correct	correct	correct	correct	*Ocinebrellus inornatus*
Ceratostoma rorifluum	HM180494	*Ceratostoma rorifluum*	correct	correct	correct	correct	correct	correct	*Ocinebrellus inornatus*
Cerithidea alata	HE680304	*Cerithidea alata*	correct	correct	correct	correct	correct	correct	*Cerithidea alata*
Cerithidea alata	HE680305	*Cerithidea alata*	correct	correct	correct	correct	correct	correct	*Cerithidea alata*
Cerithidea alata	HE680306	*Cerithidea alata*	correct	correct	correct	correct	correct	correct	*Cerithidea alata*
Cerithidea alata	HE680307	*Cerithidea alata*	correct	correct	correct	correct	correct	correct	*Cerithidea alata*
Cerithidea alata	HE680308	*Cerithidea alata*	correct	correct	correct	correct	correct	correct	*Cerithidea alata*
Cerithidea alata	HE680309	*Cerithidea alata*	correct	correct	correct	correct	correct	correct	*Cerithidea alata*
Cerithidea alata	HE680310	*Cerithidea alata*	correct	correct	correct	correct	correct	correct	*Cerithidea alata*
Cerithidea alata	HE680311	*Cerithidea alata*	correct	correct	correct	correct	correct	correct	*Cerithidea alata*

Cerithidea cingulata	HE680432	*Cerithidea cingulata*	no id	no id	no id	no id	no id	no id	*Cerithidea cingulata*
Cerithidea cingulata	HE680433	*Cerithidea cingulata*	correct	correct	correct	correct	correct	correct	*Cerithidea cingulata*
Cerithidea cingulata	HE680434	*Cerithidea cingulata*	correct	correct	correct	correct	correct	correct	*Cerithidea cingulata*
Cerithidea cingulata	HE680435	*Cerithidea cingulata*	correct	correct	correct	correct	correct	correct	*Cerithidea cingulata*
Cerithidea cingulata	HE680436	*Cerithidea cingulata*	correct	correct	correct	correct	correct	correct	*Cerithidea cingulata*
Cerithidea cingulata	HE680437	*Cerithidea cingulata*	correct	correct	correct	correct	correct	correct	*Cerithidea cingulata*
Cerithidea cingulata	HE680438	*Cerithidea cingulata*	correct	correct	correct	correct	correct	correct	*Cerithidea cingulata*
Cerithidea cingulata	HE680439	*Cerithidea cingulata*	correct	correct	correct	correct	correct	correct	*Cerithidea cingulata*
Cerithidea cingulata	HE680440	*Cerithidea cingulata*	correct	correct	correct	correct	correct	correct	*Cerithidea cingulata*
Cerithidea cingulata	HE680441	*Cerithidea cingulata*	correct	correct	correct	correct	correct	correct	*Cerithidea cingulata*
Cerithidea cingulata	HE680442	*Cerithidea cingulata*	correct	correct	correct	correct	correct	correct	*Cerithidea cingulata*
Cerithidea cingulata	HE680443	*Cerithidea cingulata*	correct	correct	correct	correct	correct	correct	*Cerithidea cingulata*
Cerithidea cingulata	HE680444	*Cerithidea cingulata*	correct	correct	correct	correct	correct	correct	*Cerithidea cingulata*
Cerithidea cingulata	HE680445	*Cerithidea cingulata*	correct	correct	correct	correct	correct	correct	*Cerithidea cingulata*
Cerithidea cingulata	HE680446	*Cerithidea cingulata*	correct	correct	correct	correct	correct	correct	*Cerithidea cingulata*
Cerithidea cingulata	HE680447	*Cerithidea cingulata*	correct	correct	correct	correct	correct	correct	*Cerithidea cingulata*
Cerithidea cingulata	HE680448	*Cerithidea cingulata*	correct	correct	correct	correct	correct	correct	*Cerithidea cingulata*
Cerithidea cingulata	HE680449	*Cerithidea cingulata*	correct	correct	correct	correct	correct	correct	*Cerithidea cingulata*
Cerithidea cingulata	HE680450	*Cerithidea cingulata*	correct	correct	correct	correct	correct	correct	*Cerithidea cingulata*
Cerithidea cingulata	HE680451	*Cerithidea cingulata*	correct	correct	correct	correct	correct	correct	*Cerithidea cingulata*
Cerithidea cingulata	HE680452	*Cerithidea cingulata*	correct	correct	correct	correct	correct	correct	*Cerithidea cingulata*
Cerithidea cingulata	HE680454	*Cerithidea cingulata*	correct	correct	correct	correct	correct	correct	*Cerithidea cingulata*
Cerithidea cingulata	HE680455	*Cerithidea djadjariensis*	no id	ambiguous	ambiguous	no id	ambiguous	ambiguous	*Cerithidea cingulata*
Cerithidea djadjariensis	HE680556	*Cerithidea djadjariensis*	incorrect	incorrect	incorrect	incorrect	incorrect	incorrect	*Cerithidea cingulata*
Cerithidea djadjariensis	HE680559	*Cerithidea djadjariensis*	correct	correct	correct	correct	correct	correct	*Cerithidea djadjariensis*
Cerithidea djadjariensis	HE680560	*Cerithidea djadjariensis*	no id	correct	correct	no id	correct	correct	*Cerithidea djadjariensis*
Cerithidea djadjariensis	HE680561	*Cerithidea djadjariensis*	correct	correct	correct	correct	correct	correct	*Cerithidea djadjariensis*
Cerithidea djadjariensis	HE680562	*Cerithidea djadjariensis*	correct	correct	correct	correct	correct	correct	*Cerithidea djadjariensis*
Cerithidea djadjariensis	HE680563	*Cerithidea djadjariensis*	correct	correct	correct	correct	correct	correct	*Cerithidea djadjariensis*
Cerithidea djadjariensis	HE680564	*Cerithidea djadjariensis*	correct	correct	correct	correct	correct	correct	*Cerithidea djadjariensis*
Cerithidea djadjariensis	HE680565	*Cerithidea djadjariensis*	correct	correct	correct	correct	correct	correct	*Cerithidea djadjariensis*

Cerithidea djadjariensis	HE680566	*Cerithidea djadjariensis*	correct	correct	correct	correct	correct	correct	*Cerithidea djadjariensis*
Cerithidea djadjariensis	HE680567	*Cerithidea djadjariensis*	correct	correct	correct	correct	correct	correct	*Cerithidea djadjariensis*
Cerithidea djadjariensis	HE680568	*Cerithidea djadjariensis*	correct	correct	correct	correct	correct	correct	*Cerithidea djadjariensis*
Cerithidea djadjariensis	HE680569	*Cerithidea djadjariensis*	correct	correct	correct	correct	correct	correct	*Cerithidea djadjariensis*
Cerithidea djadjariensis	HE680557	*Cerithidea djadjariensis*	correct	correct	correct	correct	correct	correct	*Cerithidea djadjariensis*
Cerithidea djadjariensis	HE680558	*Cerithidea djadjariensis*	no id	no id	correct	no id	no id	correct	*Cerithidea djadjariensis*
Cerithidea djadjariensis	HE680582	*Cerithidea djadjariensis*	no id	correct	correct	no id	correct	correct	*Cerithidea djadjariensis*
Cerithidea djadjariensis	HE680583	*Cerithidea djadjariensis*	correct	correct	correct	correct	correct	correct	*Cerithidea djadjariensis*
Cerithidea djadjariensis	HE680570	*Cerithidea djadjariensis*	correct	correct	correct	correct	correct	correct	*Cerithidea djadjariensis*
Cerithidea djadjariensis	HE680571	*Cerithidea djadjariensis*	correct	correct	correct	correct	correct	correct	*Cerithidea djadjariensis*
Cerithidea djadjariensis	HE680584	*Cerithidea djadjariensis*	correct	correct	correct	correct	correct	correct	*Cerithidea djadjariensis*
Cerithidea djadjariensis	HE680572	*Cerithidea djadjariensis*	correct	correct	correct	correct	correct	correct	*Cerithidea djadjariensis*
Cerithidea djadjariensis	HE680573	*Cerithidea djadjariensis*	correct	correct	correct	correct	correct	correct	*Cerithidea djadjariensis*
Cerithidea djadjariensis	HE680574	*Cerithidea djadjariensis*	correct	correct	correct	correct	correct	correct	*Cerithidea djadjariensis*
Cerithidea djadjariensis	HE680575	*Cerithidea djadjariensis*	correct	correct	correct	correct	correct	correct	*Cerithidea djadjariensis*
Cerithidea djadjariensis	HE680576	*Cerithidea djadjariensis*	correct	correct	correct	correct	correct	correct	*Cerithidea djadjariensis*
Cerithidea djadjariensis	HE680577	*Cerithidea djadjariensis*	correct	correct	correct	correct	correct	correct	*Cerithidea djadjariensis*
Cerithidea djadjariensis	HE680578	*Cerithidea djadjariensis*	correct	correct	correct	correct	correct	correct	*Cerithidea djadjariensis*
Cerithidea djadjariensis	HE680579	*Cerithidea djadjariensis*	correct	correct	correct	correct	correct	correct	*Cerithidea djadjariensis*
Cerithidea djadjariensis	HE680580	*Cerithidea djadjariensis*	correct	correct	correct	correct	correct	correct	*Cerithidea djadjariensis*
Cerithidea djadjariensis	HE680581	*Cerithidea djadjariensis*	correct	correct	correct	correct	correct	correct	*Cerithidea djadjariensis*
Cerithidea djadjariensis	HE680553	*Cerithidea djadjariensis*	correct	correct	correct	correct	correct	correct	*Cerithidea djadjariensis*
Cerithidea djadjariensis	HE680554	*Cerithidea djadjariensis*	no id	no id	correct	no id	no id	correct	*Cerithidea djadjariensis*
Cerithidea largillierti	AM932789	*Nerita plicata*	no id	no id	correct	no id	no id	correct	*Cerithideopsis largillierti*
Cerithidea ornate	AM932767	*Cerithidea ornate*	incorrect	incorrect	incorrect	incorrect	ambiguous	ambiguous	*Cerithidea rhizophorarum*
Cerithidea ornate	AM932766	*Cerithidea ornate*	ambiguous	ambiguous	ambiguous	ambiguous	ambiguous	ambiguous	*Cerithidea rhizophorarum*
Cerithidea ornate	HE680222	*Cerithidea ornate*	ambiguous	ambiguous	ambiguous	ambiguous	ambiguous	ambiguous	*Cerithidea rhizophorarum*
Cerithidea ornate	HE680223	*Cerithidea ornate*	ambiguous	ambiguous	ambiguous	ambiguous	ambiguous	ambiguous	*Cerithidea rhizophorarum*
Cerithidea ornate	HE680224	*Cerithidea ornate*	incorrect	incorrect	incorrect	incorrect	incorrect	incorrect	*Cerithidea rhizophorarum*
Cerithidea rhizophorarum	JF694694	*Cerithidea rhizophorarum*	correct	correct	correct	correct	correct	correct	*Cerithidea ornate*
Cerithidea rhizophorarum	JF694695	*Cerithidea rhizophorarum*	correct	correct	correct	correct	correct	correct	*Cerithidea ornate*

Cerithidea rhizophorarum	AM932774	*Cerithidea rhizophorarum*	no id	no id	ambiguous	no id	no id	ambiguous	*Cerithidea ornate*
Cerithidea rhizophorarum	HE680254	*Cerithidea rhizophorarum*	correct	correct	correct	correct	correct	correct	*Cerithidea ornate*
Cerithidea rhizophorarum	HE680255	*Cerithidea rhizophorarum*	correct	correct	correct	correct	correct	correct	*Cerithidea ornate*
Cerithidea rhizophorarum	HE680257	*Cerithidea rhizophorarum*	correct	correct	correct	correct	correct	correct	*Cerithidea ornate*
Cerithidea rhizophorarum	HE680258	*Cerithidea rhizophorarum*	correct	correct	correct	correct	correct	correct	*Cerithidea ornate*
Cerithidea rhizophorarum	HE680259	*Cerithidea rhizophorarum*	correct	correct	correct	correct	correct	correct	*Cerithidea ornate*
Cerithidea rhizophorarum	HE680260	*Cerithidea rhizophorarum*	correct	correct	correct	correct	correct	correct	*Cerithidea ornate*
Cerithidea rhizophorarum	HE680261	*Cerithidea rhizophorarum*	correct	correct	correct	correct	correct	correct	*Cerithidea ornate*
Cerithidea rhizophorarum	HE680262	*Cerithidea rhizophorarum*	no id	correct	correct	no id	correct	correct	*Cerithidea ornate*
Cerithidea rhizophorarum	HE680263	*Cerithidea rhizophorarum*	correct	correct	correct	correct	correct	correct	*Cerithidea ornate*
Cerithidea rhizophorarum	HE680264	*Cerithidea rhizophorarum*	correct	correct	correct	correct	correct	correct	*Cerithidea ornate*
Cerithidea rhizophorarum	HE680265	*Cerithidea rhizophorarum*	correct	correct	correct	correct	correct	correct	*Cerithidea ornate*
Cerithidea rhizophorarum	HE680276	*Cerithidea rhizophorarum*	correct	correct	correct	correct	correct	correct	*Cerithidea ornate*
Cerithidea rhizophorarum	HE680277	*Cerithidea rhizophorarum*	correct	correct	correct	correct	correct	correct	*Cerithidea ornate*
Cerithidea rhizophorarum	HE680278	*Cerithidea rhizophorarum*	correct	correct	correct	correct	correct	correct	*Cerithidea ornate*
Cerithidea rhizophorarum	HE680279	*Cerithidea rhizophorarum*	correct	correct	correct	correct	correct	correct	*Cerithidea ornate*
Cerithidea rhizophorarum	HE680280	*Cerithidea rhizophorarum*	correct	correct	correct	correct	correct	correct	*Cerithidea ornate*
Cerithidea rhizophorarum	HE680281	*Cerithidea rhizophorarum*	correct	correct	correct	correct	correct	correct	*Cerithidea ornate*
Cerithidea rhizophorarum	HE680282	*Cerithidea rhizophorarum*	correct	correct	correct	correct	correct	correct	*Cerithidea ornate*
Cerithidea rhizophorarum	HE680271	*Cerithidea rhizophorarum*	correct	correct	correct	correct	correct	correct	*Cerithidea ornate*
Cerithidea rhizophorarum	HE680272	*Cerithidea rhizophorarum*	correct	correct	correct	correct	correct	correct	*Cerithidea ornate*
Cerithidea rhizophorarum	HE680273	*Cerithidea rhizophorarum*	correct	correct	correct	correct	correct	correct	*Cerithidea ornate*
Cerithidea rhizophorarum	HE680274	*Cerithidea rhizophorarum*	correct	correct	correct	correct	correct	correct	*Cerithidea ornate*
Cerithidea rhizophorarum	HE680275	*Cerithidea rhizophorarum*	correct	correct	correct	correct	correct	correct	*Cerithidea ornate*
Cerithidea rhizophorarum	HE680270	*Cerithidea rhizophorarum*	correct	correct	correct	correct	correct	correct	*Cerithidea ornate*
Cerithidea rhizophorarum	HE680266	*Cerithidea rhizophorarum*	correct	correct	correct	correct	correct	correct	*Cerithidea ornate*
Cerithidea rhizophorarum	HE680267	*Cerithidea rhizophorarum*	correct	correct	correct	correct	correct	correct	*Cerithidea ornate*
Cerithidea rhizophorarum	HE680268	*Cerithidea rhizophorarum*	correct	correct	correct	correct	correct	correct	*Cerithidea ornate*
Cerithidea rhizophorarum	HE680269	*Cerithidea rhizophorarum*	correct	correct	correct	correct	correct	correct	*Cerithidea ornate*
Cerithidea rhizophorarum	HE680283	*Cerithidea rhizophorarum*	correct	correct	correct	correct	correct	correct	*Cerithidea ornate*
Cerithidea rhizophorarum	HE680284	*Cerithidea rhizophorarum*	correct	correct	correct	correct	correct	correct	*Cerithidea ornate*

Cerithidea rhizophorarum	HE680285	*Cerithidea rhizophorarum*	correct	correct	correct	correct	correct	correct	*Cerithidea ornate*
Chicoreus asianus	GU188200	*Chicoreus asianus*	correct	correct	correct	correct	correct	correct	*Hexaplex chicoreus*
Chicoreus asianus	GU188201	*Chicoreus asianus*	correct	correct	correct	correct	correct	correct	*Hexaplex chicoreus*
Chicoreus asianus	GU188202	*Chicoreus asianus*	correct	correct	correct	correct	correct	correct	*Hexaplex chicoreus*
Chicoreus asianus	GU188203	*Chicoreus asianus*	correct	correct	correct	correct	correct	correct	*Hexaplex chicoreus*
Chicoreus asianus	GU188204	*Chicoreus asianus*	correct	correct	correct	correct	correct	correct	*Hexaplex chicoreus*
Chicoreus asianus	GU188205	*Chicoreus asianus*	correct	correct	correct	correct	correct	correct	*Hexaplex chicoreus*
Chicoreus torrefactus	GU188208	*Chicoreus torrefactus*	correct	correct	correct	correct	correct	correct	*Chicoreus brunneus*
Chicoreus torrefactus	GU188209	*Chicoreus torrefactus*	correct	correct	correct	correct	correct	correct	*Chicoreus brunneus*
Chicoreus torrefactus	GU188210	*Chicoreus torrefactus*	correct	correct	correct	correct	correct	correct	*Chicoreus brunneus*
Chicoreus torrefactus	GU188211	*Chicoreus torrefactus*	correct	correct	correct	correct	correct	correct	*Chicoreus brunneus*
Chlamys farreri	GU119998	*Chlamys farreri*	correct	correct	correct	correct	correct	correct	
Chlamys farreri	GU119999	*Chlamys farreri*	correct	correct	correct	correct	correct	correct	
Chlamys farreri	GU120000	*Chlamys farreri*	correct	correct	correct	correct	correct	correct	
Chlamys farreri	FJ595957	*Chlamys farreri*	correct	correct	correct	correct	correct	correct	*Laevichlamys squamosa*
Chlorostoma lischkei	EU530145	*Chlorostoma lischkei*	correct	correct	correct	correct	correct	correct	*Chlorostoma lischkei*
Chlorostoma lischkei	EU530144	*Chlorostoma lischkei*	correct	correct	correct	correct	correct	correct	*Chlorostoma lischkei*
Omphalius rusticus	HM180735	*Chlorostoma turbinatum*	incorrect	incorrect	incorrect	incorrect	incorrect	incorrect	*Omphalius pfeifferi carpenteri*
Omphalius rusticus rusticus	HM180733	*Chlorostoma turbinatum*	no id	ambiguous	ambiguous	no id	ambiguous	ambiguous	*Omphalius pfeifferi carpenteri*
Omphalius rusticus rusticus	HM180734	*Chlorostoma turbinatum*	incorrect	incorrect	incorrect	incorrect	ambiguous	ambiguous	*Omphalius pfeifferi carpenteri*
Chlorostoma turbinatum	HM180511	*Chlorostoma turbinatum*	ambiguous	ambiguous	ambiguous	ambiguous	ambiguous	ambiguous	*Omphalius pfeifferi carpenteri*
Chlorostoma turbinatum	HM180512	*Chlorostoma turbinatum*	ambiguous	ambiguous	ambiguous	ambiguous	ambiguous	ambiguous	*Omphalius pfeifferi carpenteri*
Chlorostoma turbinatum	HM180513	*Chlorostoma turbinatum*	ambiguous	ambiguous	ambiguous	ambiguous	ambiguous	ambiguous	*Omphalius pfeifferi carpenteri*
Chlorostoma turbinatum	HM180514	*Chlorostoma turbinatum*	ambiguous	ambiguous	ambiguous	ambiguous	ambiguous	ambiguous	*Omphalius pfeifferi carpenteri*
Chlorostoma turbinatum	HM180515	*Chlorostoma turbinatum*	ambiguous	ambiguous	ambiguous	ambiguous	ambiguous	ambiguous	*Omphalius pfeifferi carpenteri*
Chlorostoma turbinatum	HM180516	*Chlorostoma turbinatum*	ambiguous	ambiguous	ambiguous	ambiguous	ambiguous	ambiguous	*Omphalius pfeifferi carpenteri*
Chlorostoma turbinatum	HM180517	*Chlorostoma turbinatum*	ambiguous	ambiguous	ambiguous	ambiguous	ambiguous	ambiguous	*Omphalius pfeifferi carpenteri*
Chlorostoma turbinatum	HM180518	*Chlorostoma turbinatum*	ambiguous	ambiguous	ambiguous	ambiguous	ambiguous	ambiguous	*Omphalius pfeifferi carpenteri*
Chlorostoma turbinatum	HM180519	*Chlorostoma turbinatum*	ambiguous	ambiguous	ambiguous	ambiguous	ambiguous	ambiguous	*Omphalius pfeifferi carpenteri*
Chlorostoma turbinatum	HM180520	*Chlorostoma turbinatum*	ambiguous	ambiguous	ambiguous	ambiguous	ambiguous	ambiguous	*Omphalius pfeifferi carpenteri*
Chlorostoma turbinatum	HM180521	*Chlorostoma turbinatum*	ambiguous	ambiguous	ambiguous	ambiguous	ambiguous	ambiguous	*Omphalius pfeifferi carpenteri*

Chlorostoma turbinatum	HM180523	*Chlorostoma turbinatum*	no id	no id	no id	no id	no id	no id	*Omphalius pfeifferi carpenteri*
Chlorostoma turbinatum	HM180524	*Chlorostoma turbinatum*	ambiguous	ambiguous	ambiguous	ambiguous	ambiguous	ambiguous	*Omphalius pfeifferi carpenteri*
Chlorostoma turbinatum	HM180525	*Chlorostoma turbinatum*	ambiguous	ambiguous	ambiguous	ambiguous	ambiguous	ambiguous	*Omphalius pfeifferi carpenteri*
Chlorostoma turbinatum	HM180526	*Chlorostoma turbinatum*	ambiguous	ambiguous	ambiguous	ambiguous	ambiguous	ambiguous	*Omphalius pfeifferi carpenteri*
Chlorostoma turbinatum	HM180522	*Chlorostoma turbinatum*	ambiguous	ambiguous	ambiguous	ambiguous	ambiguous	ambiguous	*Chlorostoma turbinatum*
Circe scripta	HQ703107	*Circe scripta*	correct	correct	correct	correct	correct	correct	*Circe scripta*
Circe scripta	HQ703108	*Circe scripta*	correct	correct	correct	correct	correct	correct	*Circe scripta*
Circe scripta	HQ703109	*Circe scripta*	correct	correct	correct	correct	correct	correct	*Circe scripta*
Circe scripta	HQ703110	*Circe scripta*	correct	correct	correct	correct	correct	correct	*Circe scripta*
Circe scripta	HQ703111	*Circe scripta*	correct	correct	correct	correct	correct	correct	*Circe scripta*
Circe scripta	HQ703112	*Circe scripta*	correct	correct	correct	correct	correct	correct	*Circe scripta*
Circe scripta	HQ703113	*Circe scripta*	correct	correct	correct	correct	correct	correct	*Circe scripta*
Circe scripta	HQ703114	*Circe scripta*	correct	correct	correct	correct	correct	correct	*Circe scripta*
Cistopus indicus	JX456269	*Cistopus taiwanicus*	incorrect	incorrect	incorrect	incorrect	incorrect	incorrect	*Cistopus indicus*
Cistopus taiwanicus	HQ846142	*Cistopus indicus*	ambiguous	ambiguous	ambiguous	ambiguous	ambiguous	ambiguous	*Cistopus indicus*
Cistopus taiwanicus	HQ846143	*Cistopus indicus*	ambiguous	ambiguous	ambiguous	ambiguous	ambiguous	ambiguous	*Cistopus indicus*
Clanculus bronni	AB505296	*Trochus maculatus*	no id	no id	no id	no id	no id	no id	*Bathymophila sp.*
Clanculus margaritarius	AB505297	*Trochus maculatus*	no id	no id	no id	no id	no id	no id	*Trochidae sp.*
Clanculus microdon	AB505298	*Pseudostomatella decolorata*	no id	no id	no id	no id	no id	no id	*Trochidae sp.*
Clinocardium californiense	JN860022	*Clinocardium californiense*	correct	correct	correct	correct	correct	correct	*Keenocardium blandum*
Clinocardium californiense	JN860023	*Clinocardium californiense*	correct	correct	correct	correct	correct	correct	*Keenocardium blandum*
Clinocardium californiense	JN860024	*Clinocardium californiense*	correct	correct	correct	correct	correct	correct	*Keenocardium blandum*
Clinocardium californiense	JN860025	*Clinocardium californiense*	correct	correct	correct	correct	correct	correct	*Keenocardium blandum*
Clinocardium californiense	JN860026	*Clinocardium californiense*	correct	correct	correct	correct	correct	correct	*Keenocardium blandum*
Clinocardium californiense	JN860027	*Clinocardium californiense*	correct	correct	correct	correct	correct	correct	*Keenocardium blandum*
Clypeomorus humilis	JF693365	*Clypeomorus humilis*	no id	no id	no id	no id	no id	no id	*Batillaria cumingii*
Clypeomorus humilis	JF693366	*Clypeomorus humilis*	incorrect	incorrect	incorrect	incorrect	incorrect	incorrect	*Planaxis sulcatus*
Planaxis sulcatus	JF693413	*Planaxis sulcatus*	no id	ambiguous	ambiguous	no id	ambiguous	ambiguous	*Planaxis sulcatus*
Planaxis sulcatus	JF693411	*Planaxis sulcatus*	ambiguous	ambiguous	ambiguous	ambiguous	ambiguous	ambiguous	*Planaxis sulcatus*
Planaxis sulcatus	JF693414	*Planaxis sulcatus*	ambiguous	ambiguous	ambiguous	ambiguous	ambiguous	ambiguous	*Planaxis sulcatus*
Planaxis sulcatus	JF693412	*Planaxis sulcatus*	ambiguous	ambiguous	ambiguous	ambiguous	ambiguous	ambiguous	*Planaxis sulcatus*

Planaxis sulcatus	JF693415	*Planaxis sulcatus*	ambiguous	ambiguous	ambiguous	ambiguous	ambiguous	ambiguous	*Planaxis sulcatus*
Clypeomorus trailli	JF693363	*Clypeomorus trailli*	correct	correct	correct	correct	correct	correct	*Clypeomorus brevis*
Clypeomorus trailli	JF693364	*Clypeomorus trailli*	correct	correct	correct	correct	correct	correct	*Clypeomorus brevis*
Coccopigya punctoradiata	AB238590	*Coccopigya punctoradiata*	correct	correct	correct	correct	correct	correct	*Cocculina sp.*
Coccopigya punctoradiata	AB365259	*Coccopigya punctoradiata*	correct	correct	correct	correct	correct	correct	*Cocculina sp.*
Coelomactra antiquata	JN674607	*Coelomactra antiquata*	no id	correct	correct	no id	correct	correct	*Mactra cumingii*
Coelomactra antiquata	JN674608	*Coelomactra antiquata*	no id	correct	correct	no id	correct	correct	*Mactra cumingii*
Coelomactra antiquata	JN674609	*Coelomactra antiquata*	no id	no id	no id	no id	no id	no id	*Mactra cumingii*
Mactra cumingii	JN674610	*Coelomactra cumingii*	correct	correct	correct	correct	correct	correct	*Coelomactra antiquata*
Mactra cumingii	JN674611	*Coelomactra cumingii*	correct	correct	correct	correct	correct	correct	*Coelomactra antiquata*
Mactra cumingii	JN674612	*Coelomactra cumingii*	correct	correct	correct	correct	correct	correct	*Coelomactra antiquata*
Collonista amakusaensis	AM049345	*Collonista costulosa*	no id	no id	no id	no id	no id	no id	*Collonista costulosa*
Collonista costulosa	AM049346	*Collonista amakusaensis*	no id	no id	no id	no id	no id	no id	*Collonista amakusaensis*
Conotalopia mustelina	AB505302	*Trochus histrio*	no id	no id	no id	no id	no id	no id	*Lirularia iridescens*
Conotalopia ornata	AB505303	*Littoraria sinensis*	no id	no id	no id	no id	no id	no id	*Lirularia succincta*
Conus betulinus	HQ834088	*Conus betulinus*	correct	correct	correct	correct	correct	correct	*Conus loroisii*
Conus betulinus	JN053043	*Conus betulinus*	correct	correct	correct	correct	correct	correct	*Conus loroisii*
Conus ebraeus	EF547576	*Conus ebraeus*	correct	correct	correct	correct	correct	correct	*Conus chaldaeus*
Conus ebraeus	EF547575	*Conus ebraeus*	correct	correct	correct	correct	correct	correct	*Conus chaldaeus*
Conus ebraeus	EF547574	*Conus ebraeus*	correct	correct	correct	correct	correct	correct	*Conus chaldaeus*
Conus ebraeus	EF547573	*Conus ebraeus*	correct	correct	correct	correct	correct	correct	*Conus chaldaeus*
Conus ebraeus	EF547572	*Conus ebraeus*	correct	correct	correct	correct	correct	correct	*Conus chaldaeus*
Conus ebraeus	EF547571	*Conus ebraeus*	no id	correct	correct	no id	correct	correct	*Conus chaldaeus*
Conus ebraeus	EF547570	*Conus ebraeus*	correct	correct	correct	correct	correct	correct	*Conus chaldaeus*
Conus ebraeus	EF547569	*Conus ebraeus*	correct	correct	correct	correct	correct	correct	*Conus chaldaeus*
Conus ebraeus	EF547568	*Conus ebraeus*	correct	correct	correct	correct	correct	correct	*Conus chaldaeus*
Conus ebraeus	EF547567	*Conus ebraeus*	correct	correct	correct	correct	correct	correct	*Conus chaldaeus*
Conus ebraeus	EF547566	*Conus ebraeus*	correct	correct	correct	correct	correct	correct	*Conus chaldaeus*
Conus ebraeus	EF547565	*Conus ebraeus*	correct	correct	correct	correct	correct	correct	*Conus chaldaeus*
Conus ebraeus	EF547564	*Conus ebraeus*	correct	correct	correct	correct	correct	correct	*Conus chaldaeus*
Conus ebraeus	EF547563	*Conus ebraeus*	correct	correct	correct	correct	correct	correct	*Conus chaldaeus*

Conus ebraeus	EF547562	*Conus ebraeus*	correct	correct	correct	correct	correct	correct	*Conus chaldaeus*
Conus ebraeus	EF547561	*Conus ebraeus*	correct	correct	correct	correct	correct	correct	*Conus chaldaeus*
Conus ebraeus	EF547560	*Conus ebraeus*	correct	correct	correct	correct	correct	correct	*Conus chaldaeus*
Conus ebraeus	EF547559	*Conus ebraeus*	correct	correct	correct	correct	correct	correct	*Conus chaldaeus*
Conus judaeus	EF108266	*Conus ebraeus*	no id	no id	no id	no id	no id	no id	*Conus coronatus*
Conus quercinus	HQ834087	*Conus lividus*	no id	no id	no id	no id	no id	no id	*Conus sp.*
Conus lividus	HQ852576	*Conus lividus*	ambiguous	ambiguous	ambiguous	ambiguous	ambiguous	ambiguous	*Conus sp.*
Conus lividus	HQ852577	*Conus lividus*	ambiguous	ambiguous	ambiguous	ambiguous	ambiguous	ambiguous	*Conus sp.*
Conus lividus	HQ852575	*Conus lividus*	no id	ambiguous	ambiguous	no id	ambiguous	ambiguous	*Conus sp.*
Conus sanguinolentus	HQ834090	*Conus lividus*	incorrect	incorrect	incorrect	incorrect	incorrect	incorrect	*Conus sp.*
Conus sanguinolentus	HQ852532	*Conus sanguinolentus*	correct	correct	correct	correct	correct	correct	*Conus diadema*
Conus sanguinolentus	HQ852530	*Conus sanguinolentus*	correct	correct	correct	correct	correct	correct	*Conus diadema*
Conus sanguinolentus	HQ852528	*Conus sanguinolentus*	correct	correct	correct	correct	correct	correct	*Conus diadema*
Conus sanguinolentus	HQ852526	*Conus sanguinolentus*	correct	correct	correct	correct	correct	correct	*Conus diadema*
Conus sanguinolentus	HQ852524	*Conus sanguinolentus*	correct	correct	correct	correct	correct	correct	*Conus diadema*
Conus sanguinolentus	HQ852522	*Conus sanguinolentus*	correct	correct	correct	correct	correct	correct	*Conus diadema*
Conus sanguinolentus	HQ852520	*Conus sanguinolentus*	correct	correct	correct	correct	correct	correct	*Conus diadema*
Conus sanguinolentus	HQ852533	*Conus sanguinolentus*	correct	correct	correct	correct	correct	correct	*Conus diadema*
Conus sanguinolentus	HQ852529	*Conus sanguinolentus*	correct	correct	correct	correct	correct	correct	*Conus diadema*
Conus sanguinolentus	HQ852531	*Conus sanguinolentus*	correct	correct	correct	correct	correct	correct	*Conus diadema*
Conus sanguinolentus	HQ852527	*Conus sanguinolentus*	correct	correct	correct	correct	correct	correct	*Conus diadema*
Conus sanguinolentus	HQ852525	*Conus sanguinolentus*	correct	correct	correct	correct	correct	correct	*Conus diadema*
Conus sanguinolentus	HQ852523	*Conus sanguinolentus*	correct	correct	correct	correct	correct	correct	*Conus diadema*
Conus sanguinolentus	HQ852521	*Conus sanguinolentus*	correct	correct	correct	correct	correct	correct	*Conus diadema*
Conus textile	HQ834089	*Euplica scripta*	no id	no id	no id	no id	no id	no id	*Conus textile archiepiscopus*
Corbicula fluminea	KC211267	*Corbicula fluminea*	ambiguous	ambiguous	ambiguous	ambiguous	ambiguous	ambiguous	*Corbicula sp.*
Corbicula fluminea	KC211281	*Corbicula fluminea*	ambiguous	ambiguous	ambiguous	ambiguous	ambiguous	ambiguous	*Corbicula sp.*
Corbicula fluminea	KC211282	*Corbicula fluminea*	ambiguous	ambiguous	ambiguous	ambiguous	ambiguous	ambiguous	*Corbicula sp.*
Corbicula fluminea	KC211283	*Corbicula fluminea*	ambiguous	ambiguous	ambiguous	ambiguous	ambiguous	ambiguous	*Corbicula sp.*
Corbicula fluminea	KC211285	*Corbicula fluminea*	ambiguous	ambiguous	ambiguous	ambiguous	ambiguous	ambiguous	*Corbicula sp.*
Corbicula fluminea	KC211286	*Corbicula fluminea*	ambiguous	ambiguous	ambiguous	ambiguous	ambiguous	ambiguous	*Corbicula sp.*

Corbicula leana	KC211268	*Corbicula fluminea*	ambiguous	ambiguous	ambiguous	ambiguous	ambiguous	ambiguous	*Corbicula sp.*
Corbicula leana	KC211269	*Corbicula fluminea*	ambiguous	ambiguous	ambiguous	ambiguous	ambiguous	ambiguous	*Corbicula sp.*
Corbicula leana	KC211270	*Corbicula fluminea*	ambiguous	ambiguous	ambiguous	ambiguous	ambiguous	ambiguous	*Corbicula sp.*
Corbicula leana	AB498810	*Corbicula fluminea*	ambiguous	ambiguous	ambiguous	ambiguous	ambiguous	ambiguous	*Corbicula sp.*
Corbicula leana	AB845591	*Corbicula fluminea*	ambiguous	ambiguous	ambiguous	ambiguous	ambiguous	ambiguous	*Corbicula sp.*
Corbicula leana	KC211287	*Corbicula leana*	correct	correct	correct	correct	ambiguous	ambiguous	*Corbicula sp.*
Corbicula leana	KC211288	*Corbicula leana*	correct	correct	correct	correct	ambiguous	ambiguous	*Corbicula sp.*
Corbicula leana	KC211289	*Corbicula leana*	correct	correct	correct	correct	ambiguous	ambiguous	*Corbicula sp.*
Corbicula japonica	AB498808	*Corbicula japonica*	correct	correct	correct	correct	correct	correct	*Corbicula leana*
Corbicula japonica	AB498018	*Corbicula japonica*	correct	correct	correct	correct	correct	correct	*Corbicula leana*
Corbicula japonica	AB845593	*Corbicula japonica*	correct	correct	correct	correct	correct	correct	*Corbicula leana*
Corbicula japonica	AB971384	*Corbicula japonica*	correct	correct	correct	correct	correct	correct	*Corbicula leana*
Corbicula japonica	AB971385	*Corbicula japonica*	correct	correct	correct	correct	correct	correct	*Corbicula leana*
Corbicula japonica	AB971386	*Corbicula japonica*	correct	correct	correct	correct	correct	correct	*Corbicula leana*
Corbicula japonica	AB971387	*Corbicula japonica*	correct	correct	correct	correct	correct	correct	*Corbicula leana*
Corbicula japonica	AB971388	*Corbicula japonica*	correct	correct	correct	correct	correct	correct	*Corbicula leana*
Corbicula japonica	AB971389	*Corbicula japonica*	correct	correct	correct	correct	correct	correct	*Corbicula leana*
Corbicula japonica	AB971390	*Corbicula japonica*	correct	correct	correct	correct	correct	correct	*Corbicula leana*
Corbicula japonica	AB971391	*Corbicula japonica*	correct	correct	correct	correct	correct	correct	*Corbicula leana*
Corbicula japonica	AB971392	*Corbicula japonica*	correct	correct	correct	correct	correct	correct	*Corbicula leana*
Corbicula japonica	AB971393	*Corbicula japonica*	correct	correct	correct	correct	correct	correct	*Corbicula leana*
Corbicula japonica	AB971394	*Corbicula japonica*	correct	correct	correct	correct	correct	correct	*Corbicula leana*
Corbicula japonica	AB971395	*Corbicula japonica*	correct	correct	correct	correct	correct	correct	*Corbicula leana*
Corbicula japonica	AB971396	*Corbicula japonica*	no id	correct	correct	no id	correct	correct	*Corbicula leana*
Corbicula japonica	AB971397	*Corbicula japonica*	correct	correct	correct	correct	correct	correct	*Corbicula leana*
Corbicula japonica	AB971398	*Corbicula japonica*	correct	correct	correct	correct	correct	correct	*Corbicula leana*
Corbicula japonica	AB971399	*Corbicula japonica*	correct	correct	correct	correct	correct	correct	*Corbicula leana*
Corbicula japonica	AB971400	*Corbicula japonica*	correct	correct	correct	correct	correct	correct	*Corbicula leana*
Corbicula japonica	AB971401	*Corbicula japonica*	correct	correct	correct	correct	correct	correct	*Corbicula leana*
Corbicula japonica	AB971402	*Corbicula japonica*	correct	correct	correct	correct	correct	correct	*Corbicula leana*
Corbicula japonica	AB971403	*Corbicula japonica*	correct	correct	correct	correct	correct	correct	*Corbicula leana*

Corbicula japonica	AB971404	*Corbicula japonica*	correct	correct	correct	correct	correct	correct	*Corbicula leana*
Corbicula japonica	AB971405	*Corbicula japonica*	correct	correct	correct	correct	correct	correct	*Corbicula leana*
Corbicula japonica	AB971406	*Corbicula japonica*	correct	correct	correct	correct	correct	correct	*Corbicula leana*
Corbicula japonica	AB971407	*Corbicula japonica*	correct	correct	correct	correct	correct	correct	*Corbicula leana*
Corbicula japonica	AB971408	*Corbicula japonica*	no id	correct	correct	no id	correct	correct	*Corbicula leana*
Corbicula japonica	KC211253	*Corbicula japonica*	correct	correct	correct	correct	correct	correct	*Corbicula leana*
Corbicula japonica	KC211252	*Corbicula japonica*	correct	correct	correct	correct	correct	correct	*Corbicula leana*
Corbicula japonica	KC211254	*Corbicula japonica*	correct	correct	correct	correct	correct	correct	*Corbicula leana*
Corbicula japonica	KC211255	*Corbicula japonica*	correct	correct	correct	correct	correct	correct	*Corbicula leana*
Corbicula japonica	KC211256	*Corbicula japonica*	no id	correct	correct	no id	correct	correct	*Corbicula leana*
Corbicula japonica	KC211257	*Corbicula japonica*	correct	correct	correct	correct	correct	correct	*Corbicula leana*
Corbicula japonica	KC211258	*Corbicula japonica*	correct	correct	correct	correct	correct	correct	*Corbicula leana*
Corbicula japonica	KC211259	*Corbicula japonica*	correct	correct	correct	correct	correct	correct	*Corbicula leana*
Corbicula japonica	KC211271	*Corbicula japonica*	correct	correct	correct	correct	correct	correct	*Corbicula leana*
Corbicula japonica	KC211272	*Corbicula japonica*	correct	correct	correct	correct	correct	correct	*Corbicula leana*
Corbicula japonica	KC211273	*Corbicula japonica*	correct	correct	correct	correct	correct	correct	*Corbicula leana*
Corbicula japonica	KC211274	*Corbicula japonica*	correct	correct	correct	correct	correct	correct	*Corbicula leana*
Corbicula japonica	KC211275	*Corbicula japonica*	correct	correct	correct	correct	correct	correct	*Corbicula leana*
Corbicula japonica	KC211276	*Corbicula japonica*	correct	correct	correct	correct	correct	correct	*Corbicula leana*
Corbicula sandai	AB845590	*Corbicula sandai*	correct	correct	correct	correct	correct	correct	*Corbicula fluminea*
Corbicula sandai	KC211277	*Corbicula sandai*	correct	correct	correct	correct	correct	correct	*Corbicula fluminea*
Corbicula sandai	KC211278	*Corbicula sandai*	correct	correct	correct	correct	correct	correct	*Corbicula fluminea*
Corbicula sandai	KC211279	*Corbicula sandai*	correct	correct	correct	correct	correct	correct	*Corbicula fluminea*
Corbicula sandai	KC211280	*Corbicula sandai*	correct	correct	correct	correct	correct	correct	*Corbicula fluminea*
Corbicula sandai	AB498811	*Corbicula sandai*	correct	correct	correct	correct	correct	correct	*Corbicula fluminea*
Corbula amurensis	KJ028746	*Corbula amurensis*	correct	correct	correct	correct	correct	correct	*Potamocorbula sp.*
Corbula amurensis	KJ028747	*Corbula amurensis*	correct	correct	correct	correct	correct	correct	*Potamocorbula sp.*
Corbula amurensis	KJ028748	*Corbula amurensis*	correct	correct	correct	correct	correct	correct	*Potamocorbula sp.*
Corbula amurensis	KJ028749	*Corbula amurensis*	correct	correct	correct	correct	correct	correct	*Potamocorbula sp.*
Corbula amurensis	KJ028750	*Corbula amurensis*	correct	correct	correct	correct	correct	correct	*Potamocorbula sp.*
Corbula amurensis	KJ028751	*Corbula amurensis*	correct	correct	correct	correct	correct	correct	*Potamocorbula sp.*

Corbula amurensis	KJ028752	*Corbula amurensis*	correct	correct	correct	correct	correct	correct	*Potamocorbula sp.*
Corbula amurensis	KJ028753	*Corbula amurensis*	correct	correct	correct	correct	correct	correct	*Potamocorbula sp.*
Corbula amurensis	KJ028754	*Corbula amurensis*	correct	correct	correct	correct	correct	correct	*Potamocorbula sp.*
Corbula amurensis	KJ028755	*Corbula amurensis*	correct	correct	correct	correct	correct	correct	*Potamocorbula sp.*
Corbula amurensis	KJ028756	*Corbula amurensis*	correct	correct	correct	correct	correct	correct	*Potamocorbula sp.*
Corbula amurensis	KJ028757	*Corbula amurensis*	correct	correct	correct	correct	correct	correct	*Potamocorbula sp.*
Corbula erythrodon	KJ125419	*Pholas orientalis*	correct	correct	correct	correct	correct	correct	*Corbula tunicata*
Costacallista erycina	HQ703031	*Costacallista erycina*	correct	correct	correct	correct	correct	correct	*Costacallista lilacina*
Costacallista erycina	HQ703032	*Costacallista erycina*	correct	correct	correct	correct	correct	correct	*Costacallista lilacina*
Costacallista erycina	HQ703033	*Costacallista erycina*	correct	correct	correct	correct	correct	correct	*Costacallista lilacina*
Costacallista erycina	HQ703034	*Costacallista erycina*	correct	correct	correct	correct	correct	correct	*Costacallista lilacina*
Costacallista erycina	JN898943	*Costacallista erycina*	correct	correct	correct	correct	correct	correct	*Costacallista lilacina*
Costacallista erycina	HM124570	*Costacallista erycina*	correct	correct	correct	correct	correct	correct	*Costacallista lilacina*
Costacallista erycina	EU117993	*Costacallista erycina*	correct	correct	correct	correct	correct	correct	*Costacallista lilacina*
Crassostrea angulata	HQ661008	*Crassostrea angulata*	correct	correct	correct	correct	correct	correct	*Crassostrea gigas*
Crassostrea angulata	HQ661009	*Crassostrea angulata*	correct	correct	correct	correct	correct	correct	*Crassostrea gigas*
Crassostrea angulata	EU672832	*Crassostrea angulata*	correct	correct	correct	correct	correct	correct	*Crassostrea gigas*
Crassostrea angulata	AB904879	*Crassostrea angulata*	correct	correct	correct	correct	correct	correct	*Crassostrea gigas*
Crassostrea angulata	AB904880	*Crassostrea angulata*	correct	correct	correct	correct	correct	correct	*Crassostrea gigas*
Crassostrea angulata	AB904881	*Crassostrea angulata*	correct	correct	correct	correct	correct	correct	*Crassostrea gigas*
Crassostrea angulata	AB904882	*Crassostrea angulata*	correct	correct	correct	correct	correct	correct	*Crassostrea gigas*
Crassostrea angulata	AB904883	*Crassostrea angulata*	correct	correct	correct	correct	correct	correct	*Crassostrea gigas*
Crassostrea angulata	AB904885	*Crassostrea angulata*	correct	correct	correct	correct	correct	correct	*Crassostrea gigas*
Crassostrea angulata	AB904886	*Crassostrea angulata*	correct	correct	correct	correct	correct	correct	*Crassostrea gigas*
Crassostrea angulata	AB904887	*Crassostrea angulata*	no id	correct	correct	no id	correct	correct	*Crassostrea gigas*
Crassostrea angulata	AB904888	*Crassostrea angulata*	no id	correct	correct	no id	correct	correct	*Crassostrea gigas*
Crassostrea angulata	AB904890	*Crassostrea angulata*	correct	correct	correct	correct	correct	correct	*Crassostrea gigas*
Crassostrea ariakensis	HQ661020	*Crassostrea ariakensis*	no id	correct	correct	no id	correct	correct	*Crassostrea belcheri*
Crassostrea ariakensis	HQ661021	*Crassostrea ariakensis*	correct	correct	correct	correct	correct	correct	*Crassostrea belcheri*
Crassostrea ariakensis	EU672835	*Crassostrea ariakensis*	correct	correct	correct	correct	correct	correct	*Crassostrea belcheri*
Crassostrea gigas	HQ661002	*Crassostrea gigas*	correct	correct	correct	correct	correct	correct	*Crassostrea angulata*

Crassostrea gigas	HQ661003	*Crassostrea gigas*	correct	correct	correct	correct	correct	correct	*Crassostrea angulata*
Crassostrea gigas	HQ661004	*Crassostrea gigas*	correct	correct	correct	correct	correct	correct	*Crassostrea angulata*
Crassostrea gigas	HQ661005	*Crassostrea gigas*	correct	correct	correct	correct	correct	correct	*Crassostrea angulata*
Crassostrea gigas	HQ661006	*Crassostrea gigas*	correct	correct	correct	correct	correct	correct	*Crassostrea angulata*
Crassostrea gigas	HQ661007	*Crassostrea gigas*	correct	correct	correct	correct	correct	correct	*Crassostrea angulata*
Crassostrea gigas	AB636176	*Crassostrea gigas*	no id	correct	correct	no id	correct	correct	*Crassostrea angulata*
Crassostrea gigas	AB636174	*Crassostrea gigas*	correct	correct	correct	correct	correct	correct	*Crassostrea angulata*
Crassostrea gigas	AB636172	*Crassostrea gigas*	correct	correct	correct	correct	correct	correct	*Crassostrea angulata*
Crassostrea gigas	AB636169	*Crassostrea gigas*	correct	correct	correct	correct	correct	correct	*Crassostrea angulata*
Crassostrea gigas	AB636184	*Crassostrea gigas*	correct	correct	correct	correct	correct	correct	*Crassostrea angulata*
Crassostrea gigas	AB636183	*Crassostrea gigas*	correct	correct	correct	correct	correct	correct	*Crassostrea angulata*
Crassostrea gigas	AB636182	*Crassostrea gigas*	correct	correct	correct	correct	correct	correct	*Crassostrea angulata*
Crassostrea gigas	AB636181	*Crassostrea gigas*	correct	correct	correct	correct	correct	correct	*Crassostrea angulata*
Crassostrea gigas	AB636178	*Crassostrea gigas*	no id	correct	correct	no id	correct	correct	*Crassostrea angulata*
Crassostrea gigas	AB636171	*Crassostrea gigas*	no id	correct	correct	no id	correct	correct	*Crassostrea angulata*
Crassostrea gigas	AB636167	*Crassostrea gigas*	correct	correct	correct	correct	correct	correct	*Crassostrea angulata*
Crassostrea gigas	AB636166	*Crassostrea gigas*	correct	correct	correct	correct	correct	correct	*Crassostrea angulata*
Crassostrea gigas	AB904884	*Crassostrea gigas*	correct	correct	correct	correct	correct	correct	*Crassostrea angulata*
Crassostrea gigas	AB904889	*Crassostrea gigas*	correct	correct	correct	correct	correct	correct	*Crassostrea angulata*
Crassostrea gigas	KJ855241	*Crassostrea gigas*	correct	correct	correct	correct	correct	correct	*Crassostrea angulata*
Crassostrea hongkongensis	FJ593172	*Crassostrea hongkongensis*	correct	correct	correct	correct	correct	correct	*Crassostrea nippona*
Crassostrea hongkongensis	EU118008	*Crassostrea hongkongensis*	correct	correct	correct	correct	correct	correct	*Crassostrea nippona*
Crassostrea hongkongensis	EU672834	*Crassostrea hongkongensis*	correct	correct	correct	correct	correct	correct	*Crassostrea nippona*
Crassostrea hongkongensis	FJ593173	*Crassostrea hongkongensis*	correct	correct	correct	correct	correct	correct	*Crassostrea nippona*
Crassostrea iredalei	HQ661022	*Crassostrea iredalei*	correct	correct	correct	correct	correct	correct	*Crassostrea sp.*
Crassostrea iredalei	HQ661023	*Crassostrea iredalei*	correct	correct	correct	correct	correct	correct	*Crassostrea sp.*
Crassostrea sikamea	HQ661010	*Crassostrea sikamea*	correct	correct	correct	correct	correct	correct	*Crassostrea angulata*
Crassostrea sikamea	HQ661011	*Crassostrea sikamea*	correct	correct	correct	correct	correct	correct	*Crassostrea angulata*
Crassostrea sikamea	HQ661012	*Crassostrea sikamea*	correct	correct	correct	correct	correct	correct	*Crassostrea angulata*
Crassostrea sikamea	HQ661013	*Crassostrea sikamea*	correct	correct	correct	correct	correct	correct	*Crassostrea angulata*
Crassostrea sikamea	HQ661014	*Crassostrea sikamea*	correct	correct	correct	correct	correct	correct	*Crassostrea angulata*

Crassostrea sikamea	HQ661015	*Crassostrea sikamea*	correct	correct	correct	correct	correct	correct	*Crassostrea angulata*
Crassostrea sikamea	HQ661016	*Crassostrea sikamea*	correct	correct	correct	correct	correct	correct	*Crassostrea angulata*
Crassostrea sikamea	HQ661017	*Crassostrea sikamea*	correct	correct	correct	correct	correct	correct	*Crassostrea angulata*
Crassostrea sikamea	HQ661018	*Crassostrea sikamea*	no id	correct	correct	no id	correct	correct	*Crassostrea angulata*
Crassostrea sikamea	HQ661019	*Crassostrea sikamea*	no id	correct	correct	no id	correct	correct	*Crassostrea angulata*
Crassostrea sikamea	AB904872	*Crassostrea sikamea*	correct	correct	correct	correct	correct	correct	*Crassostrea angulata*
Crassostrea sikamea	AB904873	*Crassostrea sikamea*	correct	correct	correct	correct	correct	correct	*Crassostrea angulata*
Crassostrea sikamea	AB904874	*Crassostrea sikamea*	correct	correct	correct	correct	correct	correct	*Crassostrea angulata*
Crassostrea sikamea	AB904875	*Crassostrea sikamea*	correct	correct	correct	correct	correct	correct	*Crassostrea angulata*
Crassostrea sikamea	AB904876	*Crassostrea sikamea*	correct	correct	correct	correct	correct	correct	*Crassostrea angulata*
Crassostrea sikamea	AB904877	*Crassostrea sikamea*	correct	correct	correct	correct	correct	correct	*Crassostrea angulata*
Crassostrea sikamea	AB904878	*Crassostrea sikamea*	correct	correct	correct	correct	correct	correct	*Crassostrea angulata*
Crassostrea sikamea	AB675954	*Crassostrea sikamea*	correct	correct	correct	correct	correct	correct	*Crassostrea angulata*
Crassostrea sikamea	AB675952	*Crassostrea sikamea*	correct	correct	correct	correct	correct	correct	*Crassostrea angulata*
Crassostrea sikamea	AB675950	*Crassostrea sikamea*	correct	correct	correct	correct	correct	correct	*Crassostrea angulata*
Crassostrea sikamea	AB675948	*Crassostrea sikamea*	correct	correct	correct	correct	correct	correct	*Crassostrea angulata*
Crassostrea sikamea	AB675955	*Crassostrea sikamea*	correct	correct	correct	correct	correct	correct	*Crassostrea angulata*
Crassostrea sikamea	AB675953	*Crassostrea sikamea*	correct	correct	correct	correct	correct	correct	*Crassostrea angulata*
Crassostrea sikamea	AB675951	*Crassostrea sikamea*	correct	correct	correct	correct	correct	correct	*Crassostrea angulata*
Crassostrea sikamea	AB675949	*Crassostrea sikamea*	correct	correct	correct	correct	correct	correct	*Crassostrea angulata*
Crassostrea sikamea	AB675947	*Crassostrea sikamea*	correct	correct	correct	correct	correct	correct	*Crassostrea angulata*
Cryptobranchia kuragiensis	AB543974	*Cryptobranchia kuragiensis*	correct	correct	correct	correct	correct	correct	*Cryptobranchia concentrica*
Cryptobranchia kuragiensis	AB238457	*Cryptobranchia kuragiensis*	correct	correct	correct	correct	correct	correct	*Cryptobranchia concentrica*
Anomalocardia producta	HQ703052	*Anomalocardia producta*	correct	correct	correct	correct	correct	correct	*Macridiscus aequilatera*
Anomalocardia producta	HQ703053	*Anomalocardia producta*	correct	correct	correct	correct	correct	correct	*Macridiscus aequilatera*
Anomalocardia producta	HQ703054	*Anomalocardia producta*	correct	correct	correct	correct	correct	correct	*Macridiscus aequilatera*
Anomalocardia producta	HQ703055	*Anomalocardia producta*	correct	correct	correct	correct	correct	correct	*Macridiscus aequilatera*
Anomalocardia producta	HQ703056	*Anomalocardia producta*	correct	correct	correct	correct	correct	correct	*Macridiscus aequilatera*
Cultellus attenuatus	JN859998	*Cultellus attenuatus*	correct	correct	correct	correct	correct	correct	*Sinonovacula rivularis*
Cultellus attenuatus	JN859999	*Cultellus attenuatus*	correct	correct	correct	correct	correct	correct	*Sinonovacula rivularis*
Cultellus attenuatus	JN860000	*Cultellus attenuatus*	correct	correct	correct	correct	correct	correct	*Sinonovacula rivularis*

Cultellus attenuatus	JN860001	*Cultellus attenuatus*	correct	correct	correct	correct	correct	correct	*Sinonovacula rivularis*
Cuvierina pacifica	KP292770	*Cuvierina pacifica*	no id	correct	correct	no id	correct	correct	*Cuvierina pacifica*
Cuvierina pacifica	KP292769	*Cuvierina pacifica*	no id	no id	correct	no id	no id	correct	*Cuvierina pacifica*
Cuvierina pacifica	KP292768	*Cuvierina pacifica*	no id	correct	correct	no id	correct	correct	*Cuvierina pacifica*
Cuvierina pacifica	KP292767	*Cuvierina pacifica*	no id	correct	correct	no id	correct	correct	*Cuvierina pacifica*
Cuvierina pacifica	KP292766	*Cuvierina pacifica*	no id	correct	correct	no id	correct	correct	*Cuvierina pacifica*
Cyclina sinensis	HQ703115	*Cyclina sinensis*	correct	correct	correct	correct	correct	correct	*Chamelea gallina*
Cyclina sinensis	HQ703116	*Cyclina sinensis*	correct	correct	correct	correct	correct	correct	*Chamelea gallina*
Cyclina sinensis	HQ703117	*Cyclina sinensis*	correct	correct	correct	correct	correct	correct	*Chamelea gallina*
Cyclina sinensis	HQ703118	*Cyclina sinensis*	correct	correct	correct	correct	correct	correct	*Chamelea gallina*
Cyclina sinensis	HQ703119	*Cyclina sinensis*	correct	correct	correct	correct	correct	correct	*Chamelea gallina*
Cyclina sinensis	HQ703120	*Cyclina sinensis*	correct	correct	correct	correct	correct	correct	*Chamelea gallina*
Cyclina sinensis	HQ703121	*Cyclina sinensis*	correct	correct	correct	correct	correct	correct	*Chamelea gallina*
Cyclina sinensis	HQ703122	*Cyclina sinensis*	correct	correct	correct	correct	correct	correct	*Chamelea gallina*
Cyclina sinensis	HQ703123	*Cyclina sinensis*	correct	correct	correct	correct	correct	correct	*Chamelea gallina*
Cyclina sinensis	HQ703124	*Cyclina sinensis*	correct	correct	correct	correct	correct	correct	*Chamelea gallina*
Cyclina sinensis	HQ703125	*Cyclina sinensis*	correct	correct	correct	correct	correct	correct	*Chamelea gallina*
Cyclina sinensis	HQ703126	*Cyclina sinensis*	correct	correct	correct	correct	correct	correct	*Chamelea gallina*
Cyclina sinensis	HQ703127	*Cyclina sinensis*	correct	correct	correct	correct	correct	correct	*Chamelea gallina*
Cyclina sinensis	HQ703128	*Cyclina sinensis*	correct	correct	correct	correct	correct	correct	*Chamelea gallina*
Cyclina sinensis	HQ703129	*Cyclina sinensis*	correct	correct	correct	correct	correct	correct	*Chamelea gallina*
Cyclina sinensis	HQ703130	*Cyclina sinensis*	correct	correct	correct	correct	correct	correct	*Chamelea gallina*
Cyclina sinensis	HQ703131	*Cyclina sinensis*	correct	correct	correct	correct	correct	correct	*Chamelea gallina*
Cyclina sinensis	HM021149	*Cyclina sinensis*	correct	correct	correct	correct	correct	correct	*Chamelea gallina*
Cyclina sinensis	HM021148	*Cyclina sinensis*	correct	correct	correct	correct	correct	correct	*Chamelea gallina*
Cyclina sinensis	HM021147	*Cyclina sinensis*	correct	correct	correct	correct	correct	correct	*Chamelea gallina*
Sunetta concinna	JN898937	*Meretrix petechialis*	no id	no id	no id	no id	no id	no id	*Cyclina sinensis*
Sunetta menstrualis	HQ703213	*Sunetta menstrualis*	ambiguous	ambiguous	ambiguous	ambiguous	ambiguous	ambiguous	*Chamelea gallina*
Sunetta menstrualis	HQ703214	*Sunetta menstrualis*	correct	correct	correct	correct	correct	correct	*Chamelea gallina*
Sunetta menstrualis	HQ703215	*Sunetta menstrualis*	correct	correct	correct	correct	correct	correct	*Chamelea gallina*
Sunetta menstrualis	HQ703216	*Sunetta menstrualis*	correct	correct	correct	correct	correct	correct	*Chamelea gallina*

Sunetta menstrualis	HM124589	*Sunetta menstrualis*	correct	correct	correct	correct	correct	correct	*Chamelea gallina*
Sunetta menstrualis	HM124588	*Sunetta menstrualis*	correct	correct	correct	correct	correct	correct	*Chamelea gallina*
Cymatium cingulatum	JF693367	*Cymatium cingulatum*	correct	correct	correct	correct	correct	correct	*Cymatium labiosum*
Cymatium cingulatum	JF693368	*Cymatium cingulatum*	correct	correct	correct	correct	correct	correct	*Cymatium labiosum*
Cypraea tigris	JF693369	*Cypraea tigris*	correct	correct	correct	correct	correct	correct	*Cypraea tigris*
Cypraea tigris	JF693370	*Cypraea tigris*	correct	correct	correct	correct	correct	correct	*Cypraea tigris*
Cypraea vitellus	JF693371	*Cypraea tigris*	no id	no id	no id	no id	no id	no id	*Lyncina propinqua*
Decatopecten pilica	GU120026	*Decatopecten plica*	no id	correct	correct	no id	correct	correct	
Decatopecten pilica	GU120027	*Decatopecten plica*	correct	correct	correct	correct	correct	correct	
Decatopecten pilica	GU120029	*Decatopecten plica*	correct	correct	correct	correct	correct	correct	
Decatopecten pilica	GU120030	*Decatopecten plica*	no id	correct	correct	no id	correct	correct	
Didimacar tenebrica	KU341932	*Didimacar tenebrica*	correct	correct	correct	correct	correct	correct	
Didimacar tenebrica	KU341933	*Didimacar tenebrica*	correct	correct	correct	correct	correct	correct	
Didimacar tenebrica	KU341934	*Didimacar tenebrica*	correct	correct	correct	correct	correct	correct	
Didimacar tenebrica	KU341935	*Didimacar tenebrica*	correct	correct	correct	correct	correct	correct	
Didimacar tenebrica	KU341936	*Didimacar tenebrica*	correct	correct	correct	correct	correct	correct	
Didimacar tenebrica	HQ258871	*Didimacar tenebrica*	correct	correct	correct	correct	correct	correct	
Dillwynella vitrea	AM049336	*Dillwynella vitrea*	no id	correct	correct	no id	correct	correct	*Dillwynella sp.*
Dillwynella vitrea	EU530143	*Dillwynella vitrea*	no id	correct	correct	no id	correct	correct	*Dillwynella sp.*
Diloma piperinus	AB505288	*Pseudostomatella decolorata*	no id	no id	no id	no id	no id	no id	*Diloma constellatus*
Diloma radula	AY858090	*Gonatopsis octopedatus*	no id	no id	no id	no id	no id	no id	*Diloma samoaensis*
Diloma suavis	AB505281	*Stomatella planulata*	no id	no id	no id	no id	no id	no id	*Osilinus punctulatus*
Distorsio reticularis	JF693372	*Distorsio reticularis*	correct	correct	correct	correct	correct	correct	*Distorsio reticularis*
Distorsio reticularis	JF693373	*Distorsio reticularis*	correct	correct	correct	correct	correct	correct	*Distorsio reticularis*
Donax dysoni	JN859978	*Donax dysoni*	correct	correct	correct	correct	correct	correct	*Psammotaea sp.*
Donax dysoni	JN859979	*Donax dysoni*	correct	correct	correct	correct	correct	correct	*Psammotaea sp.*
Donax dysoni	JN859980	*Donax dysoni*	correct	correct	correct	correct	correct	correct	*Psammotaea sp.*
Donax dysoni	JN859981	*Donax dysoni*	correct	correct	correct	correct	correct	correct	*Psammotaea sp.*
Donax dysoni	JN859976	*Donax dysoni*	correct	correct	correct	correct	correct	correct	*Psammotaea sp.*
Donax dysoni	JN859977	*Donax dysoni*	correct	correct	correct	correct	correct	correct	*Psammotaea sp.*
Dosinia angulosa	HQ703148	*Dosinia angulosa*	correct	correct	correct	correct	correct	correct	*Dosinia corrugata*

Dosinia angulosa	HQ703149	*Dosinia angulosa*	correct	correct	correct	correct	correct	correct	*Dosinia corrugata*
Dosinia angulosa	HQ703150	*Dosinia angulosa*	correct	correct	correct	correct	correct	correct	*Dosinia corrugata*
Dosinia angulosa	HQ703151	*Dosinia angulosa*	no id	correct	correct	no id	correct	correct	*Dosinia corrugata*
Dosinia biscocta	HM124577	*Dosinia fibula*	ambiguous	ambiguous	ambiguous	ambiguous	ambiguous	ambiguous	*Dosinia troscheli*
Dosinia biscocta	HM124572	*Dosinia fibula*	ambiguous	ambiguous	ambiguous	ambiguous	ambiguous	ambiguous	*Dosinia troscheli*
Dosinia fibula	HQ703132	*Dosinia fibula*	correct	correct	correct	correct	correct	correct	*Dosinia troscheli*
Dosinia fibula	HQ703133	*Dosinia fibula*	ambiguous	ambiguous	ambiguous	ambiguous	ambiguous	ambiguous	*Dosinia troscheli*
Dosinia fibula	HQ703134	*Dosinia fibula*	no id	ambiguous	ambiguous	no id	ambiguous	ambiguous	*Dosinia troscheli*
Dosinia fibula	HQ703135	*Dosinia fibula*	ambiguous	ambiguous	ambiguous	ambiguous	ambiguous	ambiguous	*Dosinia troscheli*
Dosinia fibula	HQ703136	*Dosinia fibula*	ambiguous	ambiguous	ambiguous	ambiguous	ambiguous	ambiguous	*Dosinia troscheli*
Dosinia corrugata	HQ703141	*Dosinia corrugata*	correct	correct	correct	correct	correct	ambiguous	*Dosinia japonica*
Dosinia corrugata	HQ703142	*Dosinia corrugata*	correct	correct	correct	correct	correct	correct	*Dosinia japonica*
Dosinia corrugata	HQ703143	*Dosinia corrugata*	correct	correct	correct	correct	correct	correct	*Dosinia japonica*
Dosinia corrugata	HQ703144	*Dosinia corrugata*	correct	correct	correct	correct	correct	correct	*Dosinia japonica*
Dosinia corrugata	HQ703145	*Dosinia corrugata*	correct	correct	correct	correct	correct	correct	*Dosinia japonica*
Dosinia corrugata	HQ703146	*Dosinia corrugata*	correct	correct	correct	correct	correct	correct	*Dosinia japonica*
Dosinia corrugata	HQ703147	*Dosinia corrugata*	no id	correct	correct	no id	correct	correct	*Dosinia japonica*
Dosinia corrugata	JN898932	*Dosinia corrugata*	correct	correct	correct	correct	correct	correct	*Dosinia japonica*
Dosinia corrugata	HM124573	*Dosinia corrugata*	correct	correct	correct	correct	correct	correct	*Dosinia japonica*
Dosinia japonicum	HQ703137	*Dosinia japonicum*	ambiguous	ambiguous	ambiguous	ambiguous	ambiguous	ambiguous	*Dosinia troscheli*
Dosinia japonicum	HQ703138	*Dosinia japonicum*	correct	correct	correct	correct	correct	correct	*Dosinia troscheli*
Dosinia troscheli	HQ703139	*Dosinia troscheli*	correct	correct	correct	correct	correct	correct	*Dosinia fibula*
Dosinia troscheli	HQ703140	*Dosinia troscheli*	correct	correct	correct	correct	correct	ambiguous	*Dosinia fibula*
Drupa albolabris	HE584499	*Drupa albolabris*	correct	correct	correct	correct	correct	correct	*Muricidae sp.*
Drupa albolabris	HE584498	*Drupa albolabris*	correct	correct	correct	correct	correct	correct	*Muricidae sp.*
Drupa grossularia	HE584488	*Cerithidea ornate*	no id	no id	no id	no id	no id	no id	*Drupa grossularia*
Drupa rubusidaeus	HE584527	*Drupa rubusidaeus*	correct	correct	correct	correct	correct	correct	*Drupa speciosa*
Drupa rubusidaeus	HE584526	*Drupa rubusidaeus*	correct	correct	correct	correct	correct	correct	*Drupa speciosa*
Drupella cornus	FR853820	*Drupella eburnea*	no id	no id	no id	no id	no id	no id	*Drupella sp.*
Drupella eburnea	FR853828	*Drupella cornus*	no id	no id	no id	no id	no id	no id	*Drupella sp.*
Drupella fragum	FR853892	*Drupella fragum*	correct	correct	correct	correct	correct	correct	*Ergalatax margariticola*

Drupella fragum	FR853890	*Drupella fragum*	correct	correct	correct	correct	correct	correct	*Ergalatax margariticola*
Drupella fragum	FR853888	*Drupella fragum*	correct	correct	correct	correct	correct	correct	*Ergalatax margariticola*
Drupella fragum	FR853886	*Drupella fragum*	correct	correct	correct	correct	correct	correct	*Ergalatax margariticola*
Drupella fragum	FR853884	*Drupella fragum*	correct	correct	correct	correct	correct	correct	*Ergalatax margariticola*
Drupella fragum	FR853846	*Drupella fragum*	correct	correct	correct	correct	correct	correct	*Ergalatax margariticola*
Drupella fragum	FR853891	*Drupella fragum*	correct	correct	correct	correct	correct	correct	*Ergalatax margariticola*
Drupella fragum	FR853889	*Drupella fragum*	correct	correct	correct	correct	correct	correct	*Ergalatax margariticola*
Drupella fragum	FR853887	*Drupella fragum*	correct	correct	correct	correct	correct	correct	*Ergalatax margariticola*
Drupella fragum	FR853885	*Drupella fragum*	correct	correct	correct	correct	correct	correct	*Ergalatax margariticola*
Drupella fragum	FR853847	*Drupella fragum*	correct	correct	correct	correct	correct	correct	*Ergalatax margariticola*
Drupella fragum	FR853845	*Drupella fragum*	correct	correct	correct	correct	correct	correct	*Ergalatax margariticola*
Drupella margariticola	JN053037	*Drupella margariticola*	correct	correct	correct	correct	correct	correct	*Drupella margariticola*
Drupella margariticola	JN053036	*Drupella margariticola*	correct	correct	correct	correct	correct	correct	*Drupella margariticola*
Drupella margariticola	GU188268	*Drupella margariticola*	correct	correct	correct	correct	correct	correct	*Drupella margariticola*
Drupella margariticola	GU188269	*Drupella margariticola*	correct	correct	correct	correct	correct	correct	*Drupella margariticola*
Drupella margariticola	GU188270	*Drupella margariticola*	correct	correct	correct	correct	correct	correct	*Drupella margariticola*
Drupella margariticola	GU188271	*Drupella margariticola*	correct	correct	correct	correct	correct	correct	*Drupella margariticola*
Drupella margariticola	FR853858	*Drupella margariticola*	correct	correct	correct	correct	correct	correct	*Drupella margariticola*
Drupella margariticola	FR853859	*Drupella margariticola*	no id	no id	correct	no id	no id	correct	*Drupella margariticola*
Drupella margariticola	FR853857	*Drupella margariticola*	correct	correct	correct	correct	correct	correct	*Drupella margariticola*
Drupella margariticola	FR853823	*Drupella margariticola*	correct	correct	correct	correct	correct	correct	*Drupella margariticola*
Drupella margariticola	FR853860	*Drupella margariticola*	correct	correct	correct	correct	correct	correct	*Drupella margariticola*
Drupella margariticola	FR853862	*Drupella margariticola*	correct	correct	correct	correct	correct	correct	*Drupella margariticola*
Drupella margariticola	FR853861	*Drupella margariticola*	correct	correct	correct	correct	correct	correct	*Drupella margariticola*
Drupella rugosa	FR853848	*Drupella rugosa*	correct	correct	correct	correct	correct	correct	*Ergalatax margariticola*
Drupella rugosa	FR853838	*Drupella rugosa*	correct	correct	correct	correct	correct	correct	*Ergalatax margariticola*
Drupella rugosa	FR853836	*Drupella rugosa*	correct	correct	correct	correct	correct	correct	*Ergalatax margariticola*
Drupella rugosa	FR853834	*Drupella rugosa*	correct	correct	correct	correct	correct	correct	*Ergalatax margariticola*
Drupella rugosa	FR853832	*Drupella rugosa*	correct	correct	correct	correct	correct	correct	*Ergalatax margariticola*
Drupella rugosa	FR853837	*Drupella rugosa*	correct	correct	correct	correct	correct	correct	*Ergalatax margariticola*
Drupella rugosa	FR853835	*Drupella rugosa*	correct	correct	correct	correct	correct	correct	*Ergalatax margariticola*

Drupella rugosa	FR853833	*Drupella rugosa*	correct	correct	correct	correct	correct	correct	*Ergalatax margariticola*
Drupella rugosa	FR853831	*Drupella rugosa*	correct	correct	correct	correct	correct	correct	*Ergalatax margariticola*
Drupella rugosa	FR853829	*Drupella rugosa*	no id	correct	correct	no id	correct	correct	*Ergalatax margariticola*
Drupella rugosa	FR853827	*Drupella rugosa*	correct	correct	correct	correct	correct	correct	*Ergalatax margariticola*
Duplicaria dussumieri	HQ834094	*Turricula javana*	no id	no id	no id	no id	no id	no id	*Duplicaria sp.*
Echinolittorina cinerea	AJ622991	*Echinolittorina reticulata*	no id	no id	no id	no id	no id	no id	*Echinolittorina hawaiiensis*
Echinolittorina radiata	JF693405	*Echinolittorina radiata*	correct	correct	correct	correct	correct	correct	*Echinolittorina leucosticta*
Echinolittorina radiata	JF693406	*Echinolittorina radiata*	correct	correct	correct	correct	correct	correct	*Echinolittorina leucosticta*
Echinolittorina radiata	AJ623040	*Echinolittorina radiata*	correct	correct	correct	correct	correct	correct	*Echinolittorina leucosticta*
Echinolittorina reticulata	AJ623042	*Echinolittorina reticulata*	correct	correct	correct	correct	correct	correct	*Echinolittorina hawaiiensis*
Echinolittorina reticulata	AM157057	*Echinolittorina reticulata*	correct	correct	correct	correct	correct	correct	*Echinolittorina hawaiiensis*
Echinolittorina reticulata	AM157058	*Echinolittorina reticulata*	correct	correct	correct	correct	correct	correct	*Echinolittorina hawaiiensis*
Echinolittorina reticulata	AM157059	*Echinolittorina reticulata*	correct	correct	correct	correct	correct	correct	*Echinolittorina hawaiiensis*
Echinolittorina reticulata	AM157060	*Echinolittorina reticulata*	correct	correct	correct	correct	correct	correct	*Echinolittorina hawaiiensis*
Echinolittorina reticulata	AM157061	*Echinolittorina reticulata*	correct	correct	correct	correct	correct	correct	*Echinolittorina hawaiiensis*
Echinolittorina reticulata	AM157062	*Echinolittorina reticulata*	correct	correct	correct	correct	correct	correct	*Echinolittorina hawaiiensis*
Echinolittorina reticulata	AM157063	*Echinolittorina reticulata*	correct	correct	correct	correct	correct	correct	*Echinolittorina hawaiiensis*
Echinolittorina reticulata	AM157064	*Echinolittorina reticulata*	correct	correct	correct	correct	correct	correct	*Echinolittorina hawaiiensis*
Echinolittorina reticulata	AM157065	*Echinolittorina reticulata*	correct	correct	correct	correct	correct	correct	*Echinolittorina hawaiiensis*
Echinolittorina reticulata	AM157066	*Echinolittorina reticulata*	correct	correct	correct	correct	correct	correct	*Echinolittorina hawaiiensis*
Echinolittorina reticulata	AM157067	*Echinolittorina reticulata*	correct	correct	correct	correct	correct	correct	*Echinolittorina hawaiiensis*
Echinolittorina trochoides A	AM157086	*Echinolittorina trochoides*	no id	no id	no id	no id	no id	no id	*Echinolittorina trochoides A*
Echinolittorina trochoides E	AJ623057	*Echinolittorina trochoides*	correct	correct	correct	correct	correct	correct	*Echinolittorina trochoides D*
Echinolittorina trochoides E	AJ623058	*Echinolittorina trochoides*	correct	correct	correct	correct	correct	correct	*Echinolittorina trochoides D*
Echinolittorina trochoides E	AM156817	*Echinolittorina trochoides*	correct	correct	correct	correct	correct	correct	*Echinolittorina trochoides D*
Echinolittorina trochoides E	AM156818	*Echinolittorina trochoides*	correct	correct	correct	correct	correct	correct	*Echinolittorina trochoides D*
Echinolittorina trochoides E	AM156819	*Echinolittorina trochoides*	correct	correct	correct	correct	correct	correct	*Echinolittorina trochoides D*
Echinolittorina trochoides E	AM156820	*Echinolittorina trochoides*	correct	correct	correct	correct	correct	correct	*Echinolittorina trochoides D*
Echinolittorina trochoides E	AM156821	*Echinolittorina trochoides*	correct	correct	correct	correct	correct	correct	*Echinolittorina trochoides D*
Echinolittorina trochoides E	AM156822	*Echinolittorina trochoides*	correct	correct	correct	correct	correct	correct	*Echinolittorina trochoides D*
Echinolittorina trochoides E	AM156823	*Echinolittorina trochoides*	no id	correct	correct	no id	correct	correct	*Echinolittorina trochoides D*

Echinolittorina trochoides E	AM156824	*Echinolittorina trochoides*	correct	correct	correct	correct	correct	correct	*Echinolittorina trochoides D*
Echinolittorina trochoides E	AM156825	*Echinolittorina trochoides*	correct	correct	correct	correct	correct	correct	*Echinolittorina trochoides D*
Echinolittorina trochoides E	AM156826	*Echinolittorina trochoides*	correct	correct	correct	correct	correct	correct	*Echinolittorina trochoides D*
Echinolittorina trochoides E	AM156827	*Echinolittorina trochoides*	correct	correct	correct	correct	correct	correct	*Echinolittorina trochoides D*
Echinolittorina trochoides E	AM156828	*Echinolittorina trochoides*	correct	correct	correct	correct	correct	correct	*Echinolittorina trochoides D*
Echinolittorina trochoides E	AM156829	*Echinolittorina trochoides*	correct	correct	correct	correct	correct	correct	*Echinolittorina trochoides D*
Echinolittorina trochoides E	AM156830	*Echinolittorina trochoides*	correct	correct	correct	correct	correct	correct	*Echinolittorina trochoides D*
Echinolittorina trochoides E	AM156831	*Echinolittorina trochoides*	no id	correct	correct	no id	correct	correct	*Echinolittorina trochoides D*
Echinolittorina trochoides E	AM156832	*Echinolittorina trochoides*	correct	correct	correct	correct	correct	correct	*Echinolittorina trochoides D*
Echinolittorina trochoides E	AM156833	*Echinolittorina trochoides*	correct	correct	correct	correct	correct	correct	*Echinolittorina trochoides D*
Echinolittorina trochoides E	AM156834	*Echinolittorina trochoides*	correct	correct	correct	correct	correct	correct	*Echinolittorina trochoides D*
Echinolittorina trochoides E	AM156835	*Echinolittorina trochoides*	correct	correct	correct	correct	correct	correct	*Echinolittorina trochoides D*
Echinolittorina vidua	AM156986	*Echinolittorina vidua*	correct	correct	correct	correct	correct	correct	*Echinolittorina vidua*
Echinolittorina vidua	AM156985	*Echinolittorina vidua*	correct	correct	correct	correct	correct	correct	*Echinolittorina vidua*
Echinolittorina vidua	AM156984	*Echinolittorina vidua*	no id	correct	correct	no id	correct	correct	*Echinolittorina vidua*
Echinolittorina vidua	AM156983	*Echinolittorina vidua*	correct	correct	correct	correct	correct	correct	*Echinolittorina vidua*
Echinolittorina vidua	AM156982	*Echinolittorina vidua*	no id	no id	correct	no id	no id	correct	*Echinolittorina vidua*
Echinolittorina vidua	AM156981	*Echinolittorina vidua*	no id	correct	correct	no id	correct	correct	*Echinolittorina vidua*
Echinolittorina vidua	AM157002	*Echinolittorina vidua*	correct	correct	correct	correct	correct	correct	*Echinolittorina vidua*
Echinolittorina vidua	AM157003	*Echinolittorina vidua*	correct	correct	correct	correct	correct	correct	*Echinolittorina vidua*
Echinolittorina vidua	AM157004	*Echinolittorina vidua*	correct	correct	correct	correct	correct	correct	*Echinolittorina vidua*
Echinolittorina vidua	AM157005	*Echinolittorina vidua*	no id	no id	correct	no id	no id	correct	*Echinolittorina vidua*
Echinolittorina vidua	AM157006	*Echinolittorina vidua*	correct	correct	correct	correct	correct	correct	*Echinolittorina vidua*
Echinolittorina vidua	AM157007	*Echinolittorina vidua*	correct	correct	correct	correct	correct	correct	*Echinolittorina vidua*
Echinolittorina vidua	AM157008	*Echinolittorina vidua*	correct	correct	correct	correct	correct	correct	*Echinolittorina vidua*
Echinolittorina vidua	AM157009	*Echinolittorina vidua*	correct	correct	correct	correct	correct	correct	*Echinolittorina vidua*
Elysia abei	AB758955	*Elysia abei*	ambiguous	ambiguous	ambiguous	ambiguous	ambiguous	ambiguous	*Elysia sp.*
Elysia abei	AB758954	*Elysia abei*	ambiguous	ambiguous	ambiguous	ambiguous	ambiguous	ambiguous	*Elysia sp.*
Elysia abei	AB758953	*Elysia abei*	ambiguous	ambiguous	ambiguous	ambiguous	ambiguous	ambiguous	*Elysia sp.*
Elysia abei	KC573711	*Elysia abei*	ambiguous	ambiguous	ambiguous	ambiguous	ambiguous	ambiguous	*Elysia sp.*
Elysia abei	KC573712	*Elysia abei*	no id	ambiguous	ambiguous	no id	ambiguous	ambiguous	*Elysia sp.*

Elysia abei	KC573713	*Elysia abei*	no id	ambiguous	ambiguous	no id	ambiguous	ambiguous	*Elysia sp.*
Elysia amakusana	AB758956	*Elysia abei*	incorrect	incorrect	incorrect	incorrect	incorrect	incorrect	*Elysia cf. marginata*
Elysia atroviridis	AB758942	*Elysia atroviridis*	ambiguous	ambiguous	ambiguous	ambiguous	ambiguous	ambiguous	*Elysia cf. tomentosa*
Elysia atroviridis	AB758941	*Elysia atroviridis*	correct	correct	correct	correct	ambiguous	ambiguous	*Elysia cf. tomentosa*
Elysia atroviridis	AB758940	*Elysia atroviridis*	ambiguous	ambiguous	ambiguous	ambiguous	ambiguous	ambiguous	*Elysia cf. tomentosa*
Elysia atroviridis	AB758939	*Elysia atroviridis*	ambiguous	ambiguous	ambiguous	ambiguous	ambiguous	ambiguous	*Elysia cf. tomentosa*
Elysia atroviridis	AB758938	*Elysia atroviridis*	ambiguous	ambiguous	ambiguous	ambiguous	ambiguous	ambiguous	*Elysia cf. tomentosa*
Elysia atroviridis	AB758937	*Elysia atroviridis*	ambiguous	ambiguous	ambiguous	ambiguous	ambiguous	ambiguous	*Elysia cf. tomentosa*
Elysia atroviridis	AB758936	*Elysia atroviridis*	ambiguous	ambiguous	ambiguous	ambiguous	ambiguous	ambiguous	*Elysia cf. tomentosa*
Elysia atroviridis	AB758935	*Elysia atroviridis*	no id	ambiguous	ambiguous	no id	ambiguous	ambiguous	*Elysia cf. tomentosa*
Elysia atroviridis	AB758934	*Elysia atroviridis*	ambiguous	ambiguous	ambiguous	ambiguous	ambiguous	ambiguous	*Elysia cf. tomentosa*
Elysia atroviridis	AB758933	*Elysia atroviridis*	ambiguous	ambiguous	ambiguous	ambiguous	ambiguous	ambiguous	*Elysia cf. tomentosa*
Elysia atroviridis	AB758932	*Elysia atroviridis*	ambiguous	ambiguous	ambiguous	ambiguous	ambiguous	ambiguous	*Elysia cf. tomentosa*
Elysia atroviridis	AB758931	*Elysia atroviridis*	ambiguous	ambiguous	ambiguous	ambiguous	ambiguous	ambiguous	*Elysia cf. tomentosa*
Elysia atroviridis	AB758930	*Elysia atroviridis*	correct	correct	correct	correct	ambiguous	ambiguous	*Elysia cf. tomentosa*
Elysia atroviridis	AB758929	*Elysia atroviridis*	ambiguous	ambiguous	ambiguous	ambiguous	ambiguous	ambiguous	*Elysia cf. tomentosa*
Elysia atroviridis	AB758928	*Elysia atroviridis*	no id	ambiguous	ambiguous	no id	ambiguous	ambiguous	*Elysia cf. tomentosa*
Elysia atroviridis	AB758927	*Elysia atroviridis*	ambiguous	ambiguous	ambiguous	ambiguous	ambiguous	ambiguous	*Elysia cf. tomentosa*
Elysia atroviridis	AB758926	*Elysia atroviridis*	ambiguous	ambiguous	ambiguous	ambiguous	ambiguous	ambiguous	*Elysia cf. tomentosa*
Elysia atroviridis	AB758925	*Elysia atroviridis*	ambiguous	ambiguous	ambiguous	ambiguous	ambiguous	ambiguous	*Elysia cf. tomentosa*
Elysia atroviridis	AB758924	*Elysia atroviridis*	no id	no id	ambiguous	no id	no id	ambiguous	*Elysia cf. tomentosa*
Elysia atroviridis	AB758923	*Elysia atroviridis*	ambiguous	ambiguous	ambiguous	ambiguous	ambiguous	ambiguous	*Elysia cf. tomentosa*
Elysia atroviridis	AB758922	*Elysia atroviridis*	ambiguous	ambiguous	ambiguous	ambiguous	ambiguous	ambiguous	*Elysia cf. tomentosa*
Elysia atroviridis	AB758921	*Elysia atroviridis*	ambiguous	ambiguous	ambiguous	ambiguous	ambiguous	ambiguous	*Elysia cf. tomentosa*
Elysia atroviridis	AB758920	*Elysia atroviridis*	ambiguous	ambiguous	ambiguous	ambiguous	ambiguous	ambiguous	*Elysia cf. tomentosa*
Elysia atroviridis	AB758919	*Elysia atroviridis*	ambiguous	ambiguous	ambiguous	ambiguous	ambiguous	ambiguous	*Elysia cf. tomentosa*
Elysia atroviridis	AB758918	*Elysia atroviridis*	ambiguous	ambiguous	ambiguous	ambiguous	ambiguous	ambiguous	*Elysia cf. tomentosa*
Elysia atroviridis	AB758917	*Elysia atroviridis*	ambiguous	ambiguous	ambiguous	ambiguous	ambiguous	ambiguous	*Elysia cf. tomentosa*
Elysia atroviridis	AB758916	*Elysia atroviridis*	ambiguous	ambiguous	ambiguous	ambiguous	ambiguous	ambiguous	*Elysia cf. tomentosa*
Elysia atroviridis	AB758915	*Elysia atroviridis*	ambiguous	ambiguous	ambiguous	ambiguous	ambiguous	ambiguous	*Elysia cf. tomentosa*
Elysia atroviridis	AB758914	*Elysia atroviridis*	no id	ambiguous	ambiguous	no id	ambiguous	ambiguous	*Elysia cf. tomentosa*

Elysia atroviridis	AB758913	*Elysia atroviridis*	ambiguous	ambiguous	ambiguous	ambiguous	ambiguous	ambiguous	*Elysia cf. tomentosa*
Elysia atroviridis	AB758912	*Elysia atroviridis*	ambiguous	ambiguous	ambiguous	ambiguous	ambiguous	ambiguous	*Elysia cf. tomentosa*
Elysia atroviridis	AB758911	*Elysia atroviridis*	ambiguous	ambiguous	ambiguous	ambiguous	ambiguous	ambiguous	*Elysia cf. tomentosa*
Elysia atroviridis	AB758910	*Elysia atroviridis*	ambiguous	ambiguous	ambiguous	ambiguous	ambiguous	ambiguous	*Elysia cf. tomentosa*
Elysia atroviridis	AB758909	*Elysia atroviridis*	ambiguous	ambiguous	ambiguous	ambiguous	ambiguous	ambiguous	*Elysia cf. tomentosa*
Elysia atroviridis	AB758908	*Elysia atroviridis*	correct	correct	correct	correct	ambiguous	ambiguous	*Elysia cf. tomentosa*
Elysia atroviridis	AB758907	*Elysia atroviridis*	ambiguous	ambiguous	ambiguous	ambiguous	ambiguous	ambiguous	*Elysia cf. tomentosa*
Elysia atroviridis	AB758906	*Elysia atroviridis*	ambiguous	ambiguous	ambiguous	ambiguous	ambiguous	ambiguous	*Elysia cf. tomentosa*
Elysia atroviridis	AB758905	*Elysia atroviridis*	ambiguous	ambiguous	ambiguous	ambiguous	ambiguous	ambiguous	*Elysia cf. tomentosa*
Elysia atroviridis	AB758949	*Elysia atroviridis*	ambiguous	ambiguous	ambiguous	ambiguous	ambiguous	ambiguous	*Elysia cf. tomentosa*
Elysia atroviridis	AB758948	*Elysia atroviridis*	ambiguous	ambiguous	ambiguous	ambiguous	ambiguous	ambiguous	*Elysia cf. tomentosa*
Elysia atroviridis	AB758947	*Elysia atroviridis*	ambiguous	ambiguous	ambiguous	ambiguous	ambiguous	ambiguous	*Elysia cf. tomentosa*
Elysia atroviridis	AB758946	*Elysia atroviridis*	ambiguous	ambiguous	ambiguous	ambiguous	ambiguous	ambiguous	*Elysia cf. tomentosa*
Elysia atroviridis	AB758945	*Elysia atroviridis*	no id	ambiguous	ambiguous	no id	ambiguous	ambiguous	*Elysia cf. tomentosa*
Elysia atroviridis	AB758944	*Elysia atroviridis*	ambiguous	ambiguous	ambiguous	ambiguous	ambiguous	ambiguous	*Elysia cf. tomentosa*
Elysia atroviridis	AB758943	*Elysia atroviridis*	ambiguous	ambiguous	ambiguous	ambiguous	ambiguous	ambiguous	*Elysia cf. tomentosa*
Elysia atroviridis	KC573760	*Elysia atroviridis*	ambiguous	ambiguous	ambiguous	ambiguous	ambiguous	ambiguous	*Elysia cf. tomentosa*
Elysia setoensis	KC573761	*Elysia atroviridis*	incorrect	incorrect	incorrect	incorrect	incorrect	incorrect	*Elysia cf. tomentosa*
Elysia ornata	AB758967	*Elysia ornata*	correct	correct	correct	correct	correct	ambiguous	*Elysia cf. marginata*
Elysia ornata	AB758966	*Elysia ornata*	correct	correct	correct	correct	correct	ambiguous	*Elysia cf. marginata*
Elysia ornata	AB758964	*Elysia ornata*	no id	no id	correct	no id	no id	correct	*Elysia ornata*
Elysia ornata	AB758965	*Elysia ornata*	correct	correct	correct	correct	correct	ambiguous	*Elysia cf. marginata*
Elysia ornata	AB758963	*Elysia ornata*	correct	correct	correct	correct	correct	ambiguous	*Elysia cf. marginata*
Elysia ornata	AB758962	*Elysia ornata*	correct	correct	correct	correct	correct	ambiguous	*Elysia cf. marginata*
Elysia pusilla	AB758959	*Elysia pusilla*	correct	correct	correct	correct	correct	correct	*Elysia pusilla*
Elysia pusilla	AB758958	*Elysia pusilla*	correct	correct	correct	correct	correct	correct	*Elysia pusilla*
Elysia pusilla	AB758957	*Elysia pusilla*	correct	correct	correct	correct	correct	correct	*Elysia pusilla*
Elysia rufescens	AB758961	*Elysia rufescens*	no id	correct	correct	no id	correct	ambiguous	*Elysia ornata*
Elysia rufescens	AB758960	*Elysia rufescens*	no id	correct	correct	no id	correct	correct	*Elysia ornata*
Elysia trisinuata	AB758951	*Elysia atroviridis*	no id	no id	no id	no id	no id	no id	*Elysia marcusi*
Emarginula foveolata	AM049333	*Emarginula foveolata*	correct	correct	correct	correct	correct	correct	*Lineus ruber*

Emarginula foveolata	AB238593	*Emarginula foveolata*	correct	correct	correct	correct	correct	correct	*Lineus ruber*
Emarginula variegata	AB365213	*Emarginula variegata*	correct	correct	correct	correct	correct	correct	*Emarginula variegata*
Emarginula variegata	AB238594	*Emarginula variegata*	correct	correct	correct	correct	correct	correct	*Emarginula variegata*
Erronea errones	JF693396	*Erronea errones*	no id	no id	no id	no id	no id	no id	*Erronea caurica*
Erronea errones	JF693397	*Erronea errones*	no id	no id	no id	no id	no id	no id	*Erronea errones*
Ethaliella floccata	AB505304	*Ethaliella floccata*	correct	correct	correct	correct	correct	correct	*Ethaliella floccata*
Ethaliella floccata	AB505305	*Ethaliella floccata*	correct	correct	correct	correct	correct	correct	*Ethaliella floccata*
Ethaliella floccata	EU530137	*Ethaliella floccata*	correct	correct	correct	correct	correct	correct	*Ethaliella floccata*
Ethminolia stearnsii	AB505306	*Ethminolia stearnsii*	correct	correct	correct	correct	correct	correct	*Margarella antipoda rosea*
Ethminolia stearnsii	AB505307	*Ethminolia stearnsii*	correct	correct	correct	correct	correct	correct	*Margarella antipoda rosea*
Euplica scripta	HQ834054	*Euplica scripta*	correct	correct	correct	correct	correct	correct	*Amphissa versicolor*
Euplica scripta	JN052987	*Euplica scripta*	correct	correct	correct	correct	correct	correct	*Amphissa versicolor*
Euplica scripta	JN052986	*Euplica scripta*	correct	correct	correct	correct	correct	correct	*Amphissa versicolor*
Euplica scripta	JN052985	*Conus aristophanes*	no id	no id	incorrect	no id	no id	incorrect	*Conus abbreviatus*
Conus aristophanes	HQ834101	*Euplica scripta*	no id	no id	incorrect	no id	no id	incorrect	*Conus abbreviatus*
Euprymna berryi	HQ846099	*Euprymna berryi*	correct	correct	correct	correct	correct	correct	*Euprymna hyllebergi*
Euprymna berryi	HQ846100	*Euprymna berryi*	correct	correct	correct	correct	correct	correct	*Euprymna hyllebergi*
Euprymna berryi	HQ846101	*Euprymna berryi*	correct	correct	correct	correct	correct	correct	*Euprymna hyllebergi*
Euprymna berryi	HQ846102	*Euprymna berryi*	correct	correct	correct	correct	correct	correct	*Euprymna hyllebergi*
Euprymna morsei	HQ846103	*Euprymna morsei*	correct	correct	correct	correct	correct	correct	*Euprymna hyllebergi*
Euprymna morsei	HQ846104	*Euprymna morsei*	correct	correct	correct	correct	correct	correct	*Euprymna hyllebergi*
Euprymna morsei	HQ846105	*Euprymna morsei*	correct	correct	correct	correct	correct	correct	*Euprymna hyllebergi*
Eurytrochus cognatus	AB505299	*Eurytrochus cognatus*	correct	correct	correct	correct	correct	correct	*Notogibbula preissiana*
Eurytrochus cognatus	EU530133	*Eurytrochus cognatus*	correct	correct	correct	correct	correct	correct	*Notogibbula preissiana*
Ficus ficus	JF693374	*Ficus ficus*	no id	correct	correct	no id	correct	ambiguous	*Ficidae sp.*
Ficus ficus	JF693375	*Ficus ficus*	no id	correct	correct	no id	correct	ambiguous	*Ficidae sp.*
Ficus ficus	JF693376	*Ficus ficus*	no id	correct	correct	no id	correct	ambiguous	*Ficidae sp.*
Ficus gracilis	JF693380	*Ficus gracilis*	correct	correct	correct	correct	correct	correct	*Ficidae sp.*
Ficus gracilis	JF693381	*Ficus gracilis*	correct	correct	correct	correct	correct	correct	*Ficidae sp.*
Ficus gracilis	JF693382	*Ficus gracilis*	correct	correct	correct	correct	correct	correct	*Ficidae sp.*
Ficus variegata	JF693377	*Ficus variegata*	correct	correct	correct	correct	correct	ambiguous	*Clione antarctica*

Ficus variegata	JF693378	*Ficus variegata*	correct	correct	correct	correct	correct	ambiguous	*Clione antarctica*
Ficus variegata	JF693379	*Ficus variegata*	correct	correct	correct	correct	correct	ambiguous	*Clione antarctica*
Fusinus forceps	HM180580	*Fusinus forceps*	correct	correct	correct	correct	ambiguous	ambiguous	*Fusinus longicaudus*
Fusinus forceps	HM180581	*Fusinus forceps*	correct	correct	correct	correct	ambiguous	ambiguous	*Fusinus longicaudus*
Fusinus forceps	HM180582	*Fusinus longicaudus*	no id	incorrect	incorrect	no id	incorrect	ambiguous	*Fusinus longicaudus*
Fusinus forceps	HM180583	*Fusinus forceps*	correct	correct	correct	correct	ambiguous	ambiguous	*Fusinus longicaudus*
Fusinus forceps	HM180584	*Fusinus forceps*	correct	correct	correct	correct	ambiguous	ambiguous	*Fusinus longicaudus*
Fusinus longicaudus	HM180585	*Fusinus forceps*	no id	incorrect	incorrect	no id	incorrect	incorrect	*Fusinus longicaudus*
Fusinus longicaudus	HQ834100	*Fusinus longicaudus*	correct	correct	correct	correct	correct	correct	*Fusinus forceps*
Fusinus longicaudus	HQ834099	*Fusinus longicaudus*	correct	correct	correct	correct	correct	correct	*Fusinus forceps*
Gabrielona pisinna	AM049356	*Gabrielona pisinna*	correct	correct	correct	correct	correct	correct	*Turbo radiatus*
Gabrielona pisinna	AM049357	*Gabrielona pisinna*	correct	correct	correct	correct	correct	correct	*Turbo radiatus*
Gafrarium dispar	HQ703086	*Gafrarium dispar*	correct	correct	correct	correct	correct	correct	*Gafrarium tumidum*
Gafrarium dispar	HQ703087	*Gafrarium dispar*	correct	correct	correct	correct	correct	correct	*Gafrarium tumidum*
Gafrarium dispar	HQ703088	*Gafrarium dispar*	correct	correct	correct	correct	correct	correct	*Gafrarium tumidum*
Gafrarium dispar	HQ703089	*Gafrarium dispar*	correct	correct	correct	correct	correct	correct	*Gafrarium tumidum*
Gafrarium dispar	HQ703090	*Gafrarium dispar*	correct	correct	correct	correct	correct	correct	*Gafrarium tumidum*
Gafrarium dispar	HQ703091	*Gafrarium dispar*	correct	correct	correct	correct	correct	correct	*Gafrarium tumidum*
Gafrarium dispar	HQ703092	*Gafrarium dispar*	correct	correct	correct	correct	correct	correct	*Gafrarium tumidum*
Gafrarium dispar	HQ703093	*Gafrarium dispar*	correct	correct	correct	correct	correct	correct	*Gafrarium tumidum*
Gafrarium dispar	HQ703094	*Gafrarium dispar*	correct	correct	correct	correct	correct	correct	*Gafrarium dispar*
Gafrarium divaricatum	HQ703095	*Gafrarium divaricatum*	no id	no id	no id	no id	no id	no id	*Gafrarium dispar*
Gafrarium divaricatum	HQ703096	*Gafrarium divaricatum*	no id	no id	correct	no id	no id	correct	*Gafrarium dispar*
Gafrarium divaricatum	HQ703097	*Gafrarium divaricatum*	correct	correct	correct	correct	correct	correct	*Gafrarium dispar*
Gafrarium divaricatum	HQ703098	*Gafrarium divaricatum*	correct	correct	correct	correct	correct	correct	*Gafrarium dispar*
Gafrarium divaricatum	HQ703099	*Gafrarium divaricatum*	correct	correct	correct	correct	correct	correct	*Gafrarium dispar*
Gafrarium divaricatum	HQ703100	*Gafrarium divaricatum*	correct	correct	correct	correct	correct	correct	*Gafrarium dispar*
Gafrarium divaricatum	HQ703101	*Gafrarium divaricatum*	correct	correct	correct	correct	correct	correct	*Gafrarium dispar*
Gafrarium divaricatum	HQ703102	*Gafrarium divaricatum*	correct	correct	correct	correct	correct	correct	*Gafrarium dispar*
Gafrarium divaricatum	HQ703103	*Gafrarium divaricatum*	correct	correct	correct	correct	correct	correct	*Gafrarium dispar*
Gafrarium divaricatum	HQ703104	*Gafrarium divaricatum*	correct	correct	correct	correct	correct	correct	*Gafrarium dispar*

Gafrarium divaricatum	HQ703105	*Gafrarium divaricatum*	correct	correct	correct	correct	correct	correct	*Gafrarium dispar*
Gafrarium divaricatum	HQ703106	*Gafrarium divaricatum*	correct	correct	correct	correct	correct	correct	*Gafrarium dispar*
Gafrarium pectinatum	HQ703077	*Gafrarium pectinatum*	correct	correct	correct	correct	correct	correct	*Gafrarium tumidum*
Gafrarium pectinatum	HQ703078	*Gafrarium pectinatum*	correct	correct	correct	correct	correct	correct	*Gafrarium tumidum*
Gafrarium pectinatum	HQ703079	*Gafrarium pectinatum*	correct	correct	correct	correct	correct	correct	*Gafrarium tumidum*
Gafrarium pectinatum	HQ703080	*Gafrarium pectinatum*	correct	correct	correct	correct	correct	correct	*Gafrarium tumidum*
Gafrarium tumidum	HQ703081	*Gafrarium tumidum*	correct	correct	correct	correct	correct	correct	*Gafrarium tumidum*
Gafrarium tumidum	HQ703082	*Gafrarium tumidum*	correct	correct	correct	correct	correct	correct	*Gafrarium tumidum*
Gafrarium tumidum	HQ703083	*Gafrarium tumidum*	correct	correct	correct	correct	correct	correct	*Gafrarium tumidum*
Gafrarium tumidum	HQ703084	*Gafrarium tumidum*	correct	correct	correct	correct	correct	correct	*Gafrarium tumidum*
Gafrarium tumidum	HQ703085	*Gafrarium tumidum*	correct	correct	correct	correct	correct	correct	*Gafrarium tumidum*
Geloina erosa	AB076927	*Geloina expansa*	no id	no id	no id	no id	no id	no id	*Geloina expansa*
Geloina expansa	AB498812	*Geloina erosa*	no id	no id	no id	no id	no id	no id	*Geloina expansa*
Gemmula deshayesii	HQ834092	*Strombus vittatus*	no id	no id	no id	no id	no id	no id	*Gemmula hastula*
Gigantidas horikoshii	HF545113	*Gigantidas horikoshii*	correct	correct	correct	correct	correct	correct	*Idas sp.*
Gigantidas horikoshii	AB257538	*Gigantidas horikoshii*	correct	correct	correct	correct	correct	correct	*Idas sp.*
Ginebis argenteonitens	AB365231	*Ginebis argenteonitens*	correct	correct	correct	correct	correct	correct	*Cidarina cidaris*
Ginebis argenteonitens	EU530112	*Ginebis argenteonitens*	correct	correct	correct	correct	correct	correct	*Cidarina cidaris*
Ginebis argenteonitens	EU530111	*Ginebis argenteonitens*	correct	correct	correct	correct	correct	correct	*Cidarina cidaris*
Globivenus toreuma	HQ703342	*Placamen isabellina*	no id	no id	no id	no id	no id	no id	*Globivenus toreuma*
Glycymeris reevei	AB076933	*Barbatia lacerata*	no id	no id	no id	no id	no id	no id	*Cucullaea labiata*
Glycymeris rotunda	AB076934	*Scapharca gubernaculum*	no id	no id	no id	no id	no id	no id	*Glycymeris sp.*
Golikovia ennae	AB498779	*Neptunea mikawaensis*	no id	no id	no id	no id	no id	no id	*Golikovia fukueae*
Golikovia fukueae	AB498780	*Neptunea polycostata*	no id	no id	no id	no id	no id	no id	*Golikovia ennae*
Gonatopsis borealis	AB749275	*Gonatus madokai*	no id	no id	no id	no id	no id	no id	*Gonatopsis borealis*
Gonatopsis octopedatus	AB749282	*Gonatus madokai*	no id	no id	no id	no id	no id	no id	*Gonatopsis japonicus*
Gonatus berryi	AB749280	*Gonatus onyx*	no id	no id	incorrect	no id	no id	incorrect	*Gonatus fabricii*
Gonatus kamtschaticus	AB749274	*Gonatus madokai*	no id	no id	incorrect	no id	no id	incorrect	*Gonatus madokai*
Gonatus madokai	AB749284	*Gonatus madokai*	correct	correct	correct	correct	correct	correct	*Gonatus kamtschaticus*
Gonatus madokai	AB749283	*Gonatus madokai*	correct	correct	correct	correct	correct	correct	*Gonatus kamtschaticus*
Gonatus madokai	AB749278	*Gonatus madokai*	no id	correct	correct	no id	correct	correct	*Gonatus kamtschaticus*

Gonatus onyx	AB749279	*Gonatus berryi*	no id	no id	incorrect	no id	no id	incorrect	*Gonatus californiensis*
Gonatus pyros	AB749273	*Gonatus berryi*	no id	no id	no id	no id	no id	no id	*Gonatopsis japonicus*
Granata lyrata	AB365232	*Granata lyrata*	no id	correct	correct	no id	correct	correct	*Guildfordia sp.*
Granata lyrata	EU530114	*Granata lyrata*	no id	correct	correct	no id	correct	correct	*Guildfordia sp.*
Guildfordia triumphans	AM049375	*Guildfordia triumphans*	correct	correct	correct	correct	correct	correct	*Guildfordia sp.*
Guildfordia triumphans	AM049376	*Guildfordia triumphans*	correct	correct	correct	correct	correct	correct	*Guildfordia sp.*
Guildfordia yoka	AM049377	*Guildfordia yoka*	correct	correct	correct	correct	correct	correct	*Guildfordia sp.*
Guildfordia yoka	AM049378	*Guildfordia yoka*	correct	correct	correct	correct	correct	correct	*Guildfordia sp.*
Guildfordia yoka	EU530156	*Guildfordia yoka*	correct	correct	correct	correct	correct	correct	*Guildfordia sp.*
Haminoea japonica	JN830658	*Haminoea japonica*	correct	correct	correct	correct	correct	correct	*Haminoea japonica*
Haminoea japonica	JN830657	*Haminoea japonica*	correct	correct	correct	correct	correct	correct	*Haminoea japonica*
Haminoea japonica	JN830656	*Haminoea japonica*	correct	correct	correct	correct	correct	correct	*Haminoea japonica*
Haminoea japonica	JN830655	*Haminoea japonica*	correct	correct	correct	correct	correct	correct	*Haminoea japonica*
Haminoea japonica	JN830654	*Haminoea japonica*	correct	correct	correct	correct	correct	correct	*Haminoea japonica*
Haminoea japonica	JN830653	*Haminoea japonica*	correct	correct	correct	correct	correct	correct	*Haminoea japonica*
Haminoea japonica	JN830652	*Haminoea japonica*	correct	correct	correct	correct	correct	correct	*Haminoea japonica*
Haminoea japonica	KF572981	*Haminoea japonica*	correct	correct	correct	correct	correct	correct	*Haminoea japonica*
Haminoea japonica	KF572982	*Haminoea japonica*	correct	correct	correct	correct	correct	correct	*Haminoea japonica*
Haminoea japonica	KF572983	*Haminoea japonica*	correct	correct	correct	correct	correct	correct	*Haminoea japonica*
Haminoea japonica	JN830651	*Haminoea japonica*	correct	correct	correct	correct	correct	correct	*Haminoea japonica*
Haminoea japonica	KF572985	*Haminoea japonica*	correct	correct	correct	correct	correct	correct	*Haminoea japonica*
Haminoea japonica	KF572986	*Haminoea japonica*	correct	correct	correct	correct	correct	correct	*Haminoea japonica*
Haminoea japonica	KF572987	*Haminoea japonica*	correct	correct	correct	correct	correct	correct	*Haminoea japonica*
Haminoea japonica	KF572988	*Haminoea japonica*	correct	correct	correct	correct	correct	correct	*Haminoea japonica*
Haminoea japonica	JN830649	*Haminoea japonica*	correct	correct	correct	correct	correct	correct	*Haminoea japonica*
Haminoea japonica	JN830648	*Haminoea japonica*	correct	correct	correct	correct	correct	correct	*Haminoea japonica*
Haminoea japonica	JN830650	*Haminoea japonica*	no id	no id	no id	no id	no id	no id	*Haminoea japonica*
Haminoea japonica	KF572952	*Haminoea japonica*	correct	correct	correct	correct	correct	correct	*Haminoea japonica*
Haminoea japonica	KF572953	*Haminoea japonica*	correct	correct	correct	correct	correct	correct	*Haminoea japonica*
Haminoea japonica	KF572954	*Haminoea japonica*	correct	correct	correct	correct	correct	correct	*Haminoea japonica*
Haminoea japonica	KF572958	*Haminoea japonica*	correct	correct	correct	correct	correct	correct	*Haminoea japonica*

Haminoea japonica	KF572959	*Haminoea japonica*	correct	correct	correct	correct	correct	correct	*Haminoea japonica*
Haminoea japonica	KF572960	*Haminoea japonica*	correct	correct	correct	correct	correct	correct	*Haminoea japonica*
Haminoea japonica	KF572961	*Haminoea japonica*	correct	correct	correct	correct	correct	correct	*Haminoea japonica*
Haminoea japonica	KF572962	*Haminoea japonica*	correct	correct	correct	correct	correct	correct	*Haminoea japonica*
Haminoea japonica	KF572963	*Haminoea japonica*	correct	correct	correct	correct	correct	correct	*Haminoea japonica*
Haminoea japonica	KF572964	*Haminoea japonica*	correct	correct	correct	correct	correct	correct	*Haminoea japonica*
Haminoea japonica	KF572965	*Haminoea japonica*	correct	correct	correct	correct	correct	correct	*Haminoea japonica*
Haminoea japonica	KF572966	*Haminoea japonica*	correct	correct	correct	correct	correct	correct	*Haminoea japonica*
Haminoea japonica	KF572967	*Haminoea japonica*	correct	correct	correct	correct	correct	correct	*Haminoea japonica*
Haminoea japonica	KF572968	*Haminoea japonica*	correct	correct	correct	correct	correct	correct	*Haminoea japonica*
Haminoea japonica	KF572969	*Haminoea japonica*	correct	correct	correct	correct	correct	correct	*Haminoea japonica*
Haminoea japonica	KF572970	*Haminoea japonica*	no id	correct	correct	no id	correct	correct	*Haminoea japonica*
Haminoea japonica	KF572971	*Haminoea japonica*	correct	correct	correct	correct	correct	correct	*Haminoea japonica*
Haminoea japonica	KF572972	*Haminoea japonica*	correct	correct	correct	correct	correct	correct	*Haminoea japonica*
Haminoea japonica	KF572973	*Haminoea japonica*	correct	correct	correct	correct	correct	correct	*Haminoea japonica*
Haminoea japonica	KF572974	*Haminoea japonica*	correct	correct	correct	correct	correct	correct	*Haminoea japonica*
Haminoea japonica	KF572975	*Haminoea japonica*	correct	correct	correct	correct	correct	correct	*Haminoea japonica*
Haminoea japonica	KF572976	*Haminoea japonica*	correct	correct	correct	correct	correct	correct	*Haminoea japonica*
Haminoea japonica	KF572977	*Haminoea japonica*	correct	correct	correct	correct	correct	correct	*Haminoea japonica*
Haminoea japonica	KF572978	*Haminoea japonica*	correct	correct	correct	correct	correct	correct	*Haminoea japonica*
Haminoea japonica	KF572979	*Haminoea japonica*	correct	correct	correct	correct	correct	correct	*Haminoea japonica*
Haminoea japonica	KF572980	*Haminoea japonica*	correct	correct	correct	correct	correct	correct	*Haminoea japonica*
Haminoea japonica	KF572984	*Haminoea japonica*	correct	correct	correct	correct	correct	correct	*Haminoea japonica*
Hapalochlaena cf. maculosa	AB430529	*Hapalochlaena lunulata*	no id	no id	no id	no id	no id	no id	*Hapalochlaena fasciata*
Hapalochlaena fasciata	AB430530	*Amphioctopus ovulum*	no id	no id	no id	no id	no id	no id	*Hapalochlaena lunulata*
Hapalochlaena lunulata	HQ846163	*Gonatus madokai*	no id	no id	no id	no id	no id	no id	*Hapalochlaena fasciata*
Hazuregyra watanabei	HF586245	*Solariella nyssonus*	no id	no id	no id	no id	no id	no id	*Solariella nyssonus*
Hemifusus colosseus	HQ834068	*Hemifusus colosseus*	correct	correct	correct	correct	correct	correct	*Hemifusus sp.*
Hemifusus colosseus	JN053018	*Hemifusus colosseus*	correct	correct	correct	correct	correct	correct	*Hemifusus sp.*
Hemifusus ternatanus	HQ834067	*Hemifusus ternatanus*	correct	correct	correct	correct	correct	correct	*Hemifusus colosseus*
Hemifusus ternatanus	JN053017	*Hemifusus ternatanus*	correct	correct	correct	correct	correct	correct	*Hemifusus colosseus*

Hemifusus ternatanus	JN053016	*Hemifusus ternatanus*	correct	correct	correct	correct	correct	correct	*Hemifusus colosseus*
Hemifusus ternatanus	JN053015	*Hemifusus ternatanus*	correct	correct	correct	correct	correct	correct	*Hemifusus colosseus*
Hemifusus ternatanus	JN053014	*Hemifusus ternatanus*	correct	correct	correct	correct	correct	correct	*Hemifusus colosseus*
Hemifusus ternatanus	HM180607	*Hemifusus ternatanus*	correct	correct	correct	correct	correct	correct	*Hemifusus colosseus*
Hemifusus ternatanus	HM180608	*Hemifusus ternatanus*	correct	correct	correct	correct	correct	correct	*Hemifusus colosseus*
Hemifusus ternatanus	HM180609	*Hemifusus ternatanus*	correct	correct	correct	correct	correct	correct	*Hemifusus colosseus*
Hemifusus tuba	HQ834070	*Hemifusus tuba*	correct	correct	correct	correct	correct	correct	*Hemifusus ternatanus*
Hemifusus tuba	HQ834069	*Hemifusus tuba*	correct	correct	correct	correct	correct	correct	*Hemifusus ternatanus*
Hemifusus tuba	JN053024	*Hemifusus tuba*	correct	correct	correct	correct	correct	correct	*Hemifusus ternatanus*
Hemifusus tuba	JN053023	*Hemifusus tuba*	correct	correct	correct	correct	correct	correct	*Hemifusus ternatanus*
Hemifusus tuba	JN053022	*Hemifusus tuba*	correct	correct	correct	correct	correct	correct	*Hemifusus ternatanus*
Hemifusus tuba	JN053021	*Hemifusus tuba*	correct	correct	correct	correct	correct	correct	*Hemifusus ternatanus*
Hemifusus tuba	JN053020	*Hemifusus tuba*	correct	correct	correct	correct	correct	correct	*Hemifusus ternatanus*
Hemifusus tuba	JN053019	*Hemifusus tuba*	correct	correct	correct	correct	correct	correct	*Hemifusus ternatanus*
Herpetopoma pauperculus	AB365233	*Uroteuthis chinensis*	no id	no id	no id	no id	no id	no id	*Ginebis argenteonitens*
Heterololigo bleekeri	AB573761	*Heterololigo bleekeri*	correct	correct	correct	correct	correct	correct	*Loligo vulgaris*
Heterololigo bleekeri	AB573760	*Heterololigo bleekeri*	correct	correct	correct	correct	correct	correct	*Loligo vulgaris*
Heterololigo bleekeri	AB573759	*Heterololigo bleekeri*	correct	correct	correct	correct	correct	correct	*Loligo vulgaris*
Heterololigo bleekeri	AB573758	*Heterololigo bleekeri*	correct	correct	correct	correct	correct	correct	*Loligo vulgaris*
Heterololigo bleekeri	AB573757	*Heterololigo bleekeri*	correct	correct	correct	correct	correct	correct	*Loligo vulgaris*
Heterololigo bleekeri	AB573756	*Heterololigo bleekeri*	correct	correct	correct	correct	correct	correct	*Loligo vulgaris*
Heterololigo bleekeri	AB573755	*Heterololigo bleekeri*	correct	correct	correct	correct	correct	correct	*Loligo vulgaris*
Heterololigo bleekeri	AB573754	*Heterololigo bleekeri*	correct	correct	correct	correct	correct	correct	*Loligo vulgaris*
Heterololigo bleekeri	AB441190	*Heterololigo bleekeri*	correct	correct	correct	correct	correct	correct	*Loligo vulgaris*
Heterololigo bleekeri	AB441189	*Heterololigo bleekeri*	correct	correct	correct	correct	correct	correct	*Loligo vulgaris*
Heterololigo bleekeri	AB441188	*Heterololigo bleekeri*	correct	correct	correct	correct	correct	correct	*Loligo vulgaris*
Heterololigo bleekeri	AB441187	*Heterololigo bleekeri*	correct	correct	correct	correct	correct	correct	*Loligo vulgaris*
Heterololigo bleekeri	AB441185	*Heterololigo bleekeri*	correct	correct	correct	correct	correct	correct	*Loligo vulgaris*
Heterololigo bleekeri	AB441186	*Heterololigo bleekeri*	correct	correct	correct	correct	correct	correct	*Loligo vulgaris*
Heterololigo bleekeri	AB441184	*Heterololigo bleekeri*	correct	correct	correct	correct	correct	correct	*Loligo vulgaris*
Heterololigo bleekeri	AB441182	*Heterololigo bleekeri*	correct	correct	correct	correct	correct	correct	*Loligo vulgaris*

Heterololigo bleekeri	AB441181	*Heterololigo bleekeri*	no id	correct	correct	no id	correct	correct	*Loligo vulgaris*
Heterololigo bleekeri	AB441180	*Heterololigo bleekeri*	no id	correct	correct	no id	correct	correct	*Loligo vulgaris*
Heterololigo bleekeri	AB441179	*Heterololigo bleekeri*	correct	correct	correct	correct	correct	correct	*Loligo vulgaris*
Homalopoma granuliferum	AM049347	*Homalopoma granuliferum*	correct	correct	correct	correct	correct	correct	*Turbo sp.*
Homalopoma granuliferum	AB365217	*Homalopoma granuliferum*	correct	correct	correct	correct	correct	correct	*Turbo sp.*
Homalopoma nocturnum	AM049348	*Homalopoma sangarense*	no id	incorrect	incorrect	no id	incorrect	incorrect	*Homalopoma sangarense*
Homalopoma sangarense	AM049350	*Homalopoma nocturnum*	no id	incorrect	incorrect	no id	incorrect	incorrect	*Homalopoma nocturnum*
Brachidontes mutalilis	GQ480306	*Hormomya mutalilis*	correct	correct	correct	correct	correct	correct	*Hormomya exustus*
Brachidontes mutalilis	GQ480307	*Hormomya mutalilis*	no id	correct	correct	no id	correct	correct	*Hormomya exustus*
Brachidontes mutalilis	GQ480308	*Hormomya mutalilis*	correct	correct	correct	correct	correct	correct	*Hormomya exustus*
Brachidontes mutalilis	GQ480309	*Hormomya mutalilis*	correct	correct	correct	correct	correct	correct	*Hormomya exustus*
Brachidontes mutalilis	GQ480310	*Hormomya mutalilis*	no id	correct	correct	no id	correct	correct	*Hormomya exustus*
Idasola japonica	AB257537	*Idasola japonica*	correct	correct	correct	correct	correct	correct	*Idas sp.*
Idasola japonica	AB257536	*Idasola japonica*	correct	correct	correct	correct	correct	correct	*Idas sp.*
Idiosepius biserialis	EU008955	*Idiosepius paradoxus*	ambiguous	ambiguous	ambiguous	ambiguous	ambiguous	ambiguous	*Idiosepius macrocheir*
Idiosepius biserialis	EU008954	*Idiosepius paradoxus*	ambiguous	ambiguous	ambiguous	ambiguous	ambiguous	ambiguous	*Idiosepius macrocheir*
Idiosepius biserialis	EU008953	*Idiosepius paradoxus*	ambiguous	ambiguous	ambiguous	ambiguous	ambiguous	ambiguous	*Idiosepius macrocheir*
Idiosepius paradoxus	EU008997	*Idiosepius paradoxus*	correct	correct	correct	correct	ambiguous	ambiguous	*Idiosepius macrocheir*
Idiosepius paradoxus	EU008996	*Idiosepius paradoxus*	correct	correct	correct	correct	ambiguous	ambiguous	*Idiosepius macrocheir*
Idiosepius paradoxus	EU008995	*Idiosepius biserialis*	ambiguous	ambiguous	ambiguous	ambiguous	ambiguous	ambiguous	*Idiosepius macrocheir*
Idiosepius paradoxus	EU008994	*Idiosepius paradoxus*	correct	correct	correct	correct	ambiguous	ambiguous	*Idiosepius macrocheir*
Idiosepius paradoxus	EU008993	*Idiosepius paradoxus*	correct	correct	correct	correct	ambiguous	ambiguous	*Idiosepius macrocheir*
Idiosepius paradoxus	EU008992	*Idiosepius paradoxus*	correct	correct	correct	correct	ambiguous	ambiguous	*Idiosepius macrocheir*
Idiosepius paradoxus	EU008991	*Idiosepius biserialis*	ambiguous	ambiguous	ambiguous	ambiguous	ambiguous	ambiguous	*Idiosepius macrocheir*
Idiosepius paradoxus	EU008990	*Idiosepius paradoxus*	correct	correct	correct	correct	ambiguous	ambiguous	*Idiosepius macrocheir*
Idiosepius paradoxus	EU008989	*Idiosepius paradoxus*	correct	correct	correct	correct	ambiguous	ambiguous	*Idiosepius macrocheir*
Idiosepius paradoxus	EU008988	*Idiosepius paradoxus*	correct	correct	correct	correct	ambiguous	ambiguous	*Idiosepius macrocheir*
Idiosepius paradoxus	EU008985	*Idiosepius paradoxus*	correct	correct	correct	correct	ambiguous	ambiguous	*Idiosepius macrocheir*
Idiosepius paradoxus	EU008984	*Idiosepius paradoxus*	correct	correct	correct	correct	ambiguous	ambiguous	*Idiosepius macrocheir*
Idiosepius paradoxus	EU008983	*Idiosepius paradoxus*	correct	correct	correct	correct	ambiguous	ambiguous	*Idiosepius macrocheir*
Idiosepius paradoxus	EU008982	*Idiosepius paradoxus*	correct	correct	correct	correct	ambiguous	ambiguous	*Idiosepius macrocheir*

Idiosepius paradoxus	EU008981	*Idiosepius biserialis*	ambiguous	ambiguous	ambiguous	ambiguous	ambiguous	ambiguous	*Idiosepius macrocheir*
Idiosepius paradoxus	EU008980	*Idiosepius paradoxus*	correct	correct	correct	correct	ambiguous	ambiguous	*Idiosepius macrocheir*
Idiosepius paradoxus	EU008979	*Idiosepius paradoxus*	correct	correct	correct	correct	ambiguous	ambiguous	*Idiosepius macrocheir*
Idiosepius paradoxus	EU008987	*Idiosepius paradoxus*	correct	correct	correct	correct	correct	correct	*Idiosepius biserialis*
Idiosepius paradoxus	EU008986	*Idiosepius paradoxus*	correct	correct	correct	correct	correct	correct	*Idiosepius biserialis*
Isognomon acutirostris	AB076926	*Isognomon nucleus*	no id	incorrect	incorrect	no id	incorrect	incorrect	*Isognomon nucleus*
Isognomon nucleus	KU341970	*Isognomon acutirostris*	correct	correct	correct	correct	correct	correct	*Isognomon nucleus*
Isognomon ephippium	KU341971	*Isognomon ephippium*	no id	incorrect	incorrect	no id	incorrect	incorrect	*Isognomon sp.*
Isognomon ephippium	KU341972	*Isognomon ephippium*	correct	correct	correct	correct	correct	correct	*Isognomon sp.*
Isognomon ephippium	KU341973	*Isognomon ephippium*	correct	correct	correct	correct	correct	correct	*Isognomon sp.*
Isognomon ephippium	KU341974	*Isognomon ephippium*	correct	correct	correct	correct	correct	correct	*Isognomon sp.*
Isognomon ephippium	KU341975	*Isognomon ephippium*	no id	correct	correct	no id	correct	correct	*Isognomon sp.*
Isognomon legumen	KU341965	*Isognomon legumen*	correct	correct	correct	correct	correct	correct	*Isognomon ephippium*
Isognomon legumen	KU341966	*Isognomon legumen*	correct	correct	correct	correct	correct	correct	*Isognomon ephippium*
Isognomon legumen	KU341967	*Isognomon legumen*	correct	correct	correct	correct	correct	correct	*Isognomon ephippium*
Isognomon legumen	KU341968	*Isognomon legumen*	correct	correct	correct	correct	correct	correct	*Isognomon ephippium*
Isognomon legumen	KU341969	*Isognomon legumen*	correct	correct	correct	correct	correct	correct	*Isognomon ephippium*
Isognomon perna	KU341963	*Isognomon perna*	no id	no id	no id	no id	no id	no id	*Isognomon legumen*
Isognomon perna	KU341964	*Isognomon perna*	correct	correct	correct	correct	correct	correct	*Isognomon legumen*
Isognomon perna	AB076918	*Isognomon perna*	correct	correct	correct	correct	correct	correct	*Isognomon legumen*
Kelletia lischkei	HM180632	*Kelletia lischkei*	correct	correct	correct	correct	correct	correct	*Fasciolariidae sp.*
Kelletia lischkei	HM180633	*Kelletia lischkei*	correct	correct	correct	correct	correct	correct	*Fasciolariidae sp.*
Kelletia lischkei	HM180634	*Kelletia lischkei*	correct	correct	correct	correct	correct	correct	*Fasciolariidae sp.*
Kelletia lischkei	HM180635	*Kelletia lischkei*	correct	correct	correct	correct	correct	correct	*Fasciolariidae sp.*
Kelletia lischkei	HM180636	*Kelletia lischkei*	correct	correct	correct	correct	correct	correct	*Fasciolariidae sp.*
Lambis lambis	JF693383	*Lambis lambis*	correct	correct	correct	correct	correct	correct	*Lambis truncata*
Lambis lambis	JF693384	*Lambis lambis*	correct	correct	correct	correct	correct	correct	*Lambis truncata*
Lambis lambis	JF693385	*Lambis lambis*	correct	correct	correct	correct	correct	correct	*Lambis truncata*
Lepeta caeca	AB543978	*Lepeta caeca*	no id	correct	correct	no id	correct	correct	*Limalepeta lima*
Lepeta caeca	AB238458	*Lepeta caeca*	no id	correct	correct	no id	correct	correct	*Limalepeta lima*
Lepetodrilus nux	AB820805	*Lepetodrilus nux*	correct	correct	correct	correct	correct	correct	*Lepetodrilus sp.*

Lepetodrilus nux	AB820806	*Lepetodrilus nux*	correct	correct	correct	correct	correct	correct	*Lepetodrilus sp.*
Lepetodrilus nux	AB820807	*Lepetodrilus nux*	correct	correct	correct	correct	correct	correct	*Lepetodrilus sp.*
Lepetodrilus nux	AB820808	*Lepetodrilus nux*	correct	correct	correct	correct	correct	correct	*Lepetodrilus sp.*
Lepetodrilus nux	AB820809	*Lepetodrilus nux*	correct	correct	correct	correct	correct	correct	*Lepetodrilus sp.*
Lepetodrilus nux	AB820810	*Lepetodrilus nux*	correct	correct	correct	correct	correct	correct	*Lepetodrilus sp.*
Lepetodrilus nux	AB820811	*Lepetodrilus nux*	correct	correct	correct	correct	correct	correct	*Lepetodrilus sp.*
Lepetodrilus nux	AB820812	*Lepetodrilus nux*	correct	correct	correct	correct	correct	correct	*Lepetodrilus sp.*
Lepetodrilus nux	AB820813	*Lepetodrilus nux*	correct	correct	correct	correct	correct	correct	*Lepetodrilus sp.*
Lepetodrilus nux	AB820814	*Lepetodrilus nux*	correct	correct	correct	correct	correct	correct	*Lepetodrilus sp.*
Lepetodrilus nux	AB820815	*Lepetodrilus nux*	no id	correct	correct	no id	correct	correct	*Lepetodrilus sp.*
Lepetodrilus nux	AB820816	*Lepetodrilus nux*	correct	correct	correct	correct	correct	correct	*Lepetodrilus sp.*
Lepetodrilus nux	AB820817	*Lepetodrilus nux*	correct	correct	correct	correct	correct	correct	*Lepetodrilus sp.*
Lepetodrilus nux	AB820818	*Lepetodrilus nux*	correct	correct	correct	correct	correct	correct	*Lepetodrilus sp.*
Lepetodrilus nux	AB820819	*Lepetodrilus nux*	correct	correct	correct	correct	correct	correct	*Lepetodrilus sp.*
Lepetodrilus nux	AB820820	*Lepetodrilus nux*	correct	correct	correct	correct	correct	correct	*Lepetodrilus sp.*
Lepetodrilus nux	AB820821	*Lepetodrilus nux*	no id	correct	correct	no id	correct	correct	*Lepetodrilus sp.*
Lepetodrilus nux	AB820822	*Lepetodrilus nux*	correct	correct	correct	correct	correct	correct	*Lepetodrilus sp.*
Lepetodrilus nux	AB820823	*Lepetodrilus nux*	no id	correct	correct	no id	correct	correct	*Lepetodrilus sp.*
Lepetodrilus nux	AB820824	*Lepetodrilus nux*	correct	correct	correct	correct	correct	correct	*Lepetodrilus sp.*
Lepetodrilus nux	AB820825	*Lepetodrilus nux*	correct	correct	correct	correct	correct	correct	*Lepetodrilus sp.*
Lepetodrilus nux	AB820826	*Lepetodrilus nux*	correct	correct	correct	correct	correct	correct	*Lepetodrilus sp.*
Lepetodrilus nux	AB820827	*Lepetodrilus nux*	no id	correct	correct	no id	correct	correct	*Lepetodrilus sp.*
Lepetodrilus nux	AB820828	*Lepetodrilus nux*	correct	correct	correct	correct	correct	correct	*Lepetodrilus sp.*
Lepetodrilus nux	AB820829	*Lepetodrilus nux*	correct	correct	correct	correct	correct	correct	*Lepetodrilus sp.*
Lepetodrilus nux	AB820830	*Lepetodrilus nux*	correct	correct	correct	correct	correct	correct	*Lepetodrilus sp.*
Lepetodrilus nux	AB820831	*Lepetodrilus nux*	no id	correct	correct	no id	correct	correct	*Lepetodrilus sp.*
Lepetodrilus nux	AB820832	*Lepetodrilus nux*	no id	no id	correct	no id	no id	correct	*Lepetodrilus sp.*
Lepetodrilus nux	AB820833	*Lepetodrilus nux*	correct	correct	correct	correct	correct	correct	*Lepetodrilus sp.*
Lepetodrilus nux	AB820834	*Lepetodrilus nux*	correct	correct	correct	correct	correct	correct	*Lepetodrilus sp.*
Lepetodrilus nux	AB820835	*Lepetodrilus nux*	correct	correct	correct	correct	correct	correct	*Lepetodrilus sp.*
Lepetodrilus nux	AB820836	*Lepetodrilus nux*	correct	correct	correct	correct	correct	correct	*Lepetodrilus sp.*

Lepetodrilus nux	AB820837	*Lepetodrilus nux*	correct	correct	correct	correct	correct	correct	*Lepetodrilus sp.*
Lepetodrilus nux	AB820838	*Lepetodrilus nux*	correct	correct	correct	correct	correct	correct	*Lepetodrilus sp.*
Lepetodrilus nux	AB820839	*Lepetodrilus nux*	correct	correct	correct	correct	correct	correct	*Lepetodrilus sp.*
Limalepeta lima	AB543979	*Limalepeta lima*	no id	correct	correct	no id	correct	correct	*Lepeta caeca*
Limalepeta lima	AB543980	*Limalepeta lima*	no id	correct	correct	no id	correct	correct	*Lepeta caeca*
Limnoperna fortunei	AB828682	*Limnoperna fortunei*	correct	correct	correct	correct	correct	correct	*Modiolus kurilensis*
Limnoperna fortunei	AB828681	*Limnoperna fortunei*	correct	correct	correct	correct	correct	correct	*Modiolus kurilensis*
Limnoperna fortunei	AB828680	*Limnoperna fortunei*	no id	correct	correct	no id	correct	correct	*Modiolus kurilensis*
Limnoperna fortunei	AB828679	*Limnoperna fortunei*	correct	correct	correct	correct	correct	correct	*Modiolus kurilensis*
Limnoperna fortunei	AB520627	*Limnoperna fortunei*	correct	correct	correct	correct	correct	correct	*Modiolus kurilensis*
Limnoperna fortunei	AB520626	*Limnoperna fortunei*	correct	correct	correct	correct	correct	correct	*Modiolus kurilensis*
Limnoperna fortunei	AB520625	*Limnoperna fortunei*	correct	correct	correct	correct	correct	correct	*Modiolus kurilensis*
Limnoperna fortunei	AB520624	*Limnoperna fortunei*	correct	correct	correct	correct	correct	correct	*Modiolus kurilensis*
Limnoperna fortunei	AB520623	*Limnoperna fortunei*	correct	correct	correct	correct	correct	correct	*Modiolus kurilensis*
Limnoperna fortunei	AB520622	*Limnoperna fortunei*	no id	correct	correct	no id	correct	correct	*Modiolus kurilensis*
Limnoperna fortunei	AB520621	*Limnoperna fortunei*	correct	correct	correct	correct	correct	correct	*Modiolus kurilensis*
Limnoperna fortunei	AB520620	*Limnoperna fortunei*	correct	correct	correct	correct	correct	correct	*Modiolus kurilensis*
Limnoperna fortunei	AB520619	*Limnoperna fortunei*	no id	correct	correct	no id	correct	correct	*Modiolus kurilensis*
Limnoperna fortunei	AB520618	*Limnoperna fortunei*	correct	correct	correct	correct	correct	correct	*Modiolus kurilensis*
Limnoperna fortunei	AB520617	*Limnoperna fortunei*	correct	correct	correct	correct	correct	correct	*Modiolus kurilensis*
Limnoperna fortunei	AB520616	*Limnoperna fortunei*	correct	correct	correct	correct	correct	correct	*Modiolus kurilensis*
Limnoperna fortunei	AB520615	*Limnoperna fortunei*	correct	correct	correct	correct	correct	correct	*Modiolus kurilensis*
Limnoperna fortunei	AB520614	*Limnoperna fortunei*	no id	correct	correct	no id	correct	correct	*Modiolus kurilensis*
Limnoperna fortunei	AB520613	*Limnoperna fortunei*	no id	correct	correct	no id	correct	correct	*Modiolus kurilensis*
Limnoperna fortunei	AB520612	*Limnoperna fortunei*	no id	correct	correct	no id	correct	correct	*Modiolus kurilensis*
Lirularia iridescens	EU530125	*Lirularia iridescens*	correct	correct	correct	correct	correct	correct	*Lirularia pygmaea*
Lirularia iridescens	EU530124	*Lirularia iridescens*	correct	correct	correct	correct	correct	correct	*Lirularia pygmaea*
Lirularia pygmaea	AB505308	*Lirularia pygmaea*	correct	correct	correct	correct	correct	correct	*Lirularia iridescens*
Lirularia pygmaea	AB505309	*Lirularia pygmaea*	correct	correct	correct	correct	correct	correct	*Lirularia iridescens*
Littoraria coccinea	FN557093	*Littoraria intermedia*	no id	no id	no id	no id	no id	no id	*Littoraria lutea*
Littoraria intermedia	FN557104	*Littoraria scabra*	no id	no id	no id	no id	no id	no id	*Littorinidae sp.*

Littoraria intermedia	JF693389	*Clypeomorus traili*	correct	correct	correct	correct	correct	correct	*Littoraria articulata*
Littoraria intermedia	JF693390	*Littorinopsis intermedia*	correct	correct	correct	correct	correct	correct	*Littoraria articulata*
Littoraria melanostoma	HE590830	*Littoraria sinensis*	no id	no id	no id	no id	no id	no id	*Littoraria sinensis*
Littoraria pallescens	FN557122	*Littoraria pallescens*	correct	correct	correct	correct	correct	correct	*Littoraria pallescens*
Littoraria pallescens	AB611831	*Littoraria pallescens*	correct	correct	correct	correct	correct	correct	*Littoraria pallescens*
Littoraria pintado	AJ488634	*Littorinopsis intermedia*	no id	no id	no id	no id	no id	no id	*Littoraria intermedia*
Littoraria scabra	FN557135	*Littoraria intermedia*	no id	no id	no id	no id	no id	no id	*Littoraria angulifera*
Littoraria sinensis	FN557140	*Littoraria scabra*	no id	no id	no id	no id	no id	no id	*Littoraria melanostoma*
Littorina brevicula	JF693387	*Littorinopsis intermedia*	correct	correct	correct	correct	correct	correct	*Littorina mandshurica*
Littorina brevicula	JF693386	*Littorina brevicula*	correct	correct	correct	correct	correct	correct	*Littorina mandshurica*
Littorina brevicula	JF693388	*Littorina brevicula*	correct	correct	correct	correct	correct	correct	*Littorina mandshurica*
Littorina brevicula	HE590833	*Littorina brevicula*	correct	correct	correct	correct	correct	correct	*Littorina mandshurica*
Littorina horikawai	HE590836	*Littorina brevicula*	no id	no id	no id	no id	no id	no id	*Littorina sitkana*
Littorina kasatka	HE590837	*Littorina mandshurica*	no id	no id	no id	no id	no id	no id	*Littorina subrotundata*
Littorina mandshurica	HE590838	*Littorina brevicula*	no id	no id	incorrect	no id	no id	incorrect	*Littorina brevicula*
Littorina squalida	HE590843	*Littorina brevicula*	no id	no id	no id	no id	no id	no id	*Littorina littorea*
Littorinopsis scabra	JF693391	*Littorinopsis intermedia*	no id	no id	no id	no id	no id	no id	*Littoraria intermedia*
Loliolus beka	HQ529503	*Loliolus beka*	correct	correct	correct	correct	correct	correct	*Loliolus beka*
Loliolus beka	HQ529504	*Loliolus beka*	correct	correct	correct	correct	correct	correct	*Loliolus beka*
Loliolus beka	HQ529506	*Loliolus beka*	correct	correct	correct	correct	correct	correct	*Loliolus beka*
Loliolus beka	HQ529507	*Loliolus beka*	correct	correct	correct	correct	correct	correct	*Loliolus beka*
Loliolus beka	HQ529508	*Loliolus beka*	correct	correct	correct	correct	correct	correct	*Loliolus beka*
Loliolus beka	HQ529509	*Loliolus beka*	correct	correct	correct	correct	correct	correct	*Loliolus beka*
Loliolus beka	HQ529510	*Loliolus beka*	correct	correct	correct	correct	correct	correct	*Loliolus beka*
Loliolus beka	HQ529502	*Loliolus beka*	no id	no id	correct	no id	no id	correct	*Loliolus beka*
Loliolus beka	HQ529505	*Loliolus beka*	correct	correct	correct	correct	correct	correct	*Loliolus beka*
Loliolus beka	HQ529511	*Loliolus beka*	correct	correct	correct	correct	correct	correct	*Loliolus beka*
Loliolus beka	HQ529512	*Loliolus beka*	no id	correct	correct	no id	correct	correct	*Loliolus beka*
Loliolus beka	HQ529513	*Loliolus beka*	correct	correct	correct	correct	correct	correct	*Loliolus beka*
Loliolus beka	HQ529514	*Loliolus beka*	correct	correct	correct	correct	correct	correct	*Loliolus beka*
Loliolus beka	HQ529515	*Loliolus beka*	correct	correct	correct	correct	correct	correct	*Loliolus beka*

Loliolus beka	HQ529516	*Loliolus beka*	correct	correct	correct	correct	correct	correct	*Loliolus beka*
Loliolus japonica	HQ529517	*Loliolus japonicus*	correct	correct	correct	correct	correct	correct	*Loliolus beka*
Loliolus japonica	HQ529518	*Loliolus japonicus*	correct	correct	correct	correct	correct	correct	*Loliolus beka*
Loliolus japonica	HQ529519	*Loliolus japonicus*	correct	correct	correct	correct	correct	correct	*Loliolus beka*
Loliolus japonica	HQ529520	*Loliolus japonicus*	correct	correct	correct	correct	correct	correct	*Loliolus beka*
Loliolus japonica	HQ529521	*Loliolus japonicus*	correct	correct	correct	correct	correct	correct	*Loliolus beka*
Loliolus japonica	HQ529522	*Loliolus japonicus*	correct	correct	correct	correct	correct	correct	*Loliolus beka*
Loliolus uyii	HQ529524	*Loliolus uyii*	correct	correct	correct	correct	correct	correct	*Loliolus beka*
Loliolus uyii	HQ529523	*Loliolus uyii*	correct	correct	correct	correct	correct	correct	*Loliolus beka*
Loliolus uyii	HQ529525	*Loliolus uyii*	correct	correct	correct	correct	correct	correct	*Loliolus beka*
Loliolus uyii	HQ529526	*Loliolus uyii*	correct	correct	correct	correct	correct	correct	*Loliolus beka*
Loliolus uyii	HQ529527	*Loliolus uyii*	correct	correct	correct	correct	correct	correct	*Loliolus beka*
Lottia cassis	KM221034	*Lottia cassis*	correct	correct	correct	correct	correct	correct	*Lottia cassis*
Lottia cassis	KM221036	*Lottia cassis*	correct	correct	correct	correct	correct	correct	*Lottia cassis*
Lottia cassis	KM221042	*Lottia cassis*	correct	correct	correct	correct	correct	correct	*Lottia cassis*
Lottia cassis	KM221043	*Lottia cassis*	correct	correct	correct	correct	correct	correct	*Lottia cassis*
Lottia cassis	KM221115	*Lottia cassis*	correct	correct	correct	correct	correct	correct	*Lottia cassis*
Lottia cassis	KM221116	*Lottia cassis*	correct	correct	correct	correct	correct	correct	*Lottia cassis*
Lottia dorsuosa	KM221054	*Lottia dorsuosa*	correct	correct	correct	correct	correct	correct	*Lottia luchuana*
Lottia dorsuosa	KM221109	*Lottia dorsuosa*	correct	correct	correct	correct	correct	correct	*Lottia luchuana*
Lottia dorsuosa	KM221108	*Lottia dorsuosa*	correct	correct	correct	correct	correct	correct	*Lottia luchuana*
Lottia kogamogai	AB238467	*Lottia cassis*	no id	no id	no id	no id	no id	no id	*Lottia digitalis*
Lottia langfordi	AB238468	*Nipponacmea nigrans*	no id	no id	no id	no id	no id	no id	*Lottia sp.*
Lottia lindbergi	AB238470	*Lottia cassis*	no id	no id	no id	no id	no id	no id	*Lottia luchuana*
Lottia luchuana	KM221056	*Lottia luchuana*	correct	correct	correct	correct	correct	correct	*Lottia luchuana*
Lottia luchuana	KM221094	*Lottia luchuana*	correct	correct	correct	correct	correct	correct	*Lottia luchuana*
Lottia luchuana	KM221044	*Lottia luchuana*	correct	correct	correct	correct	correct	correct	*Lottia luchuana*
Lottia luchuana	KM221045	*Lottia luchuana*	correct	correct	correct	correct	correct	correct	*Lottia luchuana*
Lottia luchuana	KM221048	*Lottia luchuana*	correct	correct	correct	correct	correct	correct	*Lottia luchuana*
Lottia luchuana	KM221049	*Lottia luchuana*	correct	correct	correct	correct	correct	correct	*Lottia luchuana*
Lottia luchuana	KM221101	*Lottia luchuana*	correct	correct	correct	correct	correct	correct	*Lottia luchuana*

Lottia luchuana	AB238471	*Lottia luchuana*	correct	correct	correct	correct	correct	correct	*Lottia luchuana*
Lottia luchuana	KM221100	*Lottia luchuana*	no id	correct	correct	no id	correct	correct	*Lottia luchuana*
Lottia tenuisculpta	AB238482	*Lottia luchuana*	no id	no id	no id	no id	no id	no id	*Lottia septiformis*
Lunella cinerea	AB588872	*Lunella cinerea*	correct	correct	correct	correct	correct	correct	*Lunella cinerea*
Lunella cinerea	AB297735	*Lunella cinerea*	correct	correct	correct	correct	correct	correct	*Lunella cinerea*
Lunella cinerea	AB297734	*Lunella cinerea*	correct	correct	correct	correct	correct	correct	*Lunella cinerea*
Lunella cinerea	AB297733	*Lunella cinerea*	correct	correct	correct	correct	correct	correct	*Lunella cinerea*
Lunella coreensis	HQ681192	*Lunella coreensis*	ambiguous	ambiguous	ambiguous	ambiguous	ambiguous	ambiguous	*Lunella coronata*
Lunella coreensis	HQ681191	*Lunella coreensis*	ambiguous	ambiguous	ambiguous	ambiguous	ambiguous	ambiguous	*Lunella coronata*
Lunella coreensis	AM403861	*Lunella coreensis*	ambiguous	ambiguous	ambiguous	ambiguous	ambiguous	ambiguous	*Lunella coronata*
Lunella coreensis	AM403860	*Lunella coreensis*	ambiguous	ambiguous	ambiguous	ambiguous	ambiguous	ambiguous	*Lunella coronata*
Lunella coreensis	AB297732	*Lunella coreensis*	ambiguous	ambiguous	ambiguous	ambiguous	ambiguous	ambiguous	*Lunella coronata*
Lunella coreensis	AB297731	*Lunella coreensis*	ambiguous	ambiguous	ambiguous	ambiguous	ambiguous	ambiguous	*Lunella coronata*
Lunella coreensis	AB297730	*Lunella coreensis*	ambiguous	ambiguous	ambiguous	ambiguous	ambiguous	ambiguous	*Lunella coronata*
Lunella coreensis	HM180657	*Lunella coreensis*	ambiguous	ambiguous	ambiguous	ambiguous	ambiguous	ambiguous	*Lunella coronata*
Lunella coreensis	HM180658	*Lunella coreensis*	ambiguous	ambiguous	ambiguous	ambiguous	ambiguous	ambiguous	*Lunella coronata*
Lunella coreensis	HM180659	*Lunella coreensis*	ambiguous	ambiguous	ambiguous	ambiguous	ambiguous	ambiguous	*Lunella coronata*
Lunella moniliformis	AB588878	*Lunella coreensis*	ambiguous	ambiguous	ambiguous	ambiguous	ambiguous	ambiguous	*Lunella coronata*
Lunella moniliformis	AB588877	*Lunella coreensis*	ambiguous	ambiguous	ambiguous	ambiguous	ambiguous	ambiguous	*Lunella coronata*
Lunella moniliformis	AB588876	*Lunella coreensis*	ambiguous	ambiguous	ambiguous	ambiguous	ambiguous	ambiguous	*Lunella coronata*
Lunella coronata	AB297729	*Lunella granulata*	ambiguous	ambiguous	ambiguous	ambiguous	ambiguous	ambiguous	*Lunella ogasawarana*
Lunella coronata	AB297728	*Lunella granulata*	ambiguous	ambiguous	ambiguous	ambiguous	ambiguous	ambiguous	*Lunella ogasawarana*
Lunella coronata	AB297727	*Lunella granulata*	ambiguous	ambiguous	ambiguous	ambiguous	ambiguous	ambiguous	*Lunella ogasawarana*
Lunella granulata	AM403863	*Lunella coronata*	ambiguous	ambiguous	ambiguous	ambiguous	ambiguous	ambiguous	*Lunella ogasawarana*
Lunella granulata	AB588892	*Lunella coronata*	ambiguous	ambiguous	ambiguous	ambiguous	ambiguous	ambiguous	*Lunella ogasawarana*
Lunella granulata	AB588891	*Lunella coronata*	ambiguous	ambiguous	ambiguous	ambiguous	ambiguous	ambiguous	*Lunella ogasawarana*
Lunella granulata	AM403862	*Lunella coronata*	ambiguous	ambiguous	ambiguous	ambiguous	ambiguous	ambiguous	*Lunella ogasawarana*
Lunella ogasawarana	AB588883	*Lunella ogasawarana*	correct	correct	correct	correct	correct	correct	*Lunella coronata*
Lunella ogasawarana	AB588882	*Lunella ogasawarana*	correct	correct	correct	correct	correct	correct	*Lunella coronata*
Lunella ogasawarana	AB588881	*Lunella ogasawarana*	correct	correct	correct	correct	correct	correct	*Lunella coronata*
Lunella ogasawarana	AB588880	*Lunella ogasawarana*	correct	correct	correct	correct	correct	correct	*Lunella coronata*

Lunella ogasawarana	AB588879	*Lunella ogasawarana*	correct	correct	correct	correct	correct	correct	*Lunella coronata*
Lunella ogasawarana	AB297723	*Lunella ogasawarana*	correct	correct	correct	correct	correct	correct	*Lunella coronata*
Lunella ogasawarana	AB297725	*Lunella ogasawarana*	correct	correct	correct	correct	correct	correct	*Lunella coronata*
Lunella ogasawarana	AB297724	*Lunella ogasawarana*	correct	correct	correct	correct	correct	correct	*Lunella coronata*
Lunella ogasawarana	AB297726	*Lunella ogasawarana*	correct	correct	correct	correct	correct	correct	*Lunella coronata*
Lunella ogasawarana	AB297722	*Lunella ogasawarana*	correct	correct	correct	correct	correct	correct	*Lunella coronata*
Lutraria arcuata	JN674601	*Lutraria arcuata*	correct	correct	correct	correct	correct	correct	*Lutraria australis*
Lutraria arcuata	JN674602	*Lutraria arcuata*	correct	correct	correct	correct	correct	correct	*Lutraria australis*
Lutraria arcuata	JN674603	*Lutraria arcuata*	correct	correct	correct	correct	correct	correct	*Lutraria australis*
Lutraria australis	JN674600	*Lutraria arcuata*	no id	no id	no id	no id	no id	no id	*Lutraria sp.*
Macoma candida	JN859965	*Macoma candida*	no id	no id	no id	no id	no id	no id	*Moerella iridescens*
Macoma tokyoensis	JN859962	*Macoma tokyoensis*	correct	correct	correct	correct	correct	correct	*Macoma nasuta*
Macoma tokyoensis	JN859963	*Macoma tokyoensis*	correct	correct	correct	correct	correct	correct	*Macoma nasuta*
Macridiscus multifarius	HQ224672	*Macridiscus aequilatera*	ambiguous	ambiguous	ambiguous	ambiguous	ambiguous	ambiguous	*Macridiscus semicancellata*
Macridiscus multifarius	HQ224673	*Macridiscus aequilatera*	ambiguous	ambiguous	ambiguous	ambiguous	ambiguous	ambiguous	*Macridiscus semicancellata*
Macridiscus multifarius	HQ224674	*Macridiscus aequilatera*	ambiguous	ambiguous	ambiguous	ambiguous	ambiguous	ambiguous	*Macridiscus semicancellata*
Macridiscus multifarius	HQ224675	*Macridiscus aequilatera*	no id	ambiguous	ambiguous	no id	ambiguous	ambiguous	*Macridiscus semicancellata*
Macridiscus multifarius	HQ224676	*Macridiscus aequilatera*	no id	ambiguous	ambiguous	no id	ambiguous	ambiguous	*Macridiscus semicancellata*
Macridiscus multifarius	HQ224677	*Macridiscus aequilatera*	ambiguous	ambiguous	ambiguous	ambiguous	ambiguous	ambiguous	*Macridiscus semicancellata*
Macridiscus multifarius	HQ224678	*Macridiscus aequilatera*	ambiguous	ambiguous	ambiguous	ambiguous	ambiguous	ambiguous	*Macridiscus semicancellata*
Macridiscus multifarius	HQ224679	*Macridiscus aequilatera*	no id	ambiguous	ambiguous	no id	ambiguous	ambiguous	*Macridiscus semicancellata*
Macridiscus aequilatera	HQ703266	*Macridiscus aequilatera*	correct	correct	correct	correct	correct	correct	*Macridiscus semicancellata*
Macridiscus aequilatera	HQ703267	*Macridiscus aequilatera*	ambiguous	ambiguous	ambiguous	ambiguous	ambiguous	ambiguous	*Macridiscus semicancellata*
Macridiscus aequilatera	HQ703268	*Macridiscus aequilatera*	ambiguous	ambiguous	ambiguous	ambiguous	ambiguous	ambiguous	*Macridiscus semicancellata*
Macridiscus aequilatera	HQ703269	*Macridiscus aequilatera*	ambiguous	ambiguous	ambiguous	ambiguous	ambiguous	ambiguous	*Macridiscus semicancellata*
Macridiscus aequilatera	HQ703270	*Macridiscus aequilatera*	ambiguous	ambiguous	ambiguous	ambiguous	ambiguous	ambiguous	*Macridiscus semicancellata*
Macridiscus aequilatera	HQ703271	*Macridiscus aequilatera*	ambiguous	ambiguous	ambiguous	ambiguous	ambiguous	ambiguous	*Macridiscus semicancellata*
Macridiscus aequilatera	HQ703272	*Macridiscus aequilatera*	ambiguous	ambiguous	ambiguous	ambiguous	ambiguous	ambiguous	*Macridiscus semicancellata*
Macridiscus aequilatera	GQ855272	*Macridiscus semicancellata*	ambiguous	ambiguous	ambiguous	ambiguous	ambiguous	ambiguous	*Macridiscus multifarius*
Macridiscus aequilatera	GQ855273	*Macridiscus semicancellata*	incorrect	incorrect	incorrect	incorrect	ambiguous	ambiguous	*Macridiscus multifarius*
Macridiscus aequilatera	GQ855274	*Macridiscus semicancellata*	ambiguous	ambiguous	ambiguous	ambiguous	ambiguous	ambiguous	*Macridiscus multifarius*

Macridiscus aequilatera	GQ855275	*Macridiscus semicancellata*	ambiguous	ambiguous	ambiguous	ambiguous	ambiguous	ambiguous	*Macridiscus multifarius*
Macridiscus aequilatera	GQ855276	*Macridiscus semicancellata*	ambiguous	ambiguous	ambiguous	ambiguous	ambiguous	ambiguous	*Macridiscus multifarius*
Macridiscus semicancellata	HQ703275	*Macridiscus semicancellata*	correct	correct	correct	correct	correct	correct	*Macridiscus multifarius*
Macridiscus semicancellata	HQ703276	*Macridiscus aequilatera*	ambiguous	ambiguous	ambiguous	ambiguous	ambiguous	ambiguous	*Macridiscus multifarius*
Macridiscus semicancellata	HQ703277	*Macridiscus aequilatera*	ambiguous	ambiguous	ambiguous	ambiguous	ambiguous	ambiguous	*Macridiscus multifarius*
Macridiscus semicancellata	HQ703278	*Macridiscus semicancellata*	ambiguous	ambiguous	ambiguous	ambiguous	ambiguous	ambiguous	*Macridiscus multifarius*
Macridiscus semicancellata	HQ703279	*Macridiscus aequilatera*	ambiguous	ambiguous	ambiguous	ambiguous	ambiguous	ambiguous	*Macridiscus multifarius*
Macridiscus semicancellata	HQ703280	*Macridiscus aequilatera*	ambiguous	ambiguous	ambiguous	ambiguous	ambiguous	ambiguous	*Macridiscus multifarius*
Macridiscus semicancellata	HQ703281	*Macridiscus semicancellata*	ambiguous	ambiguous	ambiguous	ambiguous	ambiguous	ambiguous	*Macridiscus multifarius*
Macridiscus semicancellata	HQ224686	*Macridiscus semicancellata*	ambiguous	ambiguous	ambiguous	ambiguous	ambiguous	ambiguous	*Macridiscus multifarius*
Macridiscus semicancellata	HQ224687	*Macridiscus semicancellata*	correct	correct	correct	correct	ambiguous	ambiguous	*Macridiscus multifarius*
Macridiscus semicancellata	HQ224688	*Macridiscus semicancellata*	ambiguous	ambiguous	ambiguous	ambiguous	ambiguous	ambiguous	*Macridiscus multifarius*
Macridiscus semicancellata	HQ224689	*Macridiscus semicancellata*	correct	correct	correct	correct	ambiguous	ambiguous	*Macridiscus multifarius*
Macridiscus semicancellata	HQ224690	*Macridiscus semicancellata*	ambiguous	ambiguous	ambiguous	ambiguous	ambiguous	ambiguous	*Macridiscus multifarius*
Macridiscus semicancellata	HQ224692	*Macridiscus semicancellata*	ambiguous	ambiguous	ambiguous	ambiguous	ambiguous	ambiguous	*Macridiscus multifarius*
Macridiscus semicancellata	HQ224693	*Macridiscus semicancellata*	ambiguous	ambiguous	ambiguous	ambiguous	ambiguous	ambiguous	*Macridiscus multifarius*
Macridiscus semicancellata	HQ224694	*Macridiscus aequilatera*	ambiguous	ambiguous	ambiguous	ambiguous	ambiguous	ambiguous	*Macridiscus multifarius*
Macridiscus melanaegis	HQ703273	*Macridiscus melanaegis*	ambiguous	ambiguous	ambiguous	ambiguous	ambiguous	ambiguous	*Macridiscus semicancellata*
Macridiscus melanaegis	HQ703274	*Macridiscus melanaegis*	correct	correct	correct	correct	correct	correct	*Macridiscus semicancellata*
Macroschisma dilatata	AM049334	*Macroschisma dilatata*	no id	no id	correct	no id	no id	correct	*Macroschisma dilatata*
Macroschisma dilatata	AB365212	*Macroschisma dilatata*	no id	no id	correct	no id	no id	correct	*Macroschisma dilatata*
Mactra alta	JN674615	*Mactra alta*	correct	correct	correct	correct	correct	correct	*Mactra veneriformis*
Mactra alta	JN674616	*Mactra alta*	correct	correct	correct	correct	correct	correct	*Mactra veneriformis*
Mactra alta	JN674617	*Mactra alta*	correct	correct	correct	correct	correct	correct	*Mactra veneriformis*
Mactra alta	JN674618	*Mactra alta*	correct	correct	correct	correct	correct	correct	*Mactra veneriformis*
Mactra alta	JN674619	*Mactra alta*	correct	correct	correct	correct	correct	correct	*Mactra veneriformis*
Mactra alta	JN674620	*Mactra alta*	correct	correct	correct	correct	correct	correct	*Mactra veneriformis*
Mactra chinensis	JN674630	*Mactra chinensis*	no id	correct	correct	no id	correct	correct	*Mactra chinensis*
Mactra chinensis	JN674631	*Mactra chinensis*	correct	correct	correct	correct	correct	correct	*Mactra chinensis*
Mactra chinensis	JN674632	*Mactra chinensis*	correct	correct	correct	correct	correct	correct	*Mactra chinensis*
Mactra chinensis	JN674633	*Mactra chinensis*	correct	correct	correct	correct	correct	correct	*Mactra chinensis*

Mactra chinensis	JN674634	*Mactra chinensis*	correct	correct	correct	correct	correct	correct	*Mactra chinensis*
Mactra chinensis	JN674635	*Mactra chinensis*	correct	correct	correct	correct	correct	correct	*Mactra chinensis*
Mactra chinensis	KC205870	*Mactra chinensis*	correct	correct	correct	correct	correct	correct	*Mactra chinensis*
Mactra chinensis	KC205871	*Mactra chinensis*	correct	correct	correct	correct	correct	correct	*Mactra chinensis*
Mactra chinensis	KC205872	*Mactra chinensis*	correct	correct	correct	correct	correct	correct	*Mactra chinensis*
Mactra chinensis	KC205873	*Mactra chinensis*	no id	correct	correct	no id	correct	correct	*Mactra chinensis*
Mactra chinensis	KC205874	*Mactra chinensis*	correct	correct	correct	correct	correct	correct	*Mactra chinensis*
Mactra chinensis	KC205875	*Mactra chinensis*	correct	correct	correct	correct	correct	correct	*Mactra chinensis*
Mactra chinensis	KC205876	*Mactra chinensis*	correct	correct	correct	correct	correct	correct	*Mactra chinensis*
Mactra chinensis	KC205877	*Mactra chinensis*	correct	correct	correct	correct	correct	correct	*Mactra chinensis*
Mactra mauclata	JN674613	*Mactra maculata*	correct	correct	correct	correct	correct	correct	*Mactra stultorum*
Mactra mauclata	JN674614	*Mactra maculata*	correct	correct	correct	correct	correct	correct	*Mactra stultorum*
Mancinella echinata	HE584343	*Mancinella siro*	no id	no id	no id	no id	no id	no id	*Mancinella alouina*
Mancinella siro	HE584344	*Strombus marginatus robustus*	no id	no id	no id	no id	no id	no id	*Thais echinulatus*
Katelysia hiantiana	GQ855255	*Katelysia hiantiana*	ambiguous	ambiguous	ambiguous	ambiguous	ambiguous	ambiguous	*Marcia japonica*
Katelysia hiantiana	GQ855256	*Katelysia hiantiana*	ambiguous	ambiguous	ambiguous	ambiguous	ambiguous	ambiguous	*Marcia japonica*
Katelysia hiantiana	GQ855257	*Katelysia hiantiana*	ambiguous	ambiguous	ambiguous	ambiguous	ambiguous	ambiguous	*Marcia japonica*
Katelysia hiantiana	HQ703287	*Katelysia hiantiana*	correct	correct	correct	correct	correct	correct	*Marcia japonica*
Katelysia hiantiana	HQ703288	*Katelysia hiantiana*	ambiguous	ambiguous	ambiguous	ambiguous	ambiguous	ambiguous	*Marcia japonica*
Katelysia hiantiana	HQ703289	*Katelysia hiantiana*	ambiguous	ambiguous	ambiguous	ambiguous	ambiguous	ambiguous	*Marcia japonica*
Katelysia hiantiana	HQ703290	*Katelysia hiantiana*	ambiguous	ambiguous	ambiguous	ambiguous	ambiguous	ambiguous	*Marcia japonica*
Katelysia hiantiana	HQ703291	*Katelysia hiantiana*	ambiguous	ambiguous	ambiguous	ambiguous	ambiguous	ambiguous	*Marcia japonica*
Katelysia hiantiana	HQ703292	*Katelysia hiantiana*	ambiguous	ambiguous	ambiguous	ambiguous	ambiguous	ambiguous	*Marcia japonica*
Katelysia hiantiana	HQ703293	*Katelysia hiantiana*	ambiguous	ambiguous	ambiguous	ambiguous	ambiguous	ambiguous	*Marcia japonica*
Katelysia hiantiana	HQ703294	*Katelysia hiantiana*	ambiguous	ambiguous	ambiguous	ambiguous	ambiguous	ambiguous	*Marcia japonica*
Katelysia hiantiana	JN898939	*Katelysia hiantiana*	no id	ambiguous	ambiguous	no id	ambiguous	ambiguous	*Marcia japonica*
Katelysia hiantiana	HM124599	*Katelysia hiantiana*	incorrect	incorrect	incorrect	incorrect	ambiguous	ambiguous	*Marcia japonica*
Marcia japonica	HQ703282	*Marcia japonica*	ambiguous	ambiguous	ambiguous	ambiguous	ambiguous	ambiguous	*Katelysia hiantina*
Marcia japonica	HQ703283	*Marcia japonica*	correct	correct	correct	correct	correct	correct	*Katelysia hiantina*
Marcia japonica	HQ703284	*Marcia japonica*	correct	correct	correct	correct	correct	correct	*Katelysia hiantina*
Marcia japonica	HQ703285	*Marcia japonica*	correct	correct	correct	correct	correct	correct	*Katelysia hiantina*

Marcia japonica	HQ703286	*Marcia japonica*	correct	correct	correct	correct	correct	correct	*Katelysia hiantina*
Marcia japonica	GQ855258	*Marcia japonica*	correct	correct	correct	correct	correct	correct	*Katelysia hiantina*
Marcia japonica	GQ855259	*Marcia japonica*	correct	correct	correct	correct	correct	correct	*Katelysia hiantina*
Marcia japonica	GQ855260	*Marcia japonica*	correct	correct	correct	correct	correct	correct	*Katelysia hiantina*
Marcia japonica	GQ855261	*Marcia japonica*	correct	correct	correct	correct	correct	correct	*Katelysia hiantina*
Marcia japonica	GQ855262	*Marcia japonica*	correct	correct	correct	correct	correct	correct	*Katelysia hiantina*
Marcia marmorata	HQ703296	*Marcia marmorata*	correct	correct	correct	correct	correct	correct	*Tapes dorsatus*
Marcia marmorata	HQ703297	*Marcia marmorata*	correct	correct	correct	correct	correct	correct	*Tapes dorsatus*
Marcia marmorata	HQ703298	*Marcia marmorata*	correct	correct	correct	correct	correct	correct	*Tapes dorsatus*
Marcia marmorata	HQ703299	*Marcia marmorata*	correct	correct	correct	correct	correct	correct	*Tapes dorsatus*
Marcia marmorata	HQ703300	*Marcia marmorata*	correct	correct	correct	correct	correct	correct	*Tapes dorsatus*
Marcia marmorata	HQ703301	*Marcia marmorata*	no id	correct	correct	no id	correct	correct	*Tapes dorsatus*
Marcia marmorata	HQ703302	*Marcia marmorata*	correct	correct	correct	correct	correct	correct	*Tapes dorsatus*
Marcia marmorata	HQ703303	*Marcia marmorata*	correct	correct	correct	correct	correct	correct	*Tapes dorsatus*
Martesia striata	KJ125424	*Martesia striata*	correct	correct	correct	correct	correct	correct	*Mollusca sp.*
Martesia striata	KJ125425	*Martesia striata*	correct	correct	correct	correct	correct	correct	*Mollusca sp.*
Mauritia arabica	JF693392	*Mauritia arabica*	no id	no id	correct	no id	no id	correct	*Mauritia arabica*
Mauritia arabica	JF693393	*Mauritia arabica*	correct	correct	correct	correct	correct	correct	*Mauritia arabica*
Mauritia arabica	JF693394	*Mauritia arabica*	correct	correct	correct	correct	correct	correct	*Mauritia arabica*
Mauritia arabica	JF693395	*Mauritia arabica*	correct	correct	correct	correct	correct	correct	*Mauritia arabica*
Melanochlamys ezoensis	KJ704899	*Melanochlamys ezoensis*	correct	correct	correct	correct	correct	correct	*Chelidonura berolina*
Melanochlamys ezoensis	KJ704900	*Melanochlamys ezoensis*	correct	correct	correct	correct	correct	correct	*Chelidonura berolina*
Melanochlamys ezoensis	KJ704901	*Melanochlamys ezoensis*	correct	correct	correct	correct	correct	correct	*Chelidonura berolina*
Melanochlamys ezoensis	KJ704902	*Melanochlamys ezoensis*	correct	correct	correct	correct	correct	correct	*Chelidonura berolina*
Melanochlamys ezoensis	KJ704903	*Melanochlamys ezoensis*	no id	correct	correct	no id	correct	correct	*Chelidonura berolina*
Melanochlamys ezoensis	KJ704904	*Melanochlamys ezoensis*	correct	correct	correct	correct	correct	correct	*Chelidonura berolina*
Melanochlamys ezoensis	KJ704905	*Melanochlamys ezoensis*	correct	correct	correct	correct	correct	correct	*Chelidonura berolina*
Melanochlamys fukudai	KJ704906	*Melanochlamys fukudai*	correct	correct	correct	correct	correct	correct	*Melanochlamys ezoensis*
Melanochlamys fukudai	KJ704907	*Melanochlamys fukudai*	correct	correct	correct	correct	correct	correct	*Melanochlamys ezoensis*
Melanochlamys fukudai	KJ704908	*Melanochlamys fukudai*	correct	correct	correct	correct	correct	correct	*Melanochlamys ezoensis*
Melanochlamys fukudai	KJ704909	*Melanochlamys fukudai*	correct	correct	correct	correct	correct	correct	*Melanochlamys ezoensis*

Melanochlamys fukudai	KJ704910	*Melanochlamys fukudai*	correct	correct	correct	correct	correct	correct	*Melanochlamys ezoensis*
Melanochlamys fukudai	KJ704911	*Melanochlamys fukudai*	correct	correct	correct	correct	correct	correct	*Melanochlamys ezoensis*
Melanochlamys fukudai	KJ704912	*Melanochlamys fukudai*	correct	correct	correct	correct	correct	correct	*Melanochlamys ezoensis*
Melanochlamys fukudai	KJ704913	*Melanochlamys fukudai*	correct	correct	correct	correct	correct	correct	*Melanochlamys ezoensis*
Melanochlamys fukudai	KJ704914	*Melanochlamys fukudai*	correct	correct	correct	correct	correct	correct	*Melanochlamys ezoensis*
Melanochlamys fukudai	KJ704915	*Melanochlamys fukudai*	correct	correct	correct	correct	correct	correct	*Melanochlamys ezoensis*
Melanochlamys fukudai	KJ704916	*Melanochlamys fukudai*	correct	correct	correct	correct	correct	correct	*Melanochlamys ezoensis*
Melanochlamys fukudai	KJ704917	*Melanochlamys fukudai*	correct	correct	correct	correct	correct	correct	*Melanochlamys ezoensis*
Melanochlamys fukudai	KJ704918	*Melanochlamys fukudai*	correct	correct	correct	correct	correct	correct	*Melanochlamys ezoensis*
Melanochlamys fukudai	KJ704919	*Melanochlamys fukudai*	correct	correct	correct	correct	correct	correct	*Melanochlamys ezoensis*
Melanochlamys fukudai	KJ704920	*Melanochlamys fukudai*	correct	correct	correct	correct	correct	correct	*Melanochlamys ezoensis*
Melanochlamys fukudai	KJ704921	*Melanochlamys fukudai*	correct	correct	correct	correct	correct	correct	*Melanochlamys ezoensis*
Melanochlamys fukudai	KJ704922	*Melanochlamys fukudai*	correct	correct	correct	correct	correct	correct	*Melanochlamys ezoensis*
Melanochlamys fukudai	KJ704923	*Melanochlamys fukudai*	correct	correct	correct	correct	correct	correct	*Melanochlamys ezoensis*
Melanochlamys fukudai	KJ704924	*Melanochlamys fukudai*	correct	correct	correct	correct	correct	correct	*Melanochlamys ezoensis*
Melanochlamys fukudai	KJ704925	*Melanochlamys fukudai*	correct	correct	correct	correct	correct	correct	*Melanochlamys ezoensis*
Melanochlamys fukudai	KJ704926	*Melanochlamys fukudai*	no id	correct	correct	no id	correct	correct	*Melanochlamys ezoensis*
Melanochlamys fukudai	KJ704927	*Melanochlamys fukudai*	correct	correct	correct	correct	correct	correct	*Melanochlamys ezoensis*
Melanochlamys fukudai	KJ704928	*Melanochlamys fukudai*	correct	correct	correct	correct	correct	correct	*Melanochlamys ezoensis*
Melanochlamys fukudai	KJ704929	*Melanochlamys fukudai*	correct	correct	correct	correct	correct	correct	*Melanochlamys ezoensis*
Melanochlamys fukudai	KJ704930	*Melanochlamys fukudai*	correct	correct	correct	correct	correct	correct	*Melanochlamys ezoensis*
Melanochlamys kohi	KJ704933	*Melanochlamys kohi*	no id	correct	correct	no id	correct	correct	*Melanochlamys sp.*
Melanochlamys kohi	KJ704934	*Melanochlamys kohi*	no id	correct	correct	no id	correct	correct	*Melanochlamys sp.*
Melanochlamys kohi	KJ704935	*Melanochlamys kohi*	no id	correct	correct	no id	correct	correct	*Melanochlamys sp.*
Melo melo	JN053026	*Melo melo*	correct	correct	correct	correct	correct	correct	*Cymbiola vespertilio*
Melo melo	HQ834086	*Melo melo*	correct	correct	correct	correct	correct	correct	*Cymbiola vespertilio*
Melo melo	HQ834085	*Melo melo*	correct	correct	correct	correct	correct	correct	*Cymbiola vespertilio*
Melo melo	JN053027	*Melo melo*	correct	correct	correct	correct	correct	correct	*Cymbiola vespertilio*
Mercenaria mercenaria	HQ703071	*Mercenaria mercenaria*	no id	correct	correct	no id	correct	correct	*Humilaria kennerleyi*
Mercenaria mercenaria	HQ703072	*Mercenaria mercenaria*	correct	correct	correct	correct	correct	correct	*Humilaria kennerleyi*
Mercenaria mercenaria	HQ703073	*Mercenaria mercenaria*	correct	correct	correct	correct	correct	correct	*Humilaria kennerleyi*

Mercenaria mercenaria	HQ703074	*Mercenaria mercenaria*	correct	correct	correct	correct	correct	correct	*Humilaria kennerleyi*
Mercenaria mercenaria	HQ703075	*Mercenaria mercenaria*	correct	correct	correct	correct	correct	correct	*Humilaria kennerleyi*
Mercenaria mercenaria	HQ703076	*Mercenaria mercenaria*	correct	correct	correct	correct	correct	correct	*Humilaria kennerleyi*
Mercenaria mercenaria	JN898949	*Mercenaria mercenaria*	correct	correct	correct	correct	correct	correct	*Humilaria kennerleyi*
Mercenaria mercenaria	HM124578	*Mercenaria mercenaria*	correct	correct	correct	correct	correct	correct	*Humilaria kennerleyi*
Mercenaria mercenaria	DQ399398	*Mercenaria mercenaria*	correct	correct	correct	correct	correct	correct	*Humilaria kennerleyi*
Mercenaria mercenaria	JN043623	*Mercenaria mercenaria*	correct	correct	correct	correct	correct	correct	*Humilaria kennerleyi*
Mercenaria mercenaria	AB685212	*Mercenaria mercenaria*	correct	correct	correct	correct	correct	correct	*Humilaria kennerleyi*
Mercenaria mercenaria	AB685211	*Mercenaria mercenaria*	correct	correct	correct	correct	correct	correct	*Humilaria kennerleyi*
Mercenaria mercenaria	AB685210	*Mercenaria mercenaria*	correct	correct	correct	correct	correct	correct	*Humilaria kennerleyi*
Mercenaria mercenaria	AB685209	*Mercenaria mercenaria*	correct	correct	correct	correct	correct	correct	*Humilaria kennerleyi*
Mercenaria mercenaria	AB685208	*Mercenaria mercenaria*	correct	correct	correct	correct	correct	correct	*Humilaria kennerleyi*
Mercenaria mercenaria	AB685207	*Mercenaria mercenaria*	correct	correct	correct	correct	correct	correct	*Humilaria kennerleyi*
Mercenaria mercenaria	AB685206	*Mercenaria mercenaria*	no id	correct	correct	no id	correct	correct	*Humilaria kennerleyi*
Mercenaria mercenaria	AB685205	*Mercenaria mercenaria*	correct	correct	correct	correct	correct	correct	*Humilaria kennerleyi*
Mercenaria mercenaria	AB685204	*Mercenaria mercenaria*	correct	correct	correct	correct	correct	correct	*Humilaria kennerleyi*
Mercenaria mercenaria	AB685203	*Mercenaria mercenaria*	no id	correct	correct	no id	correct	correct	*Humilaria kennerleyi*
Mercenaria mercenaria	AB685202	*Mercenaria mercenaria*	no id	correct	correct	no id	correct	correct	*Humilaria kennerleyi*
Mercenaria mercenaria	AB685201	*Mercenaria mercenaria*	correct	correct	correct	correct	correct	correct	*Humilaria kennerleyi*
Mercenaria mercenaria	AB685200	*Mercenaria mercenaria*	correct	correct	correct	correct	correct	correct	*Humilaria kennerleyi*
Mercenaria mercenaria	AB685199	*Mercenaria mercenaria*	correct	correct	correct	correct	correct	correct	*Humilaria kennerleyi*
Mercenaria mercenaria	AB685198	*Mercenaria mercenaria*	correct	correct	correct	correct	correct	correct	*Humilaria kennerleyi*
Mercenaria mercenaria	AB685197	*Mercenaria mercenaria*	correct	correct	correct	correct	correct	correct	*Humilaria kennerleyi*
Mercenaria mercenaria	AB685196	*Mercenaria mercenaria*	correct	correct	correct	correct	correct	correct	*Humilaria kennerleyi*
Mercenaria mercenaria	AB685195	*Mercenaria mercenaria*	correct	correct	correct	correct	correct	correct	*Humilaria kennerleyi*
Mercenaria mercenaria	AB685194	*Mercenaria mercenaria*	correct	correct	correct	correct	correct	correct	*Humilaria kennerleyi*
Mercenaria mercenaria	AB685193	*Mercenaria mercenaria*	correct	correct	correct	correct	correct	correct	*Humilaria kennerleyi*
Mercenaria mercenaria	AB670734	*Mercenaria mercenaria*	no id	correct	correct	no id	correct	correct	*Humilaria kennerleyi*
Mercenaria mercenaria	AB670733	*Mercenaria mercenaria*	correct	correct	correct	correct	correct	correct	*Humilaria kennerleyi*
Mercenaria mercenaria	AB670732	*Mercenaria mercenaria*	correct	correct	correct	correct	correct	correct	*Humilaria kennerleyi*
Mercenaria mercenaria	AB670731	*Mercenaria mercenaria*	correct	correct	correct	correct	correct	correct	*Humilaria kennerleyi*

Mercenaria mercenaria	AB670730	*Mercenaria mercenaria*	correct	correct	correct	correct	correct	correct	*Humilaria kennerleyi*
Mercenaria mercenaria	AB670729	*Mercenaria mercenaria*	correct	correct	correct	correct	correct	correct	*Humilaria kennerleyi*
Mercenaria mercenaria	AB670728	*Mercenaria mercenaria*	correct	correct	correct	correct	correct	correct	*Humilaria kennerleyi*
Mercenaria mercenaria	AB670727	*Mercenaria mercenaria*	correct	correct	correct	correct	correct	correct	*Humilaria kennerleyi*
Mercenaria mercenaria	AB670726	*Mercenaria mercenaria*	correct	correct	correct	correct	correct	correct	*Humilaria kennerleyi*
Mercenaria mercenaria	AB670725	*Mercenaria mercenaria*	correct	correct	correct	correct	correct	correct	*Humilaria kennerleyi*
Mercenaria mercenaria	AB670724	*Mercenaria mercenaria*	correct	correct	correct	correct	correct	correct	*Humilaria kennerleyi*
Mercenaria mercenaria	AB670723	*Mercenaria mercenaria*	correct	correct	correct	correct	correct	correct	*Humilaria kennerleyi*
Mercenaria mercenaria	AB670722	*Mercenaria mercenaria*	correct	correct	correct	correct	correct	correct	*Humilaria kennerleyi*
Mercenaria mercenaria	AB670721	*Mercenaria mercenaria*	correct	correct	correct	correct	correct	correct	*Humilaria kennerleyi*
Mercenaria mercenaria	AB670720	*Mercenaria mercenaria*	correct	correct	correct	correct	correct	correct	*Humilaria kennerleyi*
Mercenaria mercenaria	AB670719	*Mercenaria mercenaria*	correct	correct	correct	correct	correct	correct	*Humilaria kennerleyi*
Meretrix lamarckii	HQ703188	*Meretrix lamarckii*	ambiguous	ambiguous	ambiguous	ambiguous	ambiguous	ambiguous	*Meretrix lamarckii*
Meretrix lamarckii	HQ703189	*Meretrix lamarckii*	correct	correct	correct	correct	correct	correct	*Meretrix lamarckii*
Meretrix lamarckii	HQ703190	*Meretrix lamarckii*	correct	correct	correct	correct	correct	correct	*Meretrix lamarckii*
Meretrix lamarckii	HQ703191	*Meretrix lamarckii*	correct	correct	correct	correct	correct	correct	*Meretrix lamarckii*
Meretrix lamarckii	HM124579	*Meretrix lamarckii*	correct	correct	correct	correct	correct	correct	*Meretrix lamarckii*
Meretrix lamarckii	JN043625	*Meretrix lamarckii*	correct	correct	correct	correct	correct	correct	*Meretrix lamarckii*
Meretrix lyrata	HQ703192	*Meretrix lyrata*	correct	correct	correct	correct	correct	correct	*Meretrix lyrata*
Meretrix lyrata	HQ703193	*Meretrix lyrata*	correct	correct	correct	correct	correct	correct	*Meretrix lyrata*
Meretrix lyrata	HQ703194	*Meretrix lyrata*	correct	correct	correct	correct	correct	correct	*Meretrix lyrata*
Meretrix lyrata	HQ703195	*Meretrix lyrata*	correct	correct	correct	correct	correct	correct	*Meretrix lyrata*
Meretrix lyrata	HQ703196	*Meretrix lyrata*	correct	correct	correct	correct	correct	correct	*Meretrix lyrata*
Meretrix lyrata	HQ703197	*Meretrix lyrata*	correct	correct	correct	correct	correct	correct	*Meretrix lyrata*
Meretrix lyrata	HQ703198	*Meretrix lyrata*	correct	correct	correct	correct	correct	correct	*Meretrix lyrata*
Meretrix lyrata	JN043622	*Meretrix lyrata*	no id	correct	correct	no id	correct	correct	*Meretrix lyrata*
Meretrix lyrata	JN898944	*Meretrix lyrata*	correct	correct	correct	correct	correct	correct	*Meretrix lyrata*
Meretrix lyrata	HM124581	*Meretrix lyrata*	correct	correct	correct	correct	correct	correct	*Meretrix lyrata*
Meretrix lusoria	JN043624	*Meretrix petechialis*	incorrect	incorrect	incorrect	incorrect	ambiguous	ambiguous	*Meretrix petechialis*
Meretrix lusoria	JN898936	*Meretrix petechialis*	ambiguous	ambiguous	ambiguous	ambiguous	ambiguous	ambiguous	*Meretrix petechialis*
Meretrix lusoria	JN898935	*Meretrix petechialis*	ambiguous	ambiguous	ambiguous	ambiguous	ambiguous	ambiguous	*Meretrix petechialis*

Meretrix meretrix	DQ399399	*Meretrix petechialis*	incorrect	incorrect	incorrect	incorrect	incorrect	incorrect	*Meretrix petechialis*
Meretrix meretrix	HQ703159	*Meretrix meretrix*	correct	correct	correct	correct	correct	correct	*Meretrix lyrata*
Meretrix meretrix	HQ703160	*Meretrix meretrix*	correct	correct	correct	correct	correct	correct	*Meretrix lyrata*
Meretrix meretrix	HQ703161	*Meretrix meretrix*	correct	correct	correct	correct	correct	correct	*Meretrix lyrata*
Meretrix meretrix	HQ703162	*Meretrix meretrix*	correct	correct	correct	correct	correct	correct	*Meretrix lyrata*
Meretrix meretrix	HQ703163	*Meretrix meretrix*	correct	correct	correct	correct	correct	correct	*Meretrix lyrata*
Meretrix meretrix	HM124619	*Meretrix meretrix*	correct	correct	correct	correct	correct	correct	*Meretrix lyrata*
Meretrix meretrix	DQ399403	*Meretrix meretrix*	correct	correct	correct	correct	correct	correct	*Meretrix lyrata*
Meretrix meretrix	HM124618	*Meretrix petechialis*	ambiguous	ambiguous	ambiguous	ambiguous	ambiguous	ambiguous	*Meretrix lusoria*
Meretrix meretrix	JN898950	*Meretrix petechialis*	ambiguous	ambiguous	ambiguous	ambiguous	ambiguous	ambiguous	*Meretrix lusoria*
Meretrix meretrix	DQ399400	*Meretrix petechialis*	ambiguous	ambiguous	ambiguous	ambiguous	ambiguous	ambiguous	*Meretrix lusoria*
Meretrix meretrix	DQ399401	*Meretrix petechialis*	ambiguous	ambiguous	ambiguous	ambiguous	ambiguous	ambiguous	*Meretrix lusoria*
Meretrix meretrix	DQ399402	*Meretrix petechialis*	ambiguous	ambiguous	ambiguous	ambiguous	ambiguous	ambiguous	*Meretrix lusoria*
Meretrix petechialis	HQ703164	*Meretrix petechialis*	correct	correct	correct	correct	correct	correct	*Meretrix lusoria*
Meretrix petechialis	HQ703165	*Meretrix petechialis*	ambiguous	ambiguous	ambiguous	ambiguous	ambiguous	ambiguous	*Meretrix lusoria*
Meretrix petechialis	HQ703166	*Meretrix petechialis*	ambiguous	ambiguous	ambiguous	ambiguous	ambiguous	ambiguous	*Meretrix lusoria*
Meretrix petechialis	HQ703167	*Meretrix petechialis*	ambiguous	ambiguous	ambiguous	ambiguous	ambiguous	ambiguous	*Meretrix lusoria*
Meretrix petechialis	HQ703168	*Meretrix petechialis*	ambiguous	ambiguous	ambiguous	ambiguous	ambiguous	ambiguous	*Meretrix lusoria*
Meretrix petechialis	HQ703169	*Meretrix petechialis*	no id	ambiguous	ambiguous	no id	ambiguous	ambiguous	*Meretrix lusoria*
Meretrix petechialis	HQ703170	*Meretrix petechialis*	ambiguous	ambiguous	ambiguous	ambiguous	ambiguous	ambiguous	*Meretrix lusoria*
Meretrix petechialis	HQ703171	*Meretrix petechialis*	ambiguous	ambiguous	ambiguous	ambiguous	ambiguous	ambiguous	*Meretrix lusoria*
Meretrix petechialis	HQ703172	*Meretrix petechialis*	ambiguous	ambiguous	ambiguous	ambiguous	ambiguous	ambiguous	*Meretrix lusoria*
Meretrix petechialis	AB853869	*Meretrix petechialis*	ambiguous	ambiguous	ambiguous	ambiguous	ambiguous	ambiguous	*Meretrix lusoria*
Meretrix petechialis	HM124582	*Meretrix petechialis*	ambiguous	ambiguous	ambiguous	ambiguous	ambiguous	ambiguous	*Meretrix lusoria*
Meretrix petechialis	HQ703173	*Meretrix petechialis*	ambiguous	ambiguous	ambiguous	ambiguous	ambiguous	ambiguous	*Meretrix petechialis*
Meretrix petechialis	HQ703174	*Meretrix petechialis*	ambiguous	ambiguous	ambiguous	ambiguous	ambiguous	ambiguous	*Meretrix petechialis*
Meretrix petechialis	HQ703175	*Meretrix petechialis*	ambiguous	ambiguous	ambiguous	ambiguous	ambiguous	ambiguous	*Meretrix petechialis*
Meretrix petechialis	HQ703176	*Meretrix petechialis*	ambiguous	ambiguous	ambiguous	ambiguous	ambiguous	ambiguous	*Meretrix petechialis*
Meretrix petechialis	HQ703177	*Meretrix petechialis*	ambiguous	ambiguous	ambiguous	ambiguous	ambiguous	ambiguous	*Meretrix petechialis*
Meretrix petechialis	HQ703178	*Meretrix petechialis*	ambiguous	ambiguous	ambiguous	ambiguous	ambiguous	ambiguous	*Meretrix petechialis*
Meretrix petechialis	HQ703179	*Meretrix petechialis*	no id	ambiguous	ambiguous	no id	ambiguous	ambiguous	*Meretrix petechialis*

Meretrix petechialis	HQ703180	*Meretrix petechialis*	ambiguous	ambiguous	ambiguous	ambiguous	ambiguous	ambiguous	*Meretrix petechialis*
Meretrix petechialis	HQ703181	*Meretrix lusoria*	ambiguous	ambiguous	ambiguous	ambiguous	ambiguous	ambiguous	*Meretrix petechialis*
Meretrix petechialis	HQ703182	*Meretrix lusoria*	ambiguous	ambiguous	ambiguous	ambiguous	ambiguous	ambiguous	*Meretrix petechialis*
Meretrix petechialis	HQ703183	*Meretrix petechialis*	ambiguous	ambiguous	ambiguous	ambiguous	ambiguous	ambiguous	*Meretrix petechialis*
Meretrix petechialis	HQ703184	*Meretrix petechialis*	ambiguous	ambiguous	ambiguous	ambiguous	ambiguous	ambiguous	*Meretrix petechialis*
Meretrix petechialis	HQ703185	*Meretrix petechialis*	ambiguous	ambiguous	ambiguous	ambiguous	ambiguous	ambiguous	*Meretrix petechialis*
Meretrix petechialis	HQ703186	*Meretrix petechialis*	ambiguous	ambiguous	ambiguous	ambiguous	ambiguous	ambiguous	*Meretrix petechialis*
Meretrix petechialis	HQ703187	*Meretrix petechialis*	ambiguous	ambiguous	ambiguous	ambiguous	ambiguous	ambiguous	*Meretrix petechialis*
Meretrix petechialis	HM124584	*Meretrix petechialis*	ambiguous	ambiguous	ambiguous	ambiguous	ambiguous	ambiguous	*Meretrix petechialis*
Meretrix petechialis	HM124583	*Meretrix petechialis*	ambiguous	ambiguous	ambiguous	ambiguous	ambiguous	ambiguous	*Meretrix petechialis*
Meretrix lusoria	FJ434681	*Meretrix petechialis*	no id	ambiguous	ambiguous	no id	ambiguous	ambiguous	*Meretrix lusoria*
Meretrix lusoria	AB076924	*Meretrix petechialis*	incorrect	incorrect	incorrect	incorrect	ambiguous	ambiguous	*Meretrix lusoria*
Meretrix lusoria	AB853864	*Meretrix lusoria*	correct	correct	correct	correct	correct	correct	*Meretrix petechialis*
Meretrix lusoria	AB853865	*Meretrix lusoria*	correct	correct	correct	correct	correct	correct	*Meretrix petechialis*
Meretrix lusoria	AB613023	*Meretrix lusoria*	correct	correct	correct	correct	correct	correct	*Meretrix petechialis*
Meretrix lusoria	AB613022	*Meretrix lusoria*	correct	correct	correct	correct	correct	correct	*Meretrix petechialis*
Meretrix lusoria	AB280786	*Meretrix lusoria*	correct	correct	correct	correct	correct	correct	*Meretrix petechialis*
Mikadotrochus beyrichii	AM049331	*Mikadotrochus beyrichii*	no id	correct	correct	no id	correct	correct	*Bayerotrochus africanus*
Mikadotrochus beyrichii	EU530109	*Mikadotrochus beyrichii*	no id	correct	correct	no id	correct	correct	*Bayerotrochus africanus*
Mimachlamys nobilis	GU119985	*Mimachlamys nobilis*	correct	correct	correct	correct	correct	correct	
Mimachlamys nobilis	GU119986	*Mimachlamys nobilis*	correct	correct	correct	correct	correct	correct	
Mimachlamys nobilis	GU119987	*Mimachlamys nobilis*	no id	correct	correct	no id	correct	correct	
Mimachlamys nobilis	GU119988	*Mimachlamys nobilis*	correct	correct	correct	correct	correct	correct	
Mimachlamys nobilis	GU119989	*Mimachlamys nobilis*	correct	correct	correct	correct	correct	correct	
Mitrella bicincta	HQ834055	*Mitrella bicincta*	correct	correct	correct	correct	correct	correct	*Amphissa versicolor*
Mitrella bicincta	JN052991	*Mitrella bicincta*	correct	correct	correct	correct	correct	correct	*Amphissa versicolor*
Mitrella bicincta	JN052990	*Mitrella bicincta*	correct	correct	correct	correct	correct	correct	*Amphissa versicolor*
Mitrella bicincta	JN052989	*Mitrella bicincta*	correct	correct	correct	correct	correct	correct	*Amphissa versicolor*
Mitrella bicincta	JN052988	*Mitrella bicincta*	correct	correct	correct	correct	correct	correct	*Amphissa versicolor*
Mitrella bicincta	HM180686	*Mitrella bicincta*	no id	correct	correct	no id	correct	correct	*Amphissa versicolor*
Mitrella bicincta	HM180689	*Mitrella bicincta*	no id	no id	correct	no id	no id	correct	*Amphissa versicolor*

Mitrella bicincta	HM180683	*Mitrella bicincta*	no id	ambiguous	ambiguous	no id	ambiguous	ambiguous	*Amphissa columbiana*
Mitrella bicincta	HM180684	*Mitrella bicincta*	correct	correct	correct	correct	correct	correct	*Amphissa columbiana*
Mitrella bicincta	HM180685	*Mitrella bicincta*	correct	correct	correct	correct	correct	correct	*Amphissa columbiana*
Mitrella bicincta	HM180687	*Mitrella bicincta*	ambiguous	ambiguous	ambiguous	ambiguous	ambiguous	ambiguous	*Amphissa columbiana*
Mitrella bicincta	HM180688	*Mitrella bicincta*	ambiguous	ambiguous	ambiguous	ambiguous	ambiguous	ambiguous	*Amphissa columbiana*
Mitrella bicincta	HM180690	*Mitrella bicincta*	ambiguous	ambiguous	ambiguous	ambiguous	ambiguous	ambiguous	*Amphissa columbiana*
Mitrella bicincta	HM180691	*Mitrella bicincta*	no id	no id	correct	no id	no id	correct	*Amphissa columbiana*
Mitrella bicincta	HM180692	*Mitrella bicincta*	ambiguous	ambiguous	ambiguous	ambiguous	ambiguous	ambiguous	*Amphissa columbiana*
Mitrella burchardi	HQ834098	*Mitrella bicincta*	incorrect	incorrect	incorrect	incorrect	ambiguous	ambiguous	*Amphissa columbiana*
Mitrella burchardi	JN053028	*Mitrella bicincta*	no id	ambiguous	ambiguous	no id	ambiguous	ambiguous	*Amphissa columbiana*
Mizuhopecten yessoensis	GU119990	*Mizuhopecten yessoensis*	no id	no id	correct	no id	no id	correct	
Mizuhopecten yessoensis	GU119991	*Mizuhopecten yessoensis*	no id	correct	correct	no id	correct	correct	
Mizuhopecten yessoensis	GU119992	*Mizuhopecten yessoensis*	correct	correct	correct	correct	correct	correct	
Mizuhopecten yessoensis	GU119993	*Mizuhopecten yessoensis*	correct	correct	correct	correct	correct	correct	
Mizuhopecten yessoensis	GU119994	*Mizuhopecten yessoensis*	correct	correct	correct	correct	correct	correct	
Mizuhopecten yessoensis	GU119995	*Mizuhopecten yessoensis*	no id	correct	correct	no id	correct	correct	
Mizuhopecten yessoensis	GU119996	*Mizuhopecten yessoensis*	no id	correct	correct	no id	correct	correct	
Mizuhopecten yessoensis	GU119997	*Mizuhopecten yessoensis*	no id	correct	correct	no id	correct	correct	
Modiolus auriculatus	GQ480317	*Adipicola crypta*	no id	no id	no id	no id	no id	no id	*Modiolus rumphii*
Modiolus comptus	GQ480313	*Modiolus comptus*	ambiguous	ambiguous	ambiguous	ambiguous	ambiguous	ambiguous	*Modiolus metcalfei*
Modiolus comptus	GQ480314	*Modiolus comptus*	ambiguous	ambiguous	ambiguous	ambiguous	ambiguous	ambiguous	*Modiolus metcalfei*
Modiolus comptus	GQ480315	*Modiolus comptus*	ambiguous	ambiguous	ambiguous	ambiguous	ambiguous	ambiguous	*Modiolus metcalfei*
Modiolus comptus	GQ480316	*Modiolus comptus*	ambiguous	ambiguous	ambiguous	ambiguous	ambiguous	ambiguous	*Modiolus metcalfei*
Modiolus nipponicus	AB076912	*Modiolus comptus*	incorrect	incorrect	incorrect	incorrect	incorrect	incorrect	*Modiolus metcalfei*
Modiolus elongatus	GQ480318	*Bathymodiolus septemdierum*	no id	no id	no id	no id	no id	no id	*Micragone tholloni*
Modiolus kurilensis	KP243079	*Modiolus kurilensis*	correct	correct	correct	correct	correct	correct	*Modiolus modiolus*
Modiolus kurilensis	KP243078	*Modiolus kurilensis*	correct	correct	correct	correct	correct	correct	*Modiolus modiolus*
Modiolus kurilensis	KP243077	*Modiolus kurilensis*	correct	correct	correct	correct	correct	correct	*Modiolus modiolus*
Modiolus kurilensis	KP243076	*Modiolus kurilensis*	correct	correct	correct	correct	correct	correct	*Modiolus modiolus*
Modiolus kurilensis	KP243075	*Modiolus kurilensis*	no id	correct	correct	no id	correct	correct	*Modiolus modiolus*
Modiolus kurilensis	KP243074	*Modiolus kurilensis*	correct	correct	correct	correct	correct	correct	*Modiolus modiolus*

Modiolus metcalfei	GQ480319	*Modiolus metcalfei*	no id	correct	correct	no id	correct	correct	*Modiolus rumphii*
Modiolus metcalfei	GQ480320	*Modiolus metcalfei*	correct	correct	correct	correct	correct	correct	*Modiolus rumphii*
Modiolus metcalfei	GQ480321	*Modiolus metcalfei*	correct	correct	correct	correct	correct	correct	*Modiolus rumphii*
Modiolus metcalfei	GQ480322	*Modiolus metcalfei*	correct	correct	correct	correct	correct	correct	*Modiolus rumphii*
Moerella iridescens	JN859970	*Moerella iridescens*	no id	no id	no id	no id	no id	no id	*Moerella iridescens*
Monilea smithi	AB505310	*Monilea smithi*	correct	correct	correct	correct	correct	correct	*Monilea lentiginosa*
Monilea smithi	AB505311	*Monilea smithi*	correct	correct	correct	correct	correct	correct	*Monilea lentiginosa*
Monodonta australis	HM180693	*Monodonta australis*	correct	correct	correct	correct	correct	correct	*Monodonta australis*
Monodonta australis	HM180696	*Monodonta australis*	correct	correct	correct	correct	correct	correct	*Monodonta australis*
Monodonta australis	HM180694	*Monodonta australis*	correct	correct	correct	correct	correct	correct	*Monodonta australis*
Monodonta australis	HM180695	*Monodonta australis*	correct	correct	correct	correct	correct	correct	*Monodonta australis*
Monodonta australis	HM180697	*Monodonta australis*	correct	correct	correct	correct	correct	correct	*Monodonta australis*
Monodonta australis	HM180698	*Monodonta australis*	correct	correct	correct	correct	correct	correct	*Monodonta australis*
Monodonta australis	HM180699	*Monodonta australis*	correct	correct	correct	correct	correct	correct	*Monodonta australis*
Monodonta canalifera	DQ061095	*Monodonta canalifera*	correct	correct	correct	correct	correct	correct	*Monodonta canalifera*
Monodonta canalifera	EU530128	*Monodonta canalifera*	correct	correct	correct	correct	correct	correct	*Monodonta canalifera*
Monodonta perplexa	DQ061096	*Clanculus margaritarius*	no id	no id	no id	no id	no id	no id	*Monodonta australis*
Morula funiculata	HE584047	*Morula funiculata*	correct	correct	correct	correct	correct	correct	*Orania pacifica*
Morula funiculata	HE584046	*Morula funiculata*	correct	correct	correct	correct	correct	correct	*Orania pacifica*
Morula funiculata	HE584045	*Morula funiculata*	correct	correct	correct	correct	correct	correct	*Orania pacifica*
Morula granulata	JN053032	*Morula granulata*	correct	correct	correct	correct	correct	correct	*Morula marginalba*
Morula granulata	JN053031	*Morula granulata*	correct	correct	correct	correct	correct	correct	*Morula marginalba*
Morula granulata	JN053030	*Morula granulata*	correct	correct	correct	correct	correct	correct	*Morula marginalba*
Morula granulata	JN053029	*Morula granulata*	correct	correct	correct	correct	correct	correct	*Morula marginalba*
Morula japonica	HE584023	*Littorina kasatka*	no id	no id	no id	no id	no id	no id	*Morula sp.*
Morula purpureocincta	HE584049	*Morula funiculata*	no id	no id	no id	no id	no id	no id	*Morula funiculata*
Morula rugosa	JN053034	*Morula rugosa*	correct	correct	correct	correct	correct	correct	*Morula anaxares*
Morula rugosa	JN053033	*Morula rugosa*	correct	correct	correct	correct	correct	correct	*Morula anaxares*
Morula rumphiusi	HE584019	*Morula zebrina*	no id	no id	incorrect	no id	no id	incorrect	*Morula rumphiusi*
Morula spinosa	HE584026	*Morula spinosa*	no id	correct	correct	no id	correct	correct	*Thais sp.*
Morula spinosa	HE584025	*Morula spinosa*	no id	correct	correct	no id	correct	correct	*Thais sp.*

Morula striata	HE584028	*Morula striata*	no id	correct	correct	no id	correct	correct	*Thais sp.*
Morula striata	HE584027	*Morula striata*	no id	correct	correct	no id	correct	correct	*Morula striata*
Morula zebrina	HE584033	*Morula rumphiusi*	no id	no id	incorrect	no id	no id	incorrect	*Morula rumphiusi*
Munditiella ammonoceras	AM049337	*Munditiella ammonoceras*	no id	correct	correct	no id	correct	correct	*Trochus maculatus*
Munditiella ammonoceras	AB365244	*Munditiella ammonoceras*	no id	correct	correct	no id	correct	correct	*Trochus maculatus*
Murex trapa	GU188195	*Murex trapa*	correct	correct	correct	correct	correct	correct	*Muricidae sp.*
Murex trapa	GU188196	*Murex trapa*	correct	correct	correct	correct	correct	correct	*Muricidae sp.*
Murex trapa	GU188197	*Murex trapa*	correct	correct	correct	correct	correct	correct	*Muricidae sp.*
Murex trapa	GU188198	*Murex trapa*	correct	correct	correct	correct	correct	correct	*Muricidae sp.*
Murex trapa	GU188199	*Murex trapa*	correct	correct	correct	correct	correct	correct	*Muricidae sp.*
Musculista senhousia	AB076942	*Musculista senhousia*	correct	correct	correct	correct	correct	correct	*Musculista senhousia*
Musculista senhousia	AB498016	*Musculista senhousia*	correct	correct	correct	correct	correct	correct	*Musculista senhousia*
Mya arenaria	KJ125420	*Mya arenaria*	no id	no id	no id	no id	no id	no id	*Veneroida sp.*
Mya arenaria	KJ125421	*Mya arenaria*	correct	correct	correct	correct	correct	correct	*Veneroida sp.*
Mytilus coruscus	GQ480287	*Mytilus galloprovincialis*	ambiguous	ambiguous	ambiguous	ambiguous	ambiguous	ambiguous	*Mytilus californianus*
Mytilus coruscus	GQ480288	*Mytilus galloprovincialis*	ambiguous	ambiguous	ambiguous	ambiguous	ambiguous	ambiguous	*Mytilus californianus*
Mytilus coruscus	GQ480289	*Mytilus galloprovincialis*	ambiguous	ambiguous	ambiguous	ambiguous	ambiguous	ambiguous	*Mytilus californianus*
Mytilus coruscus	GQ480290	*Mytilus galloprovincialis*	ambiguous	ambiguous	ambiguous	ambiguous	ambiguous	ambiguous	*Mytilus californianus*
Mytilus coruscus	GQ480291	*Mytilus galloprovincialis*	ambiguous	ambiguous	ambiguous	ambiguous	ambiguous	ambiguous	*Mytilus californianus*
Mytilus coruscus	GQ480295	*Mytilus galloprovincialis*	ambiguous	ambiguous	ambiguous	ambiguous	ambiguous	ambiguous	*Mytilus californianus*
Mytilus coruscus	GQ480283	*Mytilus galloprovincialis*	ambiguous	ambiguous	ambiguous	ambiguous	ambiguous	ambiguous	*Mytilus californianus*
Mytilus galloprovincialis	HM180705	*Mytilus coruscus*	ambiguous	ambiguous	ambiguous	ambiguous	ambiguous	ambiguous	*Mytilus californianus*
Mytilus galloprovincialis	HM180706	*Mytilus coruscus*	ambiguous	ambiguous	ambiguous	ambiguous	ambiguous	ambiguous	*Mytilus californianus*
Mytilus galloprovincialis	HM180707	*Mytilus coruscus*	ambiguous	ambiguous	ambiguous	ambiguous	ambiguous	ambiguous	*Mytilus californianus*
Mytilus galloprovincialis	HM180708	*Mytilus coruscus*	no id	ambiguous	ambiguous	no id	ambiguous	ambiguous	*Mytilus californianus*
Mytilus galloprovincialis	HM180709	*Mytilus coruscus*	ambiguous	ambiguous	ambiguous	ambiguous	ambiguous	ambiguous	*Mytilus californianus*
Mytilus galloprovincialis	HM180710	*Mytilus coruscus*	ambiguous	ambiguous	ambiguous	ambiguous	ambiguous	ambiguous	*Mytilus californianus*
Mytilus galloprovincialis	HM180711	*Mytilus coruscus*	ambiguous	ambiguous	ambiguous	ambiguous	ambiguous	ambiguous	*Mytilus californianus*
Mytilus galloprovincialis	HM180712	*Mytilus coruscus*	ambiguous	ambiguous	ambiguous	ambiguous	ambiguous	ambiguous	*Mytilus californianus*
Mytilus galloprovincialis	GQ480281	*Mytilus galloprovincialis*	correct	correct	correct	correct	ambiguous	ambiguous	*Mytilus galloprovincialis*
Mytilus galloprovincialis	GQ480282	*Mytilus galloprovincialis*	no id	correct	correct	no id	correct	ambiguous	*Mytilus galloprovincialis*

Mytilus galloprovincialis	GQ480284	*Mytilus galloprovincialis*	correct	correct	correct	correct	correct	ambiguous	*Mytilus galloprovincialis*
Mytilus galloprovincialis	GQ480285	*Mytilus galloprovincialis*	correct	correct	correct	correct	ambiguous	ambiguous	*Mytilus galloprovincialis*
Mytilus galloprovincialis	GQ480286	*Mytilus galloprovincialis*	correct	correct	correct	correct	ambiguous	ambiguous	*Mytilus galloprovincialis*
Mytilus galloprovincialis	GQ480292	*Mytilus galloprovincialis*	correct	correct	correct	correct	ambiguous	ambiguous	*Mytilus galloprovincialis*
Mytilus galloprovincialis	GQ480293	*Mytilus galloprovincialis*	correct	correct	correct	correct	correct	ambiguous	*Mytilus galloprovincialis*
Mytilus galloprovincialis	GQ480294	*Mytilus galloprovincialis*	correct	correct	correct	correct	ambiguous	ambiguous	*Mytilus galloprovincialis*
Mytilus edulis	HM180704	*Mytilus galloprovincialis*	no id	incorrect	incorrect	no id	incorrect	incorrect	*Mytilus galloprovincialis*
Nassarius conoidalis	JQ975567	*Nassarius conoidalis*	correct	correct	correct	correct	ambiguous	ambiguous	*Nassarius sp.*
Nassarius conoidalis	JQ975566	*Nassarius conoidalis*	correct	correct	correct	correct	ambiguous	ambiguous	*Nassarius sp.*
Nassarius conoidalis	JQ975565	*Nassarius conoidalis*	correct	correct	correct	correct	ambiguous	ambiguous	*Nassarius sp.*
Nassarius dorsatus	JQ975553	*Nassarius dorsatus*	correct	correct	correct	correct	correct	correct	*Nassarius sp.*
Nassarius dorsatus	JQ975554	*Nassarius dorsatus*	correct	correct	correct	correct	correct	correct	*Nassarius sp.*
Nassarius festivus	JQ975456	*Nassarius festivus*	correct	correct	correct	correct	correct	correct	*Nassarius festivus*
Nassarius festivus	JQ975460	*Nassarius festivus*	no id	correct	correct	no id	correct	correct	*Nassarius festivus*
Nassarius festivus	JQ975459	*Nassarius festivus*	correct	correct	correct	correct	correct	correct	*Nassarius festivus*
Nassarius festivus	JQ975458	*Nassarius festivus*	correct	correct	correct	correct	correct	correct	*Nassarius festivus*
Nassarius festivus	JQ975457	*Nassarius festivus*	correct	correct	correct	correct	correct	correct	*Nassarius festivus*
Nassarius festivus	JQ975455	*Nassarius festivus*	correct	correct	correct	correct	correct	correct	*Nassarius festivus*
Nassarius hepaticus	JQ975487	*Nassarius hepaticus*	correct	correct	correct	correct	correct	correct	*Nassarius fulgurans*
Nassarius hepaticus	JQ975491	*Nassarius hepaticus*	correct	correct	correct	correct	correct	correct	*Nassarius fulgurans*
Nassarius hepaticus	JQ975490	*Nassarius hepaticus*	correct	correct	correct	correct	correct	correct	*Nassarius fulgurans*
Nassarius hepaticus	JQ975489	*Nassarius hepaticus*	correct	correct	correct	correct	correct	correct	*Nassarius fulgurans*
Nassarius hepaticus	JQ975488	*Nassarius hepaticus*	correct	correct	correct	correct	correct	correct	*Nassarius fulgurans*
Nassarius hepaticus	JQ975486	*Nassarius hepaticus*	correct	correct	correct	correct	correct	correct	*Nassarius fulgurans*
Nassarius livescens	JQ975514	*Nassarius livescens*	no id	no id	correct	no id	no id	correct	*Nassarius livescens*
Nassarius livescens	JQ975515	*Nassarius livescens*	correct	correct	correct	correct	correct	correct	*Nassarius livescens*
Nassarius livescens	JQ975516	*Nassarius livescens*	correct	correct	correct	correct	correct	correct	*Nassarius livescens*
Nassarius livescens	JQ975517	*Nassarius livescens*	correct	correct	correct	correct	correct	correct	*Nassarius livescens*
Nassarius pullus	JQ975561	*Nassarius pullus*	correct	correct	correct	correct	correct	correct	*Nassarius sp.*
Nassarius pullus	JQ975560	*Nassarius pullus*	correct	correct	correct	correct	correct	correct	*Nassarius sp.*
Nassarius pullus	JQ975559	*Nassarius pullus*	correct	correct	correct	correct	correct	correct	*Nassarius sp.*

Nassarius pullus	JQ975558	*Nassarius pullus*	correct	correct	correct	correct	correct	correct	*Nassarius sp.*
Nassarius pullus	JQ975557	*Nassarius pullus*	correct	correct	correct	correct	correct	correct	*Nassarius sp.*
Nassarius pullus	JQ975556	*Nassarius pullus*	correct	correct	correct	correct	correct	correct	*Nassarius sp.*
Nassarius pullus	JQ975555	*Nassarius pullus*	correct	correct	correct	correct	correct	correct	*Nassarius sp.*
Nassarius semiplicatus	JQ975564	*Nassarius semiplicatus*	correct	correct	correct	correct	correct	correct	*Nassarius sp.*
Nassarius semiplicatus	JQ975563	*Nassarius semiplicatus*	correct	correct	correct	correct	correct	correct	*Nassarius sp.*
Nassarius siquijorensis	JQ975552	*Nassarius siquijorensis*	correct	correct	correct	correct	correct	ambiguous	*Nassarius canaliculatus*
Nassarius siquijorensis	HQ834076	*Nassarius siquijorensis*	correct	correct	correct	correct	correct	ambiguous	*Nassarius canaliculatus*
Nassarius siquijorensis	JN053047	*Nassarius conoidalis*	no id	incorrect	incorrect	no id	incorrect	ambiguous	*Nassarius canaliculatus*
Natica lineata	JF693401	*Natica lineata*	correct	correct	correct	correct	correct	correct	*Naticidae sp.*
Natica lineata	JF693402	*Natica lineata*	correct	correct	correct	correct	correct	correct	*Naticidae sp.*
Natica lineata	JF693404	*Natica lineata*	correct	correct	correct	correct	correct	correct	*Naticidae sp.*
Natica lineata	JF693403	*Natica lineata*	correct	correct	correct	correct	correct	correct	*Naticidae sp.*
Natica tigrina	JF693400	*Drupella fragum*	no id	no id	no id	no id	no id	no id	*Cryptonatica aleutica*
Neptunea arthritica	AB498778	*Neptunea cumingi*	ambiguous	ambiguous	ambiguous	ambiguous	ambiguous	ambiguous	*Neptunea cumingi*
Neptunea arthritica	AB498777	*Neptunea cumingi*	ambiguous	ambiguous	ambiguous	ambiguous	ambiguous	ambiguous	*Neptunea cumingi*
Neptunea arthritica	AB498776	*Neptunea cumingi*	ambiguous	ambiguous	ambiguous	ambiguous	ambiguous	ambiguous	*Neptunea cumingi*
Neptunea constricta	AB498766	*Neptunea polycostata*	no id	no id	incorrect	no id	no id	incorrect	*Neptunea constricta*
Ocinebrellus inornatus	HM180491	*Neptunea cumingi*	ambiguous	ambiguous	ambiguous	ambiguous	ambiguous	ambiguous	*Neptunea cumingi*
Ocinebrellus inornatus	HM180492	*Neptunea cumingi*	ambiguous	ambiguous	ambiguous	ambiguous	ambiguous	ambiguous	*Neptunea cumingi*
Ocinebrellus inornatus	HM180493	*Neptunea cumingi*	ambiguous	ambiguous	ambiguous	ambiguous	ambiguous	ambiguous	*Neptunea cumingi*
Neptunea cumingi	HQ834061	*Neptunea cumingi*	ambiguous	ambiguous	ambiguous	ambiguous	ambiguous	ambiguous	*Neptunea cumingi*
Neptunea cumingi	JN053006	*Neptunea cumingi*	ambiguous	ambiguous	ambiguous	ambiguous	ambiguous	ambiguous	*Neptunea cumingi*
Neptunea cumingi	JN053005	*Neptunea cumingi*	ambiguous	ambiguous	ambiguous	ambiguous	ambiguous	ambiguous	*Neptunea cumingi*
Neptunea cumingi	HM180715	*Neptunea cumingi*	ambiguous	ambiguous	ambiguous	ambiguous	ambiguous	ambiguous	*Neptunea arthritica*
Neptunea cumingi	HM180716	*Neptunea cumingi*	ambiguous	ambiguous	ambiguous	ambiguous	ambiguous	ambiguous	*Neptunea arthritica*
Neptunea kuroshio	AB498772	*Neptunea frater*	no id	incorrect	incorrect	no id	incorrect	incorrect	*Neptunea mikawaensis*
Neptunea frater	AB498769	*Neptunea kuroshio*	no id	incorrect	incorrect	no id	ambiguous	ambiguous	*Neptunea mikawaensis*
Neptunea frater	AB498768	*Neptunea frater*	correct	correct	correct	correct	correct	ambiguous	*Neptunea mikawaensis*
Neptunea frater	AB498767	*Neptunea frater*	correct	correct	correct	correct	correct	ambiguous	*Neptunea mikawaensis*
Neptunea intersculpta	AB498771	*Neptunea intersculpta*	correct	correct	correct	correct	correct	correct	*Neptunea polycostata*

Neptunea intersculpta	AB498770	*Neptunea intersculpta*	correct	correct	correct	correct	correct	correct	*Neptunea polycostata*
Neptunea mikawaensis	AB498773	*Neptunea kuroshio*	no id	no id	incorrect	no id	no id	incorrect	*Neptunea kuroshio*
Neptunea polycostata	AB498775	*Neptunea polycostata*	correct	correct	correct	correct	correct	ambiguous	*Neptunea intersculpta*
Neptunea polycostata	AB498774	*Neptunea polycostata*	correct	correct	correct	correct	correct	ambiguous	*Neptunea intersculpta*
Nerita albicilla	AM049327	*Nerita yoldii*	no id	no id	no id	no id	no id	no id	*Nerita albicilla*
Nerita helicinoides	EU732252	*Nerita helicinoides*	no id	no id	correct	no id	no id	correct	*Nerita helicinoides*
Nerita helicinoides	EU732251	*Nerita helicinoides*	no id	no id	correct	no id	no id	correct	*Nerita helicinoides*
Nerita japonica	EU732260	*Nerita japonica*	correct	correct	correct	correct	correct	ambiguous	*Nerita yoldii*
Nerita japonica	EU732259	*Nerita japonica*	correct	correct	correct	correct	correct	ambiguous	*Nerita yoldii*
Nerita ocellata	EU732280	*Nerita ocellata*	no id	correct	correct	no id	correct	correct	*Nerita argus*
Nerita ocellata	EU732279	*Nerita ocellata*	no id	correct	correct	no id	correct	correct	*Nerita argus*
Nerita planospira	EU732291	*Nerita japonica*	no id	no id	no id	no id	no id	no id	*Nerita adenensis*
Nerita plicata	EU732293	*Nerita yoldii*	no id	no id	no id	no id	no id	no id	*Nerita plicata*
Nerita tristis	EU732324	*Nerita tristis*	correct	correct	correct	correct	correct	correct	*Nerita undata*
Nerita tristis	EU732323	*Nerita tristis*	correct	correct	correct	correct	correct	correct	*Nerita undata*
Nerita undata	EU732333	*Nerita undata*	correct	correct	correct	correct	correct	correct	*Nerita undata*
Nerita undata	EU732334	*Nerita undata*	correct	correct	correct	correct	correct	correct	*Nerita undata*
Nerita undata	EU732336	*Nerita undata*	correct	correct	correct	correct	correct	correct	*Nerita undata*
Nerita undata	EU732335	*Nerita undata*	correct	correct	correct	correct	correct	correct	*Nerita undata*
Nerita undulata	EU732352	*Nerita undulata*	correct	correct	correct	correct	correct	correct	*Nerita undata*
Nerita undulata	EU732351	*Nerita undulata*	correct	correct	correct	correct	correct	correct	*Nerita undata*
Nerita yoldii	EU732359	*Nerita yoldii*	no id	correct	correct	no id	correct	correct	*Nerita japonica*
Nerita yoldii	EU732360	*Nerita yoldii*	no id	correct	correct	no id	correct	correct	*Nerita japonica*
Neritilia littoralis	AB102710	*Cerithidea djadjariensis*	no id	no id	no id	no id	no id	no id	*Neritilia cavernicola*
Neritilia mimotoi	AB102711	*Nassarius pullus*	no id	no id	no id	no id	no id	no id	*Neritilia cavernicola*
Neritilia rubida	AB102712	*Neritina petitii*	no id	no id	no id	no id	no id	no id	*Neritilia cavernicola*
Neritina asperulata	AB477474	*Neritina asperulata*	correct	correct	correct	correct	correct	correct	*Neritina asperulata*
Neritina asperulata	AB477473	*Neritina asperulata*	correct	correct	correct	correct	correct	correct	*Neritina asperulata*
Neritina asperulata	AB477472	*Neritina asperulata*	correct	correct	correct	correct	correct	correct	*Neritina asperulata*
Neritina iris	AB477491	*Neritina iris*	correct	correct	correct	correct	correct	correct	*Neritina canalis*
Neritina iris	AB477490	*Neritina iris*	correct	correct	correct	correct	correct	correct	*Neritina canalis*

Neritina petitii	AB477497	*Neritina petitii*	correct	correct	correct	correct	correct	correct	*Neritina sp.*
Neritina petitii	AB477496	*Neritina petitii*	correct	correct	correct	correct	correct	correct	*Neritina sp.*
Neritina petitii	AB477495	*Neritina petitii*	correct	correct	correct	correct	correct	correct	*Neritina sp.*
Neritina pulligera	AB477502	*Neritina pulligera*	correct	correct	correct	correct	correct	correct	*Neritina canalis*
Neritina pulligera	AB477501	*Neritina pulligera*	correct	correct	correct	correct	correct	correct	*Neritina canalis*
Nipponacmea fuscoviridis	KF953455	*Nipponacmea fuscoviridis*	no id	correct	correct	no id	correct	correct	*Nipponacmea fuscoviridis*
Nipponacmea fuscoviridis	KF953456	*Nipponacmea fuscoviridis*	correct	correct	correct	correct	correct	correct	*Nipponacmea fuscoviridis*
Nipponacmea fuscoviridis	KF953457	*Nipponacmea fuscoviridis*	correct	correct	correct	correct	correct	correct	*Nipponacmea fuscoviridis*
Nipponacmea fuscoviridis	KF953458	*Nipponacmea fuscoviridis*	correct	correct	correct	correct	correct	correct	*Nipponacmea fuscoviridis*
Nipponacmea fuscoviridis	KF953459	*Nipponacmea fuscoviridis*	correct	correct	correct	correct	correct	correct	*Nipponacmea fuscoviridis*
Nipponacmea gloriosa	AB238488	*Patelloida signata*	no id	incorrect	incorrect	no id	incorrect	incorrect	*Patelloida signata*
Nipponacmea habei	AB238489	*Lottia luchuana*	no id	no id	no id	no id	no id	no id	*Nipponacmea teramachii*
Nipponacmea concinna	KM221103	*Nipponacmea nigrans*	incorrect	incorrect	incorrect	incorrect	incorrect	incorrect	*Nipponacmea moskalevi*
Nipponacmea nigrans	KF953202	*Nipponacmea nigrans*	ambiguous	ambiguous	ambiguous	ambiguous	ambiguous	ambiguous	*Nipponacmea moskalevi*
Nipponacmea nigrans	KF953204	*Nipponacmea nigrans*	ambiguous	ambiguous	ambiguous	ambiguous	ambiguous	ambiguous	*Nipponacmea moskalevi*
Nipponacmea nigrans	KF953205	*Nipponacmea nigrans*	ambiguous	ambiguous	ambiguous	ambiguous	ambiguous	ambiguous	*Nipponacmea moskalevi*
Nipponacmea nigrans	KF953206	*Nipponacmea nigrans*	ambiguous	ambiguous	ambiguous	ambiguous	ambiguous	ambiguous	*Nipponacmea moskalevi*
Nipponacmea nigrans	KF953207	*Nipponacmea nigrans*	ambiguous	ambiguous	ambiguous	ambiguous	ambiguous	ambiguous	*Nipponacmea moskalevi*
Nipponacmea nigrans	AB238490	*Nipponacmea nigrans*	no id	no id	no id	no id	no id	no id	*Nipponacmea moskalevi*
Nipponacmea radula	KF953288	*Nipponacmea schrenckii*	ambiguous	ambiguous	ambiguous	ambiguous	ambiguous	ambiguous	*Nipponacmea schrenckii*
Nipponacmea radula	KF953289	*Nipponacmea schrenckii*	ambiguous	ambiguous	ambiguous	ambiguous	ambiguous	ambiguous	*Nipponacmea schrenckii*
Nipponacmea radula	KF953290	*Nipponacmea schrenckii*	ambiguous	ambiguous	ambiguous	ambiguous	ambiguous	ambiguous	*Nipponacmea schrenckii*
Nipponacmea radula	KF953291	*Nipponacmea schrenckii*	ambiguous	ambiguous	ambiguous	ambiguous	ambiguous	ambiguous	*Nipponacmea schrenckii*
Nipponacmea radula	KF953292	*Nipponacmea schrenckii*	ambiguous	ambiguous	ambiguous	ambiguous	ambiguous	ambiguous	*Nipponacmea schrenckii*
Nipponacmea radula	AB238491	*Nipponacmea schrenckii*	ambiguous	ambiguous	ambiguous	ambiguous	ambiguous	ambiguous	*Nipponacmea schrenckii*
Nipponacmea schrenckii	KM221040	*Nipponacmea radula*	ambiguous	ambiguous	ambiguous	ambiguous	ambiguous	ambiguous	*Nipponacmea schrenckii*
Nipponacmea schrenckii	KM221114	*Nipponacmea radula*	ambiguous	ambiguous	ambiguous	ambiguous	ambiguous	ambiguous	*Nipponacmea schrenckii*
Nipponacmea schrenckii	KM221128	*Nipponacmea radula*	ambiguous	ambiguous	ambiguous	ambiguous	ambiguous	ambiguous	*Nipponacmea schrenckii*
Nipponacmea schrenckii	KM221130	*Nipponacmea radula*	ambiguous	ambiguous	ambiguous	ambiguous	ambiguous	ambiguous	*Nipponacmea schrenckii*
Nipponacmea schrenckii	KM221131	*Nipponacmea radula*	ambiguous	ambiguous	ambiguous	ambiguous	ambiguous	ambiguous	*Nipponacmea schrenckii*
Nipponacmea schrenckii	KM221123	*Nipponacmea radula*	ambiguous	ambiguous	ambiguous	ambiguous	ambiguous	ambiguous	*Nipponacmea schrenckii*

Nipponacmea teramachii	AB238493	*Lottia tenuisculpta*	no id	no id	no id	no id	no id	no id	*Nipponacmea habei*
Nucella freycinetii	AB743761	*Nucella freycinetii*	correct	correct	correct	correct	correct	correct	
Nucella freycinetii	AB743760	*Nucella freycinetii*	correct	correct	correct	correct	correct	correct	
Nucella freycinetii	AB743759	*Nucella freycinetii*	correct	correct	correct	correct	correct	correct	
Nucella freycinetii	AB743758	*Nucella freycinetii*	correct	correct	correct	correct	correct	correct	
Nucella freycinetii	AB743757	*Nucella freycinetii*	correct	correct	correct	correct	correct	correct	
Nucella freycinetii	AB743756	*Nucella freycinetii*	correct	correct	correct	correct	correct	correct	
Octopus conispadiceus	AB430532	*Octopus vulgaris*	no id	no id	no id	no id	no id	no id	*Octopus sp.*
Octopus cyanea	AB430535	*Octopus cyanea*	correct	correct	correct	correct	correct	correct	*Octopus sp.*
Octopus cyanea	AB430534	*Octopus cyanea*	correct	correct	correct	correct	correct	correct	*Octopus sp.*
Octopus incella	AB430542	*Octopus nanhaiensis*	no id	no id	no id	no id	no id	no id	*Octopus rubescens*
Octopus longispadiceus	AB430539	*Sepia subtenuipes*	no id	no id	no id	no id	no id	no id	*Octopus rubescens*
Octopus laqueus	AB430543	*Octopus cyanea*	no id	no id	no id	no id	no id	no id	*Octopus cf. bocki*
Octopus nanhaiensis	HQ846121	*Octopus incella*	no id	no id	no id	no id	no id	no id	*Octopus cf. bocki*
Octopus oliveri	AB430533	*Sepia subtenuipes*	no id	no id	incorrect	no id	no id	incorrect	*Sepia subtenuipes*
Octopus parvus	AB430544	*Octopus wolfi*	no id	no id	no id	no id	no id	no id	*Octopodidae sp.*
Octopus tankahkeei	JX456264	*Cistopus taiwanicus*	no id	no id	no id	no id	no id	no id	*Cistopus taiwanicus*
Octopus vulgaris	HQ846110	*Octopus vulgaris*	correct	correct	correct	correct	correct	correct	*Octopus vulgaris*
Octopus vulgaris	HQ846154	*Octopus vulgaris*	correct	correct	correct	correct	correct	correct	*Octopus vulgaris*
Octopus vulgaris	JX456270	*Octopus vulgaris*	correct	correct	correct	correct	correct	correct	*Octopus vulgaris*
Octopus vulgaris	AB430547	*Octopus vulgaris*	correct	correct	correct	correct	correct	correct	*Octopus vulgaris*
Octopus vulgaris	AB430546	*Octopus vulgaris*	correct	correct	correct	correct	correct	correct	*Octopus vulgaris*
Octopus oshimai	JX456268	*Octopus vulgaris*	no id	no id	no id	no id	no id	no id	*Octopus vulgaris*
Octopus wolfi	AB430545	*Octopus longispadiceus*	no id	no id	no id	no id	no id	no id	*Octopoda sp.*
Omphalius pfeifferi	HM180730	*Omphalius pfeifferi carpenteri*	correct	correct	correct	correct	correct	correct	*Chlorostoma turbinatum*
Omphalius pfeifferi	HM180731	*Omphalius pfeifferi carpenteri*	correct	correct	correct	correct	correct	correct	*Chlorostoma turbinatum*
Ostrea stentina	LC051588	*Ostrea stentina*	correct	correct	correct	correct	correct	correct	*Ostreola stentina*
Ostrea stentina	LC051586	*Ostrea stentina*	correct	correct	correct	correct	correct	correct	*Ostreola stentina*
Ostrea stentina	LC051585	*Ostrea stentina*	no id	correct	correct	no id	correct	correct	*Ostreola stentina*
Ostrea stentina	LC051589	*Ostrea stentina*	correct	correct	correct	correct	correct	correct	*Ostreola stentina*
Ostrea stentina	LC051587	*Ostrea stentina*	correct	correct	correct	correct	correct	correct	*Ostreola stentina*

Ostrea stentina	LC051590	*Ostrea stentina*	correct	correct	correct	correct	correct	correct	*Ostreola stentina*
Ostrea stentina	LC051583	*Ostrea stentina*	no id	correct	correct	no id	correct	correct	*Ostreola stentina*
Ostrea stentina	LC051584	*Ostrea stentina*	correct	correct	correct	correct	correct	correct	*Ostreola stentina*
Ostrea stentina	LC051582	*Ostrea stentina*	correct	correct	correct	correct	correct	correct	*Ostreola stentina*
Ostrea stentina	LC051591	*Ostrea stentina*	no id	correct	correct	no id	correct	correct	*Ostreola equestris*
Paphia amabilis	HQ703249	*Paphia amabilis*	correct	correct	correct	correct	correct	correct	*Paphia euglypta*
Paphia amabilis	HQ703250	*Paphia amabilis*	correct	correct	correct	correct	correct	correct	*Paphia euglypta*
Paphia amabilis	HQ703251	*Paphia amabilis*	correct	correct	correct	correct	correct	correct	*Paphia euglypta*
Paphia amabilis	HQ703252	*Paphia amabilis*	correct	correct	correct	correct	correct	correct	*Paphia euglypta*
Paphia amabilis	HQ703253	*Paphia amabilis*	correct	correct	correct	correct	correct	correct	*Paphia euglypta*
Paphia amabilis	HQ703254	*Paphia amabilis*	correct	correct	correct	correct	correct	correct	*Paphia euglypta*
Paphia amabilis	HQ703255	*Paphia amabilis*	correct	correct	correct	correct	correct	correct	*Paphia euglypta*
Paphia amabilis	HQ703256	*Paphia amabilis*	correct	correct	correct	correct	correct	correct	*Paphia euglypta*
Paphia amabilis	HQ703257	*Paphia amabilis*	correct	correct	correct	correct	correct	correct	*Paphia euglypta*
Paphia amabilis	HQ703258	*Paphia amabilis*	correct	correct	correct	correct	correct	correct	*Paphia euglypta*
Paphia amabilis	HQ703259	*Paphia amabilis*	correct	correct	correct	correct	correct	correct	*Paphia euglypta*
Paphia amabilis	HQ703260	*Paphia amabilis*	correct	correct	correct	correct	correct	correct	*Paphia euglypta*
Paphia amabilis	HQ703261	*Paphia amabilis*	correct	correct	correct	correct	correct	correct	*Paphia euglypta*
Paphia amabilis	HQ703262	*Paphia amabilis*	correct	correct	correct	correct	correct	correct	*Paphia euglypta*
Paphia amabilis	GQ855251	*Paphia amabilis*	correct	correct	correct	correct	correct	correct	*Paphia euglypta*
Paphia amabilis	GQ855252	*Paphia amabilis*	correct	correct	correct	correct	correct	correct	*Paphia euglypta*
Paphia amabilis	GQ855253	*Paphia amabilis*	correct	correct	correct	correct	correct	correct	*Paphia euglypta*
Paphia amabilis	GQ855254	*Paphia amabilis*	correct	correct	correct	correct	correct	correct	*Paphia euglypta*
Paphia euglypta	DQ184827	*Paphia amabilis*	no id	no id	no id	no id	no id	no id	*Paphia semirugata*
Paphia gallus	HQ703233	*Paphia gallus*	no id	no id	no id	no id	no id	no id	*Paphia gallus*
Paphia gallus	HQ703234	*Paphia gallus*	correct	correct	correct	correct	correct	correct	*Paphia gallus*
Paphia gallus	HQ703235	*Paphia gallus*	correct	correct	correct	correct	correct	correct	*Paphia gallus*
Paphia gallus	GQ855248	*Paphia gallus*	correct	correct	correct	correct	correct	correct	*Paphia gallus*
Paphia gallus	GQ855249	*Paphia gallus*	correct	correct	correct	correct	correct	correct	*Paphia gallus*
Paphia gallus	GQ855250	*Paphia gallus*	correct	correct	correct	correct	correct	correct	*Paphia gallus*
Paphia gallus	JQ277814	*Paphia gallus*	correct	correct	correct	correct	correct	correct	*Paphia gallus*

Paphia gallus	HM124597	*Paphia gallus*	correct	correct	correct	correct	correct	correct	*Paphia gallus*
Paphia gallus	JQ277813	*Paphia gallus*	correct	correct	correct	correct	correct	correct	*Paphia gallus*
Paphia gallus	JQ277812	*Paphia gallus*	correct	correct	correct	correct	correct	correct	*Paphia gallus*
Paphia gallus	JQ277811	*Paphia gallus*	correct	correct	correct	correct	correct	correct	*Paphia gallus*
Paphia gallus	JQ277810	*Paphia gallus*	correct	correct	correct	correct	correct	correct	*Paphia gallus*
Paphia gallus	JQ277809	*Paphia gallus*	correct	correct	correct	correct	correct	correct	*Paphia gallus*
Paphia gallus	JQ277808	*Paphia gallus*	correct	correct	correct	correct	correct	correct	*Paphia gallus*
Paphia gallus	JQ277807	*Paphia gallus*	correct	correct	correct	correct	correct	correct	*Paphia gallus*
Paphia gallus	JQ277806	*Paphia gallus*	correct	correct	correct	correct	correct	correct	*Paphia gallus*
Paphia gallus	JQ277805	*Paphia gallus*	correct	correct	correct	correct	correct	correct	*Paphia gallus*
Paphia gallus	JQ277804	*Paphia gallus*	correct	correct	correct	correct	correct	correct	*Paphia gallus*
Paphia gallus	JQ277803	*Paphia gallus*	correct	correct	correct	correct	correct	correct	*Paphia gallus*
Paphia papilionacea	HQ703238	*Paphia papilionacea*	ambiguous	ambiguous	ambiguous	ambiguous	ambiguous	ambiguous	*Paphia semirugata*
Paphia papilionacea	HQ703239	*Paphia papilionacea*	correct	correct	correct	correct	correct	correct	*Paphia semirugata*
Paphia papilionacea	HQ703240	*Paphia papilionacea*	correct	correct	correct	correct	correct	correct	*Paphia semirugata*
Paphia papilionacea	HQ703241	*Paphia papilionacea*	correct	correct	correct	correct	correct	correct	*Paphia semirugata*
Paphia papilionacea	HQ703242	*Paphia papilionacea*	correct	correct	correct	correct	correct	correct	*Paphia semirugata*
Paphia papilionacea	HQ703243	*Paphia papilionacea*	correct	correct	correct	correct	correct	correct	*Paphia semirugata*
Paphia papilionacea	GQ855238	*Paphia papilionacea*	correct	correct	correct	correct	correct	correct	*Paphia semirugata*
Paphia papilionacea	GQ855239	*Paphia papilionacea*	correct	correct	correct	correct	correct	correct	*Paphia semirugata*
Paphia papilionacea	GQ855240	*Paphia papilionacea*	correct	correct	correct	correct	correct	correct	*Paphia semirugata*
Paphia papilionacea	GQ855241	*Paphia papilionacea*	correct	correct	correct	correct	correct	correct	*Paphia semirugata*
Paphia papilionacea	GQ855242	*Paphia papilionacea*	correct	correct	correct	correct	correct	correct	*Paphia semirugata*
Paphia papilionacea	GQ855243	*Paphia papilionacea*	correct	correct	correct	correct	correct	correct	*Paphia semirugata*
Paphia papilionacea	JN898946	*Paphia papilionacea*	correct	correct	correct	correct	correct	correct	*Paphia semirugata*
Paphia papilionacea	HM124590	*Paphia papilionacea*	correct	correct	correct	correct	correct	correct	*Paphia semirugata*
Paphia semirugata	HQ703263	*Paphia semirugata*	correct	correct	correct	correct	correct	correct	*Paphia semirugata*
Paphia semirugata	HQ703265	*Paphia semirugata*	no id	correct	correct	no id	correct	correct	*Paphia semirugata*
Paphia semirugata	HM124602	*Paphia semirugata*	correct	correct	correct	correct	correct	correct	*Paphia semirugata*
Paphia semirugata	HQ703264	*Paphia semirugata*	correct	correct	correct	correct	correct	correct	*Paphia semirugata*
Paphia sinuosa	HQ703231	*Paphia gallus*	correct	correct	correct	correct	correct	correct	*Paphia sp.*

Paphia sinuosa	JQ277815	*Paphia sinuosa*	no id	no id	no id	no id	no id	no id	*Paphia sinuosa*
Paphia textile	HQ703236	*Paphia textile*	correct	correct	correct	correct	correct	correct	*Paphia textile*
Paphia textile	HQ703237	*Paphia textile*	ambiguous	ambiguous	ambiguous	ambiguous	ambiguous	ambiguous	*Paphia textile*
Paphia textile	JN898938	*Paphia textile*	no id	ambiguous	ambiguous	no id	ambiguous	ambiguous	*Paphia textile*
Paphia undulata	HM124591	*Paphia undulata*	incorrect	incorrect	incorrect	incorrect	incorrect	incorrect	*Paphia textile*
Paphia undulata	HQ703244	*Paphia undulata*	correct	correct	correct	correct	correct	correct	*Paphia textile*
Paphia undulata	HQ703245	*Paphia undulata*	correct	correct	correct	correct	correct	correct	*Paphia textile*
Paphia undulata	HQ703246	*Paphia undulata*	correct	correct	correct	correct	correct	correct	*Paphia textile*
Paphia undulata	HQ703247	*Paphia undulata*	correct	correct	correct	correct	correct	correct	*Paphia textile*
Paphia undulata	HQ703248	*Paphia undulata*	correct	correct	correct	correct	correct	correct	*Paphia textile*
Paphia undulata	GQ855244	*Paphia undulata*	correct	correct	correct	correct	correct	correct	*Paphia textile*
Paphia undulata	GQ855245	*Paphia undulata*	correct	correct	correct	correct	correct	correct	*Paphia textile*
Paphia undulata	GQ855246	*Paphia undulata*	correct	correct	correct	correct	correct	correct	*Paphia textile*
Paphia undulata	GQ855247	*Paphia undulata*	correct	correct	correct	correct	correct	correct	*Paphia textile*
Paphia undulata	JN898933	*Paphia undulata*	correct	correct	correct	correct	correct	correct	*Paphia textile*
Paphia vernicosa	DQ184828	*Paphia papilionacea*	no id	no id	no id	no id	no id	no id	*Paphia papilionacea*
Paraoncidium reevesii	JN543145	*Paraoncidium reevesii*	correct	correct	correct	correct	correct	correct	*Platevindex cf. coriaceus*
Paraoncidium reevesii	JN543146	*Paraoncidium reevesii*	correct	correct	correct	correct	correct	correct	*Platevindex cf. coriaceus*
Paraoncidium reevesii	JN543147	*Paraoncidium reevesii*	correct	correct	correct	correct	correct	correct	*Platevindex cf. coriaceus*
Paraoncidium reevesii	JN543148	*Paraoncidium reevesii*	correct	correct	correct	correct	correct	correct	*Platevindex cf. coriaceus*
Paraoncidium reevesii	JN543149	*Paraoncidium reevesii*	correct	correct	correct	correct	correct	correct	*Platevindex cf. coriaceus*
Paraoncidium reevesii	JN543150	*Paraoncidium reevesii*	correct	correct	correct	correct	correct	correct	*Platevindex cf. coriaceus*
Paraoncidium reevesii	JN543151	*Paraoncidium reevesii*	correct	correct	correct	correct	correct	correct	*Platevindex cf. coriaceus*
Patelloida conulus	AB161565	*Patelloida conulus*	correct	correct	correct	correct	correct	correct	*Patelloida conulus*
Patelloida conulus	AB161566	*Patelloida conulus*	correct	correct	correct	correct	correct	correct	*Patelloida conulus*
Patelloida conulus	AB161567	*Patelloida conulus*	correct	correct	correct	correct	correct	correct	*Patelloida conulus*
Patelloida conulus	AB161568	*Patelloida conulus*	no id	correct	correct	no id	correct	correct	*Patelloida conulus*
Patelloida conulus	AB161569	*Patelloida conulus*	correct	correct	correct	correct	correct	correct	*Patelloida conulus*
Patelloida conulus	AB161570	*Patelloida conulus*	no id	correct	correct	no id	correct	correct	*Patelloida conulus*
Patelloida conulus	AB161571	*Patelloida conulus*	correct	correct	correct	correct	correct	correct	*Patelloida conulus*
Patelloida conulus	AB161572	*Patelloida conulus*	correct	correct	correct	correct	correct	correct	*Patelloida conulus*

Patelloida conulus	AB161573	*Patelloida conulus*	no id	correct	correct	no id	correct	correct	*Patelloida conulus*
Patelloida conulus	AB161574	*Patelloida conulus*	correct	correct	correct	correct	correct	correct	*Patelloida conulus*
Patelloida conulus	AB161604	*Patelloida conulus*	correct	correct	correct	correct	correct	correct	*Patelloida conulus*
Patelloida conulus	AB161605	*Patelloida conulus*	correct	correct	correct	correct	correct	correct	*Patelloida conulus*
Patelloida conulus	AB161606	*Patelloida conulus*	correct	correct	correct	correct	correct	correct	*Patelloida conulus*
Patelloida conulus	AB161607	*Patelloida conulus*	correct	correct	correct	correct	correct	correct	*Patelloida conulus*
Patelloida conulus	AB161608	*Patelloida conulus*	correct	correct	correct	correct	correct	correct	*Patelloida conulus*
Patelloida conulus	AB161609	*Patelloida conulus*	correct	correct	correct	correct	correct	correct	*Patelloida conulus*
Patelloida conulus	AB161610	*Patelloida conulus*	correct	correct	correct	correct	correct	correct	*Patelloida conulus*
Patelloida conulus	AB161611	*Patelloida conulus*	correct	correct	correct	correct	correct	correct	*Patelloida conulus*
Patelloida conulus	AB161612	*Patelloida conulus*	correct	correct	correct	correct	correct	correct	*Patelloida conulus*
Patelloida conulus	AB161613	*Patelloida conulus*	correct	correct	correct	correct	correct	correct	*Patelloida conulus*
Patelloida conulus	AB161614	*Patelloida conulus*	correct	correct	correct	correct	correct	correct	*Patelloida conulus*
Patelloida conulus	AB161615	*Patelloida conulus*	correct	correct	correct	correct	correct	correct	*Patelloida conulus*
Patelloida conulus	AB161616	*Patelloida conulus*	correct	correct	correct	correct	correct	correct	*Patelloida conulus*
Patelloida conulus	AB161617	*Patelloida conulus*	correct	correct	correct	correct	correct	correct	*Patelloida conulus*
Patelloida conulus	AB238514	*Patelloida conulus*	correct	correct	correct	correct	correct	correct	*Patelloida conulus*
Patelloida heroldi	AB161575	*Patelloida heroldi*	correct	correct	correct	correct	correct	correct	*Patelloida ryukyuensis*
Patelloida heroldi	AB161576	*Patelloida heroldi*	correct	correct	correct	correct	correct	correct	*Patelloida ryukyuensis*
Patelloida heroldi	AB161577	*Patelloida heroldi*	correct	correct	correct	correct	correct	correct	*Patelloida ryukyuensis*
Patelloida heroldi	AB161578	*Patelloida heroldi*	correct	correct	correct	correct	correct	correct	*Patelloida ryukyuensis*
Patelloida heroldi	AB161579	*Patelloida heroldi*	correct	correct	correct	correct	correct	correct	*Patelloida ryukyuensis*
Patelloida heroldi	AB161580	*Patelloida heroldi*	correct	correct	correct	correct	correct	correct	*Patelloida ryukyuensis*
Patelloida heroldi	AB161581	*Patelloida heroldi*	correct	correct	correct	correct	correct	correct	*Patelloida ryukyuensis*
Patelloida heroldi	AB161582	*Patelloida heroldi*	correct	correct	correct	correct	correct	correct	*Patelloida ryukyuensis*
Patelloida heroldi	AB161618	*Patelloida heroldi*	correct	correct	correct	correct	correct	correct	*Patelloida ryukyuensis*
Patelloida heroldi	AB161619	*Patelloida heroldi*	correct	correct	correct	correct	correct	correct	*Patelloida ryukyuensis*
Patelloida heroldi	AB161620	*Patelloida heroldi*	correct	correct	correct	correct	correct	correct	*Patelloida ryukyuensis*
Patelloida heroldi	AB161621	*Patelloida heroldi*	correct	correct	correct	correct	correct	correct	*Patelloida ryukyuensis*
Patelloida heroldi	AB161622	*Patelloida heroldi*	correct	correct	correct	correct	correct	correct	*Patelloida ryukyuensis*
Patelloida heroldi	AB161623	*Patelloida heroldi*	correct	correct	correct	correct	correct	correct	*Patelloida ryukyuensis*

Patelloida heroldi	AB161624	*Patelloida heroldi*	correct	correct	correct	correct	correct	correct	*Patelloida ryukyuensis*
Patelloida heroldi	AB161625	*Patelloida heroldi*	correct	correct	correct	correct	correct	correct	*Patelloida ryukyuensis*
Patelloida heroldi	AB161626	*Patelloida heroldi*	correct	correct	correct	correct	correct	correct	*Patelloida ryukyuensis*
Patelloida heroldi	AB161627	*Patelloida heroldi*	correct	correct	correct	correct	correct	correct	*Patelloida ryukyuensis*
Patelloida heroldi	AB161628	*Patelloida heroldi*	correct	correct	correct	correct	correct	correct	*Patelloida ryukyuensis*
Patelloida heroldi	AB161629	*Patelloida heroldi*	correct	correct	correct	correct	correct	correct	*Patelloida ryukyuensis*
Patelloida heroldi	AB161630	*Patelloida heroldi*	correct	correct	correct	correct	correct	correct	*Patelloida ryukyuensis*
Patelloida heroldi	AB161631	*Patelloida heroldi*	correct	correct	correct	correct	correct	correct	*Patelloida ryukyuensis*
Patelloida lentiginosa	AB238517	*Patelloida heroldi*	no id	no id	no id	no id	no id	no id	*Patelloida sp.*
Patelloida pygmaea	KM221033	*Patelloida pygmaea*	correct	correct	correct	correct	correct	correct	*Patelloida conulus*
Patelloida pygmaea	KM221111	*Patelloida pygmaea*	correct	correct	correct	correct	correct	correct	*Patelloida conulus*
Patelloida pygmaea	KM221112	*Patelloida pygmaea*	correct	correct	correct	correct	correct	correct	*Patelloida conulus*
Patelloida pygmaea	KM221113	*Patelloida pygmaea*	correct	correct	correct	correct	correct	correct	*Patelloida conulus*
Patelloida pygmaea	KM221041	*Patelloida pygmaea*	correct	correct	correct	correct	correct	correct	*Patelloida conulus*
Patelloida pygmaea	KM221147	*Patelloida pygmaea*	correct	correct	correct	correct	correct	correct	*Patelloida conulus*
Patelloida pygmaea	KM221148	*Patelloida pygmaea*	correct	correct	correct	correct	correct	correct	*Patelloida conulus*
Patelloida pygmaea	KM221149	*Patelloida pygmaea*	correct	correct	correct	correct	correct	correct	*Patelloida conulus*
Patelloida pygmaea	KM221150	*Patelloida pygmaea*	correct	correct	correct	correct	correct	correct	*Patelloida conulus*
Patelloida pygmaea	AB161552	*Patelloida pygmaea*	correct	correct	correct	correct	correct	correct	*Patelloida conulus*
Patelloida pygmaea	AB161553	*Patelloida pygmaea*	correct	correct	correct	correct	correct	correct	*Patelloida conulus*
Patelloida pygmaea	AB161554	*Patelloida pygmaea*	correct	correct	correct	correct	correct	correct	*Patelloida conulus*
Patelloida pygmaea	AB161555	*Patelloida pygmaea*	correct	correct	correct	correct	correct	correct	*Patelloida conulus*
Patelloida pygmaea	AB161556	*Patelloida pygmaea*	correct	correct	correct	correct	correct	correct	*Patelloida conulus*
Patelloida pygmaea	AB161557	*Patelloida pygmaea*	correct	correct	correct	correct	correct	correct	*Patelloida conulus*
Patelloida pygmaea	AB161560	*Patelloida pygmaea*	correct	correct	correct	correct	correct	correct	*Patelloida conulus*
Patelloida pygmaea	AB161561	*Patelloida pygmaea*	correct	correct	correct	correct	correct	correct	*Patelloida conulus*
Patelloida pygmaea	AB161558	*Patelloida pygmaea*	correct	correct	correct	correct	correct	correct	*Patelloida conulus*
Patelloida pygmaea	AB161559	*Patelloida pygmaea*	correct	correct	correct	correct	correct	correct	*Patelloida conulus*
Patelloida pygmaea	AB161562	*Patelloida pygmaea*	correct	correct	correct	correct	correct	correct	*Patelloida conulus*
Patelloida pygmaea	AB161563	*Patelloida pygmaea*	correct	correct	correct	correct	correct	correct	*Patelloida conulus*
Patelloida pygmaea	AB161564	*Patelloida pygmaea*	correct	correct	correct	correct	correct	correct	*Patelloida conulus*

Patelloida pygmaea	AB161590	*Patelloida pygmaea*	correct	correct	correct	correct	correct	correct	*Patelloida conulus*
Patelloida pygmaea	AB161591	*Patelloida pygmaea*	correct	correct	correct	correct	correct	correct	*Patelloida conulus*
Patelloida pygmaea	AB161592	*Patelloida pygmaea*	correct	correct	correct	correct	correct	correct	*Patelloida conulus*
Patelloida pygmaea	AB161593	*Patelloida pygmaea*	no id	correct	correct	no id	correct	correct	*Patelloida conulus*
Patelloida pygmaea	AB161594	*Patelloida pygmaea*	no id	correct	correct	no id	correct	correct	*Patelloida conulus*
Patelloida pygmaea	AB161595	*Patelloida pygmaea*	correct	correct	correct	correct	correct	correct	*Patelloida conulus*
Patelloida pygmaea	AB161596	*Patelloida pygmaea*	correct	correct	correct	correct	correct	correct	*Patelloida conulus*
Patelloida pygmaea	AB161599	*Patelloida pygmaea*	correct	correct	correct	correct	correct	correct	*Patelloida conulus*
Patelloida pygmaea	AB161600	*Patelloida pygmaea*	correct	correct	correct	correct	correct	correct	*Patelloida conulus*
Patelloida pygmaea	AB161601	*Patelloida pygmaea*	correct	correct	correct	correct	correct	correct	*Patelloida conulus*
Patelloida pygmaea	AB161602	*Patelloida pygmaea*	correct	correct	correct	correct	correct	correct	*Patelloida conulus*
Patelloida pygmaea	AB161603	*Patelloida pygmaea*	correct	correct	correct	correct	correct	correct	*Patelloida conulus*
Patelloida ryukyuensis	KM221059	*Patelloida ryukyuensis*	correct	correct	correct	correct	correct	correct	*Patelloida ryukyuensis*
Patelloida ryukyuensis	KM221075	*Patelloida ryukyuensis*	correct	correct	correct	correct	correct	correct	*Patelloida ryukyuensis*
Patelloida ryukyuensis	KM221076	*Patelloida ryukyuensis*	correct	correct	correct	correct	correct	correct	*Patelloida ryukyuensis*
Patelloida ryukyuensis	KM221091	*Patelloida ryukyuensis*	correct	correct	correct	correct	correct	correct	*Patelloida ryukyuensis*
Patelloida ryukyuensis	KM221092	*Patelloida ryukyuensis*	no id	correct	correct	no id	correct	correct	*Patelloida ryukyuensis*
Patelloida ryukyuensis	KM221093	*Patelloida ryukyuensis*	no id	correct	correct	no id	correct	correct	*Patelloida ryukyuensis*
Patelloida ryukyuensis	KM221117	*Patelloida ryukyuensis*	correct	correct	correct	correct	correct	correct	*Patelloida ryukyuensis*
Patelloida ryukyuensis	KM221099	*Patelloida ryukyuensis*	correct	correct	correct	correct	correct	correct	*Patelloida ryukyuensis*
Patelloida ryukyuensis	AB196508	*Patelloida ryukyuensis*	correct	correct	correct	correct	correct	correct	*Patelloida ryukyuensis*
Patelloida ryukyuensis	AB196509	*Patelloida ryukyuensis*	correct	correct	correct	correct	correct	correct	*Patelloida ryukyuensis*
Patelloida saccharina form lanx	KM221083	*Patelloida saccharina form lanx*	correct	correct	correct	correct	correct	correct	*Patelloida saccharinoides*
Patelloida saccharina form lanx	KM221084	*Patelloida saccharina form lanx*	correct	correct	correct	correct	correct	correct	*Patelloida saccharinoides*
Patelloida saccharina form lanx	KM221085	*Patelloida saccharina form lanx*	correct	correct	correct	correct	correct	correct	*Patelloida saccharinoides*
Patelloida saccharina form lanx	KM221086	*Patelloida saccharina form lanx*	correct	correct	correct	correct	correct	correct	*Patelloida saccharinoides*
Patelloida saccharina form lanx	KM221087	*Patelloida saccharina form lanx*	correct	correct	correct	correct	correct	correct	*Patelloida saccharinoides*
Patelloida saccharina form lanx	KM221090	*Patelloida saccharina form lanx*	correct	correct	correct	correct	correct	correct	*Patelloida saccharinoides*
Patelloida saccharina form lanx	KM221102	*Patelloida saccharina form lanx*	correct	correct	correct	correct	correct	correct	*Patelloida saccharinoides*
Patelloida signata	AB161632	*Nipponacmea gloriosa*	no id	no id	no id	no id	no id	no id	*Nipponacmea gloriosa*
Patelloida striata	AB161589	*Uroteuthis duvaucelii*	no id	incorrect	incorrect	no id	incorrect	incorrect	*Dascyllus trimaculatus*

Pelecyora isocardia	HQ703209	*Pelecyora isocardia*	no id	no id	correct	no id	no id	correct	*Callista chione*
Pelecyora isocardia	HQ703210	*Pelecyora isocardia*	ambiguous	ambiguous	ambiguous	ambiguous	ambiguous	ambiguous	*Callista chione*
Pelecyora trigona	HM124586	*Pelecyora isocardia*	incorrect	incorrect	incorrect	incorrect	incorrect	incorrect	*Callista chione*
Periglypta chemnitzii	HQ703330	*Periglypta chemnitzi*	correct	correct	correct	correct	correct	correct	*Antigona lamellaris*
Periglypta chemnitzii	HQ703331	*Periglypta chemnitzi*	correct	correct	correct	correct	correct	correct	*Antigona lamellaris*
Periglypta chemnitzii	HQ703332	*Periglypta chemnitzi*	correct	correct	correct	correct	correct	correct	*Antigona lamellaris*
Periglypta chemnitzii	HQ703333	*Periglypta chemnitzi*	correct	correct	correct	correct	correct	correct	*Antigona lamellaris*
Periglypta chemnitzii	HM124614	*Periglypta chemnitzi*	correct	correct	correct	correct	correct	correct	*Antigona lamellaris*
Periglypta puerpera	HQ703318	*Periglypta puerpera*	correct	correct	correct	correct	correct	correct	*Periglypta puerpera*
Periglypta puerpera	HQ703319	*Periglypta puerpera*	correct	correct	correct	correct	correct	correct	*Periglypta puerpera*
Periglypta puerpera	HQ703320	*Periglypta puerpera*	correct	correct	correct	correct	correct	correct	*Periglypta puerpera*
Periglypta puerpera	HQ703321	*Periglypta puerpera*	correct	correct	correct	correct	correct	correct	*Periglypta puerpera*
Periglypta puerpera	HQ703322	*Periglypta puerpera*	correct	correct	correct	correct	correct	correct	*Periglypta puerpera*
Periglypta puerpera	HQ703323	*Periglypta puerpera*	correct	correct	correct	correct	correct	correct	*Periglypta puerpera*
Periglypta puerpera	HQ703324	*Periglypta puerpera*	correct	correct	correct	correct	correct	correct	*Periglypta puerpera*
Periglypta puerpera	HQ703325	*Periglypta puerpera*	correct	correct	correct	correct	correct	correct	*Periglypta puerpera*
Periglypta puerpera	HQ703326	*Periglypta puerpera*	correct	correct	correct	correct	correct	correct	*Periglypta puerpera*
Periglypta puerpera	HQ703327	*Periglypta puerpera*	correct	correct	correct	correct	correct	correct	*Periglypta puerpera*
Periglypta puerpera	HQ703328	*Periglypta puerpera*	correct	correct	correct	correct	correct	correct	*Periglypta puerpera*
Periglypta puerpera	HQ703329	*Periglypta compressa*	incorrect	incorrect	incorrect	incorrect	incorrect	incorrect	*Periglypta puerpera*
Periglypta compressa	HM124606	*Periglypta puerpera*	incorrect	incorrect	incorrect	incorrect	incorrect	incorrect	*Periglypta puerpera*
Perna viridis	GQ480296	*Perna viridis*	correct	correct	correct	correct	correct	correct	*Mytella charruana*
Perna viridis	GQ480297	*Perna viridis*	correct	correct	correct	correct	correct	correct	*Mytella charruana*
Perna viridis	GQ480298	*Perna viridis*	correct	correct	correct	correct	correct	correct	*Mytella charruana*
Perna viridis	GQ480299	*Perna viridis*	correct	correct	correct	correct	correct	correct	*Mytella charruana*
Perna viridis	GQ480300	*Perna viridis*	correct	correct	correct	correct	correct	correct	*Mytella charruana*
Perna viridis	GQ480301	*Perna viridis*	correct	correct	correct	correct	correct	correct	*Mytella charruana*
Perna viridis	GQ480302	*Perna viridis*	correct	correct	correct	correct	correct	correct	*Mytella charruana*
Perna viridis	GQ480303	*Perna viridis*	correct	correct	correct	correct	correct	correct	*Mytella charruana*
Perna viridis	GQ480304	*Perna viridis*	correct	correct	correct	correct	correct	correct	*Mytella charruana*
Peronia verruculata	JN543152	*Peronia verruculata*	correct	correct	correct	correct	correct	correct	*Peronia sp.*

Peronia verruculata	JN543153	*Peronia verruculata*	correct	correct	correct	correct	correct	correct	*Peronia sp.*
Peronia verruculata	JN543154	*Peronia verruculata*	correct	correct	correct	correct	correct	correct	*Peronia sp.*
Phalium bisulcatum	JF693409	*Ficus variegata*	no id	no id	no id	no id	no id	no id	*Casmaria ponderosa*
Phalium flammiferum	JF693410	*Duplicaria dussumieri*	no id	no id	no id	no id	no id	no id	*Casmaria boblehmani*
Phasianella solida	AM049353	*Phasianella solida*	no id	correct	correct	no id	correct	correct	*Phasianella ventricosa*
Phasianella solida	AM049354	*Phasianella solida*	no id	correct	correct	no id	correct	correct	*Phasianella ventricosa*
Pholas orientalis	KJ125422	*Pholas orientalis*	correct	correct	correct	correct	correct	correct	*Martesia striata*
Pholas orientalis	KJ125423	*Pholas orientalis*	no id	no id	correct	no id	no id	correct	*Martesia striata*
Phos senticosus	JN053008	*Phos senticosus*	correct	correct	correct	correct	correct	correct	*Phos textilis*
Phos senticosus	JN053009	*Phos senticosus*	no id	correct	correct	no id	correct	correct	*Phos textilis*
Phos senticosus	HQ834064	*Phos senticosus*	correct	correct	correct	correct	correct	correct	*Phos textilis*
Phos senticosus	HQ834065	*Phos senticosus*	correct	correct	correct	correct	correct	correct	*Phos textilis*
Pinctada albina	AB261165	*Pinctada maxima*	no id	no id	no id	no id	no id	no id	*Pinctada maculata*
Pinctada chemnitzi	KU341955	*Pinctada chemnitzi*	correct	correct	correct	correct	correct	correct	*Pinctada maculata*
Pinctada chemnitzi	KU341956	*Pinctada chemnitzi*	correct	correct	correct	correct	correct	correct	*Pinctada maculata*
Pinctada chemnitzi	KU341957	*Pinctada chemnitzi*	correct	correct	correct	correct	correct	correct	*Pinctada maculata*
Pinctada chemnitzi	KU341958	*Pinctada chemnitzi*	correct	correct	correct	correct	correct	correct	*Pinctada maculata*
Pinctada fucata	KU341939	*Pinctada fucata*	correct	correct	correct	correct	correct	correct	*Pinctada radiata*
Pinctada fucata	KU341940	*Pinctada fucata*	ambiguous	ambiguous	ambiguous	ambiguous	ambiguous	ambiguous	*Pinctada radiata*
Pinctada fucata	KU341941	*Pinctada fucata*	ambiguous	ambiguous	ambiguous	ambiguous	ambiguous	ambiguous	*Pinctada radiata*
Pinctada fucata	KU341942	*Pinctada fucata*	ambiguous	ambiguous	ambiguous	ambiguous	ambiguous	ambiguous	*Pinctada radiata*
Pinctada fucata	KU341943	*Pinctada fucata*	ambiguous	ambiguous	ambiguous	ambiguous	ambiguous	ambiguous	*Pinctada radiata*
Pinctada fucata	KU341944	*Pinctada fucata*	ambiguous	ambiguous	ambiguous	ambiguous	ambiguous	ambiguous	*Pinctada radiata*
Pinctada fucata	GQ355871	*Pinctada fucata*	no id	ambiguous	ambiguous	no id	ambiguous	ambiguous	*Pinctada radiata*
Pinctada martensi	GQ355882	*Pinctada fucata*	ambiguous	ambiguous	ambiguous	ambiguous	ambiguous	ambiguous	*Pinctada radiata*
Pinctada martensi	AB076915	*Pinctada fucata*	ambiguous	ambiguous	ambiguous	ambiguous	ambiguous	ambiguous	*Pinctada radiata*
Pinctada margaritifera	KU341945	*Pinctada margaritifera*	ambiguous	ambiguous	ambiguous	ambiguous	ambiguous	ambiguous	*Pinctada sp.*
Pinctada margaritifera	KU341946	*Pinctada margaritifera*	no id	correct	correct	no id	correct	correct	*Pinctada sp.*
Pinctada margaritifera	KU341947	*Pinctada margaritifera*	correct	correct	correct	correct	correct	correct	*Pinctada sp.*
Pinctada margaritifera	KU341948	*Pinctada margaritifera*	correct	correct	correct	correct	correct	correct	*Pinctada sp.*
Pinctada margaritifera	KU341949	*Pinctada margaritifera*	correct	correct	correct	correct	correct	correct	*Pinctada sp.*

Pinctada margaritifera	KU341950	*Pinctada margaritifera*	correct	correct	correct	correct	correct	correct	*Pinctada sp.*
Pinctada margaritifera	KU341951	*Pinctada margaritifera*	correct	correct	correct	correct	correct	correct	*Pinctada sp.*
Pinctada margaritifera	KU341952	*Pinctada margaritifera*	correct	correct	correct	correct	correct	correct	*Pinctada sp.*
Pinctada margaritifera	KU341953	*Pinctada margaritifera*	correct	correct	correct	correct	correct	correct	*Pinctada sp.*
Pinctada margaritifera	KU341954	*Pinctada margaritifera*	correct	correct	correct	correct	correct	correct	*Pinctada sp.*
Pinctada margaritifera	AB259166	*Pinctada margaritifera*	no id	correct	correct	no id	correct	correct	*Pinctada sp.*
Pinctada maxima	KU341937	*Pinctada maxima*	correct	correct	correct	correct	correct	correct	*Pinctada margaritifera*
Pinctada maxima	KU341938	*Pinctada maxima*	correct	correct	correct	correct	correct	correct	*Pinctada margaritifera*
Pitarina japonica	HQ703206	*Pitarina japonica*	correct	correct	correct	correct	correct	correct	*Pitar japonicum*
Pitarina japonica	HQ703207	*Pitarina japonica*	no id	no id	correct	no id	no id	correct	*Pitar japonicum*
Pitarina striatum	HQ703203	*Pitar striatus*	correct	correct	correct	correct	correct	correct	*Pitar japonicum*
Pitarina striatum	HQ703204	*Pitar striatus*	correct	correct	correct	correct	correct	correct	*Pitar japonicum*
Pitarina striatum	HQ703205	*Pitar striatus*	correct	correct	correct	correct	correct	correct	*Pitar japonicum*
Placamen calophylla	HQ703062	*Placamen calophylla*	correct	correct	correct	correct	correct	correct	*Placamen sp.*
Placamen calophylla	HQ703063	*Placamen calophylla*	correct	correct	correct	correct	correct	correct	*Placamen sp.*
Placamen calophylla	HQ703064	*Placamen calophylla*	correct	correct	correct	correct	correct	correct	*Placamen sp.*
Placamen isabellina	HQ703057	*Placamen isabellina*	correct	correct	correct	correct	correct	correct	*Placamen flindersi*
Placamen isabellina	HQ703058	*Placamen isabellina*	correct	correct	correct	correct	correct	correct	*Placamen flindersi*
Placamen isabellina	HQ703059	*Placamen isabellina*	correct	correct	correct	correct	correct	correct	*Placamen flindersi*
Placamen isabellina	HQ703060	*Placamen isabellina*	correct	correct	correct	correct	correct	correct	*Placamen flindersi*
Placamen isabellina	HQ703061	*Placamen isabellina*	correct	correct	correct	correct	correct	correct	*Placamen flindersi*
Plakobranchus ocellatus	AB758971	*Elysia ornata*	no id	no id	no id	no id	no id	no id	*Plakobranchus ocellatus*
Plakobranchus ocellatus	AB758970	*Plakobranchus ocellatus*	no id	correct	correct	no id	correct	correct	*Plakobranchus sp.*
Plakobranchus ocellatus	AB501307	*Plakobranchus ocellatus*	no id	correct	correct	no id	correct	correct	*Plakobranchus sp.*
Plakobranchus ocellatus	AB758969	*Plakobranchus ocellatus*	no id	no id	no id	no id	no id	no id	*Plakobranchus sp.*
Plakobranchus ocellatus	AB758968	*Elysia ornata*	no id	no id	no id	no id	no id	no id	*Plakobranchus sp.*
Platevindex mortoni	JN543136	*Platevindex mortoni*	correct	correct	correct	correct	correct	correct	*Platevindex mortoni*
Platevindex mortoni	JN543137	*Platevindex mortoni*	correct	correct	correct	correct	correct	correct	*Platevindex mortoni*
Platevindex mortoni	JN543139	*Platevindex mortoni*	correct	correct	correct	correct	correct	correct	*Platevindex mortoni*
Platevindex mortoni	JN543140	*Platevindex mortoni*	correct	correct	correct	correct	correct	correct	*Platevindex mortoni*
Platevindex mortoni	JN543141	*Platevindex mortoni*	correct	correct	correct	correct	correct	correct	*Platevindex mortoni*

Platevindex mortoni	JN543138	*Platevindex mortoni*	no id	no id	no id	no id	no id	no id	*Platevindex mortoni*
Pomaulax japonicus	AM049380	*Pomaulax japonicus*	correct	correct	correct	correct	correct	correct	*Pomaulax gibberosus*
Pomaulax japonicus	AB297737	*Pomaulax japonicus*	correct	correct	correct	correct	correct	correct	*Pomaulax gibberosus*
Protaeolidiella atra	KP143676	*Protaeolidiella atra*	correct	correct	correct	correct	correct	correct	*Doto cf. cuspidata*
Protaeolidiella atra	KP143675	*Protaeolidiella atra*	correct	correct	correct	correct	correct	correct	*Doto cf. cuspidata*
Protothaca jedoensis	HQ703065	*Protothaca jedoensis*	correct	correct	correct	correct	correct	correct	*Protothaca jedoensis*
Protothaca jedoensis	HQ703066	*Protothaca jedoensis*	correct	correct	correct	correct	correct	correct	*Protothaca jedoensis*
Protothaca jedoensis	HQ703067	*Protothaca jedoensis*	no id	correct	correct	no id	correct	correct	*Protothaca jedoensis*
Protothaca jedoensis	HQ703068	*Protothaca jedoensis*	correct	correct	correct	correct	correct	correct	*Protothaca jedoensis*
Protothaca jedoensis	HQ703069	*Protothaca jedoensis*	correct	correct	correct	correct	correct	correct	*Protothaca jedoensis*
Protothaca jedoensis	HQ703070	*Protothaca jedoensis*	correct	correct	correct	correct	correct	correct	*Protothaca jedoensis*
Protothaca jedoensis	HM124607	*Protothaca jedoensis*	correct	correct	correct	correct	correct	correct	*Protothaca jedoensis*
Protothaca jedoensis	HM124605	*Protothaca jedoensis*	correct	correct	correct	correct	correct	correct	*Protothaca jedoensis*
Protothaca jedoensis	DQ399397	*Protothaca jedoensis*	incorrect	incorrect	incorrect	incorrect	incorrect	incorrect	*Mactra quadrangularis*
Mactra veneriformis	JN674621	*Mactra veneriformis*	ambiguous	ambiguous	ambiguous	ambiguous	ambiguous	ambiguous	*Mactra quadrangularis*
Mactra veneriformis	JN674622	*Mactra veneriformis*	ambiguous	ambiguous	ambiguous	ambiguous	ambiguous	ambiguous	*Mactra quadrangularis*
Mactra veneriformis	JN674623	*Mactra veneriformis*	ambiguous	ambiguous	ambiguous	ambiguous	ambiguous	ambiguous	*Mactra quadrangularis*
Mactra veneriformis	JN674624	*Mactra veneriformis*	no id	ambiguous	ambiguous	no id	ambiguous	ambiguous	*Mactra quadrangularis*
Mactra veneriformis	JN674625	*Mactra veneriformis*	ambiguous	ambiguous	ambiguous	ambiguous	ambiguous	ambiguous	*Mactra quadrangularis*
Mactra veneriformis	GQ864250	*Mactra veneriformis*	ambiguous	ambiguous	ambiguous	ambiguous	ambiguous	ambiguous	*Mactra quadrangularis*
Mactra veneriformis	FJ851355	*Mactra veneriformis*	ambiguous	ambiguous	ambiguous	ambiguous	ambiguous	ambiguous	*Mactra quadrangularis*
Mactra veneriformis	GQ864249	*Mactra veneriformis*	ambiguous	ambiguous	ambiguous	ambiguous	ambiguous	ambiguous	*Mactra quadrangularis*
Mactra veneriformis	GQ864248	*Mactra veneriformis*	ambiguous	ambiguous	ambiguous	ambiguous	ambiguous	ambiguous	*Mactra quadrangularis*
Mactra veneriformis	GQ864247	*Mactra veneriformis*	ambiguous	ambiguous	ambiguous	ambiguous	ambiguous	ambiguous	*Mactra quadrangularis*
Mactra veneriformis	GQ864246	*Mactra veneriformis*	no id	ambiguous	ambiguous	no id	ambiguous	ambiguous	*Mactra quadrangularis*
Mactra veneriformis	GQ864245	*Mactra veneriformis*	ambiguous	ambiguous	ambiguous	ambiguous	ambiguous	ambiguous	*Mactra quadrangularis*
Mactra veneriformis	GQ864244	*Mactra veneriformis*	ambiguous	ambiguous	ambiguous	ambiguous	ambiguous	ambiguous	*Mactra quadrangularis*
Mactra veneriformis	GQ864243	*Mactra veneriformis*	no id	ambiguous	ambiguous	no id	ambiguous	ambiguous	*Mactra quadrangularis*
Mactra veneriformis	GQ864242	*Mactra veneriformis*	ambiguous	ambiguous	ambiguous	ambiguous	ambiguous	ambiguous	*Mactra quadrangularis*
Mactra veneriformis	GQ864241	*Mactra veneriformis*	ambiguous	ambiguous	ambiguous	ambiguous	ambiguous	ambiguous	*Mactra quadrangularis*
Mactra veneriformis	GQ864240	*Mactra veneriformis*	ambiguous	ambiguous	ambiguous	ambiguous	ambiguous	ambiguous	*Mactra quadrangularis*

Mactra veneriformis	GQ864239	*Mactra veneriformis*	ambiguous	ambiguous	ambiguous	ambiguous	ambiguous	ambiguous	*Mactra quadrangularis*
Mactra veneriformis	GQ864238	*Mactra veneriformis*	ambiguous	ambiguous	ambiguous	ambiguous	ambiguous	ambiguous	*Mactra quadrangularis*
Pseudocardium sachalinensis	JN674604	*Pseudocardium sachalinensis*	no id	correct	correct	no id	correct	correct	*Pseudocardium sachalinense*
Pseudocardium sachalinensis	JN674605	*Pseudocardium sachalinensis*	no id	correct	correct	no id	correct	correct	*Pseudocardium sachalinense*
Pseudostomatella decolorata	AB505290	*Pseudostomatella decolorata*	correct	correct	correct	correct	correct	correct	*Stomatia phymotis*
Pseudostomatella decolorata	AB505291	*Pseudostomatella decolorata*	correct	correct	correct	correct	correct	correct	*Stomatia phymotis*
Pteria brevialata	KU341962	*Pteria penguin*	correct	correct	correct	correct	correct	correct	*Pteria sp.*
Pteria penguin	KU341959	*Pteria penguin*	correct	correct	correct	correct	correct	correct	*Pteria loveni*
Pteria penguin	KU341960	*Pteria penguin*	correct	correct	correct	correct	correct	correct	*Pteria loveni*
Pteria penguin	KU341961	*Pteria penguin*	no id	correct	correct	no id	correct	correct	*Pteria loveni*
Rapana bezoar	GU188166	*Rapana bezoar*	correct	correct	correct	correct	correct	correct	*Muricidae sp.*
Rapana bezoar	GU188167	*Rapana bezoar*	correct	correct	correct	correct	correct	correct	*Muricidae sp.*
Rapana bezoar	GU188168	*Rapana bezoar*	correct	correct	correct	correct	correct	correct	*Muricidae sp.*
Rapana bezoar	GU188169	*Rapana bezoar*	correct	correct	correct	correct	correct	correct	*Muricidae sp.*
Rapana bezoar	GU188170	*Rapana bezoar*	correct	correct	correct	correct	correct	correct	*Muricidae sp.*
Rapana bezoar	FN677421	*Rapana bezoar*	correct	correct	correct	correct	correct	correct	*Muricidae sp.*
Rapana rapiformis	GU188189	*Rapana rapiformis*	correct	correct	correct	correct	correct	correct	*Rapana bezoar*
Rapana rapiformis	GU188190	*Rapana rapiformis*	correct	correct	correct	correct	correct	correct	*Rapana bezoar*
Rapana rapiformis	GU188191	*Rapana rapiformis*	correct	correct	correct	correct	correct	correct	*Rapana bezoar*
Rapana rapiformis	GU188192	*Rapana rapiformis*	correct	correct	correct	correct	correct	correct	*Rapana bezoar*
Rapana rapiformis	GU188193	*Rapana rapiformis*	correct	correct	correct	correct	correct	correct	*Rapana bezoar*
Rapana rapiformis	GU188194	*Rapana rapiformis*	correct	correct	correct	correct	correct	correct	*Rapana bezoar*
Rapana venosa	GU188175	*Rapana venosa*	no id	correct	correct	no id	correct	correct	*Lataxiena blosvillei*
Rapana venosa	GU188176	*Rapana venosa*	correct	correct	correct	correct	correct	correct	*Lataxiena blosvillei*
Rapana venosa	GU188177	*Rapana venosa*	correct	correct	correct	correct	correct	correct	*Lataxiena blosvillei*
Rapana venosa	GU188178	*Rapana venosa*	correct	correct	correct	correct	correct	correct	*Lataxiena blosvillei*
Rapana venosa	GU188179	*Rapana venosa*	correct	correct	correct	correct	correct	correct	*Lataxiena blosvillei*
Rapana venosa	HE584367	*Rapana venosa*	correct	correct	correct	correct	correct	correct	*Lataxiena blosvillei*
Rapana venosa	HM180814	*Rapana venosa*	correct	correct	correct	correct	correct	correct	*Lataxiena blosvillei*
Rapana venosa	HM180815	*Rapana venosa*	correct	correct	correct	correct	correct	correct	*Lataxiena blosvillei*
Rapana venosa	HM180816	*Rapana venosa*	correct	correct	correct	correct	correct	correct	*Lataxiena blosvillei*

Rhinoclavis sinensis	JF693360	*Rhinoclavis sinensis*	correct	correct	correct	correct	correct	correct	*Batillaria cumingii*
Rhinoclavis sinensis	JF693361	*Rhinoclavis sinensis*	correct	correct	correct	correct	correct	correct	*Batillaria cumingii*
Rhinoclavis sinensis	JF693362	*Rhinoclavis sinensis*	correct	correct	correct	correct	correct	correct	*Batillaria cumingii*
Roboastra gracilis	EF142863	*Roboastra luteolineata*	no id	no id	no id	no id	no id	no id	*Roboastra ernsti*
Roboastra luteolineata	EF142861	*Roboastra gracilis*	no id	no id	no id	no id	no id	no id	*Roboastra ernsti*
Rossiteria nuclea	AB505312	*Tonna olearium*	no id	no id	no id	no id	no id	no id	*Trochidae sp.*
Ruditapes philippinarum	HQ703304	*Ruditapes philippinarum*	ambiguous	ambiguous	ambiguous	ambiguous	ambiguous	ambiguous	*Macridiscus semicancellata*
Ruditapes philippinarum	HQ703305	*Ruditapes philippinarum*	correct	correct	correct	correct	ambiguous	ambiguous	*Macridiscus semicancellata*
Ruditapes philippinarum	HQ703306	*Ruditapes philippinarum*	ambiguous	ambiguous	ambiguous	ambiguous	ambiguous	ambiguous	*Macridiscus semicancellata*
Ruditapes philippinarum	HQ703307	*Ruditapes philippinarum*	ambiguous	ambiguous	ambiguous	ambiguous	ambiguous	ambiguous	*Macridiscus semicancellata*
Ruditapes philippinarum	HQ703308	*Ruditapes philippinarum*	no id	ambiguous	ambiguous	no id	ambiguous	ambiguous	*Macridiscus semicancellata*
Ruditapes philippinarum	HQ703309	*Ruditapes philippinarum*	ambiguous	ambiguous	ambiguous	ambiguous	ambiguous	ambiguous	*Macridiscus semicancellata*
Ruditapes philippinarum	HQ703310	*Ruditapes philippinarum*	ambiguous	ambiguous	ambiguous	ambiguous	ambiguous	ambiguous	*Macridiscus semicancellata*
Ruditapes philippinarum	HQ703311	*Ruditapes philippinarum*	ambiguous	ambiguous	ambiguous	ambiguous	ambiguous	ambiguous	*Macridiscus semicancellata*
Ruditapes philippinarum	GQ855263	*Ruditapes philippinarum*	ambiguous	ambiguous	ambiguous	ambiguous	ambiguous	ambiguous	*Macridiscus semicancellata*
Ruditapes philippinarum	GQ855264	*Ruditapes philippinarum*	correct	correct	correct	correct	ambiguous	ambiguous	*Macridiscus semicancellata*
Ruditapes philippinarum	GQ855265	*Ruditapes philippinarum*	ambiguous	ambiguous	ambiguous	ambiguous	ambiguous	ambiguous	*Macridiscus semicancellata*
Ruditapes philippinarum	GQ855266	*Ruditapes philippinarum*	ambiguous	ambiguous	ambiguous	ambiguous	ambiguous	ambiguous	*Macridiscus semicancellata*
Ruditapes philippinarum	GQ855267	*Ruditapes philippinarum*	ambiguous	ambiguous	ambiguous	ambiguous	ambiguous	ambiguous	*Macridiscus semicancellata*
Ruditapes philippinarum	JN898947	*Ruditapes philippinarum*	ambiguous	ambiguous	ambiguous	ambiguous	ambiguous	ambiguous	*Macridiscus semicancellata*
Ruditapes philippinarum	HM124595	*Ruditapes philippinarum*	ambiguous	ambiguous	ambiguous	ambiguous	ambiguous	ambiguous	*Macridiscus semicancellata*
Ruditapes philippinarum	AB244389	*Ruditapes philippinarum*	no id	correct	correct	no id	correct	ambiguous	*Macridiscus semicancellata*
Ruditapes philippinarum	AB244390	*Ruditapes philippinarum*	ambiguous	ambiguous	ambiguous	ambiguous	ambiguous	ambiguous	*Macridiscus semicancellata*
Ruditapes philippinarum	AB244391	*Ruditapes philippinarum*	ambiguous	ambiguous	ambiguous	ambiguous	ambiguous	ambiguous	*Macridiscus semicancellata*
Ruditapes philippinarum	AB244392	*Ruditapes philippinarum*	ambiguous	ambiguous	ambiguous	ambiguous	ambiguous	ambiguous	*Macridiscus semicancellata*
Ruditapes philippinarum	AB244393	*Ruditapes philippinarum*	ambiguous	ambiguous	ambiguous	ambiguous	ambiguous	ambiguous	*Macridiscus semicancellata*
Ruditapes philippinarum	AB244394	*Ruditapes philippinarum*	correct	correct	correct	correct	ambiguous	ambiguous	*Macridiscus semicancellata*
Ruditapes philippinarum	AB244395	*Ruditapes philippinarum*	ambiguous	ambiguous	ambiguous	ambiguous	ambiguous	ambiguous	*Macridiscus semicancellata*
Ruditapes philippinarum	AB244396	*Ruditapes philippinarum*	correct	correct	correct	correct	ambiguous	ambiguous	*Macridiscus semicancellata*
Ruditapes philippinarum	AB244397	*Ruditapes philippinarum*	ambiguous	ambiguous	ambiguous	ambiguous	ambiguous	ambiguous	*Macridiscus semicancellata*
Ruditapes philippinarum	AB244398	*Ruditapes philippinarum*	ambiguous	ambiguous	ambiguous	ambiguous	ambiguous	ambiguous	*Macridiscus semicancellata*

Ruditapes philippinarum	AB244399	*Ruditapes philippinarum*	ambiguous	ambiguous	ambiguous	ambiguous	ambiguous	ambiguous	*Macridiscus semicancellata*
Ruditapes philippinarum	AB244400	*Ruditapes philippinarum*	ambiguous	ambiguous	ambiguous	ambiguous	ambiguous	ambiguous	*Macridiscus semicancellata*
Ruditapes philippinarum	AB244401	*Ruditapes philippinarum*	no id	ambiguous	ambiguous	no id	ambiguous	ambiguous	*Macridiscus semicancellata*
Ruditapes philippinarum	HM180833	*Ruditapes philippinarum*	ambiguous	ambiguous	ambiguous	ambiguous	ambiguous	ambiguous	*Macridiscus semicancellata*
Ruditapes philippinarum	HM180834	*Ruditapes philippinarum*	correct	correct	correct	correct	ambiguous	ambiguous	*Macridiscus semicancellata*
Ruditapes variegata	GQ855270	*Ruditapes variegata*	incorrect	incorrect	incorrect	incorrect	incorrect	incorrect	*Macridiscus semicancellata*
Ruditapes variegata	HQ703312	*Ruditapes variegata*	correct	correct	correct	correct	correct	correct	*Ruditapes variegatus*
Ruditapes variegata	HQ703313	*Ruditapes variegata*	correct	correct	correct	correct	correct	correct	*Ruditapes variegatus*
Ruditapes variegata	HQ703314	*Ruditapes variegata*	correct	correct	correct	correct	correct	correct	*Ruditapes variegatus*
Ruditapes variegata	HQ703315	*Ruditapes variegata*	correct	correct	correct	correct	correct	correct	*Ruditapes variegatus*
Ruditapes variegata	HQ703316	*Ruditapes variegata*	correct	correct	correct	correct	correct	correct	*Ruditapes variegatus*
Ruditapes variegata	HQ703317	*Ruditapes variegata*	correct	correct	correct	correct	correct	correct	*Ruditapes variegatus*
Ruditapes variegata	GQ855268	*Ruditapes variegata*	correct	correct	correct	correct	correct	correct	*Ruditapes variegatus*
Ruditapes variegata	GQ855269	*Ruditapes variegata*	correct	correct	correct	correct	correct	correct	*Ruditapes variegatus*
Ruditapes variegata	GQ855271	*Ruditapes variegata*	correct	correct	correct	correct	correct	correct	*Ruditapes variegatus*
Saccostrea mordax	HQ661025	*Saccostrea mordax*	correct	correct	correct	correct	correct	correct	*Saccostrea cucullata*
Saccostrea mordax	HQ661026	*Saccostrea mordax*	correct	correct	correct	correct	correct	correct	*Saccostrea cucullata*
Saccostrea mordax	HQ661027	*Saccostrea mordax*	correct	correct	correct	correct	correct	correct	*Saccostrea cucullata*
Saccostrea mordax	HQ661028	*Saccostrea mordax*	correct	correct	correct	correct	correct	correct	*Saccostrea cucullata*
Saccostrea mordax	HQ661029	*Saccostrea mordax*	correct	correct	correct	correct	correct	correct	*Saccostrea cucullata*
Sagamilepeta sagamiensis	AB543981	*Sagamilepeta sagamiensis*	correct	correct	correct	correct	correct	correct	*Lepeta caeca*
Sagamilepeta sagamiensis	AB543982	*Sagamilepeta sagamiensis*	correct	correct	correct	correct	correct	correct	*Lepeta caeca*
Soletellina diphos	JN859948	*Donax dysoni*	no id	no id	no id	no id	no id	no id	*Psammotaea sp.*
Psammotaea elongata	JN859936	*Psammotaea elongata*	correct	correct	correct	correct	correct	correct	*Semele rubropicta*
Psammotaea elongata	JN859938	*Psammotaea elongata*	correct	correct	correct	correct	correct	correct	*Semele rubropicta*
Psammotaea elongata	JN859940	*Psammotaea elongata*	correct	correct	correct	correct	correct	correct	*Semele rubropicta*
Psammotaea elongata	JN859941	*Psammotaea elongata*	correct	correct	correct	correct	correct	correct	*Semele rubropicta*
Nuttallia olivacea	JN859954	*Nuttallia olivacea*	no id	correct	correct	no id	correct	correct	*Psammotaea sp.*
Nuttallia olivacea	JN859955	*Nuttallia olivacea*	no id	correct	correct	no id	correct	correct	*Psammotaea sp.*
Sanguinolaria tchangsii	JN859945	*Sanguinolaria tchangsii*	correct	correct	correct	correct	correct	correct	*Tellina carpenteri*
Sanguinolaria tchangsii	JN859947	*Sanguinolaria tchangsii*	correct	correct	correct	correct	correct	correct	*Tellina carpenteri*

Soletellina virescens	JN859943	*Sanguinolaria olivacea*	no id	no id	no id	no id	no id	no id	*Soletellina chinensis*
Saxidomus purpuratus	HQ703041	*Saxidomus purpurata*	correct	correct	correct	correct	correct	correct	*Saxidomus gigantea*
Saxidomus purpuratus	HQ703042	*Saxidomus purpurata*	correct	correct	correct	correct	correct	correct	*Saxidomus gigantea*
Saxidomus purpuratus	HQ703043	*Saxidomus purpurata*	correct	correct	correct	correct	correct	correct	*Saxidomus gigantea*
Saxidomus purpuratus	HQ703044	*Saxidomus purpurata*	correct	correct	correct	correct	correct	correct	*Saxidomus gigantea*
Saxidomus purpuratus	HQ703045	*Saxidomus purpurata*	correct	correct	correct	correct	correct	correct	*Saxidomus gigantea*
Saxidomus purpuratus	HQ703046	*Saxidomus purpurata*	no id	correct	correct	no id	correct	correct	*Saxidomus gigantea*
Saxidomus purpuratus	HQ703047	*Saxidomus purpurata*	correct	correct	correct	correct	correct	correct	*Saxidomus gigantea*
Saxidomus purpuratus	KP419933	*Saxidomus purpurata*	correct	correct	correct	correct	correct	correct	*Saxidomus gigantea*
Saxidomus purpuratus	JN898951	*Saxidomus purpurata*	correct	correct	correct	correct	correct	correct	*Saxidomus gigantea*
Saxidomus purpuratus	HM124571	*Saxidomus purpurata*	correct	correct	correct	correct	correct	correct	*Saxidomus gigantea*
Saxidomus purpuratus	EU118007	*Saxidomus purpurata*	correct	correct	correct	correct	correct	correct	*Saxidomus gigantea*
Scapharca broughtonii	AB050894	*Scapharca broughtonii*	correct	correct	correct	correct	correct	correct	*Scapharca broughtonii*
Scapharca broughtonii	AB729113	*Scapharca broughtonii*	correct	correct	correct	correct	correct	correct	*Scapharca broughtonii*
Scapharca broughtonii	KU341869	*Scapharca broughtonii*	correct	correct	correct	correct	correct	correct	
Scapharca broughtonii	KU341870	*Scapharca broughtonii*	correct	correct	correct	correct	correct	correct	
Scapharca broughtonii	KU341871	*Scapharca broughtonii*	correct	correct	correct	correct	correct	correct	
Scapharca broughtonii	KU341872	*Scapharca broughtonii*	correct	correct	correct	correct	correct	correct	
Scapharca broughtonii	KU341873	*Scapharca broughtonii*	correct	correct	correct	correct	correct	correct	
Scapharca cornea	KU341858	*Scapharca cornea*	correct	correct	correct	correct	correct	correct	
Scapharca cornea	KU341859	*Scapharca cornea*	correct	correct	correct	correct	correct	correct	
Scapharca cornea	KU341860	*Scapharca cornea*	correct	correct	correct	correct	correct	correct	
Scapharca cornea	KU341861	*Scapharca cornea*	correct	correct	correct	correct	correct	correct	
Scapharca cornea	KU341862	*Scapharca cornea*	correct	correct	correct	correct	correct	correct	
Scapharca cornea	KU341863	*Scapharca cornea*	correct	correct	correct	correct	correct	correct	
Scapharca cornea	KU341864	*Scapharca cornea*	correct	correct	correct	correct	correct	correct	
Scapharca globosa	KU341846	*Scapharca globosa*	correct	correct	correct	correct	correct	correct	
Scapharca globosa	KU341847	*Scapharca globosa*	correct	correct	correct	correct	correct	correct	
Scapharca globosa	KU341848	*Scapharca globosa*	correct	correct	correct	correct	correct	correct	
Scapharca globosa	KU341849	*Scapharca globosa*	correct	correct	correct	correct	correct	correct	
Scapharca globosa	KU341850	*Scapharca globosa*	correct	correct	correct	correct	correct	correct	

Scapharca globosa	KU341851	*Scapharca globosa*	correct	correct	correct	correct	correct	correct	
Scapharca globosa	KU341852	*Scapharca globosa*	correct	correct	correct	correct	correct	correct	
Scapharca globosa	KU341853	*Scapharca globosa*	correct	correct	correct	correct	correct	correct	
Scapharca globosa	AB254194	*Scapharca globosa*	no id	no id	correct	no id	no id	correct	*Diluvarca ferruginea*
Scapharca gubernaculum	KU341854	*Scapharca gubernaculum*	correct	correct	correct	correct	correct	correct	
Scapharca gubernaculum	KU341855	*Scapharca gubernaculum*	correct	correct	correct	correct	correct	correct	
Scapharca gubernaculum	KU341856	*Scapharca gubernaculum*	correct	correct	correct	correct	correct	correct	
Scapharca gubernaculum	KU341857	*Scapharca gubernaculum*	correct	correct	correct	correct	correct	correct	
Scapharca inaequivalvis	KU341865	*Scapharca inaequivalvis*	correct	correct	correct	correct	correct	correct	
Scapharca inaequivalvis	KU341866	*Scapharca inaequivalvis*	correct	correct	correct	correct	correct	correct	
Scapharca inaequivalvis	KU341867	*Scapharca inaequivalvis*	correct	correct	correct	correct	correct	correct	
Scapharca inaequivalvis	KU341868	*Scapharca inaequivalvis*	correct	correct	correct	correct	correct	correct	
Scapharca inaequivalvis	AB076937	*Scapharca inaequivalvis*	correct	correct	correct	correct	correct	correct	*Scapharca broughtonii*
Scapharca kagoshimensis	KU341874	*Scapharca kagoshimensis*	correct	correct	correct	correct	correct	correct	
Scapharca kagoshimensis	KU341875	*Scapharca kagoshimensis*	correct	correct	correct	correct	correct	correct	
Scapharca kagoshimensis	KU341876	*Scapharca kagoshimensis*	correct	correct	correct	correct	correct	correct	
Scapharca kagoshimensis	KU341877	*Scapharca kagoshimensis*	correct	correct	correct	correct	correct	correct	
Scapharca kagoshimensis	KU341878	*Scapharca kagoshimensis*	correct	correct	correct	correct	correct	correct	
Scapharca kagoshimensis	KU341879	*Scapharca kagoshimensis*	correct	correct	correct	correct	correct	correct	
Scapharca kagoshimensis	KU341880	*Scapharca kagoshimensis*	correct	correct	correct	correct	correct	correct	
Scapharca kagoshimensis	AB854392	*Scapharca kagoshimensis*	correct	correct	correct	correct	correct	correct	*Scapharca broughtonii*
Scapharca kagoshimensis	AB854393	*Scapharca kagoshimensis*	correct	correct	correct	correct	correct	correct	*Scapharca broughtonii*
Scapharca kagoshimensis	AB854394	*Scapharca kagoshimensis*	correct	correct	correct	correct	correct	correct	*Scapharca broughtonii*
Scapharca kagoshimensis	AB854395	*Scapharca kagoshimensis*	no id	correct	correct	no id	correct	correct	*Scapharca broughtonii*
Scapharca kagoshimensis	AB854396	*Scapharca kagoshimensis*	correct	correct	correct	correct	correct	correct	*Scapharca broughtonii*
Scapharca kagoshimensis	AB854397	*Scapharca kagoshimensis*	correct	correct	correct	correct	correct	correct	*Scapharca broughtonii*
Scapharca kagoshimensis	AB854398	*Scapharca kagoshimensis*	correct	correct	correct	correct	correct	correct	*Scapharca broughtonii*
Scapharca kagoshimensis	AB854399	*Scapharca kagoshimensis*	correct	correct	correct	correct	correct	correct	*Scapharca broughtonii*
Scapharca kagoshimensis	AB854400	*Scapharca kagoshimensis*	correct	correct	correct	correct	correct	correct	*Scapharca broughtonii*
Scapharca kagoshimensis	AB854401	*Scapharca kagoshimensis*	correct	correct	correct	correct	correct	correct	*Scapharca broughtonii*
Scapharca kagoshimensis	AB854402	*Scapharca kagoshimensis*	correct	correct	correct	correct	correct	correct	*Scapharca broughtonii*

Scapharca kagoshimensis	AB854403	*Scapharca kagoshimensis*	correct	correct	correct	correct	correct	correct	*Scapharca broughtonii*
Scapharca kagoshimensis	AB854404	*Scapharca kagoshimensis*	correct	correct	correct	correct	correct	correct	*Scapharca broughtonii*
Scapharca kagoshimensis	AB854405	*Scapharca kagoshimensis*	no id	no id	correct	no id	no id	correct	*Scapharca broughtonii*
Scapharca satowi	AB050898	*Scapharca inaequivalvis*	no id	no id	no id	no id	no id	no id	*Scapharca broughtonii*
Scutellastra flexuosa	AB238583	*Scutellastra optima*	no id	no id	no id	no id	no id	no id	*Scutellastra optima*
Scutellastra optima	AB238585	*Scutellastra flexuosa*	no id	no id	no id	no id	no id	no id	*Scutellastra flexuosa*
Semele cf amabilis	JN859975	*Sanguinolaria olivacea*	no id	no id	no id	no id	no id	no id	*Soletellina chinensis*
Semele scaba	JN859973	*Semele scabra*	correct	correct	correct	correct	correct	correct	*Semele cf. amabilis*
Semele scaba	JN859974	*Semele scabra*	correct	correct	correct	correct	correct	correct	*Semele cf. amabilis*
Sepia aculeata	HQ846106	*Sepia aculeata*	correct	correct	correct	correct	correct	correct	*Sepiida sp.*
Sepia aculeata	HQ846107	*Sepia aculeata*	correct	correct	correct	correct	correct	correct	*Sepiida sp.*
Sepia aculeata	HQ846108	*Sepia aculeata*	correct	correct	correct	correct	correct	correct	*Sepiida sp.*
Sepia aculeata	HQ846083	*Sepia aculeata*	correct	correct	correct	correct	correct	correct	*Sepiida sp.*
Sepia andreana	AB430401	*Sepia kobiensis*	no id	no id	incorrect	no id	no id	incorrect	*Sepia hirunda*
Sepia aureomaculata	AB430402	*Sepia lorigera*	no id	incorrect	incorrect	no id	incorrect	incorrect	*Sepia sp.*
Sepia esculenta	HQ846084	*Sepia esculenta*	correct	correct	correct	correct	correct	correct	*Sepia esculenta*
Sepia esculenta	HQ846085	*Sepia esculenta*	correct	correct	correct	correct	correct	correct	*Sepia esculenta*
Sepia esculenta	HQ846086	*Sepia esculenta*	correct	correct	correct	correct	correct	correct	*Sepia esculenta*
Sepia esculenta	HQ846089	*Sepia esculenta*	correct	correct	correct	correct	correct	correct	*Sepia esculenta*
Sepia esculenta	HQ846087	*Sepia esculenta*	correct	correct	correct	correct	correct	correct	*Sepia esculenta*
Sepia esculenta	HQ846088	*Sepia esculenta*	correct	correct	correct	correct	correct	correct	*Sepia esculenta*
Sepia esculenta	HQ846091	*Sepia esculenta*	correct	correct	correct	correct	correct	correct	*Sepia esculenta*
Sepia esculenta	HQ846090	*Sepia esculenta*	correct	correct	correct	correct	correct	correct	*Sepia esculenta*
Sepia esculenta	AB192335	*Sepia esculenta*	no id	no id	no id	no id	no id	no id	*Sepia esculenta*
Sepia kobiensis	AB193813	*Sepia andreana*	no id	no id	incorrect	no id	no id	incorrect	*Sepia furcata*
Sepia latimanus	AB192338	*Sepia esculenta*	no id	no id	no id	no id	no id	no id	*Sepiida sp.*
Sepia lorigera	AB193810	*Sepia aureomaculata*	no id	incorrect	incorrect	no id	incorrect	incorrect	*Sepia aureomaculata*
Sepia lycidas	HQ846109	*Sepia lycidas*	correct	correct	correct	correct	correct	correct	*Sepia pharaonis*
Sepia lycidas	AB192337	*Sepia lycidas*	correct	correct	correct	correct	correct	correct	*Sepia pharaonis*
Sepia lycidas	AB675088	*Sepia lycidas*	correct	correct	correct	correct	correct	correct	*Sepia pharaonis*
Sepia madokai	AB430407	*Sepiola birostrata*	no id	no id	no id	no id	no id	no id	*Sepia lorigera*

Sepia pardex	AB193809	*Sepia aureomaculata*	no id	no id	no id	no id	no id	no id	*Sepia aureomaculata*
Sepia peterseni	AB192339	*Sepia andreana*	no id	no id	incorrect	no id	no id	incorrect	*Sepia andreana*
Sepia pharaonis	HQ846093	*Sepia pharaonis*	correct	correct	correct	correct	correct	correct	*Sepia pharaonis*
Sepia pharaonis	JN315869	*Sepia pharaonis*	correct	correct	correct	correct	correct	correct	*Sepia pharaonis*
Sepia pharaonis	JN315870	*Sepia pharaonis*	correct	correct	correct	correct	correct	correct	*Sepia pharaonis*
Sepia pharaonis	JN315871	*Sepia pharaonis*	correct	correct	correct	correct	correct	correct	*Sepia pharaonis*
Sepia pharaonis	JN315872	*Sepia pharaonis*	correct	correct	correct	correct	correct	correct	*Sepia pharaonis*
Sepia pharaonis	JN315873	*Sepia pharaonis*	correct	correct	correct	correct	correct	correct	*Sepia pharaonis*
Sepia pharaonis	JN315874	*Sepia pharaonis*	correct	correct	correct	correct	correct	correct	*Sepia pharaonis*
Sepia recurvirostra	HQ846092	*Sepia recurvirostra*	correct	correct	correct	correct	correct	correct	*Sepia recurvirostra*
Sepia recurvirostra	HQ846161	*Sepia recurvirostra*	correct	correct	correct	correct	correct	correct	*Sepia recurvirostra*
Sepia recurvirostra	HQ846162	*Sepia recurvirostra*	correct	correct	correct	correct	correct	correct	*Sepia recurvirostra*
Sepia subtenuipes	AB430414	*Octopus oliveri*	no id	no id	incorrect	no id	no id	incorrect	*Octopus conispadiceus*
Sepia tenuipes	AB430411	*Sepia lycidas*	no id	no id	no id	no id	no id	no id	*Sepia bertheloti*
Sepia tokioensis	AB430412	*Sepia andreana*	no id	no id	incorrect	no id	no id	incorrect	*Sepia andreana*
Sepiella inermis	HQ846080	*Sepiella inermis*	no id	correct	correct	no id	correct	correct	*Sepiella inermis*
Sepiella inermis	HQ846081	*Sepiella inermis*	no id	correct	correct	no id	correct	correct	*Sepiella inermis*
Sepiella maindroni	AB192341	*Sepiella inermis*	correct	correct	correct	correct	ambiguous	ambiguous	*Sepiella inermis*
Sepiella maindroni	AF346853	*Sepiella inermis*	correct	correct	correct	correct	ambiguous	ambiguous	*Sepiella inermis*
Sepiella japonica	HQ846078	*Sepiella japonica*	correct	correct	correct	correct	ambiguous	ambiguous	*Sepiella inermis*
Sepiella japonica	HQ846079	*Sepiella japonica*	correct	correct	correct	correct	ambiguous	ambiguous	*Sepiella inermis*
Sepiella japonica	HQ846082	*Sepiella japonica*	correct	correct	correct	correct	ambiguous	ambiguous	*Sepiella inermis*
Sepiola birostrata	HQ846094	*Sepiola birostrata*	correct	correct	correct	correct	correct	correct	*Sepiola pfefferi*
Sepiola birostrata	HQ846095	*Sepiola birostrata*	correct	correct	correct	correct	correct	correct	*Sepiola pfefferi*
Sepiola birostrata	HQ846096	*Sepiola birostrata*	correct	correct	correct	correct	correct	correct	*Sepiola pfefferi*
Sepiola birostrata	HQ846097	*Sepiola birostrata*	correct	correct	correct	correct	correct	correct	*Sepiola pfefferi*
Sepiola birostrata	HQ846098	*Sepiola birostrata*	correct	correct	correct	correct	correct	correct	*Sepiola pfefferi*
Sepiolina nipponensis	AB591073	*Architeuthis dux*	no id	no id	no id	no id	no id	no id	*Stoloteuthis japonica*
Sepiolina petasa	AB591071	*Octopus longispadiceus*	no id	no id	no id	no id	no id	no id	*Sepiola pfefferi*
Sepioteuthis lessoniana	AB986213	*Sepioteuthis lessoniana*	correct	correct	correct	correct	correct	correct	*Sepioteuthis cf. lessoniana*
Sepioteuthis lessoniana	AB986214	*Sepioteuthis lessoniana*	correct	correct	correct	correct	correct	correct	*Sepioteuthis cf. lessoniana*

Sepioteuthis lessoniana	AB986215	*Sepioteuthis lessoniana*	correct	correct	correct	correct	correct	correct	*Sepioteuthis cf. lessoniana*
Sepioteuthis lessoniana	AB986216	*Sepioteuthis lessoniana*	correct	correct	correct	correct	correct	correct	*Sepioteuthis cf. lessoniana*
Sepioteuthis lessoniana	AB986217	*Sepioteuthis lessoniana*	correct	correct	correct	correct	correct	correct	*Sepioteuthis cf. lessoniana*
Sepioteuthis lessoniana	AB986218	*Sepioteuthis lessoniana*	correct	correct	correct	correct	correct	correct	*Sepioteuthis cf. lessoniana*
Sepioteuthis lessoniana	AB986219	*Sepioteuthis lessoniana*	correct	correct	correct	correct	correct	correct	*Sepioteuthis cf. lessoniana*
Sepioteuthis lessoniana	AB986220	*Sepioteuthis lessoniana*	correct	correct	correct	correct	correct	correct	*Sepioteuthis cf. lessoniana*
Sepioteuthis lessoniana	HQ529542	*Sepioteuthis lessoniana*	correct	correct	correct	correct	correct	correct	*Sepioteuthis cf. lessoniana*
Sepioteuthis lessoniana	HQ529541	*Sepioteuthis lessoniana*	correct	correct	correct	correct	correct	correct	*Sepioteuthis cf. lessoniana*
Sepioteuthis lessoniana	HQ529540	*Sepioteuthis lessoniana*	correct	correct	correct	correct	correct	correct	*Sepioteuthis cf. lessoniana*
Sepioteuthis lessoniana	EU349466	*Sepioteuthis lessoniana*	correct	correct	correct	correct	correct	correct	*Sepioteuthis cf. lessoniana*
Sepioteuthis lessoniana	AB986221	*Sepioteuthis lessoniana*	correct	correct	correct	correct	correct	correct	*Sepioteuthis cf. lessoniana*
Sepioteuthis lessoniana	AB986222	*Sepioteuthis lessoniana*	correct	correct	correct	correct	correct	correct	*Sepioteuthis cf. lessoniana*
Sepioteuthis lessoniana	AB986223	*Sepioteuthis lessoniana*	correct	correct	correct	correct	correct	correct	*Sepioteuthis cf. lessoniana*
Sepioteuthis lessoniana	AB986224	*Sepioteuthis lessoniana*	correct	correct	correct	correct	correct	correct	*Sepioteuthis cf. lessoniana*
Sepioteuthis lessoniana	AB986225	*Sepioteuthis lessoniana*	correct	correct	correct	correct	correct	correct	*Sepioteuthis cf. lessoniana*
Sepioteuthis lessoniana	AB986226	*Sepioteuthis lessoniana*	correct	correct	correct	correct	correct	correct	*Sepioteuthis cf. lessoniana*
Serratina capsoides	AB691947	*Serratina capsoides*	correct	correct	correct	correct	correct	correct	
Serratina capsoides	AB691946	*Serratina capsoides*	no id	correct	correct	no id	correct	correct	
Serratina capsoides	AB691945	*Serratina capsoides*	correct	correct	correct	correct	correct	correct	
Serratina capsoides	AB691950	*Serratina capsoides*	ambiguous	ambiguous	ambiguous	ambiguous	ambiguous	ambiguous	*Solecurtus divaricatus*
Serratina capsoides	AB691949	*Serratina capsoides*	ambiguous	ambiguous	ambiguous	ambiguous	ambiguous	ambiguous	*Solecurtus divaricatus*
Serratina capsoides	AB691948	*Serratina capsoides*	ambiguous	ambiguous	ambiguous	ambiguous	ambiguous	ambiguous	*Solecurtus divaricatus*
Serratina capsoides	JN859959	*Serratina capsoides*	ambiguous	ambiguous	ambiguous	ambiguous	ambiguous	ambiguous	*Solecurtus divaricatus*
Serratina capsoides	JN859960	*Serratina capsoides*	ambiguous	ambiguous	ambiguous	ambiguous	ambiguous	ambiguous	*Solecurtus divaricatus*
Siliqua radiata	JN860002	*Siliqua radiata*	correct	correct	correct	correct	correct	correct	*Siliqua radiata*
Siliqua radiata	JN860003	*Siliqua radiata*	correct	correct	correct	correct	correct	correct	*Siliqua radiata*
Siliqua radiata	JN860004	*Siliqua radiata*	correct	correct	correct	correct	correct	correct	*Siliqua radiata*
Siliqua radiata	JN860005	*Siliqua radiata*	no id	correct	correct	no id	correct	correct	*Siliqua radiata*
Sinonovacula constricta	JN859986	*Sinonovacula constricta*	correct	correct	correct	correct	correct	correct	*Sinonovacula constricta*
Sinonovacula constricta	JN859987	*Sinonovacula constricta*	correct	correct	correct	correct	correct	correct	*Sinonovacula constricta*
Sinonovacula constricta	JN859988	*Sinonovacula constricta*	correct	correct	correct	correct	correct	correct	*Sinonovacula constricta*

Sinonovacula constricta	JN859989	*Sinonovacula constricta*	correct	correct	correct	correct	correct	correct	*Sinonovacula constricta*
Sinonovacula constricta	JN859991	*Sinonovacula constricta*	correct	correct	correct	correct	correct	correct	*Sinonovacula constricta*
Sinonovacula constricta	JN859993	*Sinonovacula constricta*	correct	correct	correct	correct	correct	correct	*Sinonovacula constricta*
Sinonovacula constricta	HM180885	*Sinonovacula constricta*	correct	correct	correct	correct	correct	correct	*Sinonovacula constricta*
Sinonovacula constricta	HM180886	*Sinonovacula constricta*	correct	correct	correct	correct	correct	correct	*Sinonovacula constricta*
Siphonaria japonica	KF716648	*Siphonaria japonica*	no id	correct	correct	no id	correct	correct	*Limax cf. cinereoniger*
Siphonaria japonica	KF716623	*Siphonaria japonica*	correct	correct	correct	correct	correct	correct	*Limax cf. cinereoniger*
Siphonaria japonica	KF716594	*Siphonaria japonica*	correct	correct	correct	correct	correct	correct	*Limax cf. cinereoniger*
Siphonaria japonica	KF716568	*Siphonaria japonica*	correct	correct	correct	correct	correct	correct	*Limax cf. cinereoniger*
Siphonaria japonica	KF716536	*Siphonaria japonica*	correct	correct	correct	correct	correct	correct	*Limax cf. cinereoniger*
Siphonaria japonica	KF716747	*Siphonaria japonica*	correct	correct	correct	correct	correct	correct	*Limax cf. cinereoniger*
Siphonaria japonica	KF716745	*Siphonaria japonica*	no id	correct	correct	no id	correct	correct	*Limax cf. cinereoniger*
Siphonaria japonica	KF716718	*Siphonaria japonica*	correct	correct	correct	correct	correct	correct	*Limax cf. cinereoniger*
Siphonaria japonica	KF716717	*Siphonaria japonica*	correct	correct	correct	correct	correct	correct	*Limax cf. cinereoniger*
Siphonaria japonica	KF716701	*Siphonaria japonica*	correct	correct	correct	correct	correct	correct	*Limax cf. cinereoniger*
Siphonaria japonica	KF716700	*Siphonaria japonica*	correct	correct	correct	correct	correct	correct	*Limax cf. cinereoniger*
Siphonaria japonica	KF716679	*Siphonaria japonica*	correct	correct	correct	correct	correct	correct	*Limax cf. cinereoniger*
Solariella nyssonus	HF586294	*Solariella nyssonus*	correct	correct	correct	correct	correct	correct	*Minolia sp.*
Solariella nyssonus	HF586295	*Solariella nyssonus*	correct	correct	correct	correct	correct	correct	*Minolia sp.*
Solecurtus divaricatus	JN859983	*Solecurtus divaricatus*	no id	no id	no id	no id	no id	no id	*Solecurtus divaricatus*
Solen grandis	JN860010	*Solen grandis*	no id	no id	no id	no id	no id	no id	*Solen regularis*
Solen grandis	JN860011	*Solen grandis*	correct	correct	correct	correct	correct	correct	*Solen sarawakensis*
Solen grandis	JN860012	*Solen grandis*	correct	correct	correct	correct	correct	correct	*Solen sarawakensis*
Solen strictus	JN860006	*Solen strictus*	correct	correct	correct	correct	correct	correct	*Solen strictus*
Solen strictus	JN860007	*Solen strictus*	correct	correct	correct	correct	correct	correct	*Solen strictus*
Solen strictus	JN860008	*Solen strictus*	no id	correct	correct	no id	correct	correct	*Solen strictus*
Solen strictus	JN860009	*Solen strictus*	no id	no id	no id	no id	no id	no id	*Solen strictus*
Stiliger ornatus	AB501311	*Elysia ornata*	no id	no id	no id	no id	no id	no id	*Stiliger ornatus*
Stiliger smaragdinus	AB501310	*Thuridilla gracilis*	no id	no id	no id	no id	no id	no id	*Elysia translucens*
Stomatella impertusa	AB505292	*Stomatia phymotis*	no id	no id	no id	no id	no id	no id	*Stomatella impertusa*
Stomatella planulata	AB505293	*Stomatella planulata*	correct	correct	correct	correct	correct	correct	*Stomatella planulata*

Stomatella planulata	EU530132	*Stomatella planulata*	correct	correct	correct	correct	correct	correct	*Stomatella planulata*
Stomatella planulata	EU530131	*Stomatella planulata*	correct	correct	correct	correct	correct	correct	*Stomatella planulata*
Stomatella planulata	EU530130	*Stomatella planulata*	correct	correct	correct	correct	correct	correct	*Stomatella planulata*
Stomatia obscura	AB505294	*Littorina brevicula*	no id	no id	no id	no id	no id	no id	*Stomatia phymotis*
Stomatia phymotis	AB365223	*Stomatella impertusa*	no id	no id	no id	no id	no id	no id	*Stomatia phymotis*
Strombus lentiginosu	JF693421	*Strombus lentiginosus*	correct	correct	correct	correct	ambiguous	ambiguous	*Volutidae sp.*
Strombus lentiginosu	JF693422	*Strombus lentiginosus*	correct	correct	correct	correct	ambiguous	ambiguous	*Volutidae sp.*
Strombus mutabiis	JF693420	*Strombus lentiginosus*	no id	incorrect	incorrect	no id	incorrect	incorrect	*Volutidae sp.*
Strombus luhuanus	JF693429	*Strombus luhuanus*	correct	correct	correct	correct	correct	correct	*Strombus persicus*
Strombus luhuanus	JF693430	*Strombus luhuanus*	correct	correct	correct	correct	correct	correct	*Strombus persicus*
Strombus luhuanus	JF693431	*Strombus luhuanus*	correct	correct	correct	correct	correct	correct	*Strombus persicus*
Strombus luhuanus	JF693432	*Strombus luhuanus*	correct	correct	correct	correct	correct	correct	*Strombus persicus*
Strombus marginatus robustus	JF693426	*Strombus marginatus robustus*	no id	correct	correct	no id	correct	correct	*Strombus vittatus*
Strombus marginatus robustus	JF693427	*Strombus marginatus robustus*	correct	correct	correct	correct	correct	correct	*Strombus vittatus*
Strombus marginatus robustus	JF693428	*Strombus marginatus robustus*	correct	correct	correct	correct	correct	correct	*Strombus vittatus*
Strombus marginatus robustus	JF693436	*Strombus marginatus robustus*	no id	correct	correct	no id	correct	correct	*Strombus vittatus*
Strombus marginatus robustus	JF693437	*Strombus marginatus robustus*	correct	correct	correct	correct	correct	correct	*Strombus vittatus*
Strombus urceus	JF693423	*Strombus urceus*	correct	correct	correct	correct	correct	correct	*Strombus urceus*
Strombus urceus	JF693424	*Strombus urceus*	correct	correct	correct	correct	correct	correct	*Strombus urceus*
Strombus urceus	JF693425	*Strombus urceus*	correct	correct	correct	correct	correct	correct	*Strombus urceus*
Strombus vittatus	JF693433	*Strombus vittatus*	no id	correct	correct	no id	correct	correct	*Strombus vittatus*
Strombus vittatus	JF693434	*Strombus vittatus*	correct	correct	correct	correct	correct	correct	*Strombus vittatus*
Strombus vittatus	JF693435	*Strombus vittatus*	correct	correct	correct	correct	correct	correct	*Strombus vittatus*
Tambja amakusana	EF142877	*Tambja limaciformis*	no id	no id	no id	no id	no id	no id	*Tambja limaciformis*
Tambja limaciformis	EF142878	*Tambja amakusana*	no id	no id	no id	no id	no id	no id	*Gastropoda sp.*
Tambja morosa	EF142867	*Tambja sagamiana*	no id	no id	no id	no id	no id	no id	*Tambja victoriae*
Tambja sagamiana	EF142870	*Tambja morosa*	no id	no id	no id	no id	no id	no id	*Tambja eliora*
Tapes dorsatus	HQ703224	*Tapes dorsatus*	correct	correct	correct	correct	correct	correct	*Marcia marmorata*
Tapes dorsatus	HQ703225	*Tapes dorsatus*	correct	correct	correct	correct	correct	correct	*Marcia marmorata*
Tapes dorsatus	HQ703226	*Tapes dorsatus*	correct	correct	correct	correct	correct	correct	*Marcia marmorata*
Tapes dorsatus	HQ703227	*Tapes dorsatus*	correct	correct	correct	correct	correct	correct	*Marcia marmorata*

Tapes dorsatus	HQ703228	*Tapes dorsatus*	correct	correct	correct	correct	correct	correct	*Marcia marmorata*
Tapes dorsatus	HQ703229	*Tapes dorsatus*	correct	correct	correct	correct	correct	correct	*Marcia marmorata*
Tapes dorsatus	HQ703230	*Tapes dorsatus*	correct	correct	correct	correct	correct	correct	*Marcia marmorata*
Tapes dorsatus	GQ855277	*Tapes dorsatus*	correct	correct	correct	correct	correct	correct	*Marcia marmorata*
Tapes dorsatus	JN898942	*Tapes dorsatus*	correct	correct	correct	correct	correct	correct	*Marcia marmorata*
Tapes dorsatus	HM124594	*Tapes dorsatus*	correct	correct	correct	correct	correct	correct	*Marcia marmorata*
Tapes literatus	HQ703217	*Tapes literata*	no id	correct	correct	no id	correct	correct	*Tapes belcheri*
Tapes literatus	HQ703218	*Tapes literata*	correct	correct	correct	correct	correct	correct	*Tapes belcheri*
Tapes literatus	HQ703219	*Tapes literata*	correct	correct	correct	correct	correct	correct	*Tapes belcheri*
Tapes literatus	HQ703220	*Tapes literata*	correct	correct	correct	correct	correct	correct	*Tapes belcheri*
Tapes literatus	HQ703221	*Tapes literata*	correct	correct	correct	correct	correct	correct	*Tapes belcheri*
Tapes literatus	HQ703222	*Tapes literata*	correct	correct	correct	correct	correct	correct	*Tapes belcheri*
Tapes literatus	HQ703223	*Tapes literata*	correct	correct	correct	correct	correct	correct	*Tapes belcheri*
Tapes literatus	GQ855278	*Tapes literata*	correct	correct	correct	correct	correct	correct	*Tapes belcheri*
Tapes literatus	GQ855279	*Tapes literata*	correct	correct	correct	correct	correct	correct	*Tapes belcheri*
Tapes literatus	GQ855280	*Tapes literata*	correct	correct	correct	correct	correct	correct	*Tapes belcheri*
Tapes literatus	JN898941	*Tapes literata*	correct	correct	correct	correct	correct	correct	*Tapes belcheri*
Tapes literatus	HM124603	*Tapes literata*	correct	correct	correct	correct	correct	correct	*Tapes belcheri*
Tectarius spinulosus	AJ488641	*Tectarius spinulosus*	correct	correct	correct	correct	correct	correct	*Tectarius spinulosus*
Tectarius spinulosus	AB611827	*Tectarius spinulosus*	correct	correct	correct	correct	correct	correct	*Tectarius spinulosus*
Tegillarca granosa	KU341910	*Tegillarca granosa*	correct	correct	correct	correct	correct	correct	
Tegillarca granosa	KU341911	*Tegillarca granosa*	correct	correct	correct	correct	correct	correct	
Tegillarca granosa	KU341912	*Tegillarca granosa*	correct	correct	correct	correct	correct	correct	
Tegillarca granosa	KU341913	*Tegillarca granosa*	correct	correct	correct	correct	correct	correct	
Tegillarca granosa	KU341914	*Tegillarca granosa*	correct	correct	correct	correct	correct	correct	
Tegillarca granosa	KU341915	*Tegillarca granosa*	correct	correct	correct	correct	correct	correct	
Tegillarca granosa	KU341916	*Tegillarca granosa*	correct	correct	correct	correct	correct	correct	
Tegillarca granosa	KU341917	*Tegillarca granosa*	correct	correct	correct	correct	correct	correct	
Tegillarca granosa	KU341918	*Tegillarca granosa*	correct	correct	correct	correct	correct	correct	
Tegillarca granosa	KU341919	*Tegillarca granosa*	correct	correct	correct	correct	correct	correct	
Tegillarca granosa	HQ258868	*Tegillarca granosa*	correct	correct	correct	correct	correct	correct	

Tegillarca nodifera	KU341906	*Tegillarca nodifera*	correct	correct	correct	correct	correct	correct	
Tegillarca nodifera	KU341907	*Tegillarca nodifera*	correct	correct	correct	correct	correct	correct	
Tegillarca nodifera	KU341908	*Tegillarca nodifera*	correct	correct	correct	correct	correct	correct	
Tegillarca nodifera	KU341909	*Tegillarca nodifera*	correct	correct	correct	correct	correct	correct	
Terebralia palustris	HE680652	*Cerithidea rhizophorarum*	no id	no id	no id	no id	no id	no id	*Terebralia palustris*
Terebralia sulcata	HE680669	*Terebralia sulcata*	correct	correct	correct	correct	correct	correct	*Terebralia sulcata*
Terebralia sulcata	HE680670	*Terebralia sulcata*	correct	correct	correct	correct	correct	correct	*Terebralia sulcata*
Terebralia sulcata	HE680663	*Terebralia sulcata*	no id	correct	correct	no id	correct	correct	*Terebralia sulcata*
Terebralia sulcata	HE680630	*Terebralia sulcata*	correct	correct	correct	correct	correct	correct	*Terebralia sulcata*
Terebralia sulcata	HE680667	*Terebralia sulcata*	correct	correct	correct	correct	correct	correct	*Terebralia sulcata*
Terebralia sulcata	HE680668	*Terebralia sulcata*	correct	correct	correct	correct	correct	correct	*Terebralia sulcata*
Terebralia sulcata	HE680671	*Terebralia sulcata*	correct	correct	correct	correct	correct	correct	*Terebralia sulcata*
Thais clavigera	GU188218	*Thais clavigera*	ambiguous	ambiguous	ambiguous	ambiguous	ambiguous	ambiguous	*Thais luteostoma*
Thais clavigera	GU188213	*Thais clavigera*	ambiguous	ambiguous	ambiguous	ambiguous	ambiguous	ambiguous	*Thais luteostoma*
Thais clavigera	GU188214	*Thais clavigera*	ambiguous	ambiguous	ambiguous	ambiguous	ambiguous	ambiguous	*Thais luteostoma*
Thais clavigera	GU188212	*Thais clavigera*	ambiguous	ambiguous	ambiguous	ambiguous	ambiguous	ambiguous	*Thais luteostoma*
Thais clavigera	GU188216	*Thais clavigera*	ambiguous	ambiguous	ambiguous	ambiguous	ambiguous	ambiguous	*Thais luteostoma*
Thais clavigera	GU188217	*Thais clavigera*	ambiguous	ambiguous	ambiguous	ambiguous	ambiguous	ambiguous	*Thais luteostoma*
Thais clavigera	HM180817	*Thais clavigera*	ambiguous	ambiguous	ambiguous	ambiguous	ambiguous	ambiguous	*Thais luteostoma*
Thais clavigera	HM180818	*Thais clavigera*	ambiguous	ambiguous	ambiguous	ambiguous	ambiguous	ambiguous	*Thais luteostoma*
Thais clavigera	HM180819	*Thais clavigera*	ambiguous	ambiguous	ambiguous	ambiguous	ambiguous	ambiguous	*Thais luteostoma*
Thais clavigera	HM180820	*Thais clavigera*	ambiguous	ambiguous	ambiguous	ambiguous	ambiguous	ambiguous	*Thais luteostoma*
Thais clavigera	HM180821	*Thais clavigera*	ambiguous	ambiguous	ambiguous	ambiguous	ambiguous	ambiguous	*Thais luteostoma*
Thais luteostoma	HM180831	*Thais clavigera*	incorrect	incorrect	incorrect	incorrect	incorrect	ambiguous	*Thais luteostoma*
Reishia bronni	HE584369	*Thais luteostoma*	ambiguous	ambiguous	ambiguous	ambiguous	ambiguous	ambiguous	*Reishia clavigera*
Reishia bronni	HE584368	*Thais luteostoma*	ambiguous	ambiguous	ambiguous	ambiguous	ambiguous	ambiguous	*Reishia clavigera*
Reishia bronni	FR695722	*Thais luteostoma*	ambiguous	ambiguous	ambiguous	ambiguous	ambiguous	ambiguous	*Reishia clavigera*
Thais luteostoma	GU188232	*Thais luteostoma*	ambiguous	ambiguous	ambiguous	ambiguous	ambiguous	ambiguous	*Reishia clavigera*
Thais luteostoma	GU188233	*Thais luteostoma*	ambiguous	ambiguous	ambiguous	ambiguous	ambiguous	ambiguous	*Reishia clavigera*
Thais luteostoma	GU188234	*Thais luteostoma*	correct	correct	correct	correct	ambiguous	ambiguous	*Reishia clavigera*
Thais luteostoma	GU188235	*Thais luteostoma*	ambiguous	ambiguous	ambiguous	ambiguous	ambiguous	ambiguous	*Reishia clavigera*

Thais luteostoma	GU188236	*Thais luteostoma*	correct	correct	correct	correct	ambiguous	ambiguous	*Reishia clavigera*
Thais luteostoma	GU188237	*Thais luteostoma*	ambiguous	ambiguous	ambiguous	ambiguous	ambiguous	ambiguous	*Reishia clavigera*
Thais luteostoma	HE584372	*Thais luteostoma*	no id	ambiguous	ambiguous	no id	ambiguous	ambiguous	*Reishia clavigera*
Thais luteostoma	HM180822	*Thais luteostoma*	no id	ambiguous	ambiguous	no id	ambiguous	ambiguous	*Reishia clavigera*
Thais luteostoma	HM180823	*Thais luteostoma*	ambiguous	ambiguous	ambiguous	ambiguous	ambiguous	ambiguous	*Reishia clavigera*
Thais luteostoma	HM180824	*Thais luteostoma*	ambiguous	ambiguous	ambiguous	ambiguous	ambiguous	ambiguous	*Reishia clavigera*
Thais luteostoma	HM180825	*Thais luteostoma*	ambiguous	ambiguous	ambiguous	ambiguous	ambiguous	ambiguous	*Reishia clavigera*
Thais luteostoma	HM180826	*Thais luteostoma*	ambiguous	ambiguous	ambiguous	ambiguous	ambiguous	ambiguous	*Reishia clavigera*
Thais luteostoma	HM180827	*Thais luteostoma*	ambiguous	ambiguous	ambiguous	ambiguous	ambiguous	ambiguous	*Reishia clavigera*
Thais luteostoma	HM180828	*Thais luteostoma*	ambiguous	ambiguous	ambiguous	ambiguous	ambiguous	ambiguous	*Reishia clavigera*
Thais luteostoma	HM180829	*Thais luteostoma*	ambiguous	ambiguous	ambiguous	ambiguous	ambiguous	ambiguous	*Reishia clavigera*
Thais luteostoma	HM180830	*Thais luteostoma*	ambiguous	ambiguous	ambiguous	ambiguous	ambiguous	ambiguous	*Reishia clavigera*
Thais gradata	GU188228	*Thais gradata*	correct	correct	correct	correct	correct	correct	*Thais javanica*
Thais gradata	GU188229	*Thais gradata*	correct	correct	correct	correct	correct	correct	*Thais javanica*
Thais javanica	GU188230	*Thais javanica*	correct	correct	correct	correct	correct	correct	*Thais sp.*
Thais javanica	GU188231	*Thais javanica*	correct	correct	correct	correct	correct	correct	*Thais sp.*
Thais mutabilis	GU188248	*Thais mutabilis*	correct	correct	correct	correct	correct	correct	*Drupella rugosa*
Thais mutabilis	GU188249	*Thais mutabilis*	correct	correct	correct	correct	correct	correct	*Drupella rugosa*
Thais mutabilis	GU188250	*Thais mutabilis*	correct	correct	correct	correct	correct	correct	*Drupella rugosa*
Thais mutabilis	GU188251	*Thais mutabilis*	correct	correct	correct	correct	correct	correct	*Drupella rugosa*
Thais mutabilis	GU188252	*Thais mutabilis*	correct	correct	correct	correct	correct	correct	*Drupella rugosa*
Thais mutabilis	GU188253	*Thais mutabilis*	correct	correct	correct	correct	correct	correct	*Drupella rugosa*
Thuridilla gracilis	AB758972	*Stiliger smaragdinus*	no id	no id	no id	no id	no id	no id	*Thuridilla bayeri*
Thuridilla splendens	AB758973	*Thuridilla gracilis*	no id	no id	no id	no id	no id	no id	*Thuridilla gracilis*
Tonna dolium	JF693441	*Tonna dolium*	correct	correct	correct	correct	correct	correct	*Tonna galea*
Tonna dolium	JF693442	*Tonna dolium*	correct	correct	correct	correct	correct	correct	*Tonna galea*
Tonna olearium	JF693438	*Tonna olearium*	no id	correct	correct	no id	correct	correct	*Tonna galea*
Tonna olearium	JF693439	*Tonna olearium*	no id	correct	correct	no id	correct	correct	*Tonna galea*
Tonna sulcosa	JF693440	*Duplicaria dussumieri*	no id	no id	no id	no id	no id	no id	*Crosseana crosseana*
Trisidos kiyonoi	HQ258842	*Trisidos kiyonoi*	no id	no id	correct	no id	no id	correct	
Trisidos kiyonoi	HQ258843	*Trisidos kiyonoi*	no id	correct	correct	no id	correct	correct	

Trisidos kiyonoi	KU341930	*Trisidos kiyonoi*	correct	correct	correct	correct	correct	correct	
Trisidos kiyonoi	KU341931	*Trisidos kiyonoi*	correct	correct	correct	correct	correct	correct	
Trisidos kiyonoi	HQ258845	*Trisidos kiyonoi*	correct	correct	correct	correct	correct	correct	
Trisidos kiyonoi	HQ258846	*Trisidos kiyonoi*	no id	correct	correct	no id	correct	correct	
Trochus histrio	AB505300	*Conotalopia mustelina*	no id	no id	no id	no id	no id	no id	*Trochus stellatus*
Trochus maculatus	EU530134	*Trochus maculatus*	correct	correct	correct	correct	correct	correct	*Trochus stellatus*
Trochus maculatus	AB365224	*Trochus maculatus*	no id	no id	correct	no id	no id	correct	*Trochus stellatus*
Trochus maculatus	AB505301	*Trochus maculatus*	correct	correct	correct	correct	correct	correct	*Trochus stellatus*
Trochus stellatus	EU530135	*Trochus maculatus*	no id	no id	no id	no id	no id	no id	*Trochus histrio*
Turritella terebra	JF693443	*Turritella terebra*	correct	correct	correct	correct	correct	correct	*Turritellidae sp.*
Turritella terebra	JF693444	*Turritella terebra*	correct	correct	correct	correct	correct	correct	*Turritellidae sp.*
Turritella terebra	JF693445	*Turritella terebra*	correct	correct	correct	correct	correct	correct	*Turritellidae sp.*
Turritella terebra	JF693446	*Turritella terebra*	no id	correct	correct	no id	correct	correct	*Turritellidae sp.*
Turritella terebra	JF693447	*Turritella terebra*	correct	correct	correct	correct	correct	correct	*Turritellidae sp.*
Turbo argyrostomus	AM403899	*Turbo cornutus*	no id	no id	incorrect	no id	no id	incorrect	*Turbo sp.*
Turbo chrysostomus	AM403903	*Turbo setosus*	no id	no id	no id	no id	no id	no id	*Turbinidae sp.*
Turbo cornutus	AM403882	*Turbo cornutus*	correct	correct	correct	correct	correct	correct	*Turbo marmoratus*
Turbo cornutus	AM403881	*Turbo cornutus*	correct	correct	correct	correct	correct	correct	*Turbo marmoratus*
Turbo cornutus	HM180932	*Turbo cornutus*	no id	correct	correct	no id	correct	ambiguous	*Turbo marmoratus*
Turbo cornutus	HM180933	*Turbo cornutus*	no id	correct	correct	no id	correct	correct	*Turbo marmoratus*
Turbo cornutus	HM180934	*Turbo cornutus*	no id	correct	correct	no id	correct	correct	*Turbo marmoratus*
Turbo marmoratus	AM403895	*Turbo marmoratus*	correct	correct	correct	correct	correct	correct	*Turbo cornutus*
Turbo marmoratus	AM403894	*Turbo marmoratus*	correct	correct	correct	correct	correct	correct	*Turbo cornutus*
Turbo petholatus	AM049383	*Turbo marmoratus*	no id	no id	no id	no id	no id	no id	*Turbo petholatus*
Turbo reevii	AM403878	*Echinolittorina trochoides E*	no id	no id	no id	no id	no id	no id	*Gastropoda sp.*
Turbo setosus	AM403909	*Turbo argyrostomus*	no id	no id	no id	no id	no id	no id	*Turbo setosus*
Turbo stenogyrus	AM403916	*Turbo stenogyrus*	correct	correct	correct	correct	correct	correct	*Turbo tuberculosus*
Turbo stenogyrus	AM403915	*Turbo stenogyrus*	correct	correct	correct	correct	correct	correct	*Turbo tuberculosus*
Turricula javana	HQ834091	*Duplicaria dussumieri*	no id	no id	no id	no id	no id	no id	*Turricula javana*
Umbonium giganteum	AB505314	*Umbonium giganteum*	correct	correct	correct	correct	correct	correct	*Umbonium costatum*
Umbonium giganteum	AB505313	*Umbonium giganteum*	correct	correct	correct	correct	correct	correct	*Umbonium costatum*

Umbonium moniliferum	AB505315	*Umbonium moniliferum*	no id	correct	correct	no id	correct	correct	*Umbonium costatum*
Umbonium moniliferum	AB365227	*Umbonium moniliferum*	no id	correct	correct	no id	correct	correct	*Umbonium costatum*
Uroteuthis sibogae	HQ529537	*Uroteuthis sibogae*	correct	correct	correct	correct	correct	correct	*Lolliguncula panamensis*
Uroteuthis sibogae	HQ529538	*Uroteuthis sibogae*	correct	correct	correct	correct	correct	correct	*Lolliguncula panamensis*
Uroteuthis sibogae	HQ529539	*Uroteuthis sibogae*	correct	correct	correct	correct	correct	correct	*Lolliguncula panamensis*
Uroteuthis duvaucelii	HQ529529	*Uroteuthis duvaucelii*	correct	correct	correct	correct	correct	correct	*Uroteuthis duvaucelii*
Uroteuthis duvaucelii	HQ529530	*Uroteuthis duvaucelii*	correct	correct	correct	correct	correct	correct	*Uroteuthis duvaucelii*
Uroteuthis duvaucelii	HQ529531	*Uroteuthis duvaucelii*	correct	correct	correct	correct	correct	correct	*Uroteuthis duvaucelii*
Uroteuthis duvaucelii	HQ529532	*Uroteuthis duvaucelii*	correct	correct	correct	correct	correct	correct	*Uroteuthis duvaucelii*
Uroteuthis duvaucelii	HQ529533	*Uroteuthis duvaucelii*	correct	correct	correct	correct	correct	correct	*Uroteuthis duvaucelii*
Uroteuthis duvaucelii	HQ529534	*Uroteuthis duvaucelii*	correct	correct	correct	correct	correct	correct	*Uroteuthis duvaucelii*
Uroteuthis duvaucelii	HQ529535	*Uroteuthis duvaucelii*	correct	correct	correct	correct	correct	correct	*Uroteuthis duvaucelii*
Uroteuthis duvaucelii	EU349465	*Uroteuthis duvaucelii*	correct	correct	correct	correct	correct	correct	*Uroteuthis duvaucelii*
Uroteuthis duvaucelii	EU349464	*Uroteuthis duvaucelii*	correct	correct	correct	correct	correct	correct	*Uroteuthis duvaucelii*
Uroteuthis duvaucelii	EU349463	*Uroteuthis duvaucelii*	correct	correct	correct	correct	correct	correct	*Uroteuthis duvaucelii*
Uroteuthis chinensis	HQ529528	*Uroteuthis chinensis*	correct	correct	correct	correct	correct	correct	*Uroteuthis etheridgei*
Uroteuthis chinensis	EU349446	*Uroteuthis chinensis*	correct	correct	correct	correct	correct	correct	*Uroteuthis etheridgei*
Uroteuthis chinensis	EU349445	*Uroteuthis chinensis*	correct	correct	correct	correct	correct	correct	*Uroteuthis etheridgei*
Uroteuthis chinensis	EU349444	*Uroteuthis chinensis*	correct	correct	correct	correct	correct	correct	*Uroteuthis etheridgei*
Uroteuthis chinensis	EU349443	*Uroteuthis chinensis*	correct	correct	correct	correct	correct	correct	*Uroteuthis etheridgei*
Uroteuthis chinensis	EU349438	*Uroteuthis chinensis*	correct	correct	correct	correct	correct	correct	*Uroteuthis etheridgei*
Uroteuthis chinensis	EU349437	*Uroteuthis chinensis*	correct	correct	correct	correct	correct	correct	*Uroteuthis etheridgei*
Uroteuthis edulis	EU349462	*Uroteuthis edulis*	correct	correct	correct	correct	correct	correct	*Uroteuthis edulis*
Uroteuthis edulis	EU349461	*Uroteuthis edulis*	correct	correct	correct	correct	correct	correct	*Uroteuthis edulis*
Uroteuthis edulis	EU349460	*Uroteuthis edulis*	correct	correct	correct	correct	correct	correct	*Uroteuthis edulis*
Uroteuthis edulis	EU349459	*Uroteuthis edulis*	correct	correct	correct	correct	correct	correct	*Uroteuthis edulis*
Uroteuthis edulis	EU349458	*Uroteuthis edulis*	correct	correct	correct	correct	correct	correct	*Uroteuthis edulis*
Uroteuthis edulis	AB675080	*Uroteuthis edulis*	correct	correct	correct	correct	correct	correct	*Uroteuthis edulis*
Uroteuthis edulis	AB675081	*Uroteuthis edulis*	correct	correct	correct	correct	correct	correct	*Uroteuthis edulis*
Uroteuthis edulis	KF032040	*Uroteuthis edulis*	correct	correct	correct	correct	correct	correct	*Uroteuthis edulis*
Uroteuthis edulis	KF032039	*Uroteuthis edulis*	correct	correct	correct	correct	correct	correct	*Uroteuthis edulis*

Uroteuthis edulis	KF032038	*Uroteuthis edulis*	correct	correct	correct	correct	correct	correct	*Uroteuthis edulis*
Uroteuthis edulis	KF032037	*Uroteuthis edulis*	correct	correct	correct	correct	correct	correct	*Uroteuthis edulis*
Uroteuthis edulis	EU349456	*Uroteuthis edulis*	correct	correct	correct	correct	correct	correct	*Uroteuthis edulis*
Uroteuthis edulis	EU349455	*Uroteuthis edulis*	correct	correct	correct	correct	correct	correct	*Uroteuthis edulis*
Uroteuthis edulis	EU349454	*Uroteuthis edulis*	correct	correct	correct	correct	correct	correct	*Uroteuthis edulis*
Uroteuthis edulis	EU349453	*Uroteuthis edulis*	correct	correct	correct	correct	correct	correct	*Uroteuthis edulis*
Uroteuthis edulis	EU349452	*Uroteuthis edulis*	correct	correct	correct	correct	correct	correct	*Uroteuthis edulis*
Uroteuthis edulis	EU349451	*Uroteuthis edulis*	correct	correct	correct	correct	correct	correct	*Uroteuthis edulis*
Uroteuthis edulis	EU349450	*Uroteuthis edulis*	correct	correct	correct	correct	correct	correct	*Uroteuthis edulis*
Uroteuthis edulis	EU349449	*Uroteuthis edulis*	correct	correct	correct	correct	correct	correct	*Uroteuthis edulis*
Uroteuthis edulis	EU349448	*Uroteuthis edulis*	correct	correct	correct	correct	correct	correct	*Uroteuthis edulis*
Uroteuthis edulis	EU349447	*Uroteuthis edulis*	correct	correct	correct	correct	correct	correct	*Uroteuthis edulis*
Varicinassa variciferus	JQ975551	*Varicinassa variciferus*	correct	correct	correct	correct	correct	correct	*Nassarius wolffi*
Varicinassa variciferus	JQ975546	*Varicinassa variciferus*	correct	correct	correct	correct	correct	correct	*Nassarius wolffi*
Varicinassa variciferus	JQ975547	*Varicinassa variciferus*	no id	correct	correct	no id	correct	correct	*Nassarius wolffi*
Varicinassa variciferus	JQ975548	*Varicinassa variciferus*	correct	correct	correct	correct	correct	correct	*Nassarius wolffi*
Varicinassa variciferus	JQ975549	*Varicinassa variciferus*	correct	correct	correct	correct	correct	correct	*Nassarius wolffi*
Varicinassa variciferus	JQ975550	*Varicinassa variciferus*	correct	correct	correct	correct	correct	correct	*Nassarius wolffi*
Vasticardium flavum	JN860016	*Vasticardium flavum*	correct	correct	correct	correct	correct	correct	*Vasticardium flavum*
Vasticardium flavum	JN860017	*Vasticardium flavum*	correct	correct	correct	correct	correct	correct	*Vasticardium flavum*
Vasticardium flavum	JN860018	*Vasticardium flavum*	correct	correct	correct	correct	correct	correct	*Vasticardium flavum*
Vasticardium flavum	JN860019	*Vasticardium flavum*	correct	correct	correct	correct	correct	correct	*Vasticardium flavum*
Vasticardium flavum	JN860020	*Vasticardium flavum*	no id	correct	correct	no id	correct	correct	*Vasticardium flavum*
Vasticardium flavum	JN860021	*Vasticardium flavum*	no id	correct	correct	no id	correct	correct	*Vasticardium flavum*
Vasum turbinellus	JN053025	*Vasum turbinellus*	correct	correct	correct	correct	correct	correct	*Vasum ceramicum*
Vasum turbinellus	HQ834084	*Vasum turbinellus*	correct	correct	correct	correct	correct	correct	*Vasum ceramicum*
Venerupis bruguieri	DQ184829	*Ruditapes variegata*	no id	no id	incorrect	no id	no id	incorrect	*Ruditapes variegatus*
Vepricardium coronatum	JN860013	*Vepricardium coronatum*	no id	correct	correct	no id	correct	correct	*Clinocardium nuttallii*
Vepricardium coronatum	JN860014	*Vepricardium coronatum*	correct	correct	correct	correct	correct	correct	*Clinocardium nuttallii*
Vepricardium coronatum	JN860015	*Vepricardium coronatum*	correct	correct	correct	correct	correct	correct	*Clinocardium nuttallii*
Volachlamys hirasei	GU120001	*Volachlamys hirasei*	no id	no id	no id	no id	no id	no id	

Volachlamys hirasei	GU120002	*Volachlamys hirasei*	correct	correct	correct	correct	correct	correct	
Volachlamys hirasei	GU120003	*Volachlamys hirasei*	correct	correct	correct	correct	correct	correct	
Volachlamys hirasei	GU120004	*Volachlamys hirasei*	correct	correct	correct	correct	correct	correct	
Volachlamys hirasei	GU120005	*Volachlamys hirasei*	correct	correct	correct	correct	correct	correct	
Volachlamys hirasei	GU120006	*Volachlamys hirasei*	correct	correct	correct	correct	correct	correct	
Volachlamys hirasei	GU120007	*Volachlamys hirasei*	correct	correct	correct	correct	correct	correct	
Volachlamys hirasei	GU120008	*Volachlamys hirasei*	no id	no id	correct	no id	no id	correct	
Volachlamys hirasei	GU120009	*Volachlamys hirasei*	no id	no id	correct	no id	no id	correct	
Volachlamys hirasei	GU120010	*Volachlamys hirasei*	correct	correct	correct	correct	correct	correct	
Volutharpa ampullacea perryi	JN053003	*Volutharpa ampullaceaperryi*	correct	correct	correct	correct	correct	correct	*Buccinum senshumaruae*
Volutharpa ampullacea perryi	JN053004	*Volutharpa ampullaceaperryi*	correct	correct	correct	correct	correct	correct	*Buccinum senshumaruae*
Volutharpa ampullacea perryi	HQ834060	*Volutharpa ampullaceaperryi*	correct	correct	correct	correct	correct	correct	*Buccinum senshumaruae*
Volutharpa ampullacea perryi	JN053002	*Volutharpa ampullaceaperryi*	correct	correct	correct	correct	correct	correct	*Buccinum senshumaruae*
Xenostrobus atrata	GQ480323	*Xenostrobus atrata*	correct	correct	correct	correct	correct	correct	*Xenostrobus atratus*
Xenostrobus atrata	GQ480324	*Xenostrobus atrata*	correct	correct	correct	correct	correct	correct	*Xenostrobus atratus*
Xenostrobus atrata	GQ480325	*Xenostrobus atrata*	correct	correct	correct	correct	correct	correct	*Xenostrobus atratus*
Xenostrobus atrata	GQ480326	*Xenostrobus atrata*	no id	correct	correct	no id	correct	correct	*Xenostrobus atratus*
Xenostrobus atrata	GQ480327	*Xenostrobus atrata*	correct	correct	correct	correct	correct	correct	*Xenostrobus atratus*

Supplementary Table 4. BINs with concordant taxonomy.

Process ID	Identification	Rank of Conflict	BIN	BIN Total Members
SSEO036-15	Bonartemis histrio	Genus	BOLD:AAO9165	4
SSEO037-15	Bonartemis histrio			
SSEO383-16	Adipicola crypta	Species	BOLD:AAD3750	3
SSEO387-16	Adipicola pacifica	Species	BOLD:ACH5470	4
SSEO386-16	Adipicola pacifica			
SSEO384-16	Adipicola pacifica			
SSEO388-16	Adipicola pacifica			
SSEO366-16	Adipicola pacifica			
SSEO385-16	Adipicola pacifica			
SSEO778-16	Alcyna ocellata	Species	BOLD:ACB8409	4
SSEO777-16	Alcyna ocellata			
QWEAS1346-15	Amphioctopus aegina	Species	BOLD:AAM5036	29
QWEAS192-15	Amphioctopus aegina			
QWEAS191-15	Amphioctopus aegina			
QWEAS187-15	Amphioctopus aegina			
QWEAS188-15	Amphioctopus aegina			
QWEAS190-15	Amphioctopus aegina			
QWEAS189-15	Amphioctopus aegina			
SSEO265-16	Amphioctopus fangsiao	Species	BOLD:AAE5989	22
QWEAS195-15	Amphioctopus fangsiao			
QWEAS193-15	Amphioctopus fangsiao			

QWEAS194-15	Amphioctopus fangsiao			
QWEAS197-15	Amphioctopus fangsiao			
SSEO264-16	Amphioctopus fangsiao	Species	BOLD:ABX6367	3
SSEO1314-16	Angaria formosa	Species	BOLD:AAJ2656	4
SSEO1315-16	Angaria formosa			
QWEAS938-15	Anomalocardia producta			
QWEAS939-15	Anomalocardia producta			
QWEAS936-15	Anomalocardia producta	Species	BOLD:AAO8010	17
QWEAS935-15	Anomalocardia producta			
QWEAS937-15	Anomalocardia producta			
QWEAS1488-15	Anomalodiscus squamosus			
QWEAS926-15	Anomalodiscus squamosus			
QWEAS924-15	Anomalodiscus squamosus	Species	BOLD:AAO8009	14
QWEAS925-15	Anomalodiscus squamosus			
QWEAS923-15	Anomalodiscus squamosus			
SSEO390-16	Anteaeolidiella takanosimensis	Species	BOLD:ACI1278	4
SSEO389-16	Anteaeolidiella takanosimensis			
QWEAS1489-15	Antigona lamellaris			
QWEAS1212-15	Antigona lamellaris			
QWEAS1215-15	Antigona lamellaris			
QWEAS1213-15	Antigona lamellaris			
QWEAS1211-15	Antigona lamellaris	Species	BOLD:AAJ2504	25
QWEAS1209-15	Antigona lamellaris			
QWEAS1214-15	Antigona lamellaris			
QWEAS1216-15	Antigona lamellaris			
QWEAS1210-15	Antigona lamellaris			

SSEO065-16	Aplysia kurodai	Species	BOLD:AAJ8409	6
SSEO066-16	Aplysia kurodai			
SSEO760-16	Atrina pectinata	Species	BOLD:AAD9828	90
SSEO757-16	Atrina pectinata			
SSEO759-16	Atrina pectinata			
SSEO758-16	Atrina pectinata	Species	BOLD:AAD9827	17
SSEO756-16	Atrina pectinata			
QWEAS318-15	Babylonia areolata	Species	BOLD:AAB7071	23
QWEAS319-15	Babylonia areolata			
QWEAS320-15	Babylonia areolata			
QWEAS317-15	Babylonia areolata			
SSEO751-16	Barbatia fusca	Species	BOLD:AAW3133	2
SSEO753-16	Barbatia lacerata	Species	BOLD:AAW3137	2
SSEO752-16	Barbatia lima	Species	BOLD:AAW3139	2
QWEAS1538-15	Barnea davidi	Species	BOLD:ACQ7230	2
QWEAS1540-15	Barnea dilatata	Species	BOLD:ACQ0719	4
QWEAS1539-15	Barnea dilatata			
SSEO421-16	Bathymodiolus aduloides	Species	BOLD:ACB6944	4
SSEO419-16	Bathymodiolus aduloides			
SSEO396-16	Bathymodiolus aduloides			
SSEO418-16	Bathymodiolus aduloides			
SSEO420-16	Bathymodiolus aduloides			
SSEO395-16	Bathymodiolus aduloides			
SSEO416-16	Bathymodiolus aduloides			
SSEO417-16	Bathymodiolus aduloides			
SSEO429-16	Bathymodiolus hirtus	Species	BOLD:AAW2782	2

SSEO430-16	Bathymodiolus hirtus			
SSEO426-16	Bathymodiolus japonicus			
SSEO427-16	Bathymodiolus japonicus	Species	BOLD:ACQ4898	2
SSEO428-16	Bathymodiolus japonicus			
SSEO412-16	Bathymodiolus platifrons			
SSEO432-16	Bathymodiolus platifrons	Species	BOLD:AAW2781	5
SSEO415-16	Bathymodiolus platifrons			
SSEO436-16	Bathymodiolus securiformis	Species	BOLD:ACQ4440	1
SSEO443-16	Bathymodiolus securiformis			
SSEO1153-16	Batillaria cumingii			
QWEAS475-15	Batillaria cumingii	Species	BOLD:ACY9200	11
QWEAS474-15	Batillaria cumingii			
QWEAS476-15	Batillaria cumingii			
QWEAS480-15	Batillaria zonalis	Species	BOLD:AAA7589	8
QWEAS481-15	Batillaria zonalis			
SSEO355-16	Benthomodiolus geikotsucola	Species	BOLD:ACH5802	4
SSEO350-16	Benthomodiolus geikotsucola			
SSEO321-16	Berryteuthis anonychus	Species	BOLD:AAM9198	3
SSEO322-16	Berryteuthis magister	Species	BOLD:AAD8239	7
QWEAS281-15	Boreotrophon xestra			
QWEAS284-15	Boreotrophon xestra	Species	BOLD:ACX4004	4
QWEAS282-15	Boreotrophon xestra			
QWEAS283-15	Boreotrophon xestra			
QWEAS056-15	Brachidontes mutabilis			
QWEAS059-15	Brachidontes mutabilis	Species	BOLD:ACQ6976	8
QWEAS057-15	Brachidontes mutabilis			

QWEAS058-15	Brachidontes mutabilis			
SSEO776-16	Broderipia iridescens	Species	BOLD:AAW6962	4
SSEO775-16	Broderipia iridescens			
SSEO007-15	Volutharpa ampullacea perryi	Species	BOLD:ACX3628	8
SSEO005-15	Volutharpa ampullacea perryi			
SSEO004-15	Volutharpa ampullacea perryi			
SSEO006-15	Volutharpa ampullacea perryi			
QWEAS237-15	Buccinum yokomaruae	Species	BOLD:ACX3481	4
QWEAS235-15	Buccinum yokomaruae			
QWEAS236-15	Buccinum yokomaruae			
QWEAS238-15	Buccinum yokomaruae			
QWEAS538-15	Bursa granularis	Species	BOLD:ACB7446	10
QWEAS540-15	Bursa granularis			
QWEAS542-15	Bursa granularis			
QWEAS541-15	Bursa granularis			
QWEAS539-15	Bursa granularis			
QWEAS560-15	Bursa rana	Species	BOLD:ACB8165	10
QWEAS564-15	Bursa rana			
QWEAS561-15	Bursa rana			
QWEAS562-15	Bursa rana			
QWEAS563-15	Bursa rana			
SSEO885-16	Calliostoma akoya	Species	BOLD:ACB7652	2
SSEO883-16	Calliostoma haliarchus	Species	BOLD:ACB7156	2
SSEO882-16	Calliostoma shinagawaensis	Species	BOLD:ACB7821	2
QWEAS913-15	Callista brevisiphonata	Species	BOLD:AAO9337	16
QWEAS915-15	Callista brevisiphonata			

QWEAS1472-15	Callista brevisiphonata			
QWEAS914-15	Callista brevisiphonata			
QWEAS912-15	Callista brevisiphonata			
QWEAS1471-15	Callista brevisiphonata			
QWEAS910-15	Callista chione	Species	BOLD:AAO9335	3
QWEAS911-15	Callista chione	Species	BOLD:AAO9336	3
SSEO239-16	Callistoctopus luteus	Species	BOLD:AAF5624	4
SSEO241-16	Callistoctopus luteus			
SSEO681-16	Calyptogena phaseoliformis	Species	BOLD:ACY9773	2
SSEO771-16	Cantharidus callichroa	Species	BOLD:AAF7715	4
SSEO769-16	Cantharidus callichroa			
SSEO770-16	Cantharidus callichroa	Species	BOLD:AAF7716	2
SSEO773-16	Cantharidus infuscatus	Species	BOLD:ACB8343	2
QWEAS243-15	Cantharus melanostomus	Species	BOLD:ACX3925	2
QWEAS244-15	Cantharus melanostomus			
QWEAS245-15	Cantharus cecillei	Species	BOLD:ACX3567	2
QWEAS246-15	Cantharus cecillei			
QWEAS1474-15	Cellana grata	Species	BOLD:ACQ5849	24
QWEAS1323-15	Cellana grata			
QWEAS1473-15	Cellana grata			
QWEAS1326-15	Cellana grata			
QWEAS1327-15	Cellana grata			
QWEAS1322-15	Cellana grata			
QWEAS1324-15	Cellana grata			
QWEAS1325-15	Cellana grata			
SSEO998-16	Cellana mazatlandica	Species	BOLD:AAD2518	26

SSEO994-16	Cellana mazatlandica			
SSEO979-16	Cellana mazatlandica			
SSEO997-16	Cellana mazatlandica			
SSEO996-16	Cellana mazatlandica			
SSEO960-16	Cellana mazatlandica			
SSEO964-16	Cellana mazatlandica			
SSEO995-16	Cellana mazatlandica			
SSEO962-16	Cellana mazatlandica			
SSEO993-16	Cellana mazatlandica			
SSEO963-16	Cellana mazatlandica			
SSEO961-16	Cellana mazatlandica			
SSEO958-16	Cellana mazatlandica			
SSEO926-16	Cellana nigrolineata	Species	BOLD:ACQ2208	68
SSEO927-16	Cellana nigrolineata			
SSEO953-16	Cellana nigrolineata			
SSEO934-16	Cellana nigrolineata			
SSEO957-16	Cellana nigrolineata			
SSEO946-16	Cellana nigrolineata			
SSEO936-16	Cellana nigrolineata			
SSEO933-16	Cellana nigrolineata			
SSEO916-16	Cellana nigrolineata			
SSEO929-16	Cellana nigrolineata			
SSEO940-16	Cellana nigrolineata			
SSEO945-16	Cellana nigrolineata			
SSEO941-16	Cellana nigrolineata			
SSEO955-16	Cellana nigrolineata			

SSEO923-16	Cellana nigrolineata			
SSEO948-16	Cellana nigrolineata			
SSEO924-16	Cellana nigrolineata			
SSEO939-16	Cellana nigrolineata			
SSEO935-16	Cellana nigrolineata			
SSEO931-16	Cellana nigrolineata			
SSEO922-16	Cellana nigrolineata			
SSEO930-16	Cellana nigrolineata			
SSEO938-16	Cellana nigrolineata			
SSEO932-16	Cellana nigrolineata			
SSEO952-16	Cellana nigrolineata			
SSEO942-16	Cellana nigrolineata			
SSEO925-16	Cellana nigrolineata			
SSEO956-16	Cellana nigrolineata			
SSEO920-16	Cellana nigrolineata			
SSEO928-16	Cellana nigrolineata			
SSEO947-16	Cellana nigrolineata			
SSEO921-16	Cellana nigrolineata			
SSEO954-16	Cellana nigrolineata			
SSEO937-16	Cellana nigrolineata			
SSEO970-16	Cellana nigrolineata	Species	BOLD:AAI7331	55
SSEO971-16	Cellana nigrolineata			
SSEO975-16	Cellana nigrolineata			
SSEO968-16	Cellana nigrolineata			
SSEO951-16	Cellana nigrolineata			
SSEO1372-16	Cellana nigrolineata			

SSEO972-16	Cellana nigrolineata			
SSEO913-16	Cellana nigrolineata			
SSEO917-16	Cellana nigrolineata			
SSEO943-16	Cellana nigrolineata			
SSEO974-16	Cellana nigrolineata			
SSEO978-16	Cellana nigrolineata			
SSEO944-16	Cellana nigrolineata			
SSEO976-16	Cellana nigrolineata			
SSEO919-16	Cellana nigrolineata			
SSEO914-16	Cellana nigrolineata			
SSEO1371-16	Cellana nigrolineata			
SSEO969-16	Cellana nigrolineata			
SSEO950-16	Cellana nigrolineata			
SSEO949-16	Cellana nigrolineata			
SSEO967-16	Cellana nigrolineata			
SSEO918-16	Cellana nigrolineata			
SSEO966-16	Cellana nigrolineata			
SSEO965-16	Cellana nigrolineata			
SSEO912-16	Cellana nigrolineata			
SSEO977-16	Cellana nigrolineata			
SSEO973-16	Cellana nigrolineata			
SSEO988-16	Cellana testudinaria	Species	BOLD:AAW6217	2
SSEO1401-16	Ceratostoma rorifluum	Species	BOLD:ACB8155	21
SSEO1378-16	Ceratostoma rorifluum			
QWEAS388-15	Ceratostoma rorifluum			
QWEAS389-15	Ceratostoma rorifluum			

QWEAS387-15	Ceratostoma rorifluum			
QWEAS384-15	Ceratostoma rorifluum			
QWEAS385-15	Ceratostoma rorifluum			
QWEAS386-15	Ceratostoma rorifluum			
SSEO1195-16	Cerithidea ornate	Species	BOLD:AAD8882	12
SSEO1191-16	Cerithidea ornate			
SSEO1193-16	Cerithidea ornate			
SSEO1192-16	Cerithidea ornate			
SSEO1194-16	Cerithidea ornate			
QWEAS490-15	Cerithium traillii	Species	BOLD:ACB7348	4
QWEAS491-15	Cerithium traillii			
QWEAS377-15	Chicoreus sp.	Species	BOLD:ACH7451	14
QWEAS375-15	Chicoreus sp.			
QWEAS376-15	Chicoreus sp.			
QWEAS379-15	Chicoreus sp.			
QWEAS374-15	Chicoreus sp.			
QWEAS378-15	Chicoreus sp.			
SSEO841-16	Chlorostoma lischkei	Species	BOLD:AAJ0065	5
SSEO1368-16	Chlorostoma lischkei			
SSEO1415-16	Chlorostoma turbinatum	Species	BOLD:ACB8508	2
QWEAS988-15	Circe scripta	Species	BOLD:AAO5746	18
QWEAS984-15	Circe scripta			
QWEAS986-15	Circe scripta			
QWEAS985-15	Circe scripta			
QWEAS987-15	Circe scripta			
QWEAS989-15	Circe scripta			

QWEAS983-15	Circe scripta	Species	BOLD:AAO5747	7
QWEAS982-15	Circe scripta			
SSEO768-16	Clanculus bronni	Species	BOLD:ACB7592	2
SSEO767-16	Clanculus margaritarius	Species	BOLD:ACB7049	2
SSEO810-16	Clanculus microdon	Species	BOLD:ACB7593	2
QWEAS631-15	Clinocardium californiense	Species	BOLD:ACQ0592	12
QWEAS632-15	Clinocardium californiense			
QWEAS636-15	Clinocardium californiense			
QWEAS635-15	Clinocardium californiense			
QWEAS633-15	Clinocardium californiense			
QWEAS634-15	Clinocardium californiense			
SSEO071-16	Coccopigya punctoradiata	Species	BOLD:AAJ0679	4
SSEO070-16	Coccopigya punctoradiata			
QWEAS1284-15	Coelomactra antiquata	Species	BOLD:ACH4893	16
QWEAS1285-15	Coelomactra antiquata			
QWEAS1286-15	Coelomactra antiquata	Species	BOLD:ACH4894	16
SSEO1317-16	Collonista amakusaensis	Species	BOLD:AAR9984	2
SSEO1318-16	Collonista costulosa	Species	BOLD:AAR9985	2
SSEO809-16	Conotalopia mustelina	Species	BOLD:ACB8111	2
SSEO808-16	Conotalopia ornata	Species	BOLD:ACB8718	2
QWEAS323-15	Conus betulinus	Species	BOLD:ACH7650	4
QWEAS324-15	Conus betulinus			
SSEO1063-16	Conus ebraeus	Species	BOLD:AAA4120	176
SSEO1072-16	Conus ebraeus			
SSEO1066-16	Conus ebraeus			
SSEO1103-16	Conus ebraeus			

SSEO1070-16	Conus ebraeus			
SSEO1068-16	Conus ebraeus			
SSEO1069-16	Conus ebraeus			
SSEO1065-16	Conus ebraeus			
SSEO1102-16	Conus ebraeus			
SSEO1067-16	Conus ebraeus			
SSEO1062-16	Conus ebraeus			
SSEO1104-16	Conus ebraeus			
SSEO1073-16	Conus ebraeus			
SSEO1081-16	Conus ebraeus			
SSEO1064-16	Conus ebraeus			
SSEO1188-16	Conus ebraeus			
SSEO1074-16	Conus ebraeus			
SSEO1071-16	Conus ebraeus			
SSEO1075-16	Conus judaeus	Species	BOLD:AAS0164	6
QWEAS325-15	Conus quercinus	Species	BOLD:AAO6779	7
QWEAS322-15	Conus textile	Species	BOLD:ACX4065	2
SSEO658-16	Corbicula sandai	Species	BOLD:ABZ7626	19
SSEO684-16	Corbicula sandai			
SSEO683-16	Corbicula sandai			
SSEO659-16	Corbicula sandai			
SSEO685-16	Corbicula sandai			
SSEO660-16	Corbicula sandai			
QWEAS091-15	Corbula erythrodon	Species	BOLD:ACH5926	3
SSEO690-16	Crassostrea angulata	Species	BOLD:ACH3409	48
SSEO687-16	Crassostrea angulata			

SSEO700-16	Crassostrea angulata			
SSEO699-16	Crassostrea angulata			
SSEO689-16	Crassostrea angulata			
SSEO713-16	Crassostrea angulata			
SSEO701-16	Crassostrea angulata			
SSEO715-16	Crassostrea angulata			
SSEO688-16	Crassostrea angulata			
SSEO714-16	Crassostrea angulata			
QWEAS1530-15	Crassostrea angulata			
QWEAS008-15	Crassostrea angulata			
QWEAS007-15	Crassostrea angulata			
SSEO392-16	Cryptobranchia kuragiensis	Species	BOLD:AAR4677	4
SSEO391-16	Cryptobranchia kuragiensis			
QWEAS610-15	Cultellus attenuatus	Species	BOLD:ACQ3574	8
QWEAS609-15	Cultellus attenuatus			
QWEAS607-15	Cultellus attenuatus			
QWEAS608-15	Cultellus attenuatus			
SSEO473-16	Cyclina sinensis	Species	BOLD:AAE2934	116
SSEO478-16	Cyclina sinensis			
SSEO472-16	Cyclina sinensis			
QWEAS997-15	Cyclina sinensis			
QWEAS1003-15	Cyclina sinensis			
QWEAS994-15	Cyclina sinensis			
QWEAS998-15	Cyclina sinensis			
QWEAS990-15	Cyclina sinensis			
QWEAS1001-15	Cyclina sinensis			

QWEAS993-15	Cyclina sinensis			
QWEAS1005-15	Cyclina sinensis			
QWEAS996-15	Cyclina sinensis			
QWEAS991-15	Cyclina sinensis			
QWEAS992-15	Cyclina sinensis			
QWEAS1004-15	Cyclina sinensis			
QWEAS1000-15	Cyclina sinensis			
QWEAS1002-15	Cyclina sinensis			
QWEAS1006-15	Cyclina sinensis			
QWEAS995-15	Cyclina sinensis			
QWEAS999-15	Cyclina sinensis			
QWEAS493-15	Cymatium cingulatum	Species	BOLD:ACB8396	4
QWEAS492-15	Cymatium cingulatum			
SSEO871-16	Dillwynella vitrea	Species	BOLD:AAI9907	5
SSEO872-16	Dillwynella vitrea			
SSEO807-16	Diloma piperinus	Species	BOLD:ACB8024	2
SSEO805-16	Diloma radula	Species	BOLD:AAX9594	3
SSEO806-16	Diloma suavis	Species	BOLD:ACB8023	2
QWEAS544-15	Distorsio reticularis	Species	BOLD:ACX3726	2
QWEAS543-15	Distorsio reticularis	Species	BOLD:ACB8328	2
QWEAS589-15	Donax dysoni	Species	BOLD:ACQ5978	13
QWEAS592-15	Donax dysoni			
QWEAS591-15	Donax dysoni			
QWEAS588-15	Donax dysoni			
QWEAS590-15	Donax dysoni			
QWEAS593-15	Donax dysoni			

QWEAS1014-15	Dosinia troscheli	Species	BOLD:AAO9164	7
QWEAS1015-15	Dosinia troscheli			
SSEO1099-16	Drupa grossularia	Species	BOLD:ACB7326	6
SSEO1095-16	Drupa rubusidaeus	Species	BOLD:ACB7008	9
SSEO1096-16	Drupa rubusidaeus			
SSEO1110-16	Drupella cornus	Species	BOLD:ACB7782	12
SSEO1094-16	Drupella eburnea	Species	BOLD:ACB8027	2
SSEO1109-16	Drupella rugosa	Species	BOLD:ACB8666	22
SSEO1108-16	Drupella rugosa			
QWEAS1518-15	Drupella rugosa			
QWEAS1512-15	Drupella rugosa			
QWEAS1519-15	Drupella rugosa			
QWEAS1515-15	Drupella rugosa			
QWEAS1513-15	Drupella rugosa			
QWEAS1516-15	Drupella rugosa			
QWEAS1514-15	Drupella rugosa			
QWEAS1511-15	Drupella rugosa			
QWEAS1517-15	Drupella rugosa			
QWEAS1337-15	Duplicaria sp.	Species	BOLD:ACX8893	2
SSEO1011-16	Echinolittorina cinerea	Species	BOLD:AAI8981	3
SSEO1010-16	Echinolittorina radiata	Species	BOLD:AAF6108	41
QWEAS559-15	Echinolittorina radiata			
QWEAS558-15	Echinolittorina radiata			
SSEO1059-16	Echinolittorina trochoides E	Species	BOLD:AAB5305	42
SSEO1037-16	Echinolittorina trochoides E			
SSEO1036-16	Echinolittorina trochoides E			

SSEO1032-16	Echinolittorina trochoides E			
SSEO1030-16	Echinolittorina trochoides E			
SSEO1060-16	Echinolittorina trochoides E			
SSEO1019-16	Echinolittorina trochoides E			
SSEO1022-16	Echinolittorina trochoides E			
SSEO1026-16	Echinolittorina trochoides E			
SSEO1020-16	Echinolittorina trochoides E			
SSEO1034-16	Echinolittorina trochoides E			
SSEO1033-16	Echinolittorina trochoides E			
SSEO1024-16	Echinolittorina trochoides E			
SSEO1035-16	Echinolittorina trochoides E			
SSEO1025-16	Echinolittorina trochoides E			
SSEO1029-16	Echinolittorina trochoides E			
SSEO1021-16	Echinolittorina trochoides E			
SSEO1031-16	Echinolittorina trochoides E			
SSEO1028-16	Echinolittorina trochoides E			
SSEO1023-16	Echinolittorina trochoides E			
SSEO1027-16	Echinolittorina trochoides E			
SSEO1050-16	Echinolittorina vidua			
SSEO1052-16	Echinolittorina vidua			
SSEO1056-16	Echinolittorina vidua			
SSEO1055-16	Echinolittorina vidua	Species	BOLD:ABY6936	31
SSEO1054-16	Echinolittorina vidua			
SSEO1057-16	Echinolittorina vidua			
SSEO1051-16	Echinolittorina vidua			
SSEO1058-16	Echinolittorina vidua			

SSEO177-16	Elysia pusilla	Species	BOLD:ACI0336	12
SSEO175-16	Elysia pusilla			
SSEO176-16	Elysia pusilla			
SSEO179-16	Elysia rufescens	Species	BOLD:ACH5921	7
SSEO178-16	Elysia rufescens			
SSEO173-16	Elysia trisinuata	Species	BOLD:ACI0714	2
SSEO447-16	Emarginula foveolata	Species	BOLD:AAI9219	4
SSEO448-16	Emarginula foveolata			
SSEO445-16	Emarginula variegata	Species	BOLD:AAI9222	4
SSEO446-16	Emarginula variegata			
SSEO804-16	Ethaliella floccata	Species	BOLD:ACY9621	4
SSEO803-16	Ethaliella floccata			
SSEO802-16	Ethaliella floccata	Species	BOLD:AAX7800	2
SSEO801-16	Ethminolia stearnsii	Species	BOLD:ACB8018	4
SSEO800-16	Ethminolia stearnsii			
QWEAS256-15	Euplica varians	Species	BOLD:ACX3948	3
QWEAS255-15	Euplica varians			
QWEAS254-15	Euplica varians			
SSEO799-16	Eurytrochus cognatus	Species	BOLD:AAY4216	4
SSEO798-16	Eurytrochus cognatus			
QWEAS547-15	Ficus ficus	Species	BOLD:ACB8561	6
QWEAS545-15	Ficus ficus			
QWEAS546-15	Ficus ficus			
QWEAS552-15	Ficus gracilis	Species	BOLD:ACB8357	6
QWEAS551-15	Ficus gracilis			
QWEAS553-15	Ficus gracilis			

QWEAS310-15	Fusinus longicaudus	Species	BOLD:ACX3667	2
QWEAS311-15	Fusinus longicaudus			
SSEO1321-16	Gabrielona pisinna	Species	BOLD:AAI7261	4
SSEO1322-16	Gabrielona pisinna			
QWEAS967-15	Gafrarium dispar	Species	BOLD:AAO5706	20
QWEAS965-15	Gafrarium dispar			
QWEAS966-15	Gafrarium dispar			
QWEAS969-15	Gafrarium dispar			
QWEAS962-15	Gafrarium dispar			
QWEAS968-15	Gafrarium dispar			
QWEAS961-15	Gafrarium dispar	Species	BOLD:AAO5707	3
QWEAS971-15	Gafrarium divaricatum	Species	BOLD:AAO5629	49
QWEAS978-15	Gafrarium divaricatum			
QWEAS979-15	Gafrarium divaricatum			
QWEAS976-15	Gafrarium divaricatum			
QWEAS972-15	Gafrarium divaricatum			
QWEAS970-15	Gafrarium divaricatum			
QWEAS974-15	Gafrarium divaricatum			
QWEAS981-15	Gafrarium divaricatum			
QWEAS975-15	Gafrarium divaricatum			
QWEAS980-15	Gafrarium divaricatum			
QWEAS977-15	Gafrarium divaricatum			
QWEAS973-15	Gafrarium divaricatum			
QWEAS954-15	Gafrarium pectinatum	Species	BOLD:AAO6328	11
QWEAS955-15	Gafrarium pectinatum			
QWEAS952-15	Gafrarium pectinatum			

QWEAS953-15	Gafrarium pectinatum			
QWEAS957-15	Gafrarium tumidum	Species	BOLD:AAO6039	16
QWEAS956-15	Gafrarium tumidum			
QWEAS959-15	Gafrarium tumidum			
QWEAS960-15	Gafrarium tumidum			
QWEAS958-15	Gafrarium tumidum			
SSEO678-16	Geloina expansa	Species	BOLD:AAY1138	3
SSEO433-16	Gigantidas horikoshii	Species	BOLD:ACQ2605	2
SSEO434-16	Gigantidas horikoshii			
SSEO394-16	Ginebis argenteonitens	Species	BOLD:ACY9249	3
SSEO400-16	Ginebis argenteonitens			
SSEO393-16	Ginebis argenteonitens			
QWEAS1217-15	Globivenus toreuma	Species	BOLD:AAO8887	3
SSEO754-16	Glycymeris reevei	Species	BOLD:AAY0660	2
SSEO755-16	Glycymeris rotunda	Species	BOLD:AAY0659	2
SSEO1138-16	Golikovia ennae	Species	BOLD:AAY0973	2
SSEO1137-16	Golikovia fukueae	Species	BOLD:AAY0975	2
SSEO323-16	Gonatopsis borealis	Species	BOLD:ACH8106	2
SSEO324-16	Gonatopsis octopedatus	Species	BOLD:AAL3847	4
SSEO306-16	Gonatus kamtschaticus	Species	BOLD:AAE3425	3
SSEO401-16	Granata lyrata	Species	BOLD:ACY9350	2
SSEO404-16	Granata lyrata			
SSEO1323-16	Guildfordia triumphans	Species	BOLD:AAI6429	4
SSEO1324-16	Guildfordia triumphans			
SSEO1325-16	Guildfordia yoka	Species	BOLD:AAF7349	6
SSEO1326-16	Guildfordia yoka			

SSEO1327-16	Guildfordia yoka			
SSEO112-16	Haminoea japonica	Species	BOLD:ACH4494	14
SSEO149-16	Haminoea japonica			
SSEO115-16	Haminoea japonica			
SSEO147-16	Haminoea japonica			
SSEO113-16	Haminoea japonica			
SSEO146-16	Haminoea japonica			
SSEO114-16	Haminoea japonica			
SSEO148-16	Haminoea japonica	Species	BOLD:ACH5215	10
SSEO118-16	Haminoea japonica	Species	BOLD:ACH4492	31
SSEO151-16	Haminoea japonica			
SSEO155-16	Haminoea japonica			
SSEO156-16	Haminoea japonica			
SSEO153-16	Haminoea japonica			
SSEO154-16	Haminoea japonica			
SSEO119-16	Haminoea japonica			
SSEO152-16	Haminoea japonica			
SSEO150-16	Haminoea japonica			
SSEO117-16	Haminoea japonica			
SSEO121-16	Haminoea japonica	Species	BOLD:ACI2127	128
SSEO142-16	Haminoea japonica			
SSEO132-16	Haminoea japonica			
SSEO137-16	Haminoea japonica			
SSEO125-16	Haminoea japonica			
SSEO129-16	Haminoea japonica			
SSEO131-16	Haminoea japonica			

SSEO130-16	Haminoea japonica			
SSEO144-16	Haminoea japonica			
SSEO140-16	Haminoea japonica			
SSEO139-16	Haminoea japonica			
SSEO141-16	Haminoea japonica			
SSEO134-16	Haminoea japonica			
SSEO143-16	Haminoea japonica			
SSEO136-16	Haminoea japonica			
SSEO120-16	Haminoea japonica			
SSEO127-16	Haminoea japonica			
SSEO133-16	Haminoea japonica			
SSEO145-16	Haminoea japonica			
SSEO135-16	Haminoea japonica			
SSEO124-16	Haminoea japonica			
SSEO128-16	Haminoea japonica			
SSEO123-16	Haminoea japonica			
SSEO138-16	Haminoea japonica			
SSEO116-16	Haminoea japonica			
SSEO122-16	Haminoea japonica			
SSEO126-16	Haminoea japonica			
SSEO351-16	Hapalochlaena fasciata	Species	BOLD:AAY1713	2
QWEAS217-15	Hapalochlaena maculosa	Species	BOLD:ABA8620	3
SSEO779-16	Hazuregyra watanabei	Species	BOLD:ACP9863	2
QWEAS267-15	Hemifusus colosseus	Species	BOLD:AAI6811	7
QWEAS266-15	Hemifusus colosseus			
SSEO1428-16	Hemifusus ternatanus	Species	BOLD:ABY6656	13

SSEO1429-16	Hemifusus ternatanus			
SSEO1430-16	Hemifusus ternatanus			
QWEAS269-15	Hemifusus ternatanus			
QWEAS270-15	Hemifusus ternatanus			
QWEAS271-15	Hemifusus ternatanus			
QWEAS268-15	Hemifusus ternatanus			
QWEAS272-15	Hemifusus ternatanus			
QWEAS276-15	Hemifusus tuba	Species	BOLD:ACX3419	8
QWEAS279-15	Hemifusus tuba			
QWEAS273-15	Hemifusus tuba			
QWEAS277-15	Hemifusus tuba			
QWEAS280-15	Hemifusus tuba			
QWEAS274-15	Hemifusus tuba			
QWEAS278-15	Hemifusus tuba			
QWEAS275-15	Hemifusus tuba			
SSEO211-16	Heterololigo bleekeri	Species	BOLD:AAB9046	47
SSEO213-16	Heterololigo bleekeri			
SSEO198-16	Heterololigo bleekeri			
SSEO215-16	Heterololigo bleekeri			
SSEO205-16	Heterololigo bleekeri			
SSEO201-16	Heterololigo bleekeri			
SSEO202-16	Heterololigo bleekeri			
SSEO203-16	Heterololigo bleekeri			
SSEO207-16	Heterololigo bleekeri			
SSEO200-16	Heterololigo bleekeri			
SSEO196-16	Heterololigo bleekeri			

SSEO228-16	Heterololigo bleekeri			
SSEO224-16	Heterololigo bleekeri			
SSEO226-16	Heterololigo bleekeri			
SSEO220-16	Heterololigo bleekeri			
SSEO216-16	Heterololigo bleekeri			
SSEO222-16	Heterololigo bleekeri			
SSEO212-16	Heterololigo bleekeri			
SSEO197-16	Heterololigo bleekeri			
SSEO422-16	Homalopoma granuliferum	Species	BOLD:AAI8216	4
SSEO411-16	Homalopoma granuliferum			
SSEO410-16	Homalopoma nocturnum	Species	BOLD:AAX1073	2
SSEO408-16	Homalopoma sangarense	Species	BOLD:AAX1078	2
SSEO289-16	Idiosepius paradoxus	Species	BOLD:ACH3045	4
SSEO288-16	Idiosepius paradoxus			
QWEAS879-15	Isognomon ephippium	Species	BOLD:ACX5630	5
QWEAS877-15	Isognomon ephippium			
QWEAS880-15	Isognomon ephippium			
QWEAS878-15	Isognomon ephippium			
QWEAS876-15	Isognomon ephippium			
QWEAS871-15	Isognomon legumen	Species	BOLD:ACX5794	5
QWEAS870-15	Isognomon legumen			
QWEAS872-15	Isognomon legumen			
QWEAS873-15	Isognomon legumen			
QWEAS874-15	Isognomon legumen			
SSEO1434-16	Kelletia lischkei	Species	BOLD:ACB7423	10
SSEO1431-16	Kelletia lischkei			

SSEO1432-16	Kelletia lischkei			
SSEO1435-16	Kelletia lischkei			
SSEO1433-16	Kelletia lischkei			
QWEAS505-15	Lambis lambis	Species	BOLD:AAX0552	7
QWEAS506-15	Lambis lambis			
QWEAS507-15	Lambis lambis			
SSEO835-16	Lepetodrilus nux	Species	BOLD:AAE4120	75
SSEO832-16	Lepetodrilus nux			
SSEO827-16	Lepetodrilus nux			
SSEO857-16	Lepetodrilus nux			
SSEO839-16	Lepetodrilus nux			
SSEO840-16	Lepetodrilus nux			
SSEO858-16	Lepetodrilus nux			
SSEO861-16	Lepetodrilus nux			
SSEO865-16	Lepetodrilus nux			
SSEO855-16	Lepetodrilus nux			
SSEO828-16	Lepetodrilus nux			
SSEO863-16	Lepetodrilus nux			
SSEO830-16	Lepetodrilus nux			
SSEO860-16	Lepetodrilus nux			
SSEO849-16	Lepetodrilus nux			
SSEO853-16	Lepetodrilus nux			
SSEO831-16	Lepetodrilus nux			
SSEO852-16	Lepetodrilus nux			
SSEO848-16	Lepetodrilus nux			
SSEO851-16	Lepetodrilus nux			

SSEO862-16	Lepetodrilus nux			
SSEO833-16	Lepetodrilus nux			
SSEO859-16	Lepetodrilus nux			
SSEO864-16	Lepetodrilus nux			
SSEO850-16	Lepetodrilus nux			
SSEO834-16	Lepetodrilus nux			
SSEO822-16	Lepetodrilus nux			
SSEO837-16	Lepetodrilus nux			
SSEO854-16	Lepetodrilus nux			
SSEO866-16	Lepetodrilus nux			
SSEO829-16	Lepetodrilus nux			
SSEO836-16	Lepetodrilus nux			
SSEO856-16	Lepetodrilus nux			
SSEO838-16	Lepetodrilus nux			
SSEO826-16	Lepetodrilus nux			
SSEO847-16	Limalepeta lima	Species	BOLD:ACB8179	4
SSEO846-16	Limalepeta lima			
SSEO361-16	Limnoperna fortunei	Species	BOLD:AAJ2118	79
SSEO348-16	Limnoperna fortunei			
SSEO365-16	Limnoperna fortunei			
SSEO371-16	Limnoperna fortunei			
SSEO363-16	Limnoperna fortunei			
SSEO347-16	Limnoperna fortunei			
SSEO356-16	Limnoperna fortunei			
SSEO373-16	Limnoperna fortunei			
SSEO372-16	Limnoperna fortunei			

SSEO359-16	Limnoperna fortunei			
SSEO374-16	Limnoperna fortunei			
SSEO369-16	Limnoperna fortunei			
SSEO368-16	Limnoperna fortunei			
SSEO346-16	Limnoperna fortunei			
SSEO358-16	Limnoperna fortunei			
SSEO362-16	Limnoperna fortunei			
SSEO364-16	Limnoperna fortunei			
SSEO370-16	Limnoperna fortunei			
SSEO357-16	Limnoperna fortunei			
SSEO360-16	Limnoperna fortunei			
SSEO794-16	Lirularia iridescens	Species	BOLD:AAJ2105	4
SSEO795-16	Lirularia iridescens			
SSEO797-16	Lirularia pygmaea	Species	BOLD:ACB7356	4
SSEO796-16	Lirularia pygmaea			
SSEO1015-16	Littoraria intermedia	Species	BOLD:ACH3623	8
QWEAS1506-15	Littoraria melanostoma	Species	BOLD:ACB7954	3
SSEO1017-16	Littoraria pallescens	Species	BOLD:AAO8511	8
SSEO1016-16	Littoraria pallescens			
SSEO1018-16	Littoraria scabra	Species	BOLD:AAK6714	30
SSEO980-16	Littoraria sinensis	Species	BOLD:ACH3757	6
SSEO986-16	Littorina brevicula	Species	BOLD:ACB8372	12
QWEAS556-15	Littorina brevicula			
QWEAS555-15	Littorina brevicula			
QWEAS554-15	Littorina brevicula			
SSEO985-16	Littorina horikawai	Species	BOLD:ACB8147	2

SSEO984-16	Littorina kasatka	Species	BOLD:ACB7143	2
SSEO983-16	Littorina mandshurica	Species	BOLD:ACB8237	2
SSEO982-16	Littorina squalida	Species	BOLD:ACB8238	2
QWEAS111-15	Loliolus beka	Species	BOLD:ABA8796	21
QWEAS112-15	Loliolus beka			
QWEAS109-15	Loliolus beka			
QWEAS110-15	Loliolus beka			
QWEAS113-15	Loliolus beka			
QWEAS106-15	Loliolus beka			
QWEAS107-15	Loliolus beka			
QWEAS115-15	Loliolus beka	Species	BOLD:ABA8797	24
QWEAS116-15	Loliolus beka			
QWEAS119-15	Loliolus beka			
QWEAS114-15	Loliolus beka			
QWEAS117-15	Loliolus beka			
QWEAS105-15	Loliolus beka			
QWEAS118-15	Loliolus beka			
QWEAS108-15	Loliolus beka			
QWEAS123-15	Loliolus uyii	Species	BOLD:ABA8798	15
QWEAS120-15	Loliolus uyii			
QWEAS124-15	Loliolus uyii			
QWEAS121-15	Loliolus uyii			
QWEAS122-15	Loliolus uyii			
QWEAS406-15	Lottia cassis	Species	BOLD:ACS6007	17
QWEAS411-15	Lottia cassis			
QWEAS408-15	Lottia cassis			

QWEAS409-15	Lottia cassis			
QWEAS410-15	Lottia cassis			
QWEAS407-15	Lottia cassis			
QWEAS397-15	Lottia dorsuosa			
QWEAS396-15	Lottia dorsuosa	Species	BOLD:ACS4419	6
QWEAS395-15	Lottia dorsuosa			
SSEO522-16	Lottia kogamogai	Species	BOLD:AAX6537	2
SSEO526-16	Lottia langfordi	Species	BOLD:AAX6536	2
SSEO527-16	Lottia lindbergi	Species	BOLD:AAX6534	2
SSEO528-16	Lottia luchuana			
QWEAS414-15	Lottia luchuana			
QWEAS418-15	Lottia luchuana			
QWEAS412-15	Lottia luchuana			
QWEAS415-15	Lottia luchuana	Species	BOLD:AAJ2353	16
QWEAS416-15	Lottia luchuana			
QWEAS417-15	Lottia luchuana			
QWEAS413-15	Lottia luchuana			
QWEAS419-15	Lottia luchuana	Species	BOLD:ACX3578	2
SSEO524-16	Lottia tenuisculpta	Species	BOLD:AAX6505	2
SSEO1281-16	Lunella ogasawarana			
SSEO1272-16	Lunella ogasawarana			
SSEO1277-16	Lunella ogasawarana			
SSEO1279-16	Lunella ogasawarana	Species	BOLD:AAE3889	20
SSEO1280-16	Lunella ogasawarana			
SSEO1275-16	Lunella ogasawarana			
SSEO1276-16	Lunella ogasawarana			

SSEO1282-16	Lunella ogasawarana			
SSEO1273-16	Lunella ogasawarana			
SSEO1278-16	Lunella ogasawarana			
QWEAS1292-15	Lutraria arcuata	Species	BOLD:ACH8903	6
QWEAS1291-15	Lutraria arcuata			
QWEAS1293-15	Lutraria arcuata			
QWEAS1290-15	Lutraria australis	Species	BOLD:ACH7908	4
QWEAS581-15	Macoma candida	Species	BOLD:ACQ7092	4
QWEAS578-15	Macoma tokyoensis	Species	BOLD:ACQ2628	5
QWEAS579-15	Macoma tokyoensis			
SSEO438-16	Macroschisma dilatata	Species	BOLD:AAJ1496	2
SSEO441-16	Macroschisma dilatata	Species	BOLD:AAJ1495	2
QWEAS1433-15	Mactra chinensis	Species	BOLD:AAX3441	84
QWEAS1437-15	Mactra chinensis			
QWEAS1432-15	Mactra chinensis			
QWEAS1436-15	Mactra chinensis			
QWEAS1430-15	Mactra chinensis			
QWEAS1434-15	Mactra chinensis			
QWEAS1435-15	Mactra chinensis			
QWEAS1431-15	Mactra chinensis			
QWEAS1277-15	Mactra chinensis			
QWEAS1275-15	Mactra chinensis			
QWEAS1278-15	Mactra chinensis			
QWEAS1279-15	Mactra chinensis			
QWEAS1274-15	Mactra chinensis			
QWEAS1276-15	Mactra chinensis			

QWEAS1289-15	Mactra cumingii			
QWEAS1287-15	Mactra cumingii	Species	BOLD:ACH8792	6
QWEAS1288-15	Mactra cumingii			
QWEAS1267-15	Mactra maculata	Species	BOLD:ACH8794	4
QWEAS1266-15	Mactra maculata			
QWEAS1273-15	Mactra sp.			
QWEAS1271-15	Mactra sp.			
QWEAS1268-15	Mactra sp.	Species	BOLD:ACH8793	12
QWEAS1269-15	Mactra sp.			
QWEAS1270-15	Mactra sp.			
QWEAS1272-15	Mactra sp.			
SSEO1111-16	Mancinella echinata	Species	BOLD:ACB8741	6
QWEAS1157-15	Marcia japonica			
QWEAS1241-15	Marcia japonica			
QWEAS1240-15	Marcia japonica			
QWEAS1242-15	Marcia japonica			
QWEAS1238-15	Marcia japonica	Species	BOLD:AAK7715	27
QWEAS1160-15	Marcia japonica			
QWEAS1159-15	Marcia japonica			
QWEAS1158-15	Marcia japonica			
QWEAS1239-15	Marcia japonica			
QWEAS1161-15	Marcia japonica			
QWEAS501-15	Margistrombus robustus			
QWEAS497-15	Margistrombus robustus	Species	BOLD:ACB7311	10
QWEAS500-15	Margistrombus robustus			
QWEAS498-15	Margistrombus robustus			

QWEAS499-15	Margistrombus robustus			
QWEAS1542-15	Martesia striata	Species	BOLD:ACH5863	8
QWEAS1541-15	Martesia striata			
QWEAS520-15	Mauritia arabica	Species	BOLD:AAC4112	12
QWEAS521-15	Mauritia arabica			
QWEAS523-15	Mauritia arabica			
QWEAS522-15	Mauritia arabica			
SSEO106-16	Melanochlamys ezoensis	Species	BOLD:ACQ8416	15
SSEO110-16	Melanochlamys ezoensis			
SSEO107-16	Melanochlamys ezoensis			
SSEO105-16	Melanochlamys ezoensis			
SSEO109-16	Melanochlamys ezoensis			
SSEO108-16	Melanochlamys ezoensis			
SSEO111-16	Melanochlamys ezoensis			
SSEO086-16	Melanochlamys fukudai	Species	BOLD:ACQ1294	50
SSEO100-16	Melanochlamys fukudai			
SSEO097-16	Melanochlamys fukudai			
SSEO083-16	Melanochlamys fukudai			
SSEO099-16	Melanochlamys fukudai			
SSEO095-16	Melanochlamys fukudai			
SSEO085-16	Melanochlamys fukudai			
SSEO084-16	Melanochlamys fukudai			
SSEO088-16	Melanochlamys fukudai			
SSEO089-16	Melanochlamys fukudai			
SSEO090-16	Melanochlamys fukudai			
SSEO096-16	Melanochlamys fukudai			

SSEO080-16	Melanochlamys fukudai			
SSEO102-16	Melanochlamys fukudai			
SSEO082-16	Melanochlamys fukudai			
SSEO104-16	Melanochlamys fukudai			
SSEO087-16	Melanochlamys fukudai			
SSEO091-16	Melanochlamys fukudai			
SSEO081-16	Melanochlamys fukudai			
SSEO098-16	Melanochlamys fukudai			
SSEO094-16	Melanochlamys fukudai			
SSEO101-16	Melanochlamys fukudai			
SSEO103-16	Melanochlamys fukudai			
SSEO092-16	Melanochlamys fukudai			
SSEO093-16	Melanochlamys fukudai			
SSEO079-16	Melanochlamys kohi	Species	BOLD:ACQ4317	8
SSEO077-16	Melanochlamys kohi			
SSEO078-16	Melanochlamys kohi			

QWEAS315-15	Melo melo	Species	BOLD:ACH8424	8
QWEAS314-15	Melo melo			
QWEAS313-15	Melo melo			
QWEAS312-15	Melo melo			
SSEO487-16	Mercenaria mercenaria	Species	BOLD:AAA3989	216
SSEO500-16	Mercenaria mercenaria			
SSEO495-16	Mercenaria mercenaria			
SSEO525-16	Mercenaria mercenaria			

SSEO501-16	Mercenaria mercenaria			
SSEO516-16	Mercenaria mercenaria			
SSEO465-16	Mercenaria mercenaria			
SSEO489-16	Mercenaria mercenaria			
SSEO514-16	Mercenaria mercenaria			
SSEO467-16	Mercenaria mercenaria			
SSEO488-16	Mercenaria mercenaria			
SSEO484-16	Mercenaria mercenaria			
SSEO513-16	Mercenaria mercenaria			
SSEO470-16	Mercenaria mercenaria			
SSEO572-16	Mercenaria mercenaria			
SSEO486-16	Mercenaria mercenaria			
SSEO520-16	Mercenaria mercenaria			
SSEO509-16	Mercenaria mercenaria			
SSEO519-16	Mercenaria mercenaria			
SSEO512-16	Mercenaria mercenaria			
SSEO471-16	Mercenaria mercenaria			
SSEO506-16	Mercenaria mercenaria			
SSEO490-16	Mercenaria mercenaria			
SSEO523-16	Mercenaria mercenaria			
SSEO469-16	Mercenaria mercenaria			
SSEO505-16	Mercenaria mercenaria			
SSEO515-16	Mercenaria mercenaria			
SSEO511-16	Mercenaria mercenaria			
SSEO521-16	Mercenaria mercenaria			
SSEO493-16	Mercenaria mercenaria			

SSEO518-16	Mercenaria mercenaria			
SSEO492-16	Mercenaria mercenaria			
SSEO507-16	Mercenaria mercenaria			
SSEO503-16	Mercenaria mercenaria			
SSEO517-16	Mercenaria mercenaria			
SSEO491-16	Mercenaria mercenaria			
QWEAS1452-15	Mercenaria mercenaria			
QWEAS1453-15	Mercenaria mercenaria			
QWEAS1450-15	Mercenaria mercenaria			
QWEAS1451-15	Mercenaria mercenaria			
QWEAS947-15	Mercenaria mercenaria			
QWEAS946-15	Mercenaria mercenaria			
QWEAS950-15	Mercenaria mercenaria			
QWEAS948-15	Mercenaria mercenaria			
QWEAS951-15	Mercenaria mercenaria			
QWEAS949-15	Mercenaria mercenaria			
QWEAS1494-15	Meretrix lamarckii	Species	BOLD:AAE9722	25
QWEAS1493-15	Meretrix lamarckii			
QWEAS1065-15	Meretrix lamarckii			
QWEAS1063-15	Meretrix lamarckii			
QWEAS1064-15	Meretrix lamarckii			
QWEAS1066-15	Meretrix lamarckii			
SSEO564-16	Meretrix lusoria	Species	BOLD:AAD4072	27
SSEO1365-16	Meretrix lusoria			
SSEO569-16	Meretrix lusoria			
SSEO568-16	Meretrix lusoria			

SSEO571-16	Meretrix lusoria			
SSEO873-16	Mikadotrochus beyrichii	Species	BOLD:AAG5178	4
SSEO887-16	Mikadotrochus beyrichii			
SSEO1443-16	Mitrella bicincta	Species	BOLD:ACB6968	9
SSEO1446-16	Mitrella bicincta			
QWEAS259-15	Mitrella bicincta			
QWEAS263-15	Mitrella bicincta			
QWEAS260-15	Mitrella bicincta			
QWEAS262-15	Mitrella bicincta			
QWEAS261-15	Mitrella bicincta			
QWEAS067-15	Modiolus auriculatus	Species	BOLD:ACQ2675	2
QWEAS068-15	Modiolus elongatus	Species	BOLD:ACQ6657	2
QWEAS1373-15	Modiolus kurilensis	Species	BOLD:ACV6375	12
QWEAS1374-15	Modiolus kurilensis			
QWEAS1372-15	Modiolus kurilensis			
QWEAS1370-15	Modiolus kurilensis			
QWEAS1369-15	Modiolus kurilensis			
QWEAS1371-15	Modiolus kurilensis			
QWEAS069-15	Modiolus metcalfei	Species	BOLD:ACQ2878	8
QWEAS072-15	Modiolus metcalfei			
QWEAS071-15	Modiolus metcalfei			
QWEAS070-15	Modiolus metcalfei			
SSEO792-16	Monilea smithi	Species	BOLD:ACB8008	4
SSEO793-16	Monilea smithi			
SSEO1453-16	Monodonta australis	Species	BOLD:ACB7447	7
SSEO1450-16	Monodonta australis			

SSEO1452-16	Monodonta australis	Species	BOLD:ACB7257	10
SSEO1454-16	Monodonta australis			
SSEO1455-16	Monodonta australis			
SSEO1456-16	Monodonta australis			
SSEO1451-16	Monodonta australis			
SSEO824-16	Monodonta canalifera	Species	BOLD:AAG7382	4
SSEO823-16	Monodonta canalifera			
SSEO1127-16	Morula funiculata	Species	BOLD:ACH9039	6
SSEO1129-16	Morula funiculata			
SSEO1126-16	Morula funiculata			
SSEO1135-16	Morula japonica	Species	BOLD:ACH9037	2
SSEO1136-16	Morula rumphiusi	Species	BOLD:ACH5962	2
QWEAS285-15	Morula sp.	Species	BOLD:ACX3606	3
QWEAS286-15	Morula sp.			
SSEO1131-16	Morula striata	Species	BOLD:ACY9406	3
SSEO1132-16	Morula striata	Species	BOLD:ACH4892	3
SSEO869-16	Munditiella ammonoceras	Species	BOLD:AAG1056	4
SSEO870-16	Munditiella ammonoceras			
QWEAS372-15	Murex trapa	Species	BOLD:ACI2167	12
QWEAS369-15	Murex trapa			
QWEAS370-15	Murex trapa			
QWEAS373-15	Murex trapa			
QWEAS371-15	Murex trapa			
SSEO453-16	Musculista senhousia	Species	BOLD:AAB4685	15
QWEAS093-15	Mya arenaria	Species	BOLD:ABX1812	18
QWEAS092-15	Mya arenaria			

QWEAS1544-15	Nassarius conoidalis	Species	BOLD:ACI2486	6
QWEAS1545-15	Nassarius conoidalis			
QWEAS1543-15	Nassarius conoidalis			
QWEAS307-15	Nassarius festivus	Species	BOLD:ACH7262	28
QWEAS305-15	Nassarius festivus			
QWEAS303-15	Nassarius festivus			
QWEAS306-15	Nassarius festivus			
QWEAS308-15	Nassarius festivus			
QWEAS309-15	Nassarius festivus			
QWEAS1561-15	Nassarius livescens	Species	BOLD:ACH4907	2
QWEAS1564-15	Nassarius livescens	Species	BOLD:ACH4906	6
QWEAS1562-15	Nassarius livescens			
QWEAS1563-15	Nassarius livescens			
QWEAS1550-15	Nassarius pullus	Species	BOLD:ACH4905	14
QWEAS1554-15	Nassarius pullus			
QWEAS1549-15	Nassarius pullus			
QWEAS1551-15	Nassarius pullus			
QWEAS1553-15	Nassarius pullus			
QWEAS1548-15	Nassarius pullus			
QWEAS1552-15	Nassarius pullus			
QWEAS1546-15	Nassarius semiplicatus	Species	BOLD:ACH4904	4
QWEAS1547-15	Nassarius semiplicatus			
QWEAS535-15	Natica lineata	Species	BOLD:ACB7071	8
QWEAS534-15	Natica lineata			
QWEAS537-15	Natica lineata			
QWEAS536-15	Natica lineata			

SSEO1154-16	Neptunea constricta	Species	BOLD:AAM1847	4
SSEO1467-16	Neptunea cumingi	Species	BOLD:ACF4244	4
SSEO1466-16	Neptunea cumingi			
SSEO1161-16	Neptunea mikawaensis	Species	BOLD:AAX6641	2
SSEO1163-16	Neptunea polycostata	Species	BOLD:AAH6465	6
SSEO1162-16	Neptunea polycostata			
SSEO510-16	Nerita albicilla	Species	BOLD:AAA1276	234
SSEO479-16	Nerita helicinoides	Species	BOLD:AAH0947	2
SSEO480-16	Nerita helicinoides	Species	BOLD:AAH0946	2
SSEO481-16	Nerita japonica	Species	BOLD:AAE5970	9
SSEO482-16	Nerita japonica			
SSEO483-16	Nerita ocellata	Species	BOLD:AAH2200	4
SSEO485-16	Nerita ocellata			
SSEO494-16	Nerita planospira	Species	BOLD:AAH2844	18
SSEO496-16	Nerita plicata	Species	BOLD:AAA1249	456
SSEO497-16	Nerita tristis	Species	BOLD:AAH3283	4
SSEO498-16	Nerita tristis			
QWEAS1586-15	Nerita undata	Species	BOLD:ABY4809	6
QWEAS1587-15	Nerita undata			
SSEO499-16	Nerita undata	Species	BOLD:ABY9761	4
SSEO502-16	Nerita undata			
SSEO508-16	Nerita undulata	Species	BOLD:ABZ5000	7
SSEO504-16	Nerita undulata			
QWEAS1589-15	Nerita yoldii	Species	BOLD:AAH2930	4
QWEAS1588-15	Nerita yoldii			
SSEO069-16	Neritilia littoralis	Species	BOLD:AAX6363	2

SSEO068-16	Neritilia mimotoi	Species	BOLD:AAX6364	2
SSEO067-16	Neritilia rubida	Species	BOLD:AAX6365	2
SSEO458-16	Neritina asperulata	Species	BOLD:ACH4773	9
SSEO457-16	Neritina asperulata			
SSEO460-16	Neritina asperulata			
SSEO463-16	Neritina iris	Species	BOLD:ACI2200	7
SSEO462-16	Neritina iris			
SSEO477-16	Neritina pulligera	Species	BOLD:ACH4875	6
SSEO476-16	Neritina pulligera			
SSEO531-16	Nipponacmea gloriosa	Species	BOLD:AAX1748	2
SSEO532-16	Nipponacmea habei	Species	BOLD:AAX6435	2
SSEO533-16	Nipponacmea nigrans	Species	BOLD:AAX6433	2
SSEO555-16	Nipponacmea teramachii	Species	BOLD:AAX6428	2
SSEO354-16	Octopus conispadiceus	Species	BOLD:AAI3633	2
SSEO342-16	Octopus incella	Species	BOLD:AAW9984	2
SSEO343-16	Octopus laqueus	Species	BOLD:AAW9979	2
QWEAS224-15	Octopus nanhaiensis	Species	BOLD:ABA8784	3
SSEO353-16	Octopus oliveri	Species	BOLD:AAW9976	5
SSEO344-16	Octopus parvus	Species	BOLD:AAI3609	2
SSEO345-16	Octopus wolfi	Species	BOLD:AAW9986	2
SSEO1478-16	Omphalius pfeifferi carpenteri	Species	BOLD:ACB7312	7
SSEO1479-16	Omphalius pfeifferi carpenteri			
SSEO546-16	Paphia euglypta	Species	BOLD:AAX2585	2
QWEAS1218-15	Paphia papilionacea	Species	BOLD:AAO8674	34
QWEAS1223-15	Paphia papilionacea			
QWEAS1117-15	Paphia papilionacea			

QWEAS1118-15	Paphia papilionacea			
QWEAS1115-15	Paphia papilionacea			
QWEAS1222-15	Paphia papilionacea			
QWEAS1221-15	Paphia papilionacea			
QWEAS1113-15	Paphia papilionacea			
QWEAS1456-15	Paphia papilionacea			
QWEAS1457-15	Paphia papilionacea			
QWEAS1220-15	Paphia papilionacea			
QWEAS1116-15	Paphia papilionacea			
QWEAS1114-15	Paphia papilionacea			
QWEAS1219-15	Paphia papilionacea			
QWEAS1138-15	Paphia semirugata			
QWEAS1497-15	Paphia semirugata	Species	BOLD:AAO8677	10
QWEAS1140-15	Paphia semirugata			
QWEAS1139-15	Paphia semirugata	Species	BOLD:AAO8678	3
QWEAS1389-15	Paphia sinuosa	Species	BOLD:ABA7706	3
QWEAS1106-15	Paphia sinuosa	Species	BOLD:AAO8671	3
QWEAS1227-15	Paphia undulata			
QWEAS1225-15	Paphia undulata			
QWEAS1122-15	Paphia undulata			
QWEAS1123-15	Paphia undulata			
QWEAS1121-15	Paphia undulata	Species	BOLD:AAO8675	27
QWEAS1467-15	Paphia undulata			
QWEAS1119-15	Paphia undulata			
QWEAS1224-15	Paphia undulata			
QWEAS1226-15	Paphia undulata			

QWEAS1120-15	Paphia undulata			
SSEO545-16	Paphia vernicosa	Species	BOLD:AAX2573	2
QWEAS1575-15	Paraoncidium reevesii	Species	BOLD:AAM2279	31
QWEAS1572-15	Paraoncidium reevesii			
QWEAS1570-15	Paraoncidium reevesii			
QWEAS1574-15	Paraoncidium reevesii			
QWEAS1576-15	Paraoncidium reevesii			
QWEAS1573-15	Paraoncidium reevesii			
QWEAS1571-15	Paraoncidium reevesii			
SSEO621-16	Patelloida conulus	Species	BOLD:AAB2536	54
SSEO1289-16	Patelloida conulus			
SSEO1291-16	Patelloida conulus			
SSEO1295-16	Patelloida conulus			
SSEO1287-16	Patelloida conulus			
SSEO1299-16	Patelloida conulus			
SSEO624-16	Patelloida conulus			
SSEO620-16	Patelloida conulus			
SSEO1297-16	Patelloida conulus			
SSEO1293-16	Patelloida conulus			
SSEO622-16	Patelloida conulus			
SSEO1296-16	Patelloida conulus			
SSEO1288-16	Patelloida conulus			
SSEO1292-16	Patelloida conulus			
SSEO1301-16	Patelloida conulus			
SSEO1298-16	Patelloida conulus			
SSEO1294-16	Patelloida conulus			

SSEO1312-16	Patelloida conulus			
SSEO615-16	Patelloida conulus			
SSEO616-16	Patelloida conulus			
SSEO1300-16	Patelloida conulus			
SSEO618-16	Patelloida conulus			
SSEO619-16	Patelloida conulus			
SSEO614-16	Patelloida conulus			
SSEO617-16	Patelloida conulus			
SSEO591-16	Patelloida heroldi	Species	BOLD:AAB4086	60
SSEO608-16	Patelloida heroldi			
SSEO605-16	Patelloida heroldi			
SSEO583-16	Patelloida heroldi			
SSEO613-16	Patelloida heroldi			
SSEO596-16	Patelloida heroldi			
SSEO593-16	Patelloida heroldi			
SSEO607-16	Patelloida heroldi			
SSEO602-16	Patelloida heroldi			
SSEO580-16	Patelloida heroldi			
SSEO611-16	Patelloida heroldi			
SSEO600-16	Patelloida heroldi			
SSEO609-16	Patelloida heroldi			
SSEO582-16	Patelloida heroldi			
SSEO586-16	Patelloida heroldi			
SSEO612-16	Patelloida heroldi			
SSEO589-16	Patelloida heroldi			
SSEO585-16	Patelloida heroldi			

SSEO584-16	Patelloida heroldi			
SSEO587-16	Patelloida heroldi			
SSEO610-16	Patelloida heroldi			
SSEO581-16	Patelloida heroldi			
SSEO559-16	Patelloida lentiginosa	Species	BOLD:AAX1753	2
SSEO1349-16	Patelloida pygmaea			
SSEO1304-16	Patelloida pygmaea			
SSEO1339-16	Patelloida pygmaea			
SSEO1329-16	Patelloida pygmaea			
SSEO1316-16	Patelloida pygmaea			
SSEO1337-16	Patelloida pygmaea			
SSEO1346-16	Patelloida pygmaea			
SSEO1342-16	Patelloida pygmaea			
SSEO1303-16	Patelloida pygmaea			
SSEO1358-16	Patelloida pygmaea			
SSEO1361-16	Patelloida pygmaea	Species	BOLD:AAB1669	54
SSEO1363-16	Patelloida pygmaea			
SSEO1331-16	Patelloida pygmaea			
SSEO1348-16	Patelloida pygmaea			
SSEO1245-16	Patelloida pygmaea			
SSEO1353-16	Patelloida pygmaea			
SSEO1347-16	Patelloida pygmaea			
SSEO1362-16	Patelloida pygmaea			
SSEO1302-16	Patelloida pygmaea			
SSEO1351-16	Patelloida pygmaea			
SSEO1359-16	Patelloida pygmaea			

SSEO1345-16	Patelloida pygmaea			
SSEO1360-16	Patelloida pygmaea			
SSEO1343-16	Patelloida pygmaea			
SSEO1246-16	Patelloida pygmaea			
QWEAS1537-15	Patelloida ryukyuensis	Species	BOLD:AAC5240	22
QWEAS1536-15	Patelloida ryukyuensis			
QWEAS421-15	Patelloida ryukyuensis			
QWEAS425-15	Patelloida ryukyuensis			
QWEAS420-15	Patelloida ryukyuensis			
QWEAS426-15	Patelloida ryukyuensis			
QWEAS423-15	Patelloida ryukyuensis			
QWEAS424-15	Patelloida ryukyuensis			
QWEAS422-15	Patelloida ryukyuensis			
QWEAS427-15	Patelloida ryukyuensis			
SSEO570-16	Patelloida signata	Species	BOLD:ACY9919	2
QWEAS1208-15	Periglypta chemnitzi	Species	BOLD:AAO8666	14
QWEAS1499-15	Periglypta chemnitzi			
QWEAS1205-15	Periglypta chemnitzi			
QWEAS1207-15	Periglypta chemnitzi			
QWEAS1206-15	Periglypta chemnitzi			
QWEAS054-15	Perna viridis	Species	BOLD:ACY1487	73
QWEAS051-15	Perna viridis			
QWEAS046-15	Perna viridis			
QWEAS052-15	Perna viridis			
QWEAS050-15	Perna viridis			
QWEAS047-15	Perna viridis			

QWEAS048-15	Perna viridis			
QWEAS049-15	Perna viridis			
QWEAS053-15	Perna viridis			
QWEAS528-15	Phalium bisulcatum	Species	BOLD:ACB7934	2
QWEAS529-15	Phalium flammiferum	Species	BOLD:ACB7935	2
SSEO1319-16	Phasianella solida	Species	BOLD:AAI4608	4
SSEO1320-16	Phasianella solida			
QWEAS097-15	Pholas orientalis	Species	BOLD:ACH8007	6
QWEAS098-15	Pholas orientalis			
QWEAS239-15	Phos senticosus	Species	BOLD:ACX3677	4
QWEAS241-15	Phos senticosus			
QWEAS242-15	Phos senticosus			
QWEAS240-15	Phos senticosus			
QWEAS839-15	Pinctada maxima	Species	BOLD:ACB3526	51
QWEAS1082-15	Pitar japonicum	Species	BOLD:ACH3330	3
SSEO1330-16	Pomaulax japonicus	Species	BOLD:AAH7899	4
SSEO1328-16	Pomaulax japonicus			
SSEO380-16	Protaeolidiella atra	Species	BOLD:ACV6117	4
SSEO378-16	Protaeolidiella atra			
QWEAS1502-15	Protothaca jedoensis	Species	BOLD:AAO5902	19
QWEAS1501-15	Protothaca jedoensis			
QWEAS945-15	Protothaca jedoensis			
QWEAS944-15	Protothaca jedoensis			
QWEAS940-15	Protothaca jedoensis			
QWEAS942-15	Protothaca jedoensis			
QWEAS943-15	Protothaca jedoensis			

QWEAS1300-15	Psammotaea elongata	Species	BOLD:ACQ2021	11
QWEAS1298-15	Psammotaea elongata			
QWEAS1303-15	Psammotaea elongata			
QWEAS1302-15	Psammotaea elongata			
QWEAS1295-15	Pseudocardium sachalinense	Species	BOLD:ACX7097	2
QWEAS1296-15	Pseudocardium sachalinense	Species	BOLD:ACI1599	2
SSEO790-16	Pseudostomatella decolorata	Species	BOLD:ACB7465	4
SSEO791-16	Pseudostomatella decolorata			
QWEAS863-15	Pteria sp.	Species	BOLD:ACX5542	3
QWEAS864-15	Pteria sp.			
QWEAS862-15	Pteria sp.			
SSEO1115-16	Rapana bezoar	Species	BOLD:ABV4540	38
QWEAS336-15	Rapana bezoar			
QWEAS335-15	Rapana bezoar			
QWEAS338-15	Rapana bezoar			
QWEAS337-15	Rapana bezoar			
QWEAS339-15	Rapana bezoar			
SSEO285-16	Roboastra luteolineata	Species	BOLD:AAI0870	3
SSEO789-16	Rossiteria nuclea	Species	BOLD:ACB7424	2
SSEO845-16	Sagamilepeta sagamiensis	Species	BOLD:ACB7707	4
SSEO844-16	Sagamilepeta sagamiensis			
QWEAS572-15	Sanguinolaria tchangsii	Species	BOLD:ACQ7492	5
QWEAS570-15	Sanguinolaria tchangsii			
QWEAS1438-15	Saxidomus purpuratus	Species	BOLD:AAI0674	46
QWEAS1440-15	Saxidomus purpuratus			
QWEAS1439-15	Saxidomus purpuratus			

QWEAS1441-15	Saxidomus purpuratus			
QWEAS921-15	Saxidomus purpuratus			
QWEAS919-15	Saxidomus purpuratus			
QWEAS922-15	Saxidomus purpuratus			
QWEAS916-15	Saxidomus purpuratus			
QWEAS918-15	Saxidomus purpuratus			
QWEAS920-15	Saxidomus purpuratus			
QWEAS917-15	Saxidomus purpuratus			
SSEO737-16	Scapharca broughtonii	Species	BOLD:AAA3571	26
SSEO735-16	Scapharca broughtonii			
SSEO766-16	Scapharca globosa	Species	BOLD:AAR4118	2
SSEO734-16	Scapharca satowi	Species	BOLD:AAI0660	2
SSEO999-16	Scutellastra flexuosa	Species	BOLD:AAH9728	2
SSEO987-16	Scutellastra optima	Species	BOLD:AAJ5791	2
SSEO029-15	Semele cf. amabilis ZY2012	Species	BOLD:ACQ8415	3
QWEAS585-15	Semele scabra	Species	BOLD:ACD2399	8
QWEAS584-15	Semele scabra			
QWEAS148-15	Sepia aculeata	Species	BOLD:AAH9790	14
QWEAS149-15	Sepia aculeata			
QWEAS146-15	Sepia aculeata			
QWEAS147-15	Sepia aculeata			
SSEO060-16	Sepia andreana	Species	BOLD:ABZ4460	2
SSEO059-16	Sepia aureomaculata	Species	BOLD:AAJ7621	2
SSEO061-16	Sepia esculenta	Species	BOLD:AAE9621	2
SSEO189-16	Sepia kobiensis	Species	BOLD:AAJ7651	2
SSEO063-16	Sepia latimanus	Species	BOLD:AAF0784	6

SSEO186-16	Sepia lycidas	Species	BOLD:ABA8975	9
QWEAS158-15	Sepia lycidas			
SSEO185-16	Sepia lycidas			
SSEO193-16	Sepia madokai	Species	BOLD:AAH9804	2
SSEO188-16	Sepia pardex	Species	BOLD:AAJ7638	2
SSEO187-16	Sepia peterseni	Species	BOLD:ABZ4217	2
QWEAS163-15	Sepia pharaonis	Species	BOLD:AAH9805	28
QWEAS160-15	Sepia pharaonis			
QWEAS165-15	Sepia pharaonis			
QWEAS161-15	Sepia pharaonis			
QWEAS162-15	Sepia pharaonis			
QWEAS159-15	Sepia pharaonis			
QWEAS164-15	Sepia pharaonis			
QWEAS167-15	Sepia recurvirostra	Species	BOLD:AAH9802	9
QWEAS166-15	Sepia recurvirostra			
QWEAS168-15	Sepia recurvirostra			
SSEO191-16	Sepia tokioensis	Species	BOLD:AAJ7615	2
QWEAS172-15	Sepiella inermis	Species	BOLD:AAO2726	7
QWEAS173-15	Sepiella inermis			
QWEAS176-15	Sepiola birostrata	Species	BOLD:AAJ0424	11
QWEAS175-15	Sepiola birostrata			
QWEAS178-15	Sepiola birostrata			
QWEAS177-15	Sepiola birostrata			
QWEAS179-15	Sepiola birostrata			
SSEO303-16	Sepiolina nipponensis	Species	BOLD:AAJ7587	3
SSEO304-16	Sepiolina petasa	Species	BOLD:ACH2726	2

SSEO542-16	Serratina capsoides	Species	BOLD:ACQ0043	6
SSEO544-16	Serratina capsoides			
SSEO540-16	Serratina capsoides			
QWEAS576-15	Serratina capsoides			
QWEAS575-15	Serratina capsoides			
QWEAS612-15	Siliqua radiata	Species	BOLD:ACQ2803	6
QWEAS611-15	Siliqua radiata			
QWEAS613-15	Siliqua radiata			
QWEAS614-15	Siliqua radiata	Species	BOLD:ACQ0765	2
SSEO1517-16	Sinonovacula constricta	Species	BOLD:AAA2942	111
SSEO1516-16	Sinonovacula constricta			
QWEAS599-15	Sinonovacula constricta			
QWEAS598-15	Sinonovacula constricta			
QWEAS602-15	Sinonovacula constricta			
QWEAS601-15	Sinonovacula constricta			
QWEAS600-15	Sinonovacula constricta			
QWEAS597-15	Sinonovacula constricta			
SSEO780-16	Solariella nyssonus	Species	BOLD:ACQ3687	4
SSEO781-16	Solariella nyssonus			
QWEAS594-15	Solecurtus divaricatus	Species	BOLD:ACQ0166	2
QWEAS621-15	Solen grandis	Species	BOLD:ACQ3781	6
QWEAS620-15	Solen grandis			
QWEAS619-15	Solen grandis	Species	BOLD:ACQ3780	2
QWEAS615-15	Solen strictus	Species	BOLD:ACQ5937	6
QWEAS616-15	Solen strictus			
QWEAS617-15	Solen strictus			

QWEAS618-15	Solen strictus	Species	BOLD:ACH5588	6
QWEAS1304-15	Soletellina virescens	Species	BOLD:ACQ3739	4
SSEO159-16	Stiliger ornatus	Species	BOLD:ACQ5939	2
SSEO160-16	Stiliger smaragdinus	Species	BOLD:ACQ5940	2
SSEO878-16	Stomatella impertusa	Species	BOLD:ACB7427	2
SSEO876-16	Stomatella planulata	Species	BOLD:AAF3287	6
SSEO874-16	Stomatella planulata			
SSEO875-16	Stomatella planulata			
SSEO877-16	Stomatella planulata	Species	BOLD:ACY9511	2
SSEO788-16	Stomatia obscura	Species	BOLD:ACB7092	2
SSEO787-16	Stomatia phymotis	Species	BOLD:AAJ4162	2
QWEAS512-15	Strombus luhuanus	Species	BOLD:AAH9999	12
QWEAS513-15	Strombus luhuanus			
QWEAS511-15	Strombus luhuanus			
QWEAS514-15	Strombus luhuanus			
QWEAS496-15	Strombus urceus	Species	BOLD:ACB6979	6
QWEAS494-15	Strombus urceus			
QWEAS495-15	Strombus urceus			
QWEAS502-15	Strombus vittatus	Species	BOLD:ACX3385	2
QWEAS504-15	Strombus vittatus	Species	BOLD:ACB8333	4
QWEAS503-15	Strombus vittatus			
QWEAS1505-15	Sunetta menstrualis	Species	BOLD:AAO8014	16
QWEAS1504-15	Sunetta menstrualis			
QWEAS1091-15	Sunetta menstrualis			
QWEAS1089-15	Sunetta menstrualis			
QWEAS1090-15	Sunetta menstrualis			

QWEAS1088-15	Sunetta menstrualis			
SSEO375-16	Tambja amakusana	Species	BOLD:AAK0292	2
SSEO376-16	Tambja limaciformis	Species	BOLD:AAK0293	2
SSEO292-16	Tambja sagamiana	Species	BOLD:AAK0295	2
QWEAS1461-15	Tapes dorsatus	Species	BOLD:AAK0248	28
QWEAS1460-15	Tapes dorsatus			
QWEAS1257-15	Tapes dorsatus			
QWEAS1102-15	Tapes dorsatus			
QWEAS1104-15	Tapes dorsatus			
QWEAS1101-15	Tapes dorsatus			
QWEAS1103-15	Tapes dorsatus			
QWEAS1105-15	Tapes dorsatus			
QWEAS1100-15	Tapes dorsatus			
QWEAS1099-15	Tapes dorsatus			
QWEAS1096-15	Tapes literata	Species	BOLD:AAO8786	31
QWEAS1095-15	Tapes literata			
QWEAS1094-15	Tapes literata			
QWEAS1258-15	Tapes literata			
QWEAS1259-15	Tapes literata			
QWEAS1462-15	Tapes literata			
QWEAS1463-15	Tapes literata			
QWEAS1092-15	Tapes literata			
QWEAS1093-15	Tapes literata			
QWEAS1098-15	Tapes literata			
QWEAS1260-15	Tapes literata			
QWEAS1097-15	Tapes literata			

SSEO1012-16	Tectarius spinulosus	Species	BOLD:ACY9257	2
SSEO1013-16	Tectarius spinulosus	Species	BOLD:AAK0770	2
SSEO1264-16	Terebralia sulcata	Species	BOLD:ACQ3189	11
SSEO1258-16	Terebralia sulcata			
SSEO1259-16	Terebralia sulcata			
SSEO1257-16	Terebralia sulcata			
QWEAS1618-15	Terebralia sulcata			
QWEAS352-15	Thais gradata	Species	BOLD:ACB7121	8
QWEAS353-15	Thais gradata			
SSEO157-16	Thuridilla gracilis	Species	BOLD:AAZ8673	3
SSEO158-16	Thuridilla splendens	Species	BOLD:ACH9448	2
QWEAS568-15	Tonna dolium	Species	BOLD:ACB8732	4
QWEAS569-15	Tonna dolium			
QWEAS565-15	Tonna galea	Species	BOLD:ACB8731	4
QWEAS566-15	Tonna galea			
QWEAS567-15	Tonna sulcosa	Species	BOLD:ACB8314	2
SSEO819-16	Trochus histrio	Species	BOLD:ACB7109	2
SSEO816-16	Trochus maculatus	Species	BOLD:AAH8276	7
SSEO817-16	Trochus maculatus			
SSEO815-16	Trochus maculatus			
SSEO818-16	Trochus stellatus	Species	BOLD:AAW7874	2
SSEO1336-16	Turbo chrysostomus	Species	BOLD:ACY9409	2
SSEO1341-16	Turbo marmoratus	Species	BOLD:AAF6524	5
SSEO1340-16	Turbo marmoratus			

SSEO1332-16	Turbo petholatus	Species	BOLD:AAD9725	4
SSEO1352-16	Turbo reevii	Species	BOLD:AAH7669	3
SSEO1335-16	Turbo setosus	Species	BOLD:AAD9686	3
SSEO1334-16	Turbo stenogyrus	Species	BOLD:AAH7676	4
SSEO1333-16	Turbo stenogyrus			
QWEAS327-15	Turricula javana	Species	BOLD:ACV8315	3
SSEO814-16	Umbonium giganteum	Species	BOLD:ACB7064	4
SSEO812-16	Umbonium giganteum			
SSEO811-16	Umbonium moniliferum	Species	BOLD:AAW8352	4
SSEO813-16	Umbonium moniliferum			
QWEAS1367-15	Uroteuthis chinensis	Species	BOLD:AAB5881	30
QWEAS1366-15	Uroteuthis chinensis			
QWEAS1368-15	Uroteuthis chinensis			
QWEAS1365-15	Uroteuthis chinensis			
QWEAS1364-15	Uroteuthis chinensis			
QWEAS1363-15	Uroteuthis chinensis			
QWEAS139-15	Uroteuthis chinensis			
QWEAS1357-15	Uroteuthis duvaucelii	Species	BOLD:AAE3352	32
QWEAS1355-15	Uroteuthis duvaucelii			
QWEAS1356-15	Uroteuthis duvaucelii			
QWEAS137-15	Uroteuthis duvaucelii			
QWEAS132-15	Uroteuthis duvaucelii			
QWEAS134-15	Uroteuthis duvaucelii			
QWEAS133-15	Uroteuthis duvaucelii			

QWEAS131-15	Uroteuthis duvaucelii			
QWEAS135-15	Uroteuthis duvaucelii			
QWEAS136-15	Uroteuthis duvaucelii			
SSEO050-16	Uroteuthis edulis	Species	BOLD:AAB8873	44
SSEO054-16	Uroteuthis edulis			
SSEO046-16	Uroteuthis edulis			
SSEO040-16	Uroteuthis edulis			
SSEO052-16	Uroteuthis edulis			
SSEO042-16	Uroteuthis edulis			
SSEO053-16	Uroteuthis edulis			
SSEO051-16	Uroteuthis edulis			
SSEO055-16	Uroteuthis edulis			
SSEO043-16	Uroteuthis edulis			
SSEO047-16	Uroteuthis edulis			
SSEO049-16	Uroteuthis edulis			
SSEO048-16	Uroteuthis edulis			
SSEO044-16	Uroteuthis edulis			
SSEO045-16	Uroteuthis edulis			
SSEO041-16	Uroteuthis edulis			
QWEAS1358-15	Uroteuthis edulis			
QWEAS1362-15	Uroteuthis edulis			
QWEAS1360-15	Uroteuthis edulis			
QWEAS1361-15	Uroteuthis edulis			
QWEAS1359-15	Uroteuthis edulis			
QWEAS1558-15	Varicinassa variciferus	Species	BOLD:ACB8189	44
QWEAS1559-15	Varicinassa variciferus			

QWEAS1555-15	Varicinassa variciferus			
QWEAS1560-15	Varicinassa variciferus			
QWEAS1557-15	Varicinassa variciferus			
QWEAS1556-15	Varicinassa variciferus			
QWEAS630-15	Vasticardium flavum	Species	BOLD:ACX4007	2
QWEAS625-15	Vasticardium flavum	Species	BOLD:ACQ2883	8
QWEAS628-15	Vasticardium flavum			
QWEAS627-15	Vasticardium flavum			
QWEAS626-15	Vasticardium flavum			
QWEAS629-15	Vasticardium flavum	Species	BOLD:ACQ2882	2
QWEAS1321-15	Vasum turbinellus	Species	BOLD:ACX7608	2
QWEAS1320-15	Vasum turbinellus			
QWEAS623-15	Vepricardium coronatum	Species	BOLD:ACQ0321	8
QWEAS624-15	Vepricardium coronatum			
QWEAS622-15	Vepricardium coronatum			

Supplementary Table 5. BINs with taxonomic discordance.

Process ID	Identification	Rank of Conflict	BIN
SSEO406-16	Herpetopoma pauperculus	Species	BOLD:ACY9302
QWEAS1319-15	Moerella iridescens	Species	BOLD:ACX7266
SSEO573-16	Patelloida striata	Species	BOLD:ACY9706
QWEAS865-15	Pteria loveni	Species	BOLD:ACX5453
QWEAS328-15	Gemmula deshayesii	Species	BOLD:ACX3938

Supplementary Table 6. BINs with Single Specimens.

Process ID	Identification	Conflicting Taxon in BIN	Rank of Conflict	BIN	BIN Total Members	BIN Tax Variation
SSEO697-16	Crassostrea gigas	Mollusca	Phylum	BOLD:AAB2297	103	Mollusca[104], Arthropoda[2]
SSEO698-16	Crassostrea gigas	Mollusca				
SSEO696-16	Crassostrea gigas	Mollusca				
QWEAS004-15	Crassostrea gigas	Mollusca				
QWEAS003-15	Crassostrea gigas	Mollusca				
QWEAS006-15	Crassostrea gigas	Mollusca				
QWEAS005-15	Crassostrea gigas	Mollusca				
QWEAS002-15	Crassostrea gigas	Mollusca				
QWEAS001-15	Crassostrea gigas	Mollusca				
SSEO1395-16	Arca avellana	Arcoida	Order	BOLD:AAY3811	7	Veneroida[4], Arcoida[3]
SSEO1396-16	Arca avellana	Arcoida				
SSEO1397-16	Arca avellana	Arcoida				
QWEAS483-15	Clypeomorus humilis	Sorbeoconcha	Order	BOLD:AAO8512	13	Mesogastropoda[12], Sorbeoconcha[1]
QWEAS469-15	Planaxis sulcatus	Mesogastropoda				
QWEAS473-15	Planaxis sulcatus	Mesogastropoda				
QWEAS471-15	Planaxis sulcatus	Mesogastropoda				
QWEAS468-15	Planaxis sulcatus	Mesogastropoda				
QWEAS470-15	Planaxis sulcatus	Mesogastropoda				
SSEO1262-16	Lunella coreensis	Gastropoda_order_incertae_sedis	Order	BOLD:AAE3868	38	Gastropoda_order_incertae_sedis[36], Archaeogastropoda[1]
SSEO1439-16	Lunella coreensis	Gastropoda_order_incertae_sedis				
SSEO1437-16	Lunella coreensis	Gastropoda_order_incertae_sedis				
SSEO1269-16	Lunella coreensis	Gastropoda_order_incer				

		tae_sedis				
SSEO1263-16	Lunella coreensis	Gastropoda_order_incertae_sedis				
SSEO1438-16	Lunella coreensis	Gastropoda_order_incertae_sedis				
SSEO1267-16	Lunella coreensis	Gastropoda_order_incertae_sedis				
SSEO1268-16	Lunella coreensis	Gastropoda_order_incertae_sedis				
SSEO1265-16	Lunella coreensis	Gastropoda_order_incertae_sedis				
SSEO1266-16	Lunella coreensis	Gastropoda_order_incertae_sedis				
SSEO1283-16	Lunella moniliformis	Gastropoda_order_incertae_sedis				
SSEO1285-16	Lunella moniliformis	Gastropoda_order_incertae_sedis				
SSEO1284-16	Lunella moniliformis	Gastropoda_order_incertae_sedis				
SSEO1125-16	Morula purpureocincta	Neogastropoda	Order	BOLD:ACH8830	4	Neogastropoda[3], Littorinimorpha[1]
SSEO190-16	Sepia subtenuipes	Sepiida	Order	BOLD:AAI3631	3	Sepiida[1], Octopoda[1]
SSEO1381-16	Acanthochitona defilippi	Acanthochitonidae	Family	BOLD:AAE6153	7	Acanthochitonidae[6], Chitonidae[1]
SSEO1385-16	Acanthochitona defilippi	Acanthochitonidae				
SSEO1382-16	Acanthochitona defilippi	Acanthochitonidae				
SSEO1383-16	Acanthochitona defilippi	Acanthochitonidae	Family	BOLD:AAE6152	7	Chitonidae[5], Acanthochitonidae[2]
SSEO907-16	Cellana toreuma	Nacellidae	Family	BOLD:AAI7335	532	Nacellidae[511], Acmaeidae[12]
SSEO1370-16	Cellana toreuma	Nacellidae				
SSEO891-16	Cellana toreuma	Nacellidae				

SSEO909-16	Cellana toreuma	Nacellidae				
SSEO888-16	Cellana toreuma	Nacellidae				
SSEO1369-16	Cellana toreuma	Nacellidae				
SSEO906-16	Cellana toreuma	Nacellidae				
SSEO911-16	Cellana toreuma	Nacellidae				
SSEO908-16	Cellana toreuma	Nacellidae				
SSEO904-16	Cellana toreuma	Nacellidae				
SSEO889-16	Cellana toreuma	Nacellidae				
SSEO890-16	Cellana toreuma	Nacellidae				
SSEO910-16	Cellana toreuma	Nacellidae				
SSEO905-16	Cellana toreuma	Nacellidae				
QWEAS1328-15	Cellana toreuma	Nacellidae				
QWEAS1334-15	Cellana toreuma	Nacellidae				
QWEAS1331-15	Cellana toreuma	Nacellidae				
QWEAS1335-15	Cellana toreuma	Nacellidae				
QWEAS1336-15	Cellana toreuma	Nacellidae				
QWEAS1332-15	Cellana toreuma	Nacellidae				
QWEAS1330-15	Cellana toreuma	Nacellidae				
QWEAS1333-15	Cellana toreuma	Nacellidae				
QWEAS1329-15	Cellana toreuma	Nacellidae				
SSEO1469-16	Notoacmea schrenckii	Acmaeidae				
SSEO1473-16	Notoacmea schrenckii	Acmaeidae				
SSEO1472-16	Notoacmea schrenckii	Acmaeidae				
SSEO1470-16	Notoacmea schrenckii	Acmaeidae				
SSEO1468-16	Notoacmea schrenckii	Acmaeidae				
SSEO1471-16	Notoacmea schrenckii	Acmaeidae				
QWEAS482-15	Clypeomorus humilis	Cerithiidae	Family	BOLD:ACB8597	4	Cerithiidae[3], Pleuroceridae[1]
QWEAS321-15	Conus aristophanes	Conidae	Family	BOLD:AAJ7375	7	Conidae[6], Columbellidae[1]
QWEAS257-15	Euplica scripta	Columbellidae				
QWEAS1377-15	Mactra veneriformis	Mactridae	Family	BOLD:AAB4298	206	Mactridae[204], Veneridae[2]

QWEAS1383-15	Mactra veneriformis	Mactridae				
QWEAS1379-15	Mactra veneriformis	Mactridae				
QWEAS1381-15	Mactra veneriformis	Mactridae				
QWEAS1385-15	Mactra veneriformis	Mactridae				
QWEAS1388-15	Mactra veneriformis	Mactridae				
QWEAS1386-15	Mactra veneriformis	Mactridae				
QWEAS1387-15	Mactra veneriformis	Mactridae				
QWEAS1378-15	Mactra veneriformis	Mactridae				
QWEAS1382-15	Mactra veneriformis	Mactridae				
QWEAS1375-15	Mactra veneriformis	Mactridae				
QWEAS1384-15	Mactra veneriformis	Mactridae				
QWEAS1376-15	Mactra veneriformis	Mactridae				
QWEAS1380-15	Mactra veneriformis	Mactridae				
QWEAS1261-15	Mactra veneriformis	Mactridae				
QWEAS1265-15	Mactra veneriformis	Mactridae				
QWEAS1264-15	Mactra veneriformis	Mactridae				
QWEAS1263-15	Mactra veneriformis	Mactridae				
QWEAS1262-15	Mactra veneriformis	Mactridae				
QWEAS1503-15	Protothaca jedoensis	Veneridae				
QWEAS1405-15	Meretrix lusoria	Veneridae	Family	BOLD:AAC6197	87	Veneridae[82], Mactridae[1]
SSEO565-16	Meretrix lusoria	Veneridae				
QWEAS1447-15	Meretrix meretrix	Veneridae				
QWEAS1449-15	Meretrix meretrix	Veneridae				
QWEAS1442-15	Meretrix meretrix	Veneridae				
QWEAS1444-15	Meretrix meretrix	Veneridae				
QWEAS1448-15	Meretrix meretrix	Veneridae				
QWEAS1410-15	Meretrix petechialis	Veneridae				
QWEAS1412-15	Meretrix petechialis	Veneridae				
QWEAS1042-15	Meretrix petechialis	Veneridae				
QWEAS1047-15	Meretrix petechialis	Veneridae				

QWEAS1041-15	Meretrix petechialis	Veneridae				
QWEAS1039-15	Meretrix petechialis	Veneridae				
QWEAS1040-15	Meretrix petechialis	Veneridae				
QWEAS1044-15	Meretrix petechialis	Veneridae				
QWEAS1046-15	Meretrix petechialis	Veneridae				
QWEAS1045-15	Meretrix petechialis	Veneridae				
QWEAS1043-15	Meretrix petechialis	Veneridae				
QWEAS251-15	Neptunea cumingi	Buccinidae	Family	BOLD:ACF4243	12	Muricidae[6], Buccinidae[6]
QWEAS252-15	Neptunea cumingi	Buccinidae				
QWEAS253-15	Neptunea cumingi	Buccinidae				
SSEO1398-16	Ocinebrellus inornatus	Muricidae				
SSEO1399-16	Ocinebrellus inornatus	Muricidae				
SSEO1400-16	Ocinebrellus inornatus	Muricidae				
QWEAS477-15	Rhinoclavis sinensis	Cerithiidae	Family	BOLD:AAA7590	7	Cerithiidae[6], Batillariidae[1]
QWEAS478-15	Rhinoclavis sinensis	Cerithiidae				
QWEAS479-15	Rhinoclavis sinensis	Cerithiidae				
SSEO402-16	Adipicola iwaotakii	Adipicola	Genus	BOLD:AAA7140	103	Adipicola[109], Idas[1]
SSEO407-16	Adipicola iwaotakii	Adipicola				
SSEO399-16	Adipicola iwaotakii	Adipicola				
SSEO398-16	Adipicola iwaotakii	Adipicola				
SSEO397-16	Adipicola iwaotakii	Adipicola				
QWEAS201-15	Amphioctopus kagoshimensis	Amphioctopus	Genus	BOLD:ABA8783	15	Amphioctopus[11], Octopus[4]
QWEAS199-15	Amphioctopus kagoshimensis	Amphioctopus				
QWEAS198-15	Amphioctopus kagoshimensis	Amphioctopus				
QWEAS200-15	Amphioctopus kagoshimensis	Amphioctopus				
SSEO248-16	Amphioctopus kagoshimensis	Amphioctopus				
SSEO250-16	Amphioctopus marginatus	Amphioctopus	Genus	BOLD:AAF4331	29	Amphioctopus[19], Octopus[10]
SSEO252-16	Amphioctopus marginatus	Amphioctopus				
QWEAS202-15	Amphioctopus marginatus	Amphioctopus				
QWEAS205-15	Amphioctopus marginatus	Amphioctopus				

QWEAS204-15	Amphioctopus marginatus	Amphioctopus				
QWEAS203-15	Amphioctopus marginatus	Amphioctopus				
QWEAS208-15	Amphioctopus ovulum	Amphioctopus	Genus	BOLD:AAJ2313	19	Amphioctopus[15], Octopus[4]
QWEAS206-15	Amphioctopus ovulum	Amphioctopus				
QWEAS209-15	Amphioctopus ovulum	Amphioctopus				
QWEAS207-15	Amphioctopus ovulum	Amphioctopus				
SSEO254-16	Amphioctopus ovulum	Amphioctopus				
SSEO255-16	Amphioctopus ovulum	Amphioctopus				
SSEO238-16	Callistoctopus aspilosomatis	Callistoctopus	Genus	BOLD:AAW8735	17	Callistoctopus[15], Octopus[3]
QWEAS223-15	Callistoctopus minor	Callistoctopus	Genus	BOLD:AAI3616	33	Callistoctopus[17], Octopus[13], Amphioctopus[1]
QWEAS218-15	Callistoctopus minor	Callistoctopus				
QWEAS220-15	Callistoctopus minor	Callistoctopus				
SSEO236-16	Callistoctopus minor	Callistoctopus				
QWEAS219-15	Callistoctopus minor	Callistoctopus				
QWEAS222-15	Callistoctopus minor	Callistoctopus				
QWEAS221-15	Callistoctopus minor	Callistoctopus				
SSEO234-16	Callistoctopus ornatus	Callistoctopus	Genus	BOLD:AAI6847	5	Callistoctopus[4], Hapalochlaena[1]
SSEO682-16	Calyptogena extenta	Calyptogena	Genus	BOLD:AAD2536	8	Ectenagena[4], Calyptogena[4]
SSEO1251-16	Cerithidea alata	Cerithidea	Genus	BOLD:ACH9667	22	Cerithidea[16], Cerithideopsilla[6]
SSEO1252-16	Cerithidea alata	Cerithidea				
SSEO1377-16	Cerithidea alata	Cerithidea				
SSEO1253-16	Cerithidea alata	Cerithidea				
SSEO1250-16	Cerithidea alata	Cerithidea				
SSEO1254-16	Cerithidea alata	Cerithidea				
SSEO1248-16	Cerithidea alata	Cerithidea				
SSEO1249-16	Cerithidea alata	Cerithidea				
SSEO1177-16	Cerithidea cingulata	Cerithidea	Genus	BOLD:AAA7612	117	Cerithidea[115], Cerithideopsilla[2]
SSEO1208-16	Cerithidea cingulata	Cerithidea				
SSEO1197-16	Cerithidea cingulata	Cerithidea				

SSEO1198-16	Cerithidea cingulata	Cerithidea				
SSEO1201-16	Cerithidea cingulata	Cerithidea				
SSEO1200-16	Cerithidea cingulata	Cerithidea				
SSEO1203-16	Cerithidea cingulata	Cerithidea				
SSEO1247-16	Cerithidea cingulata	Cerithidea				
SSEO1209-16	Cerithidea cingulata	Cerithidea				
SSEO1214-16	Cerithidea cingulata	Cerithidea				
SSEO1212-16	Cerithidea cingulata	Cerithidea				
SSEO1202-16	Cerithidea cingulata	Cerithidea				
SSEO1211-16	Cerithidea cingulata	Cerithidea				
SSEO1207-16	Cerithidea cingulata	Cerithidea				
SSEO1206-16	Cerithidea cingulata	Cerithidea				
SSEO1213-16	Cerithidea cingulata	Cerithidea				
SSEO1210-16	Cerithidea cingulata	Cerithidea				
SSEO1199-16	Cerithidea cingulata	Cerithidea				
SSEO1196-16	Cerithidea cingulata	Cerithidea				
SSEO1164-16	Cerithidea cingulata	Cerithidea				
SSEO1204-16	Cerithidea cingulata	Cerithidea				
SSEO1364-16	Cerithidea cingulata	Cerithidea				
SSEO1205-16	Cerithidea cingulata	Cerithidea				
SSEO1143-16	Cerithidea djadjariensis	Cerithidea	Genus	BOLD:AAB1673	93	Cerithidea[88], Cerithideopsilla[5]
SSEO1167-16	Cerithidea djadjariensis	Cerithidea				
SSEO1146-16	Cerithidea djadjariensis	Cerithidea				
SSEO1175-16	Cerithidea djadjariensis	Cerithidea				
SSEO1171-16	Cerithidea djadjariensis	Cerithidea				
SSEO1144-16	Cerithidea djadjariensis	Cerithidea				
SSEO1148-16	Cerithidea djadjariensis	Cerithidea				
SSEO1151-16	Cerithidea djadjariensis	Cerithidea				
SSEO1172-16	Cerithidea djadjariensis	Cerithidea				
SSEO1168-16	Cerithidea djadjariensis	Cerithidea				

SSEO1374-16	Cerithidea djadjariensis	Cerithidea				
SSEO1183-16	Cerithidea djadjariensis	Cerithidea				
SSEO1186-16	Cerithidea djadjariensis	Cerithidea				
SSEO1178-16	Cerithidea djadjariensis	Cerithidea				
SSEO1169-16	Cerithidea djadjariensis	Cerithidea				
SSEO1173-16	Cerithidea djadjariensis	Cerithidea				
SSEO1184-16	Cerithidea djadjariensis	Cerithidea				
SSEO1147-16	Cerithidea djadjariensis	Cerithidea				
SSEO1166-16	Cerithidea djadjariensis	Cerithidea				
SSEO1142-16	Cerithidea djadjariensis	Cerithidea				
SSEO1165-16	Cerithidea djadjariensis	Cerithidea				
SSEO1180-16	Cerithidea djadjariensis	Cerithidea				
SSEO1150-16	Cerithidea djadjariensis	Cerithidea				
SSEO1174-16	Cerithidea djadjariensis	Cerithidea				
SSEO1170-16	Cerithidea djadjariensis	Cerithidea				
SSEO1179-16	Cerithidea djadjariensis	Cerithidea				
SSEO1149-16	Cerithidea djadjariensis	Cerithidea				
SSEO1176-16	Cerithidea djadjariensis	Cerithidea				
SSEO1181-16	Cerithidea djadjariensis	Cerithidea				
SSEO1145-16	Cerithidea djadjariensis	Cerithidea				
SSEO1185-16	Cerithidea djadjariensis	Cerithidea				
SSEO1187-16	Cerithidea largillierti	Cerithidea	Genus	BOLD:AAB4911	11	Cerithidea[9], Cerithideopsis[2]
QWEAS1528-15	Chlamys farreri	Chlamys	Genus	BOLD:AAE9030	7	Azumapecten[99], Chlamys[3]
SSEO1417-16	Chlorostoma turbinatum	Chlorostoma				
SSEO1407-16	Chlorostoma turbinatum	Chlorostoma				
SSEO1405-16	Chlorostoma turbinatum	Chlorostoma				
SSEO1413-16	Chlorostoma turbinatum	Chlorostoma	Genus	BOLD:AAI1477	41	Chlorostoma[30], Omphalius[11]
SSEO1418-16	Chlorostoma turbinatum	Chlorostoma				
SSEO1410-16	Chlorostoma turbinatum	Chlorostoma				
SSEO1406-16	Chlorostoma turbinatum	Chlorostoma				

SSEO1412-16	Chlorostoma turbinatum	Chlorostoma				
SSEO1403-16	Chlorostoma turbinatum	Chlorostoma				
SSEO1409-16	Chlorostoma turbinatum	Chlorostoma				
SSEO1411-16	Chlorostoma turbinatum	Chlorostoma				
SSEO1408-16	Chlorostoma turbinatum	Chlorostoma				
SSEO1414-16	Chlorostoma turbinatum	Chlorostoma				
SSEO1404-16	Chlorostoma turbinatum	Chlorostoma				
SSEO1416-16	Chlorostoma turbinatum	Chlorostoma				
SSEO1483-16	Omphalius rusticus	Omphalius				
SSEO1482-16	Omphalius rusticus rusticus	Omphalius				
SSEO1481-16	Omphalius rusticus rusticus	Omphalius				
QWEAS931-15	Placamen isabellina	Clausinella	Genus	BOLD:AAO8692	16	Clausinella[11], Placamen[5]
QWEAS929-15	Placamen isabellina	Clausinella				
QWEAS927-15	Placamen isabellina	Clausinella				
QWEAS930-15	Placamen isabellina	Clausinella				
QWEAS928-15	Placamen isabellina	Clausinella				
QWEAS084-15	Corbula amurensis	Corbula	Genus	BOLD:ACH5927	104	Corbula[62], Potamocorbula[42]
QWEAS080-15	Corbula amurensis	Corbula				
QWEAS087-15	Corbula amurensis	Corbula				
QWEAS083-15	Corbula amurensis	Corbula				
QWEAS078-15	Corbula amurensis	Corbula				
QWEAS090-15	Corbula amurensis	Corbula				
QWEAS086-15	Corbula amurensis	Corbula				
QWEAS082-15	Corbula amurensis	Corbula				
QWEAS088-15	Corbula amurensis	Corbula				
QWEAS081-15	Corbula amurensis	Corbula				
QWEAS085-15	Corbula amurensis	Corbula				
QWEAS079-15	Corbula amurensis	Corbula				
QWEAS908-15	Costacallista erycina	Costacallista	Genus	BOLD:AAK2197	18	Costacallista[13], Callista[5]
QWEAS909-15	Costacallista erycina	Costacallista				

QWEAS1458-15	Costacallista erycina	Costacallista				
QWEAS1527-15	Costacallista erycina	Costacallista				
QWEAS1459-15	Costacallista erycina	Costacallista				
QWEAS906-15	Costacallista erycina	Costacallista				
QWEAS907-15	Costacallista erycina	Costacallista				
QWEAS519-15	Cypraea vitellus	Cypraea	Genus	BOLD:AAJ2598	6	Cypraea[3], Lyncina[2]
QWEAS1024-15	Dosinia angulosa	Dosinia	Genus	BOLD:AAO9166	11	Dosinia[9], Dosinella[3]
QWEAS1025-15	Dosinia angulosa	Dosinia				
QWEAS1026-15	Dosinia angulosa	Dosinia				
QWEAS1023-15	Dosinia angulosa	Dosinia				
QWEAS1022-15	Dosinia corrugata	Dosinia	Genus	BOLD:AAO5907	25	Dosinia[18], Dosinella[7]
QWEAS1019-15	Dosinia corrugata	Dosinia				
QWEAS1470-15	Dosinia corrugata	Dosinia				
QWEAS1469-15	Dosinia corrugata	Dosinia				
QWEAS1020-15	Dosinia corrugata	Dosinia				
QWEAS1016-15	Dosinia corrugata	Dosinia				
QWEAS1017-15	Dosinia corrugata	Dosinia				
QWEAS1021-15	Dosinia corrugata	Dosinia				
QWEAS1018-15	Dosinia corrugata	Dosinia				
QWEAS1013-15	Dosinia japonica	Dosinia	Genus	BOLD:AAL1648	12	Dosinia[8], Phymosoma[3], Dosinella[1]
QWEAS1012-15	Dosinia japonica	Dosinia				
SSEO1093-16	Drupella fragum	Drupella	Genus	BOLD:AAE1638	26	Drupella[25], Coralliophila[1]
SSEO1091-16	Drupella fragum	Drupella				
SSEO1085-16	Drupella fragum	Drupella				
SSEO1090-16	Drupella fragum	Drupella				
SSEO1092-16	Drupella fragum	Drupella				
SSEO1088-16	Drupella fragum	Drupella				
SSEO1083-16	Drupella fragum	Drupella				
SSEO1087-16	Drupella fragum	Drupella				
SSEO1086-16	Drupella fragum	Drupella				

SSEO1084-16	Drupella fragum	Drupella				
SSEO1089-16	Drupella fragum	Drupella				
SSEO1082-16	Drupella fragum	Drupella				
QWEAS1510-15	Drupella margariticola	Drupella	Genus	BOLD:AAD8264	52	Drupella[47], Ergalatax[5]
QWEAS1508-15	Drupella margariticola	Drupella				
QWEAS1509-15	Drupella margariticola	Drupella				
QWEAS1507-15	Drupella margariticola	Drupella				
QWEAS392-15	Drupella margariticola	Drupella				
QWEAS391-15	Drupella margariticola	Drupella				
QWEAS292-15	Drupella margariticola	Drupella				
QWEAS390-15	Drupella margariticola	Drupella				
QWEAS393-15	Drupella margariticola	Drupella				
QWEAS293-15	Drupella margariticola	Drupella				
SSEO1106-16	Drupella margariticola	Drupella				
SSEO1107-16	Drupella margariticola	Drupella	Genus	BOLD:AAD8263	42	Ergalatax[7], Drupella[6], Morula[5]
SSEO1105-16	Drupella margariticola	Drupella				
QWEAS1583-15	Echinolittorina trochoides A	Echinolittorina	Genus	BOLD:AAA7808	35	Echinolittorina[33], Nodilittorina[2]
QWEAS515-15	Erronea errones	Erronea	Genus	BOLD:AAF2702	6	Erronea[5], Blasicrura[1]
SSEO308-16	Gonatus onyx	Gonatus	Genus	BOLD:AAD4845	11	Gonatus[10], Gonatopsis[1]
SSEO352-16	Hapalochlaena lunulata	Hapalochlaena	Genus	BOLD:AAI3607	3	Hapalochlaena[2], Callistoctopus[1]
SSEO474-16	Idasola japonica	Idasola	Genus	BOLD:AAB6925	19	Idas[19], Idasola[1]
QWEAS1464-15	Katelysia hiantina	Katelysia	Genus	BOLD:AAO7358	36	Katelysia[27], Marcia[9]
QWEAS1465-15	Katelysia hiantina	Katelysia				
QWEAS1167-15	Katelysia hiantina	Katelysia				
QWEAS1166-15	Katelysia hiantina	Katelysia				
QWEAS1165-15	Katelysia hiantina	Katelysia				
QWEAS1236-15	Katelysia hiantina	Katelysia				
QWEAS1169-15	Katelysia hiantina	Katelysia				
QWEAS1164-15	Katelysia hiantina	Katelysia				

QWEAS1162-15	Katelysia hiantina	Katelysia				
QWEAS1237-15	Katelysia hiantina	Katelysia				
QWEAS1163-15	Katelysia hiantina	Katelysia				
QWEAS1235-15	Katelysia hiantina	Katelysia				
QWEAS1168-15	Katelysia hiantina	Katelysia				
QWEAS531-15	Lunatia alderi	Lunatia	Genus	BOLD:ACB8212	6	Laguncula[4], Notocochlis[1], Lunatia[1]
QWEAS532-15	Lunatia grossularia	Lunatia	Genus	BOLD:ACB6964	2	Polinices[1], Lunatia[1]
QWEAS1254-15	Macridiscus aequilatera	Macridiscus				
QWEAS1253-15	Macridiscus aequilatera	Macridiscus				
QWEAS1255-15	Macridiscus aequilatera	Macridiscus				
QWEAS1252-15	Macridiscus aequilatera	Macridiscus				
QWEAS1256-15	Macridiscus aequilatera	Macridiscus				
QWEAS1152-15	Macridiscus semicancellata	Macridiscus				
QWEAS1155-15	Macridiscus semicancellata	Macridiscus				
QWEAS1151-15	Macridiscus semicancellata	Macridiscus				
QWEAS1427-15	Macridiscus semicancellata	Macridiscus				
QWEAS1429-15	Macridiscus semicancellata	Macridiscus				
QWEAS1426-15	Macridiscus semicancellata	Macridiscus	Genus	BOLD:AAO8016	141	Macridiscus[127], Gomphina[7]
QWEAS1422-15	Macridiscus semicancellata	Macridiscus				
QWEAS1428-15	Macridiscus semicancellata	Macridiscus				
QWEAS1424-15	Macridiscus semicancellata	Macridiscus				
QWEAS1425-15	Macridiscus semicancellata	Macridiscus				
QWEAS1150-15	Macridiscus semicancellata	Macridiscus				
QWEAS1156-15	Macridiscus semicancellata	Macridiscus				
QWEAS1154-15	Macridiscus semicancellata	Macridiscus				
QWEAS1153-15	Macridiscus semicancellata	Macridiscus				
QWEAS1423-15	Macridiscus semicancellata	Macridiscus				
QWEAS1148-15	Macridiscus melanaegis	Macridiscus	Genus	BOLD:AAL3879	15	Macridiscus[10], Gomphina[3]
QWEAS1149-15	Macridiscus melanaegis	Macridiscus				

SSEO1112-16	Mancinella siro	Mancinella	Genus	BOLD:AAO9096	3	Mancinella[2], Thais[1]
QWEAS1174-15	Marcia marmorata	Marcia	Genus	BOLD:AAO7359	25	Marcia[24], Tapes[1]
QWEAS1176-15	Marcia marmorata	Marcia				
QWEAS1171-15	Marcia marmorata	Marcia				
QWEAS1172-15	Marcia marmorata	Marcia				
QWEAS1178-15	Marcia marmorata	Marcia				
QWEAS1173-15	Marcia marmorata	Marcia				
QWEAS1177-15	Marcia marmorata	Marcia				
QWEAS1175-15	Marcia marmorata	Marcia				
SSEO1134-16	Morula spinosa	Morula	Genus	BOLD:AAR4788	8	Morula[5], Cronia[1]
SSEO1133-16	Morula spinosa	Morula				
QWEAS294-15	Nassarius siquijorensis	Nassarius	Genus	BOLD:AAE0953	8	Nassarius[6], Zeuxis[2]
QWEAS295-15	Nassarius siquijorensis	Nassarius				
QWEAS296-15	Nassarius siquijorensis	Nassarius	Genus	BOLD:AAE0952	7	Nassarius[4], Zeuxis[3]
QWEAS533-15	Natica tigrina	Natica	Genus	BOLD:ACB8211	2	Notocochlis[1], Natica[1]
QWEAS1308-15	Nuttallia olivacea	Nuttallia	Genus	BOLD:ACQ3764	6	Soletellina[4], Nuttallia[2]
QWEAS1309-15	Nuttallia olivacea	Nuttallia				
SSEO341-16	Octopus longispadiceus	Octopus	Genus	BOLD:AAD5241	13	Muusoctopus[10], Octopus[3]
QWEAS1345-15	Octopus oshimai	Octopus	Genus	BOLD:AAB0289	25	Octopus[23], Amphioctopus[1]
QWEAS225-15	Octopus vulgaris	Octopus				
SSEO336-16	Octopus vulgaris	Octopus				
SSEO335-16	Octopus vulgaris	Octopus				
QWEAS1343-15	Octopus vulgaris	Octopus				
QWEAS226-15	Octopus vulgaris	Octopus				
QWEAS1349-15	Octopus tankahkeei	Octopus	Genus	BOLD:ABA8846	18	Cistopus[16], Octopus[2]
SSEO728-16	Ostrea stentina	Ostrea	Genus	BOLD:AAD3640	29	Ostrea[21], Ostreola[7]
SSEO725-16	Ostrea stentina	Ostrea				
SSEO729-16	Ostrea stentina	Ostrea				
SSEO724-16	Ostrea stentina	Ostrea				
SSEO731-16	Ostrea stentina	Ostrea				

SSEO723-16	Ostrea stentina	Ostrea				
SSEO722-16	Ostrea stentina	Ostrea				
SSEO730-16	Ostrea stentina	Ostrea				
SSEO726-16	Ostrea stentina	Ostrea				
SSEO727-16	Ostrea stentina	Ostrea	Genus	BOLD:AAD5609	6	Ostreola[4], Ostrea[2]
QWEAS1126-15	Paphia amabilis	Paphia	Genus	BOLD:AAO8676	56	Paphia[53], Meretrix[1]
QWEAS1131-15	Paphia amabilis	Paphia				
QWEAS1127-15	Paphia amabilis	Paphia				
QWEAS1134-15	Paphia amabilis	Paphia				
QWEAS1136-15	Paphia amabilis	Paphia				
QWEAS1133-15	Paphia amabilis	Paphia				
QWEAS1124-15	Paphia amabilis	Paphia				
QWEAS1135-15	Paphia amabilis	Paphia				
QWEAS1130-15	Paphia amabilis	Paphia				
QWEAS1234-15	Paphia amabilis	Paphia				
QWEAS1132-15	Paphia amabilis	Paphia				
QWEAS1129-15	Paphia amabilis	Paphia				
QWEAS1233-15	Paphia amabilis	Paphia				
QWEAS1125-15	Paphia amabilis	Paphia				
QWEAS1232-15	Paphia amabilis	Paphia				
QWEAS1128-15	Paphia amabilis	Paphia				
QWEAS1231-15	Paphia amabilis	Paphia				
QWEAS1137-15	Paphia amabilis	Paphia				
QWEAS932-15	Placamen calophylla	Placamen	Genus	BOLD:AAJ3245	12	Placamen[11], Clausinella[1]
QWEAS934-15	Placamen calophylla	Placamen				
QWEAS933-15	Placamen calophylla	Placamen				
QWEAS1582-15	Platevindex mortoni	Platevindex	Genus	BOLD:AAM1753	35	Platevindex[23], Onchidium[11]
QWEAS1581-15	Platevindex mortoni	Platevindex				
QWEAS1578-15	Platevindex mortoni	Platevindex				
QWEAS1580-15	Platevindex mortoni	Platevindex				

QWEAS1577-15	Platevindex mortoni	Platevindex				
SSEO1130-16	Reishia bronni	Reishia	Genus	BOLD:ACB7390	81	Thais[59], Reishia[22]
SSEO1117-16	Reishia bronni	Reishia				
SSEO1118-16	Reishia bronni	Reishia				
SSEO1508-16	Thais luteostoma	Thais				
SSEO1506-16	Thais luteostoma	Thais				
SSEO1503-16	Thais luteostoma	Thais				
SSEO1504-16	Thais luteostoma	Thais				
SSEO1501-16	Thais luteostoma	Thais				
SSEO1509-16	Thais luteostoma	Thais				
SSEO1507-16	Thais luteostoma	Thais				
SSEO1502-16	Thais luteostoma	Thais				
SSEO1505-16	Thais luteostoma	Thais				
QWEAS1521-15	Thais luteostoma	Thais				
QWEAS357-15	Thais luteostoma	Thais				
QWEAS356-15	Thais luteostoma	Thais				
QWEAS358-15	Thais luteostoma	Thais				
QWEAS359-15	Thais luteostoma	Thais				
QWEAS360-15	Thais luteostoma	Thais				
QWEAS361-15	Thais luteostoma	Thais				
SSEO548-16	Ruditapes philippinarum	Ruditapes	Genus	BOLD:AAA3922	274	Ruditapes[217], Venerupis[61]
SSEO556-16	Ruditapes philippinarum	Ruditapes				
SSEO554-16	Ruditapes philippinarum	Ruditapes				
SSEO1512-16	Ruditapes philippinarum	Ruditapes				
SSEO560-16	Ruditapes philippinarum	Ruditapes				
SSEO549-16	Ruditapes philippinarum	Ruditapes				
SSEO562-16	Ruditapes philippinarum	Ruditapes				
SSEO551-16	Ruditapes philippinarum	Ruditapes				
SSEO557-16	Ruditapes philippinarum	Ruditapes				
SSEO1511-16	Ruditapes philippinarum	Ruditapes				

SSEO552-16	Ruditapes philippinarum	Ruditapes				
SSEO561-16	Ruditapes philippinarum	Ruditapes				
SSEO550-16	Ruditapes philippinarum	Ruditapes				
SSEO558-16	Ruditapes philippinarum	Ruditapes				
SSEO553-16	Ruditapes philippinarum	Ruditapes				
QWEAS1454-15	Ruditapes philippinarum	Ruditapes				
QWEAS1455-15	Ruditapes philippinarum	Ruditapes				
QWEAS1182-15	Ruditapes philippinarum	Ruditapes				
QWEAS1184-15	Ruditapes philippinarum	Ruditapes				
QWEAS1185-15	Ruditapes philippinarum	Ruditapes				
QWEAS1245-15	Ruditapes philippinarum	Ruditapes				
QWEAS1246-15	Ruditapes philippinarum	Ruditapes				
QWEAS1179-15	Ruditapes philippinarum	Ruditapes				
QWEAS1247-15	Ruditapes philippinarum	Ruditapes				
QWEAS1243-15	Ruditapes philippinarum	Ruditapes				
QWEAS1181-15	Ruditapes philippinarum	Ruditapes				
QWEAS1244-15	Ruditapes philippinarum	Ruditapes				
QWEAS1183-15	Ruditapes philippinarum	Ruditapes				
QWEAS1186-15	Ruditapes philippinarum	Ruditapes				
QWEAS1180-15	Ruditapes philippinarum	Ruditapes				
QWEAS1250-15	Ruditapes variegata	Ruditapes				
QWEAS1187-15	Ruditapes variegata	Ruditapes				
QWEAS1248-15	Ruditapes variegata	Ruditapes				
QWEAS1251-15	Ruditapes variegata	Ruditapes				
QWEAS1189-15	Ruditapes variegata	Ruditapes				
QWEAS1188-15	Ruditapes variegata	Ruditapes	Genus	BOLD:AAH7873	26	Ruditapes[25], Venerupis[1]
QWEAS1191-15	Ruditapes variegata	Ruditapes				
QWEAS1249-15	Ruditapes variegata	Ruditapes				
QWEAS1192-15	Ruditapes variegata	Ruditapes				
QWEAS1190-15	Ruditapes variegata	Ruditapes				

SSEO750-16	Scapharca inaequivalvis	Scapharca	Genus	BOLD:AAR4114	4	Scapharca[2], Anadara[2]
SSEO740-16	Scapharca kagoshimensis	Scapharca	Genus	BOLD:AAI0662	94	Scapharca[66], Anadara[26]
SSEO743-16	Scapharca kagoshimensis	Scapharca				
SSEO742-16	Scapharca kagoshimensis	Scapharca				
SSEO746-16	Scapharca kagoshimensis	Scapharca				
SSEO739-16	Scapharca kagoshimensis	Scapharca				
SSEO719-16	Scapharca kagoshimensis	Scapharca				
SSEO741-16	Scapharca kagoshimensis	Scapharca				
SSEO747-16	Scapharca kagoshimensis	Scapharca				
SSEO745-16	Scapharca kagoshimensis	Scapharca				
SSEO720-16	Scapharca kagoshimensis	Scapharca				
SSEO738-16	Scapharca kagoshimensis	Scapharca				
SSEO721-16	Scapharca kagoshimensis	Scapharca				
SSEO1367-16	Scapharca kagoshimensis	Scapharca				
SSEO748-16	Scapharca kagoshimensis	Scapharca				
QWEAS171-15	Sepiella japonica	Sepiella	Genus	BOLD:AAD8673	47	Sepiella[37], Sepia[4]
QWEAS170-15	Sepiella japonica	Sepiella				
QWEAS169-15	Sepiella japonica	Sepiella				
SSEO058-16	Sepiella maindroni	Sepiella				
QWEAS1313-15	Soletellina diphos	Soletellina	Genus	BOLD:ACQ3738	3	Soletellina[2], Psammotaea[1]
QWEAS510-15	Strombus lentiginosus	Strombus	Genus	BOLD:ACB7576	6	Strombus[5], Canarium[1]
QWEAS509-15	Strombus lentiginosus	Strombus				
QWEAS508-15	Strombus mutabilis	Strombus				
SSEO038-15	Sunetta concinna	Sunetta	Genus	BOLD:AAO8013	8	Sunetta[4], Chamelea[2]
SSEO1260-16	Terebralia sulcata	Terebralia	Genus	BOLD:AAE4101	16	Terebralia[15], Cerithidea[1]
SSEO1255-16	Terebralia sulcata	Terebralia				
QWEAS347-15	Thais clavigera	Thais	Genus	BOLD:AAW6905	60	Reishia[44], Thais[13]
SSEO1499-16	Thais clavigera	Thais				
SSEO1496-16	Thais clavigera	Thais				
SSEO1500-16	Thais clavigera	Thais				

SSEO1497-16	Thais clavigera	Thais				
QWEAS351-15	Thais clavigera	Thais				
QWEAS348-15	Thais clavigera	Thais				
QWEAS346-15	Thais clavigera	Thais				
QWEAS349-15	Thais clavigera	Thais				
QWEAS350-15	Thais clavigera	Thais				
SSEO1498-16	Thais clavigera	Thais				
SSEO1510-16	Thais luteostoma	Thais				
QWEAS367-15	Thais mutabilis	Thais	Genus	BOLD:ACB7302	24	Thais[16], Indothais[8]
QWEAS364-15	Thais mutabilis	Thais				
QWEAS363-15	Thais mutabilis	Thais				
QWEAS366-15	Thais mutabilis	Thais				
QWEAS365-15	Thais mutabilis	Thais				
QWEAS362-15	Thais mutabilis	Thais				
QWEAS140-15	Uroteuthis sibogae	Uroteuthis	Genus	BOLD:AAD8238	18	Uroteuthis[10], Doryteuthis[8]
QWEAS141-15	Uroteuthis sibogae	Uroteuthis				
QWEAS142-15	Uroteuthis sibogae	Uroteuthis				
SSEO547-16	Venerupis bruguieri	Venerupis	Genus	BOLD:AAW7336	8	Ruditapes[6], Venerupis[2]
QWEAS076-15	Xenostrobus atratus	Xenostrobus	Genus	BOLD:ACQ2673	10	Xenostrobus[5], Limnoperna[5]
QWEAS075-15	Xenostrobus atratus	Xenostrobus				
QWEAS074-15	Xenostrobus atratus	Xenostrobus				
QWEAS073-15	Xenostrobus atratus	Xenostrobus				
QWEAS077-15	Xenostrobus atratus	Xenostrobus				
SSEO1379-16	Acanthochitona achates	Acanthochitona achates	Species	BOLD:ACB8074	16	Acanthochitona rubrolineata[8], Acanthochitona achates[4], Acanthochitona defilippi[3]
SSEO1380-16	Acanthochitona achates	Acanthochitona achates				
SSEO1386-16	Acanthochitona defilippi	Acanthochitona defilippi				
SSEO1391-16	Acanthochitona rubrolineata	Acanthochitona rubrolineata				
SSEO1392-16	Acanthochitona rubrolineata	Acanthochitona rubrolineata				

SSEO1394-16	Acanthochitona rubrolineata	Acanthochitona rubrolineata				
SSEO1393-16	Acanthochitona rubrolineata	Acanthochitona rubrolineata				
SSEO314-16	Architeuthis dux	Architeuthis dux	Species	BOLD:AAC3700	65	Architeuthis dux[60], Architeuthis sp. ARL-2008[1], Architeuthis sp.[1]
SSEO315-16	Architeuthis dux	Architeuthis dux				
SSEO296-16	Architeuthis dux	Architeuthis dux				
SSEO312-16	Architeuthis dux	Architeuthis dux				
SSEO305-16	Architeuthis dux	Architeuthis dux				
SSEO313-16	Architeuthis dux	Architeuthis dux				
QWEAS316-15	Babylonia lutosa	Babylonia lutosa	Species	BOLD:AAW3123	3	Babylonia lutosa[1], Babylonia formosae[1], Babylonia areolata[1]
SSEO440-16	Bathymodiolus septemdierum	Bathymodiolus septemdierum	Species	BOLD:AAI7746	8	Bathymodiolus brevior[8], Bathymodiolus septemdierum[6], Bathymodiolus marisindicus[2], Bathymodiolus aff. brevior[2]
SSEO442-16	Bathymodiolus septemdierum	Bathymodiolus septemdierum				
SSEO424-16	Bathymodiolus septemdierum	Bathymodiolus septemdierum				
SSEO1152-16	Batillaria cumingii	Batillaria cumingii	Species	BOLD:ACB7408	15	Batillaria attramentaria[13], Batillaria cumingii[2]
QWEAS060-15	Brachidontes mutabilis	Brachidontes mutabilis	Species	BOLD:AAD4589	12	Brachidontes sp. 3 MC-morph[6], Brachidontes sp. 2 ON-morph[4], Brachidontes mutabilis[2]
QWEAS229-15	Buccinum pemphigum	Buccinum pemphigum	Species	BOLD:AAW7286	8	Buccinum pemphigum[7], Buccinum yokomaruae[1]
QWEAS232-15	Buccinum pemphigum	Buccinum pemphigum				
QWEAS230-15	Buccinum pemphigum	Buccinum pemphigum				
QWEAS233-15	Buccinum pemphigum	Buccinum pemphigum				
QWEAS234-15	Buccinum pemphigum	Buccinum pemphigum				
QWEAS231-15	Buccinum pemphigum	Buccinum pemphigum				
SSEO886-16	Calliostoma aculeatum	Calliostoma aculeatum	Species	BOLD:AAW8762	4	Calliostoma sakashitai[2],

SSEO879-16	Calliostoma sakashitai	Calliostoma sakashitai				Calliostoma aculeatum[2]
SSEO884-16	Calliostoma consors	Calliostoma consors	Species	BOLD:AAW8761	4	Calliostoma consors[2], Calliostoma unicum[1]
SSEO680-16	Calyptogena kawamurai	Calyptogena kawamurai	Species	BOLD:AAC7877	11	Calyptogena solidissima[9], Calyptogena kawamurai[2]
SSEO774-16	Cantharidus bisbalteatus	Cantharidus bisbalteatus	Species	BOLD:ACB7697	4	Cantharidus jessoensis[2], Cantharidus bisbalteatus[2]
SSEO772-16	Cantharidus jessoensis	Cantharidus jessoensis				
SSEO990-16	Cellana grata	Cellana grata	Species	BOLD:AAW6225	10	Cellana grata[8], Cellana nigrolineata[2]
SSEO989-16	Cellana grata	Cellana grata				
SSEO991-16	Cellana grata	Cellana grata				
SSEO992-16	Cellana grata	Cellana grata				
SSEO915-16	Cellana nigrolineata	Cellana nigrolineata				
SSEO896-16	Cellana radiata	Cellana radiata	Species	BOLD:AAC0533	25	Cellana radiata[21], Cellana radiata enneagona[4]
SSEO902-16	Cellana radiata	Cellana radiata				
SSEO868-16	Cellana radiata	Cellana radiata				
SSEO898-16	Cellana radiata	Cellana radiata				
SSEO901-16	Cellana radiata	Cellana radiata				
SSEO903-16	Cellana radiata	Cellana radiata				
SSEO867-16	Cellana radiata	Cellana radiata				
SSEO900-16	Cellana radiata	Cellana radiata				
SSEO899-16	Cellana radiata	Cellana radiata				
SSEO894-16	Cellana radiata	Cellana radiata				
SSEO892-16	Cellana radiata enneagona	Cellana radiata enneagona				
SSEO893-16	Cellana radiata enneagona	Cellana radiata enneagona				
SSEO1238-16	Cerithidea rhizophorarum	Cerithidea rhizophorarum	Species	BOLD:AAA9510	100	Cerithidea rhizophorarum[98], Cerithidea cingulata[2]
SSEO1240-16	Cerithidea rhizophorarum	Cerithidea				

		rhizophorarum				
SSEO1219-16	Cerithidea rhizophorarum	Cerithidea rhizophorarum				
SSEO1241-16	Cerithidea rhizophorarum	Cerithidea rhizophorarum				
SSEO1190-16	Cerithidea rhizophorarum	Cerithidea rhizophorarum				
SSEO1189-16	Cerithidea rhizophorarum	Cerithidea rhizophorarum				
SSEO1375-16	Cerithidea rhizophorarum	Cerithidea rhizophorarum				
SSEO1242-16	Cerithidea rhizophorarum	Cerithidea rhizophorarum				
SSEO1231-16	Cerithidea rhizophorarum	Cerithidea rhizophorarum				
SSEO1227-16	Cerithidea rhizophorarum	Cerithidea rhizophorarum				
SSEO1244-16	Cerithidea rhizophorarum	Cerithidea rhizophorarum				
SSEO1225-16	Cerithidea rhizophorarum	Cerithidea rhizophorarum				
SSEO1220-16	Cerithidea rhizophorarum	Cerithidea rhizophorarum				
SSEO1230-16	Cerithidea rhizophorarum	Cerithidea rhizophorarum				
SSEO1232-16	Cerithidea rhizophorarum	Cerithidea rhizophorarum				
SSEO1218-16	Cerithidea rhizophorarum	Cerithidea rhizophorarum				
SSEO1234-16	Cerithidea rhizophorarum	Cerithidea				

		rhizophorarum				
SSEO1228-16	Cerithidea rhizophorarum	Cerithidea rhizophorarum				
SSEO1223-16	Cerithidea rhizophorarum	Cerithidea rhizophorarum				
SSEO1235-16	Cerithidea rhizophorarum	Cerithidea rhizophorarum				
SSEO1236-16	Cerithidea rhizophorarum	Cerithidea rhizophorarum				
SSEO1215-16	Cerithidea rhizophorarum	Cerithidea rhizophorarum				
QWEAS1584-15	Cerithidea rhizophorarum	Cerithidea rhizophorarum				
QWEAS1585-15	Cerithidea rhizophorarum	Cerithidea rhizophorarum				
SSEO1226-16	Cerithidea rhizophorarum	Cerithidea rhizophorarum				
SSEO1221-16	Cerithidea rhizophorarum	Cerithidea rhizophorarum				
SSEO1237-16	Cerithidea rhizophorarum	Cerithidea rhizophorarum				
SSEO1376-16	Cerithidea rhizophorarum	Cerithidea rhizophorarum				
SSEO1224-16	Cerithidea rhizophorarum	Cerithidea rhizophorarum				
SSEO1229-16	Cerithidea rhizophorarum	Cerithidea rhizophorarum				
SSEO1239-16	Cerithidea rhizophorarum	Cerithidea rhizophorarum				
SSEO1222-16	Cerithidea rhizophorarum	Cerithidea				

		rhizophorarum				
SSEO1233-16	Cerithidea rhizophorarum	Cerithidea rhizophorarum				
SSEO1216-16	Cerithidea rhizophorarum	Cerithidea rhizophorarum				
QWEAS383-15	Chicoreus torrefactus	Chicoreus torrefactus	Species	BOLD:AAJ0544	22	Chicoreus torrefactus[10], Chicoreus sp.[1], Chicoreus aculeatus[1]
QWEAS380-15	Chicoreus torrefactus	Chicoreus torrefactus				
QWEAS382-15	Chicoreus torrefactus	Chicoreus torrefactus				
QWEAS381-15	Chicoreus torrefactus	Chicoreus torrefactus				
QWEAS1344-15	Cistopus indicus	Cistopus indicus	Species	BOLD:ABA3763	13	Cistopus indicus[5], Cistopus taiwanicus[4], Cistopus cf. indicus NK-2008[1]
QWEAS211-15	Cistopus taiwanicus	Cistopus taiwanicus				
QWEAS210-15	Cistopus taiwanicus	Cistopus taiwanicus				
SSEO1049-16	Conus lividus	Conus lividus	Species	BOLD:AAO6206	79	Conus lividus[78], Conus sanguinolentus[1]
SSEO1047-16	Conus lividus	Conus lividus				
SSEO1048-16	Conus lividus	Conus lividus				
QWEAS326-15	Conus sanguinolentus	Conus sanguinolentus				
SSEO1041-16	Conus sanguinolentus	Conus sanguinolentus	Species	BOLD:ACB8444	119	Conus sanguinolentus[118], Conus lividus[1]
SSEO1077-16	Conus sanguinolentus	Conus sanguinolentus				
SSEO1080-16	Conus sanguinolentus	Conus sanguinolentus				
SSEO1044-16	Conus sanguinolentus	Conus sanguinolentus				
SSEO1040-16	Conus sanguinolentus	Conus sanguinolentus				
SSEO1079-16	Conus sanguinolentus	Conus sanguinolentus				
SSEO1061-16	Conus sanguinolentus	Conus sanguinolentus				
SSEO1046-16	Conus sanguinolentus	Conus sanguinolentus				
SSEO1053-16	Conus sanguinolentus	Conus sanguinolentus				
SSEO1076-16	Conus sanguinolentus	Conus sanguinolentus				
SSEO1045-16	Conus sanguinolentus	Conus sanguinolentus				
SSEO1078-16	Conus sanguinolentus	Conus sanguinolentus				
SSEO1042-16	Conus sanguinolentus	Conus sanguinolentus				
SSEO1043-16	Conus sanguinolentus	Conus sanguinolentus				

SSEO597-16	Corbicula fluminea	Corbicula fluminea	Species	BOLD:ACF5867	86	Corbicula leana[29], Corbicula fluminea[15], Corbicula sp.[13], Corbicula africana[4], Corbicula North American form B[3], Corbicula fluminalis[3], Corbicula sp. China/CN-F[1], Corbicula sp. China/CN-B[1], Corbicula sp. China/CN-G[1], Corbicula sp. China/CN-C[1], Corbicula sp. LMP-2010[1], Corbicula sp. PKa-2002[1], Corbicula sp. 57Rho2[1], Corbicula sp. PKb-2002[1], Corbicula sp. China/CN-H[1], Corbicula sp. 34IL4[1], Corbicula sp. 31IL1[1], Corbicula North American form C[1], Corbicula javanica[1], Corbicula sp. 32IL2[1], Corbicula sp. 33IL3[1], Corbicula sp. 44C_ea[1], Corbicula sp. 35IL11[1], Corbicula sp. 56Rho1[1]
SSEO588-16	Corbicula fluminea	Corbicula fluminea				
SSEO595-16	Corbicula fluminea	Corbicula fluminea				
SSEO592-16	Corbicula fluminea	Corbicula fluminea				
SSEO590-16	Corbicula fluminea	Corbicula fluminea				
SSEO536-16	Corbicula fluminea	Corbicula fluminea				
SSEO668-16	Corbicula leana	Corbicula leana				
SSEO661-16	Corbicula leana	Corbicula leana				
SSEO666-16	Corbicula leana	Corbicula leana				
SSEO662-16	Corbicula leana	Corbicula leana				
SSEO667-16	Corbicula leana	Corbicula leana				
SSEO601-16	Corbicula japonica	Corbicula japonica	Species	BOLD:AAC3963	219	Corbicula japonica[199], Corbicula fluminalis[3], Corbicula sp. Kor2[1], Corbicula sp. Kor3[1], Corbicula sp. Kor4[1], Corbicula sp. T01[1], Corbicula sp. Kor1[1], Corbicula sp. Jpn5[1], Corbicula fluminea[1], Corbicula sp.[1], Corbicula sp. Jpn1[1], Corbicula sp. Jpn3[1], Corbicula sp. Jpn6[1]
SSEO637-16	Corbicula japonica	Corbicula japonica				
SSEO623-16	Corbicula japonica	Corbicula japonica				
SSEO627-16	Corbicula japonica	Corbicula japonica				
SSEO643-16	Corbicula japonica	Corbicula japonica				
SSEO652-16	Corbicula japonica	Corbicula japonica				
SSEO635-16	Corbicula japonica	Corbicula japonica				
SSEO631-16	Corbicula japonica	Corbicula japonica				
SSEO638-16	Corbicula japonica	Corbicula japonica				

SSEO647-16	Corbicula japonica	Corbicula japonica				
SSEO657-16	Corbicula japonica	Corbicula japonica				
SSEO654-16	Corbicula japonica	Corbicula japonica				
SSEO639-16	Corbicula japonica	Corbicula japonica				
SSEO625-16	Corbicula japonica	Corbicula japonica				
SSEO655-16	Corbicula japonica	Corbicula japonica				
SSEO603-16	Corbicula japonica	Corbicula japonica				
SSEO628-16	Corbicula japonica	Corbicula japonica				
SSEO604-16	Corbicula japonica	Corbicula japonica				
SSEO644-16	Corbicula japonica	Corbicula japonica				
SSEO651-16	Corbicula japonica	Corbicula japonica				
SSEO670-16	Corbicula japonica	Corbicula japonica				
SSEO641-16	Corbicula japonica	Corbicula japonica				
SSEO649-16	Corbicula japonica	Corbicula japonica				
SSEO626-16	Corbicula japonica	Corbicula japonica				
SSEO653-16	Corbicula japonica	Corbicula japonica				
SSEO599-16	Corbicula japonica	Corbicula japonica				
SSEO636-16	Corbicula japonica	Corbicula japonica				
SSEO646-16	Corbicula japonica	Corbicula japonica				
SSEO656-16	Corbicula japonica	Corbicula japonica				
SSEO629-16	Corbicula japonica	Corbicula japonica				
SSEO634-16	Corbicula japonica	Corbicula japonica				
SSEO630-16	Corbicula japonica	Corbicula japonica				
SSEO671-16	Corbicula japonica	Corbicula japonica				
SSEO606-16	Corbicula japonica	Corbicula japonica				
SSEO650-16	Corbicula japonica	Corbicula japonica				
SSEO642-16	Corbicula japonica	Corbicula japonica				
SSEO1366-16	Corbicula japonica	Corbicula japonica				
SSEO640-16	Corbicula japonica	Corbicula japonica				
SSEO632-16	Corbicula japonica	Corbicula japonica				

SSEO669-16	Corbicula japonica	Corbicula japonica				
SSEO645-16	Corbicula japonica	Corbicula japonica				
SSEO633-16	Corbicula japonica	Corbicula japonica				
SSEO663-16	Corbicula leana	Corbicula leana	Species	BOLD:AAC2296	543	Corbicula fluminea[25], Corbicula North American form A[13], Corbicula leana[6], Corbicula largillierti[2], Corbicula sp. 37Virg2[1], Corbicula sp. 36Virg1[1], Corbicula sp. 38Virg3[1], Corbicula sp. 29HK2[1], Corbicula sp. 53WC1[1], Corbicula sp. 28HK1[1], Corbicula sp. 30HK3[1], Corbicula sp. 58Sao1[1], Corbicula sp. LMP-2010[1], Corbicula sp. Taiwan[1], Corbicula sp. 60Sao4[1], Corbicula sp. 59Sao2[1], Corbicula sp. 55WC5[1], Corbicula sp. 27Wi9[1], Corbicula sp. 54WC3[1], Corbicula sp. 24Le3[1], Corbicula sp. 14B4[1], Corbicula sp. 16506_10[1], Corbicula sp. 13B20[1], Corbicula sp. 12B19[1], Corbicula javanica[1], Corbicula sp. 02A15[1], Corbicula sp. 19560_34[1], Corbicula sp. 20De6[1], Corbicula cf. fluminea[1], Corbicula sp. 25Wi10[1], Corbicula sp. 23Le1[1],
SSEO665-16	Corbicula leana	Corbicula leana				
SSEO664-16	Corbicula leana	Corbicula leana				

						Corbicula sp. 22Ko4[1], Corbicula sp. 21De7[1], Corbicula sp. 26Wi3[1]
QWEAS020-15	Crassostrea ariakensis	Crassostrea ariakensis	Species	BOLD:AAD6870	38	Crassostrea ariakensis[34], Crassostrea sp. RE-2008[2]
QWEAS019-15	Crassostrea ariakensis	Crassostrea ariakensis				
QWEAS1535-15	Crassostrea ariakensis	Crassostrea ariakensis				
QWEAS1533-15	Crassostrea hongkongensis	Crassostrea hongkongensis	Species	BOLD:AAE3959	35	Crassostrea hongkongensis[23], Crassostrea sp. WE-2008[3], Crassostrea sp. F-KL-2003[1], Crassostrea sp. SL-2003[1], Crassostrea sp. E-KL-2003[1], Crassostrea sp. D-KL-2003[1], Crassostrea sp. A-KL-2003[1], Crassostrea sp. B-KL-2003[1], Crassostrea sp. C-KL-2003[1]
QWEAS1531-15	Crassostrea hongkongensis	Crassostrea hongkongensis				
QWEAS1532-15	Crassostrea hongkongensis	Crassostrea hongkongensis				
QWEAS1534-15	Crassostrea hongkongensis	Crassostrea hongkongensis				
QWEAS022-15	Crassostrea iredalei	Crassostrea iredalei	Species	BOLD:AAB5109	119	Crassostrea iredalei[87], Crassostrea madrasensis[29], Crassostrea sp. KL-2003[1]
QWEAS021-15	Crassostrea iredalei	Crassostrea iredalei				
SSEO712-16	Crassostrea sikamea	Crassostrea sikamea	Species	BOLD:AAI9053	73	Crassostrea sikamea[69], Crassostrea sp. HL-2008[2]
SSEO716-16	Crassostrea sikamea	Crassostrea sikamea				
SSEO709-16	Crassostrea sikamea	Crassostrea sikamea				
SSEO706-16	Crassostrea sikamea	Crassostrea sikamea				
SSEO711-16	Crassostrea sikamea	Crassostrea sikamea				
SSEO702-16	Crassostrea sikamea	Crassostrea sikamea				
SSEO705-16	Crassostrea sikamea	Crassostrea sikamea				
SSEO703-16	Crassostrea sikamea	Crassostrea sikamea				
SSEO717-16	Crassostrea sikamea	Crassostrea sikamea				
SSEO708-16	Crassostrea sikamea	Crassostrea sikamea				
SSEO718-16	Crassostrea sikamea	Crassostrea sikamea				
SSEO707-16	Crassostrea sikamea	Crassostrea sikamea				

SSEO704-16	Crassostrea sikamea	Crassostrea sikamea				
SSEO733-16	Crassostrea sikamea	Crassostrea sikamea				
SSEO710-16	Crassostrea sikamea	Crassostrea sikamea				
QWEAS014-15	Crassostrea sikamea	Crassostrea sikamea				
QWEAS009-15	Crassostrea sikamea	Crassostrea sikamea				
QWEAS017-15	Crassostrea sikamea	Crassostrea sikamea				
QWEAS013-15	Crassostrea sikamea	Crassostrea sikamea				
QWEAS010-15	Crassostrea sikamea	Crassostrea sikamea				
QWEAS015-15	Crassostrea sikamea	Crassostrea sikamea				
QWEAS011-15	Crassostrea sikamea	Crassostrea sikamea				
QWEAS016-15	Crassostrea sikamea	Crassostrea sikamea				
QWEAS012-15	Crassostrea sikamea	Crassostrea sikamea				
QWEAS018-15	Crassostrea sikamea	Crassostrea sikamea				
SSEO732-16	Crassostrea sikamea	Crassostrea sikamea				
SSEO073-16	Cuvierina pacifica	Cuvierina pacifica	Species	BOLD:ACH8721	53	Cuvierina pacifica[48], Cuvierina columnella[3], Cuvierina urceolaris[2]
SSEO075-16	Cuvierina pacifica	Cuvierina pacifica				
SSEO076-16	Cuvierina pacifica	Cuvierina pacifica				
SSEO072-16	Cuvierina pacifica	Cuvierina pacifica				
SSEO074-16	Cuvierina pacifica	Cuvierina pacifica				
QWEAS517-15	Cypraea tigris	Cypraea tigris	Species	BOLD:AAD6059	9	Cypraea tigris[8], Cypraea sp.[1]
QWEAS518-15	Cypraea tigris	Cypraea tigris				
QWEAS1492-15	Dosinia biscocta	Dosinia biscocta	Species	BOLD:AAO9163	19	Dosinia fibula[10], Dosinia biscocta[4]
QWEAS1491-15	Dosinia biscocta	Dosinia biscocta				
QWEAS1007-15	Dosinia fibula	Dosinia fibula				
QWEAS1010-15	Dosinia fibula	Dosinia fibula				
QWEAS1009-15	Dosinia fibula	Dosinia fibula				
QWEAS1008-15	Dosinia fibula	Dosinia fibula				
QWEAS1011-15	Dosinia fibula	Dosinia fibula				
SSEO1100-16	Drupa albolabris	Drupa albolabris	Species	BOLD:ACB7183	14	Drupa albolabris[12], Drupa ricinus[1]
SSEO1101-16	Drupa albolabris	Drupa albolabris				

SSEO1006-16	Echinolittorina reticulata	Echinolittorina reticulata	Species	BOLD:AAA9391	61	Echinolittorina reticulata[49], Echinolittorina millegrana[12]
SSEO1003-16	Echinolittorina reticulata	Echinolittorina reticulata				
SSEO1001-16	Echinolittorina reticulata	Echinolittorina reticulata				
SSEO1009-16	Echinolittorina reticulata	Echinolittorina reticulata				
SSEO1038-16	Echinolittorina reticulata	Echinolittorina reticulata				
SSEO1004-16	Echinolittorina reticulata	Echinolittorina reticulata				
SSEO1002-16	Echinolittorina reticulata	Echinolittorina reticulata				
SSEO1007-16	Echinolittorina reticulata	Echinolittorina reticulata				
SSEO1005-16	Echinolittorina reticulata	Echinolittorina reticulata				
SSEO1008-16	Echinolittorina reticulata	Echinolittorina reticulata				
SSEO1000-16	Echinolittorina reticulata	Echinolittorina reticulata				
SSEO1039-16	Echinolittorina reticulata	Echinolittorina reticulata				
QWEAS1600-15	Echinolittorina vidua	Echinolittorina vidua	Species	BOLD:AAA4229	29	Echinolittorina vidua[27], Echinolittorina vidua B[2]
QWEAS1596-15	Echinolittorina vidua	Echinolittorina vidua				
QWEAS1599-15	Echinolittorina vidua	Echinolittorina vidua				
QWEAS1597-15	Echinolittorina vidua	Echinolittorina vidua				
QWEAS1601-15	Echinolittorina vidua	Echinolittorina vidua				
QWEAS1598-15	Echinolittorina vidua	Echinolittorina vidua				
SSEO170-16	Elysia abei	Elysia abei	Species	BOLD:ACI2275	16	Elysia abei[14], Elysia amakusana[2]
SSEO167-16	Elysia abei	Elysia abei				
SSEO171-16	Elysia abei	Elysia abei				
SSEO168-16	Elysia abei	Elysia abei				
SSEO166-16	Elysia abei	Elysia abei				
SSEO169-16	Elysia abei	Elysia abei				
SSEO174-16	Elysia amakusana	Elysia amakusana				
SSEO210-16	Elysia atroviridis	Elysia atroviridis	Species	BOLD:ACI2277	95	Elysia atroviridis[92], Elysia setoensis[2], Elysia cf. flavomacula[1]
SSEO214-16	Elysia atroviridis	Elysia atroviridis				
SSEO260-16	Elysia atroviridis	Elysia atroviridis				
SSEO276-16	Elysia atroviridis	Elysia atroviridis				
SSEO268-16	Elysia atroviridis	Elysia atroviridis				

SSEO237-16	Elysia atroviridis	Elysia atroviridis				
SSEO256-16	Elysia atroviridis	Elysia atroviridis				
SSEO233-16	Elysia atroviridis	Elysia atroviridis				
SSEO219-16	Elysia atroviridis	Elysia atroviridis				
SSEO262-16	Elysia atroviridis	Elysia atroviridis				
SSEO209-16	Elysia atroviridis	Elysia atroviridis				
SSEO273-16	Elysia atroviridis	Elysia atroviridis				
SSEO267-16	Elysia atroviridis	Elysia atroviridis				
SSEO231-16	Elysia atroviridis	Elysia atroviridis				
SSEO235-16	Elysia atroviridis	Elysia atroviridis				
SSEO217-16	Elysia atroviridis	Elysia atroviridis				
SSEO271-16	Elysia atroviridis	Elysia atroviridis				
SSEO281-16	Elysia atroviridis	Elysia atroviridis				
SSEO258-16	Elysia atroviridis	Elysia atroviridis				
SSEO251-16	Elysia atroviridis	Elysia atroviridis				
SSEO278-16	Elysia atroviridis	Elysia atroviridis				
SSEO245-16	Elysia atroviridis	Elysia atroviridis				
SSEO218-16	Elysia atroviridis	Elysia atroviridis				
SSEO223-16	Elysia atroviridis	Elysia atroviridis				
SSEO206-16	Elysia atroviridis	Elysia atroviridis				
SSEO227-16	Elysia atroviridis	Elysia atroviridis				
SSEO253-16	Elysia atroviridis	Elysia atroviridis				
SSEO247-16	Elysia atroviridis	Elysia atroviridis				
SSEO204-16	Elysia atroviridis	Elysia atroviridis				
SSEO229-16	Elysia atroviridis	Elysia atroviridis				
SSEO208-16	Elysia atroviridis	Elysia atroviridis				
SSEO266-16	Elysia atroviridis	Elysia atroviridis				
SSEO243-16	Elysia atroviridis	Elysia atroviridis				
SSEO225-16	Elysia atroviridis	Elysia atroviridis				
SSEO280-16	Elysia atroviridis	Elysia atroviridis				

SSEO221-16	Elysia atroviridis	Elysia atroviridis				
SSEO277-16	Elysia atroviridis	Elysia atroviridis				
SSEO242-16	Elysia atroviridis	Elysia atroviridis				
SSEO246-16	Elysia atroviridis	Elysia atroviridis				
SSEO230-16	Elysia atroviridis	Elysia atroviridis				
SSEO232-16	Elysia atroviridis	Elysia atroviridis				
SSEO279-16	Elysia atroviridis	Elysia atroviridis				
SSEO244-16	Elysia atroviridis	Elysia atroviridis				
SSEO240-16	Elysia atroviridis	Elysia atroviridis				
SSEO249-16	Elysia atroviridis	Elysia atroviridis				
SSEO263-16	Elysia atroviridis	Elysia atroviridis				
SSEO172-16	Elysia setoensis	Elysia setoensis				
SSEO180-16	Elysia atroviridis	Elysia atroviridis	Species	BOLD:AAM5939	20	Elysia cf. marginata 3 JV2013[12], Elysia ornata[7], Elysia atroviridis[1]
SSEO183-16	Elysia ornata	Elysia ornata				
SSEO181-16	Elysia ornata	Elysia ornata				
SSEO184-16	Elysia ornata	Elysia ornata	Species	BOLD:ACI0075	6	Elysia ornata[4], Elysia cf. marginata[2]
SSEO199-16	Elysia ornata	Elysia ornata				
SSEO182-16	Elysia ornata	Elysia ornata	Species	BOLD:ACI0076	6	Elysia cf. marginata[4], Elysia ornata[2]
QWEAS516-15	Erronea errones	Erronea errones	Species	BOLD:AAB7225	7	Erronea caurica[5], Erronea errones[2]
QWEAS181-15	Euprymna berryi	Euprymna berryi	Species	BOLD:AAC6030	15	Euprymna berryi[8], Euprymna hyllebergi[4]
QWEAS182-15	Euprymna berryi	Euprymna berryi				
QWEAS183-15	Euprymna berryi	Euprymna berryi				
QWEAS180-15	Euprymna berryi	Euprymna berryi				
QWEAS186-15	Euprymna morsei	Euprymna morsei	Species	BOLD:AAJ0427	10	Euprymna morsei[6], Euprymna berryi[1]
QWEAS184-15	Euprymna morsei	Euprymna morsei				
QWEAS185-15	Euprymna morsei	Euprymna morsei				
QWEAS550-15	Ficus variegata	Ficus variegata	Species	BOLD:ACB8356	6	Ficus variegata Roding 1798[3], Ficus variegata[3]
QWEAS548-15	Ficus variegata	Ficus variegata				

QWEAS549-15	Ficus variegata	Ficus variegata				
SSEO1423-16	Fusinus forceps	Fusinus forceps	Species	BOLD:ACB7195	12	Fusinus forceps[10], Fusinus longicaudus[2]
SSEO1425-16	Fusinus forceps	Fusinus forceps				
SSEO1426-16	Fusinus forceps	Fusinus forceps				
SSEO1424-16	Fusinus forceps	Fusinus forceps				
SSEO1422-16	Fusinus forceps	Fusinus forceps				
SSEO1427-16	Fusinus longicaudus	Fusinus longicaudus				
SSEO679-16	Geloina erosa	Geloina erosa	Species	BOLD:AAY1140	3	Geloina erosa[2], Geloina expansa[1]
SSEO309-16	Gonatus berryi	Gonatus berryi	Species	BOLD:ACH3990	4	Gonatus californiensis[2], Gonatus berryi[2]
SSEO307-16	Gonatus madokai	Gonatus madokai	Species	BOLD:AAE3426	11	Gonatus madokai[9], Gonatus kamtschaticus[2]
SSEO311-16	Gonatus madokai	Gonatus madokai				
SSEO310-16	Gonatus madokai	Gonatus madokai				
SSEO325-16	Gonatus pyros	Gonatus pyros	Species	BOLD:AAE3427	7	Gonatus pyros[5], Gonatus kamtschaticus[2]
SSEO302-16	Idiosepius biserialis	Idiosepius biserialis	Species	BOLD:AAW9588	41	Idiosepius paradoxus[35], Idiosepius biserialis[6]
SSEO300-16	Idiosepius biserialis	Idiosepius biserialis				
SSEO301-16	Idiosepius biserialis	Idiosepius biserialis				
SSEO299-16	Idiosepius paradoxus	Idiosepius paradoxus				
SSEO257-16	Idiosepius paradoxus	Idiosepius paradoxus				
SSEO282-16	Idiosepius paradoxus	Idiosepius paradoxus				
SSEO272-16	Idiosepius paradoxus	Idiosepius paradoxus				
SSEO287-16	Idiosepius paradoxus	Idiosepius paradoxus				
SSEO298-16	Idiosepius paradoxus	Idiosepius paradoxus				
SSEO269-16	Idiosepius paradoxus	Idiosepius paradoxus				
SSEO270-16	Idiosepius paradoxus	Idiosepius paradoxus				
SSEO275-16	Idiosepius paradoxus	Idiosepius paradoxus				
SSEO295-16	Idiosepius paradoxus	Idiosepius paradoxus				
SSEO297-16	Idiosepius paradoxus	Idiosepius paradoxus				

SSEO274-16	Idiosepius paradoxus	Idiosepius paradoxus				
SSEO286-16	Idiosepius paradoxus	Idiosepius paradoxus				
SSEO284-16	Idiosepius paradoxus	Idiosepius paradoxus				
SSEO291-16	Idiosepius paradoxus	Idiosepius paradoxus				
SSEO294-16	Idiosepius paradoxus	Idiosepius paradoxus				
SSEO293-16	Idiosepius paradoxus	Idiosepius paradoxus				
SSEO783-16	Isognomon acutirostris	Isognomon acutirostris	Species	BOLD:AAW9229	3	Isognomon acutirostris[2], Isognomon nucleus[1]
QWEAS875-15	Isognomon nucleus	Isognomon nucleus				
SSEO784-16	Isognomon perna	Isognomon perna	Species	BOLD:AAW9227	6	Isognomon perna[4], Isognomon sp.[2]
QWEAS866-15	Isognomon perna	Isognomon perna				
QWEAS867-15	Isognomon perna	Isognomon perna				
SSEO843-16	Lepeta caeca pacifica	Lepeta caeca pacifica	Species	BOLD:AAX5488	4	Lepeta caeca pacifica[3], Lepeta caeca[1]
SSEO842-16	Lepeta caeca pacifica	Lepeta caeca pacifica				
SSEO1014-16	Littoraria coccinea	Littoraria coccinea	Species	BOLD:AAU1059	19	Littoraria coccinea glabrata[8], Littoraria glabrata[7], Littoraria coccinea[3]
QWEAS524-15	Littoraria intermedia	Littoraria intermedia	Species	BOLD:ACB7473	5	Littoraria intermedia[4], Littoraria articulata[1]
QWEAS525-15	Littoraria intermedia	Littoraria intermedia				
SSEO981-16	Littoraria pintado	Littoraria pintado	Species	BOLD:AAX6439	8	Littoraria pintado[3], Littoraria pintado pintado[3], Littoraria pintado pullata[2]
QWEAS557-15	Littoraria scabra	Littoraria scabra	Species	BOLD:ACB7955	4	Littoraria scabra[2], Littoraria ardouiniana[2]
QWEAS125-15	Loliolus japonicus	Loliolus japonicus	Species	BOLD:AAX6339	20	Loliolus japonicus[19], Loliolus beka[1]
QWEAS128-15	Loliolus japonicus	Loliolus japonicus				
QWEAS126-15	Loliolus japonicus	Loliolus japonicus				
QWEAS129-15	Loliolus japonicus	Loliolus japonicus				
QWEAS130-15	Loliolus japonicus	Loliolus japonicus				
QWEAS127-15	Loliolus japonicus	Loliolus japonicus				
SSEO1307-16	Lunella cinerea	Lunella cinerea	Species	BOLD:AAD3795	45	Lunella sp. ciwp[14], Lunella

SSEO1308-16	Lunella cinerea	Lunella cinerea				cinerea[11]
SSEO1309-16	Lunella cinerea	Lunella cinerea				
SSEO1313-16	Lunella coronata	Lunella coronata				
SSEO1311-16	Lunella coronata	Lunella coronata				
SSEO1310-16	Lunella coronata	Lunella coronata				
SSEO1270-16	Lunella granulata	Lunella granulata	Species	BOLD:AAD3503	141	Lunella granulata[134], Lunella coronata[7]
SSEO1274-16	Lunella granulata	Lunella granulata				
SSEO1271-16	Lunella granulata	Lunella granulata				
QWEAS1602-15	Lunella granulata	Lunella granulata				
QWEAS1142-15	Macridiscus aequilatera	Macridiscus aequilatera				
QWEAS1143-15	Macridiscus aequilatera	Macridiscus aequilatera				
QWEAS1147-15	Macridiscus aequilatera	Macridiscus aequilatera				
QWEAS1144-15	Macridiscus aequilatera	Macridiscus aequilatera				
QWEAS1141-15	Macridiscus aequilatera	Macridiscus aequilatera				
QWEAS1146-15	Macridiscus aequilatera	Macridiscus aequilatera				
QWEAS1145-15	Macridiscus aequilatera	Macridiscus aequilatera				
QWEAS1415-15	Macridiscus multifarius	Macridiscus multifarius	Species	BOLD:AAO8015	43	Macridiscus multifarius[22], Macridiscus aequilatera[7]
QWEAS1419-15	Macridiscus multifarius	Macridiscus multifarius				
QWEAS1414-15	Macridiscus multifarius	Macridiscus multifarius				
QWEAS1420-15	Macridiscus multifarius	Macridiscus multifarius				
QWEAS1417-15	Macridiscus multifarius	Macridiscus multifarius				
QWEAS1416-15	Macridiscus multifarius	Macridiscus multifarius				
QWEAS1418-15	Macridiscus multifarius	Macridiscus multifarius				
QWEAS1421-15	Macridiscus multifarius	Macridiscus multifarius				
QWEAS1403-15	Meretrix lusoria	Meretrix lusoria				
QWEAS1406-15	Meretrix lusoria	Meretrix lusoria				
QWEAS1404-15	Meretrix lusoria	Meretrix lusoria	Species	BOLD:AAC6198	74	Meretrix petechialis[49], Meretrix lusoria[12], Meretrix meretrix[8], Meretrix sp. Taiwanese[2]
QWEAS1446-15	Meretrix meretrix	Meretrix meretrix				
QWEAS1413-15	Meretrix petechialis	Meretrix petechialis				
QWEAS1411-15	Meretrix petechialis	Meretrix petechialis				

QWEAS1051-15	Meretrix petechialis	Meretrix petechialis				
QWEAS1057-15	Meretrix petechialis	Meretrix petechialis				
QWEAS1062-15	Meretrix petechialis	Meretrix petechialis				
QWEAS1055-15	Meretrix petechialis	Meretrix petechialis				
QWEAS1059-15	Meretrix petechialis	Meretrix petechialis				
QWEAS1060-15	Meretrix petechialis	Meretrix petechialis				
QWEAS1049-15	Meretrix petechialis	Meretrix petechialis				
QWEAS1048-15	Meretrix petechialis	Meretrix petechialis				
QWEAS1061-15	Meretrix petechialis	Meretrix petechialis				
QWEAS1054-15	Meretrix petechialis	Meretrix petechialis				
QWEAS1058-15	Meretrix petechialis	Meretrix petechialis				
QWEAS1050-15	Meretrix petechialis	Meretrix petechialis				
QWEAS1053-15	Meretrix petechialis	Meretrix petechialis				
QWEAS1052-15	Meretrix petechialis	Meretrix petechialis				
QWEAS1056-15	Meretrix petechialis	Meretrix petechialis				
QWEAS1407-15	Meretrix lyrata	Meretrix lyrata	Species	BOLD:AAH7529	45	Meretrix lyrata[42], Meretrix lamarckii[1]
QWEAS1409-15	Meretrix lyrata	Meretrix lyrata				
QWEAS1068-15	Meretrix lyrata	Meretrix lyrata				
QWEAS1070-15	Meretrix lyrata	Meretrix lyrata				
QWEAS1072-15	Meretrix lyrata	Meretrix lyrata				
QWEAS1069-15	Meretrix lyrata	Meretrix lyrata				
QWEAS1071-15	Meretrix lyrata	Meretrix lyrata				
QWEAS1073-15	Meretrix lyrata	Meretrix lyrata				
QWEAS1067-15	Meretrix lyrata	Meretrix lyrata				
QWEAS1408-15	Meretrix lyrata	Meretrix lyrata				
QWEAS1445-15	Meretrix meretrix	Meretrix meretrix	Species	BOLD:AAO5535	26	Meretrix meretrix[25], Meretrix sp. YD-2011[1]
QWEAS1443-15	Meretrix meretrix	Meretrix meretrix				
QWEAS1036-15	Meretrix meretrix	Meretrix meretrix				
QWEAS1038-15	Meretrix meretrix	Meretrix meretrix				
QWEAS1035-15	Meretrix meretrix	Meretrix meretrix				

QWEAS1034-15	Meretrix meretrix	Meretrix meretrix				
QWEAS1037-15	Meretrix meretrix	Meretrix meretrix				
SSEO1448-16	Mitrella bicincta	Mitrella bicincta	Species	BOLD:ACB6970	18	Mitrella bicincta[16], Mitrella cf. tuberosa[2]
SSEO1441-16	Mitrella bicincta	Mitrella bicincta				
SSEO1444-16	Mitrella bicincta	Mitrella bicincta				
SSEO1449-16	Mitrella bicincta	Mitrella bicincta				
SSEO1442-16	Mitrella bicincta	Mitrella bicincta				
SSEO1447-16	Mitrella bicincta	Mitrella bicincta				
SSEO1445-16	Mitrella bicincta	Mitrella bicincta				
SSEO1440-16	Mitrella bicincta	Mitrella bicincta				
QWEAS264-15	Mitrella cf. tuberosa	Mitrella cf. tuberosa				
QWEAS265-15	Mitrella cf. tuberosa	Mitrella cf. tuberosa				
QWEAS065-15	Modiolus comptus	Modiolus comptus	Species	BOLD:AAX4596	10	Modiolus comptus[8], Modiolus nipponicus[2]
QWEAS064-15	Modiolus comptus	Modiolus comptus				
QWEAS063-15	Modiolus comptus	Modiolus comptus				
QWEAS066-15	Modiolus comptus	Modiolus comptus				
SSEO461-16	Modiolus nipponicus	Modiolus nipponicus				
SSEO825-16	Monodonta perplexa	Monodonta perplexa	Species	BOLD:AAX4757	5	Monodonta neritoides[3], Monodonta perplexa[2]
QWEAS288-15	Morula granulata	Morula granulata	Species	BOLD:ACH7783	6	Morula granulata[5], Morula ceylonica[1]
QWEAS287-15	Morula granulata	Morula granulata				
QWEAS290-15	Morula granulata	Morula granulata				
QWEAS289-15	Morula granulata	Morula granulata				
SSEO1128-16	Morula zebrina	Morula zebrina	Species	BOLD:ACH7627	10	Morula uva[4], Morula zebrina[2], Morula aspera[1]
QWEAS039-15	Mytilus coruscus	Mytilus coruscus	Species	BOLD:AAB1503	133	Mytilus coruscus[115], Mytilus galloprovincialis[16]
QWEAS043-15	Mytilus coruscus	Mytilus coruscus				
QWEAS040-15	Mytilus coruscus	Mytilus coruscus				
QWEAS042-15	Mytilus coruscus	Mytilus coruscus				
QWEAS041-15	Mytilus coruscus	Mytilus coruscus				

QWEAS044-15	Mytilus coruscus	Mytilus coruscus				
QWEAS045-15	Mytilus coruscus	Mytilus coruscus				
SSEO1465-16	Mytilus galloprovincialis	Mytilus galloprovincialis				
SSEO1459-16	Mytilus galloprovincialis	Mytilus galloprovincialis				
SSEO1461-16	Mytilus galloprovincialis	Mytilus galloprovincialis				
SSEO1460-16	Mytilus galloprovincialis	Mytilus galloprovincialis				
SSEO1463-16	Mytilus galloprovincialis	Mytilus galloprovincialis				
SSEO1458-16	Mytilus galloprovincialis	Mytilus galloprovincialis				
SSEO1462-16	Mytilus galloprovincialis	Mytilus galloprovincialis				
SSEO1464-16	Mytilus galloprovincialis	Mytilus galloprovincialis				
SSEO1457-16	Mytilus edulis	Mytilus edulis	Species	BOLD:AAA2184	550	Mytilus galloprovincialis[285], Mytilus edulis[121], Mytilus sp. 2[69], Mytilus trossulus[19], Mytilus sp.[7], Mytilus sp. CHLF1[1], Mytilus sp. CHGF6[1], Mytilus sp. CHGF5[1], Mytilus sp. CHLF2[1], Mytilus sp. CHLF5[1], Mytilus sp. WHBF2[1], Mytilus sp. CHLF6[1], Mytilus sp. CHGF4[1], Mytilus sp. CHLF4[1], Mytilus sp. CHLF3[1], Mytilus sp. CHGF1[1], Mytilus sp. AFRF4[1], Mytilus sp. AFRF3[1], Mytilus sp. AFRF2[1], Mytilus sp. AFRF1[1], Mytilus sp. AUSF1[1], Mytilus sp. AUSF2[1], Mytilus sp. CHGF2[1], Mytilus sp. AUSF4[1], Mytilus sp. AUSF3[1], Mytilus sp. CHGF3[1]
QWEAS037-15	Mytilus galloprovincialis	Mytilus galloprovincialis				
QWEAS031-15	Mytilus galloprovincialis	Mytilus galloprovincialis				
QWEAS034-15	Mytilus galloprovincialis	Mytilus galloprovincialis				
QWEAS038-15	Mytilus galloprovincialis	Mytilus galloprovincialis				
QWEAS032-15	Mytilus galloprovincialis	Mytilus galloprovincialis				
QWEAS036-15	Mytilus galloprovincialis	Mytilus galloprovincialis				
QWEAS033-15	Mytilus galloprovincialis	Mytilus galloprovincialis				
QWEAS035-15	Mytilus galloprovincialis	Mytilus galloprovincialis				
QWEAS1565-15	Nassarius dorsatus	Nassarius dorsatus	Species	BOLD:ACB6997	5	Nassarius dorsatus[4], Nassarius sufflatus[1]
QWEAS1566-15	Nassarius dorsatus	Nassarius dorsatus				

QWEAS298-15	Nassarius hepaticus	Nassarius hepaticus	Species	BOLD:AAB3092	62	Nassarius hepaticus[60], Nassarius festivus[2]
QWEAS300-15	Nassarius hepaticus	Nassarius hepaticus				
QWEAS299-15	Nassarius hepaticus	Nassarius hepaticus				
QWEAS297-15	Nassarius hepaticus	Nassarius hepaticus				
QWEAS302-15	Nassarius hepaticus	Nassarius hepaticus				
QWEAS301-15	Nassarius hepaticus	Nassarius hepaticus				
SSEO1141-16	Neptunea arthritica	Neptunea arthritica	Species	BOLD:AAC0450	10	Neptunea arthritica[7], Neptunea cumingi[3]
SSEO1139-16	Neptunea arthritica	Neptunea arthritica				
SSEO1140-16	Neptunea arthritica	Neptunea arthritica				
SSEO1373-16	Neptunea frater	Neptunea frater	Species	BOLD:AAF4517	12	Neptunea frater[9], Neptunea kuroshio[3]
SSEO1156-16	Neptunea frater	Neptunea frater				
SSEO1157-16	Neptunea frater	Neptunea frater				
SSEO1160-16	Neptunea kuroshio	Neptunea kuroshio				
SSEO1158-16	Neptunea intersculpta	Neptunea intersculpta	Species	BOLD:AAM4420	5	Neptunea intersculpta[4], Neptunea constricta[1]
SSEO1159-16	Neptunea intersculpta	Neptunea intersculpta				
SSEO466-16	Neritina petitii	Neritina petitii	Species	BOLD:AAM1170	11	Neritina petitii[8], Neritina canalis[1]
SSEO468-16	Neritina petitii	Neritina petitii				
SSEO464-16	Neritina petitii	Neritina petitii				
QWEAS459-15	Nipponacmea concinna	Nipponacmea concinna	Species	BOLD:ACS5305	45	Nipponacmea nigrans[42], Nipponacmea concinna[3]
QWEAS1610-15	Nipponacmea nigrans	Nipponacmea nigrans				
QWEAS1611-15	Nipponacmea nigrans	Nipponacmea nigrans				
QWEAS1609-15	Nipponacmea nigrans	Nipponacmea nigrans				
QWEAS1612-15	Nipponacmea nigrans	Nipponacmea nigrans				
QWEAS1608-15	Nipponacmea nigrans	Nipponacmea nigrans				
QWEAS1603-15	Nipponacmea fuscoviridis	Nipponacmea fuscoviridis	Species	BOLD:ACS4711	163	Nipponacmea fuscoviridis[157], Nipponacmea sp. JL2014[3], Nipponacmea schrenckii[3]
QWEAS1605-15	Nipponacmea fuscoviridis	Nipponacmea fuscoviridis				
QWEAS1607-15	Nipponacmea fuscoviridis	Nipponacmea fuscoviridis				

QWEAS1606-15	Nipponacmea fuscoviridis	Nipponacmea fuscoviridis				
QWEAS1604-15	Nipponacmea fuscoviridis	Nipponacmea fuscoviridis				
QWEAS1614-15	Nipponacmea radula	Nipponacmea radula	Species	BOLD:AAX6432	113	Nipponacmea radula[92], Nipponacmea schrenckii[21]
QWEAS1615-15	Nipponacmea radula	Nipponacmea radula				
QWEAS1617-15	Nipponacmea radula	Nipponacmea radula				
QWEAS1613-15	Nipponacmea radula	Nipponacmea radula				
QWEAS1616-15	Nipponacmea radula	Nipponacmea radula				
SSEO541-16	Nipponacmea radula	Nipponacmea radula				
QWEAS448-15	Nipponacmea schrenckii	Nipponacmea schrenckii				
QWEAS450-15	Nipponacmea schrenckii	Nipponacmea schrenckii				
QWEAS451-15	Nipponacmea schrenckii	Nipponacmea schrenckii				
QWEAS449-15	Nipponacmea schrenckii	Nipponacmea schrenckii				
QWEAS447-15	Nipponacmea schrenckii	Nipponacmea schrenckii				
QWEAS446-15	Nipponacmea schrenckii	Nipponacmea schrenckii				
SSEO337-16	Octopus cyanea	Octopus cyanea	Species	BOLD:AAF4471	21	Octopus cyanea[11], Octopus sp.[8], Octopus bocki[1]
SSEO338-16	Octopus cyanea	Octopus cyanea				
QWEAS1108-15	Paphia gallus	Paphia gallus	Species	BOLD:AAK5288	55	Paphia gallus[38], Paphia exarata[1]
QWEAS1110-15	Paphia gallus	Paphia gallus				
QWEAS1395-15	Paphia gallus	Paphia gallus				
QWEAS1390-15	Paphia gallus	Paphia gallus				
QWEAS1398-15	Paphia gallus	Paphia gallus				
QWEAS1397-15	Paphia gallus	Paphia gallus				
QWEAS1392-15	Paphia gallus	Paphia gallus				
QWEAS1401-15	Paphia gallus	Paphia gallus				
QWEAS1391-15	Paphia gallus	Paphia gallus				
QWEAS1399-15	Paphia gallus	Paphia gallus				
QWEAS1400-15	Paphia gallus	Paphia gallus				
QWEAS1394-15	Paphia gallus	Paphia gallus				

QWEAS1402-15	Paphia gallus	Paphia gallus				
QWEAS1396-15	Paphia gallus	Paphia gallus				
QWEAS1393-15	Paphia gallus	Paphia gallus				
QWEAS1229-15	Paphia gallus	Paphia gallus				
QWEAS1228-15	Paphia gallus	Paphia gallus				
QWEAS1109-15	Paphia gallus	Paphia gallus				
QWEAS1230-15	Paphia gallus	Paphia gallus				
QWEAS1111-15	Paphia textile	Paphia textile	Species	BOLD:AAO8673	12	Paphia textile[6], Paphia undulata[2]
QWEAS1112-15	Paphia textile	Paphia textile				
QWEAS1466-15	Paphia textile	Paphia textile				
QWEAS1468-15	Paphia undulata	Paphia undulata				
QWEAS440-15	Patelloida pygmaea	Patelloida pygmaea	Species	BOLD:ACB8437	19	Patelloida pygmaea[9], Patelloida conulus[9]
QWEAS442-15	Patelloida pygmaea	Patelloida pygmaea				
QWEAS439-15	Patelloida pygmaea	Patelloida pygmaea				
QWEAS441-15	Patelloida pygmaea	Patelloida pygmaea				
QWEAS435-15	Patelloida pygmaea	Patelloida pygmaea				
QWEAS438-15	Patelloida pygmaea	Patelloida pygmaea				
QWEAS437-15	Patelloida pygmaea	Patelloida pygmaea				
QWEAS436-15	Patelloida pygmaea	Patelloida pygmaea				
QWEAS443-15	Patelloida pygmaea	Patelloida pygmaea				
QWEAS455-15	Patelloida saccharina lanx	Patelloida saccharina lanx	Species	BOLD:AAE7072	15	Patelloida saccharina lanx[14], Patelloida sp. ESU 1[1]
QWEAS453-15	Patelloida saccharina lanx	Patelloida saccharina lanx				
QWEAS456-15	Patelloida saccharina lanx	Patelloida saccharina lanx				
QWEAS458-15	Patelloida saccharina lanx	Patelloida saccharina lanx				
QWEAS452-15	Patelloida saccharina lanx	Patelloida saccharina lanx				

QWEAS454-15	Patelloida saccharina lanx	Patelloida saccharina lanx				
QWEAS457-15	Patelloida saccharina lanx	Patelloida saccharina lanx				
QWEAS1085-15	Pelecyora isocardia	Pelecyora isocardia	Species	BOLD:AAO7896	8	Pelecyora isocardia[4], Pelecyora trigona[2]
QWEAS1084-15	Pelecyora isocardia	Pelecyora isocardia				
QWEAS1498-15	Pelecyora trigona	Pelecyora trigona				
QWEAS1500-15	Periglypta compressa	Periglypta compressa	Species	BOLD:AAL2655	6	Periglypta puerpera[4], Periglypta compressa[2]
QWEAS1204-15	Periglypta puerpera	Periglypta puerpera				
QWEAS1203-15	Periglypta puerpera	Periglypta puerpera	Species	BOLD:AAL2654	35	Periglypta puerpera[34], Periglypta puerperal[1]
QWEAS1194-15	Periglypta puerpera	Periglypta puerpera				
QWEAS1199-15	Periglypta puerpera	Periglypta puerpera				
QWEAS1201-15	Periglypta puerpera	Periglypta puerpera				
QWEAS1197-15	Periglypta puerpera	Periglypta puerpera				
QWEAS1196-15	Periglypta puerpera	Periglypta puerpera				
QWEAS1198-15	Periglypta puerpera	Periglypta puerpera				
QWEAS1202-15	Periglypta puerpera	Periglypta puerpera				
QWEAS1200-15	Periglypta puerpera	Periglypta puerpera				
QWEAS1195-15	Periglypta puerpera	Periglypta puerpera				
QWEAS1193-15	Periglypta puerpera	Periglypta puerpera				
QWEAS1569-15	Peronia verruculata	Peronia verruculata	Species	BOLD:AAM2482	25	Peronia verruculata[18], Peronia sp. CC-2010[3]
QWEAS1567-15	Peronia verruculata	Peronia verruculata				
QWEAS1568-15	Peronia verruculata	Peronia verruculata				
SSEO763-16	Pinctada albina	Pinctada albina	Species	BOLD:AAH9358	4	Pinctada albina[2], Pinctada maculata[1]
QWEAS844-15	Pinctada fucata	Pinctada fucata	Species	BOLD:AAY3639	9	Pinctada fucata[7], Pinctada martensi[4]
QWEAS841-15	Pinctada fucata	Pinctada fucata				
SSEO785-16	Pinctada fucata	Pinctada fucata				
QWEAS843-15	Pinctada fucata	Pinctada fucata				
QWEAS840-15	Pinctada fucata	Pinctada fucata				

QWEAS845-15	Pinctada fucata	Pinctada fucata				
QWEAS842-15	Pinctada fucata	Pinctada fucata				
SSEO786-16	Pinctada martensi	Pinctada martensi				
SSEO749-16	Pinctada martensi	Pinctada martensi				
QWEAS846-15	Pinctada margaritifera	Pinctada margaritifera	Species	BOLD:AAY3638	15	Pinctada margaritifera[12], Pinctada maxima[1]
QWEAS852-15	Pinctada margaritifera	Pinctada margaritifera				
QWEAS855-15	Pinctada margaritifera	Pinctada margaritifera				
QWEAS854-15	Pinctada margaritifera	Pinctada margaritifera				
QWEAS849-15	Pinctada margaritifera	Pinctada margaritifera				
SSEO764-16	Pinctada margaritifera	Pinctada margaritifera				
QWEAS858-15	Pinctada sp.	Pinctada sp.	Species	BOLD:ACX4447	4	Pinctada sp.[3], Pinctada maculata[1]
QWEAS856-15	Pinctada sp.	Pinctada sp.				
QWEAS859-15	Pinctada sp.	Pinctada sp.				
QWEAS1081-15	Pitar japonicum	Pitar japonicum	Species	BOLD:AAO6833	4	Pitar japonicum[2], Pitar sulfureum[1]
QWEAS1079-15	Pitar striatus	Pitar striatus	Species	BOLD:AAL4095	11	Pitar striatus[7], Pitar japonicum[1]
QWEAS1078-15	Pitar striatus	Pitar striatus				
QWEAS1080-15	Pitar striatus	Pitar striatus				
SSEO161-16	Plakobranchus ocellatus	Plakobranchus ocellatus	Species	BOLD:ACB7131	49	Plakobranchus ocellatus[41], Plakobranchus sp. white JV2013[7], Plakobranchus cf. ocellatus KH2010[1]
SSEO164-16	Plakobranchus ocellatus	Plakobranchus ocellatus				
SSEO163-16	Plakobranchus ocellatus	Plakobranchus ocellatus	Species	BOLD:ACH4500	4	Plakobranchus sp. purple JV2013[2], Plakobranchus ocellatus[2]
SSEO165-16	Plakobranchus ocellatus	Plakobranchus ocellatus	Species	BOLD:ACH4499	3	Plakobranchus ocellatus[2], Plakobranchus sp. black JV2013[1]
SSEO162-16	Plakobranchus ocellatus	Plakobranchus ocellatus	Species	BOLD:ACH4501	5	Plakobranchus sp. blue JV2013[3], Plakobranchus ocellatus[2]
QWEAS1579-15	Platevindex mortoni	Platevindex mortoni	Species	BOLD:AAM4035	6	Platevindex cf. mortoni

						HDS-2009[4], Platevindex mortoni[2]
SSEO1116-16	Rapana bezoar	Rapana bezoar	Species	BOLD:AAA6876	97	Rapana venosa[91], Rapana bezoar[1]
QWEAS332-15	Rapana venosa	Rapana venosa				
QWEAS334-15	Rapana venosa	Rapana venosa				
QWEAS331-15	Rapana venosa	Rapana venosa				
QWEAS330-15	Rapana venosa	Rapana venosa				
QWEAS333-15	Rapana venosa	Rapana venosa				
SSEO1495-16	Rapana venosa	Rapana venosa				
SSEO1493-16	Rapana venosa	Rapana venosa				
SSEO1494-16	Rapana venosa	Rapana venosa				
QWEAS343-15	Rapana rapiformis	Rapana rapiformis	Species	BOLD:AAY0420	16	Rapana rapiformis[14], Rapana rapiormis[1]
QWEAS344-15	Rapana rapiformis	Rapana rapiformis				
QWEAS342-15	Rapana rapiformis	Rapana rapiformis				
QWEAS345-15	Rapana rapiformis	Rapana rapiformis				
QWEAS341-15	Rapana rapiformis	Rapana rapiformis				
QWEAS340-15	Rapana rapiformis	Rapana rapiformis				
SSEO283-16	Roboastra gracilis	Roboastra gracilis	Species	BOLD:AAX8780	3	Roboastra gracilis[2], Roboastra tigris[1]
QWEAS024-15	Saccostrea mordax	Saccostrea mordax	Species	BOLD:AAM4926	15	Saccostrea mordax[10], Saccostrea cuccullata[1]
QWEAS027-15	Saccostrea mordax	Saccostrea mordax				
QWEAS028-15	Saccostrea mordax	Saccostrea mordax				
QWEAS025-15	Saccostrea mordax	Saccostrea mordax				
QWEAS026-15	Saccostrea mordax	Saccostrea mordax				
QWEAS151-15	Sepia esculenta	Sepia esculenta	Species	BOLD:AAE9622	27	Sepia esculenta[16], Sepia aculeata[1]
QWEAS156-15	Sepia esculenta	Sepia esculenta				
QWEAS157-15	Sepia esculenta	Sepia esculenta				
QWEAS154-15	Sepia esculenta	Sepia esculenta				
QWEAS150-15	Sepia esculenta	Sepia esculenta				
QWEAS153-15	Sepia esculenta	Sepia esculenta				

QWEAS152-15	Sepia esculenta	Sepia esculenta				
QWEAS155-15	Sepia esculenta	Sepia esculenta				
SSEO064-16	Sepia lorigera	Sepia lorigera	Species	BOLD:AAJ7641	3	Sepia lorigera[2], Sepia sp. SI0604[1]
SSEO192-16	Sepia tenuipes	Sepia tenuipes	Species	BOLD:AAA1559	317	Sepia officinalis[314], Sepia tenuipes[2]
SSEO062-16	Sepiella maindroni	Sepiella maindroni	Species	BOLD:AAD8672	2	Sepiella maindroni[1], Sepiella inermis[1]
SSEO328-16	Sepioteuthis lessoniana	Sepioteuthis lessoniana	Species	BOLD:AAA9505	57	Sepioteuthis lessoniana[36], Sepioteuthis cf. lessoniana[15], Sepioteuthis cf. lessoniana VN108[1], Sepioteuthis cf. lessoniana VN068[1], Sepioteuthis cf. lessoniana BAL00610[1], Sepioteuthis cf. lessoniana ACEH12MAY02[1], Sepioteuthis cf. lessoniana BAL00501[1], Sepioteuthis cf. lessoniana BAL00637[1]
SSEO317-16	Sepioteuthis lessoniana	Sepioteuthis lessoniana				
SSEO327-16	Sepioteuthis lessoniana	Sepioteuthis lessoniana				
SSEO319-16	Sepioteuthis lessoniana	Sepioteuthis lessoniana				
SSEO318-16	Sepioteuthis lessoniana	Sepioteuthis lessoniana				
SSEO316-16	Sepioteuthis lessoniana	Sepioteuthis lessoniana				
SSEO326-16	Sepioteuthis lessoniana	Sepioteuthis lessoniana				
SSEO320-16	Sepioteuthis lessoniana	Sepioteuthis lessoniana				
SSEO330-16	Sepioteuthis lessoniana	Sepioteuthis lessoniana	Species	BOLD:AAA9503	138	Sepioteuthis cf. lessoniana[74], Sepioteuthis lessoniana[45], Sepioteuthis cf. lessoniana TIC00513[1], Sepioteuthis cf. lessoniana TIC008[1], Sepioteuthis cf. lessoniana TIC00421[1], Sepioteuthis cf. lessoniana TIC022[1], Sepioteuthis cf. lessoniana VN150[1], Sepioteuthis cf. lessoniana VN070[1], Sepioteuthis cf. lessoniana
SSEO333-16	Sepioteuthis lessoniana	Sepioteuthis lessoniana				
SSEO334-16	Sepioteuthis lessoniana	Sepioteuthis lessoniana				
SSEO329-16	Sepioteuthis lessoniana	Sepioteuthis lessoniana				
SSEO331-16	Sepioteuthis lessoniana	Sepioteuthis lessoniana				
SSEO332-16	Sepioteuthis lessoniana	Sepioteuthis lessoniana				
SSEO003-15	Sepioteuthis lessoniana	Sepioteuthis lessoniana				
SSEO002-15	Sepioteuthis lessoniana	Sepioteuthis lessoniana				
SSEO001-15	Sepioteuthis lessoniana	Sepioteuthis lessoniana				
QWEAS1354-15	Sepioteuthis lessoniana	Sepioteuthis lessoniana				

						TIC00101[1], Sepioteuthis cf. lessoniana TIC016[1], Sepioteuthis cf. lessoniana RAJ005[1], Sepioteuthis cf. lessoniana DUM035[1], Sepioteuthis cf. lessoniana DON013[1], Sepioteuthis cf. lessoniana BAL00604[1], Sepioteuthis cf. lessoniana DUM04402[1], Sepioteuthis cf. lessoniana DUM04410[1], Sepioteuthis cf. lessoniana RAJ00401[1], Sepioteuthis cf. lessoniana PSE001[1], Sepioteuthis cf. lessoniana MAN040[1], Sepioteuthis cf. lessoniana RAJ020[1]
QWEAS1483-15	Siphonaria japonica	Siphonaria japonica	Species	BOLD:ACH7183	264	Siphonaria japonica[259], Siphonaria sp.[2], Siphonaria sp. UF 350553[1], Siphonaria sp. UF 310519[1], Siphonaria sp. UF 310521[1]
QWEAS1481-15	Siphonaria japonica	Siphonaria japonica				
QWEAS1476-15	Siphonaria japonica	Siphonaria japonica				
QWEAS1478-15	Siphonaria japonica	Siphonaria japonica				
QWEAS1482-15	Siphonaria japonica	Siphonaria japonica				
QWEAS1479-15	Siphonaria japonica	Siphonaria japonica				
QWEAS1475-15	Siphonaria japonica	Siphonaria japonica				
QWEAS1477-15	Siphonaria japonica	Siphonaria japonica				
QWEAS1484-15	Siphonaria japonica	Siphonaria japonica				
QWEAS1485-15	Siphonaria japonica	Siphonaria japonica				
QWEAS1480-15	Siphonaria japonica	Siphonaria japonica				
QWEAS1486-15	Siphonaria japonica	Siphonaria japonica				
SSEO290-16	Tambja morosa	Tambja morosa	Species	BOLD:AAH9566	5	Tambja morosa[3], Tambja

						olivaria[2]
SSEO1261-16	Terebralia palustris	Terebralia palustris	Species	BOLD:AAF2150	191	Terebralia palustris[190], Terebralia sulcata[1]
QWEAS355-15	Thais javanica	Thais javanica	Species	BOLD:ACB7301	7	Thais javanica[6], Thais rufotincta[1]
QWEAS354-15	Thais javanica	Thais javanica				
SSEO1338-16	Turbo argyrostomus	Turbo argyrostomus	Species	BOLD:AAF6399	6	Turbo cepoides[3], Turbo argyrostomus[3]
SSEO1350-16	Turbo cornutus	Turbo cornutus	Species	BOLD:AAF6398	16	Turbo cornutus[13], Turbo chinensis[3]
SSEO1522-16	Turbo cornutus	Turbo cornutus				
SSEO1521-16	Turbo cornutus	Turbo cornutus				
SSEO1344-16	Turbo cornutus	Turbo cornutus				
SSEO1523-16	Turbo cornutus	Turbo cornutus				
QWEAS467-15	Turritella terebra	Turritella terebra	Species	BOLD:ACB7650	12	Turritella terebra[10], Turritella bacillum[2]
QWEAS465-15	Turritella terebra	Turritella terebra				
QWEAS466-15	Turritella terebra	Turritella terebra				
QWEAS463-15	Turritella terebra	Turritella terebra				
QWEAS464-15	Turritella terebra	Turritella terebra				

Supplementary Fig 1. The relationship between GC content and COI distance across genera. Mean nearest neighbour distance (% K2P) between congeneric species at COI plotted against mean GC content (%) in the 123 genera of marine molluscs. The regression was significant (*P*<0.001; R^2 = 0.167).

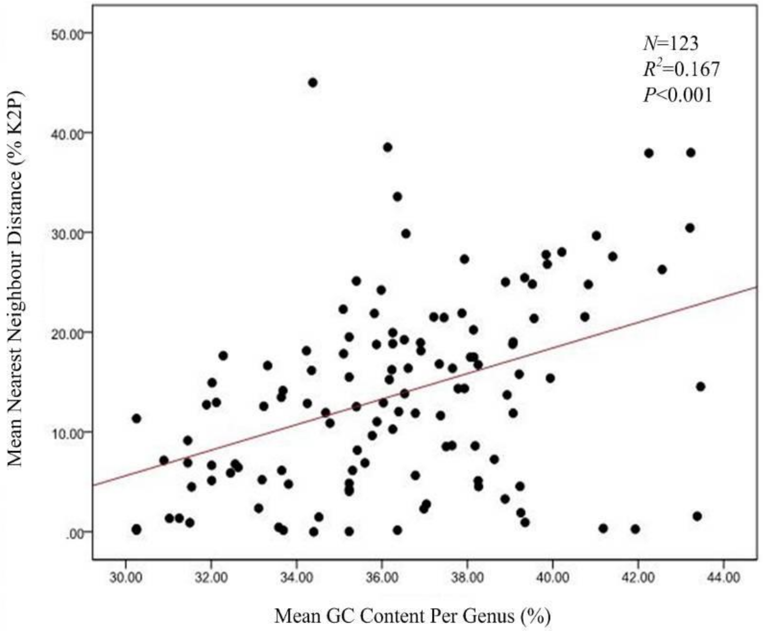

Supplementary Fig 2. Histogram of threshold optimization method reporting the frequencies of false-positive error (no conspecific matches within threshold but conspecific samples available) and false-negative error (more than one species recorded within threshold) identifications across thresholds values from 0.01 to 0.1.

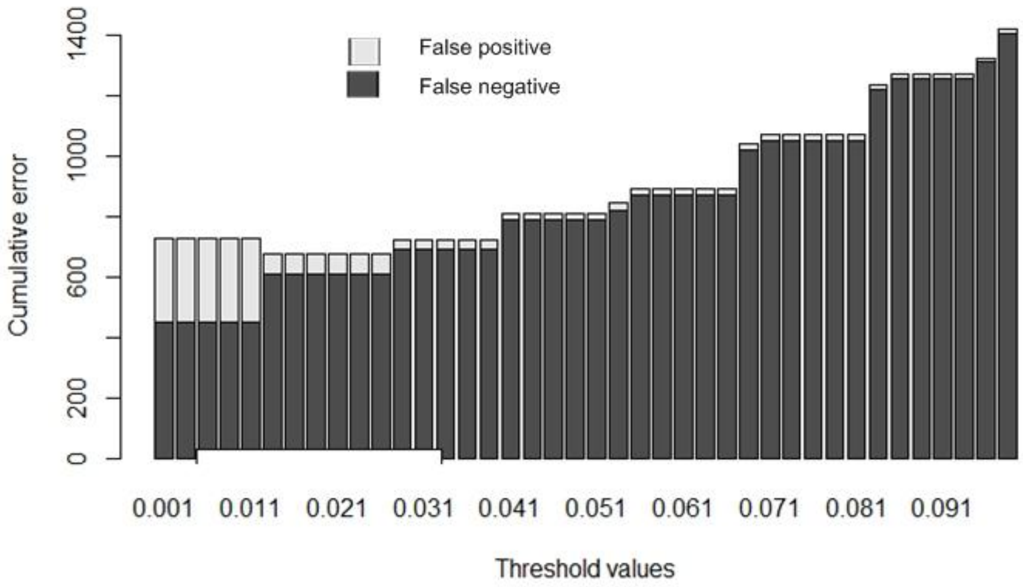

Supplementary Fig 3. Density plot of genetic distances generated by the function 'localMinima' in SPIDER package corresponding to the transition between intra- and interspecific distances. The minimum of a density plot of genetic distances is highlighted in grey.

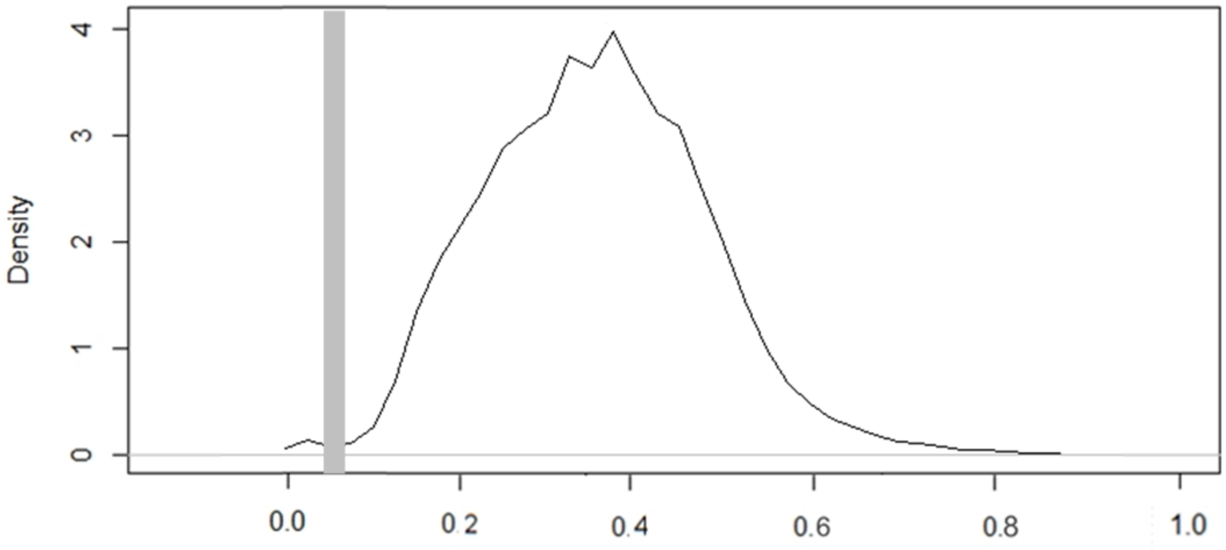

Supplementary Fig 4 Neighbour-Joining (NJ) tree of 2801 COI barcodes based on K2P model (performed on the BOLD database). The highlighted cluster in magenta and yellow indicate species with a high cryptic diversity and species formed paraphyly clusters, respectively. The framed clusters indicate the species characterized by haplotype sharing.

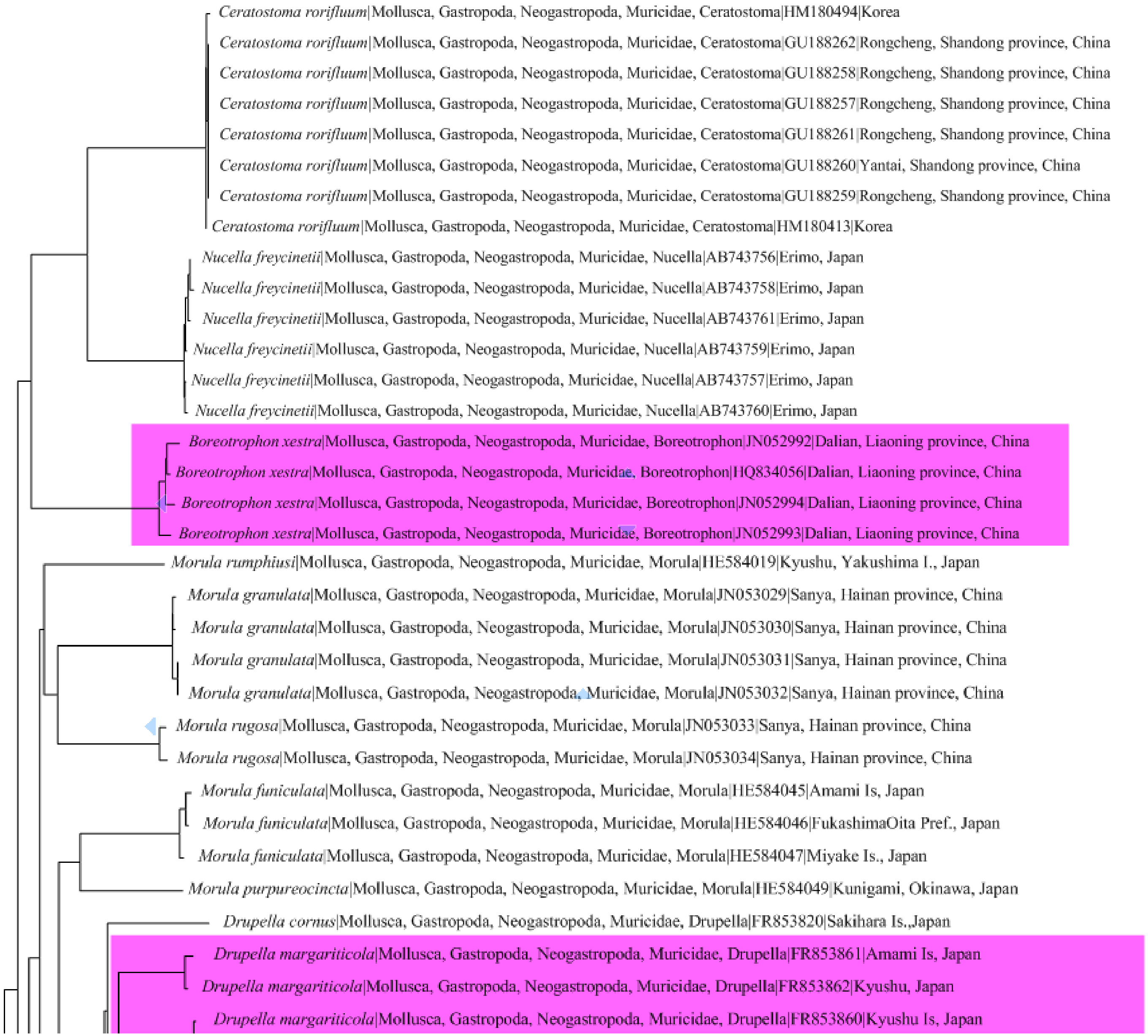

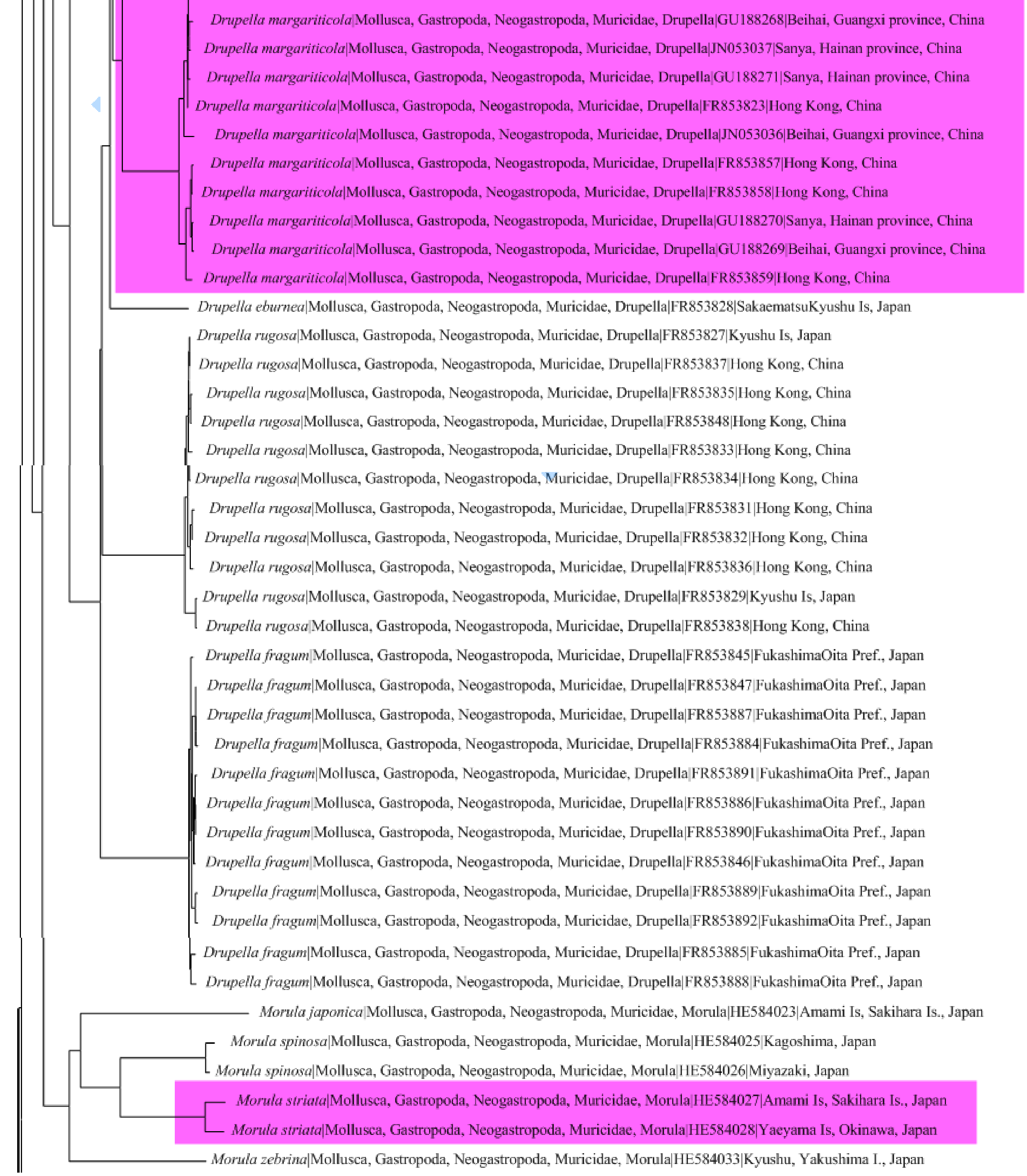

Drupella margariticola|Mollusca, Gastropoda, Neogastropoda, Muricidae, Drupella|GU188268|Beihai, Guangxi province, China
Drupella margariticola|Mollusca, Gastropoda, Neogastropoda, Muricidae, Drupella|JN053037|Sanya, Hainan province, China
Drupella margariticola|Mollusca, Gastropoda, Neogastropoda, Muricidae, Drupella|GU188271|Sanya, Hainan province, China
Drupella margariticola|Mollusca, Gastropoda, Neogastropoda, Muricidae, Drupella|FR853823|Hong Kong, China
Drupella margariticola|Mollusca, Gastropoda, Neogastropoda, Muricidae, Drupella|JN053036|Beihai, Guangxi province, China
Drupella margariticola|Mollusca, Gastropoda, Neogastropoda, Muricidae, Drupella|FR853857|Hong Kong, China
Drupella margariticola|Mollusca, Gastropoda, Neogastropoda, Muricidae, Drupella|FR853858|Hong Kong, China
Drupella margariticola|Mollusca, Gastropoda, Neogastropoda, Muricidae, Drupella|GU188270|Sanya, Hainan province, China
Drupella margariticola|Mollusca, Gastropoda, Neogastropoda, Muricidae, Drupella|GU188269|Beihai, Guangxi province, China
Drupella margariticola|Mollusca, Gastropoda, Neogastropoda, Muricidae, Drupella|FR853859|Hong Kong, China
Drupella eburnea|Mollusca, Gastropoda, Neogastropoda, Muricidae, Drupella|FR853828|SakaematsuKyushu Is, Japan
Drupella rugosa|Mollusca, Gastropoda, Neogastropoda, Muricidae, Drupella|FR853827|Kyushu Is, Japan
Drupella rugosa|Mollusca, Gastropoda, Neogastropoda, Muricidae, Drupella|FR853837|Hong Kong, China
Drupella rugosa|Mollusca, Gastropoda, Neogastropoda, Muricidae, Drupella|FR853835|Hong Kong, China
Drupella rugosa|Mollusca, Gastropoda, Neogastropoda, Muricidae, Drupella|FR853848|Hong Kong, China
Drupella rugosa|Mollusca, Gastropoda, Neogastropoda, Muricidae, Drupella|FR853833|Hong Kong, China
Drupella rugosa|Mollusca, Gastropoda, Neogastropoda, Muricidae, Drupella|FR853834|Hong Kong, China
Drupella rugosa|Mollusca, Gastropoda, Neogastropoda, Muricidae, Drupella|FR853831|Hong Kong, China
Drupella rugosa|Mollusca, Gastropoda, Neogastropoda, Muricidae, Drupella|FR853832|Hong Kong, China
Drupella rugosa|Mollusca, Gastropoda, Neogastropoda, Muricidae, Drupella|FR853836|Hong Kong, China
Drupella rugosa|Mollusca, Gastropoda, Neogastropoda, Muricidae, Drupella|FR853829|Kyushu Is, Japan
Drupella rugosa|Mollusca, Gastropoda, Neogastropoda, Muricidae, Drupella|FR853838|Hong Kong, China
Drupella fragum|Mollusca, Gastropoda, Neogastropoda, Muricidae, Drupella|FR853845|FukashimaOita Pref., Japan
Drupella fragum|Mollusca, Gastropoda, Neogastropoda, Muricidae, Drupella|FR853847|FukashimaOita Pref., Japan
Drupella fragum|Mollusca, Gastropoda, Neogastropoda, Muricidae, Drupella|FR853887|FukashimaOita Pref., Japan
Drupella fragum|Mollusca, Gastropoda, Neogastropoda, Muricidae, Drupella|FR853884|FukashimaOita Pref., Japan
Drupella fragum|Mollusca, Gastropoda, Neogastropoda, Muricidae, Drupella|FR853891|FukashimaOita Pref., Japan
Drupella fragum|Mollusca, Gastropoda, Neogastropoda, Muricidae, Drupella|FR853886|FukashimaOita Pref., Japan
Drupella fragum|Mollusca, Gastropoda, Neogastropoda, Muricidae, Drupella|FR853890|FukashimaOita Pref., Japan
Drupella fragum|Mollusca, Gastropoda, Neogastropoda, Muricidae, Drupella|FR853846|FukashimaOita Pref., Japan
Drupella fragum|Mollusca, Gastropoda, Neogastropoda, Muricidae, Drupella|FR853889|FukashimaOita Pref., Japan
Drupella fragum|Mollusca, Gastropoda, Neogastropoda, Muricidae, Drupella|FR853892|FukashimaOita Pref., Japan
Drupella fragum|Mollusca, Gastropoda, Neogastropoda, Muricidae, Drupella|FR853885|FukashimaOita Pref., Japan
Drupella fragum|Mollusca, Gastropoda, Neogastropoda, Muricidae, Drupella|FR853888|FukashimaOita Pref., Japan
Morula japonica|Mollusca, Gastropoda, Neogastropoda, Muricidae, Morula|HE584023|Amami Is, Sakihara Is., Japan
Morula spinosa|Mollusca, Gastropoda, Neogastropoda, Muricidae, Morula|HE584025|Kagoshima, Japan
Morula spinosa|Mollusca, Gastropoda, Neogastropoda, Muricidae, Morula|HE584026|Miyazaki, Japan
Morula striata|Mollusca, Gastropoda, Neogastropoda, Muricidae, Morula|HE584027|Amami Is, Sakihara Is., Japan
Morula striata|Mollusca, Gastropoda, Neogastropoda, Muricidae, Morula|HE584028|Yaeyama Is, Okinawa, Japan
Morula zebrina|Mollusca, Gastropoda, Neogastropoda, Muricidae, Morula|HE584033|Kyushu, Yakushima I., Japan

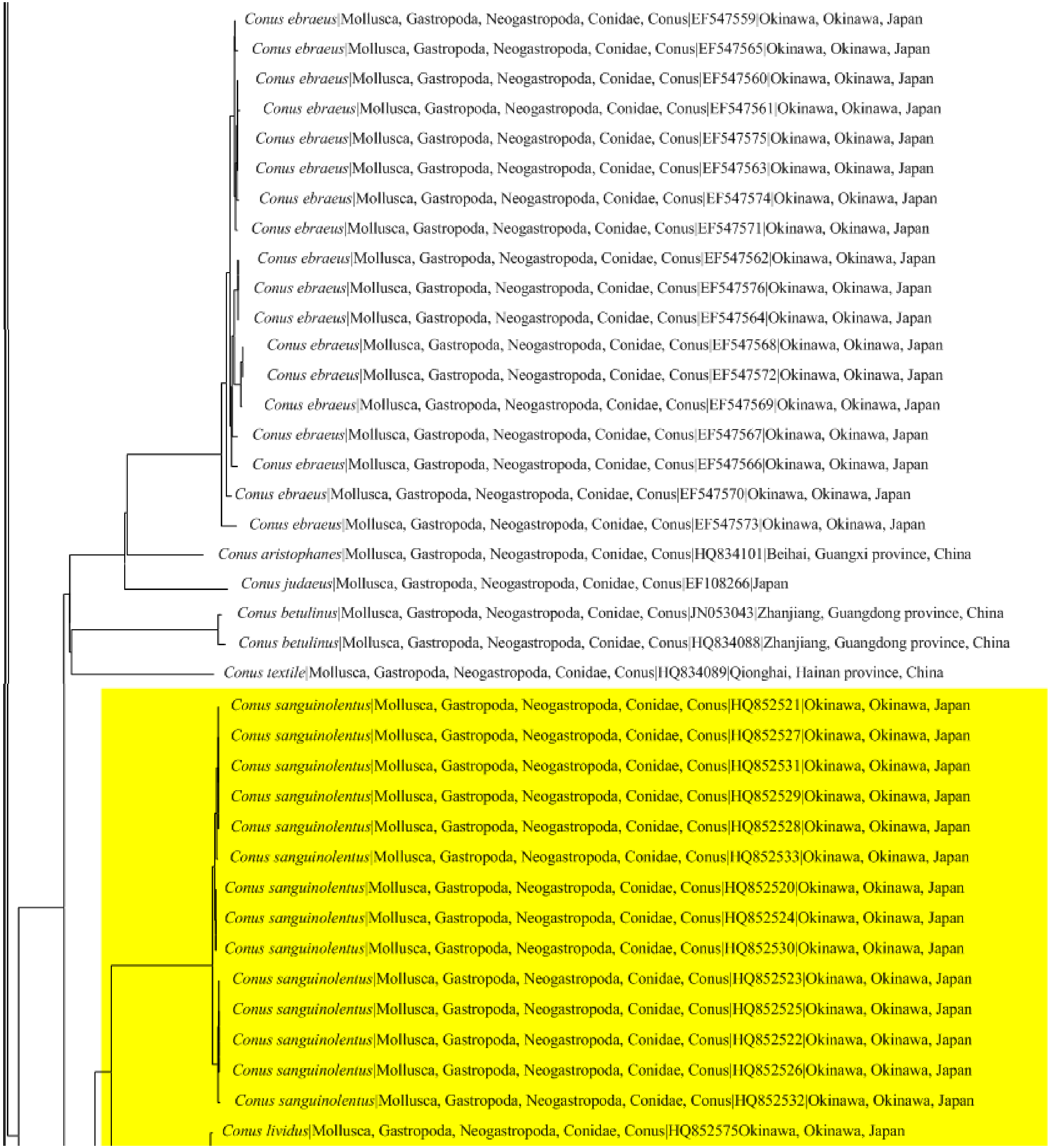
Conus ebraeus|Mollusca, Gastropoda, Neogastropoda, Conidae, Conus|EF547559|Okinawa, Okinawa, Japan
Conus ebraeus|Mollusca, Gastropoda, Neogastropoda, Conidae, Conus|EF547565|Okinawa, Okinawa, Japan
Conus ebraeus|Mollusca, Gastropoda, Neogastropoda, Conidae, Conus|EF547560|Okinawa, Okinawa, Japan
Conus ebraeus|Mollusca, Gastropoda, Neogastropoda, Conidae, Conus|EF547561|Okinawa, Okinawa, Japan
Conus ebraeus|Mollusca, Gastropoda, Neogastropoda, Conidae, Conus|EF547575|Okinawa, Okinawa, Japan
Conus ebraeus|Mollusca, Gastropoda, Neogastropoda, Conidae, Conus|EF547563|Okinawa, Okinawa, Japan
Conus ebraeus|Mollusca, Gastropoda, Neogastropoda, Conidae, Conus|EF547574|Okinawa, Okinawa, Japan
Conus ebraeus|Mollusca, Gastropoda, Neogastropoda, Conidae, Conus|EF547571|Okinawa, Okinawa, Japan
Conus ebraeus|Mollusca, Gastropoda, Neogastropoda, Conidae, Conus|EF547562|Okinawa, Okinawa, Japan
Conus ebraeus|Mollusca, Gastropoda, Neogastropoda, Conidae, Conus|EF547576|Okinawa, Okinawa, Japan
Conus ebraeus|Mollusca, Gastropoda, Neogastropoda, Conidae, Conus|EF547564|Okinawa, Okinawa, Japan
Conus ebraeus|Mollusca, Gastropoda, Neogastropoda, Conidae, Conus|EF547568|Okinawa, Okinawa, Japan
Conus ebraeus|Mollusca, Gastropoda, Neogastropoda, Conidae, Conus|EF547572|Okinawa, Okinawa, Japan
Conus ebraeus|Mollusca, Gastropoda, Neogastropoda, Conidae, Conus|EF547569|Okinawa, Okinawa, Japan
Conus ebraeus|Mollusca, Gastropoda, Neogastropoda, Conidae, Conus|EF547567|Okinawa, Okinawa, Japan
Conus ebraeus|Mollusca, Gastropoda, Neogastropoda, Conidae, Conus|EF547566|Okinawa, Okinawa, Japan
Conus ebraeus|Mollusca, Gastropoda, Neogastropoda, Conidae, Conus|EF547570|Okinawa, Okinawa, Japan
Conus ebraeus|Mollusca, Gastropoda, Neogastropoda, Conidae, Conus|EF547573|Okinawa, Okinawa, Japan
Conus aristophanes|Mollusca, Gastropoda, Neogastropoda, Conidae, Conus|HQ834101|Beihai, Guangxi province, China
Conus judaeus|Mollusca, Gastropoda, Neogastropoda, Conidae, Conus|EF108266|Japan
Conus betulinus|Mollusca, Gastropoda, Neogastropoda, Conidae, Conus|JN053043|Zhanjiang, Guangdong province, China
Conus betulinus|Mollusca, Gastropoda, Neogastropoda, Conidae, Conus|HQ834088|Zhanjiang, Guangdong province, China
Conus textile|Mollusca, Gastropoda, Neogastropoda, Conidae, Conus|HQ834089|Qionghai, Hainan province, China
Conus sanguinolentus|Mollusca, Gastropoda, Neogastropoda, Conidae, Conus|HQ852521|Okinawa, Okinawa, Japan
Conus sanguinolentus|Mollusca, Gastropoda, Neogastropoda, Conidae, Conus|HQ852527|Okinawa, Okinawa, Japan
Conus sanguinolentus|Mollusca, Gastropoda, Neogastropoda, Conidae, Conus|HQ852531|Okinawa, Okinawa, Japan
Conus sanguinolentus|Mollusca, Gastropoda, Neogastropoda, Conidae, Conus|HQ852529|Okinawa, Okinawa, Japan
Conus sanguinolentus|Mollusca, Gastropoda, Neogastropoda, Conidae, Conus|HQ852528|Okinawa, Okinawa, Japan
Conus sanguinolentus|Mollusca, Gastropoda, Neogastropoda, Conidae, Conus|HQ852533|Okinawa, Okinawa, Japan
Conus sanguinolentus|Mollusca, Gastropoda, Neogastropoda, Conidae, Conus|HQ852520|Okinawa, Okinawa, Japan
Conus sanguinolentus|Mollusca, Gastropoda, Neogastropoda, Conidae, Conus|HQ852524|Okinawa, Okinawa, Japan
Conus sanguinolentus|Mollusca, Gastropoda, Neogastropoda, Conidae, Conus|HQ852530|Okinawa, Okinawa, Japan
Conus sanguinolentus|Mollusca, Gastropoda, Neogastropoda, Conidae, Conus|HQ852523|Okinawa, Okinawa, Japan
Conus sanguinolentus|Mollusca, Gastropoda, Neogastropoda, Conidae, Conus|HQ852525|Okinawa, Okinawa, Japan
Conus sanguinolentus|Mollusca, Gastropoda, Neogastropoda, Conidae, Conus|HQ852522|Okinawa, Okinawa, Japan
Conus sanguinolentus|Mollusca, Gastropoda, Neogastropoda, Conidae, Conus|HQ852526|Okinawa, Okinawa, Japan
Conus sanguinolentus|Mollusca, Gastropoda, Neogastropoda, Conidae, Conus|HQ852532|Okinawa, Okinawa, Japan
Conus lividus|Mollusca, Gastropoda, Neogastropoda, Conidae, Conus|HQ852575Okinawa, Okinawa, Japan

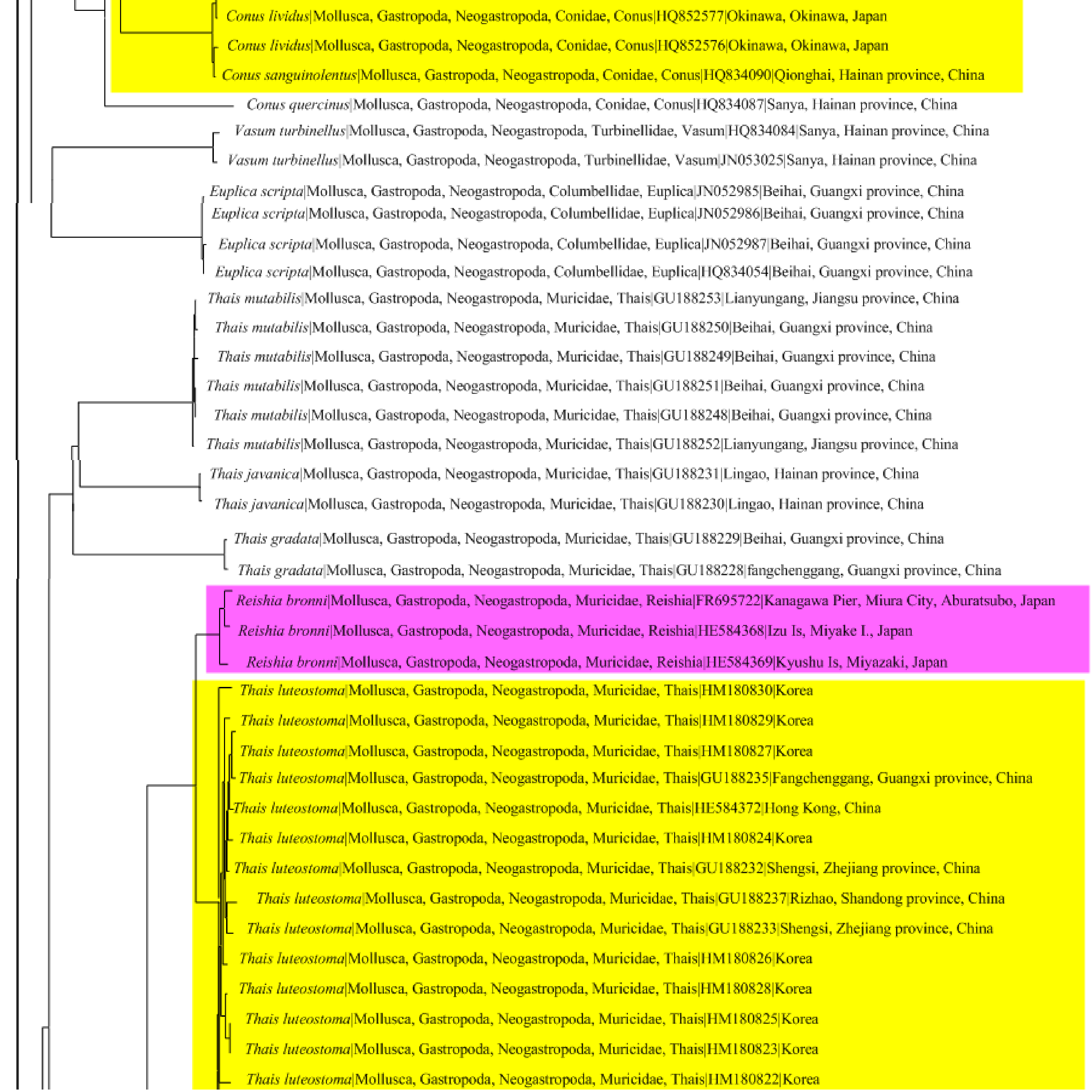
Conus lividus|Mollusca, Gastropoda, Neogastropoda, Conidae, Conus|HQ852577|Okinawa, Okinawa, Japan
Conus lividus|Mollusca, Gastropoda, Neogastropoda, Conidae, Conus|HQ852576|Okinawa, Okinawa, Japan
Conus sanguinolentus|Mollusca, Gastropoda, Neogastropoda, Conidae, Conus|HQ834090|Qionghai, Hainan province, China
Conus quercinus|Mollusca, Gastropoda, Neogastropoda, Conidae, Conus|HQ834087|Sanya, Hainan province, China
Vasum turbinellus|Mollusca, Gastropoda, Neogastropoda, Turbinellidae, Vasum|HQ834084|Sanya, Hainan province, China
Vasum turbinellus|Mollusca, Gastropoda, Neogastropoda, Turbinellidae, Vasum|JN053025|Sanya, Hainan province, China
Euplica scripta|Mollusca, Gastropoda, Neogastropoda, Columbellidae, Euplica|JN052985|Beihai, Guangxi province, China
Euplica scripta|Mollusca, Gastropoda, Neogastropoda, Columbellidae, Euplica|JN052986|Beihai, Guangxi province, China
Euplica scripta|Mollusca, Gastropoda, Neogastropoda, Columbellidae, Euplica|JN052987|Beihai, Guangxi province, China
Euplica scripta|Mollusca, Gastropoda, Neogastropoda, Columbellidae, Euplica|HQ834054|Beihai, Guangxi province, China
Thais mutabilis|Mollusca, Gastropoda, Neogastropoda, Muricidae, Thais|GU188253|Lianyungang, Jiangsu province, China
Thais mutabilis|Mollusca, Gastropoda, Neogastropoda, Muricidae, Thais|GU188250|Beihai, Guangxi province, China
Thais mutabilis|Mollusca, Gastropoda, Neogastropoda, Muricidae, Thais|GU188249|Beihai, Guangxi province, China
Thais mutabilis|Mollusca, Gastropoda, Neogastropoda, Muricidae, Thais|GU188251|Beihai, Guangxi province, China
Thais mutabilis|Mollusca, Gastropoda, Neogastropoda, Muricidae, Thais|GU188248|Beihai, Guangxi province, China
Thais mutabilis|Mollusca, Gastropoda, Neogastropoda, Muricidae, Thais|GU188252|Lianyungang, Jiangsu province, China
Thais javanica|Mollusca, Gastropoda, Neogastropoda, Muricidae, Thais|GU188231|Lingao, Hainan province, China
Thais javanica|Mollusca, Gastropoda, Neogastropoda, Muricidae, Thais|GU188230|Lingao, Hainan province, China
Thais gradata|Mollusca, Gastropoda, Neogastropoda, Muricidae, Thais|GU188229|Beihai, Guangxi province, China
Thais gradata|Mollusca, Gastropoda, Neogastropoda, Muricidae, Thais|GU188228|fangchenggang, Guangxi province, China
Reishia bronni|Mollusca, Gastropoda, Neogastropoda, Muricidae, Reishia|FR695722|Kanagawa Pier, Miura City, Aburatsubo, Japan
Reishia bronni|Mollusca, Gastropoda, Neogastropoda, Muricidae, Reishia|HE584368|Izu Is, Miyake I., Japan
Reishia bronni|Mollusca, Gastropoda, Neogastropoda, Muricidae, Reishia|HE584369|Kyushu Is, Miyazaki, Japan
Thais luteostoma|Mollusca, Gastropoda, Neogastropoda, Muricidae, Thais|HM180830|Korea
Thais luteostoma|Mollusca, Gastropoda, Neogastropoda, Muricidae, Thais|HM180829|Korea
Thais luteostoma|Mollusca, Gastropoda, Neogastropoda, Muricidae, Thais|HM180827|Korea
Thais luteostoma|Mollusca, Gastropoda, Neogastropoda, Muricidae, Thais|GU188235|Fangchenggang, Guangxi province, China
Thais luteostoma|Mollusca, Gastropoda, Neogastropoda, Muricidae, Thais|HE584372|Hong Kong, China
Thais luteostoma|Mollusca, Gastropoda, Neogastropoda, Muricidae, Thais|HM180824|Korea
Thais luteostoma|Mollusca, Gastropoda, Neogastropoda, Muricidae, Thais|GU188232|Shengsi, Zhejiang province, China
Thais luteostoma|Mollusca, Gastropoda, Neogastropoda, Muricidae, Thais|GU188237|Rizhao, Shandong province, China
Thais luteostoma|Mollusca, Gastropoda, Neogastropoda, Muricidae, Thais|GU188233|Shengsi, Zhejiang province, China
Thais luteostoma|Mollusca, Gastropoda, Neogastropoda, Muricidae, Thais|HM180826|Korea
Thais luteostoma|Mollusca, Gastropoda, Neogastropoda, Muricidae, Thais|HM180828|Korea
Thais luteostoma|Mollusca, Gastropoda, Neogastropoda, Muricidae, Thais|HM180825|Korea
Thais luteostoma|Mollusca, Gastropoda, Neogastropoda, Muricidae, Thais|HM180823|Korea
Thais luteostoma|Mollusca, Gastropoda, Neogastropoda, Muricidae, Thais|HM180822|Korea

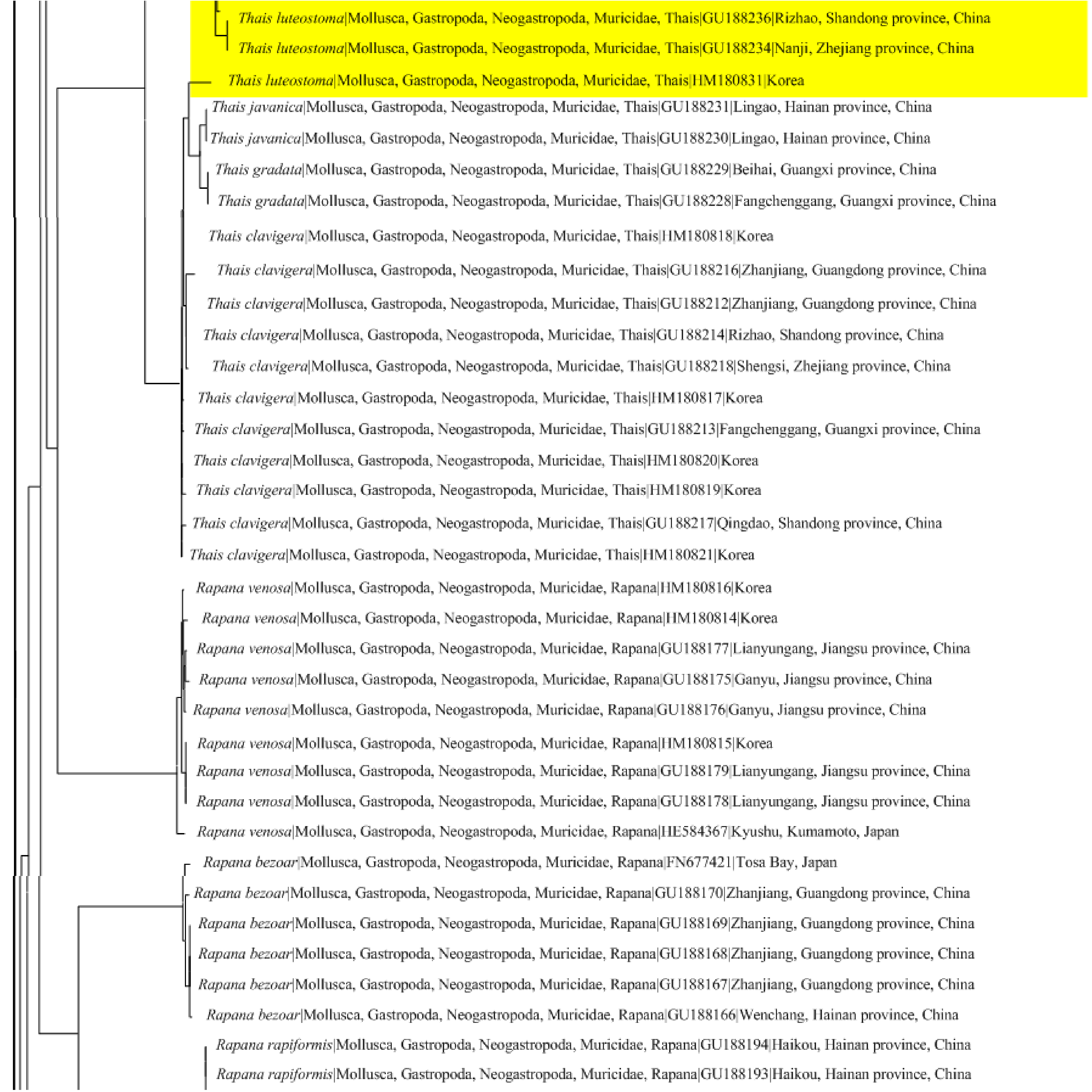

Thais luteostoma|Mollusca, Gastropoda, Neogastropoda, Muricidae, Thais|GU188236|Rizhao, Shandong province, China
Thais luteostoma|Mollusca, Gastropoda, Neogastropoda, Muricidae, Thais|GU188234|Nanji, Zhejiang province, China
Thais luteostoma|Mollusca, Gastropoda, Neogastropoda, Muricidae, Thais|HM180831|Korea
Thais javanica|Mollusca, Gastropoda, Neogastropoda, Muricidae, Thais|GU188231|Lingao, Hainan province, China
Thais javanica|Mollusca, Gastropoda, Neogastropoda, Muricidae, Thais|GU188230|Lingao, Hainan province, China
Thais gradata|Mollusca, Gastropoda, Neogastropoda, Muricidae, Thais|GU188229|Beihai, Guangxi province, China
Thais gradata|Mollusca, Gastropoda, Neogastropoda, Muricidae, Thais|GU188228|Fangchenggang, Guangxi province, China
Thais clavigera|Mollusca, Gastropoda, Neogastropoda, Muricidae, Thais|HM180818|Korea
Thais clavigera|Mollusca, Gastropoda, Neogastropoda, Muricidae, Thais|GU188216|Zhanjiang, Guangdong province, China
Thais clavigera|Mollusca, Gastropoda, Neogastropoda, Muricidae, Thais|GU188212|Zhanjiang, Guangdong province, China
Thais clavigera|Mollusca, Gastropoda, Neogastropoda, Muricidae, Thais|GU188214|Rizhao, Shandong province, China
Thais clavigera|Mollusca, Gastropoda, Neogastropoda, Muricidae, Thais|GU188218|Shengsi, Zhejiang province, China
Thais clavigera|Mollusca, Gastropoda, Neogastropoda, Muricidae, Thais|HM180817|Korea
Thais clavigera|Mollusca, Gastropoda, Neogastropoda, Muricidae, Thais|GU188213|Fangchenggang, Guangxi province, China
Thais clavigera|Mollusca, Gastropoda, Neogastropoda, Muricidae, Thais|HM180820|Korea
Thais clavigera|Mollusca, Gastropoda, Neogastropoda, Muricidae, Thais|HM180819|Korea
Thais clavigera|Mollusca, Gastropoda, Neogastropoda, Muricidae, Thais|GU188217|Qingdao, Shandong province, China
Thais clavigera|Mollusca, Gastropoda, Neogastropoda, Muricidae, Thais|HM180821|Korea
Rapana venosa|Mollusca, Gastropoda, Neogastropoda, Muricidae, Rapana|HM180816|Korea
Rapana venosa|Mollusca, Gastropoda, Neogastropoda, Muricidae, Rapana|HM180814|Korea
Rapana venosa|Mollusca, Gastropoda, Neogastropoda, Muricidae, Rapana|GU188177|Lianyungang, Jiangsu province, China
Rapana venosa|Mollusca, Gastropoda, Neogastropoda, Muricidae, Rapana|GU188175|Ganyu, Jiangsu province, China
Rapana venosa|Mollusca, Gastropoda, Neogastropoda, Muricidae, Rapana|GU188176|Ganyu, Jiangsu province, China
Rapana venosa|Mollusca, Gastropoda, Neogastropoda, Muricidae, Rapana|HM180815|Korea
Rapana venosa|Mollusca, Gastropoda, Neogastropoda, Muricidae, Rapana|GU188179|Lianyungang, Jiangsu province, China
Rapana venosa|Mollusca, Gastropoda, Neogastropoda, Muricidae, Rapana|GU188178|Lianyungang, Jiangsu province, China
Rapana venosa|Mollusca, Gastropoda, Neogastropoda, Muricidae, Rapana|HE584367|Kyushu, Kumamoto, Japan
Rapana bezoar|Mollusca, Gastropoda, Neogastropoda, Muricidae, Rapana|FN677421|Tosa Bay, Japan
Rapana bezoar|Mollusca, Gastropoda, Neogastropoda, Muricidae, Rapana|GU188170|Zhanjiang, Guangdong province, China
Rapana bezoar|Mollusca, Gastropoda, Neogastropoda, Muricidae, Rapana|GU188169|Zhanjiang, Guangdong province, China
Rapana bezoar|Mollusca, Gastropoda, Neogastropoda, Muricidae, Rapana|GU188168|Zhanjiang, Guangdong province, China
Rapana bezoar|Mollusca, Gastropoda, Neogastropoda, Muricidae, Rapana|GU188167|Zhanjiang, Guangdong province, China
Rapana bezoar|Mollusca, Gastropoda, Neogastropoda, Muricidae, Rapana|GU188166|Wenchang, Hainan province, China
Rapana rapiformis|Mollusca, Gastropoda, Neogastropoda, Muricidae, Rapana|GU188194|Haikou, Hainan province, China
Rapana rapiformis|Mollusca, Gastropoda, Neogastropoda, Muricidae, Rapana|GU188193|Haikou, Hainan province, China

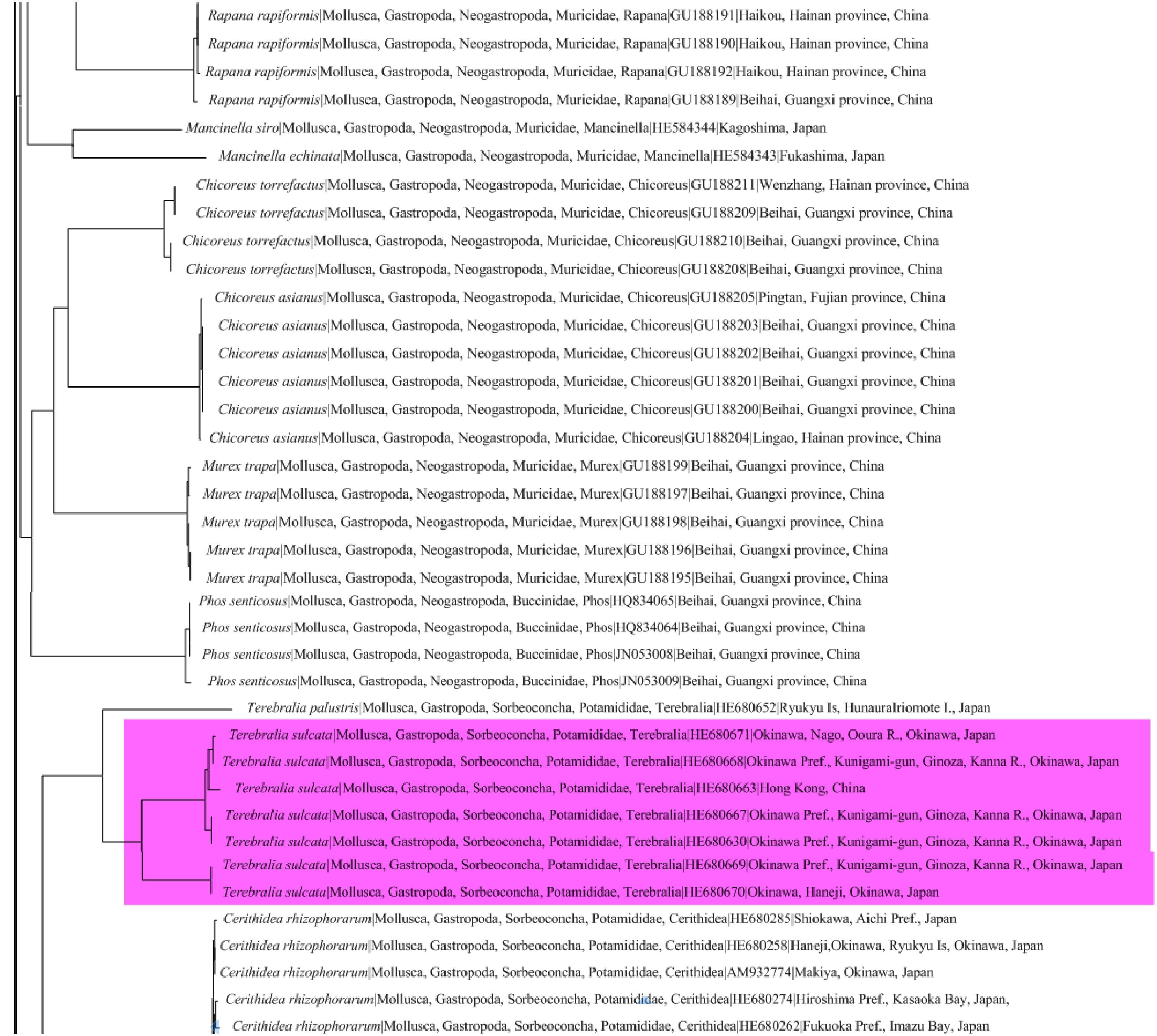

Rapana rapiformis|Mollusca, Gastropoda, Neogastropoda, Muricidae, Rapana|GU188191|Haikou, Hainan province, China
Rapana rapiformis|Mollusca, Gastropoda, Neogastropoda, Muricidae, Rapana|GU188190|Haikou, Hainan province, China
Rapana rapiformis|Mollusca, Gastropoda, Neogastropoda, Muricidae, Rapana|GU188192|Haikou, Hainan province, China
Rapana rapiformis|Mollusca, Gastropoda, Neogastropoda, Muricidae, Rapana|GU188189|Beihai, Guangxi province, China
Mancinella siro|Mollusca, Gastropoda, Neogastropoda, Muricidae, Mancinella|HE584344|Kagoshima, Japan
Mancinella echinata|Mollusca, Gastropoda, Neogastropoda, Muricidae, Mancinella|HE584343|Fukashima, Japan
Chicoreus torrefactus|Mollusca, Gastropoda, Neogastropoda, Muricidae, Chicoreus|GU188211|Wenzhang, Hainan province, China
Chicoreus torrefactus|Mollusca, Gastropoda, Neogastropoda, Muricidae, Chicoreus|GU188209|Beihai, Guangxi province, China
Chicoreus torrefactus|Mollusca, Gastropoda, Neogastropoda, Muricidae, Chicoreus|GU188210|Beihai, Guangxi province, China
Chicoreus torrefactus|Mollusca, Gastropoda, Neogastropoda, Muricidae, Chicoreus|GU188208|Beihai, Guangxi province, China
Chicoreus asianus|Mollusca, Gastropoda, Neogastropoda, Muricidae, Chicoreus|GU188205|Pingtan, Fujian province, China
Chicoreus asianus|Mollusca, Gastropoda, Neogastropoda, Muricidae, Chicoreus|GU188203|Beihai, Guangxi province, China
Chicoreus asianus|Mollusca, Gastropoda, Neogastropoda, Muricidae, Chicoreus|GU188202|Beihai, Guangxi province, China
Chicoreus asianus|Mollusca, Gastropoda, Neogastropoda, Muricidae, Chicoreus|GU188201|Beihai, Guangxi province, China
Chicoreus asianus|Mollusca, Gastropoda, Neogastropoda, Muricidae, Chicoreus|GU188200|Beihai, Guangxi province, China
Chicoreus asianus|Mollusca, Gastropoda, Neogastropoda, Muricidae, Chicoreus|GU188204|Lingao, Hainan province, China
Murex trapa|Mollusca, Gastropoda, Neogastropoda, Muricidae, Murex|GU188199|Beihai, Guangxi province, China
Murex trapa|Mollusca, Gastropoda, Neogastropoda, Muricidae, Murex|GU188197|Beihai, Guangxi province, China
Murex trapa|Mollusca, Gastropoda, Neogastropoda, Muricidae, Murex|GU188198|Beihai, Guangxi province, China
Murex trapa|Mollusca, Gastropoda, Neogastropoda, Muricidae, Murex|GU188196|Beihai, Guangxi province, China
Murex trapa|Mollusca, Gastropoda, Neogastropoda, Muricidae, Murex|GU188195|Beihai, Guangxi province, China
Phos senticosus|Mollusca, Gastropoda, Neogastropoda, Buccinidae, Phos|HQ834065|Beihai, Guangxi province, China
Phos senticosus|Mollusca, Gastropoda, Neogastropoda, Buccinidae, Phos|HQ834064|Beihai, Guangxi province, China
Phos senticosus|Mollusca, Gastropoda, Neogastropoda, Buccinidae, Phos|JN053008|Beihai, Guangxi province, China
Phos senticosus|Mollusca, Gastropoda, Neogastropoda, Buccinidae, Phos|JN053009|Beihai, Guangxi province, China
Terebralia palustris|Mollusca, Gastropoda, Sorbeoconcha, Potamididae, Terebralia|HE680652|Ryukyu Is, HunauraIriomote I., Japan
Terebralia sulcata|Mollusca, Gastropoda, Sorbeoconcha, Potamididae, Terebralia|HE680671|Okinawa, Nago, Ooura R., Okinawa, Japan
Terebralia sulcata|Mollusca, Gastropoda, Sorbeoconcha, Potamididae, Terebralia|HE680668|Okinawa Pref., Kunigami-gun, Ginoza, Kanna R., Okinawa, Japan
Terebralia sulcata|Mollusca, Gastropoda, Sorbeoconcha, Potamididae, Terebralia|HE680663|Hong Kong, China
Terebralia sulcata|Mollusca, Gastropoda, Sorbeoconcha, Potamididae, Terebralia|HE680667|Okinawa Pref., Kunigami-gun, Ginoza, Kanna R., Okinawa, Japan
Terebralia sulcata|Mollusca, Gastropoda, Sorbeoconcha, Potamididae, Terebralia|HE680630|Okinawa Pref., Kunigami-gun, Ginoza, Kanna R., Okinawa, Japan
Terebralia sulcata|Mollusca, Gastropoda, Sorbeoconcha, Potamididae, Terebralia|HE680669|Okinawa Pref., Kunigami-gun, Ginoza, Kanna R., Okinawa, Japan
Terebralia sulcata|Mollusca, Gastropoda, Sorbeoconcha, Potamididae, Terebralia|HE680670|Okinawa, Haneji, Okinawa, Japan
Cerithidea rhizophorarum|Mollusca, Gastropoda, Sorbeoconcha, Potamididae, Cerithidea|HE680285|Shiokawa, Aichi Pref., Japan
Cerithidea rhizophorarum|Mollusca, Gastropoda, Sorbeoconcha, Potamididae, Cerithidea|HE680258|Haneji,Okinawa, Ryukyu Is, Okinawa, Japan
Cerithidea rhizophorarum|Mollusca, Gastropoda, Sorbeoconcha, Potamididae, Cerithidea|AM932774|Makiya, Okinawa, Japan
Cerithidea rhizophorarum|Mollusca, Gastropoda, Sorbeoconcha, Potamididae, Cerithidea|HE680274|Hiroshima Pref., Kasaoka Bay, Japan,
Cerithidea rhizophorarum|Mollusca, Gastropoda, Sorbeoconcha, Potamididae, Cerithidea|HE680262|Fukuoka Pref., Imazu Bay, Japan

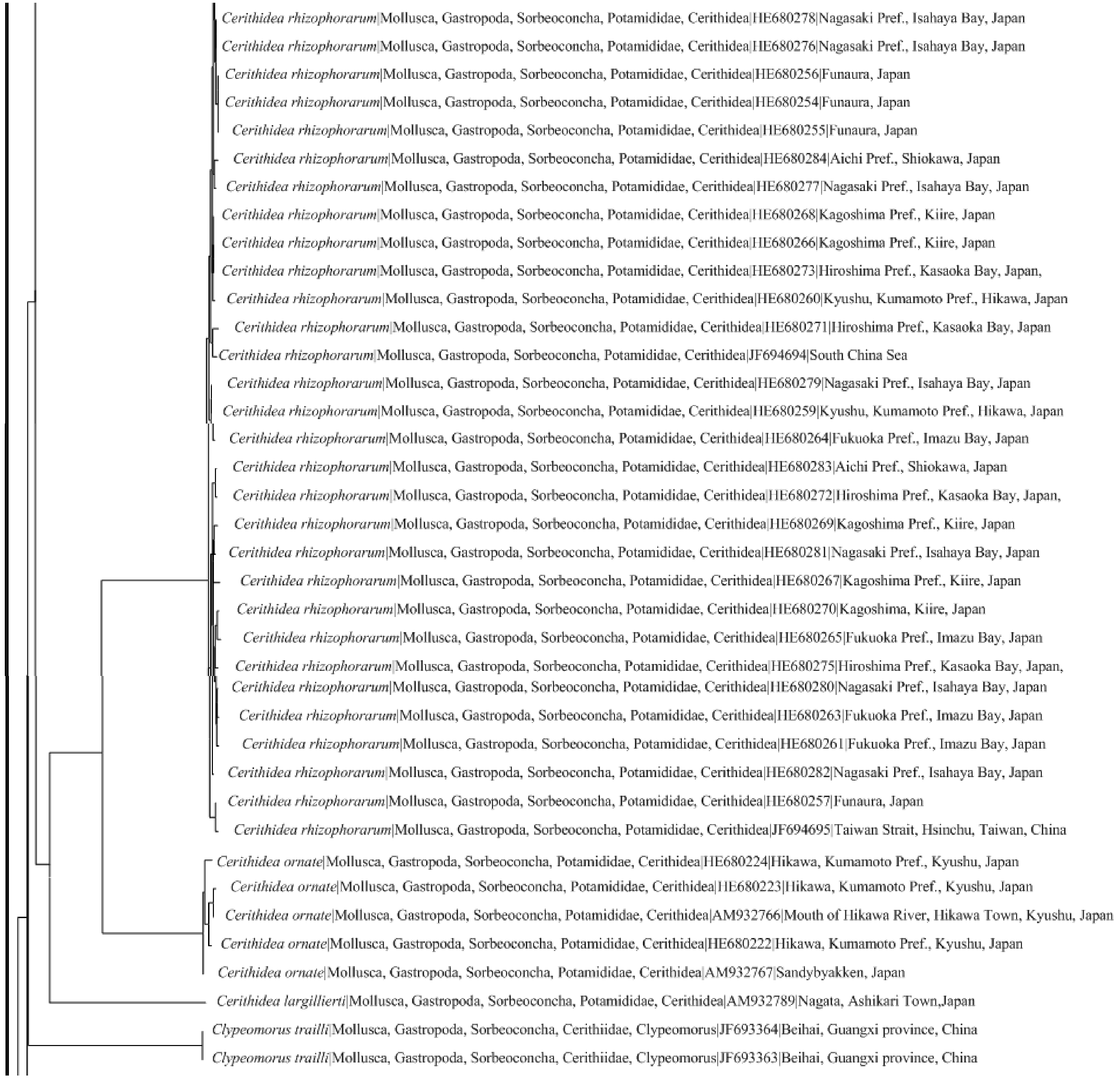
Cerithidea rhizophorarum|Mollusca, Gastropoda, Sorbeoconcha, Potamididae, Cerithidea|HE680278|Nagasaki Pref., Isahaya Bay, Japan
Cerithidea rhizophorarum|Mollusca, Gastropoda, Sorbeoconcha, Potamididae, Cerithidea|HE680276|Nagasaki Pref., Isahaya Bay, Japan
Cerithidea rhizophorarum|Mollusca, Gastropoda, Sorbeoconcha, Potamididae, Cerithidea|HE680256|Funaura, Japan
Cerithidea rhizophorarum|Mollusca, Gastropoda, Sorbeoconcha, Potamididae, Cerithidea|HE680254|Funaura, Japan
Cerithidea rhizophorarum|Mollusca, Gastropoda, Sorbeoconcha, Potamididae, Cerithidea|HE680255|Funaura, Japan
Cerithidea rhizophorarum|Mollusca, Gastropoda, Sorbeoconcha, Potamididae, Cerithidea|HE680284|Aichi Pref., Shiokawa, Japan
Cerithidea rhizophorarum|Mollusca, Gastropoda, Sorbeoconcha, Potamididae, Cerithidea|HE680277|Nagasaki Pref., Isahaya Bay, Japan
Cerithidea rhizophorarum|Mollusca, Gastropoda, Sorbeoconcha, Potamididae, Cerithidea|HE680268|Kagoshima Pref., Kiire, Japan
Cerithidea rhizophorarum|Mollusca, Gastropoda, Sorbeoconcha, Potamididae, Cerithidea|HE680266|Kagoshima Pref., Kiire, Japan
Cerithidea rhizophorarum|Mollusca, Gastropoda, Sorbeoconcha, Potamididae, Cerithidea|HE680273|Hiroshima Pref., Kasaoka Bay, Japan,
Cerithidea rhizophorarum|Mollusca, Gastropoda, Sorbeoconcha, Potamididae, Cerithidea|HE680260|Kyushu, Kumamoto Pref., Hikawa, Japan
Cerithidea rhizophorarum|Mollusca, Gastropoda, Sorbeoconcha, Potamididae, Cerithidea|HE680271|Hiroshima Pref., Kasaoka Bay, Japan
Cerithidea rhizophorarum|Mollusca, Gastropoda, Sorbeoconcha, Potamididae, Cerithidea|JF694694|South China Sea
Cerithidea rhizophorarum|Mollusca, Gastropoda, Sorbeoconcha, Potamididae, Cerithidea|HE680279|Nagasaki Pref., Isahaya Bay, Japan
Cerithidea rhizophorarum|Mollusca, Gastropoda, Sorbeoconcha, Potamididae, Cerithidea|HE680259|Kyushu, Kumamoto Pref., Hikawa, Japan
Cerithidea rhizophorarum|Mollusca, Gastropoda, Sorbeoconcha, Potamididae, Cerithidea|HE680264|Fukuoka Pref., Imazu Bay, Japan
Cerithidea rhizophorarum|Mollusca, Gastropoda, Sorbeoconcha, Potamididae, Cerithidea|HE680283|Aichi Pref., Shiokawa, Japan
Cerithidea rhizophorarum|Mollusca, Gastropoda, Sorbeoconcha, Potamididae, Cerithidea|HE680272|Hiroshima Pref., Kasaoka Bay, Japan,
Cerithidea rhizophorarum|Mollusca, Gastropoda, Sorbeoconcha, Potamididae, Cerithidea|HE680269|Kagoshima Pref., Kiire, Japan
Cerithidea rhizophorarum|Mollusca, Gastropoda, Sorbeoconcha, Potamididae, Cerithidea|HE680281|Nagasaki Pref., Isahaya Bay, Japan
Cerithidea rhizophorarum|Mollusca, Gastropoda, Sorbeoconcha, Potamididae, Cerithidea|HE680267|Kagoshima Pref., Kiire, Japan
Cerithidea rhizophorarum|Mollusca, Gastropoda, Sorbeoconcha, Potamididae, Cerithidea|HE680270|Kagoshima, Kiire, Japan
Cerithidea rhizophorarum|Mollusca, Gastropoda, Sorbeoconcha, Potamididae, Cerithidea|HE680265|Fukuoka Pref., Imazu Bay, Japan
Cerithidea rhizophorarum|Mollusca, Gastropoda, Sorbeoconcha, Potamididae, Cerithidea|HE680275|Hiroshima Pref., Kasaoka Bay, Japan,
Cerithidea rhizophorarum|Mollusca, Gastropoda, Sorbeoconcha, Potamididae, Cerithidea|HE680280|Nagasaki Pref., Isahaya Bay, Japan
Cerithidea rhizophorarum|Mollusca, Gastropoda, Sorbeoconcha, Potamididae, Cerithidea|HE680263|Fukuoka Pref., Imazu Bay, Japan
Cerithidea rhizophorarum|Mollusca, Gastropoda, Sorbeoconcha, Potamididae, Cerithidea|HE680261|Fukuoka Pref., Imazu Bay, Japan
Cerithidea rhizophorarum|Mollusca, Gastropoda, Sorbeoconcha, Potamididae, Cerithidea|HE680282|Nagasaki Pref., Isahaya Bay, Japan
Cerithidea rhizophorarum|Mollusca, Gastropoda, Sorbeoconcha, Potamididae, Cerithidea|HE680257|Funaura, Japan
Cerithidea rhizophorarum|Mollusca, Gastropoda, Sorbeoconcha, Potamididae, Cerithidea|JF694695|Taiwan Strait, Hsinchu, Taiwan, China
Cerithidea ornate|Mollusca, Gastropoda, Sorbeoconcha, Potamididae, Cerithidea|HE680224|Hikawa, Kumamoto Pref., Kyushu, Japan
Cerithidea ornate|Mollusca, Gastropoda, Sorbeoconcha, Potamididae, Cerithidea|HE680223|Hikawa, Kumamoto Pref., Kyushu, Japan
Cerithidea ornate|Mollusca, Gastropoda, Sorbeoconcha, Potamididae, Cerithidea|AM932766|Mouth of Hikawa River, Hikawa Town, Kyushu, Japan
Cerithidea ornate|Mollusca, Gastropoda, Sorbeoconcha, Potamididae, Cerithidea|HE680222|Hikawa, Kumamoto Pref., Kyushu, Japan
Cerithidea ornate|Mollusca, Gastropoda, Sorbeoconcha, Potamididae, Cerithidea|AM932767|Sandybyakken, Japan
Cerithidea largillierti|Mollusca, Gastropoda, Sorbeoconcha, Potamididae, Cerithidea|AM932789|Nagata, Ashikari Town,Japan
Clypeomorus trailli|Mollusca, Gastropoda, Sorbeoconcha, Cerithiidae, Clypeomorus|JF693364|Beihai, Guangxi province, China
Clypeomorus trailli|Mollusca, Gastropoda, Sorbeoconcha, Cerithiidae, Clypeomorus|JF693363|Beihai, Guangxi province, China

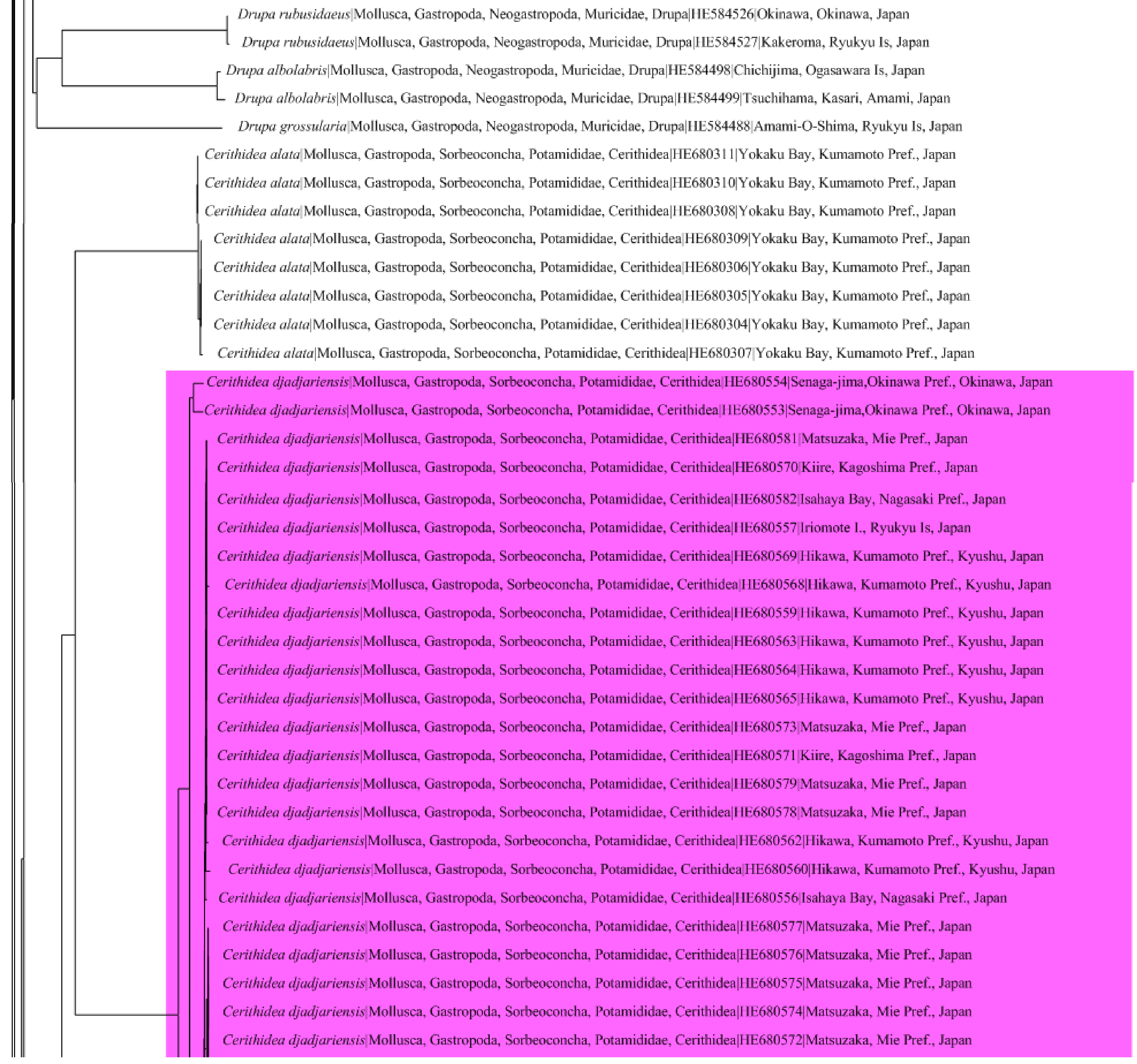

Drupa rubusidaeus|Mollusca, Gastropoda, Neogastropoda, Muricidae, Drupa|HE584526|Okinawa, Okinawa, Japan
Drupa rubusidaeus|Mollusca, Gastropoda, Neogastropoda, Muricidae, Drupa|HE584527|Kakeroma, Ryukyu Is, Japan
Drupa albolabris|Mollusca, Gastropoda, Neogastropoda, Muricidae, Drupa|HE584498|Chichijima, Ogasawara Is, Japan
Drupa albolabris|Mollusca, Gastropoda, Neogastropoda, Muricidae, Drupa|HE584499|Tsuchihama, Kasari, Amami, Japan
Drupa grossularia|Mollusca, Gastropoda, Neogastropoda, Muricidae, Drupa|HE584488|Amami-O-Shima, Ryukyu Is, Japan
Cerithidea alata|Mollusca, Gastropoda, Sorbeoconcha, Potamididae, Cerithidea|HE680311|Yokaku Bay, Kumamoto Pref., Japan
Cerithidea alata|Mollusca, Gastropoda, Sorbeoconcha, Potamididae, Cerithidea|HE680310|Yokaku Bay, Kumamoto Pref., Japan
Cerithidea alata|Mollusca, Gastropoda, Sorbeoconcha, Potamididae, Cerithidea|HE680308|Yokaku Bay, Kumamoto Pref., Japan
Cerithidea alata|Mollusca, Gastropoda, Sorbeoconcha, Potamididae, Cerithidea|HE680309|Yokaku Bay, Kumamoto Pref., Japan
Cerithidea alata|Mollusca, Gastropoda, Sorbeoconcha, Potamididae, Cerithidea|HE680306|Yokaku Bay, Kumamoto Pref., Japan
Cerithidea alata|Mollusca, Gastropoda, Sorbeoconcha, Potamididae, Cerithidea|HE680305|Yokaku Bay, Kumamoto Pref., Japan
Cerithidea alata|Mollusca, Gastropoda, Sorbeoconcha, Potamididae, Cerithidea|HE680304|Yokaku Bay, Kumamoto Pref., Japan
Cerithidea alata|Mollusca, Gastropoda, Sorbeoconcha, Potamididae, Cerithidea|HE680307|Yokaku Bay, Kumamoto Pref., Japan
Cerithidea djadjariensis|Mollusca, Gastropoda, Sorbeoconcha, Potamididae, Cerithidea|HE680554|Senaga-jima,Okinawa Pref., Okinawa, Japan
Cerithidea djadjariensis|Mollusca, Gastropoda, Sorbeoconcha, Potamididae, Cerithidea|HE680553|Senaga-jima,Okinawa Pref., Okinawa, Japan
Cerithidea djadjariensis|Mollusca, Gastropoda, Sorbeoconcha, Potamididae, Cerithidea|HE680581|Matsuzaka, Mie Pref., Japan
Cerithidea djadjariensis|Mollusca, Gastropoda, Sorbeoconcha, Potamididae, Cerithidea|HE680570|Kiire, Kagoshima Pref., Japan
Cerithidea djadjariensis|Mollusca, Gastropoda, Sorbeoconcha, Potamididae, Cerithidea|HE680582|Isahaya Bay, Nagasaki Pref., Japan
Cerithidea djadjariensis|Mollusca, Gastropoda, Sorbeoconcha, Potamididae, Cerithidea|HE680557|Iriomote I., Ryukyu Is, Japan
Cerithidea djadjariensis|Mollusca, Gastropoda, Sorbeoconcha, Potamididae, Cerithidea|HE680569|Hikawa, Kumamoto Pref., Kyushu, Japan
Cerithidea djadjariensis|Mollusca, Gastropoda, Sorbeoconcha, Potamididae, Cerithidea|HE680568|Hikawa, Kumamoto Pref., Kyushu, Japan
Cerithidea djadjariensis|Mollusca, Gastropoda, Sorbeoconcha, Potamididae, Cerithidea|HE680559|Hikawa, Kumamoto Pref., Kyushu, Japan
Cerithidea djadjariensis|Mollusca, Gastropoda, Sorbeoconcha, Potamididae, Cerithidea|HE680563|Hikawa, Kumamoto Pref., Kyushu, Japan
Cerithidea djadjariensis|Mollusca, Gastropoda, Sorbeoconcha, Potamididae, Cerithidea|HE680564|Hikawa, Kumamoto Pref., Kyushu, Japan
Cerithidea djadjariensis|Mollusca, Gastropoda, Sorbeoconcha, Potamididae, Cerithidea|HE680565|Hikawa, Kumamoto Pref., Kyushu, Japan
Cerithidea djadjariensis|Mollusca, Gastropoda, Sorbeoconcha, Potamididae, Cerithidea|HE680573|Matsuzaka, Mie Pref., Japan
Cerithidea djadjariensis|Mollusca, Gastropoda, Sorbeoconcha, Potamididae, Cerithidea|HE680571|Kiire, Kagoshima Pref., Japan
Cerithidea djadjariensis|Mollusca, Gastropoda, Sorbeoconcha, Potamididae, Cerithidea|HE680579|Matsuzaka, Mie Pref., Japan
Cerithidea djadjariensis|Mollusca, Gastropoda, Sorbeoconcha, Potamididae, Cerithidea|HE680578|Matsuzaka, Mie Pref., Japan
Cerithidea djadjariensis|Mollusca, Gastropoda, Sorbeoconcha, Potamididae, Cerithidea|HE680562|Hikawa, Kumamoto Pref., Kyushu, Japan
Cerithidea djadjariensis|Mollusca, Gastropoda, Sorbeoconcha, Potamididae, Cerithidea|HE680560|Hikawa, Kumamoto Pref., Kyushu, Japan
Cerithidea djadjariensis|Mollusca, Gastropoda, Sorbeoconcha, Potamididae, Cerithidea|HE680556|Isahaya Bay, Nagasaki Pref., Japan
Cerithidea djadjariensis|Mollusca, Gastropoda, Sorbeoconcha, Potamididae, Cerithidea|HE680577|Matsuzaka, Mie Pref., Japan
Cerithidea djadjariensis|Mollusca, Gastropoda, Sorbeoconcha, Potamididae, Cerithidea|HE680576|Matsuzaka, Mie Pref., Japan
Cerithidea djadjariensis|Mollusca, Gastropoda, Sorbeoconcha, Potamididae, Cerithidea|HE680575|Matsuzaka, Mie Pref., Japan
Cerithidea djadjariensis|Mollusca, Gastropoda, Sorbeoconcha, Potamididae, Cerithidea|HE680574|Matsuzaka, Mie Pref., Japan
Cerithidea djadjariensis|Mollusca, Gastropoda, Sorbeoconcha, Potamididae, Cerithidea|HE680572|Matsuzaka, Mie Pref., Japan

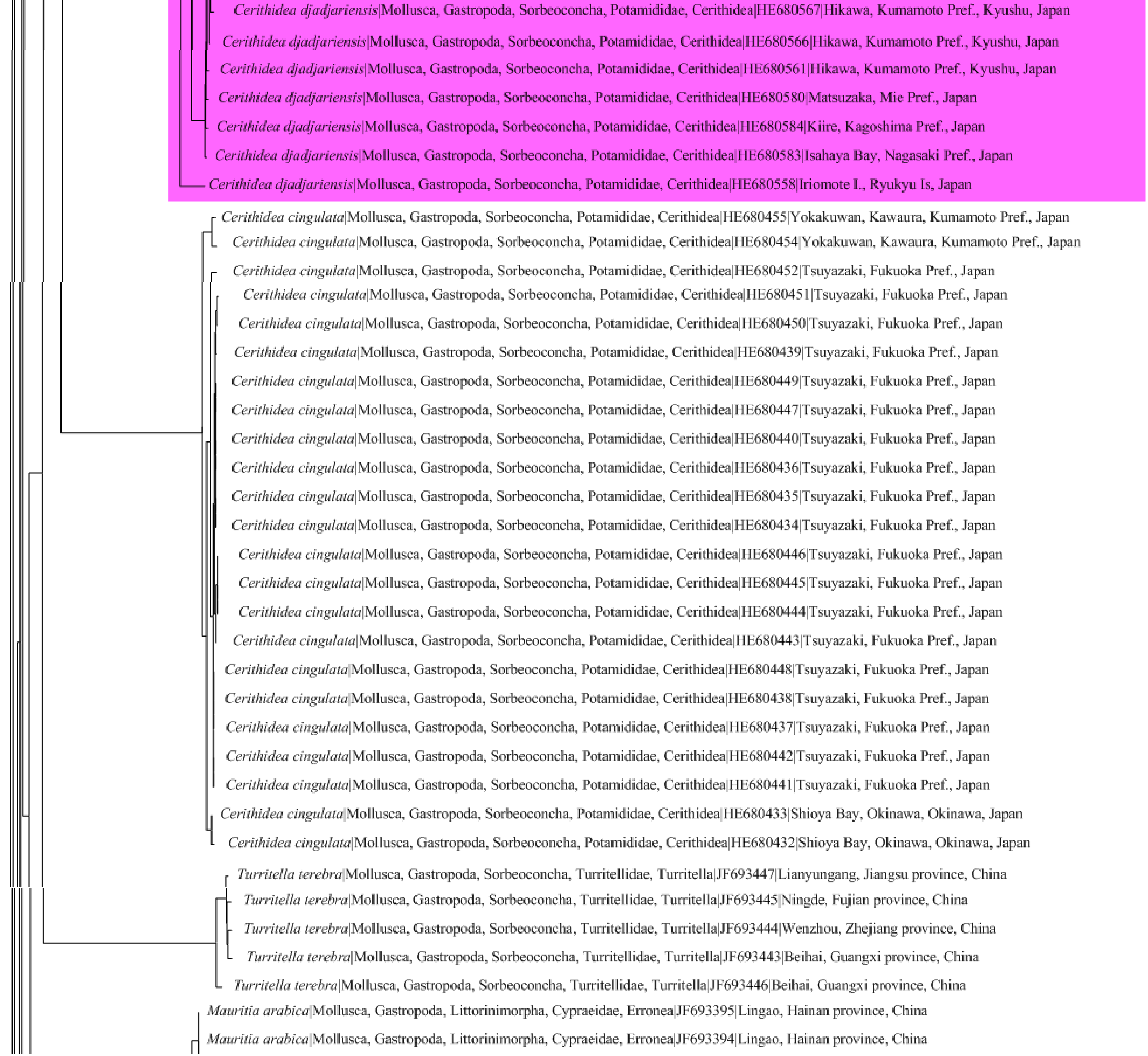

Cerithidea djadjariensis|Mollusca, Gastropoda, Sorbeoconcha, Potamididae, Cerithidea|HE680567|Hikawa, Kumamoto Pref., Kyushu, Japan
Cerithidea djadjariensis|Mollusca, Gastropoda, Sorbeoconcha, Potamididae, Cerithidea|HE680566|Hikawa, Kumamoto Pref., Kyushu, Japan
Cerithidea djadjariensis|Mollusca, Gastropoda, Sorbeoconcha, Potamididae, Cerithidea|HE680561|Hikawa, Kumamoto Pref., Kyushu, Japan
Cerithidea djadjariensis|Mollusca, Gastropoda, Sorbeoconcha, Potamididae, Cerithidea|HE680580|Matsuzaka, Mie Pref., Japan
Cerithidea djadjariensis|Mollusca, Gastropoda, Sorbeoconcha, Potamididae, Cerithidea|HE680584|Kiire, Kagoshima Pref., Japan
Cerithidea djadjariensis|Mollusca, Gastropoda, Sorbeoconcha, Potamididae, Cerithidea|HE680583|Isahaya Bay, Nagasaki Pref., Japan
Cerithidea djadjariensis|Mollusca, Gastropoda, Sorbeoconcha, Potamididae, Cerithidea|HE680558|Iriomote I., Ryukyu Is, Japan
Cerithidea cingulata|Mollusca, Gastropoda, Sorbeoconcha, Potamididae, Cerithidea|HE680455|Yokakuwan, Kawaura, Kumamoto Pref., Japan
Cerithidea cingulata|Mollusca, Gastropoda, Sorbeoconcha, Potamididae, Cerithidea|HE680454|Yokakuwan, Kawaura, Kumamoto Pref., Japan
Cerithidea cingulata|Mollusca, Gastropoda, Sorbeoconcha, Potamididae, Cerithidea|HE680452|Tsuyazaki, Fukuoka Pref., Japan
Cerithidea cingulata|Mollusca, Gastropoda, Sorbeoconcha, Potamididae, Cerithidea|HE680451|Tsuyazaki, Fukuoka Pref., Japan
Cerithidea cingulata|Mollusca, Gastropoda, Sorbeoconcha, Potamididae, Cerithidea|HE680450|Tsuyazaki, Fukuoka Pref., Japan
Cerithidea cingulata|Mollusca, Gastropoda, Sorbeoconcha, Potamididae, Cerithidea|HE680439|Tsuyazaki, Fukuoka Pref., Japan
Cerithidea cingulata|Mollusca, Gastropoda, Sorbeoconcha, Potamididae, Cerithidea|HE680449|Tsuyazaki, Fukuoka Pref., Japan
Cerithidea cingulata|Mollusca, Gastropoda, Sorbeoconcha, Potamididae, Cerithidea|HE680447|Tsuyazaki, Fukuoka Pref., Japan
Cerithidea cingulata|Mollusca, Gastropoda, Sorbeoconcha, Potamididae, Cerithidea|HE680440|Tsuyazaki, Fukuoka Pref., Japan
Cerithidea cingulata|Mollusca, Gastropoda, Sorbeoconcha, Potamididae, Cerithidea|HE680436|Tsuyazaki, Fukuoka Pref., Japan
Cerithidea cingulata|Mollusca, Gastropoda, Sorbeoconcha, Potamididae, Cerithidea|HE680435|Tsuyazaki, Fukuoka Pref., Japan
Cerithidea cingulata|Mollusca, Gastropoda, Sorbeoconcha, Potamididae, Cerithidea|HE680434|Tsuyazaki, Fukuoka Pref., Japan
Cerithidea cingulata|Mollusca, Gastropoda, Sorbeoconcha, Potamididae, Cerithidea|HE680446|Tsuyazaki, Fukuoka Pref., Japan
Cerithidea cingulata|Mollusca, Gastropoda, Sorbeoconcha, Potamididae, Cerithidea|HE680445|Tsuyazaki, Fukuoka Pref., Japan
Cerithidea cingulata|Mollusca, Gastropoda, Sorbeoconcha, Potamididae, Cerithidea|HE680444|Tsuyazaki, Fukuoka Pref., Japan
Cerithidea cingulata|Mollusca, Gastropoda, Sorbeoconcha, Potamididae, Cerithidea|HE680443|Tsuyazaki, Fukuoka Pref., Japan
Cerithidea cingulata|Mollusca, Gastropoda, Sorbeoconcha, Potamididae, Cerithidea|HE680448|Tsuyazaki, Fukuoka Pref., Japan
Cerithidea cingulata|Mollusca, Gastropoda, Sorbeoconcha, Potamididae, Cerithidea|HE680438|Tsuyazaki, Fukuoka Pref., Japan
Cerithidea cingulata|Mollusca, Gastropoda, Sorbeoconcha, Potamididae, Cerithidea|HE680437|Tsuyazaki, Fukuoka Pref., Japan
Cerithidea cingulata|Mollusca, Gastropoda, Sorbeoconcha, Potamididae, Cerithidea|HE680442|Tsuyazaki, Fukuoka Pref., Japan
Cerithidea cingulata|Mollusca, Gastropoda, Sorbeoconcha, Potamididae, Cerithidea|HE680441|Tsuyazaki, Fukuoka Pref., Japan
Cerithidea cingulata|Mollusca, Gastropoda, Sorbeoconcha, Potamididae, Cerithidea|HE680433|Shioya Bay, Okinawa, Okinawa, Japan
Cerithidea cingulata|Mollusca, Gastropoda, Sorbeoconcha, Potamididae, Cerithidea|HE680432|Shioya Bay, Okinawa, Okinawa, Japan
Turritella terebra|Mollusca, Gastropoda, Sorbeoconcha, Turritellidae, Turritella|JF693447|Lianyungang, Jiangsu province, China
Turritella terebra|Mollusca, Gastropoda, Sorbeoconcha, Turritellidae, Turritella|JF693445|Ningde, Fujian province, China
Turritella terebra|Mollusca, Gastropoda, Sorbeoconcha, Turritellidae, Turritella|JF693444|Wenzhou, Zhejiang province, China
Turritella terebra|Mollusca, Gastropoda, Sorbeoconcha, Turritellidae, Turritella|JF693443|Beihai, Guangxi province, China
Turritella terebra|Mollusca, Gastropoda, Sorbeoconcha, Turritellidae, Turritella|JF693446|Beihai, Guangxi province, China
Mauritia arabica|Mollusca, Gastropoda, Littorinimorpha, Cypraeidae, Erronea|JF693395|Lingao, Hainan province, China
Mauritia arabica|Mollusca, Gastropoda, Littorinimorpha, Cypraeidae, Erronea|JF693394|Lingao, Hainan province, China

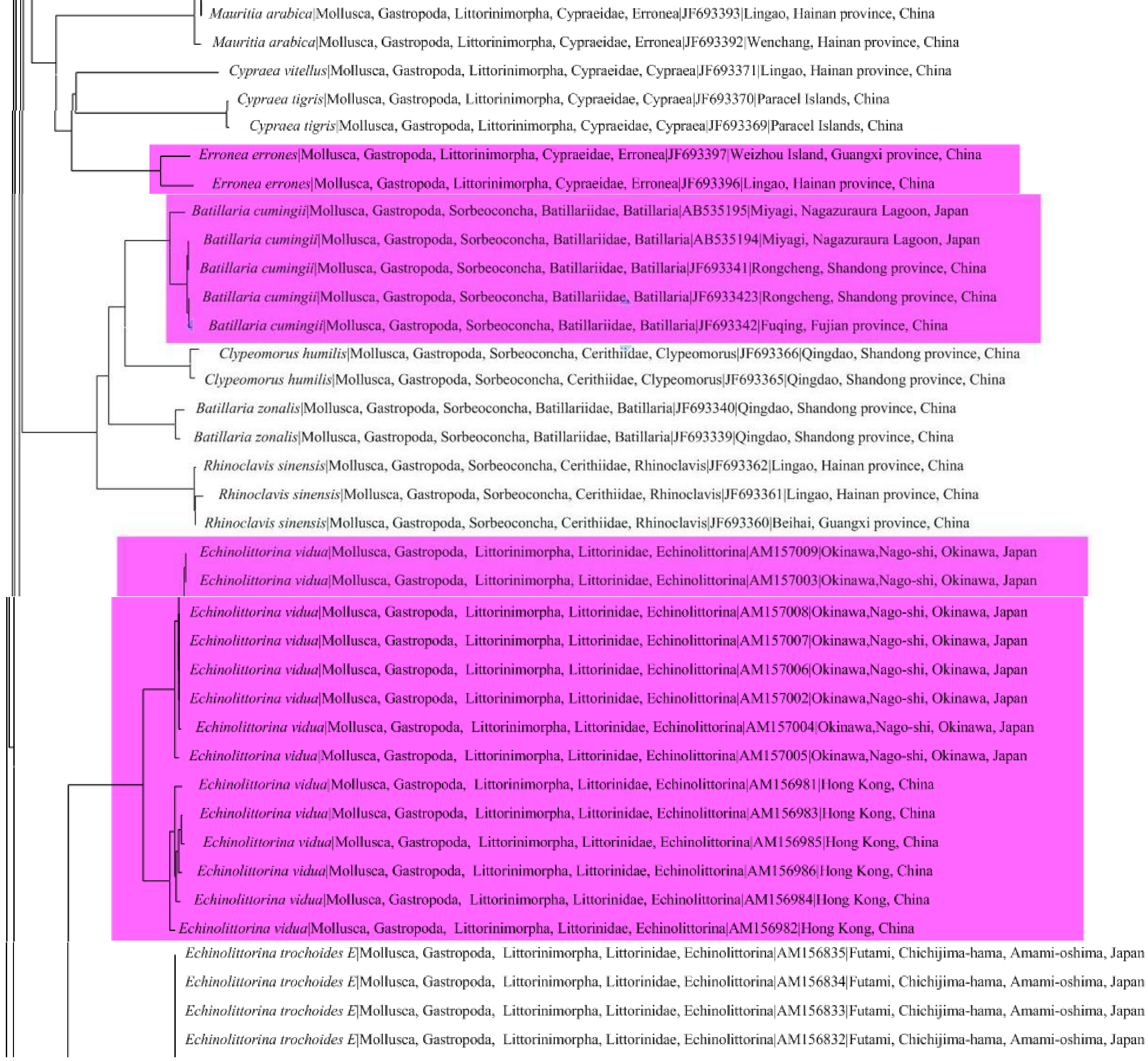
Mauritia arabica|Mollusca, Gastropoda, Littorinimorpha, Cypraeidae, Erronea|JF693393|Lingao, Hainan province, China
Mauritia arabica|Mollusca, Gastropoda, Littorinimorpha, Cypraeidae, Erronea|JF693392|Wenchang, Hainan province, China
Cypraea vitellus|Mollusca, Gastropoda, Littorinimorpha, Cypraeidae, Cypraea|JF693371|Lingao, Hainan province, China
Cypraea tigris|Mollusca, Gastropoda, Littorinimorpha, Cypraeidae, Cypraea|JF693370|Paracel Islands, China
Cypraea tigris|Mollusca, Gastropoda, Littorinimorpha, Cypraeidae, Cypraea|JF693369|Paracel Islands, China
Erronea errones|Mollusca, Gastropoda, Littorinimorpha, Cypraeidae, Erronea|JF693397|Weizhou Island, Guangxi province, China
Erronea errones|Mollusca, Gastropoda, Littorinimorpha, Cypraeidae, Erronea|JF693396|Lingao, Hainan province, China
Batillaria cumingii|Mollusca, Gastropoda, Sorbeoconcha, Batillariidae, Batillaria|AB535195|Miyagi, Nagazuraura Lagoon, Japan
Batillaria cumingii|Mollusca, Gastropoda, Sorbeoconcha, Batillariidae, Batillaria|AB535194|Miyagi, Nagazuraura Lagoon, Japan
Batillaria cumingii|Mollusca, Gastropoda, Sorbeoconcha, Batillariidae, Batillaria|JF693341|Rongcheng, Shandong province, China
Batillaria cumingii|Mollusca, Gastropoda, Sorbeoconcha, Batillariidae, Batillaria|JF6933423|Rongcheng, Shandong province, China
Batillaria cumingii|Mollusca, Gastropoda, Sorbeoconcha, Batillariidae, Batillaria|JF693342|Fuqing, Fujian province, China
Clypeomorus humilis|Mollusca, Gastropoda, Sorbeoconcha, Cerithiidae, Clypeomorus|JF693366|Qingdao, Shandong province, China
Clypeomorus humilis|Mollusca, Gastropoda, Sorbeoconcha, Cerithiidae, Clypeomorus|JF693365|Qingdao, Shandong province, China
Batillaria zonalis|Mollusca, Gastropoda, Sorbeoconcha, Batillariidae, Batillaria|JF693340|Qingdao, Shandong province, China
Batillaria zonalis|Mollusca, Gastropoda, Sorbeoconcha, Batillariidae, Batillaria|JF693339|Qingdao, Shandong province, China
Rhinoclavis sinensis|Mollusca, Gastropoda, Sorbeoconcha, Cerithiidae, Rhinoclavis|JF693362|Lingao, Hainan province, China
Rhinoclavis sinensis|Mollusca, Gastropoda, Sorbeoconcha, Cerithiidae, Rhinoclavis|JF693361|Lingao, Hainan province, China
Rhinoclavis sinensis|Mollusca, Gastropoda, Sorbeoconcha, Cerithiidae, Rhinoclavis|JF693360|Beihai, Guangxi province, China
Echinolittorina vidua|Mollusca, Gastropoda, Littorinimorpha, Littorinidae, Echinolittorina|AM157009|Okinawa,Nago-shi, Okinawa, Japan
Echinolittorina vidua|Mollusca, Gastropoda, Littorinimorpha, Littorinidae, Echinolittorina|AM157003|Okinawa,Nago-shi, Okinawa, Japan
Echinolittorina vidua|Mollusca, Gastropoda, Littorinimorpha, Littorinidae, Echinolittorina|AM157008|Okinawa,Nago-shi, Okinawa, Japan
Echinolittorina vidua|Mollusca, Gastropoda, Littorinimorpha, Littorinidae, Echinolittorina|AM157007|Okinawa,Nago-shi, Okinawa, Japan
Echinolittorina vidua|Mollusca, Gastropoda, Littorinimorpha, Littorinidae, Echinolittorina|AM157006|Okinawa,Nago-shi, Okinawa, Japan
Echinolittorina vidua|Mollusca, Gastropoda, Littorinimorpha, Littorinidae, Echinolittorina|AM157002|Okinawa,Nago-shi, Okinawa, Japan
Echinolittorina vidua|Mollusca, Gastropoda, Littorinimorpha, Littorinidae, Echinolittorina|AM157004|Okinawa,Nago-shi, Okinawa, Japan
Echinolittorina vidua|Mollusca, Gastropoda, Littorinimorpha, Littorinidae, Echinolittorina|AM157005|Okinawa,Nago-shi, Okinawa, Japan
Echinolittorina vidua|Mollusca, Gastropoda, Littorinimorpha, Littorinidae, Echinolittorina|AM156981|Hong Kong, China
Echinolittorina vidua|Mollusca, Gastropoda, Littorinimorpha, Littorinidae, Echinolittorina|AM156983|Hong Kong, China
Echinolittorina vidua|Mollusca, Gastropoda, Littorinimorpha, Littorinidae, Echinolittorina|AM156985|Hong Kong, China
Echinolittorina vidua|Mollusca, Gastropoda, Littorinimorpha, Littorinidae, Echinolittorina|AM156986|Hong Kong, China
Echinolittorina vidua|Mollusca, Gastropoda, Littorinimorpha, Littorinidae, Echinolittorina|AM156984|Hong Kong, China
Echinolittorina vidua|Mollusca, Gastropoda, Littorinimorpha, Littorinidae, Echinolittorina|AM156982|Hong Kong, China
Echinolittorina trochoides E|Mollusca, Gastropoda, Littorinimorpha, Littorinidae, Echinolittorina|AM156835|Futami, Chichijima-hama, Amami-oshima, Japan
Echinolittorina trochoides E|Mollusca, Gastropoda, Littorinimorpha, Littorinidae, Echinolittorina|AM156834|Futami, Chichijima-hama, Amami-oshima, Japan
Echinolittorina trochoides E|Mollusca, Gastropoda, Littorinimorpha, Littorinidae, Echinolittorina|AM156833|Futami, Chichijima-hama, Amami-oshima, Japan
Echinolittorina trochoides E|Mollusca, Gastropoda, Littorinimorpha, Littorinidae, Echinolittorina|AM156832|Futami, Chichijima-hama, Amami-oshima, Japan

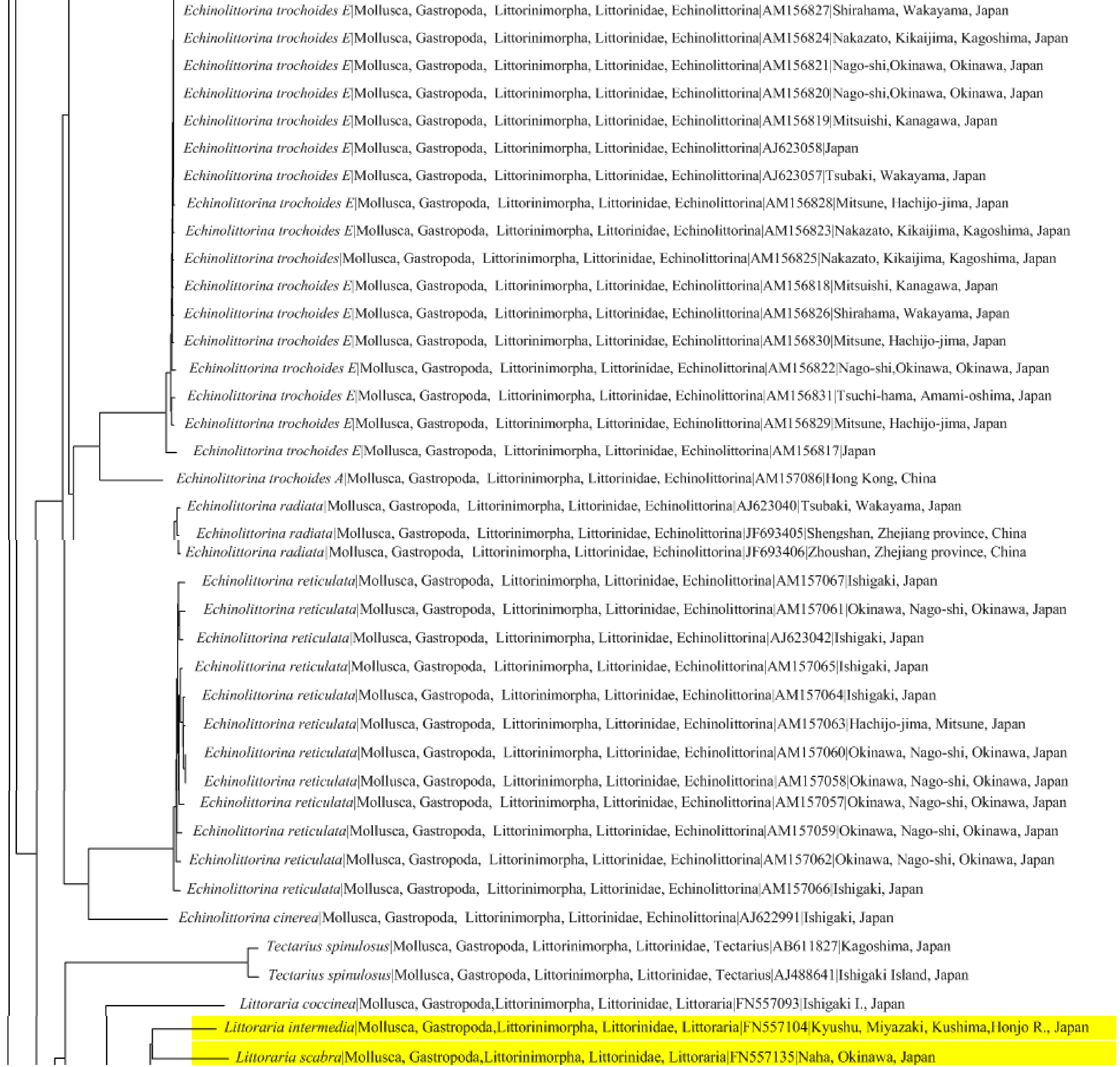
Echinolittorina trochoides E|Mollusca, Gastropoda, Littorinimorpha, Littorinidae, Echinolittorina|AM156827|Shirahama, Wakayama, Japan
Echinolittorina trochoides E|Mollusca, Gastropoda, Littorinimorpha, Littorinidae, Echinolittorina|AM156824|Nakazato, Kikaijima, Kagoshima, Japan
Echinolittorina trochoides E|Mollusca, Gastropoda, Littorinimorpha, Littorinidae, Echinolittorina|AM156821|Nago-shi,Okinawa, Okinawa, Japan
Echinolittorina trochoides E|Mollusca, Gastropoda, Littorinimorpha, Littorinidae, Echinolittorina|AM156820|Nago-shi,Okinawa, Okinawa, Japan
Echinolittorina trochoides E|Mollusca, Gastropoda, Littorinimorpha, Littorinidae, Echinolittorina|AM156819|Mitsuishi, Kanagawa, Japan
Echinolittorina trochoides E|Mollusca, Gastropoda, Littorinimorpha, Littorinidae, Echinolittorina|AJ623058|Japan
Echinolittorina trochoides E|Mollusca, Gastropoda, Littorinimorpha, Littorinidae, Echinolittorina|AJ623057|Tsubaki, Wakayama, Japan
Echinolittorina trochoides E|Mollusca, Gastropoda, Littorinimorpha, Littorinidae, Echinolittorina|AM156828|Mitsune, Hachijo-jima, Japan
Echinolittorina trochoides E|Mollusca, Gastropoda, Littorinimorpha, Littorinidae, Echinolittorina|AM156823|Nakazato, Kikaijima, Kagoshima, Japan
Echinolittorina trochoides|Mollusca, Gastropoda, Littorinimorpha, Littorinidae, Echinolittorina|AM156825|Nakazato, Kikaijima, Kagoshima, Japan
Echinolittorina trochoides E|Mollusca, Gastropoda, Littorinimorpha, Littorinidae, Echinolittorina|AM156818|Mitsuishi, Kanagawa, Japan
Echinolittorina trochoides E|Mollusca, Gastropoda, Littorinimorpha, Littorinidae, Echinolittorina|AM156826|Shirahama, Wakayama, Japan
Echinolittorina trochoides E|Mollusca, Gastropoda, Littorinimorpha, Littorinidae, Echinolittorina|AM156830|Mitsune, Hachijo-jima, Japan
Echinolittorina trochoides E|Mollusca, Gastropoda, Littorinimorpha, Littorinidae, Echinolittorina|AM156822|Nago-shi,Okinawa, Okinawa, Japan
Echinolittorina trochoides E|Mollusca, Gastropoda, Littorinimorpha, Littorinidae, Echinolittorina|AM156831|Tsuchi-hama, Amami-oshima, Japan
Echinolittorina trochoides E|Mollusca, Gastropoda, Littorinimorpha, Littorinidae, Echinolittorina|AM156829|Mitsune, Hachijo-jima, Japan
Echinolittorina trochoides E|Mollusca, Gastropoda, Littorinimorpha, Littorinidae, Echinolittorina|AM156817|Japan
Echinolittorina trochoides A|Mollusca, Gastropoda, Littorinimorpha, Littorinidae, Echinolittorina|AM157086|Hong Kong, China
Echinolittorina radiata|Mollusca, Gastropoda, Littorinimorpha, Littorinidae, Echinolittorina|AJ623040|Tsubaki, Wakayama, Japan
Echinolittorina radiata|Mollusca, Gastropoda, Littorinimorpha, Littorinidae, Echinolittorina|JF693405|Shengshan, Zhejiang province, China
Echinolittorina radiata|Mollusca, Gastropoda, Littorinimorpha, Littorinidae, Echinolittorina|JF693406|Zhoushan, Zhejiang province, China
Echinolittorina reticulata|Mollusca, Gastropoda, Littorinimorpha, Littorinidae, Echinolittorina|AM157067|Ishigaki, Japan
Echinolittorina reticulata|Mollusca, Gastropoda, Littorinimorpha, Littorinidae, Echinolittorina|AM157061|Okinawa, Nago-shi, Okinawa, Japan
Echinolittorina reticulata|Mollusca, Gastropoda, Littorinimorpha, Littorinidae, Echinolittorina|AJ623042|Ishigaki, Japan
Echinolittorina reticulata|Mollusca, Gastropoda, Littorinimorpha, Littorinidae, Echinolittorina|AM157065|Ishigaki, Japan
Echinolittorina reticulata|Mollusca, Gastropoda, Littorinimorpha, Littorinidae, Echinolittorina|AM157064|Ishigaki, Japan
Echinolittorina reticulata|Mollusca, Gastropoda, Littorinimorpha, Littorinidae, Echinolittorina|AM157063|Hachijo-jima, Mitsune, Japan
Echinolittorina reticulata|Mollusca, Gastropoda, Littorinimorpha, Littorinidae, Echinolittorina|AM157060|Okinawa, Nago-shi, Okinawa, Japan
Echinolittorina reticulata|Mollusca, Gastropoda, Littorinimorpha, Littorinidae, Echinolittorina|AM157058|Okinawa, Nago-shi, Okinawa, Japan
Echinolittorina reticulata|Mollusca, Gastropoda, Littorinimorpha, Littorinidae, Echinolittorina|AM157057|Okinawa, Nago-shi, Okinawa, Japan
Echinolittorina reticulata|Mollusca, Gastropoda, Littorinimorpha, Littorinidae, Echinolittorina|AM157059|Okinawa, Nago-shi, Okinawa, Japan
Echinolittorina reticulata|Mollusca, Gastropoda, Littorinimorpha, Littorinidae, Echinolittorina|AM157062|Okinawa, Nago-shi, Okinawa, Japan
Echinolittorina reticulata|Mollusca, Gastropoda, Littorinimorpha, Littorinidae, Echinolittorina|AM157066|Ishigaki, Japan
Echinolittorina cinerea|Mollusca, Gastropoda, Littorinimorpha, Littorinidae, Echinolittorina|AJ622991|Ishigaki, Japan
Tectarius spinulosus|Mollusca, Gastropoda, Littorinimorpha, Littorinidae, Tectarius|AB611827|Kagoshima, Japan
Tectarius spinulosus|Mollusca, Gastropoda, Littorinimorpha, Littorinidae, Tectarius|AJ488641|Ishigaki Island, Japan
Littoraria coccinea|Mollusca, Gastropoda,Littorinimorpha, Littorinidae, Littoraria|FN557093|Ishigaki I., Japan
Littoraria intermedia|Mollusca, Gastropoda,Littorinimorpha, Littorinidae, Littoraria|FN557104|Kyushu, Miyazaki, Kushima,Honjo R., Japan
Littoraria scabra|Mollusca, Gastropoda,Littorinimorpha, Littorinidae, Littoraria|FN557135|Naha, Okinawa, Japan

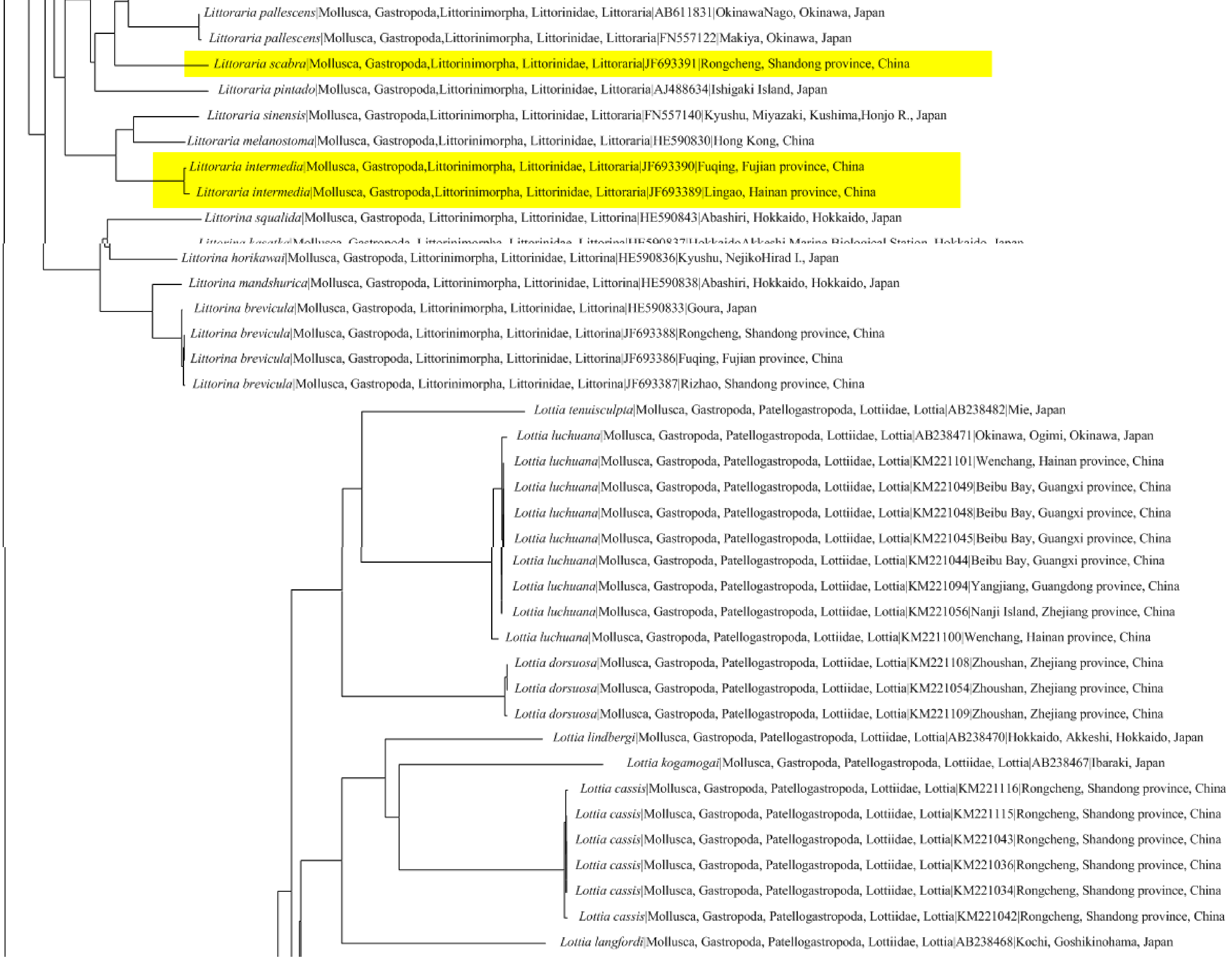
Littoraria pallescens|Mollusca, Gastropoda,Littorinimorpha, Littorinidae, Littoraria|AB611831|OkinawaNago, Okinawa, Japan
Littoraria pallescens|Mollusca, Gastropoda,Littorinimorpha, Littorinidae, Littoraria|FN557122|Makiya, Okinawa, Japan
Littoraria scabra|Mollusca, Gastropoda,Littorinimorpha, Littorinidae, Littoraria|JF693391|Rongcheng, Shandong province, China
Littoraria pintado|Mollusca, Gastropoda,Littorinimorpha, Littorinidae, Littoraria|AJ488634|Ishigaki Island, Japan
Littoraria sinensis|Mollusca, Gastropoda,Littorinimorpha, Littorinidae, Littoraria|FN557140|Kyushu, Miyazaki, Kushima,Honjo R., Japan
Littoraria melanostoma|Mollusca, Gastropoda,Littorinimorpha, Littorinidae, Littoraria|HE590830|Hong Kong, China
Littoraria intermedia|Mollusca, Gastropoda,Littorinimorpha, Littorinidae, Littoraria|JF693390|Fuqing, Fujian province, China
Littoraria intermedia|Mollusca, Gastropoda,Littorinimorpha, Littorinidae, Littoraria|JF693389|Lingao, Hainan province, China
Littorina squalida|Mollusca, Gastropoda, Littorinimorpha, Littorinidae, Littorina|HE590843|Abashiri, Hokkaido, Hokkaido, Japan
Littorina horikawai|Mollusca, Gastropoda, Littorinimorpha, Littorinidae, Littorina|HE590836|Kyushu, NejikoHirad I., Japan
Littorina mandshurica|Mollusca, Gastropoda, Littorinimorpha, Littorinidae, Littorina|HE590838|Abashiri, Hokkaido, Hokkaido, Japan
Littorina brevicula|Mollusca, Gastropoda, Littorinimorpha, Littorinidae, Littorina|HE590833|Goura, Japan
Littorina brevicula|Mollusca, Gastropoda, Littorinimorpha, Littorinidae, Littorina|JF693388|Rongcheng, Shandong province, China
Littorina brevicula|Mollusca, Gastropoda, Littorinimorpha, Littorinidae, Littorina|JF693386|Fuqing, Fujian province, China
Littorina brevicula|Mollusca, Gastropoda, Littorinimorpha, Littorinidae, Littorina|JF693387|Rizhao, Shandong province, China
Lottia tenuisculpta|Mollusca, Gastropoda, Patellogastropoda, Lottiidae, Lottia|AB238482|Mie, Japan
Lottia luchuana|Mollusca, Gastropoda, Patellogastropoda, Lottiidae, Lottia|AB238471|Okinawa, Ogimi, Okinawa, Japan
Lottia luchuana|Mollusca, Gastropoda, Patellogastropoda, Lottiidae, Lottia|KM221101|Wenchang, Hainan province, China
Lottia luchuana|Mollusca, Gastropoda, Patellogastropoda, Lottiidae, Lottia|KM221049|Beibu Bay, Guangxi province, China
Lottia luchuana|Mollusca, Gastropoda, Patellogastropoda, Lottiidae, Lottia|KM221048|Beibu Bay, Guangxi province, China
Lottia luchuana|Mollusca, Gastropoda, Patellogastropoda, Lottiidae, Lottia|KM221045|Beibu Bay, Guangxi province, China
Lottia luchuana|Mollusca, Gastropoda, Patellogastropoda, Lottiidae, Lottia|KM221044|Beibu Bay, Guangxi province, China
Lottia luchuana|Mollusca, Gastropoda, Patellogastropoda, Lottiidae, Lottia|KM221094|Yangjiang, Guangdong province, China
Lottia luchuana|Mollusca, Gastropoda, Patellogastropoda, Lottiidae, Lottia|KM221056|Nanji Island, Zhejiang province, China
Lottia luchuana|Mollusca, Gastropoda, Patellogastropoda, Lottiidae, Lottia|KM221100|Wenchang, Hainan province, China
Lottia dorsuosa|Mollusca, Gastropoda, Patellogastropoda, Lottiidae, Lottia|KM221108|Zhoushan, Zhejiang province, China
Lottia dorsuosa|Mollusca, Gastropoda, Patellogastropoda, Lottiidae, Lottia|KM221054|Zhoushan, Zhejiang province, China
Lottia dorsuosa|Mollusca, Gastropoda, Patellogastropoda, Lottiidae, Lottia|KM221109|Zhoushan, Zhejiang province, China
Lottia lindbergi|Mollusca, Gastropoda, Patellogastropoda, Lottiidae, Lottia|AB238470|Hokkaido, Akkeshi, Hokkaido, Japan
Lottia kogamogai|Mollusca, Gastropoda, Patellogastropoda, Lottiidae, Lottia|AB238467|Ibaraki, Japan
Lottia cassis|Mollusca, Gastropoda, Patellogastropoda, Lottiidae, Lottia|KM221116|Rongcheng, Shandong province, China
Lottia cassis|Mollusca, Gastropoda, Patellogastropoda, Lottiidae, Lottia|KM221115|Rongcheng, Shandong province, China
Lottia cassis|Mollusca, Gastropoda, Patellogastropoda, Lottiidae, Lottia|KM221043|Rongcheng, Shandong province, China
Lottia cassis|Mollusca, Gastropoda, Patellogastropoda, Lottiidae, Lottia|KM221036|Rongcheng, Shandong province, China
Lottia cassis|Mollusca, Gastropoda, Patellogastropoda, Lottiidae, Lottia|KM221034|Rongcheng, Shandong province, China
Lottia cassis|Mollusca, Gastropoda, Patellogastropoda, Lottiidae, Lottia|KM221042|Rongcheng, Shandong province, China
Lottia langfordi|Mollusca, Gastropoda, Patellogastropoda, Lottiidae, Lottia|AB238468|Kochi, Goshikinohama, Japan

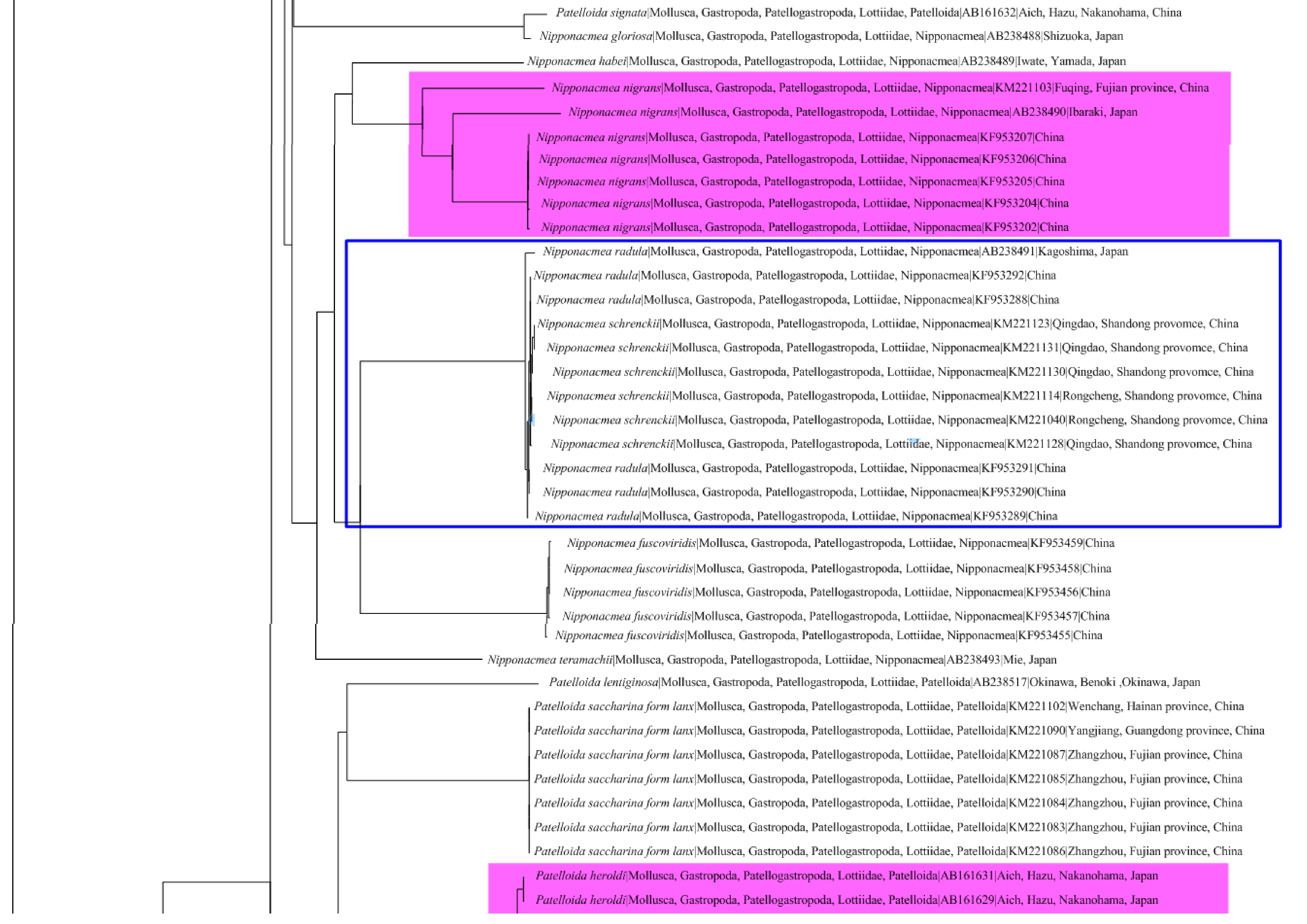

Patelloida signata|Mollusca, Gastropoda, Patellogastropoda, Lottiidae, Patelloida|AB161632|Aich, Hazu, Nakanohama, China
Nipponacmea gloriosa|Mollusca, Gastropoda, Patellogastropoda, Lottiidae, Nipponacmea|AB238488|Shizuoka, Japan
Nipponacmea habei|Mollusca, Gastropoda, Patellogastropoda, Lottiidae, Nipponacmea|AB238489|Iwate, Yamada, Japan
Nipponacmea nigrans|Mollusca, Gastropoda, Patellogastropoda, Lottiidae, Nipponacmea|KM221103|Fuqing, Fujian province, China
Nipponacmea nigrans|Mollusca, Gastropoda, Patellogastropoda, Lottiidae, Nipponacmea|AB238490|Ibaraki, Japan
Nipponacmea nigrans|Mollusca, Gastropoda, Patellogastropoda, Lottiidae, Nipponacmea|KF953207|China
Nipponacmea nigrans|Mollusca, Gastropoda, Patellogastropoda, Lottiidae, Nipponacmea|KF953206|China
Nipponacmea nigrans|Mollusca, Gastropoda, Patellogastropoda, Lottiidae, Nipponacmea|KF953205|China
Nipponacmea nigrans|Mollusca, Gastropoda, Patellogastropoda, Lottiidae, Nipponacmea|KF953204|China
Nipponacmea nigrans|Mollusca, Gastropoda, Patellogastropoda, Lottiidae, Nipponacmea|KF953202|China
Nipponacmea radula|Mollusca, Gastropoda, Patellogastropoda, Lottiidae, Nipponacmea|AB238491|Kagoshima, Japan
Nipponacmea radula|Mollusca, Gastropoda, Patellogastropoda, Lottiidae, Nipponacmea|KF953292|China
Nipponacmea radula|Mollusca, Gastropoda, Patellogastropoda, Lottiidae, Nipponacmea|KF953288|China
Nipponacmea schrenckii|Mollusca, Gastropoda, Patellogastropoda, Lottiidae, Nipponacmea|KM221123|Qingdao, Shandong provomce, China
Nipponacmea schrenckii|Mollusca, Gastropoda, Patellogastropoda, Lottiidae, Nipponacmea|KM221131|Qingdao, Shandong provomce, China
Nipponacmea schrenckii|Mollusca, Gastropoda, Patellogastropoda, Lottiidae, Nipponacmea|KM221130|Qingdao, Shandong provomce, China
Nipponacmea schrenckii|Mollusca, Gastropoda, Patellogastropoda, Lottiidae, Nipponacmea|KM221114|Rongcheng, Shandong provomce, China
Nipponacmea schrenckii|Mollusca, Gastropoda, Patellogastropoda, Lottiidae, Nipponacmea|KM221040|Rongcheng, Shandong provomce, China
Nipponacmea schrenckii|Mollusca, Gastropoda, Patellogastropoda, Lottiidae, Nipponacmea|KM221128|Qingdao, Shandong provomce, China
Nipponacmea radula|Mollusca, Gastropoda, Patellogastropoda, Lottiidae, Nipponacmea|KF953291|China
Nipponacmea radula|Mollusca, Gastropoda, Patellogastropoda, Lottiidae, Nipponacmea|KF953290|China
Nipponacmea radula|Mollusca, Gastropoda, Patellogastropoda, Lottiidae, Nipponacmea|KF953289|China
Nipponacmea fuscoviridis|Mollusca, Gastropoda, Patellogastropoda, Lottiidae, Nipponacmea|KF953459|China
Nipponacmea fuscoviridis|Mollusca, Gastropoda, Patellogastropoda, Lottiidae, Nipponacmea|KF953458|China
Nipponacmea fuscoviridis|Mollusca, Gastropoda, Patellogastropoda, Lottiidae, Nipponacmea|KF953456|China
Nipponacmea fuscoviridis|Mollusca, Gastropoda, Patellogastropoda, Lottiidae, Nipponacmea|KF953457|China
Nipponacmea fuscoviridis|Mollusca, Gastropoda, Patellogastropoda, Lottiidae, Nipponacmea|KF953455|China
Nipponacmea teramachii|Mollusca, Gastropoda, Patellogastropoda, Lottiidae, Nipponacmea|AB238493|Mie, Japan
Patelloida lentiginosa|Mollusca, Gastropoda, Patellogastropoda, Lottiidae, Patelloida|AB238517|Okinawa, Benoki ,Okinawa, Japan
Patelloida saccharina form lanx|Mollusca, Gastropoda, Patellogastropoda, Lottiidae, Patelloida|KM221102|Wenchang, Hainan province, China
Patelloida saccharina form lanx|Mollusca, Gastropoda, Patellogastropoda, Lottiidae, Patelloida|KM221090|Yangjiang, Guangdong province, China
Patelloida saccharina form lanx|Mollusca, Gastropoda, Patellogastropoda, Lottiidae, Patelloida|KM221087|Zhangzhou, Fujian province, China
Patelloida saccharina form lanx|Mollusca, Gastropoda, Patellogastropoda, Lottiidae, Patelloida|KM221085|Zhangzhou, Fujian province, China
Patelloida saccharina form lanx|Mollusca, Gastropoda, Patellogastropoda, Lottiidae, Patelloida|KM221084|Zhangzhou, Fujian province, China
Patelloida saccharina form lanx|Mollusca, Gastropoda, Patellogastropoda, Lottiidae, Patelloida|KM221083|Zhangzhou, Fujian province, China
Patelloida saccharina form lanx|Mollusca, Gastropoda, Patellogastropoda, Lottiidae, Patelloida|KM221086|Zhangzhou, Fujian province, China
Patelloida heroldi|Mollusca, Gastropoda, Patellogastropoda, Lottiidae, Patelloida|AB161631|Aich, Hazu, Nakanohama, Japan
Patelloida heroldi|Mollusca, Gastropoda, Patellogastropoda, Lottiidae, Patelloida|AB161629|Aich, Hazu, Nakanohama, Japan

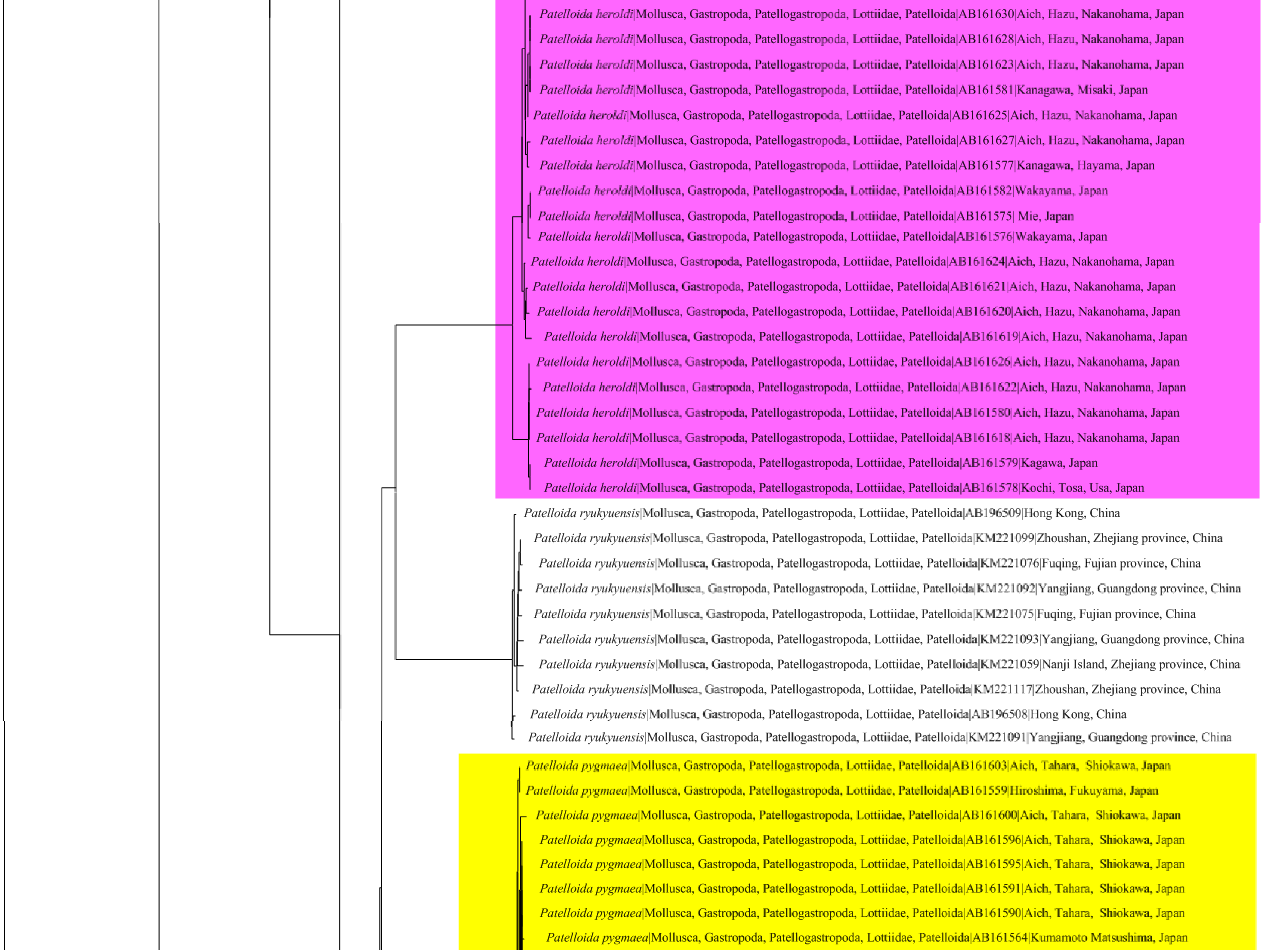

Patelloida heroldi|Mollusca, Gastropoda, Patellogastropoda, Lottiidae, Patelloida|AB161630|Aich, Hazu, Nakanohama, Japan
Patelloida heroldi|Mollusca, Gastropoda, Patellogastropoda, Lottiidae, Patelloida|AB161628|Aich, Hazu, Nakanohama, Japan
Patelloida heroldi|Mollusca, Gastropoda, Patellogastropoda, Lottiidae, Patelloida|AB161623|Aich, Hazu, Nakanohama, Japan
Patelloida heroldi|Mollusca, Gastropoda, Patellogastropoda, Lottiidae, Patelloida|AB161581|Kanagawa, Misaki, Japan
Patelloida heroldi|Mollusca, Gastropoda, Patellogastropoda, Lottiidae, Patelloida|AB161625|Aich, Hazu, Nakanohama, Japan
Patelloida heroldi|Mollusca, Gastropoda, Patellogastropoda, Lottiidae, Patelloida|AB161627|Aich, Hazu, Nakanohama, Japan
Patelloida heroldi|Mollusca, Gastropoda, Patellogastropoda, Lottiidae, Patelloida|AB161577|Kanagawa, Hayama, Japan
Patelloida heroldi|Mollusca, Gastropoda, Patellogastropoda, Lottiidae, Patelloida|AB161582|Wakayama, Japan
Patelloida heroldi|Mollusca, Gastropoda, Patellogastropoda, Lottiidae, Patelloida|AB161575| Mie, Japan
Patelloida heroldi|Mollusca, Gastropoda, Patellogastropoda, Lottiidae, Patelloida|AB161576|Wakayama, Japan
Patelloida heroldi|Mollusca, Gastropoda, Patellogastropoda, Lottiidae, Patelloida|AB161624|Aich, Hazu, Nakanohama, Japan
Patelloida heroldi|Mollusca, Gastropoda, Patellogastropoda, Lottiidae, Patelloida|AB161621|Aich, Hazu, Nakanohama, Japan
Patelloida heroldi|Mollusca, Gastropoda, Patellogastropoda, Lottiidae, Patelloida|AB161620|Aich, Hazu, Nakanohama, Japan
Patelloida heroldi|Mollusca, Gastropoda, Patellogastropoda, Lottiidae, Patelloida|AB161619|Aich, Hazu, Nakanohama, Japan
Patelloida heroldi|Mollusca, Gastropoda, Patellogastropoda, Lottiidae, Patelloida|AB161626|Aich, Hazu, Nakanohama, Japan
Patelloida heroldi|Mollusca, Gastropoda, Patellogastropoda, Lottiidae, Patelloida|AB161622|Aich, Hazu, Nakanohama, Japan
Patelloida heroldi|Mollusca, Gastropoda, Patellogastropoda, Lottiidae, Patelloida|AB161580|Aich, Hazu, Nakanohama, Japan
Patelloida heroldi|Mollusca, Gastropoda, Patellogastropoda, Lottiidae, Patelloida|AB161618|Aich, Hazu, Nakanohama, Japan
Patelloida heroldi|Mollusca, Gastropoda, Patellogastropoda, Lottiidae, Patelloida|AB161579|Kagawa, Japan
Patelloida heroldi|Mollusca, Gastropoda, Patellogastropoda, Lottiidae, Patelloida|AB161578|Kochi, Tosa, Usa, Japan
Patelloida ryukyuensis|Mollusca, Gastropoda, Patellogastropoda, Lottiidae, Patelloida|AB196509|Hong Kong, China
Patelloida ryukyuensis|Mollusca, Gastropoda, Patellogastropoda, Lottiidae, Patelloida|KM221099|Zhoushan, Zhejiang province, China
Patelloida ryukyuensis|Mollusca, Gastropoda, Patellogastropoda, Lottiidae, Patelloida|KM221076|Fuqing, Fujian province, China
Patelloida ryukyuensis|Mollusca, Gastropoda, Patellogastropoda, Lottiidae, Patelloida|KM221092|Yangjiang, Guangdong province, China
Patelloida ryukyuensis|Mollusca, Gastropoda, Patellogastropoda, Lottiidae, Patelloida|KM221075|Fuqing, Fujian province, China
Patelloida ryukyuensis|Mollusca, Gastropoda, Patellogastropoda, Lottiidae, Patelloida|KM221093|Yangjiang, Guangdong province, China
Patelloida ryukyuensis|Mollusca, Gastropoda, Patellogastropoda, Lottiidae, Patelloida|KM221059|Nanji Island, Zhejiang province, China
Patelloida ryukyuensis|Mollusca, Gastropoda, Patellogastropoda, Lottiidae, Patelloida|KM221117|Zhoushan, Zhejiang province, China
Patelloida ryukyuensis|Mollusca, Gastropoda, Patellogastropoda, Lottiidae, Patelloida|AB196508|Hong Kong, China
Patelloida ryukyuensis|Mollusca, Gastropoda, Patellogastropoda, Lottiidae, Patelloida|KM221091|Yangjiang, Guangdong province, China
Patelloida pygmaea|Mollusca, Gastropoda, Patellogastropoda, Lottiidae, Patelloida|AB161603|Aich, Tahara, Shiokawa, Japan
Patelloida pygmaea|Mollusca, Gastropoda, Patellogastropoda, Lottiidae, Patelloida|AB161559|Hiroshima, Fukuyama, Japan
Patelloida pygmaea|Mollusca, Gastropoda, Patellogastropoda, Lottiidae, Patelloida|AB161600|Aich, Tahara, Shiokawa, Japan
Patelloida pygmaea|Mollusca, Gastropoda, Patellogastropoda, Lottiidae, Patelloida|AB161596|Aich, Tahara, Shiokawa, Japan
Patelloida pygmaea|Mollusca, Gastropoda, Patellogastropoda, Lottiidae, Patelloida|AB161595|Aich, Tahara, Shiokawa, Japan
Patelloida pygmaea|Mollusca, Gastropoda, Patellogastropoda, Lottiidae, Patelloida|AB161591|Aich, Tahara, Shiokawa, Japan
Patelloida pygmaea|Mollusca, Gastropoda, Patellogastropoda, Lottiidae, Patelloida|AB161590|Aich, Tahara, Shiokawa, Japan
Patelloida pygmaea|Mollusca, Gastropoda, Patellogastropoda, Lottiidae, Patelloida|AB161564|Kumamoto Matsushima, Japan

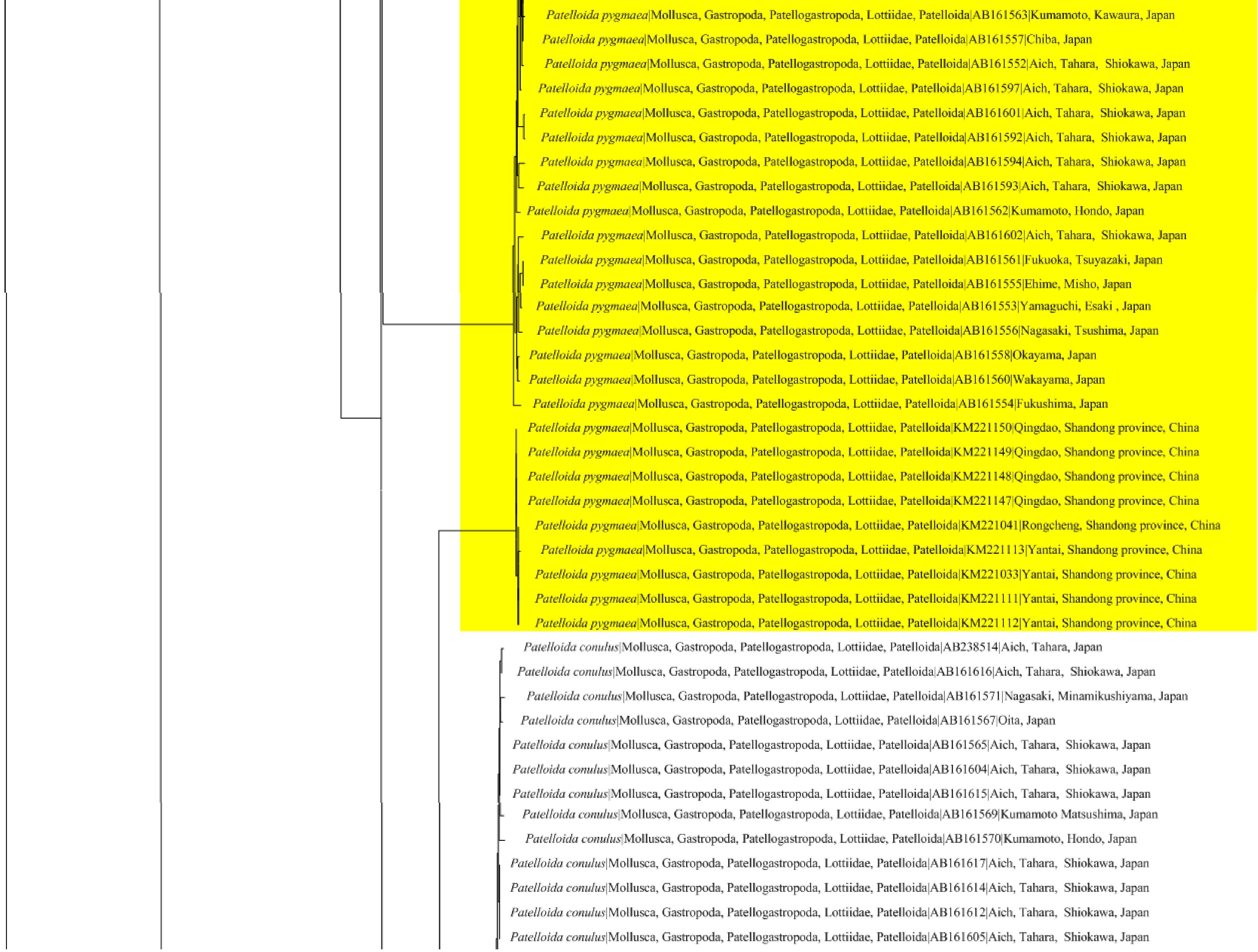

Patelloida pygmaea|Mollusca, Gastropoda, Patellogastropoda, Lottiidae, Patelloida|AB161563|Kumamoto, Kawaura, Japan
Patelloida pygmaea|Mollusca, Gastropoda, Patellogastropoda, Lottiidae, Patelloida|AB161557|Chiba, Japan
Patelloida pygmaea|Mollusca, Gastropoda, Patellogastropoda, Lottiidae, Patelloida|AB161552|Aich, Tahara, Shiokawa, Japan
Patelloida pygmaea|Mollusca, Gastropoda, Patellogastropoda, Lottiidae, Patelloida|AB161597|Aich, Tahara, Shiokawa, Japan
Patelloida pygmaea|Mollusca, Gastropoda, Patellogastropoda, Lottiidae, Patelloida|AB161601|Aich, Tahara, Shiokawa, Japan
Patelloida pygmaea|Mollusca, Gastropoda, Patellogastropoda, Lottiidae, Patelloida|AB161592|Aich, Tahara, Shiokawa, Japan
Patelloida pygmaea|Mollusca, Gastropoda, Patellogastropoda, Lottiidae, Patelloida|AB161594|Aich, Tahara, Shiokawa, Japan
Patelloida pygmaea|Mollusca, Gastropoda, Patellogastropoda, Lottiidae, Patelloida|AB161593|Aich, Tahara, Shiokawa, Japan
Patelloida pygmaea|Mollusca, Gastropoda, Patellogastropoda, Lottiidae, Patelloida|AB161562|Kumamoto, Hondo, Japan
Patelloida pygmaea|Mollusca, Gastropoda, Patellogastropoda, Lottiidae, Patelloida|AB161602|Aich, Tahara, Shiokawa, Japan
Patelloida pygmaea|Mollusca, Gastropoda, Patellogastropoda, Lottiidae, Patelloida|AB161561|Fukuoka, Tsuyazaki, Japan
Patelloida pygmaea|Mollusca, Gastropoda, Patellogastropoda, Lottiidae, Patelloida|AB161555|Ehime, Misho, Japan
Patelloida pygmaea|Mollusca, Gastropoda, Patellogastropoda, Lottiidae, Patelloida|AB161553|Yamaguchi, Esaki , Japan
Patelloida pygmaea|Mollusca, Gastropoda, Patellogastropoda, Lottiidae, Patelloida|AB161556|Nagasaki, Tsushima, Japan
Patelloida pygmaea|Mollusca, Gastropoda, Patellogastropoda, Lottiidae, Patelloida|AB161558|Okayama, Japan
Patelloida pygmaea|Mollusca, Gastropoda, Patellogastropoda, Lottiidae, Patelloida|AB161560|Wakayama, Japan
Patelloida pygmaea|Mollusca, Gastropoda, Patellogastropoda, Lottiidae, Patelloida|AB161554|Fukushima, Japan
Patelloida pygmaea|Mollusca, Gastropoda, Patellogastropoda, Lottiidae, Patelloida|KM221150|Qingdao, Shandong province, China
Patelloida pygmaea|Mollusca, Gastropoda, Patellogastropoda, Lottiidae, Patelloida|KM221149|Qingdao, Shandong province, China
Patelloida pygmaea|Mollusca, Gastropoda, Patellogastropoda, Lottiidae, Patelloida|KM221148|Qingdao, Shandong province, China
Patelloida pygmaea|Mollusca, Gastropoda, Patellogastropoda, Lottiidae, Patelloida|KM221147|Qingdao, Shandong province, China
Patelloida pygmaea|Mollusca, Gastropoda, Patellogastropoda, Lottiidae, Patelloida|KM221041|Rongcheng, Shandong province, China
Patelloida pygmaea|Mollusca, Gastropoda, Patellogastropoda, Lottiidae, Patelloida|KM221113|Yantai, Shandong province, China
Patelloida pygmaea|Mollusca, Gastropoda, Patellogastropoda, Lottiidae, Patelloida|KM221033|Yantai, Shandong province, China
Patelloida pygmaea|Mollusca, Gastropoda, Patellogastropoda, Lottiidae, Patelloida|KM221111|Yantai, Shandong province, China
Patelloida pygmaea|Mollusca, Gastropoda, Patellogastropoda, Lottiidae, Patelloida|KM221112|Yantai, Shandong province, China
Patelloida conulus|Mollusca, Gastropoda, Patellogastropoda, Lottiidae, Patelloida|AB238514|Aich, Tahara, Japan
Patelloida conulus|Mollusca, Gastropoda, Patellogastropoda, Lottiidae, Patelloida|AB161616|Aich, Tahara, Shiokawa, Japan
Patelloida conulus|Mollusca, Gastropoda, Patellogastropoda, Lottiidae, Patelloida|AB161571|Nagasaki, Minamikushiyama, Japan
Patelloida conulus|Mollusca, Gastropoda, Patellogastropoda, Lottiidae, Patelloida|AB161567|Oita, Japan
Patelloida conulus|Mollusca, Gastropoda, Patellogastropoda, Lottiidae, Patelloida|AB161565|Aich, Tahara, Shiokawa, Japan
Patelloida conulus|Mollusca, Gastropoda, Patellogastropoda, Lottiidae, Patelloida|AB161604|Aich, Tahara, Shiokawa, Japan
Patelloida conulus|Mollusca, Gastropoda, Patellogastropoda, Lottiidae, Patelloida|AB161615|Aich, Tahara, Shiokawa, Japan
Patelloida conulus|Mollusca, Gastropoda, Patellogastropoda, Lottiidae, Patelloida|AB161569|Kumamoto Matsushima, Japan
Patelloida conulus|Mollusca, Gastropoda, Patellogastropoda, Lottiidae, Patelloida|AB161570|Kumamoto, Hondo, Japan
Patelloida conulus|Mollusca, Gastropoda, Patellogastropoda, Lottiidae, Patelloida|AB161617|Aich, Tahara, Shiokawa, Japan
Patelloida conulus|Mollusca, Gastropoda, Patellogastropoda, Lottiidae, Patelloida|AB161614|Aich, Tahara, Shiokawa, Japan
Patelloida conulus|Mollusca, Gastropoda, Patellogastropoda, Lottiidae, Patelloida|AB161612|Aich, Tahara, Shiokawa, Japan
Patelloida conulus|Mollusca, Gastropoda, Patellogastropoda, Lottiidae, Patelloida|AB161605|Aich, Tahara, Shiokawa, Japan

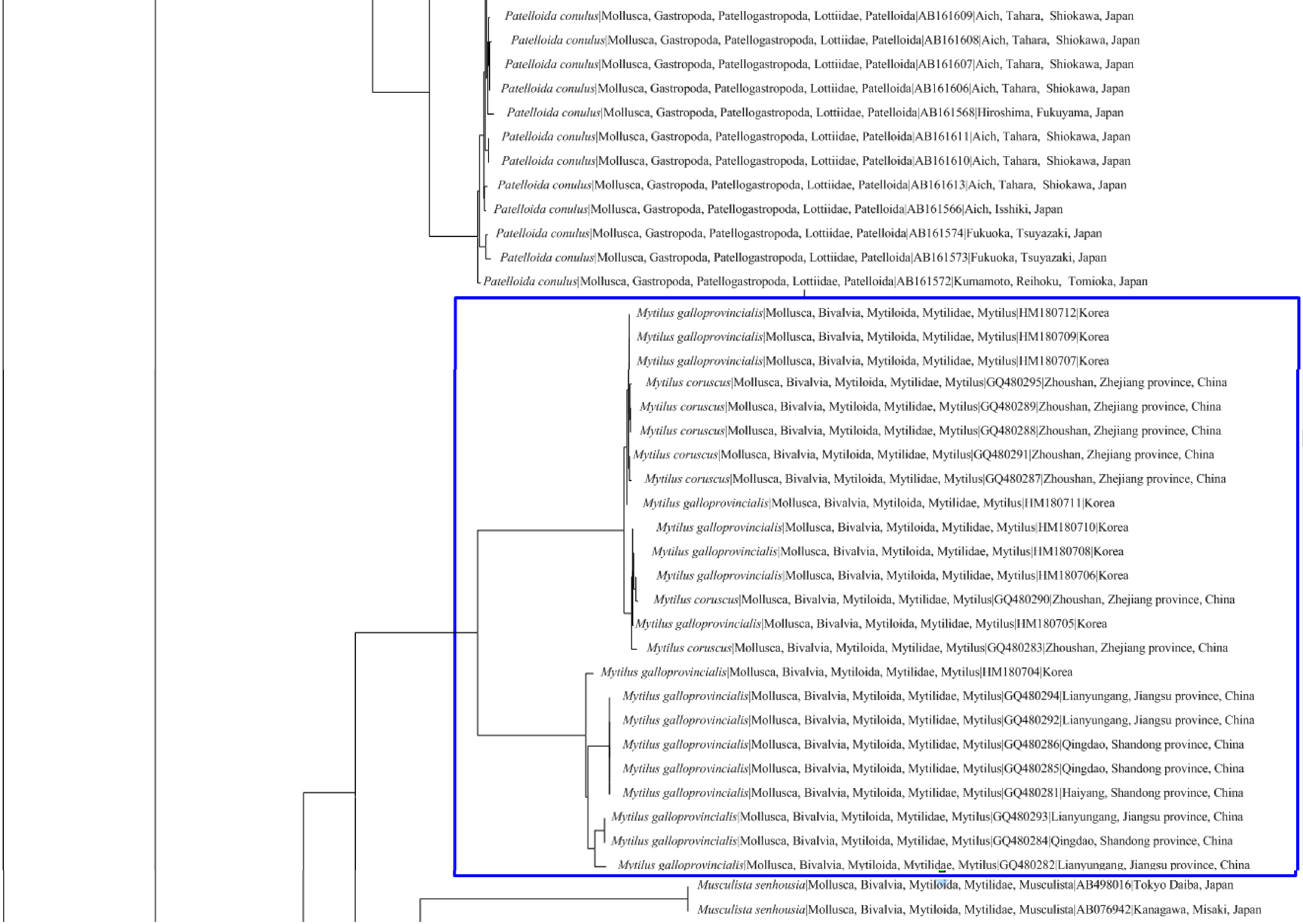

Patelloida conulus|Mollusca, Gastropoda, Patellogastropoda, Lottiidae, Patelloida|AB161609|Aich, Tahara, Shiokawa, Japan
Patelloida conulus|Mollusca, Gastropoda, Patellogastropoda, Lottiidae, Patelloida|AB161608|Aich, Tahara, Shiokawa, Japan
Patelloida conulus|Mollusca, Gastropoda, Patellogastropoda, Lottiidae, Patelloida|AB161607|Aich, Tahara, Shiokawa, Japan
Patelloida conulus|Mollusca, Gastropoda, Patellogastropoda, Lottiidae, Patelloida|AB161606|Aich, Tahara, Shiokawa, Japan
Patelloida conulus|Mollusca, Gastropoda, Patellogastropoda, Lottiidae, Patelloida|AB161568|Hiroshima, Fukuyama, Japan
Patelloida conulus|Mollusca, Gastropoda, Patellogastropoda, Lottiidae, Patelloida|AB161611|Aich, Tahara, Shiokawa, Japan
Patelloida conulus|Mollusca, Gastropoda, Patellogastropoda, Lottiidae, Patelloida|AB161610|Aich, Tahara, Shiokawa, Japan
Patelloida conulus|Mollusca, Gastropoda, Patellogastropoda, Lottiidae, Patelloida|AB161613|Aich, Tahara, Shiokawa, Japan
Patelloida conulus|Mollusca, Gastropoda, Patellogastropoda, Lottiidae, Patelloida|AB161566|Aich, Isshiki, Japan
Patelloida conulus|Mollusca, Gastropoda, Patellogastropoda, Lottiidae, Patelloida|AB161574|Fukuoka, Tsuyazaki, Japan
Patelloida conulus|Mollusca, Gastropoda, Patellogastropoda, Lottiidae, Patelloida|AB161573|Fukuoka, Tsuyazaki, Japan
Patelloida conulus|Mollusca, Gastropoda, Patellogastropoda, Lottiidae, Patelloida|AB161572|Kumamoto, Reihoku, Tomioka, Japan
Mytilus galloprovincialis|Mollusca, Bivalvia, Mytiloida, Mytilidae, Mytilus|HM180712|Korea
Mytilus galloprovincialis|Mollusca, Bivalvia, Mytiloida, Mytilidae, Mytilus|HM180709|Korea
Mytilus galloprovincialis|Mollusca, Bivalvia, Mytiloida, Mytilidae, Mytilus|HM180707|Korea
Mytilus coruscus|Mollusca, Bivalvia, Mytiloida, Mytilidae, Mytilus|GQ480295|Zhoushan, Zhejiang province, China
Mytilus coruscus|Mollusca, Bivalvia, Mytiloida, Mytilidae, Mytilus|GQ480289|Zhoushan, Zhejiang province, China
Mytilus coruscus|Mollusca, Bivalvia, Mytiloida, Mytilidae, Mytilus|GQ480288|Zhoushan, Zhejiang province, China
Mytilus coruscus|Mollusca, Bivalvia, Mytiloida, Mytilidae, Mytilus|GQ480291|Zhoushan, Zhejiang province, China
Mytilus coruscus|Mollusca, Bivalvia, Mytiloida, Mytilidae, Mytilus|GQ480287|Zhoushan, Zhejiang province, China
Mytilus galloprovincialis|Mollusca, Bivalvia, Mytiloida, Mytilidae, Mytilus|HM180711|Korea
Mytilus galloprovincialis|Mollusca, Bivalvia, Mytiloida, Mytilidae, Mytilus|HM180710|Korea
Mytilus galloprovincialis|Mollusca, Bivalvia, Mytiloida, Mytilidae, Mytilus|HM180708|Korea
Mytilus galloprovincialis|Mollusca, Bivalvia, Mytiloida, Mytilidae, Mytilus|HM180706|Korea
Mytilus coruscus|Mollusca, Bivalvia, Mytiloida, Mytilidae, Mytilus|GQ480290|Zhoushan, Zhejiang province, China
Mytilus galloprovincialis|Mollusca, Bivalvia, Mytiloida, Mytilidae, Mytilus|HM180705|Korea
Mytilus coruscus|Mollusca, Bivalvia, Mytiloida, Mytilidae, Mytilus|GQ480283|Zhoushan, Zhejiang province, China
Mytilus galloprovincialis|Mollusca, Bivalvia, Mytiloida, Mytilidae, Mytilus|HM180704|Korea
Mytilus galloprovincialis|Mollusca, Bivalvia, Mytiloida, Mytilidae, Mytilus|GQ480294|Lianyungang, Jiangsu province, China
Mytilus galloprovincialis|Mollusca, Bivalvia, Mytiloida, Mytilidae, Mytilus|GQ480292|Lianyungang, Jiangsu province, China
Mytilus galloprovincialis|Mollusca, Bivalvia, Mytiloida, Mytilidae, Mytilus|GQ480286|Qingdao, Shandong province, China
Mytilus galloprovincialis|Mollusca, Bivalvia, Mytiloida, Mytilidae, Mytilus|GQ480285|Qingdao, Shandong province, China
Mytilus galloprovincialis|Mollusca, Bivalvia, Mytiloida, Mytilidae, Mytilus|GQ480281|Haiyang, Shandong province, China
Mytilus galloprovincialis|Mollusca, Bivalvia, Mytiloida, Mytilidae, Mytilus|GQ480293|Lianyungang, Jiangsu province, China
Mytilus galloprovincialis|Mollusca, Bivalvia, Mytiloida, Mytilidae, Mytilus|GQ480284|Qingdao, Shandong province, China
Mytilus galloprovincialis|Mollusca, Bivalvia, Mytiloida, Mytilidae, Mytilus|GQ480282|Lianyungang, Jiangsu province, China
Musculista senhousia|Mollusca, Bivalvia, Mytiloida, Mytilidae, Musculista|AB498016|Tokyo Daiba, Japan
Musculista senhousia|Mollusca, Bivalvia, Mytiloida, Mytilidae, Musculista|AB076942|Kanagawa, Misaki, Japan

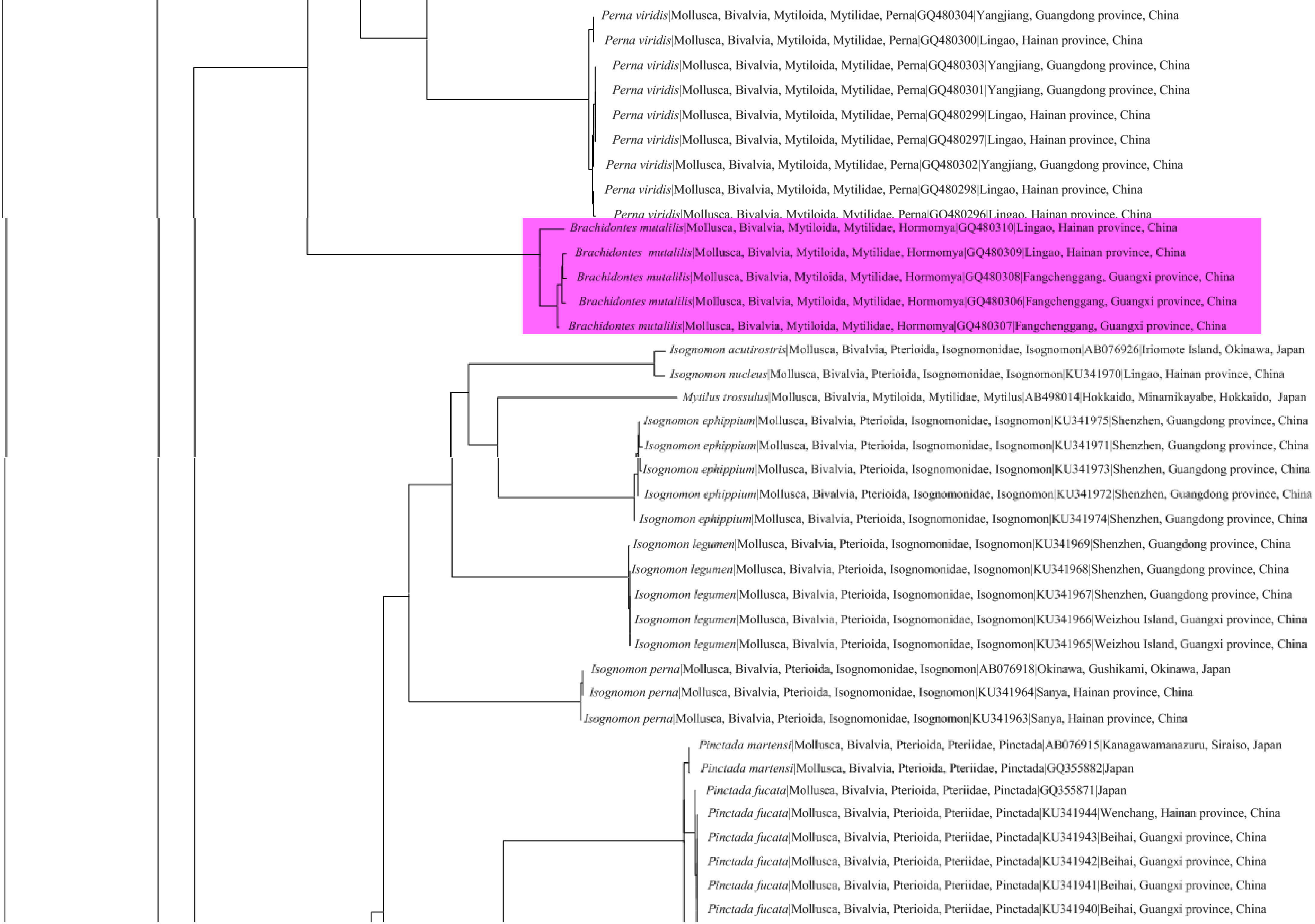

Perna viridis|Mollusca, Bivalvia, Mytiloida, Mytilidae, Perna|GQ480304|Yangjiang, Guangdong province, China
Perna viridis|Mollusca, Bivalvia, Mytiloida, Mytilidae, Perna|GQ480300|Lingao, Hainan province, China
Perna viridis|Mollusca, Bivalvia, Mytiloida, Mytilidae, Perna|GQ480303|Yangjiang, Guangdong province, China
Perna viridis|Mollusca, Bivalvia, Mytiloida, Mytilidae, Perna|GQ480301|Yangjiang, Guangdong province, China
Perna viridis|Mollusca, Bivalvia, Mytiloida, Mytilidae, Perna|GQ480299|Lingao, Hainan province, China
Perna viridis|Mollusca, Bivalvia, Mytiloida, Mytilidae, Perna|GQ480297|Lingao, Hainan province, China
Perna viridis|Mollusca, Bivalvia, Mytiloida, Mytilidae, Perna|GQ480302|Yangjiang, Guangdong province, China
Perna viridis|Mollusca, Bivalvia, Mytiloida, Mytilidae, Perna|GQ480298|Lingao, Hainan province, China
Perna viridis|Mollusca, Bivalvia, Mytiloida, Mytilidae, Perna|GQ480296|Lingao, Hainan province, China
Brachidontes mutalilis|Mollusca, Bivalvia, Mytiloida, Mytilidae, Hormomya|GQ480310|Lingao, Hainan province, China
Brachidontes mutalilis|Mollusca, Bivalvia, Mytiloida, Mytilidae, Hormomya|GQ480309|Lingao, Hainan province, China
Brachidontes mutalilis|Mollusca, Bivalvia, Mytiloida, Mytilidae, Hormomya|GQ480308|Fangchenggang, Guangxi province, China
Brachidontes mutalilis|Mollusca, Bivalvia, Mytiloida, Mytilidae, Hormomya|GQ480306|Fangchenggang, Guangxi province, China
Brachidontes mutalilis|Mollusca, Bivalvia, Mytiloida, Mytilidae, Hormomya|GQ480307|Fangchenggang, Guangxi province, China
Isognomon acutirostris|Mollusca, Bivalvia, Pterioida, Isognomonidae, Isognomon|AB076926|Iriomote Island, Okinawa, Japan
Isognomon nucleus|Mollusca, Bivalvia, Pterioida, Isognomonidae, Isognomon|KU341970|Lingao, Hainan province, China
Mytilus trossulus|Mollusca, Bivalvia, Mytiloida, Mytilidae, Mytilus|AB498014|Hokkaido, Minamikayabe, Hokkaido, Japan
Isognomon ephippium|Mollusca, Bivalvia, Pterioida, Isognomonidae, Isognomon|KU341975|Shenzhen, Guangdong province, China
Isognomon ephippium|Mollusca, Bivalvia, Pterioida, Isognomonidae, Isognomon|KU341971|Shenzhen, Guangdong province, China
Isognomon ephippium|Mollusca, Bivalvia, Pterioida, Isognomonidae, Isognomon|KU341973|Shenzhen, Guangdong province, China
Isognomon ephippium|Mollusca, Bivalvia, Pterioida, Isognomonidae, Isognomon|KU341972|Shenzhen, Guangdong province, China
Isognomon ephippium|Mollusca, Bivalvia, Pterioida, Isognomonidae, Isognomon|KU341974|Shenzhen, Guangdong province, China
Isognomon legumen|Mollusca, Bivalvia, Pterioida, Isognomonidae, Isognomon|KU341969|Shenzhen, Guangdong province, China
Isognomon legumen|Mollusca, Bivalvia, Pterioida, Isognomonidae, Isognomon|KU341968|Shenzhen, Guangdong province, China
Isognomon legumen|Mollusca, Bivalvia, Pterioida, Isognomonidae, Isognomon|KU341967|Shenzhen, Guangdong province, China
Isognomon legumen|Mollusca, Bivalvia, Pterioida, Isognomonidae, Isognomon|KU341966|Weizhou Island, Guangxi province, China
Isognomon legumen|Mollusca, Bivalvia, Pterioida, Isognomonidae, Isognomon|KU341965|Weizhou Island, Guangxi province, China
Isognomon perna|Mollusca, Bivalvia, Pterioida, Isognomonidae, Isognomon|AB076918|Okinawa, Gushikami, Okinawa, Japan
Isognomon perna|Mollusca, Bivalvia, Pterioida, Isognomonidae, Isognomon|KU341964|Sanya, Hainan province, China
Isognomon perna|Mollusca, Bivalvia, Pterioida, Isognomonidae, Isognomon|KU341963|Sanya, Hainan province, China
Pinctada martensi|Mollusca, Bivalvia, Pterioida, Pteriidae, Pinctada|AB076915|Kanagawamanazuru, Siraiso, Japan
Pinctada martensi|Mollusca, Bivalvia, Pterioida, Pteriidae, Pinctada|GQ355882|Japan
Pinctada fucata|Mollusca, Bivalvia, Pterioida, Pteriidae, Pinctada|GQ355871|Japan
Pinctada fucata|Mollusca, Bivalvia, Pterioida, Pteriidae, Pinctada|KU341944|Wenchang, Hainan province, China
Pinctada fucata|Mollusca, Bivalvia, Pterioida, Pteriidae, Pinctada|KU341943|Beihai, Guangxi province, China
Pinctada fucata|Mollusca, Bivalvia, Pterioida, Pteriidae, Pinctada|KU341942|Beihai, Guangxi province, China
Pinctada fucata|Mollusca, Bivalvia, Pterioida, Pteriidae, Pinctada|KU341941|Beihai, Guangxi province, China
Pinctada fucata|Mollusca, Bivalvia, Pterioida, Pteriidae, Pinctada|KU341940|Beihai, Guangxi province, China

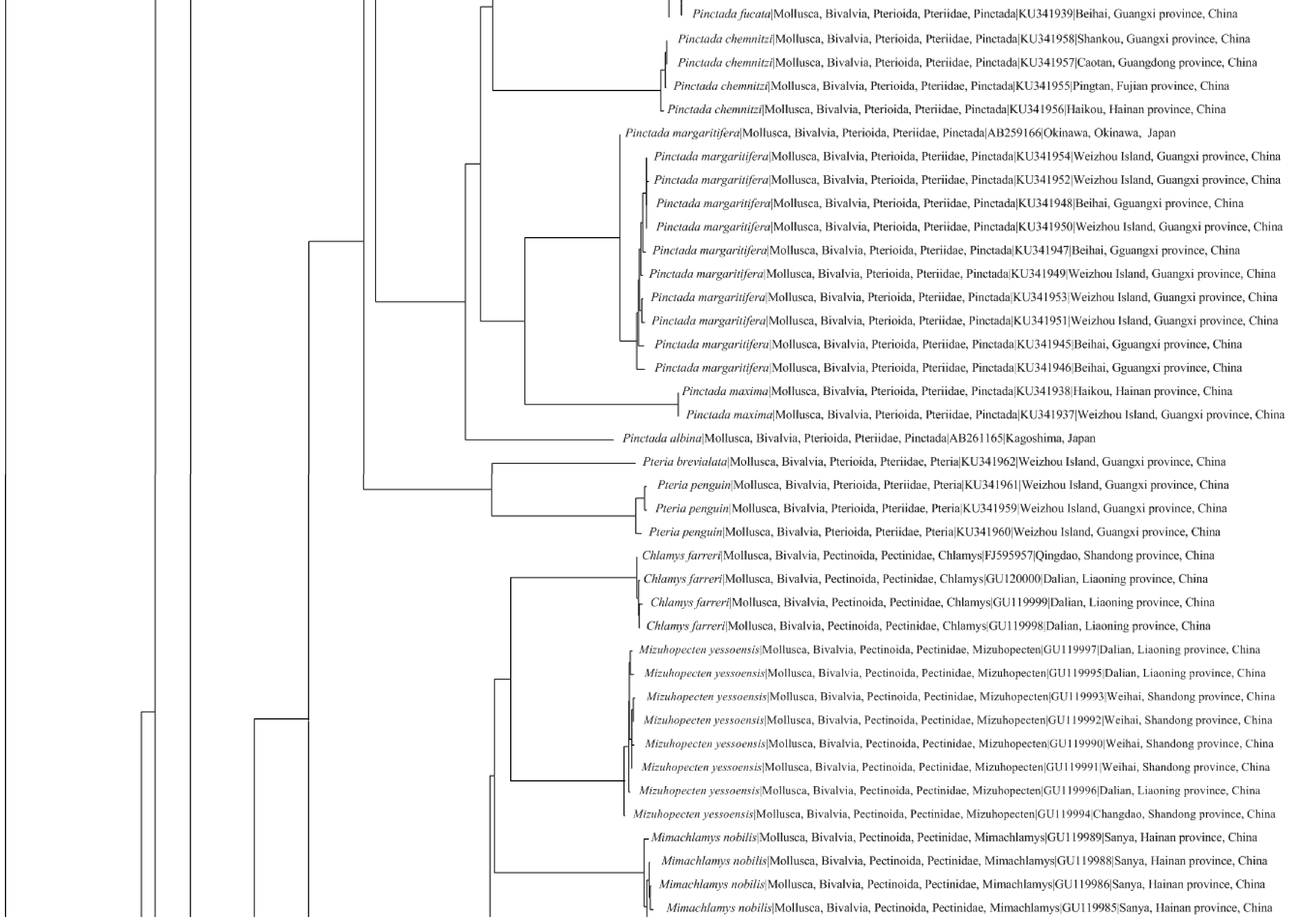

Pinctada fucata|Mollusca, Bivalvia, Pterioida, Pteriidae, Pinctada|KU341939|Beihai, Guangxi province, China
Pinctada chemnitzi|Mollusca, Bivalvia, Pterioida, Pteriidae, Pinctada|KU341958|Shankou, Guangxi province, China
Pinctada chemnitzi|Mollusca, Bivalvia, Pterioida, Pteriidae, Pinctada|KU341957|Caotan, Guangdong province, China
Pinctada chemnitzi|Mollusca, Bivalvia, Pterioida, Pteriidae, Pinctada|KU341955|Pingtan, Fujian province, China
Pinctada chemnitzi|Mollusca, Bivalvia, Pterioida, Pteriidae, Pinctada|KU341956|Haikou, Hainan province, China
Pinctada margaritifera|Mollusca, Bivalvia, Pterioida, Pteriidae, Pinctada|AB259166|Okinawa, Okinawa, Japan
Pinctada margaritifera|Mollusca, Bivalvia, Pterioida, Pteriidae, Pinctada|KU341954|Weizhou Island, Guangxi province, China
Pinctada margaritifera|Mollusca, Bivalvia, Pterioida, Pteriidae, Pinctada|KU341952|Weizhou Island, Guangxi province, China
Pinctada margaritifera|Mollusca, Bivalvia, Pterioida, Pteriidae, Pinctada|KU341948|Beihai, Gguangxi province, China
Pinctada margaritifera|Mollusca, Bivalvia, Pterioida, Pteriidae, Pinctada|KU341950|Weizhou Island, Guangxi province, China
Pinctada margaritifera|Mollusca, Bivalvia, Pterioida, Pteriidae, Pinctada|KU341947|Beihai, Gguangxi province, China
Pinctada margaritifera|Mollusca, Bivalvia, Pterioida, Pteriidae, Pinctada|KU341949|Weizhou Island, Guangxi province, China
Pinctada margaritifera|Mollusca, Bivalvia, Pterioida, Pteriidae, Pinctada|KU341953|Weizhou Island, Guangxi province, China
Pinctada margaritifera|Mollusca, Bivalvia, Pterioida, Pteriidae, Pinctada|KU341951|Weizhou Island, Guangxi province, China
Pinctada margaritifera|Mollusca, Bivalvia, Pterioida, Pteriidae, Pinctada|KU341945|Beihai, Gguangxi province, China
Pinctada margaritifera|Mollusca, Bivalvia, Pterioida, Pteriidae, Pinctada|KU341946|Beihai, Gguangxi province, China
Pinctada maxima|Mollusca, Bivalvia, Pterioida, Pteriidae, Pinctada|KU341938|Haikou, Hainan province, China
Pinctada maxima|Mollusca, Bivalvia, Pterioida, Pteriidae, Pinctada|KU341937|Weizhou Island, Guangxi province, China
Pinctada albina|Mollusca, Bivalvia, Pterioida, Pteriidae, Pinctada|AB261165|Kagoshima, Japan
Pteria brevialata|Mollusca, Bivalvia, Pterioida, Pteriidae, Pteria|KU341962|Weizhou Island, Guangxi province, China
Pteria penguin|Mollusca, Bivalvia, Pterioida, Pteriidae, Pteria|KU341961|Weizhou Island, Guangxi province, China
Pteria penguin|Mollusca, Bivalvia, Pterioida, Pteriidae, Pteria|KU341959|Weizhou Island, Guangxi province, China
Pteria penguin|Mollusca, Bivalvia, Pterioida, Pteriidae, Pteria|KU341960|Weizhou Island, Guangxi province, China
Chlamys farreri|Mollusca, Bivalvia, Pectinoida, Pectinidae, Chlamys|FJ595957|Qingdao, Shandong province, China
Chlamys farreri|Mollusca, Bivalvia, Pectinoida, Pectinidae, Chlamys|GU120000|Dalian, Liaoning province, China
Chlamys farreri|Mollusca, Bivalvia, Pectinoida, Pectinidae, Chlamys|GU119999|Dalian, Liaoning province, China
Chlamys farreri|Mollusca, Bivalvia, Pectinoida, Pectinidae, Chlamys|GU119998|Dalian, Liaoning province, China
Mizuhopecten yessoensis|Mollusca, Bivalvia, Pectinoida, Pectinidae, Mizuhopecten|GU119997|Dalian, Liaoning province, China
Mizuhopecten yessoensis|Mollusca, Bivalvia, Pectinoida, Pectinidae, Mizuhopecten|GU119995|Dalian, Liaoning province, China
Mizuhopecten yessoensis|Mollusca, Bivalvia, Pectinoida, Pectinidae, Mizuhopecten|GU119993|Weihai, Shandong province, China
Mizuhopecten yessoensis|Mollusca, Bivalvia, Pectinoida, Pectinidae, Mizuhopecten|GU119992|Weihai, Shandong province, China
Mizuhopecten yessoensis|Mollusca, Bivalvia, Pectinoida, Pectinidae, Mizuhopecten|GU119990|Weihai, Shandong province, China
Mizuhopecten yessoensis|Mollusca, Bivalvia, Pectinoida, Pectinidae, Mizuhopecten|GU119991|Weihai, Shandong province, China
Mizuhopecten yessoensis|Mollusca, Bivalvia, Pectinoida, Pectinidae, Mizuhopecten|GU119996|Dalian, Liaoning province, China
Mizuhopecten yessoensis|Mollusca, Bivalvia, Pectinoida, Pectinidae, Mizuhopecten|GU119994|Changdao, Shandong province, China
Mimachlamys nobilis|Mollusca, Bivalvia, Pectinoida, Pectinidae, Mimachlamys|GU119989|Sanya, Hainan province, China
Mimachlamys nobilis|Mollusca, Bivalvia, Pectinoida, Pectinidae, Mimachlamys|GU119988|Sanya, Hainan province, China
Mimachlamys nobilis|Mollusca, Bivalvia, Pectinoida, Pectinidae, Mimachlamys|GU119986|Sanya, Hainan province, China
Mimachlamys nobilis|Mollusca, Bivalvia, Pectinoida, Pectinidae, Mimachlamys|GU119985|Sanya, Hainan province, China

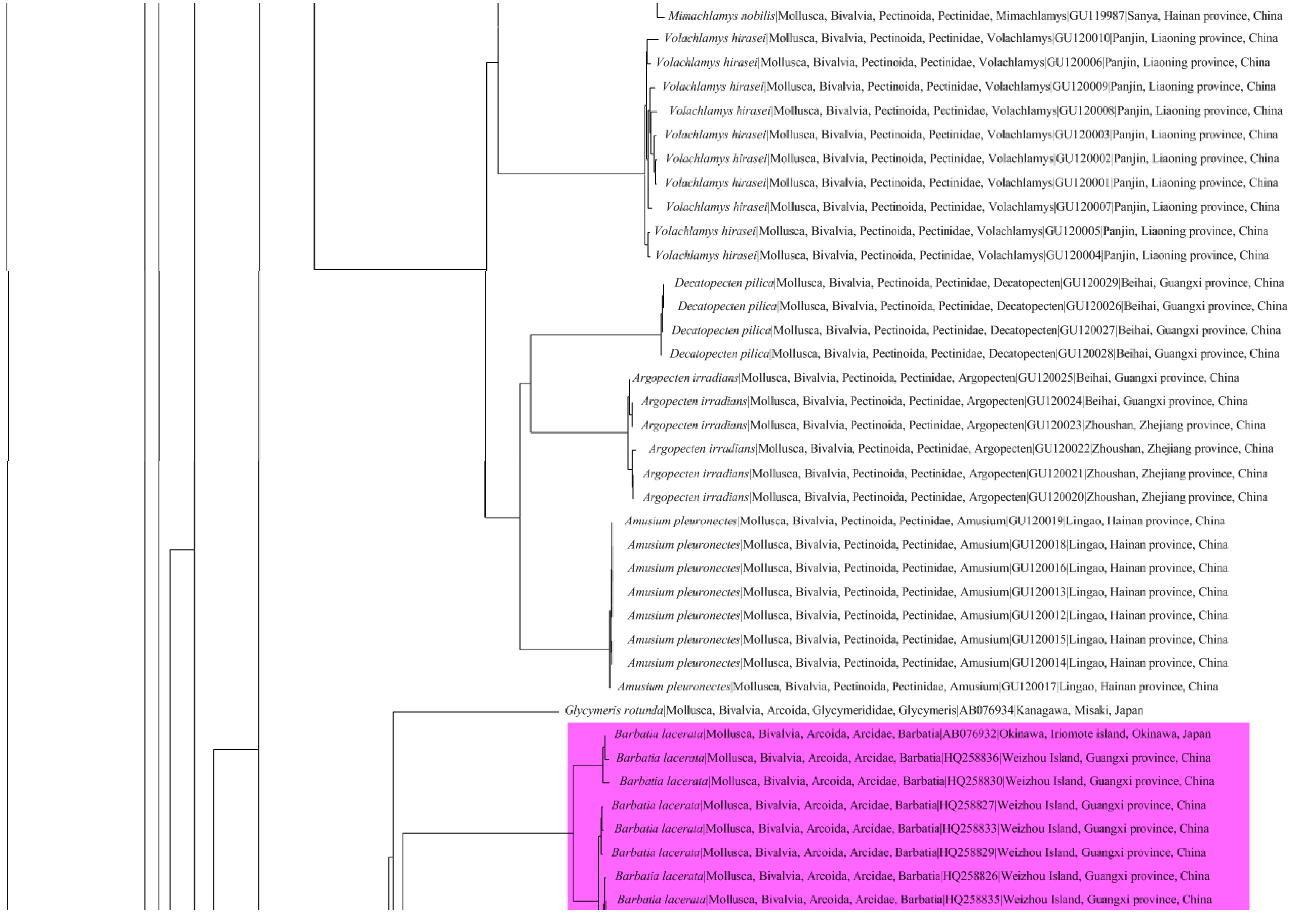

Mimachlamys nobilis|Mollusca, Bivalvia, Pectinoida, Pectinidae, Mimachlamys|GU119987|Sanya, Hainan province, China
Volachlamys hirasei|Mollusca, Bivalvia, Pectinoida, Pectinidae, Volachlamys|GU120010|Panjin, Liaoning province, China
Volachlamys hirasei|Mollusca, Bivalvia, Pectinoida, Pectinidae, Volachlamys|GU120006|Panjin, Liaoning province, China
Volachlamys hirasei|Mollusca, Bivalvia, Pectinoida, Pectinidae, Volachlamys|GU120009|Panjin, Liaoning province, China
Volachlamys hirasei|Mollusca, Bivalvia, Pectinoida, Pectinidae, Volachlamys|GU120008|Panjin, Liaoning province, China
Volachlamys hirasei|Mollusca, Bivalvia, Pectinoida, Pectinidae, Volachlamys|GU120003|Panjin, Liaoning province, China
Volachlamys hirasei|Mollusca, Bivalvia, Pectinoida, Pectinidae, Volachlamys|GU120002|Panjin, Liaoning province, China
Volachlamys hirasei|Mollusca, Bivalvia, Pectinoida, Pectinidae, Volachlamys|GU120001|Panjin, Liaoning province, China
Volachlamys hirasei|Mollusca, Bivalvia, Pectinoida, Pectinidae, Volachlamys|GU120007|Panjin, Liaoning province, China
Volachlamys hirasei|Mollusca, Bivalvia, Pectinoida, Pectinidae, Volachlamys|GU120005|Panjin, Liaoning province, China
Volachlamys hirasei|Mollusca, Bivalvia, Pectinoida, Pectinidae, Volachlamys|GU120004|Panjin, Liaoning province, China
Decatopecten pilica|Mollusca, Bivalvia, Pectinoida, Pectinidae, Decatopecten|GU120029|Beihai, Guangxi province, China
Decatopecten pilica|Mollusca, Bivalvia, Pectinoida, Pectinidae, Decatopecten|GU120026|Beihai, Guangxi province, China
Decatopecten pilica|Mollusca, Bivalvia, Pectinoida, Pectinidae, Decatopecten|GU120027|Beihai, Guangxi province, China
Decatopecten pilica|Mollusca, Bivalvia, Pectinoida, Pectinidae, Decatopecten|GU120028|Beihai, Guangxi province, China
Argopecten irradians|Mollusca, Bivalvia, Pectinoida, Pectinidae, Argopecten|GU120025|Beihai, Guangxi province, China
Argopecten irradians|Mollusca, Bivalvia, Pectinoida, Pectinidae, Argopecten|GU120024|Beihai, Guangxi province, China
Argopecten irradians|Mollusca, Bivalvia, Pectinoida, Pectinidae, Argopecten|GU120023|Zhoushan, Zhejiang province, China
Argopecten irradians|Mollusca, Bivalvia, Pectinoida, Pectinidae, Argopecten|GU120022|Zhoushan, Zhejiang province, China
Argopecten irradians|Mollusca, Bivalvia, Pectinoida, Pectinidae, Argopecten|GU120021|Zhoushan, Zhejiang province, China
Argopecten irradians|Mollusca, Bivalvia, Pectinoida, Pectinidae, Argopecten|GU120020|Zhoushan, Zhejiang province, China
Amusium pleuronectes|Mollusca, Bivalvia, Pectinoida, Pectinidae, Amusium|GU120019|Lingao, Hainan province, China
Amusium pleuronectes|Mollusca, Bivalvia, Pectinoida, Pectinidae, Amusium|GU120018|Lingao, Hainan province, China
Amusium pleuronectes|Mollusca, Bivalvia, Pectinoida, Pectinidae, Amusium|GU120016|Lingao, Hainan province, China
Amusium pleuronectes|Mollusca, Bivalvia, Pectinoida, Pectinidae, Amusium|GU120013|Lingao, Hainan province, China
Amusium pleuronectes|Mollusca, Bivalvia, Pectinoida, Pectinidae, Amusium|GU120012|Lingao, Hainan province, China
Amusium pleuronectes|Mollusca, Bivalvia, Pectinoida, Pectinidae, Amusium|GU120015|Lingao, Hainan province, China
Amusium pleuronectes|Mollusca, Bivalvia, Pectinoida, Pectinidae, Amusium|GU120014|Lingao, Hainan province, China
Amusium pleuronectes|Mollusca, Bivalvia, Pectinoida, Pectinidae, Amusium|GU120017|Lingao, Hainan province, China
Glycymeris rotunda|Mollusca, Bivalvia, Arcoida, Glycymerididae, Glycymeris|AB076934|Kanagawa, Misaki, Japan
Barbatia lacerata|Mollusca, Bivalvia, Arcoida, Arcidae, Barbatia|AB076932|Okinawa, Iriomote island, Okinawa, Japan
Barbatia lacerata|Mollusca, Bivalvia, Arcoida, Arcidae, Barbatia|HQ258836|Weizhou Island, Guangxi province, China
Barbatia lacerata|Mollusca, Bivalvia, Arcoida, Arcidae, Barbatia|HQ258830|Weizhou Island, Guangxi province, China
Barbatia lacerata|Mollusca, Bivalvia, Arcoida, Arcidae, Barbatia|HQ258827|Weizhou Island, Guangxi province, China
Barbatia lacerata|Mollusca, Bivalvia, Arcoida, Arcidae, Barbatia|HQ258833|Weizhou Island, Guangxi province, China
Barbatia lacerata|Mollusca, Bivalvia, Arcoida, Arcidae, Barbatia|HQ258829|Weizhou Island, Guangxi province, China
Barbatia lacerata|Mollusca, Bivalvia, Arcoida, Arcidae, Barbatia|HQ258826|Weizhou Island, Guangxi province, China
Barbatia lacerata|Mollusca, Bivalvia, Arcoida, Arcidae, Barbatia|HQ258835|Weizhou Island, Guangxi province, China

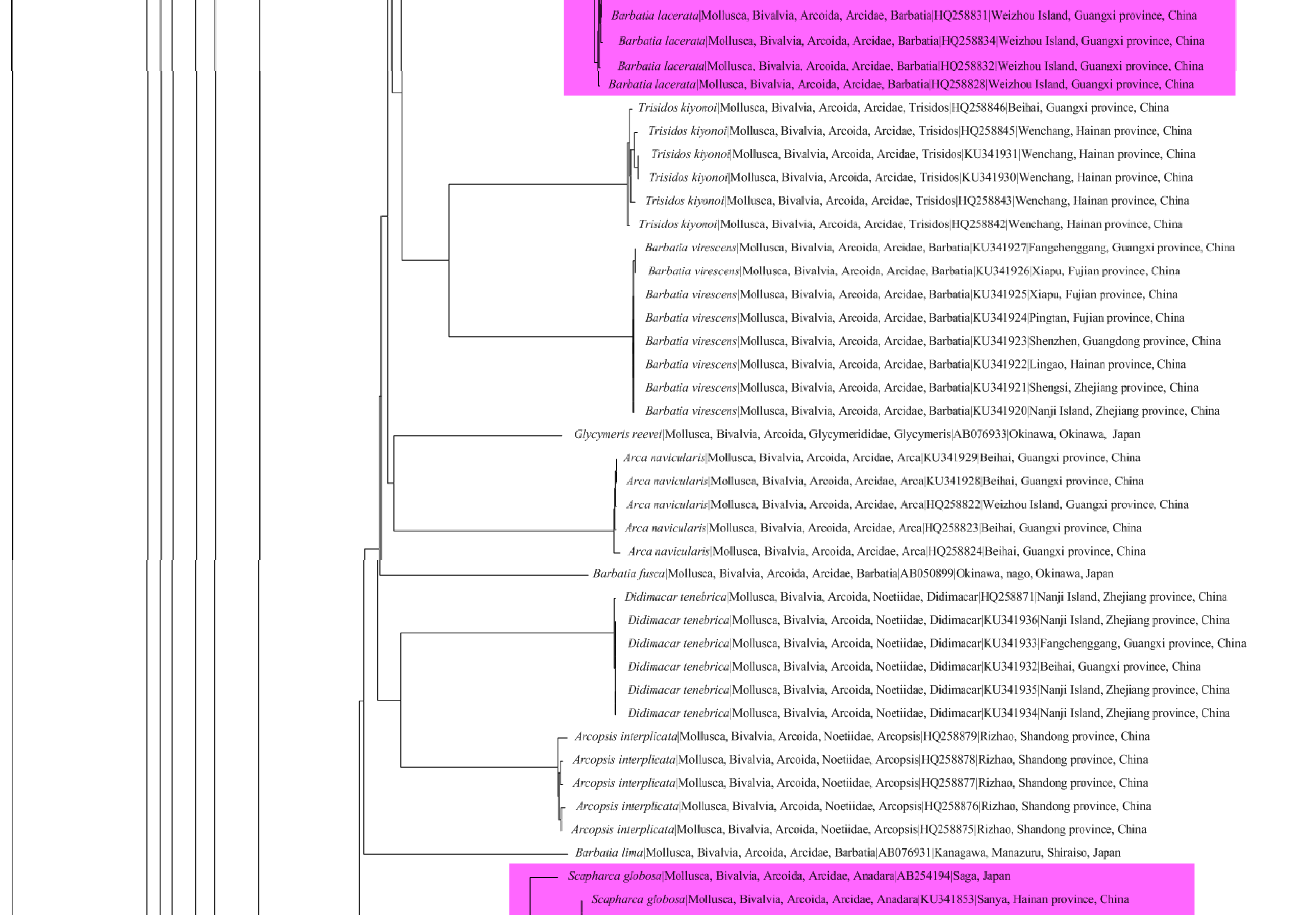
Barbatia lacerata|Mollusca, Bivalvia, Arcoida, Arcidae, Barbatia|HQ258831|Weizhou Island, Guangxi province, China
Barbatia lacerata|Mollusca, Bivalvia, Arcoida, Arcidae, Barbatia|HQ258834|Weizhou Island, Guangxi province, China
Barbatia lacerata|Mollusca, Bivalvia, Arcoida, Arcidae, Barbatia|HQ258832|Weizhou Island, Guangxi province, China
Barbatia lacerata|Mollusca, Bivalvia, Arcoida, Arcidae, Barbatia|HQ258828|Weizhou Island, Guangxi province, China
Trisidos kiyonoi|Mollusca, Bivalvia, Arcoida, Arcidae, Trisidos|HQ258846|Beihai, Guangxi province, China
Trisidos kiyonoi|Mollusca, Bivalvia, Arcoida, Arcidae, Trisidos|HQ258845|Wenchang, Hainan province, China
Trisidos kiyonoi|Mollusca, Bivalvia, Arcoida, Arcidae, Trisidos|KU341931|Wenchang, Hainan province, China
Trisidos kiyonoi|Mollusca, Bivalvia, Arcoida, Arcidae, Trisidos|KU341930|Wenchang, Hainan province, China
Trisidos kiyonoi|Mollusca, Bivalvia, Arcoida, Arcidae, Trisidos|HQ258843|Wenchang, Hainan province, China
Trisidos kiyonoi|Mollusca, Bivalvia, Arcoida, Arcidae, Trisidos|HQ258842|Wenchang, Hainan province, China
Barbatia virescens|Mollusca, Bivalvia, Arcoida, Arcidae, Barbatia|KU341927|Fangchenggang, Guangxi province, China
Barbatia virescens|Mollusca, Bivalvia, Arcoida, Arcidae, Barbatia|KU341926|Xiapu, Fujian province, China
Barbatia virescens|Mollusca, Bivalvia, Arcoida, Arcidae, Barbatia|KU341925|Xiapu, Fujian province, China
Barbatia virescens|Mollusca, Bivalvia, Arcoida, Arcidae, Barbatia|KU341924|Pingtan, Fujian province, China
Barbatia virescens|Mollusca, Bivalvia, Arcoida, Arcidae, Barbatia|KU341923|Shenzhen, Guangdong province, China
Barbatia virescens|Mollusca, Bivalvia, Arcoida, Arcidae, Barbatia|KU341922|Lingao, Hainan province, China
Barbatia virescens|Mollusca, Bivalvia, Arcoida, Arcidae, Barbatia|KU341921|Shengsi, Zhejiang province, China
Barbatia virescens|Mollusca, Bivalvia, Arcoida, Arcidae, Barbatia|KU341920|Nanji Island, Zhejiang province, China
Glycymeris reevei|Mollusca, Bivalvia, Arcoida, Glycymerididae, Glycymeris|AB076933|Okinawa, Okinawa, Japan
Arca navicularis|Mollusca, Bivalvia, Arcoida, Arcidae, Arca|KU341929|Beihai, Guangxi province, China
Arca navicularis|Mollusca, Bivalvia, Arcoida, Arcidae, Arca|KU341928|Beihai, Guangxi province, China
Arca navicularis|Mollusca, Bivalvia, Arcoida, Arcidae, Arca|HQ258822|Weizhou Island, Guangxi province, China
Arca navicularis|Mollusca, Bivalvia, Arcoida, Arcidae, Arca|HQ258823|Beihai, Guangxi province, China
Arca navicularis|Mollusca, Bivalvia, Arcoida, Arcidae, Arca|HQ258824|Beihai, Guangxi province, China
Barbatia fusca|Mollusca, Bivalvia, Arcoida, Arcidae, Barbatia|AB050899|Okinawa, nago, Okinawa, Japan
Didimacar tenebrica|Mollusca, Bivalvia, Arcoida, Noetiidae, Didimacar|HQ258871|Nanji Island, Zhejiang province, China
Didimacar tenebrica|Mollusca, Bivalvia, Arcoida, Noetiidae, Didimacar|KU341936|Nanji Island, Zhejiang province, China
Didimacar tenebrica|Mollusca, Bivalvia, Arcoida, Noetiidae, Didimacar|KU341933|Fangchenggang, Guangxi province, China
Didimacar tenebrica|Mollusca, Bivalvia, Arcoida, Noetiidae, Didimacar|KU341932|Beihai, Guangxi province, China
Didimacar tenebrica|Mollusca, Bivalvia, Arcoida, Noetiidae, Didimacar|KU341935|Nanji Island, Zhejiang province, China
Didimacar tenebrica|Mollusca, Bivalvia, Arcoida, Noetiidae, Didimacar|KU341934|Nanji Island, Zhejiang province, China
Arcopsis interplicata|Mollusca, Bivalvia, Arcoida, Noetiidae, Arcopsis|HQ258879|Rizhao, Shandong province, China
Arcopsis interplicata|Mollusca, Bivalvia, Arcoida, Noetiidae, Arcopsis|HQ258878|Rizhao, Shandong province, China
Arcopsis interplicata|Mollusca, Bivalvia, Arcoida, Noetiidae, Arcopsis|HQ258877|Rizhao, Shandong province, China
Arcopsis interplicata|Mollusca, Bivalvia, Arcoida, Noetiidae, Arcopsis|HQ258876|Rizhao, Shandong province, China
Arcopsis interplicata|Mollusca, Bivalvia, Arcoida, Noetiidae, Arcopsis|HQ258875|Rizhao, Shandong province, China
Barbatia lima|Mollusca, Bivalvia, Arcoida, Arcidae, Barbatia|AB076931|Kanagawa, Manazuru, Shiraiso, Japan
Scapharca globosa|Mollusca, Bivalvia, Arcoida, Arcidae, Anadara|AB254194|Saga, Japan
Scapharca globosa|Mollusca, Bivalvia, Arcoida, Arcidae, Anadara|KU341853|Sanya, Hainan province, China

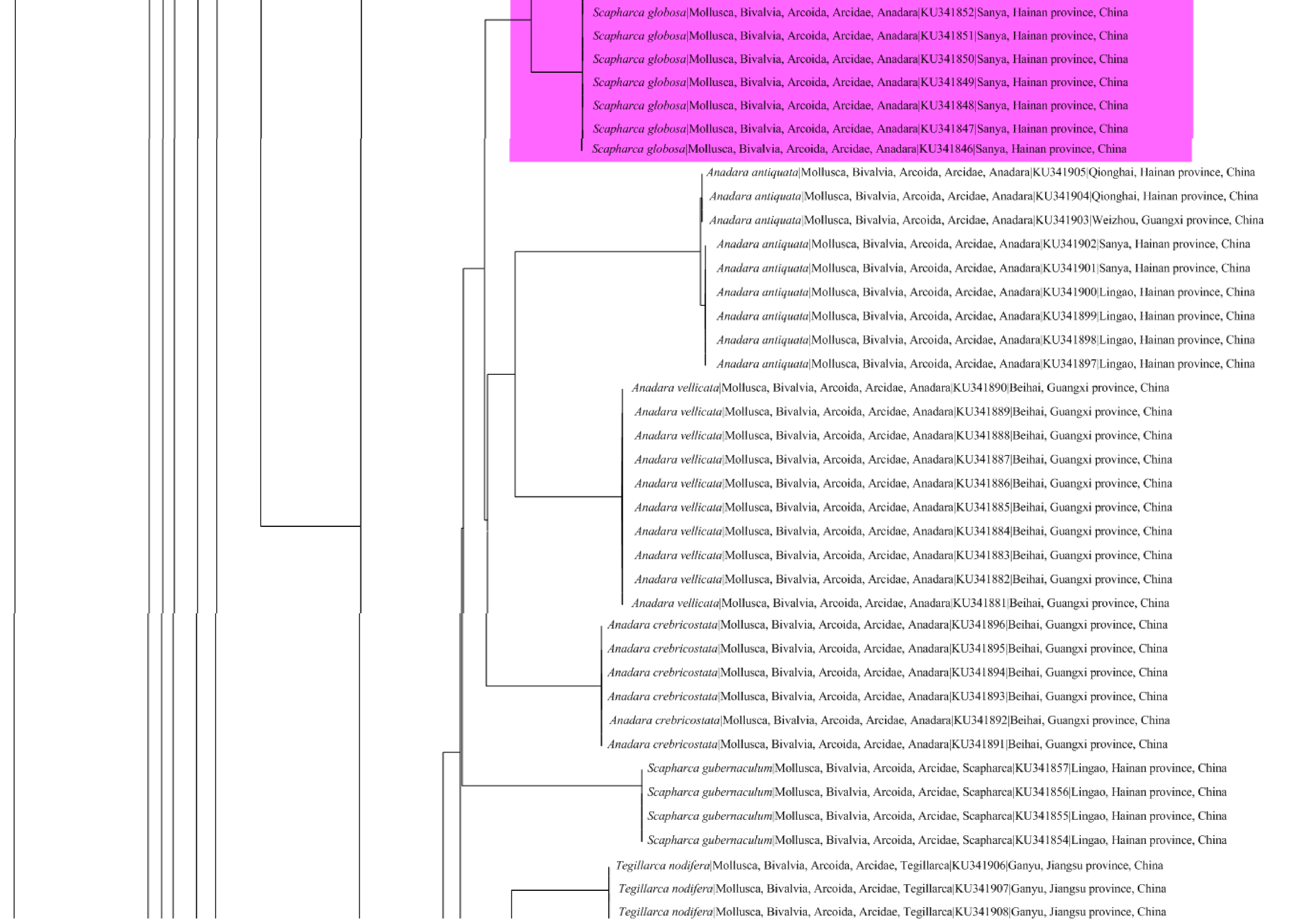
Scapharca globosa|Mollusca, Bivalvia, Arcoida, Arcidae, Anadara|KU341852|Sanya, Hainan province, China
Scapharca globosa|Mollusca, Bivalvia, Arcoida, Arcidae, Anadara|KU341851|Sanya, Hainan province, China
Scapharca globosa|Mollusca, Bivalvia, Arcoida, Arcidae, Anadara|KU341850|Sanya, Hainan province, China
Scapharca globosa|Mollusca, Bivalvia, Arcoida, Arcidae, Anadara|KU341849|Sanya, Hainan province, China
Scapharca globosa|Mollusca, Bivalvia, Arcoida, Arcidae, Anadara|KU341848|Sanya, Hainan province, China
Scapharca globosa|Mollusca, Bivalvia, Arcoida, Arcidae, Anadara|KU341847|Sanya, Hainan province, China
Scapharca globosa|Mollusca, Bivalvia, Arcoida, Arcidae, Anadara|KU341846|Sanya, Hainan province, China
Anadara antiquata|Mollusca, Bivalvia, Arcoida, Arcidae, Anadara|KU341905|Qionghai, Hainan province, China
Anadara antiquata|Mollusca, Bivalvia, Arcoida, Arcidae, Anadara|KU341904|Qionghai, Hainan province, China
Anadara antiquata|Mollusca, Bivalvia, Arcoida, Arcidae, Anadara|KU341903|Weizhou, Guangxi province, China
Anadara antiquata|Mollusca, Bivalvia, Arcoida, Arcidae, Anadara|KU341902|Sanya, Hainan province, China
Anadara antiquata|Mollusca, Bivalvia, Arcoida, Arcidae, Anadara|KU341901|Sanya, Hainan province, China
Anadara antiquata|Mollusca, Bivalvia, Arcoida, Arcidae, Anadara|KU341900|Lingao, Hainan province, China
Anadara antiquata|Mollusca, Bivalvia, Arcoida, Arcidae, Anadara|KU341899|Lingao, Hainan province, China
Anadara antiquata|Mollusca, Bivalvia, Arcoida, Arcidae, Anadara|KU341898|Lingao, Hainan province, China
Anadara antiquata|Mollusca, Bivalvia, Arcoida, Arcidae, Anadara|KU341897|Lingao, Hainan province, China
Anadara vellicata|Mollusca, Bivalvia, Arcoida, Arcidae, Anadara|KU341890|Beihai, Guangxi province, China
Anadara vellicata|Mollusca, Bivalvia, Arcoida, Arcidae, Anadara|KU341889|Beihai, Guangxi province, China
Anadara vellicata|Mollusca, Bivalvia, Arcoida, Arcidae, Anadara|KU341888|Beihai, Guangxi province, China
Anadara vellicata|Mollusca, Bivalvia, Arcoida, Arcidae, Anadara|KU341887|Beihai, Guangxi province, China
Anadara vellicata|Mollusca, Bivalvia, Arcoida, Arcidae, Anadara|KU341886|Beihai, Guangxi province, China
Anadara vellicata|Mollusca, Bivalvia, Arcoida, Arcidae, Anadara|KU341885|Beihai, Guangxi province, China
Anadara vellicata|Mollusca, Bivalvia, Arcoida, Arcidae, Anadara|KU341884|Beihai, Guangxi province, China
Anadara vellicata|Mollusca, Bivalvia, Arcoida, Arcidae, Anadara|KU341883|Beihai, Guangxi province, China
Anadara vellicata|Mollusca, Bivalvia, Arcoida, Arcidae, Anadara|KU341882|Beihai, Guangxi province, China
Anadara vellicata|Mollusca, Bivalvia, Arcoida, Arcidae, Anadara|KU341881|Beihai, Guangxi province, China
Anadara crebricostata|Mollusca, Bivalvia, Arcoida, Arcidae, Anadara|KU341896|Beihai, Guangxi province, China
Anadara crebricostata|Mollusca, Bivalvia, Arcoida, Arcidae, Anadara|KU341895|Beihai, Guangxi province, China
Anadara crebricostata|Mollusca, Bivalvia, Arcoida, Arcidae, Anadara|KU341894|Beihai, Guangxi province, China
Anadara crebricostata|Mollusca, Bivalvia, Arcoida, Arcidae, Anadara|KU341893|Beihai, Guangxi province, China
Anadara crebricostata|Mollusca, Bivalvia, Arcoida, Arcidae, Anadara|KU341892|Beihai, Guangxi province, China
Anadara crebricostata|Mollusca, Bivalvia, Arcoida, Arcidae, Anadara|KU341891|Beihai, Guangxi province, China
Scapharca gubernaculum|Mollusca, Bivalvia, Arcoida, Arcidae, Scapharca|KU341857|Lingao, Hainan province, China
Scapharca gubernaculum|Mollusca, Bivalvia, Arcoida, Arcidae, Scapharca|KU341856|Lingao, Hainan province, China
Scapharca gubernaculum|Mollusca, Bivalvia, Arcoida, Arcidae, Scapharca|KU341855|Lingao, Hainan province, China
Scapharca gubernaculum|Mollusca, Bivalvia, Arcoida, Arcidae, Scapharca|KU341854|Lingao, Hainan province, China
Tegillarca nodifera|Mollusca, Bivalvia, Arcoida, Arcidae, Tegillarca|KU341906|Ganyu, Jiangsu province, China
Tegillarca nodifera|Mollusca, Bivalvia, Arcoida, Arcidae, Tegillarca|KU341907|Ganyu, Jiangsu province, China
Tegillarca nodifera|Mollusca, Bivalvia, Arcoida, Arcidae, Tegillarca|KU341908|Ganyu, Jiangsu province, China

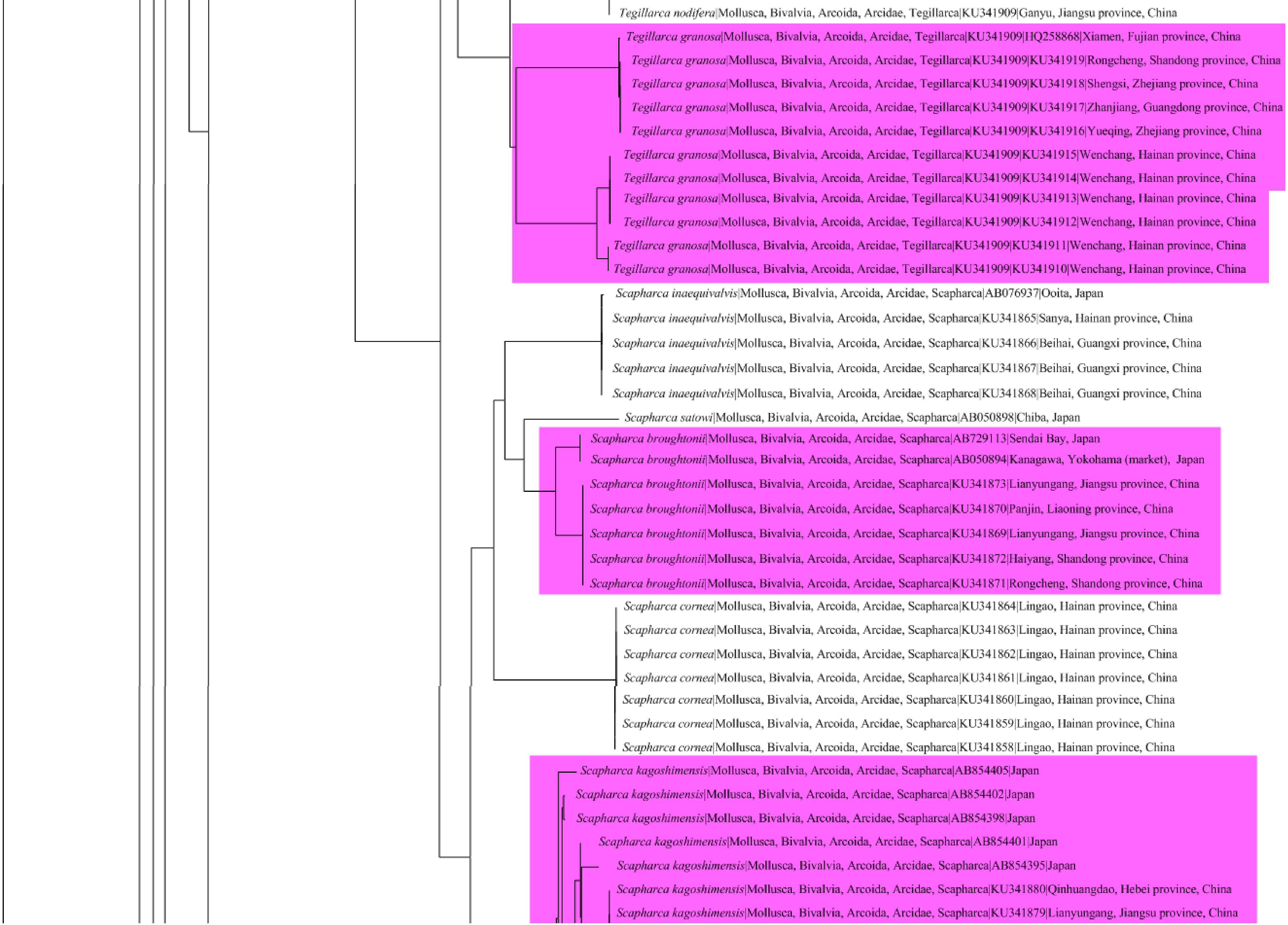
Tegillarca nodifera|Mollusca, Bivalvia, Arcoida, Arcidae, Tegillarca|KU341909|Ganyu, Jiangsu province, China
Tegillarca granosa|Mollusca, Bivalvia, Arcoida, Arcidae, Tegillarca|KU341909|HQ258868|Xiamen, Fujian province, China
Tegillarca granosa|Mollusca, Bivalvia, Arcoida, Arcidae, Tegillarca|KU341909|KU341919|Rongcheng, Shandong province, China
Tegillarca granosa|Mollusca, Bivalvia, Arcoida, Arcidae, Tegillarca|KU341909|KU341918|Shengsi, Zhejiang province, China
Tegillarca granosa|Mollusca, Bivalvia, Arcoida, Arcidae, Tegillarca|KU341909|KU341917|Zhanjiang, Guangdong province, China
Tegillarca granosa|Mollusca, Bivalvia, Arcoida, Arcidae, Tegillarca|KU341909|KU341916|Yueqing, Zhejiang province, China
Tegillarca granosa|Mollusca, Bivalvia, Arcoida, Arcidae, Tegillarca|KU341909|KU341915|Wenchang, Hainan province, China
Tegillarca granosa|Mollusca, Bivalvia, Arcoida, Arcidae, Tegillarca|KU341909|KU341914|Wenchang, Hainan province, China
Tegillarca granosa|Mollusca, Bivalvia, Arcoida, Arcidae, Tegillarca|KU341909|KU341913|Wenchang, Hainan province, China
Tegillarca granosa|Mollusca, Bivalvia, Arcoida, Arcidae, Tegillarca|KU341909|KU341912|Wenchang, Hainan province, China
Tegillarca granosa|Mollusca, Bivalvia, Arcoida, Arcidae, Tegillarca|KU341909|KU341911|Wenchang, Hainan province, China
Tegillarca granosa|Mollusca, Bivalvia, Arcoida, Arcidae, Tegillarca|KU341909|KU341910|Wenchang, Hainan province, China
Scapharca inaequivalvis|Mollusca, Bivalvia, Arcoida, Arcidae, Scapharca|AB076937|Ooita, Japan
Scapharca inaequivalvis|Mollusca, Bivalvia, Arcoida, Arcidae, Scapharca|KU341865|Sanya, Hainan province, China
Scapharca inaequivalvis|Mollusca, Bivalvia, Arcoida, Arcidae, Scapharca|KU341866|Beihai, Guangxi province, China
Scapharca inaequivalvis|Mollusca, Bivalvia, Arcoida, Arcidae, Scapharca|KU341867|Beihai, Guangxi province, China
Scapharca inaequivalvis|Mollusca, Bivalvia, Arcoida, Arcidae, Scapharca|KU341868|Beihai, Guangxi province, China
Scapharca satowi|Mollusca, Bivalvia, Arcoida, Arcidae, Scapharca|AB050898|Chiba, Japan
Scapharca broughtonii|Mollusca, Bivalvia, Arcoida, Arcidae, Scapharca|AB729113|Sendai Bay, Japan
Scapharca broughtonii|Mollusca, Bivalvia, Arcoida, Arcidae, Scapharca|AB050894|Kanagawa, Yokohama (market), Japan
Scapharca broughtonii|Mollusca, Bivalvia, Arcoida, Arcidae, Scapharca|KU341873|Lianyungang, Jiangsu province, China
Scapharca broughtonii|Mollusca, Bivalvia, Arcoida, Arcidae, Scapharca|KU341870|Panjin, Liaoning province, China
Scapharca broughtonii|Mollusca, Bivalvia, Arcoida, Arcidae, Scapharca|KU341869|Lianyungang, Jiangsu province, China
Scapharca broughtonii|Mollusca, Bivalvia, Arcoida, Arcidae, Scapharca|KU341872|Haiyang, Shandong province, China
Scapharca broughtonii|Mollusca, Bivalvia, Arcoida, Arcidae, Scapharca|KU341871|Rongcheng, Shandong province, China
Scapharca cornea|Mollusca, Bivalvia, Arcoida, Arcidae, Scapharca|KU341864|Lingao, Hainan province, China
Scapharca cornea|Mollusca, Bivalvia, Arcoida, Arcidae, Scapharca|KU341863|Lingao, Hainan province, China
Scapharca cornea|Mollusca, Bivalvia, Arcoida, Arcidae, Scapharca|KU341862|Lingao, Hainan province, China
Scapharca cornea|Mollusca, Bivalvia, Arcoida, Arcidae, Scapharca|KU341861|Lingao, Hainan province, China
Scapharca cornea|Mollusca, Bivalvia, Arcoida, Arcidae, Scapharca|KU341860|Lingao, Hainan province, China
Scapharca cornea|Mollusca, Bivalvia, Arcoida, Arcidae, Scapharca|KU341859|Lingao, Hainan province, China
Scapharca cornea|Mollusca, Bivalvia, Arcoida, Arcidae, Scapharca|KU341858|Lingao, Hainan province, China
Scapharca kagoshimensis|Mollusca, Bivalvia, Arcoida, Arcidae, Scapharca|AB854405|Japan
Scapharca kagoshimensis|Mollusca, Bivalvia, Arcoida, Arcidae, Scapharca|AB854402|Japan
Scapharca kagoshimensis|Mollusca, Bivalvia, Arcoida, Arcidae, Scapharca|AB854398|Japan
Scapharca kagoshimensis|Mollusca, Bivalvia, Arcoida, Arcidae, Scapharca|AB854401|Japan
Scapharca kagoshimensis|Mollusca, Bivalvia, Arcoida, Arcidae, Scapharca|AB854395|Japan
Scapharca kagoshimensis|Mollusca, Bivalvia, Arcoida, Arcidae, Scapharca|KU341880|Qinhuangdao, Hebei province, China
Scapharca kagoshimensis|Mollusca, Bivalvia, Arcoida, Arcidae, Scapharca|KU341879|Lianyungang, Jiangsu province, China

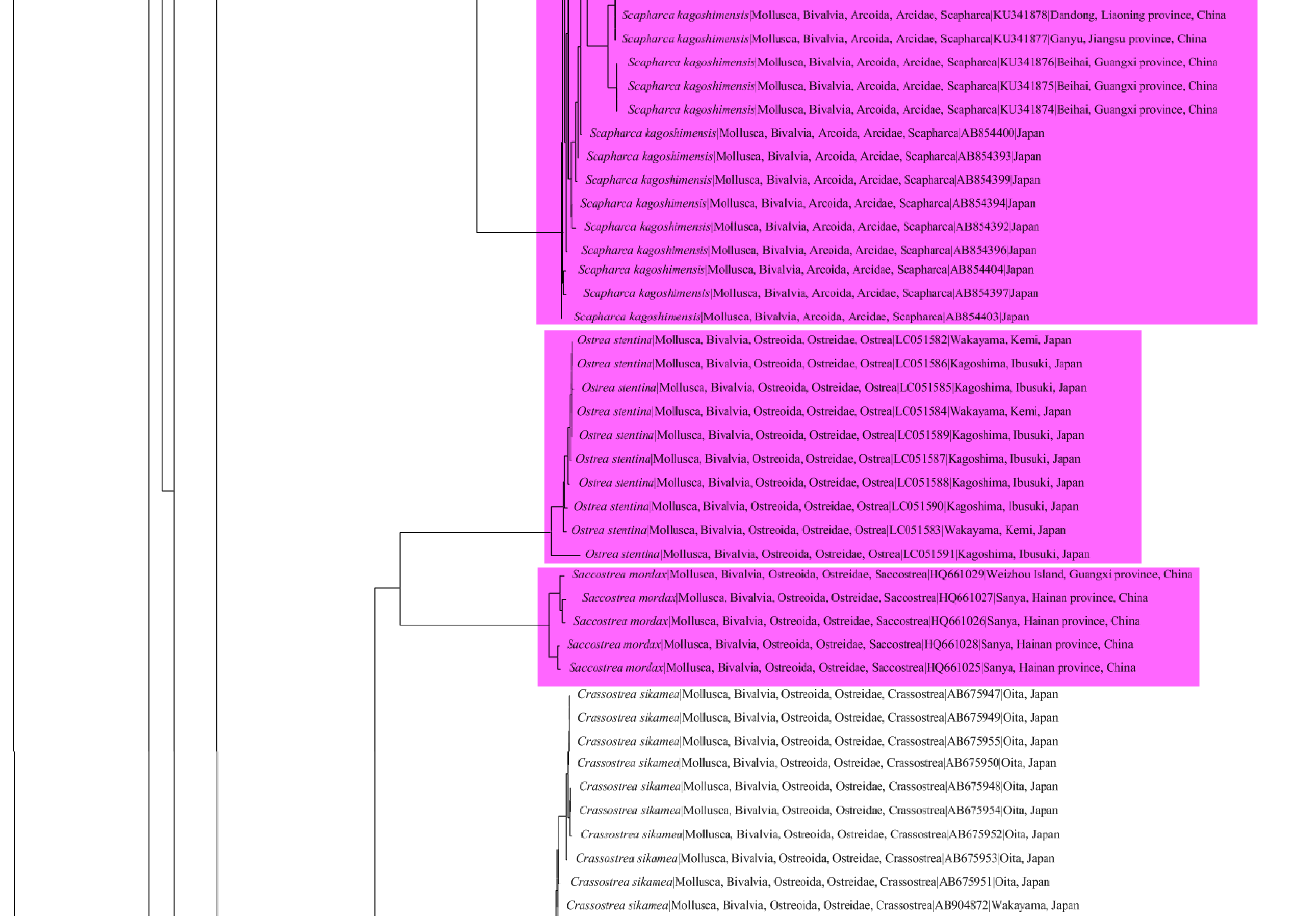
Scapharca kagoshimensis|Mollusca, Bivalvia, Arcoida, Arcidae, Scapharca|KU341878|Dandong, Liaoning province, China
Scapharca kagoshimensis|Mollusca, Bivalvia, Arcoida, Arcidae, Scapharca|KU341877|Ganyu, Jiangsu province, China
Scapharca kagoshimensis|Mollusca, Bivalvia, Arcoida, Arcidae, Scapharca|KU341876|Beihai, Guangxi province, China
Scapharca kagoshimensis|Mollusca, Bivalvia, Arcoida, Arcidae, Scapharca|KU341875|Beihai, Guangxi province, China
Scapharca kagoshimensis|Mollusca, Bivalvia, Arcoida, Arcidae, Scapharca|KU341874|Beihai, Guangxi province, China
Scapharca kagoshimensis|Mollusca, Bivalvia, Arcoida, Arcidae, Scapharca|AB854400|Japan
Scapharca kagoshimensis|Mollusca, Bivalvia, Arcoida, Arcidae, Scapharca|AB854393|Japan
Scapharca kagoshimensis|Mollusca, Bivalvia, Arcoida, Arcidae, Scapharca|AB854399|Japan
Scapharca kagoshimensis|Mollusca, Bivalvia, Arcoida, Arcidae, Scapharca|AB854394|Japan
Scapharca kagoshimensis|Mollusca, Bivalvia, Arcoida, Arcidae, Scapharca|AB854392|Japan
Scapharca kagoshimensis|Mollusca, Bivalvia, Arcoida, Arcidae, Scapharca|AB854396|Japan
Scapharca kagoshimensis|Mollusca, Bivalvia, Arcoida, Arcidae, Scapharca|AB854404|Japan
Scapharca kagoshimensis|Mollusca, Bivalvia, Arcoida, Arcidae, Scapharca|AB854397|Japan
Scapharca kagoshimensis|Mollusca, Bivalvia, Arcoida, Arcidae, Scapharca|AB854403|Japan
Ostrea stentina|Mollusca, Bivalvia, Ostreoida, Ostreidae, Ostrea|LC051582|Wakayama, Kemi, Japan
Ostrea stentina|Mollusca, Bivalvia, Ostreoida, Ostreidae, Ostrea|LC051586|Kagoshima, Ibusuki, Japan
Ostrea stentina|Mollusca, Bivalvia, Ostreoida, Ostreidae, Ostrea|LC051585|Kagoshima, Ibusuki, Japan
Ostrea stentina|Mollusca, Bivalvia, Ostreoida, Ostreidae, Ostrea|LC051584|Wakayama, Kemi, Japan
Ostrea stentina|Mollusca, Bivalvia, Ostreoida, Ostreidae, Ostrea|LC051589|Kagoshima, Ibusuki, Japan
Ostrea stentina|Mollusca, Bivalvia, Ostreoida, Ostreidae, Ostrea|LC051587|Kagoshima, Ibusuki, Japan
Ostrea stentina|Mollusca, Bivalvia, Ostreoida, Ostreidae, Ostrea|LC051588|Kagoshima, Ibusuki, Japan
Ostrea stentina|Mollusca, Bivalvia, Ostreoida, Ostreidae, Ostrea|LC051590|Kagoshima, Ibusuki, Japan
Ostrea stentina|Mollusca, Bivalvia, Ostreoida, Ostreidae, Ostrea|LC051583|Wakayama, Kemi, Japan
Ostrea stentina|Mollusca, Bivalvia, Ostreoida, Ostreidae, Ostrea|LC051591|Kagoshima, Ibusuki, Japan
Saccostrea mordax|Mollusca, Bivalvia, Ostreoida, Ostreidae, Saccostrea|HQ661029|Weizhou Island, Guangxi province, China
Saccostrea mordax|Mollusca, Bivalvia, Ostreoida, Ostreidae, Saccostrea|HQ661027|Sanya, Hainan province, China
Saccostrea mordax|Mollusca, Bivalvia, Ostreoida, Ostreidae, Saccostrea|HQ661026|Sanya, Hainan province, China
Saccostrea mordax|Mollusca, Bivalvia, Ostreoida, Ostreidae, Saccostrea|HQ661028|Sanya, Hainan province, China
Saccostrea mordax|Mollusca, Bivalvia, Ostreoida, Ostreidae, Saccostrea|HQ661025|Sanya, Hainan province, China
Crassostrea sikamea|Mollusca, Bivalvia, Ostreoida, Ostreidae, Crassostrea|AB675947|Oita, Japan
Crassostrea sikamea|Mollusca, Bivalvia, Ostreoida, Ostreidae, Crassostrea|AB675949|Oita, Japan
Crassostrea sikamea|Mollusca, Bivalvia, Ostreoida, Ostreidae, Crassostrea|AB675955|Oita, Japan
Crassostrea sikamea|Mollusca, Bivalvia, Ostreoida, Ostreidae, Crassostrea|AB675950|Oita, Japan
Crassostrea sikamea|Mollusca, Bivalvia, Ostreoida, Ostreidae, Crassostrea|AB675948|Oita, Japan
Crassostrea sikamea|Mollusca, Bivalvia, Ostreoida, Ostreidae, Crassostrea|AB675954|Oita, Japan
Crassostrea sikamea|Mollusca, Bivalvia, Ostreoida, Ostreidae, Crassostrea|AB675952|Oita, Japan
Crassostrea sikamea|Mollusca, Bivalvia, Ostreoida, Ostreidae, Crassostrea|AB675953|Oita, Japan
Crassostrea sikamea|Mollusca, Bivalvia, Ostreoida, Ostreidae, Crassostrea|AB675951|Oita, Japan
Crassostrea sikamea|Mollusca, Bivalvia, Ostreoida, Ostreidae, Crassostrea|AB904872|Wakayama, Japan

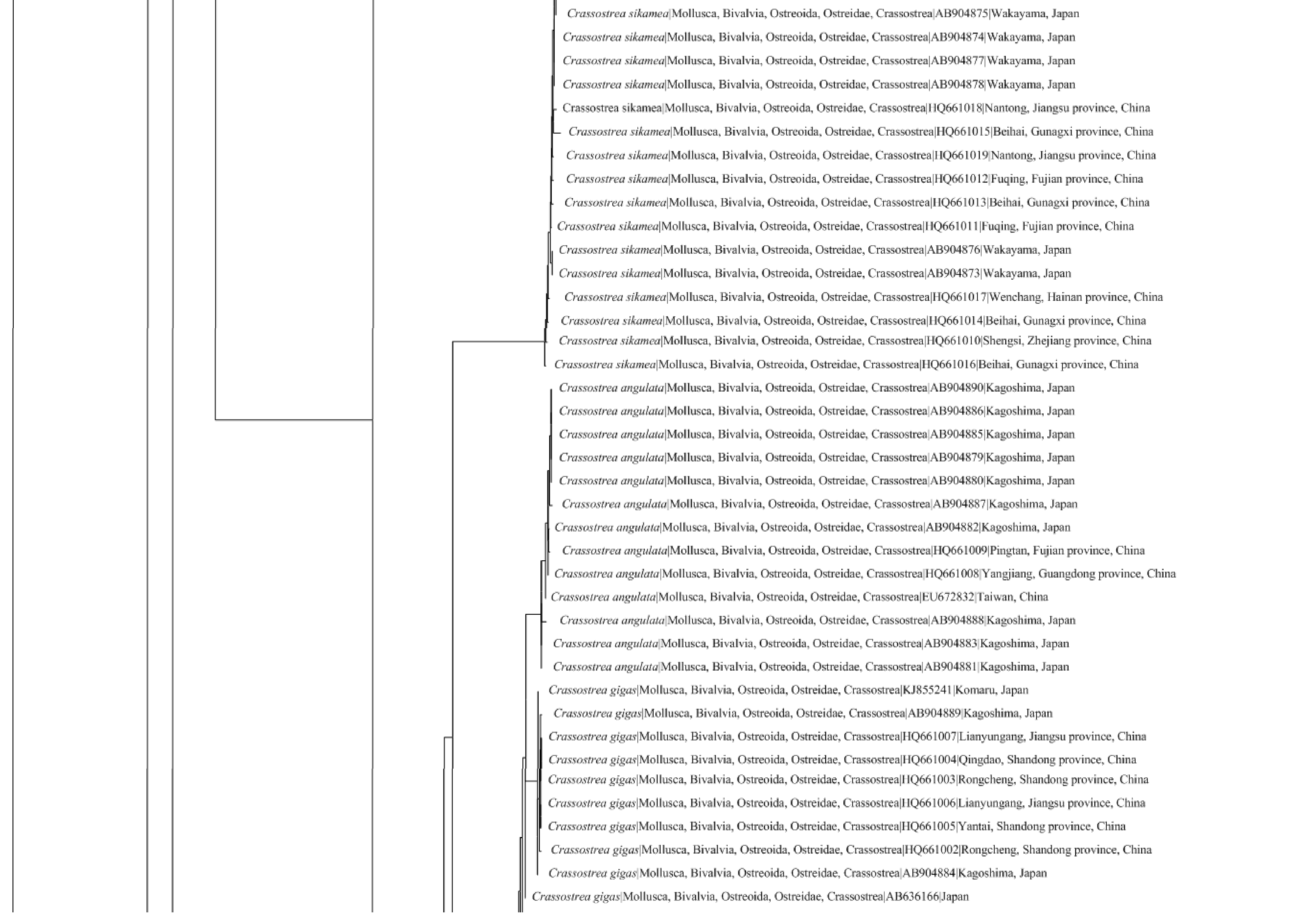

Crassostrea sikamea|Mollusca, Bivalvia, Ostreoida, Ostreidae, Crassostrea|AB904875|Wakayama, Japan
Crassostrea sikamea|Mollusca, Bivalvia, Ostreoida, Ostreidae, Crassostrea|AB904874|Wakayama, Japan
Crassostrea sikamea|Mollusca, Bivalvia, Ostreoida, Ostreidae, Crassostrea|AB904877|Wakayama, Japan
Crassostrea sikamea|Mollusca, Bivalvia, Ostreoida, Ostreidae, Crassostrea|AB904878|Wakayama, Japan
Crassostrea sikamea|Mollusca, Bivalvia, Ostreoida, Ostreidae, Crassostrea|HQ661018|Nantong, Jiangsu province, China
Crassostrea sikamea|Mollusca, Bivalvia, Ostreoida, Ostreidae, Crassostrea|HQ661015|Beihai, Gunagxi province, China
Crassostrea sikamea|Mollusca, Bivalvia, Ostreoida, Ostreidae, Crassostrea|HQ661019|Nantong, Jiangsu province, China
Crassostrea sikamea|Mollusca, Bivalvia, Ostreoida, Ostreidae, Crassostrea|HQ661012|Fuqing, Fujian province, China
Crassostrea sikamea|Mollusca, Bivalvia, Ostreoida, Ostreidae, Crassostrea|HQ661013|Beihai, Gunagxi province, China
Crassostrea sikamea|Mollusca, Bivalvia, Ostreoida, Ostreidae, Crassostrea|HQ661011|Fuqing, Fujian province, China
Crassostrea sikamea|Mollusca, Bivalvia, Ostreoida, Ostreidae, Crassostrea|AB904876|Wakayama, Japan
Crassostrea sikamea|Mollusca, Bivalvia, Ostreoida, Ostreidae, Crassostrea|AB904873|Wakayama, Japan
Crassostrea sikamea|Mollusca, Bivalvia, Ostreoida, Ostreidae, Crassostrea|HQ661017|Wenchang, Hainan province, China
Crassostrea sikamea|Mollusca, Bivalvia, Ostreoida, Ostreidae, Crassostrea|HQ661014|Beihai, Gunagxi province, China
Crassostrea sikamea|Mollusca, Bivalvia, Ostreoida, Ostreidae, Crassostrea|HQ661010|Shengsi, Zhejiang province, China
Crassostrea sikamea|Mollusca, Bivalvia, Ostreoida, Ostreidae, Crassostrea|HQ661016|Beihai, Gunagxi province, China
Crassostrea angulata|Mollusca, Bivalvia, Ostreoida, Ostreidae, Crassostrea|AB904890|Kagoshima, Japan
Crassostrea angulata|Mollusca, Bivalvia, Ostreoida, Ostreidae, Crassostrea|AB904886|Kagoshima, Japan
Crassostrea angulata|Mollusca, Bivalvia, Ostreoida, Ostreidae, Crassostrea|AB904885|Kagoshima, Japan
Crassostrea angulata|Mollusca, Bivalvia, Ostreoida, Ostreidae, Crassostrea|AB904879|Kagoshima, Japan
Crassostrea angulata|Mollusca, Bivalvia, Ostreoida, Ostreidae, Crassostrea|AB904880|Kagoshima, Japan
Crassostrea angulata|Mollusca, Bivalvia, Ostreoida, Ostreidae, Crassostrea|AB904887|Kagoshima, Japan
Crassostrea angulata|Mollusca, Bivalvia, Ostreoida, Ostreidae, Crassostrea|AB904882|Kagoshima, Japan
Crassostrea angulata|Mollusca, Bivalvia, Ostreoida, Ostreidae, Crassostrea|HQ661009|Pingtan, Fujian province, China
Crassostrea angulata|Mollusca, Bivalvia, Ostreoida, Ostreidae, Crassostrea|HQ661008|Yangjiang, Guangdong province, China
Crassostrea angulata|Mollusca, Bivalvia, Ostreoida, Ostreidae, Crassostrea|EU672832|Taiwan, China
Crassostrea angulata|Mollusca, Bivalvia, Ostreoida, Ostreidae, Crassostrea|AB904888|Kagoshima, Japan
Crassostrea angulata|Mollusca, Bivalvia, Ostreoida, Ostreidae, Crassostrea|AB904883|Kagoshima, Japan
Crassostrea angulata|Mollusca, Bivalvia, Ostreoida, Ostreidae, Crassostrea|AB904881|Kagoshima, Japan
Crassostrea gigas|Mollusca, Bivalvia, Ostreoida, Ostreidae, Crassostrea|KJ855241|Komaru, Japan
Crassostrea gigas|Mollusca, Bivalvia, Ostreoida, Ostreidae, Crassostrea|AB904889|Kagoshima, Japan
Crassostrea gigas|Mollusca, Bivalvia, Ostreoida, Ostreidae, Crassostrea|HQ661007|Lianyungang, Jiangsu province, China
Crassostrea gigas|Mollusca, Bivalvia, Ostreoida, Ostreidae, Crassostrea|HQ661004|Qingdao, Shandong province, China
Crassostrea gigas|Mollusca, Bivalvia, Ostreoida, Ostreidae, Crassostrea|HQ661003|Rongcheng, Shandong province, China
Crassostrea gigas|Mollusca, Bivalvia, Ostreoida, Ostreidae, Crassostrea|HQ661006|Lianyungang, Jiangsu province, China
Crassostrea gigas|Mollusca, Bivalvia, Ostreoida, Ostreidae, Crassostrea|HQ661005|Yantai, Shandong province, China
Crassostrea gigas|Mollusca, Bivalvia, Ostreoida, Ostreidae, Crassostrea|HQ661002|Rongcheng, Shandong province, China
Crassostrea gigas|Mollusca, Bivalvia, Ostreoida, Ostreidae, Crassostrea|AB904884|Kagoshima, Japan
Crassostrea gigas|Mollusca, Bivalvia, Ostreoida, Ostreidae, Crassostrea|AB636166|Japan

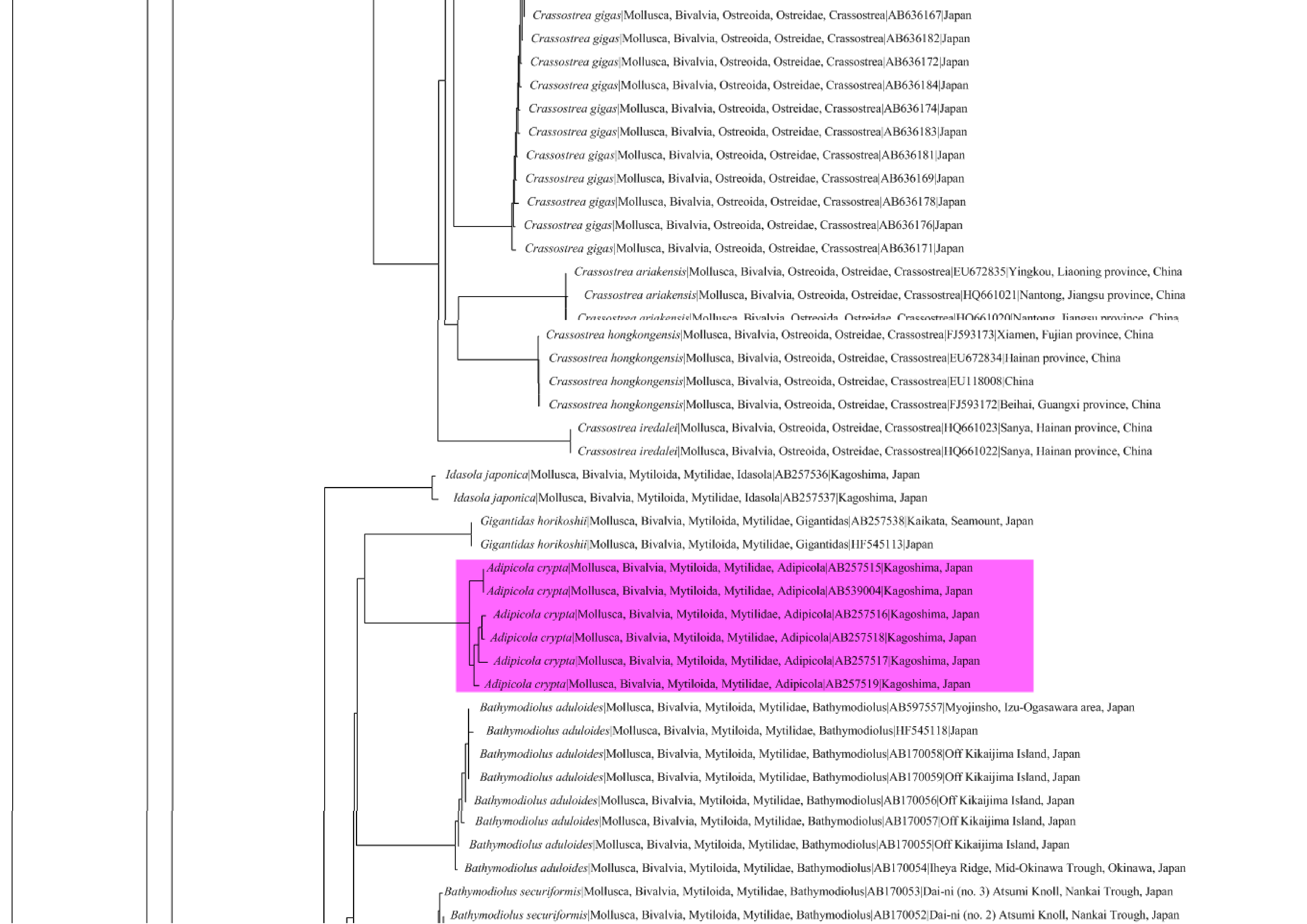

Crassostrea gigas|Mollusca, Bivalvia, Ostreoida, Ostreidae, Crassostrea|AB636167|Japan
Crassostrea gigas|Mollusca, Bivalvia, Ostreoida, Ostreidae, Crassostrea|AB636182|Japan
Crassostrea gigas|Mollusca, Bivalvia, Ostreoida, Ostreidae, Crassostrea|AB636172|Japan
Crassostrea gigas|Mollusca, Bivalvia, Ostreoida, Ostreidae, Crassostrea|AB636184|Japan
Crassostrea gigas|Mollusca, Bivalvia, Ostreoida, Ostreidae, Crassostrea|AB636174|Japan
Crassostrea gigas|Mollusca, Bivalvia, Ostreoida, Ostreidae, Crassostrea|AB636183|Japan
Crassostrea gigas|Mollusca, Bivalvia, Ostreoida, Ostreidae, Crassostrea|AB636181|Japan
Crassostrea gigas|Mollusca, Bivalvia, Ostreoida, Ostreidae, Crassostrea|AB636169|Japan
Crassostrea gigas|Mollusca, Bivalvia, Ostreoida, Ostreidae, Crassostrea|AB636178|Japan
Crassostrea gigas|Mollusca, Bivalvia, Ostreoida, Ostreidae, Crassostrea|AB636176|Japan
Crassostrea gigas|Mollusca, Bivalvia, Ostreoida, Ostreidae, Crassostrea|AB636171|Japan
Crassostrea ariakensis|Mollusca, Bivalvia, Ostreoida, Ostreidae, Crassostrea|EU672835|Yingkou, Liaoning province, China
Crassostrea ariakensis|Mollusca, Bivalvia, Ostreoida, Ostreidae, Crassostrea|HQ661021|Nantong, Jiangsu province, China
Crassostrea ariakensis|Mollusca, Bivalvia, Ostreoida, Ostreidae, Crassostrea|HQ661020|Nantong, Jiangsu province, China
Crassostrea hongkongensis|Mollusca, Bivalvia, Ostreoida, Ostreidae, Crassostrea|FJ593173|Xiamen, Fujian province, China
Crassostrea hongkongensis|Mollusca, Bivalvia, Ostreoida, Ostreidae, Crassostrea|EU672834|Hainan province, China
Crassostrea hongkongensis|Mollusca, Bivalvia, Ostreoida, Ostreidae, Crassostrea|EU118008|China
Crassostrea hongkongensis|Mollusca, Bivalvia, Ostreoida, Ostreidae, Crassostrea|FJ593172|Beihai, Guangxi province, China
Crassostrea iredalei|Mollusca, Bivalvia, Ostreoida, Ostreidae, Crassostrea|HQ661023|Sanya, Hainan province, China
Crassostrea iredalei|Mollusca, Bivalvia, Ostreoida, Ostreidae, Crassostrea|HQ661022|Sanya, Hainan province, China
Idasola japonica|Mollusca, Bivalvia, Mytiloida, Mytilidae, Idasola|AB257536|Kagoshima, Japan
Idasola japonica|Mollusca, Bivalvia, Mytiloida, Mytilidae, Idasola|AB257537|Kagoshima, Japan
Gigantidas horikoshii|Mollusca, Bivalvia, Mytiloida, Mytilidae, Gigantidas|AB257538|Kaikata, Seamount, Japan
Gigantidas horikoshii|Mollusca, Bivalvia, Mytiloida, Mytilidae, Gigantidas|HF545113|Japan
Adipicola crypta|Mollusca, Bivalvia, Mytiloida, Mytilidae, Adipicola|AB257515|Kagoshima, Japan
Adipicola crypta|Mollusca, Bivalvia, Mytiloida, Mytilidae, Adipicola|AB539004|Kagoshima, Japan
Adipicola crypta|Mollusca, Bivalvia, Mytiloida, Mytilidae, Adipicola|AB257516|Kagoshima, Japan
Adipicola crypta|Mollusca, Bivalvia, Mytiloida, Mytilidae, Adipicola|AB257518|Kagoshima, Japan
Adipicola crypta|Mollusca, Bivalvia, Mytiloida, Mytilidae, Adipicola|AB257517|Kagoshima, Japan
Adipicola crypta|Mollusca, Bivalvia, Mytiloida, Mytilidae, Adipicola|AB257519|Kagoshima, Japan
Bathymodiolus aduloides|Mollusca, Bivalvia, Mytiloida, Mytilidae, Bathymodiolus|AB597557|Myojinsho, Izu-Ogasawara area, Japan
Bathymodiolus aduloides|Mollusca, Bivalvia, Mytiloida, Mytilidae, Bathymodiolus|HF545118|Japan
Bathymodiolus aduloides|Mollusca, Bivalvia, Mytiloida, Mytilidae, Bathymodiolus|AB170058|Off Kikaijima Island, Japan
Bathymodiolus aduloides|Mollusca, Bivalvia, Mytiloida, Mytilidae, Bathymodiolus|AB170059|Off Kikaijima Island, Japan
Bathymodiolus aduloides|Mollusca, Bivalvia, Mytiloida, Mytilidae, Bathymodiolus|AB170056|Off Kikaijima Island, Japan
Bathymodiolus aduloides|Mollusca, Bivalvia, Mytiloida, Mytilidae, Bathymodiolus|AB170057|Off Kikaijima Island, Japan
Bathymodiolus aduloides|Mollusca, Bivalvia, Mytiloida, Mytilidae, Bathymodiolus|AB170055|Off Kikaijima Island, Japan
Bathymodiolus aduloides|Mollusca, Bivalvia, Mytiloida, Mytilidae, Bathymodiolus|AB170054|Iheya Ridge, Mid-Okinawa Trough, Okinawa, Japan
Bathymodiolus securiformis|Mollusca, Bivalvia, Mytiloida, Mytilidae, Bathymodiolus|AB170053|Dai-ni (no. 3) Atsumi Knoll, Nankai Trough, Japan
Bathymodiolus securiformis|Mollusca, Bivalvia, Mytiloida, Mytilidae, Bathymodiolus|AB170052|Dai-ni (no. 2) Atsumi Knoll, Nankai Trough, Japan

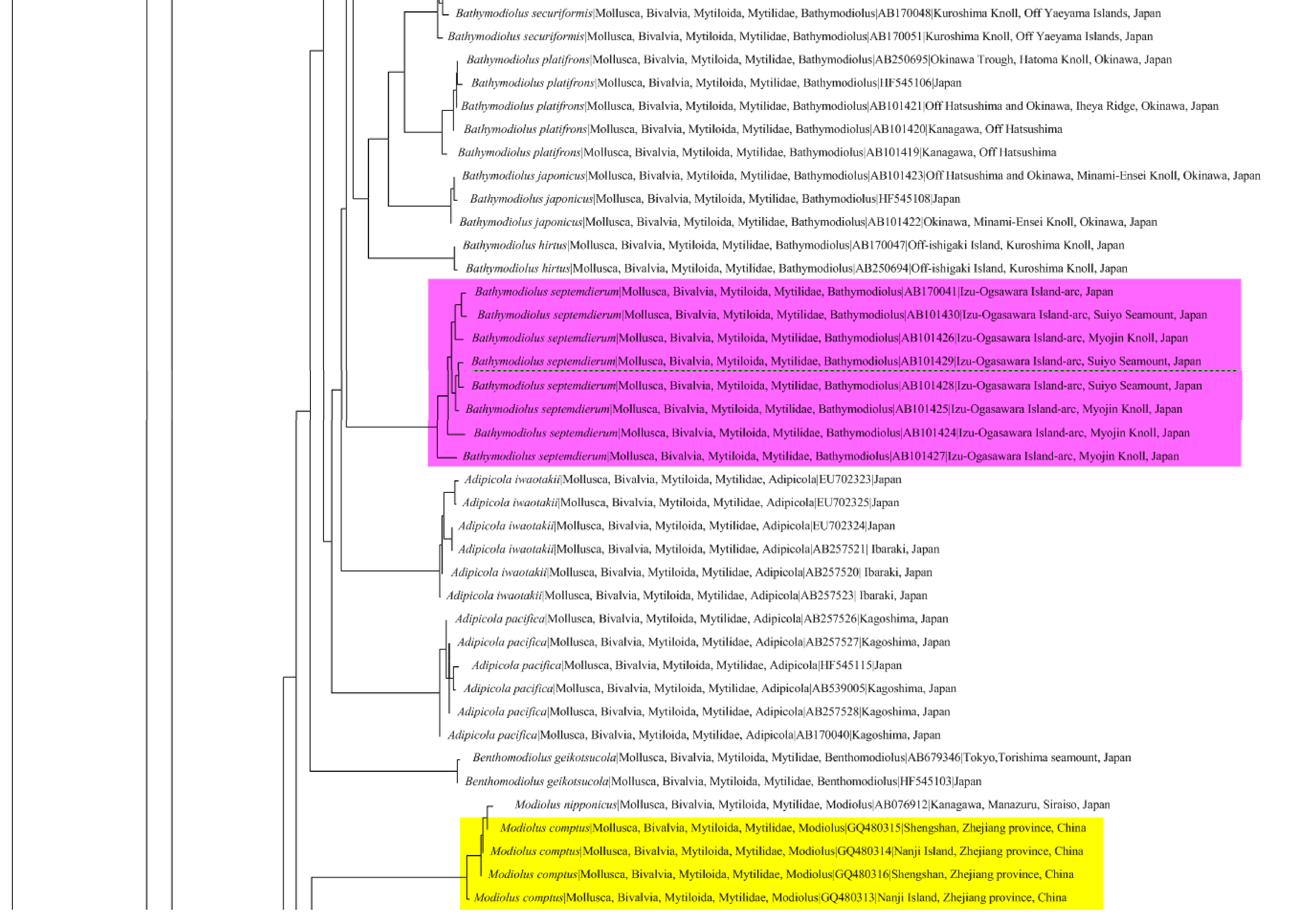

Bathymodiolus securiformis|Mollusca, Bivalvia, Mytiloida, Mytilidae, Bathymodiolus|AB170048|Kuroshima Knoll, Off Yaeyama Islands, Japan
Bathymodiolus securiformis|Mollusca, Bivalvia, Mytiloida, Mytilidae, Bathymodiolus|AB170051|Kuroshima Knoll, Off Yaeyama Islands, Japan
Bathymodiolus platifrons|Mollusca, Bivalvia, Mytiloida, Mytilidae, Bathymodiolus|AB250695|Okinawa Trough, Hatoma Knoll, Okinawa, Japan
Bathymodiolus platifrons|Mollusca, Bivalvia, Mytiloida, Mytilidae, Bathymodiolus|HF545106|Japan
Bathymodiolus platifrons|Mollusca, Bivalvia, Mytiloida, Mytilidae, Bathymodiolus|AB101421|Off Hatsushima and Okinawa, Iheya Ridge, Okinawa, Japan
Bathymodiolus platifrons|Mollusca, Bivalvia, Mytiloida, Mytilidae, Bathymodiolus|AB101420|Kanagawa, Off Hatsushima
Bathymodiolus platifrons|Mollusca, Bivalvia, Mytiloida, Mytilidae, Bathymodiolus|AB101419|Kanagawa, Off Hatsushima
Bathymodiolus japonicus|Mollusca, Bivalvia, Mytiloida, Mytilidae, Bathymodiolus|AB101423|Off Hatsushima and Okinawa, Minami-Ensei Knoll, Okinawa, Japan
Bathymodiolus japonicus|Mollusca, Bivalvia, Mytiloida, Mytilidae, Bathymodiolus|HF545108|Japan
Bathymodiolus japonicus|Mollusca, Bivalvia, Mytiloida, Mytilidae, Bathymodiolus|AB101422|Okinawa, Minami-Ensei Knoll, Okinawa, Japan
Bathymodiolus hirtus|Mollusca, Bivalvia, Mytiloida, Mytilidae, Bathymodiolus|AB170047|Off-ishigaki Island, Kuroshima Knoll, Japan
Bathymodiolus hirtus|Mollusca, Bivalvia, Mytiloida, Mytilidae, Bathymodiolus|AB250694|Off-ishigaki Island, Kuroshima Knoll, Japan
Bathymodiolus septemdierum|Mollusca, Bivalvia, Mytiloida, Mytilidae, Bathymodiolus|AB170041|Izu-Ogsawara Island-arc, Japan
Bathymodiolus septemdierum|Mollusca, Bivalvia, Mytiloida, Mytilidae, Bathymodiolus|AB101430|Izu-Ogasawara Island-arc, Suiyo Seamount, Japan
Bathymodiolus septemdierum|Mollusca, Bivalvia, Mytiloida, Mytilidae, Bathymodiolus|AB101426|Izu-Ogasawara Island-arc, Myojin Knoll, Japan
Bathymodiolus septemdierum|Mollusca, Bivalvia, Mytiloida, Mytilidae, Bathymodiolus|AB101429|Izu-Ogasawara Island-arc, Suiyo Seamount, Japan
Bathymodiolus septemdierum|Mollusca, Bivalvia, Mytiloida, Mytilidae, Bathymodiolus|AB101428|Izu-Ogasawara Island-arc, Suiyo Seamount, Japan
Bathymodiolus septemdierum|Mollusca, Bivalvia, Mytiloida, Mytilidae, Bathymodiolus|AB101425|Izu-Ogasawara Island-arc, Myojin Knoll, Japan
Bathymodiolus septemdierum|Mollusca, Bivalvia, Mytiloida, Mytilidae, Bathymodiolus|AB101424|Izu-Ogasawara Island-arc, Myojin Knoll, Japan
Bathymodiolus septemdierum|Mollusca, Bivalvia, Mytiloida, Mytilidae, Bathymodiolus|AB101427|Izu-Ogasawara Island-arc, Myojin Knoll, Japan
Adipicola iwaotakii|Mollusca, Bivalvia, Mytiloida, Mytilidae, Adipicola|EU702323|Japan
Adipicola iwaotakii|Mollusca, Bivalvia, Mytiloida, Mytilidae, Adipicola|EU702325|Japan
Adipicola iwaotakii|Mollusca, Bivalvia, Mytiloida, Mytilidae, Adipicola|EU702324|Japan
Adipicola iwaotakii|Mollusca, Bivalvia, Mytiloida, Mytilidae, Adipicola|AB257521| Ibaraki, Japan
Adipicola iwaotakii|Mollusca, Bivalvia, Mytiloida, Mytilidae, Adipicola|AB257520| Ibaraki, Japan
Adipicola iwaotakii|Mollusca, Bivalvia, Mytiloida, Mytilidae, Adipicola|AB257523| Ibaraki, Japan
Adipicola pacifica|Mollusca, Bivalvia, Mytiloida, Mytilidae, Adipicola|AB257526|Kagoshima, Japan
Adipicola pacifica|Mollusca, Bivalvia, Mytiloida, Mytilidae, Adipicola|AB257527|Kagoshima, Japan
Adipicola pacifica|Mollusca, Bivalvia, Mytiloida, Mytilidae, Adipicola|HF545115|Japan
Adipicola pacifica|Mollusca, Bivalvia, Mytiloida, Mytilidae, Adipicola|AB539005|Kagoshima, Japan
Adipicola pacifica|Mollusca, Bivalvia, Mytiloida, Mytilidae, Adipicola|AB257528|Kagoshima, Japan
Adipicola pacifica|Mollusca, Bivalvia, Mytiloida, Mytilidae, Adipicola|AB170040|Kagoshima, Japan
Benthomodiolus geikotsucola|Mollusca, Bivalvia, Mytiloida, Mytilidae, Benthomodiolus|AB679346|Tokyo,Torishima seamount, Japan
Benthomodiolus geikotsucola|Mollusca, Bivalvia, Mytiloida, Mytilidae, Benthomodiolus|HF545103|Japan
Modiolus nipponicus|Mollusca, Bivalvia, Mytiloida, Mytilidae, Modiolus|AB076912|Kanagawa, Manazuru, Siraiso, Japan
Modiolus comptus|Mollusca, Bivalvia, Mytiloida, Mytilidae, Modiolus|GQ480315|Shengshan, Zhejiang province, China
Modiolus comptus|Mollusca, Bivalvia, Mytiloida, Mytilidae, Modiolus|GQ480314|Nanji Island, Zhejiang province, China
Modiolus comptus|Mollusca, Bivalvia, Mytiloida, Mytilidae, Modiolus|GQ480316|Shengshan, Zhejiang province, China
Modiolus comptus|Mollusca, Bivalvia, Mytiloida, Mytilidae, Modiolus|GQ480313|Nanji Island, Zhejiang province, China

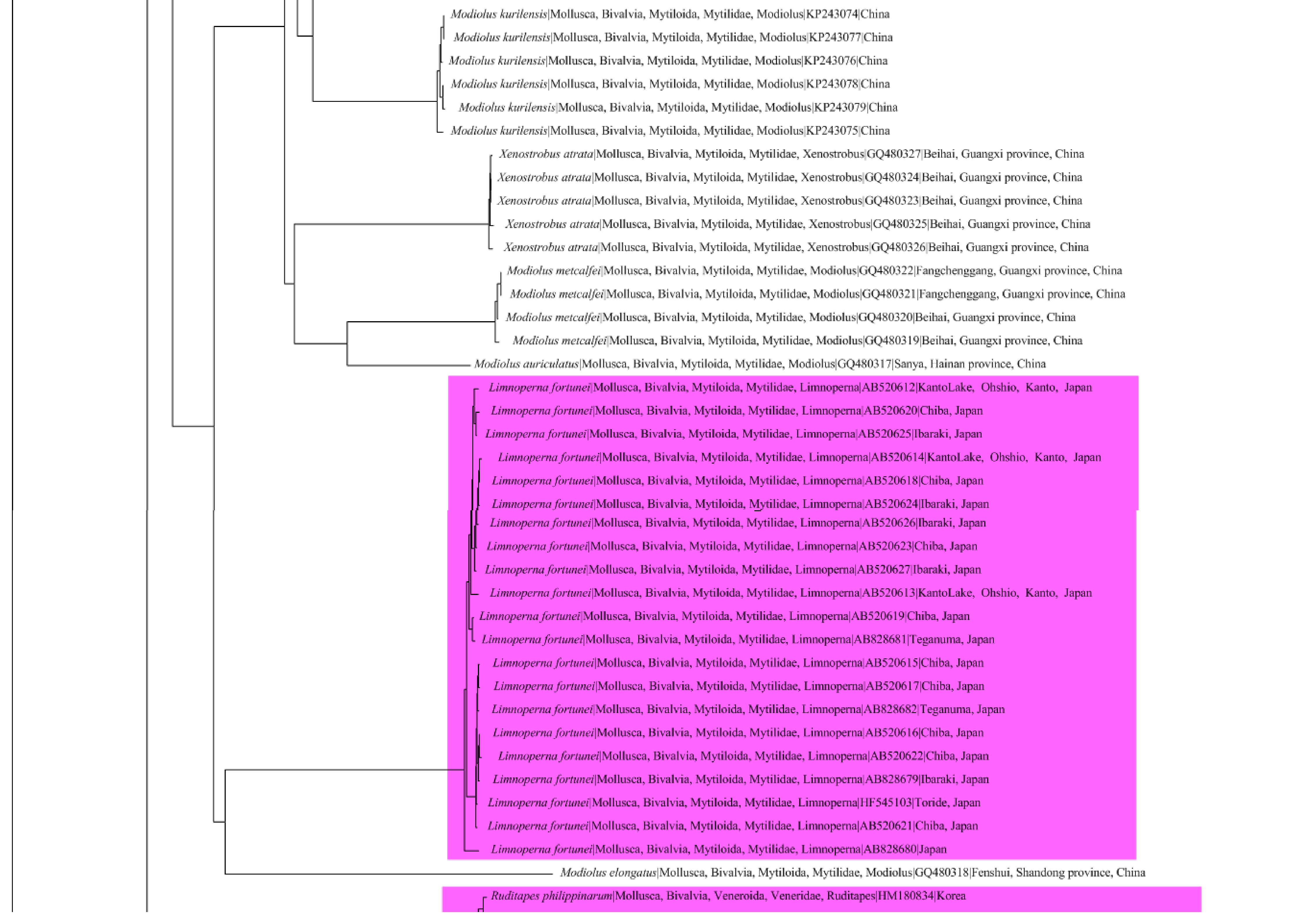
Modiolus kurilensis|Mollusca, Bivalvia, Mytiloida, Mytilidae, Modiolus|KP243074|China
Modiolus kurilensis|Mollusca, Bivalvia, Mytiloida, Mytilidae, Modiolus|KP243077|China
Modiolus kurilensis|Mollusca, Bivalvia, Mytiloida, Mytilidae, Modiolus|KP243076|China
Modiolus kurilensis|Mollusca, Bivalvia, Mytiloida, Mytilidae, Modiolus|KP243078|China
Modiolus kurilensis|Mollusca, Bivalvia, Mytiloida, Mytilidae, Modiolus|KP243079|China
Modiolus kurilensis|Mollusca, Bivalvia, Mytiloida, Mytilidae, Modiolus|KP243075|China
Xenostrobus atrata|Mollusca, Bivalvia, Mytiloida, Mytilidae, Xenostrobus|GQ480327|Beihai, Guangxi province, China
Xenostrobus atrata|Mollusca, Bivalvia, Mytiloida, Mytilidae, Xenostrobus|GQ480324|Beihai, Guangxi province, China
Xenostrobus atrata|Mollusca, Bivalvia, Mytiloida, Mytilidae, Xenostrobus|GQ480323|Beihai, Guangxi province, China
Xenostrobus atrata|Mollusca, Bivalvia, Mytiloida, Mytilidae, Xenostrobus|GQ480325|Beihai, Guangxi province, China
Xenostrobus atrata|Mollusca, Bivalvia, Mytiloida, Mytilidae, Xenostrobus|GQ480326|Beihai, Guangxi province, China
Modiolus metcalfei|Mollusca, Bivalvia, Mytiloida, Mytilidae, Modiolus|GQ480322|Fangchenggang, Guangxi province, China
Modiolus metcalfei|Mollusca, Bivalvia, Mytiloida, Mytilidae, Modiolus|GQ480321|Fangchenggang, Guangxi province, China
Modiolus metcalfei|Mollusca, Bivalvia, Mytiloida, Mytilidae, Modiolus|GQ480320|Beihai, Guangxi province, China
Modiolus metcalfei|Mollusca, Bivalvia, Mytiloida, Mytilidae, Modiolus|GQ480319|Beihai, Guangxi province, China
Modiolus auriculatus|Mollusca, Bivalvia, Mytiloida, Mytilidae, Modiolus|GQ480317|Sanya, Hainan province, China
Limnoperna fortunei|Mollusca, Bivalvia, Mytiloida, Mytilidae, Limnoperna|AB520612|KantoLake, Ohshio, Kanto, Japan
Limnoperna fortunei|Mollusca, Bivalvia, Mytiloida, Mytilidae, Limnoperna|AB520620|Chiba, Japan
Limnoperna fortunei|Mollusca, Bivalvia, Mytiloida, Mytilidae, Limnoperna|AB520625|Ibaraki, Japan
Limnoperna fortunei|Mollusca, Bivalvia, Mytiloida, Mytilidae, Limnoperna|AB520614|KantoLake, Ohshio, Kanto, Japan
Limnoperna fortunei|Mollusca, Bivalvia, Mytiloida, Mytilidae, Limnoperna|AB520618|Chiba, Japan
Limnoperna fortunei|Mollusca, Bivalvia, Mytiloida, Mytilidae, Limnoperna|AB520624|Ibaraki, Japan
Limnoperna fortunei|Mollusca, Bivalvia, Mytiloida, Mytilidae, Limnoperna|AB520626|Ibaraki, Japan
Limnoperna fortunei|Mollusca, Bivalvia, Mytiloida, Mytilidae, Limnoperna|AB520623|Chiba, Japan
Limnoperna fortunei|Mollusca, Bivalvia, Mytiloida, Mytilidae, Limnoperna|AB520627|Ibaraki, Japan
Limnoperna fortunei|Mollusca, Bivalvia, Mytiloida, Mytilidae, Limnoperna|AB520613|KantoLake, Ohshio, Kanto, Japan
Limnoperna fortunei|Mollusca, Bivalvia, Mytiloida, Mytilidae, Limnoperna|AB520619|Chiba, Japan
Limnoperna fortunei|Mollusca, Bivalvia, Mytiloida, Mytilidae, Limnoperna|AB828681|Teganuma, Japan
Limnoperna fortunei|Mollusca, Bivalvia, Mytiloida, Mytilidae, Limnoperna|AB520615|Chiba, Japan
Limnoperna fortunei|Mollusca, Bivalvia, Mytiloida, Mytilidae, Limnoperna|AB520617|Chiba, Japan
Limnoperna fortunei|Mollusca, Bivalvia, Mytiloida, Mytilidae, Limnoperna|AB828682|Teganuma, Japan
Limnoperna fortunei|Mollusca, Bivalvia, Mytiloida, Mytilidae, Limnoperna|AB520616|Chiba, Japan
Limnoperna fortunei|Mollusca, Bivalvia, Mytiloida, Mytilidae, Limnoperna|AB520622|Chiba, Japan
Limnoperna fortunei|Mollusca, Bivalvia, Mytiloida, Mytilidae, Limnoperna|AB828679|Ibaraki, Japan
Limnoperna fortunei|Mollusca, Bivalvia, Mytiloida, Mytilidae, Limnoperna|HF545103|Toride, Japan
Limnoperna fortunei|Mollusca, Bivalvia, Mytiloida, Mytilidae, Limnoperna|AB520621|Chiba, Japan
Limnoperna fortunei|Mollusca, Bivalvia, Mytiloida, Mytilidae, Limnoperna|AB828680|Japan
Modiolus elongatus|Mollusca, Bivalvia, Mytiloida, Mytilidae, Modiolus|GQ480318|Fenshui, Shandong province, China
Ruditapes philippinarum|Mollusca, Bivalvia, Veneroida, Veneridae, Ruditapes|HM180834|Korea

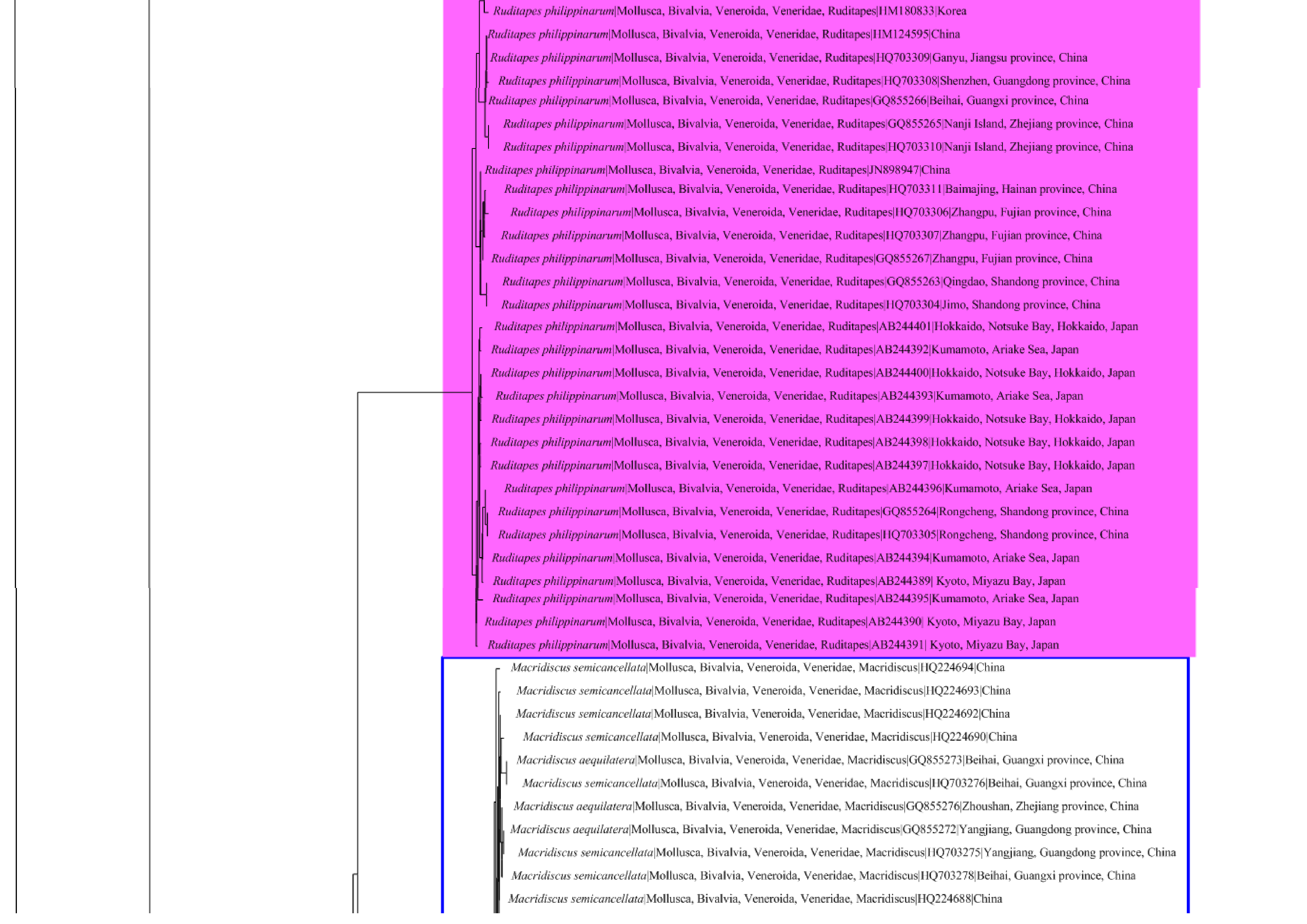

Ruditapes philippinarum|Mollusca, Bivalvia, Veneroida, Veneridae, Ruditapes|HM180833|Korea
Ruditapes philippinarum|Mollusca, Bivalvia, Veneroida, Veneridae, Ruditapes|HM124595|China
Ruditapes philippinarum|Mollusca, Bivalvia, Veneroida, Veneridae, Ruditapes|HQ703309|Ganyu, Jiangsu province, China
Ruditapes philippinarum|Mollusca, Bivalvia, Veneroida, Veneridae, Ruditapes|HQ703308|Shenzhen, Guangdong province, China
Ruditapes philippinarum|Mollusca, Bivalvia, Veneroida, Veneridae, Ruditapes|GQ855266|Beihai, Guangxi province, China
Ruditapes philippinarum|Mollusca, Bivalvia, Veneroida, Veneridae, Ruditapes|GQ855265|Nanji Island, Zhejiang province, China
Ruditapes philippinarum|Mollusca, Bivalvia, Veneroida, Veneridae, Ruditapes|HQ703310|Nanji Island, Zhejiang province, China
Ruditapes philippinarum|Mollusca, Bivalvia, Veneroida, Veneridae, Ruditapes|JN898947|China
Ruditapes philippinarum|Mollusca, Bivalvia, Veneroida, Veneridae, Ruditapes|HQ703311|Baimajing, Hainan province, China
Ruditapes philippinarum|Mollusca, Bivalvia, Veneroida, Veneridae, Ruditapes|HQ703306|Zhangpu, Fujian province, China
Ruditapes philippinarum|Mollusca, Bivalvia, Veneroida, Veneridae, Ruditapes|HQ703307|Zhangpu, Fujian province, China
Ruditapes philippinarum|Mollusca, Bivalvia, Veneroida, Veneridae, Ruditapes|GQ855267|Zhangpu, Fujian province, China
Ruditapes philippinarum|Mollusca, Bivalvia, Veneroida, Veneridae, Ruditapes|GQ855263|Qingdao, Shandong province, China
Ruditapes philippinarum|Mollusca, Bivalvia, Veneroida, Veneridae, Ruditapes|HQ703304|Jimo, Shandong province, China
Ruditapes philippinarum|Mollusca, Bivalvia, Veneroida, Veneridae, Ruditapes|AB244401|Hokkaido, Notsuke Bay, Hokkaido, Japan
Ruditapes philippinarum|Mollusca, Bivalvia, Veneroida, Veneridae, Ruditapes|AB244392|Kumamoto, Ariake Sea, Japan
Ruditapes philippinarum|Mollusca, Bivalvia, Veneroida, Veneridae, Ruditapes|AB244400|Hokkaido, Notsuke Bay, Hokkaido, Japan
Ruditapes philippinarum|Mollusca, Bivalvia, Veneroida, Veneridae, Ruditapes|AB244393|Kumamoto, Ariake Sea, Japan
Ruditapes philippinarum|Mollusca, Bivalvia, Veneroida, Veneridae, Ruditapes|AB244399|Hokkaido, Notsuke Bay, Hokkaido, Japan
Ruditapes philippinarum|Mollusca, Bivalvia, Veneroida, Veneridae, Ruditapes|AB244398|Hokkaido, Notsuke Bay, Hokkaido, Japan
Ruditapes philippinarum|Mollusca, Bivalvia, Veneroida, Veneridae, Ruditapes|AB244397|Hokkaido, Notsuke Bay, Hokkaido, Japan
Ruditapes philippinarum|Mollusca, Bivalvia, Veneroida, Veneridae, Ruditapes|AB244396|Kumamoto, Ariake Sea, Japan
Ruditapes philippinarum|Mollusca, Bivalvia, Veneroida, Veneridae, Ruditapes|GQ855264|Rongcheng, Shandong province, China
Ruditapes philippinarum|Mollusca, Bivalvia, Veneroida, Veneridae, Ruditapes|HQ703305|Rongcheng, Shandong province, China
Ruditapes philippinarum|Mollusca, Bivalvia, Veneroida, Veneridae, Ruditapes|AB244394|Kumamoto, Ariake Sea, Japan
Ruditapes philippinarum|Mollusca, Bivalvia, Veneroida, Veneridae, Ruditapes|AB244389| Kyoto, Miyazu Bay, Japan
Ruditapes philippinarum|Mollusca, Bivalvia, Veneroida, Veneridae, Ruditapes|AB244395|Kumamoto, Ariake Sea, Japan
Ruditapes philippinarum|Mollusca, Bivalvia, Veneroida, Veneridae, Ruditapes|AB244390| Kyoto, Miyazu Bay, Japan
Ruditapes philippinarum|Mollusca, Bivalvia, Veneroida, Veneridae, Ruditapes|AB244391| Kyoto, Miyazu Bay, Japan
Macridiscus semicancellata|Mollusca, Bivalvia, Veneroida, Veneridae, Macridiscus|HQ224694|China
Macridiscus semicancellata|Mollusca, Bivalvia, Veneroida, Veneridae, Macridiscus|HQ224693|China
Macridiscus semicancellata|Mollusca, Bivalvia, Veneroida, Veneridae, Macridiscus|HQ224692|China
Macridiscus semicancellata|Mollusca, Bivalvia, Veneroida, Veneridae, Macridiscus|HQ224690|China
Macridiscus aequilatera|Mollusca, Bivalvia, Veneroida, Veneridae, Macridiscus|GQ855273|Beihai, Guangxi province, China
Macridiscus semicancellata|Mollusca, Bivalvia, Veneroida, Veneridae, Macridiscus|HQ703276|Beihai, Guangxi province, China
Macridiscus aequilatera|Mollusca, Bivalvia, Veneroida, Veneridae, Macridiscus|GQ855276|Zhoushan, Zhejiang province, China
Macridiscus aequilatera|Mollusca, Bivalvia, Veneroida, Veneridae, Macridiscus|GQ855272|Yangjiang, Guangdong province, China
Macridiscus semicancellata|Mollusca, Bivalvia, Veneroida, Veneridae, Macridiscus|HQ703275|Yangjiang, Guangdong province, China
Macridiscus semicancellata|Mollusca, Bivalvia, Veneroida, Veneridae, Macridiscus|HQ703278|Beihai, Guangxi province, China
Macridiscus semicancellata|Mollusca, Bivalvia, Veneroida, Veneridae, Macridiscus|HQ224688|China

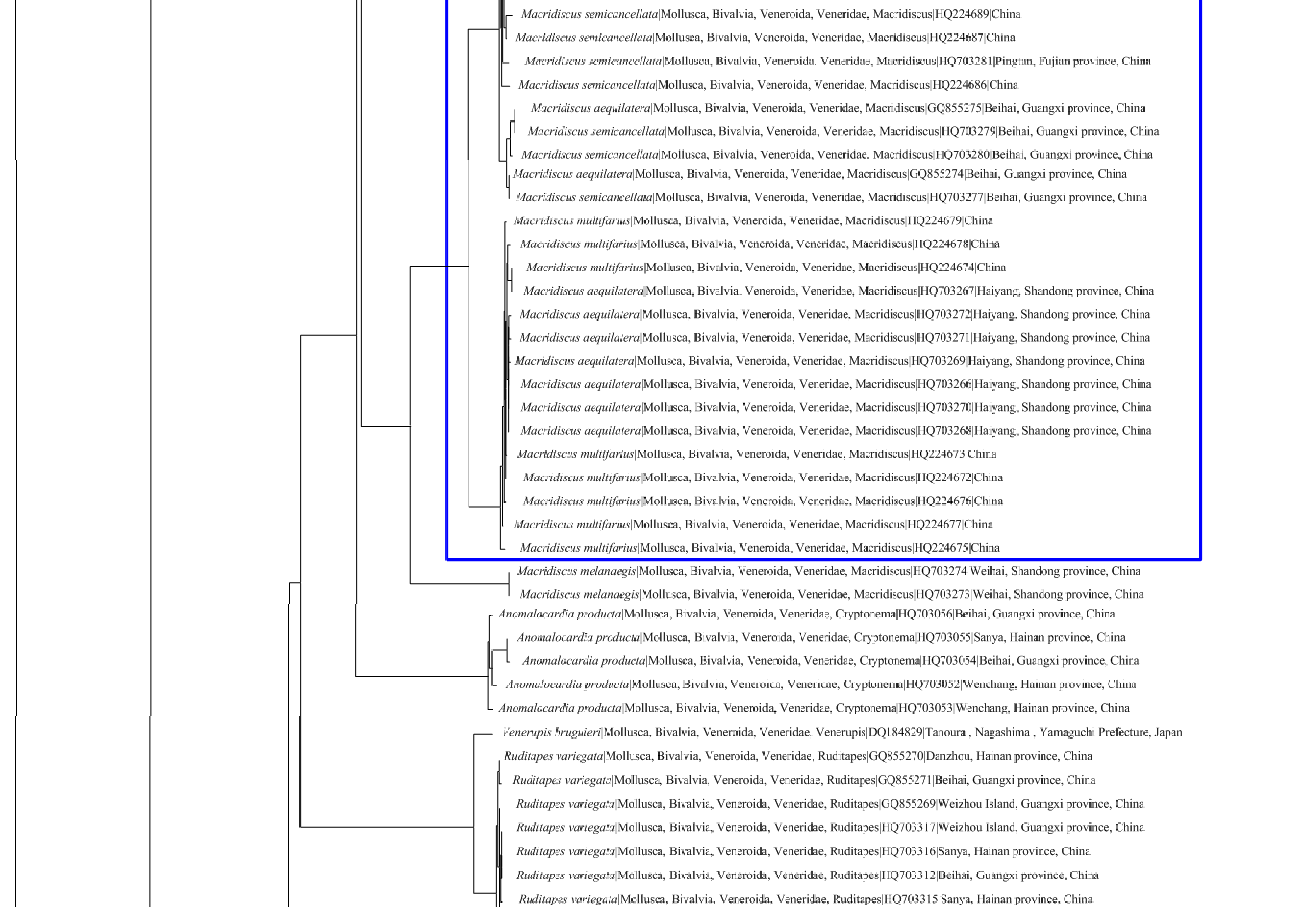

Macridiscus semicancellata|Mollusca, Bivalvia, Veneroida, Veneridae, Macridiscus|HQ224689|China
Macridiscus semicancellata|Mollusca, Bivalvia, Veneroida, Veneridae, Macridiscus|HQ224687|China
Macridiscus semicancellata|Mollusca, Bivalvia, Veneroida, Veneridae, Macridiscus|HQ703281|Pingtan, Fujian province, China
Macridiscus semicancellata|Mollusca, Bivalvia, Veneroida, Veneridae, Macridiscus|HQ224686|China
Macridiscus aequilatera|Mollusca, Bivalvia, Veneroida, Veneridae, Macridiscus|GQ855275|Beihai, Guangxi province, China
Macridiscus semicancellata|Mollusca, Bivalvia, Veneroida, Veneridae, Macridiscus|HQ703279|Beihai, Guangxi province, China
Macridiscus semicancellata|Mollusca, Bivalvia, Veneroida, Veneridae, Macridiscus|HQ703280|Beihai, Guangxi province, China
Macridiscus aequilatera|Mollusca, Bivalvia, Veneroida, Veneridae, Macridiscus|GQ855274|Beihai, Guangxi province, China
Macridiscus semicancellata|Mollusca, Bivalvia, Veneroida, Veneridae, Macridiscus|HQ703277|Beihai, Guangxi province, China
Macridiscus multifarius|Mollusca, Bivalvia, Veneroida, Veneridae, Macridiscus|HQ224679|China
Macridiscus multifarius|Mollusca, Bivalvia, Veneroida, Veneridae, Macridiscus|HQ224678|China
Macridiscus multifarius|Mollusca, Bivalvia, Veneroida, Veneridae, Macridiscus|HQ224674|China
Macridiscus aequilatera|Mollusca, Bivalvia, Veneroida, Veneridae, Macridiscus|HQ703267|Haiyang, Shandong province, China
Macridiscus aequilatera|Mollusca, Bivalvia, Veneroida, Veneridae, Macridiscus|HQ703272|Haiyang, Shandong province, China
Macridiscus aequilatera|Mollusca, Bivalvia, Veneroida, Veneridae, Macridiscus|HQ703271|Haiyang, Shandong province, China
Macridiscus aequilatera|Mollusca, Bivalvia, Veneroida, Veneridae, Macridiscus|HQ703269|Haiyang, Shandong province, China
Macridiscus aequilatera|Mollusca, Bivalvia, Veneroida, Veneridae, Macridiscus|HQ703266|Haiyang, Shandong province, China
Macridiscus aequilatera|Mollusca, Bivalvia, Veneroida, Veneridae, Macridiscus|HQ703270|Haiyang, Shandong province, China
Macridiscus aequilatera|Mollusca, Bivalvia, Veneroida, Veneridae, Macridiscus|HQ703268|Haiyang, Shandong province, China
Macridiscus multifarius|Mollusca, Bivalvia, Veneroida, Veneridae, Macridiscus|HQ224673|China
Macridiscus multifarius|Mollusca, Bivalvia, Veneroida, Veneridae, Macridiscus|HQ224672|China
Macridiscus multifarius|Mollusca, Bivalvia, Veneroida, Veneridae, Macridiscus|HQ224676|China
Macridiscus multifarius|Mollusca, Bivalvia, Veneroida, Veneridae, Macridiscus|HQ224677|China
Macridiscus multifarius|Mollusca, Bivalvia, Veneroida, Veneridae, Macridiscus|HQ224675|China
Macridiscus melanaegis|Mollusca, Bivalvia, Veneroida, Veneridae, Macridiscus|HQ703274|Weihai, Shandong province, China
Macridiscus melanaegis|Mollusca, Bivalvia, Veneroida, Veneridae, Macridiscus|HQ703273|Weihai, Shandong province, China
Anomalocardia producta|Mollusca, Bivalvia, Veneroida, Veneridae, Cryptonema|HQ703056|Beihai, Guangxi province, China
Anomalocardia producta|Mollusca, Bivalvia, Veneroida, Veneridae, Cryptonema|HQ703055|Sanya, Hainan province, China
Anomalocardia producta|Mollusca, Bivalvia, Veneroida, Veneridae, Cryptonema|HQ703054|Beihai, Guangxi province, China
Anomalocardia producta|Mollusca, Bivalvia, Veneroida, Veneridae, Cryptonema|HQ703052|Wenchang, Hainan province, China
Anomalocardia producta|Mollusca, Bivalvia, Veneroida, Veneridae, Cryptonema|HQ703053|Wenchang, Hainan province, China
Venerupis bruguieri|Mollusca, Bivalvia, Veneroida, Veneridae, Venerupis|DQ184829|Tanoura , Nagashima , Yamaguchi Prefecture, Japan
Ruditapes variegata|Mollusca, Bivalvia, Veneroida, Veneridae, Ruditapes|GQ855270|Danzhou, Hainan province, China
Ruditapes variegata|Mollusca, Bivalvia, Veneroida, Veneridae, Ruditapes|GQ855271|Beihai, Guangxi province, China
Ruditapes variegata|Mollusca, Bivalvia, Veneroida, Veneridae, Ruditapes|GQ855269|Weizhou Island, Guangxi province, China
Ruditapes variegata|Mollusca, Bivalvia, Veneroida, Veneridae, Ruditapes|HQ703317|Weizhou Island, Guangxi province, China
Ruditapes variegata|Mollusca, Bivalvia, Veneroida, Veneridae, Ruditapes|HQ703316|Sanya, Hainan province, China
Ruditapes variegata|Mollusca, Bivalvia, Veneroida, Veneridae, Ruditapes|HQ703312|Beihai, Guangxi province, China
Ruditapes variegata|Mollusca, Bivalvia, Veneroida, Veneridae, Ruditapes|HQ703315|Sanya, Hainan province, China

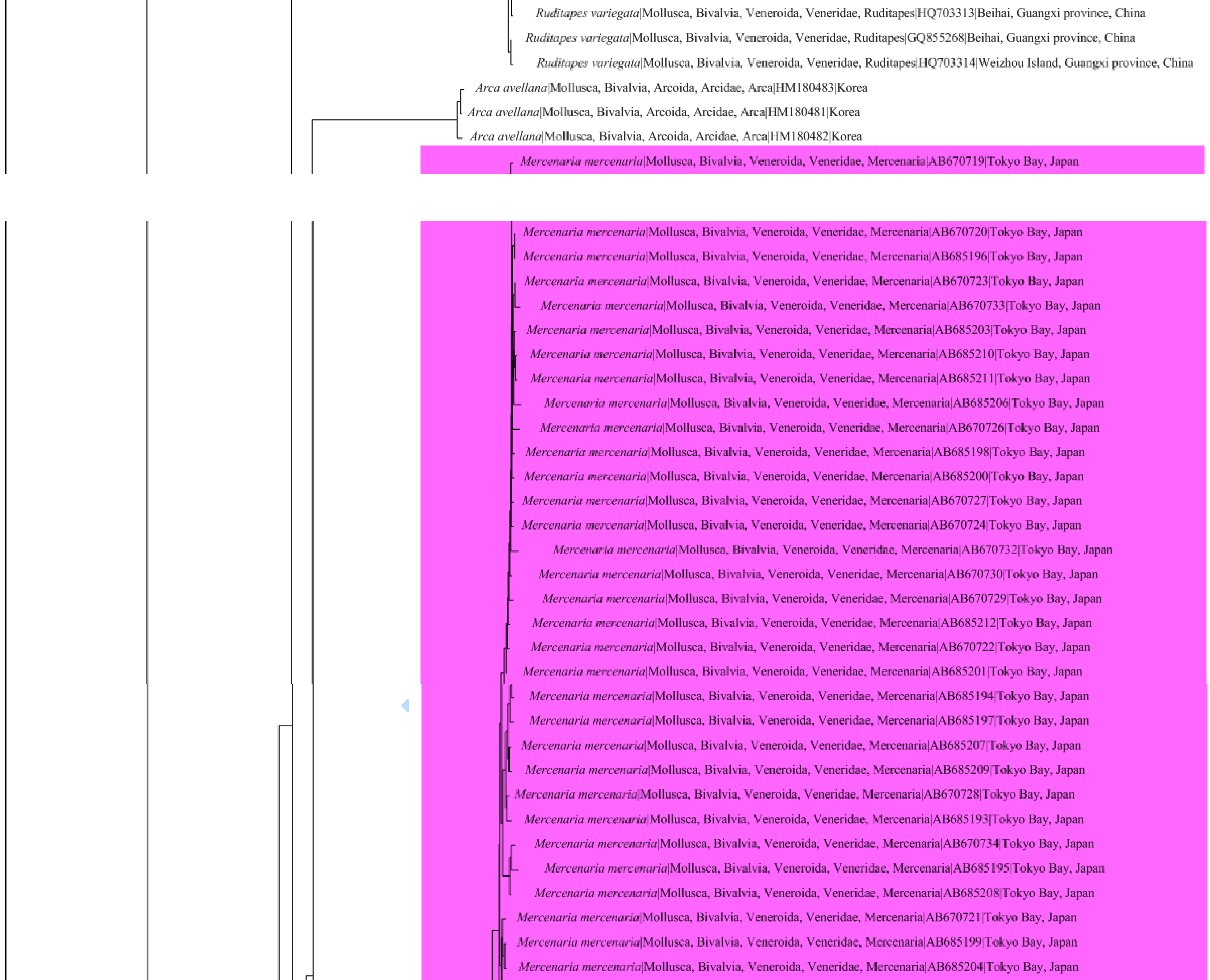

Ruditapes variegata|Mollusca, Bivalvia, Veneroida, Veneridae, Ruditapes|HQ703313|Beihai, Guangxi province, China
Ruditapes variegata|Mollusca, Bivalvia, Veneroida, Veneridae, Ruditapes|GQ855268|Beihai, Guangxi province, China
Ruditapes variegata|Mollusca, Bivalvia, Veneroida, Veneridae, Ruditapes|HQ703314|Weizhou Island, Guangxi province, China
Arca avellana|Mollusca, Bivalvia, Arcoida, Arcidae, Arca|HM180483|Korea
Arca avellana|Mollusca, Bivalvia, Arcoida, Arcidae, Arca|HM180481|Korea
Arca avellana|Mollusca, Bivalvia, Arcoida, Arcidae, Arca|HM180482|Korea
Mercenaria mercenaria|Mollusca, Bivalvia, Veneroida, Veneridae, Mercenaria|AB670719|Tokyo Bay, Japan
Mercenaria mercenaria|Mollusca, Bivalvia, Veneroida, Veneridae, Mercenaria|AB670720|Tokyo Bay, Japan
Mercenaria mercenaria|Mollusca, Bivalvia, Veneroida, Veneridae, Mercenaria|AB685196|Tokyo Bay, Japan
Mercenaria mercenaria|Mollusca, Bivalvia, Veneroida, Veneridae, Mercenaria|AB670723|Tokyo Bay, Japan
Mercenaria mercenaria|Mollusca, Bivalvia, Veneroida, Veneridae, Mercenaria|AB670733|Tokyo Bay, Japan
Mercenaria mercenaria|Mollusca, Bivalvia, Veneroida, Veneridae, Mercenaria|AB685203|Tokyo Bay, Japan
Mercenaria mercenaria|Mollusca, Bivalvia, Veneroida, Veneridae, Mercenaria|AB685210|Tokyo Bay, Japan
Mercenaria mercenaria|Mollusca, Bivalvia, Veneroida, Veneridae, Mercenaria|AB685211|Tokyo Bay, Japan
Mercenaria mercenaria|Mollusca, Bivalvia, Veneroida, Veneridae, Mercenaria|AB685206|Tokyo Bay, Japan
Mercenaria mercenaria|Mollusca, Bivalvia, Veneroida, Veneridae, Mercenaria|AB670726|Tokyo Bay, Japan
Mercenaria mercenaria|Mollusca, Bivalvia, Veneroida, Veneridae, Mercenaria|AB685198|Tokyo Bay, Japan
Mercenaria mercenaria|Mollusca, Bivalvia, Veneroida, Veneridae, Mercenaria|AB685200|Tokyo Bay, Japan
Mercenaria mercenaria|Mollusca, Bivalvia, Veneroida, Veneridae, Mercenaria|AB670727|Tokyo Bay, Japan
Mercenaria mercenaria|Mollusca, Bivalvia, Veneroida, Veneridae, Mercenaria|AB670724|Tokyo Bay, Japan
Mercenaria mercenaria|Mollusca, Bivalvia, Veneroida, Veneridae, Mercenaria|AB670732|Tokyo Bay, Japan
Mercenaria mercenaria|Mollusca, Bivalvia, Veneroida, Veneridae, Mercenaria|AB670730|Tokyo Bay, Japan
Mercenaria mercenaria|Mollusca, Bivalvia, Veneroida, Veneridae, Mercenaria|AB670729|Tokyo Bay, Japan
Mercenaria mercenaria|Mollusca, Bivalvia, Veneroida, Veneridae, Mercenaria|AB685212|Tokyo Bay, Japan
Mercenaria mercenaria|Mollusca, Bivalvia, Veneroida, Veneridae, Mercenaria|AB670722|Tokyo Bay, Japan
Mercenaria mercenaria|Mollusca, Bivalvia, Veneroida, Veneridae, Mercenaria|AB685201|Tokyo Bay, Japan
Mercenaria mercenaria|Mollusca, Bivalvia, Veneroida, Veneridae, Mercenaria|AB685194|Tokyo Bay, Japan
Mercenaria mercenaria|Mollusca, Bivalvia, Veneroida, Veneridae, Mercenaria|AB685197|Tokyo Bay, Japan
Mercenaria mercenaria|Mollusca, Bivalvia, Veneroida, Veneridae, Mercenaria|AB685207|Tokyo Bay, Japan
Mercenaria mercenaria|Mollusca, Bivalvia, Veneroida, Veneridae, Mercenaria|AB685209|Tokyo Bay, Japan
Mercenaria mercenaria|Mollusca, Bivalvia, Veneroida, Veneridae, Mercenaria|AB670728|Tokyo Bay, Japan
Mercenaria mercenaria|Mollusca, Bivalvia, Veneroida, Veneridae, Mercenaria|AB685193|Tokyo Bay, Japan
Mercenaria mercenaria|Mollusca, Bivalvia, Veneroida, Veneridae, Mercenaria|AB670734|Tokyo Bay, Japan
Mercenaria mercenaria|Mollusca, Bivalvia, Veneroida, Veneridae, Mercenaria|AB685195|Tokyo Bay, Japan
Mercenaria mercenaria|Mollusca, Bivalvia, Veneroida, Veneridae, Mercenaria|AB685208|Tokyo Bay, Japan
Mercenaria mercenaria|Mollusca, Bivalvia, Veneroida, Veneridae, Mercenaria|AB670721|Tokyo Bay, Japan
Mercenaria mercenaria|Mollusca, Bivalvia, Veneroida, Veneridae, Mercenaria|AB685199|Tokyo Bay, Japan
Mercenaria mercenaria|Mollusca, Bivalvia, Veneroida, Veneridae, Mercenaria|AB685204|Tokyo Bay, Japan

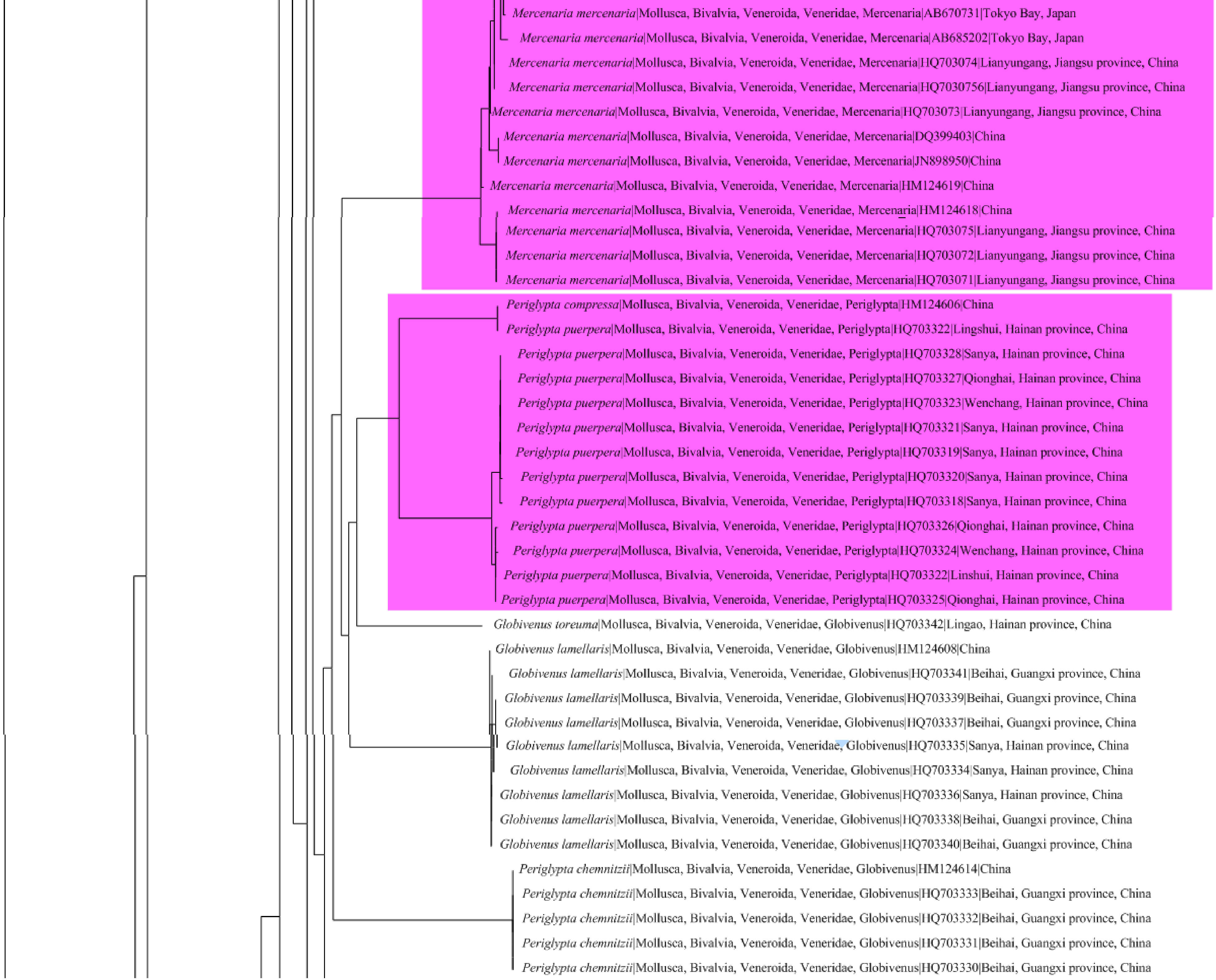

Mercenaria mercenaria|Mollusca, Bivalvia, Veneroida, Veneridae, Mercenaria|AB670731|Tokyo Bay, Japan
Mercenaria mercenaria|Mollusca, Bivalvia, Veneroida, Veneridae, Mercenaria|AB685202|Tokyo Bay, Japan
Mercenaria mercenaria|Mollusca, Bivalvia, Veneroida, Veneridae, Mercenaria|HQ703074|Lianyungang, Jiangsu province, China
Mercenaria mercenaria|Mollusca, Bivalvia, Veneroida, Veneridae, Mercenaria|HQ7030756|Lianyungang, Jiangsu province, China
Mercenaria mercenaria|Mollusca, Bivalvia, Veneroida, Veneridae, Mercenaria|HQ703073|Lianyungang, Jiangsu province, China
Mercenaria mercenaria|Mollusca, Bivalvia, Veneroida, Veneridae, Mercenaria|DQ399403|China
Mercenaria mercenaria|Mollusca, Bivalvia, Veneroida, Veneridae, Mercenaria|JN898950|China
Mercenaria mercenaria|Mollusca, Bivalvia, Veneroida, Veneridae, Mercenaria|HM124619|China
Mercenaria mercenaria|Mollusca, Bivalvia, Veneroida, Veneridae, Mercenaria|HM124618|China
Mercenaria mercenaria|Mollusca, Bivalvia, Veneroida, Veneridae, Mercenaria|HQ703075|Lianyungang, Jiangsu province, China
Mercenaria mercenaria|Mollusca, Bivalvia, Veneroida, Veneridae, Mercenaria|HQ703072|Lianyungang, Jiangsu province, China
Mercenaria mercenaria|Mollusca, Bivalvia, Veneroida, Veneridae, Mercenaria|HQ703071|Lianyungang, Jiangsu province, China
Periglypta compressa|Mollusca, Bivalvia, Veneroida, Veneridae, Periglypta|HM124606|China
Periglypta puerpera|Mollusca, Bivalvia, Veneroida, Veneridae, Periglypta|HQ703322|Lingshui, Hainan province, China
Periglypta puerpera|Mollusca, Bivalvia, Veneroida, Veneridae, Periglypta|HQ703328|Sanya, Hainan province, China
Periglypta puerpera|Mollusca, Bivalvia, Veneroida, Veneridae, Periglypta|HQ703327|Qionghai, Hainan province, China
Periglypta puerpera|Mollusca, Bivalvia, Veneroida, Veneridae, Periglypta|HQ703323|Wenchang, Hainan province, China
Periglypta puerpera|Mollusca, Bivalvia, Veneroida, Veneridae, Periglypta|HQ703321|Sanya, Hainan province, China
Periglypta puerpera|Mollusca, Bivalvia, Veneroida, Veneridae, Periglypta|HQ703319|Sanya, Hainan province, China
Periglypta puerpera|Mollusca, Bivalvia, Veneroida, Veneridae, Periglypta|HQ703320|Sanya, Hainan province, China
Periglypta puerpera|Mollusca, Bivalvia, Veneroida, Veneridae, Periglypta|HQ703318|Sanya, Hainan province, China
Periglypta puerpera|Mollusca, Bivalvia, Veneroida, Veneridae, Periglypta|HQ703326|Qionghai, Hainan province, China
Periglypta puerpera|Mollusca, Bivalvia, Veneroida, Veneridae, Periglypta|HQ703324|Wenchang, Hainan province, China
Periglypta puerpera|Mollusca, Bivalvia, Veneroida, Veneridae, Periglypta|HQ703322|Linshui, Hainan province, China
Periglypta puerpera|Mollusca, Bivalvia, Veneroida, Veneridae, Periglypta|HQ703325|Qionghai, Hainan province, China
Globivenus toreuma|Mollusca, Bivalvia, Veneroida, Veneridae, Globivenus|HQ703342|Lingao, Hainan province, China
Globivenus lamellaris|Mollusca, Bivalvia, Veneroida, Veneridae, Globivenus|HM124608|China
Globivenus lamellaris|Mollusca, Bivalvia, Veneroida, Veneridae, Globivenus|HQ703341|Beihai, Guangxi province, China
Globivenus lamellaris|Mollusca, Bivalvia, Veneroida, Veneridae, Globivenus|HQ703339|Beihai, Guangxi province, China
Globivenus lamellaris|Mollusca, Bivalvia, Veneroida, Veneridae, Globivenus|HQ703337|Beihai, Guangxi province, China
Globivenus lamellaris|Mollusca, Bivalvia, Veneroida, Veneridae, Globivenus|HQ703335|Sanya, Hainan province, China
Globivenus lamellaris|Mollusca, Bivalvia, Veneroida, Veneridae, Globivenus|HQ703334|Sanya, Hainan province, China
Globivenus lamellaris|Mollusca, Bivalvia, Veneroida, Veneridae, Globivenus|HQ703336|Sanya, Hainan province, China
Globivenus lamellaris|Mollusca, Bivalvia, Veneroida, Veneridae, Globivenus|HQ703338|Beihai, Guangxi province, China
Globivenus lamellaris|Mollusca, Bivalvia, Veneroida, Veneridae, Globivenus|HQ703340|Beihai, Guangxi province, China
Periglypta chemnitzii|Mollusca, Bivalvia, Veneroida, Veneridae, Globivenus|HM124614|China
Periglypta chemnitzii|Mollusca, Bivalvia, Veneroida, Veneridae, Globivenus|HQ703333|Beihai, Guangxi province, China
Periglypta chemnitzii|Mollusca, Bivalvia, Veneroida, Veneridae, Globivenus|HQ703332|Beihai, Guangxi province, China
Periglypta chemnitzii|Mollusca, Bivalvia, Veneroida, Veneridae, Globivenus|HQ703331|Beihai, Guangxi province, China
Periglypta chemnitzii|Mollusca, Bivalvia, Veneroida, Veneridae, Globivenus|HQ703330|Beihai, Guangxi province, China

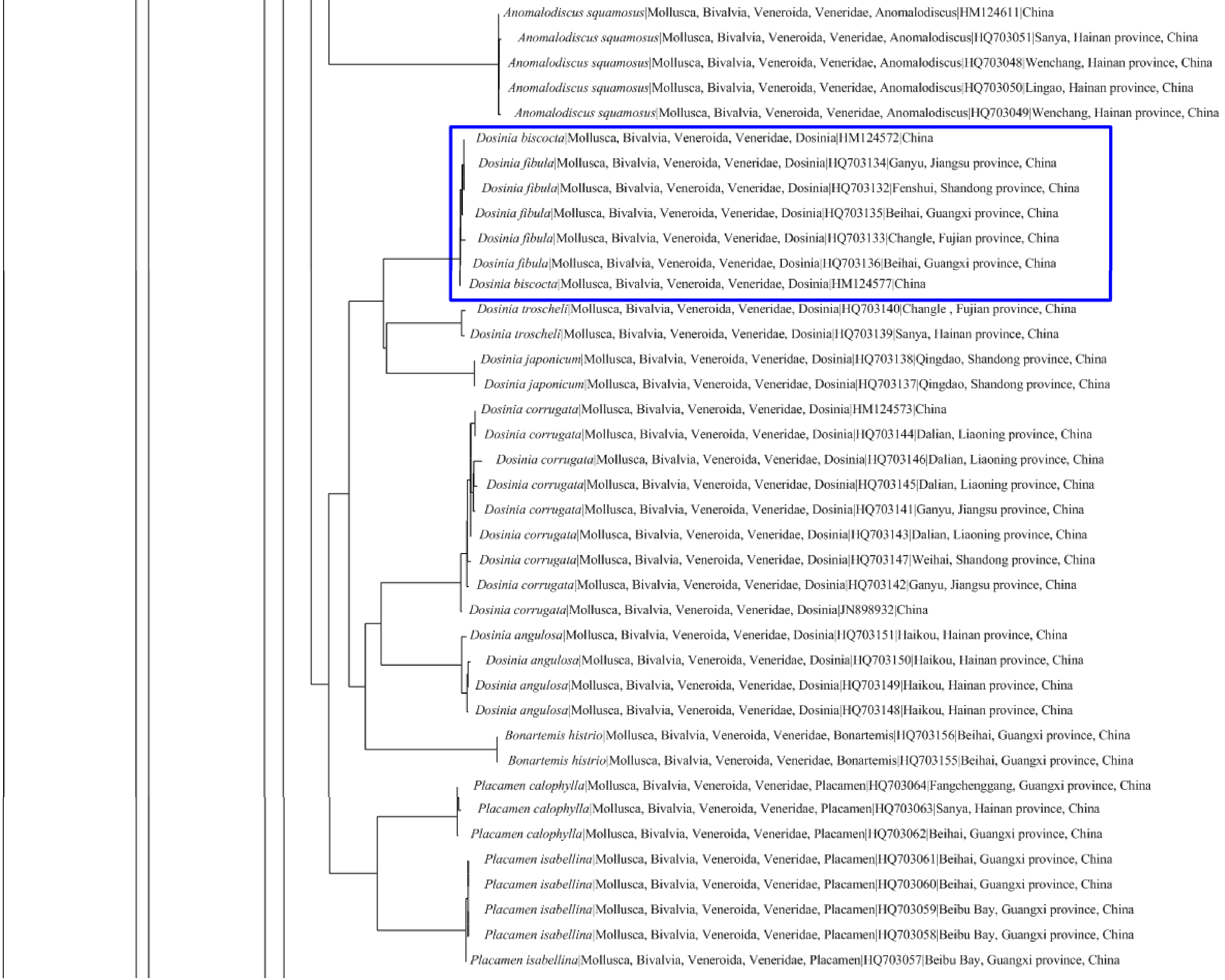

Anomalodiscus squamosus|Mollusca, Bivalvia, Veneroida, Veneridae, Anomalodiscus|HM124611|China
Anomalodiscus squamosus|Mollusca, Bivalvia, Veneroida, Veneridae, Anomalodiscus|HQ703051|Sanya, Hainan province, China
Anomalodiscus squamosus|Mollusca, Bivalvia, Veneroida, Veneridae, Anomalodiscus|HQ703048|Wenchang, Hainan province, China
Anomalodiscus squamosus|Mollusca, Bivalvia, Veneroida, Veneridae, Anomalodiscus|HQ703050|Lingao, Hainan province, China
Anomalodiscus squamosus|Mollusca, Bivalvia, Veneroida, Veneridae, Anomalodiscus|HQ703049|Wenchang, Hainan province, China
Dosinia biscocta|Mollusca, Bivalvia, Veneroida, Veneridae, Dosinia|HM124572|China
Dosinia fibula|Mollusca, Bivalvia, Veneroida, Veneridae, Dosinia|HQ703134|Ganyu, Jiangsu province, China
Dosinia fibula|Mollusca, Bivalvia, Veneroida, Veneridae, Dosinia|HQ703132|Fenshui, Shandong province, China
Dosinia fibula|Mollusca, Bivalvia, Veneroida, Veneridae, Dosinia|HQ703135|Beihai, Guangxi province, China
Dosinia fibula|Mollusca, Bivalvia, Veneroida, Veneridae, Dosinia|HQ703133|Changle, Fujian province, China
Dosinia fibula|Mollusca, Bivalvia, Veneroida, Veneridae, Dosinia|HQ703136|Beihai, Guangxi province, China
Dosinia biscocta|Mollusca, Bivalvia, Veneroida, Veneridae, Dosinia|HM124577|China
Dosinia troscheli|Mollusca, Bivalvia, Veneroida, Veneridae, Dosinia|HQ703140|Changle , Fujian province, China
Dosinia troscheli|Mollusca, Bivalvia, Veneroida, Veneridae, Dosinia|HQ703139|Sanya, Hainan province, China
Dosinia japonicum|Mollusca, Bivalvia, Veneroida, Veneridae, Dosinia|HQ703138|Qingdao, Shandong province, China
Dosinia japonicum|Mollusca, Bivalvia, Veneroida, Veneridae, Dosinia|HQ703137|Qingdao, Shandong province, China
Dosinia corrugata|Mollusca, Bivalvia, Veneroida, Veneridae, Dosinia|HM124573|China
Dosinia corrugata|Mollusca, Bivalvia, Veneroida, Veneridae, Dosinia|HQ703144|Dalian, Liaoning province, China
Dosinia corrugata|Mollusca, Bivalvia, Veneroida, Veneridae, Dosinia|HQ703146|Dalian, Liaoning province, China
Dosinia corrugata|Mollusca, Bivalvia, Veneroida, Veneridae, Dosinia|HQ703145|Dalian, Liaoning province, China
Dosinia corrugata|Mollusca, Bivalvia, Veneroida, Veneridae, Dosinia|HQ703141|Ganyu, Jiangsu province, China
Dosinia corrugata|Mollusca, Bivalvia, Veneroida, Veneridae, Dosinia|HQ703143|Dalian, Liaoning province, China
Dosinia corrugata|Mollusca, Bivalvia, Veneroida, Veneridae, Dosinia|HQ703147|Weihai, Shandong province, China
Dosinia corrugata|Mollusca, Bivalvia, Veneroida, Veneridae, Dosinia|HQ703142|Ganyu, Jiangsu province, China
Dosinia corrugata|Mollusca, Bivalvia, Veneroida, Veneridae, Dosinia|JN898932|China
Dosinia angulosa|Mollusca, Bivalvia, Veneroida, Veneridae, Dosinia|HQ703151|Haikou, Hainan province, China
Dosinia angulosa|Mollusca, Bivalvia, Veneroida, Veneridae, Dosinia|HQ703150|Haikou, Hainan province, China
Dosinia angulosa|Mollusca, Bivalvia, Veneroida, Veneridae, Dosinia|HQ703149|Haikou, Hainan province, China
Dosinia angulosa|Mollusca, Bivalvia, Veneroida, Veneridae, Dosinia|HQ703148|Haikou, Hainan province, China
Bonartemis histrio|Mollusca, Bivalvia, Veneroida, Veneridae, Bonartemis|HQ703156|Beihai, Guangxi province, China
Bonartemis histrio|Mollusca, Bivalvia, Veneroida, Veneridae, Bonartemis|HQ703155|Beihai, Guangxi province, China
Placamen calophylla|Mollusca, Bivalvia, Veneroida, Veneridae, Placamen|HQ703064|Fangchenggang, Guangxi province, China
Placamen calophylla|Mollusca, Bivalvia, Veneroida, Veneridae, Placamen|HQ703063|Sanya, Hainan province, China
Placamen calophylla|Mollusca, Bivalvia, Veneroida, Veneridae, Placamen|HQ703062|Beihai, Guangxi province, China
Placamen isabellina|Mollusca, Bivalvia, Veneroida, Veneridae, Placamen|HQ703061|Beihai, Guangxi province, China
Placamen isabellina|Mollusca, Bivalvia, Veneroida, Veneridae, Placamen|HQ703060|Beihai, Guangxi province, China
Placamen isabellina|Mollusca, Bivalvia, Veneroida, Veneridae, Placamen|HQ703059|Beibu Bay, Guangxi province, China
Placamen isabellina|Mollusca, Bivalvia, Veneroida, Veneridae, Placamen|HQ703058|Beibu Bay, Guangxi province, China
Placamen isabellina|Mollusca, Bivalvia, Veneroida, Veneridae, Placamen|HQ703057|Beibu Bay, Guangxi province, China

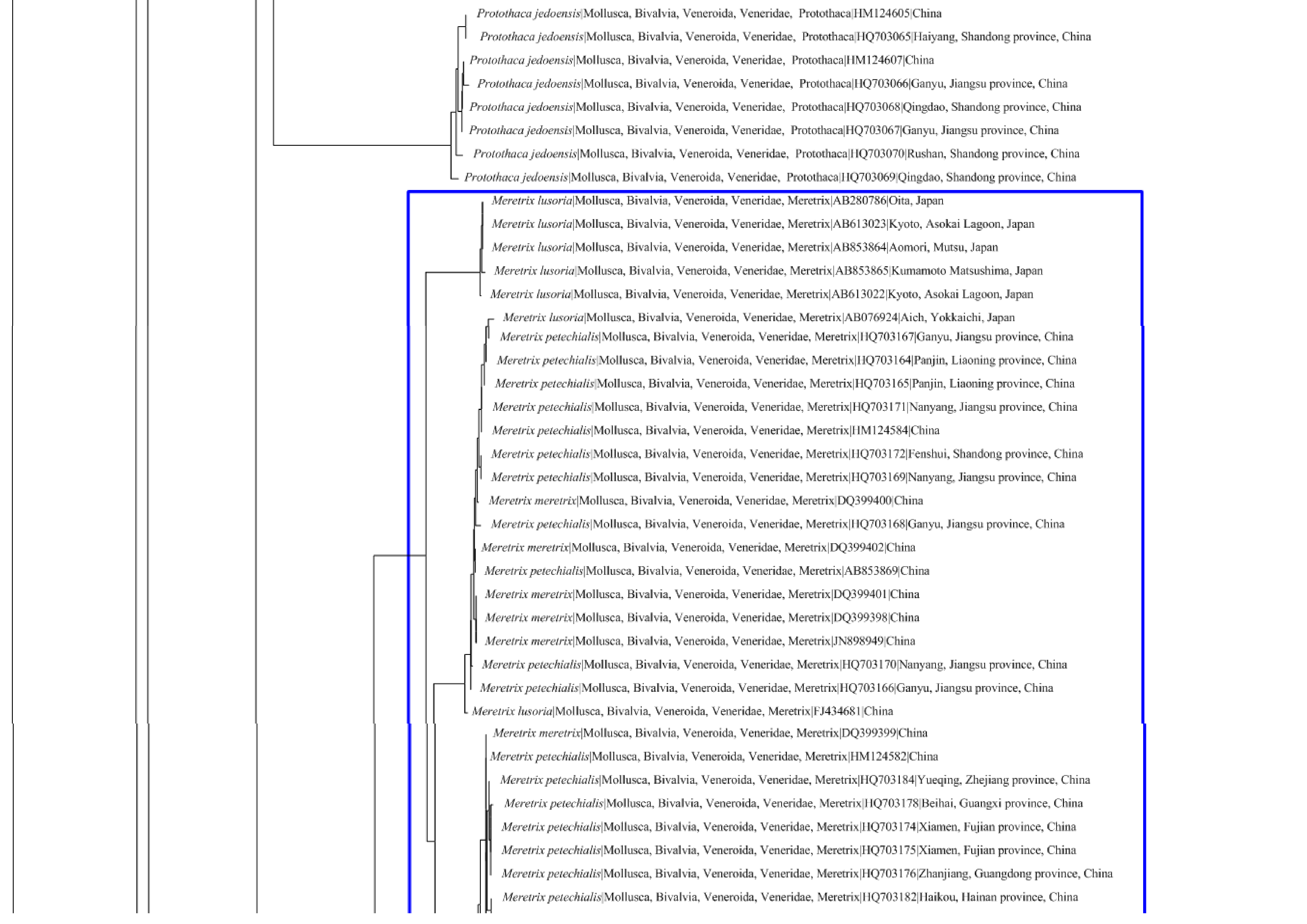
Protothaca jedoensis|Mollusca, Bivalvia, Veneroida, Veneridae, Protothaca|HM124605|China
Protothaca jedoensis|Mollusca, Bivalvia, Veneroida, Veneridae, Protothaca|HQ703065|Haiyang, Shandong province, China
Protothaca jedoensis|Mollusca, Bivalvia, Veneroida, Veneridae, Protothaca|HM124607|China
Protothaca jedoensis|Mollusca, Bivalvia, Veneroida, Veneridae, Protothaca|HQ703066|Ganyu, Jiangsu province, China
Protothaca jedoensis|Mollusca, Bivalvia, Veneroida, Veneridae, Protothaca|HQ703068|Qingdao, Shandong province, China
Protothaca jedoensis|Mollusca, Bivalvia, Veneroida, Veneridae, Protothaca|HQ703067|Ganyu, Jiangsu province, China
Protothaca jedoensis|Mollusca, Bivalvia, Veneroida, Veneridae, Protothaca|HQ703070|Rushan, Shandong province, China
Protothaca jedoensis|Mollusca, Bivalvia, Veneroida, Veneridae, Protothaca|HQ703069|Qingdao, Shandong province, China
Meretrix lusoria|Mollusca, Bivalvia, Veneroida, Veneridae, Meretrix|AB280786|Oita, Japan
Meretrix lusoria|Mollusca, Bivalvia, Veneroida, Veneridae, Meretrix|AB613023|Kyoto, Asokai Lagoon, Japan
Meretrix lusoria|Mollusca, Bivalvia, Veneroida, Veneridae, Meretrix|AB853864|Aomori, Mutsu, Japan
Meretrix lusoria|Mollusca, Bivalvia, Veneroida, Veneridae, Meretrix|AB853865|Kumamoto Matsushima, Japan
Meretrix lusoria|Mollusca, Bivalvia, Veneroida, Veneridae, Meretrix|AB613022|Kyoto, Asokai Lagoon, Japan
Meretrix lusoria|Mollusca, Bivalvia, Veneroida, Veneridae, Meretrix|AB076924|Aich, Yokkaichi, Japan
Meretrix petechialis|Mollusca, Bivalvia, Veneroida, Veneridae, Meretrix|HQ703167|Ganyu, Jiangsu province, China
Meretrix petechialis|Mollusca, Bivalvia, Veneroida, Veneridae, Meretrix|HQ703164|Panjin, Liaoning province, China
Meretrix petechialis|Mollusca, Bivalvia, Veneroida, Veneridae, Meretrix|HQ703165|Panjin, Liaoning province, China
Meretrix petechialis|Mollusca, Bivalvia, Veneroida, Veneridae, Meretrix|HQ703171|Nanyang, Jiangsu province, China
Meretrix petechialis|Mollusca, Bivalvia, Veneroida, Veneridae, Meretrix|HM124584|China
Meretrix petechialis|Mollusca, Bivalvia, Veneroida, Veneridae, Meretrix|HQ703172|Fenshui, Shandong province, China
Meretrix petechialis|Mollusca, Bivalvia, Veneroida, Veneridae, Meretrix|HQ703169|Nanyang, Jiangsu province, China
Meretrix meretrix|Mollusca, Bivalvia, Veneroida, Veneridae, Meretrix|DQ399400|China
Meretrix petechialis|Mollusca, Bivalvia, Veneroida, Veneridae, Meretrix|HQ703168|Ganyu, Jiangsu province, China
Meretrix meretrix|Mollusca, Bivalvia, Veneroida, Veneridae, Meretrix|DQ399402|China
Meretrix petechialis|Mollusca, Bivalvia, Veneroida, Veneridae, Meretrix|AB853869|China
Meretrix meretrix|Mollusca, Bivalvia, Veneroida, Veneridae, Meretrix|DQ399401|China
Meretrix meretrix|Mollusca, Bivalvia, Veneroida, Veneridae, Meretrix|DQ399398|China
Meretrix meretrix|Mollusca, Bivalvia, Veneroida, Veneridae, Meretrix|JN898949|China
Meretrix petechialis|Mollusca, Bivalvia, Veneroida, Veneridae, Meretrix|HQ703170|Nanyang, Jiangsu province, China
Meretrix petechialis|Mollusca, Bivalvia, Veneroida, Veneridae, Meretrix|HQ703166|Ganyu, Jiangsu province, China
Meretrix lusoria|Mollusca, Bivalvia, Veneroida, Veneridae, Meretrix|FJ434681|China
Meretrix meretrix|Mollusca, Bivalvia, Veneroida, Veneridae, Meretrix|DQ399399|China
Meretrix petechialis|Mollusca, Bivalvia, Veneroida, Veneridae, Meretrix|HM124582|China
Meretrix petechialis|Mollusca, Bivalvia, Veneroida, Veneridae, Meretrix|HQ703184|Yueqing, Zhejiang province, China
Meretrix petechialis|Mollusca, Bivalvia, Veneroida, Veneridae, Meretrix|HQ703178|Beihai, Guangxi province, China
Meretrix petechialis|Mollusca, Bivalvia, Veneroida, Veneridae, Meretrix|HQ703174|Xiamen, Fujian province, China
Meretrix petechialis|Mollusca, Bivalvia, Veneroida, Veneridae, Meretrix|HQ703175|Xiamen, Fujian province, China
Meretrix petechialis|Mollusca, Bivalvia, Veneroida, Veneridae, Meretrix|HQ703176|Zhanjiang, Guangdong province, China
Meretrix petechialis|Mollusca, Bivalvia, Veneroida, Veneridae, Meretrix|HQ703182|Haikou, Hainan province, China

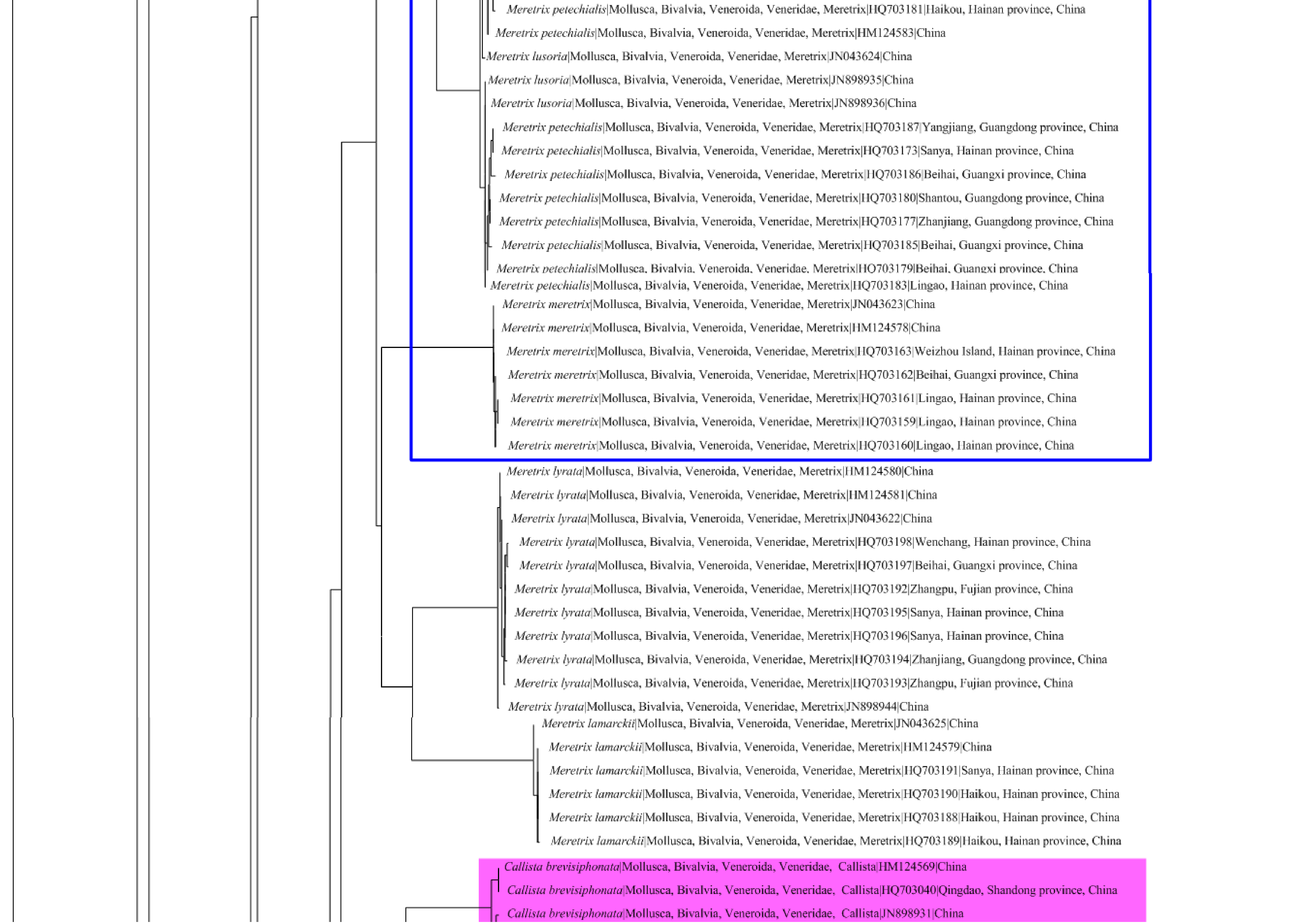

Meretrix petechialis|Mollusca, Bivalvia, Veneroida, Veneridae, Meretrix|HQ703181|Haikou, Hainan province, China
Meretrix petechialis|Mollusca, Bivalvia, Veneroida, Veneridae, Meretrix|HM124583|China
Meretrix lusoria|Mollusca, Bivalvia, Veneroida, Veneridae, Meretrix|JN043624|China
Meretrix lusoria|Mollusca, Bivalvia, Veneroida, Veneridae, Meretrix|JN898935|China
Meretrix lusoria|Mollusca, Bivalvia, Veneroida, Veneridae, Meretrix|JN898936|China
Meretrix petechialis|Mollusca, Bivalvia, Veneroida, Veneridae, Meretrix|HQ703187|Yangjiang, Guangdong province, China
Meretrix petechialis|Mollusca, Bivalvia, Veneroida, Veneridae, Meretrix|HQ703173|Sanya, Hainan province, China
Meretrix petechialis|Mollusca, Bivalvia, Veneroida, Veneridae, Meretrix|HQ703186|Beihai, Guangxi province, China
Meretrix petechialis|Mollusca, Bivalvia, Veneroida, Veneridae, Meretrix|HQ703180|Shantou, Guangdong province, China
Meretrix petechialis|Mollusca, Bivalvia, Veneroida, Veneridae, Meretrix|HQ703177|Zhanjiang, Guangdong province, China
Meretrix petechialis|Mollusca, Bivalvia, Veneroida, Veneridae, Meretrix|HQ703185|Beihai, Guangxi province, China
Meretrix petechialis|Mollusca, Bivalvia, Veneroida, Veneridae, Meretrix|HQ703179|Beihai, Guangxi province, China
Meretrix petechialis|Mollusca, Bivalvia, Veneroida, Veneridae, Meretrix|HQ703183|Lingao, Hainan province, China
Meretrix meretrix|Mollusca, Bivalvia, Veneroida, Veneridae, Meretrix|JN043623|China
Meretrix meretrix|Mollusca, Bivalvia, Veneroida, Veneridae, Meretrix|HM124578|China
Meretrix meretrix|Mollusca, Bivalvia, Veneroida, Veneridae, Meretrix|HQ703163|Weizhou Island, Hainan province, China
Meretrix meretrix|Mollusca, Bivalvia, Veneroida, Veneridae, Meretrix|HQ703162|Beihai, Guangxi province, China
Meretrix meretrix|Mollusca, Bivalvia, Veneroida, Veneridae, Meretrix|HQ703161|Lingao, Hainan province, China
Meretrix meretrix|Mollusca, Bivalvia, Veneroida, Veneridae, Meretrix|HQ703159|Lingao, Hainan province, China
Meretrix meretrix|Mollusca, Bivalvia, Veneroida, Veneridae, Meretrix|HQ703160|Lingao, Hainan province, China
Meretrix lyrata|Mollusca, Bivalvia, Veneroida, Veneridae, Meretrix|HM124580|China
Meretrix lyrata|Mollusca, Bivalvia, Veneroida, Veneridae, Meretrix|HM124581|China
Meretrix lyrata|Mollusca, Bivalvia, Veneroida, Veneridae, Meretrix|JN043622|China
Meretrix lyrata|Mollusca, Bivalvia, Veneroida, Veneridae, Meretrix|HQ703198|Wenchang, Hainan province, China
Meretrix lyrata|Mollusca, Bivalvia, Veneroida, Veneridae, Meretrix|HQ703197|Beihai, Guangxi province, China
Meretrix lyrata|Mollusca, Bivalvia, Veneroida, Veneridae, Meretrix|HQ703192|Zhangpu, Fujian province, China
Meretrix lyrata|Mollusca, Bivalvia, Veneroida, Veneridae, Meretrix|HQ703195|Sanya, Hainan province, China
Meretrix lyrata|Mollusca, Bivalvia, Veneroida, Veneridae, Meretrix|HQ703196|Sanya, Hainan province, China
Meretrix lyrata|Mollusca, Bivalvia, Veneroida, Veneridae, Meretrix|HQ703194|Zhanjiang, Guangdong province, China
Meretrix lyrata|Mollusca, Bivalvia, Veneroida, Veneridae, Meretrix|HQ703193|Zhangpu, Fujian province, China
Meretrix lyrata|Mollusca, Bivalvia, Veneroida, Veneridae, Meretrix|JN898944|China
Meretrix lamarckii|Mollusca, Bivalvia, Veneroida, Veneridae, Meretrix|JN043625|China
Meretrix lamarckii|Mollusca, Bivalvia, Veneroida, Veneridae, Meretrix|HM124579|China
Meretrix lamarckii|Mollusca, Bivalvia, Veneroida, Veneridae, Meretrix|HQ703191|Sanya, Hainan province, China
Meretrix lamarckii|Mollusca, Bivalvia, Veneroida, Veneridae, Meretrix|HQ703190|Haikou, Hainan province, China
Meretrix lamarckii|Mollusca, Bivalvia, Veneroida, Veneridae, Meretrix|HQ703188|Haikou, Hainan province, China
Meretrix lamarckii|Mollusca, Bivalvia, Veneroida, Veneridae, Meretrix|HQ703189|Haikou, Hainan province, China
Callista brevisiphonata|Mollusca, Bivalvia, Veneroida, Veneridae, Callista|HM124569|China
Callista brevisiphonata|Mollusca, Bivalvia, Veneroida, Veneridae, Callista|HQ703040|Qingdao, Shandong province, China
Callista brevisiphonata|Mollusca, Bivalvia, Veneroida, Veneridae, Callista|JN898931|China

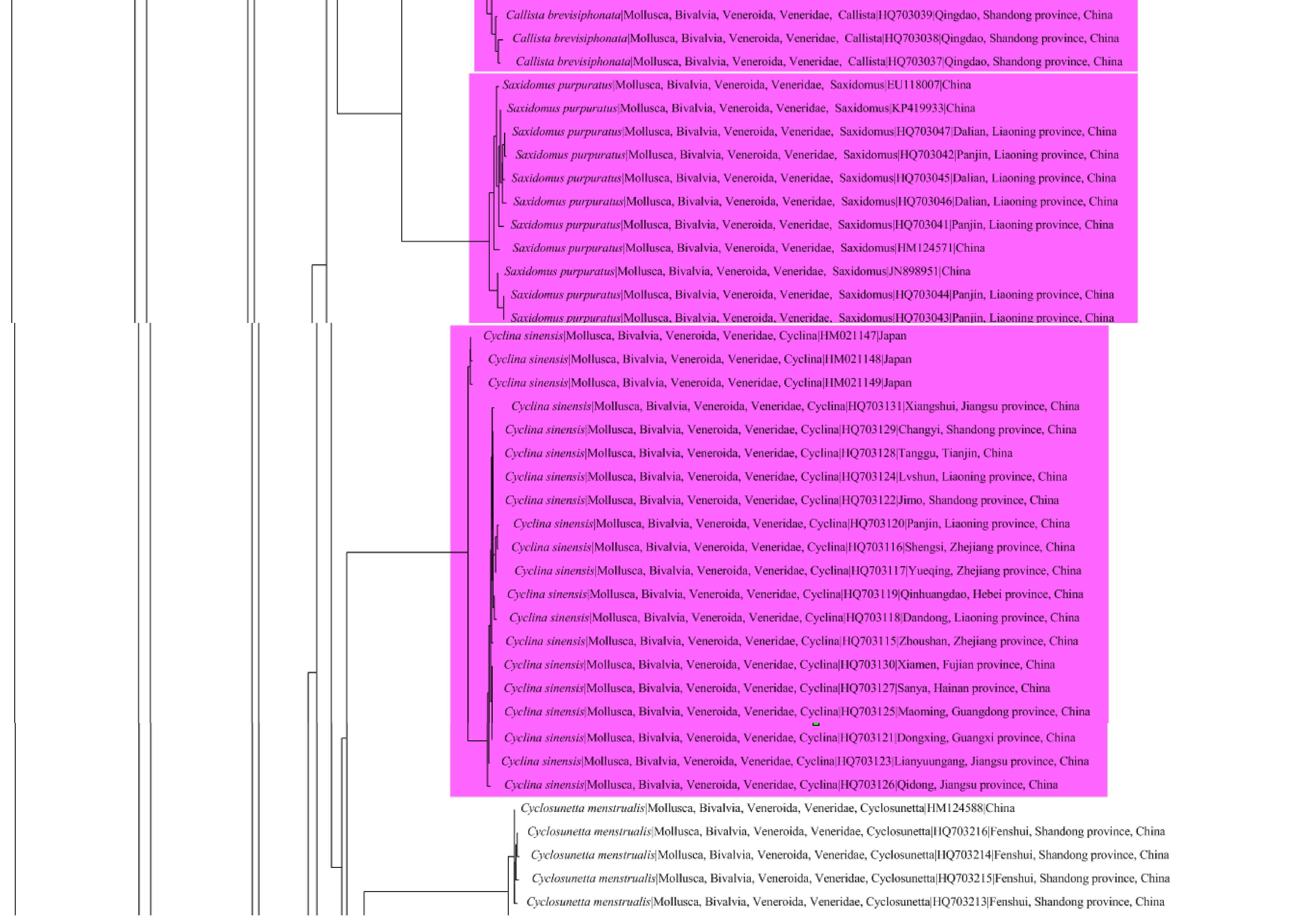
Callista brevisiphonata|Mollusca, Bivalvia, Veneroida, Veneridae, Callista|HQ703039|Qingdao, Shandong province, China
Callista brevisiphonata|Mollusca, Bivalvia, Veneroida, Veneridae, Callista|HQ703038|Qingdao, Shandong province, China
Callista brevisiphonata|Mollusca, Bivalvia, Veneroida, Veneridae, Callista|HQ703037|Qingdao, Shandong province, China
Saxidomus purpuratus|Mollusca, Bivalvia, Veneroida, Veneridae, Saxidomus|EU118007|China
Saxidomus purpuratus|Mollusca, Bivalvia, Veneroida, Veneridae, Saxidomus|KP419933|China
Saxidomus purpuratus|Mollusca, Bivalvia, Veneroida, Veneridae, Saxidomus|HQ703047|Dalian, Liaoning province, China
Saxidomus purpuratus|Mollusca, Bivalvia, Veneroida, Veneridae, Saxidomus|HQ703042|Panjin, Liaoning province, China
Saxidomus purpuratus|Mollusca, Bivalvia, Veneroida, Veneridae, Saxidomus|HQ703045|Dalian, Liaoning province, China
Saxidomus purpuratus|Mollusca, Bivalvia, Veneroida, Veneridae, Saxidomus|HQ703046|Dalian, Liaoning province, China
Saxidomus purpuratus|Mollusca, Bivalvia, Veneroida, Veneridae, Saxidomus|HQ703041|Panjin, Liaoning province, China
Saxidomus purpuratus|Mollusca, Bivalvia, Veneroida, Veneridae, Saxidomus|HM124571|China
Saxidomus purpuratus|Mollusca, Bivalvia, Veneroida, Veneridae, Saxidomus|JN898951|China
Saxidomus purpuratus|Mollusca, Bivalvia, Veneroida, Veneridae, Saxidomus|HQ703044|Panjin, Liaoning province, China
Saxidomus purpuratus|Mollusca, Bivalvia, Veneroida, Veneridae, Saxidomus|HQ703043|Panjin, Liaoning province, China
Cyclina sinensis|Mollusca, Bivalvia, Veneroida, Veneridae, Cyclina|HM021147|Japan
Cyclina sinensis|Mollusca, Bivalvia, Veneroida, Veneridae, Cyclina|HM021148|Japan
Cyclina sinensis|Mollusca, Bivalvia, Veneroida, Veneridae, Cyclina|HM021149|Japan
Cyclina sinensis|Mollusca, Bivalvia, Veneroida, Veneridae, Cyclina|HQ703131|Xiangshui, Jiangsu province, China
Cyclina sinensis|Mollusca, Bivalvia, Veneroida, Veneridae, Cyclina|HQ703129|Changyi, Shandong province, China
Cyclina sinensis|Mollusca, Bivalvia, Veneroida, Veneridae, Cyclina|HQ703128|Tanggu, Tianjin, China
Cyclina sinensis|Mollusca, Bivalvia, Veneroida, Veneridae, Cyclina|HQ703124|Lvshun, Liaoning province, China
Cyclina sinensis|Mollusca, Bivalvia, Veneroida, Veneridae, Cyclina|HQ703122|Jimo, Shandong province, China
Cyclina sinensis|Mollusca, Bivalvia, Veneroida, Veneridae, Cyclina|HQ703120|Panjin, Liaoning province, China
Cyclina sinensis|Mollusca, Bivalvia, Veneroida, Veneridae, Cyclina|HQ703116|Shengsi, Zhejiang province, China
Cyclina sinensis|Mollusca, Bivalvia, Veneroida, Veneridae, Cyclina|HQ703117|Yueqing, Zhejiang province, China
Cyclina sinensis|Mollusca, Bivalvia, Veneroida, Veneridae, Cyclina|HQ703119|Qinhuangdao, Hebei province, China
Cyclina sinensis|Mollusca, Bivalvia, Veneroida, Veneridae, Cyclina|HQ703118|Dandong, Liaoning province, China
Cyclina sinensis|Mollusca, Bivalvia, Veneroida, Veneridae, Cyclina|HQ703115|Zhoushan, Zhejiang province, China
Cyclina sinensis|Mollusca, Bivalvia, Veneroida, Veneridae, Cyclina|HQ703130|Xiamen, Fujian province, China
Cyclina sinensis|Mollusca, Bivalvia, Veneroida, Veneridae, Cyclina|HQ703127|Sanya, Hainan province, China
Cyclina sinensis|Mollusca, Bivalvia, Veneroida, Veneridae, Cyclina|HQ703125|Maoming, Guangdong province, China
Cyclina sinensis|Mollusca, Bivalvia, Veneroida, Veneridae, Cyclina|HQ703121|Dongxing, Guangxi province, China
Cyclina sinensis|Mollusca, Bivalvia, Veneroida, Veneridae, Cyclina|HQ703123|Lianyuungang, Jiangsu province, China
Cyclina sinensis|Mollusca, Bivalvia, Veneroida, Veneridae, Cyclina|HQ703126|Qidong, Jiangsu province, China
Cyclosunetta menstrualis|Mollusca, Bivalvia, Veneroida, Veneridae, Cyclosunetta|HM124588|China
Cyclosunetta menstrualis|Mollusca, Bivalvia, Veneroida, Veneridae, Cyclosunetta|HQ703216|Fenshui, Shandong province, China
Cyclosunetta menstrualis|Mollusca, Bivalvia, Veneroida, Veneridae, Cyclosunetta|HQ703214|Fenshui, Shandong province, China
Cyclosunetta menstrualis|Mollusca, Bivalvia, Veneroida, Veneridae, Cyclosunetta|HQ703215|Fenshui, Shandong province, China
Cyclosunetta menstrualis|Mollusca, Bivalvia, Veneroida, Veneridae, Cyclosunetta|HQ703213|Fenshui, Shandong province, China

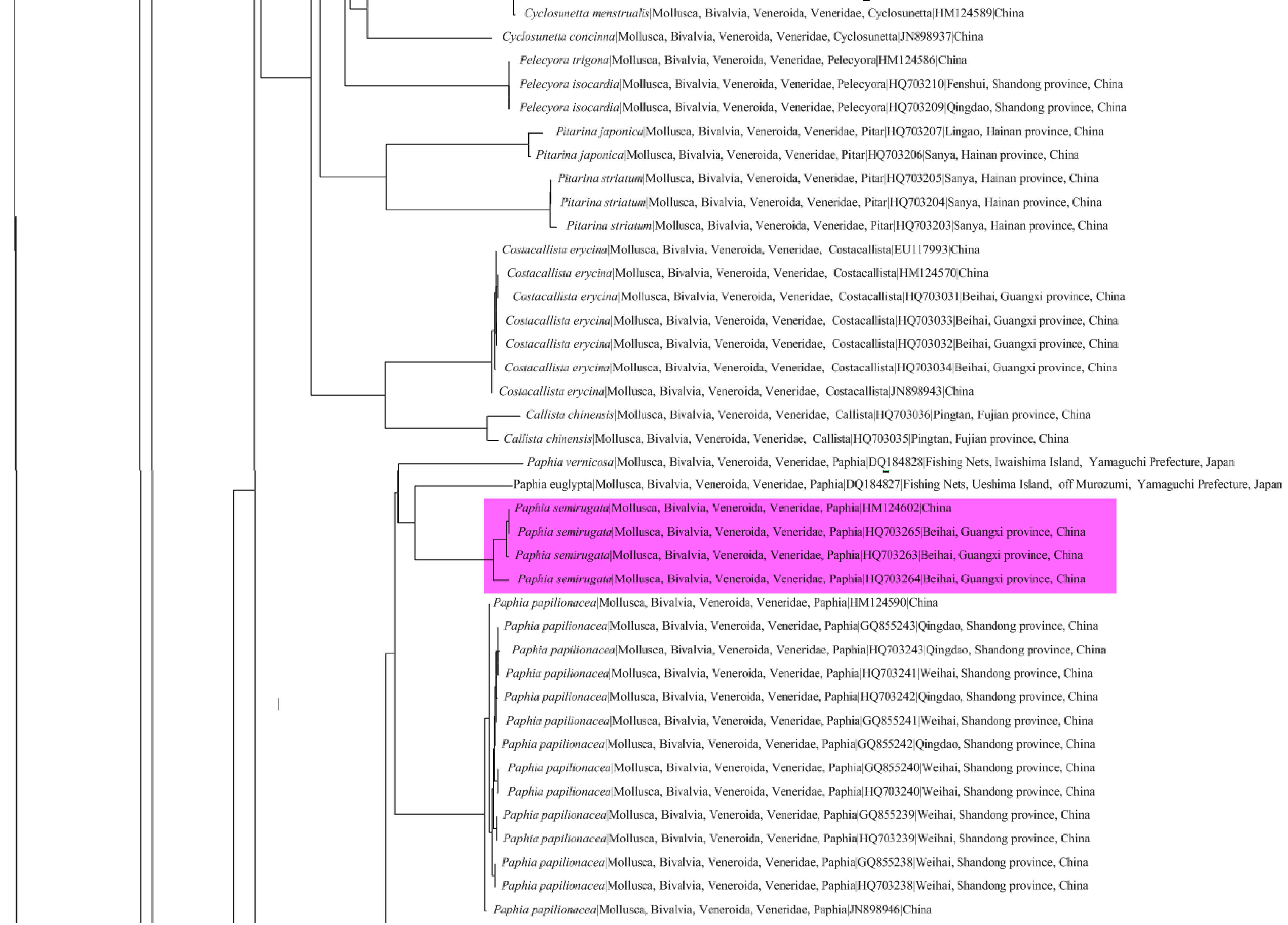
Cyclosunetta menstrualis|Mollusca, Bivalvia, Veneroida, Veneridae, Cyclosunetta|HM124589|China
Cyclosunetta concinna|Mollusca, Bivalvia, Veneroida, Veneridae, Cyclosunetta|JN898937|China
Pelecyora trigona|Mollusca, Bivalvia, Veneroida, Veneridae, Pelecyora|HM124586|China
Pelecyora isocardia|Mollusca, Bivalvia, Veneroida, Veneridae, Pelecyora|HQ703210|Fenshui, Shandong province, China
Pelecyora isocardia|Mollusca, Bivalvia, Veneroida, Veneridae, Pelecyora|HQ703209|Qingdao, Shandong province, China
Pitarina japonica|Mollusca, Bivalvia, Veneroida, Veneridae, Pitar|HQ703207|Lingao, Hainan province, China
Pitarina japonica|Mollusca, Bivalvia, Veneroida, Veneridae, Pitar|HQ703206|Sanya, Hainan province, China
Pitarina striatum|Mollusca, Bivalvia, Veneroida, Veneridae, Pitar|HQ703205|Sanya, Hainan province, China
Pitarina striatum|Mollusca, Bivalvia, Veneroida, Veneridae, Pitar|HQ703204|Sanya, Hainan province, China
Pitarina striatum|Mollusca, Bivalvia, Veneroida, Veneridae, Pitar|HQ703203|Sanya, Hainan province, China
Costacallista erycina|Mollusca, Bivalvia, Veneroida, Veneridae, Costacallista|EU117993|China
Costacallista erycina|Mollusca, Bivalvia, Veneroida, Veneridae, Costacallista|HM124570|China
Costacallista erycina|Mollusca, Bivalvia, Veneroida, Veneridae, Costacallista|HQ703031|Beihai, Guangxi province, China
Costacallista erycina|Mollusca, Bivalvia, Veneroida, Veneridae, Costacallista|HQ703033|Beihai, Guangxi province, China
Costacallista erycina|Mollusca, Bivalvia, Veneroida, Veneridae, Costacallista|HQ703032|Beihai, Guangxi province, China
Costacallista erycina|Mollusca, Bivalvia, Veneroida, Veneridae, Costacallista|HQ703034|Beihai, Guangxi province, China
Costacallista erycina|Mollusca, Bivalvia, Veneroida, Veneridae, Costacallista|JN898943|China
Callista chinensis|Mollusca, Bivalvia, Veneroida, Veneridae, Callista|HQ703036|Pingtan, Fujian province, China
Callista chinensis|Mollusca, Bivalvia, Veneroida, Veneridae, Callista|HQ703035|Pingtan, Fujian province, China
Paphia vernicosa|Mollusca, Bivalvia, Veneroida, Veneridae, Paphia|DQ184828|Fishing Nets, Iwaishima Island, Yamaguchi Prefecture, Japan
Paphia euglypta|Mollusca, Bivalvia, Veneroida, Veneridae, Paphia|DQ184827|Fishing Nets, Ueshima Island, off Murozumi, Yamaguchi Prefecture, Japan
Paphia semirugata|Mollusca, Bivalvia, Veneroida, Veneridae, Paphia|HM124602|China
Paphia semirugata|Mollusca, Bivalvia, Veneroida, Veneridae, Paphia|HQ703265|Beihai, Guangxi province, China
Paphia semirugata|Mollusca, Bivalvia, Veneroida, Veneridae, Paphia|HQ703263|Beihai, Guangxi province, China
Paphia semirugata|Mollusca, Bivalvia, Veneroida, Veneridae, Paphia|HQ703264|Beihai, Guangxi province, China
Paphia papilionacea|Mollusca, Bivalvia, Veneroida, Veneridae, Paphia|HM124590|China
Paphia papilionacea|Mollusca, Bivalvia, Veneroida, Veneridae, Paphia|GQ855243|Qingdao, Shandong province, China
Paphia papilionacea|Mollusca, Bivalvia, Veneroida, Veneridae, Paphia|HQ703243|Qingdao, Shandong province, China
Paphia papilionacea|Mollusca, Bivalvia, Veneroida, Veneridae, Paphia|HQ703241|Weihai, Shandong province, China
Paphia papilionacea|Mollusca, Bivalvia, Veneroida, Veneridae, Paphia|HQ703242|Qingdao, Shandong province, China
Paphia papilionacea|Mollusca, Bivalvia, Veneroida, Veneridae, Paphia|GQ855241|Weihai, Shandong province, China
Paphia papilionacea|Mollusca, Bivalvia, Veneroida, Veneridae, Paphia|GQ855242|Qingdao, Shandong province, China
Paphia papilionacea|Mollusca, Bivalvia, Veneroida, Veneridae, Paphia|GQ855240|Weihai, Shandong province, China
Paphia papilionacea|Mollusca, Bivalvia, Veneroida, Veneridae, Paphia|HQ703240|Weihai, Shandong province, China
Paphia papilionacea|Mollusca, Bivalvia, Veneroida, Veneridae, Paphia|GQ855239|Weihai, Shandong province, China
Paphia papilionacea|Mollusca, Bivalvia, Veneroida, Veneridae, Paphia|HQ703239|Weihai, Shandong province, China
Paphia papilionacea|Mollusca, Bivalvia, Veneroida, Veneridae, Paphia|GQ855238|Weihai, Shandong province, China
Paphia papilionacea|Mollusca, Bivalvia, Veneroida, Veneridae, Paphia|HQ703238|Weihai, Shandong province, China
Paphia papilionacea|Mollusca, Bivalvia, Veneroida, Veneridae, Paphia|JN898946|China

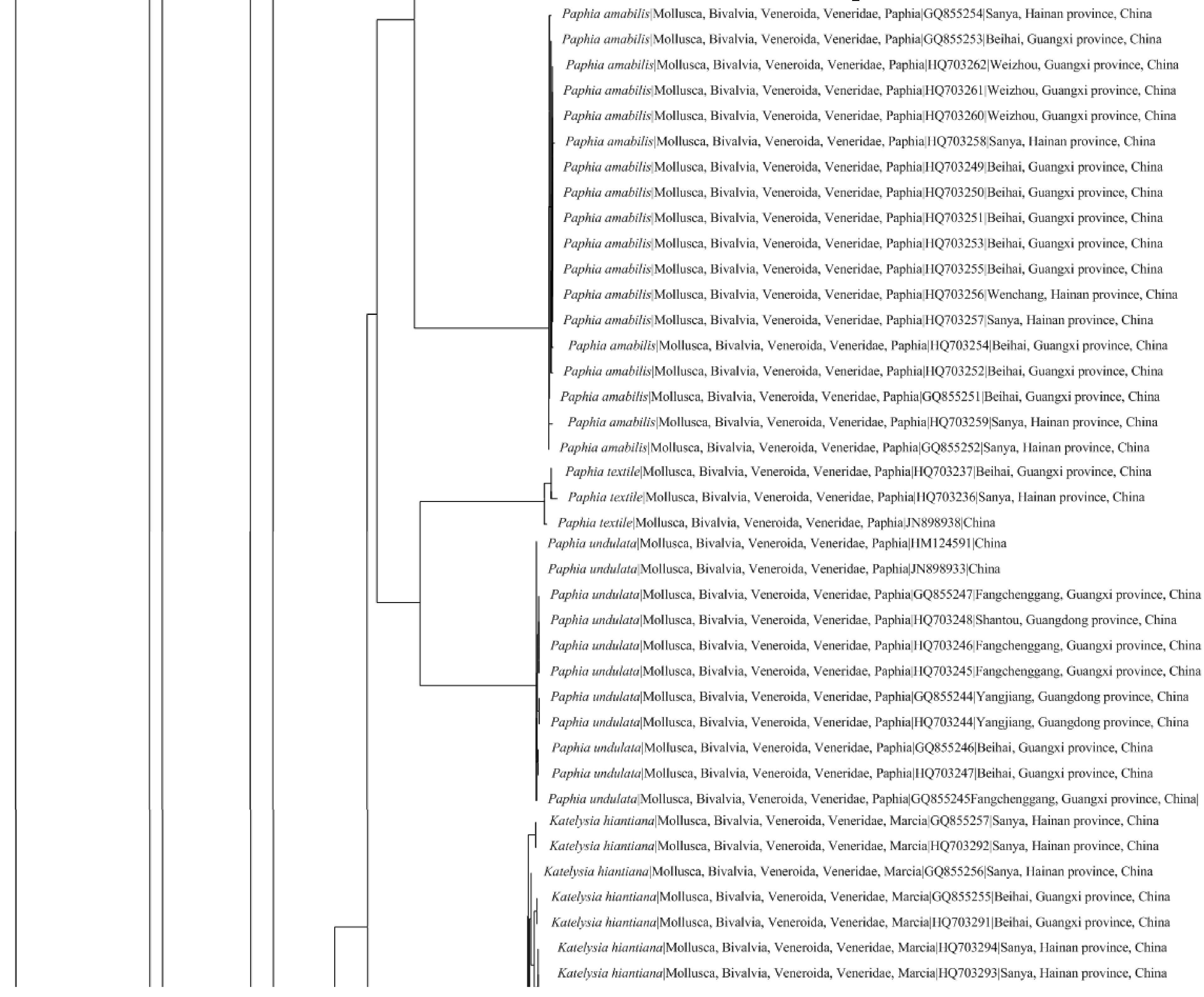
Paphia amabilis|Mollusca, Bivalvia, Veneroida, Veneridae, Paphia|GQ855254|Sanya, Hainan province, China
Paphia amabilis|Mollusca, Bivalvia, Veneroida, Veneridae, Paphia|GQ855253|Beihai, Guangxi province, China
Paphia amabilis|Mollusca, Bivalvia, Veneroida, Veneridae, Paphia|HQ703262|Weizhou, Guangxi province, China
Paphia amabilis|Mollusca, Bivalvia, Veneroida, Veneridae, Paphia|HQ703261|Weizhou, Guangxi province, China
Paphia amabilis|Mollusca, Bivalvia, Veneroida, Veneridae, Paphia|HQ703260|Weizhou, Guangxi province, China
Paphia amabilis|Mollusca, Bivalvia, Veneroida, Veneridae, Paphia|HQ703258|Sanya, Hainan province, China
Paphia amabilis|Mollusca, Bivalvia, Veneroida, Veneridae, Paphia|HQ703249|Beihai, Guangxi province, China
Paphia amabilis|Mollusca, Bivalvia, Veneroida, Veneridae, Paphia|HQ703250|Beihai, Guangxi province, China
Paphia amabilis|Mollusca, Bivalvia, Veneroida, Veneridae, Paphia|HQ703251|Beihai, Guangxi province, China
Paphia amabilis|Mollusca, Bivalvia, Veneroida, Veneridae, Paphia|HQ703253|Beihai, Guangxi province, China
Paphia amabilis|Mollusca, Bivalvia, Veneroida, Veneridae, Paphia|HQ703255|Beihai, Guangxi province, China
Paphia amabilis|Mollusca, Bivalvia, Veneroida, Veneridae, Paphia|HQ703256|Wenchang, Hainan province, China
Paphia amabilis|Mollusca, Bivalvia, Veneroida, Veneridae, Paphia|HQ703257|Sanya, Hainan province, China
Paphia amabilis|Mollusca, Bivalvia, Veneroida, Veneridae, Paphia|HQ703254|Beihai, Guangxi province, China
Paphia amabilis|Mollusca, Bivalvia, Veneroida, Veneridae, Paphia|HQ703252|Beihai, Guangxi province, China
Paphia amabilis|Mollusca, Bivalvia, Veneroida, Veneridae, Paphia|GQ855251|Beihai, Guangxi province, China
Paphia amabilis|Mollusca, Bivalvia, Veneroida, Veneridae, Paphia|HQ703259|Sanya, Hainan province, China
Paphia amabilis|Mollusca, Bivalvia, Veneroida, Veneridae, Paphia|GQ855252|Sanya, Hainan province, China
Paphia textile|Mollusca, Bivalvia, Veneroida, Veneridae, Paphia|HQ703237|Beihai, Guangxi province, China
Paphia textile|Mollusca, Bivalvia, Veneroida, Veneridae, Paphia|HQ703236|Sanya, Hainan province, China
Paphia textile|Mollusca, Bivalvia, Veneroida, Veneridae, Paphia|JN898938|China
Paphia undulata|Mollusca, Bivalvia, Veneroida, Veneridae, Paphia|HM124591|China
Paphia undulata|Mollusca, Bivalvia, Veneroida, Veneridae, Paphia|JN898933|China
Paphia undulata|Mollusca, Bivalvia, Veneroida, Veneridae, Paphia|GQ855247|Fangchenggang, Guangxi province, China
Paphia undulata|Mollusca, Bivalvia, Veneroida, Veneridae, Paphia|HQ703248|Shantou, Guangdong province, China
Paphia undulata|Mollusca, Bivalvia, Veneroida, Veneridae, Paphia|HQ703246|Fangchenggang, Guangxi province, China
Paphia undulata|Mollusca, Bivalvia, Veneroida, Veneridae, Paphia|HQ703245|Fangchenggang, Guangxi province, China
Paphia undulata|Mollusca, Bivalvia, Veneroida, Veneridae, Paphia|GQ855244|Yangjiang, Guangdong province, China
Paphia undulata|Mollusca, Bivalvia, Veneroida, Veneridae, Paphia|HQ703244|Yangjiang, Guangdong province, China
Paphia undulata|Mollusca, Bivalvia, Veneroida, Veneridae, Paphia|GQ855246|Beihai, Guangxi province, China
Paphia undulata|Mollusca, Bivalvia, Veneroida, Veneridae, Paphia|HQ703247|Beihai, Guangxi province, China
Paphia undulata|Mollusca, Bivalvia, Veneroida, Veneridae, Paphia|GQ855245Fangchenggang, Guangxi province, China|
Katelysia hiantiana|Mollusca, Bivalvia, Veneroida, Veneridae, Marcia|GQ855257|Sanya, Hainan province, China
Katelysia hiantiana|Mollusca, Bivalvia, Veneroida, Veneridae, Marcia|HQ703292|Sanya, Hainan province, China
Katelysia hiantiana|Mollusca, Bivalvia, Veneroida, Veneridae, Marcia|GQ855256|Sanya, Hainan province, China
Katelysia hiantiana|Mollusca, Bivalvia, Veneroida, Veneridae, Marcia|GQ855255|Beihai, Guangxi province, China
Katelysia hiantiana|Mollusca, Bivalvia, Veneroida, Veneridae, Marcia|HQ703291|Beihai, Guangxi province, China
Katelysia hiantiana|Mollusca, Bivalvia, Veneroida, Veneridae, Marcia|HQ703294|Sanya, Hainan province, China
Katelysia hiantiana|Mollusca, Bivalvia, Veneroida, Veneridae, Marcia|HQ703293|Sanya, Hainan province, China

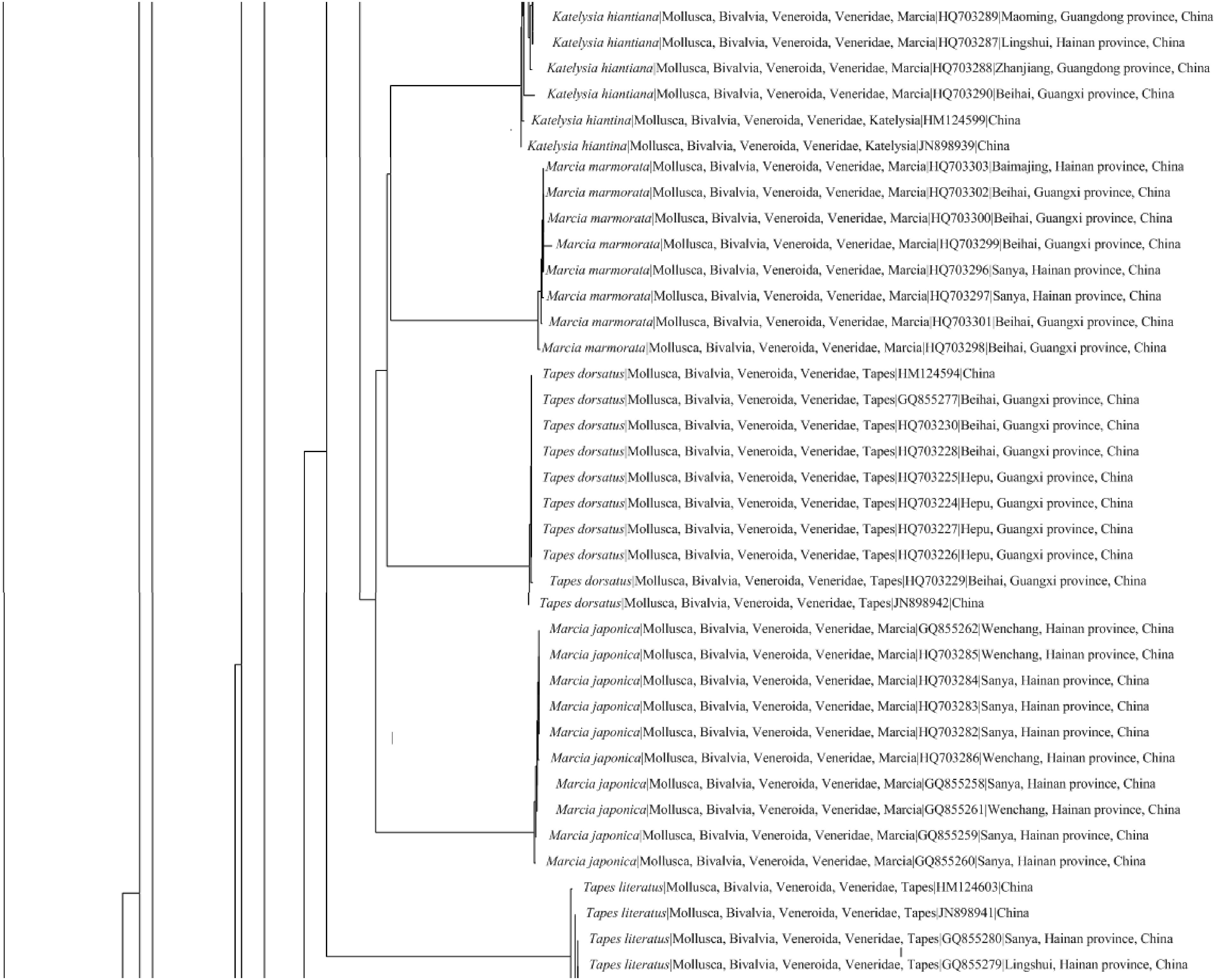
Katelysia hiantiana|Mollusca, Bivalvia, Veneroida, Veneridae, Marcia|HQ703289|Maoming, Guangdong province, China
Katelysia hiantiana|Mollusca, Bivalvia, Veneroida, Veneridae, Marcia|HQ703287|Lingshui, Hainan province, China
Katelysia hiantiana|Mollusca, Bivalvia, Veneroida, Veneridae, Marcia|HQ703288|Zhanjiang, Guangdong province, China
Katelysia hiantiana|Mollusca, Bivalvia, Veneroida, Veneridae, Marcia|HQ703290|Beihai, Guangxi province, China
Katelysia hiantina|Mollusca, Bivalvia, Veneroida, Veneridae, Katelysia|HM124599|China
Katelysia hiantina|Mollusca, Bivalvia, Veneroida, Veneridae, Katelysia|JN898939|China
Marcia marmorata|Mollusca, Bivalvia, Veneroida, Veneridae, Marcia|HQ703303|Baimajing, Hainan province, China
Marcia marmorata|Mollusca, Bivalvia, Veneroida, Veneridae, Marcia|HQ703302|Beihai, Guangxi province, China
Marcia marmorata|Mollusca, Bivalvia, Veneroida, Veneridae, Marcia|HQ703300|Beihai, Guangxi province, China
Marcia marmorata|Mollusca, Bivalvia, Veneroida, Veneridae, Marcia|HQ703299|Beihai, Guangxi province, China
Marcia marmorata|Mollusca, Bivalvia, Veneroida, Veneridae, Marcia|HQ703296|Sanya, Hainan province, China
Marcia marmorata|Mollusca, Bivalvia, Veneroida, Veneridae, Marcia|HQ703297|Sanya, Hainan province, China
Marcia marmorata|Mollusca, Bivalvia, Veneroida, Veneridae, Marcia|HQ703301|Beihai, Guangxi province, China
Marcia marmorata|Mollusca, Bivalvia, Veneroida, Veneridae, Marcia|HQ703298|Beihai, Guangxi province, China
Tapes dorsatus|Mollusca, Bivalvia, Veneroida, Veneridae, Tapes|HM124594|China
Tapes dorsatus|Mollusca, Bivalvia, Veneroida, Veneridae, Tapes|GQ855277|Beihai, Guangxi province, China
Tapes dorsatus|Mollusca, Bivalvia, Veneroida, Veneridae, Tapes|HQ703230|Beihai, Guangxi province, China
Tapes dorsatus|Mollusca, Bivalvia, Veneroida, Veneridae, Tapes|HQ703228|Beihai, Guangxi province, China
Tapes dorsatus|Mollusca, Bivalvia, Veneroida, Veneridae, Tapes|HQ703225|Hepu, Guangxi province, China
Tapes dorsatus|Mollusca, Bivalvia, Veneroida, Veneridae, Tapes|HQ703224|Hepu, Guangxi province, China
Tapes dorsatus|Mollusca, Bivalvia, Veneroida, Veneridae, Tapes|HQ703227|Hepu, Guangxi province, China
Tapes dorsatus|Mollusca, Bivalvia, Veneroida, Veneridae, Tapes|HQ703226|Hepu, Guangxi province, China
Tapes dorsatus|Mollusca, Bivalvia, Veneroida, Veneridae, Tapes|HQ703229|Beihai, Guangxi province, China
Tapes dorsatus|Mollusca, Bivalvia, Veneroida, Veneridae, Tapes|JN898942|China
Marcia japonica|Mollusca, Bivalvia, Veneroida, Veneridae, Marcia|GQ855262|Wenchang, Hainan province, China
Marcia japonica|Mollusca, Bivalvia, Veneroida, Veneridae, Marcia|HQ703285|Wenchang, Hainan province, China
Marcia japonica|Mollusca, Bivalvia, Veneroida, Veneridae, Marcia|HQ703284|Sanya, Hainan province, China
Marcia japonica|Mollusca, Bivalvia, Veneroida, Veneridae, Marcia|HQ703283|Sanya, Hainan province, China
Marcia japonica|Mollusca, Bivalvia, Veneroida, Veneridae, Marcia|HQ703282|Sanya, Hainan province, China
Marcia japonica|Mollusca, Bivalvia, Veneroida, Veneridae, Marcia|HQ703286|Wenchang, Hainan province, China
Marcia japonica|Mollusca, Bivalvia, Veneroida, Veneridae, Marcia|GQ855258|Sanya, Hainan province, China
Marcia japonica|Mollusca, Bivalvia, Veneroida, Veneridae, Marcia|GQ855261|Wenchang, Hainan province, China
Marcia japonica|Mollusca, Bivalvia, Veneroida, Veneridae, Marcia|GQ855259|Sanya, Hainan province, China
Marcia japonica|Mollusca, Bivalvia, Veneroida, Veneridae, Marcia|GQ855260|Sanya, Hainan province, China
Tapes literatus|Mollusca, Bivalvia, Veneroida, Veneridae, Tapes|HM124603|China
Tapes literatus|Mollusca, Bivalvia, Veneroida, Veneridae, Tapes|JN898941|China
Tapes literatus|Mollusca, Bivalvia, Veneroida, Veneridae, Tapes|GQ855280|Sanya, Hainan province, China
Tapes literatus|Mollusca, Bivalvia, Veneroida, Veneridae, Tapes|GQ855279|Lingshui, Hainan province, China

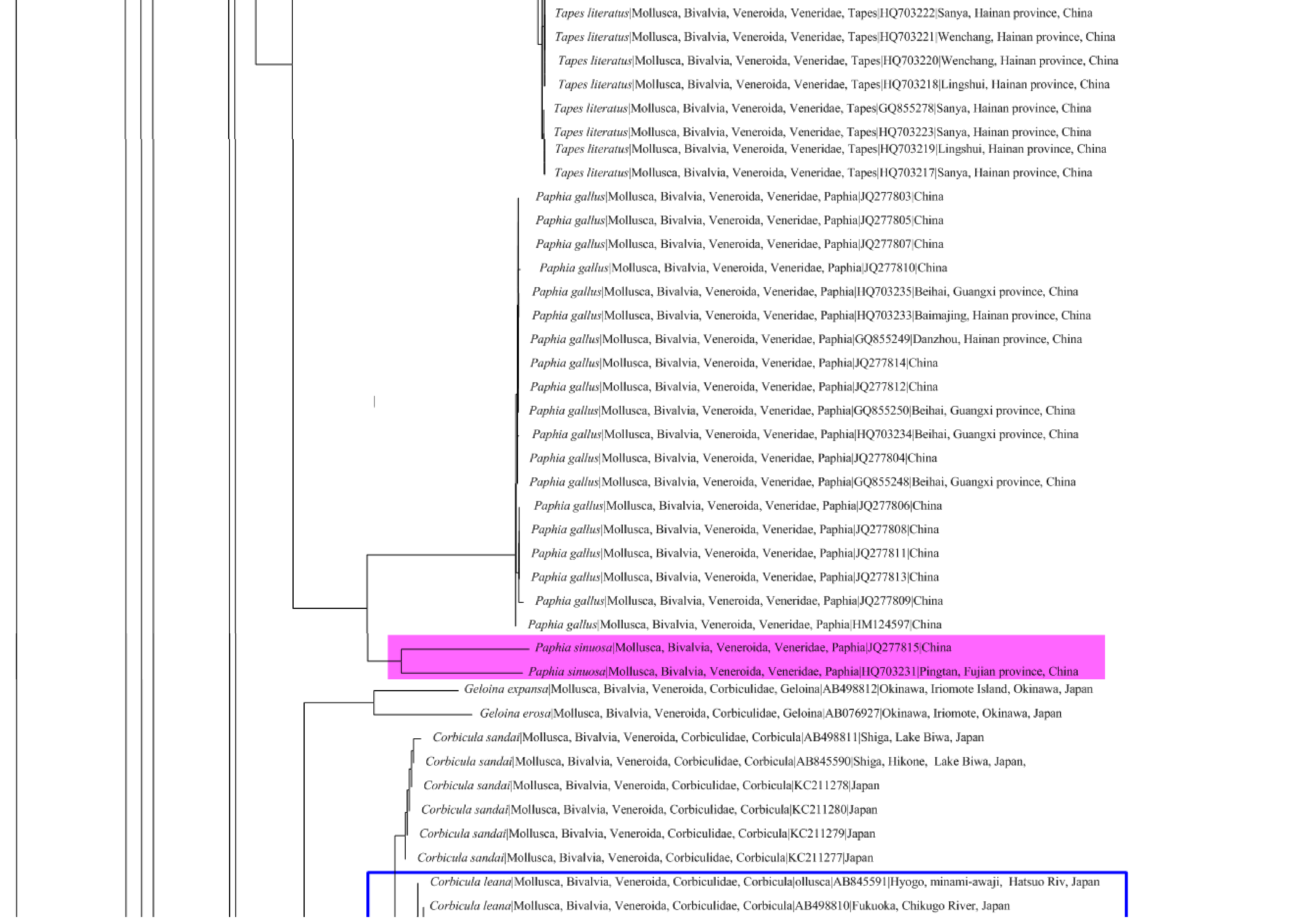

Tapes literatus|Mollusca, Bivalvia, Veneroida, Veneridae, Tapes|HQ703222|Sanya, Hainan province, China
Tapes literatus|Mollusca, Bivalvia, Veneroida, Veneridae, Tapes|HQ703221|Wenchang, Hainan province, China
Tapes literatus|Mollusca, Bivalvia, Veneroida, Veneridae, Tapes|HQ703220|Wenchang, Hainan province, China
Tapes literatus|Mollusca, Bivalvia, Veneroida, Veneridae, Tapes|HQ703218|Lingshui, Hainan province, China
Tapes literatus|Mollusca, Bivalvia, Veneroida, Veneridae, Tapes|GQ855278|Sanya, Hainan province, China
Tapes literatus|Mollusca, Bivalvia, Veneroida, Veneridae, Tapes|HQ703223|Sanya, Hainan province, China
Tapes literatus|Mollusca, Bivalvia, Veneroida, Veneridae, Tapes|HQ703219|Lingshui, Hainan province, China
Tapes literatus|Mollusca, Bivalvia, Veneroida, Veneridae, Tapes|HQ703217|Sanya, Hainan province, China
Paphia gallus|Mollusca, Bivalvia, Veneroida, Veneridae, Paphia|JQ277803|China
Paphia gallus|Mollusca, Bivalvia, Veneroida, Veneridae, Paphia|JQ277805|China
Paphia gallus|Mollusca, Bivalvia, Veneroida, Veneridae, Paphia|JQ277807|China
Paphia gallus|Mollusca, Bivalvia, Veneroida, Veneridae, Paphia|JQ277810|China
Paphia gallus|Mollusca, Bivalvia, Veneroida, Veneridae, Paphia|HQ703235|Beihai, Guangxi province, China
Paphia gallus|Mollusca, Bivalvia, Veneroida, Veneridae, Paphia|HQ703233|Baimajing, Hainan province, China
Paphia gallus|Mollusca, Bivalvia, Veneroida, Veneridae, Paphia|GQ855249|Danzhou, Hainan province, China
Paphia gallus|Mollusca, Bivalvia, Veneroida, Veneridae, Paphia|JQ277814|China
Paphia gallus|Mollusca, Bivalvia, Veneroida, Veneridae, Paphia|JQ277812|China
Paphia gallus|Mollusca, Bivalvia, Veneroida, Veneridae, Paphia|GQ855250|Beihai, Guangxi province, China
Paphia gallus|Mollusca, Bivalvia, Veneroida, Veneridae, Paphia|HQ703234|Beihai, Guangxi province, China
Paphia gallus|Mollusca, Bivalvia, Veneroida, Veneridae, Paphia|JQ277804|China
Paphia gallus|Mollusca, Bivalvia, Veneroida, Veneridae, Paphia|GQ855248|Beihai, Guangxi province, China
Paphia gallus|Mollusca, Bivalvia, Veneroida, Veneridae, Paphia|JQ277806|China
Paphia gallus|Mollusca, Bivalvia, Veneroida, Veneridae, Paphia|JQ277808|China
Paphia gallus|Mollusca, Bivalvia, Veneroida, Veneridae, Paphia|JQ277811|China
Paphia gallus|Mollusca, Bivalvia, Veneroida, Veneridae, Paphia|JQ277813|China
Paphia gallus|Mollusca, Bivalvia, Veneroida, Veneridae, Paphia|JQ277809|China
Paphia gallus|Mollusca, Bivalvia, Veneroida, Veneridae, Paphia|HM124597|China
Paphia sinuosa|Mollusca, Bivalvia, Veneroida, Veneridae, Paphia|JQ277815|China
Paphia sinuosa|Mollusca, Bivalvia, Veneroida, Veneridae, Paphia|HQ703231|Pingtan, Fujian province, China
Geloina expansa|Mollusca, Bivalvia, Veneroida, Corbiculidae, Geloina|AB498812|Okinawa, Iriomote Island, Okinawa, Japan
Geloina erosa|Mollusca, Bivalvia, Veneroida, Corbiculidae, Geloina|AB076927|Okinawa, Iriomote, Okinawa, Japan
Corbicula sandai|Mollusca, Bivalvia, Veneroida, Corbiculidae, Corbicula|AB498811|Shiga, Lake Biwa, Japan
Corbicula sandai|Mollusca, Bivalvia, Veneroida, Corbiculidae, Corbicula|AB845590|Shiga, Hikone, Lake Biwa, Japan,
Corbicula sandai|Mollusca, Bivalvia, Veneroida, Corbiculidae, Corbicula|KC211278|Japan
Corbicula sandai|Mollusca, Bivalvia, Veneroida, Corbiculidae, Corbicula|KC211280|Japan
Corbicula sandai|Mollusca, Bivalvia, Veneroida, Corbiculidae, Corbicula|KC211279|Japan
Corbicula sandai|Mollusca, Bivalvia, Veneroida, Corbiculidae, Corbicula|KC211277|Japan
Corbicula leana|Mollusca, Bivalvia, Veneroida, Corbiculidae, Corbicula|ollusca|AB845591|Hyogo, minami-awaji, Hatsuo Riv, Japan
Corbicula leana|Mollusca, Bivalvia, Veneroida, Corbiculidae, Corbicula|AB498810|Fukuoka, Chikugo River, Japan

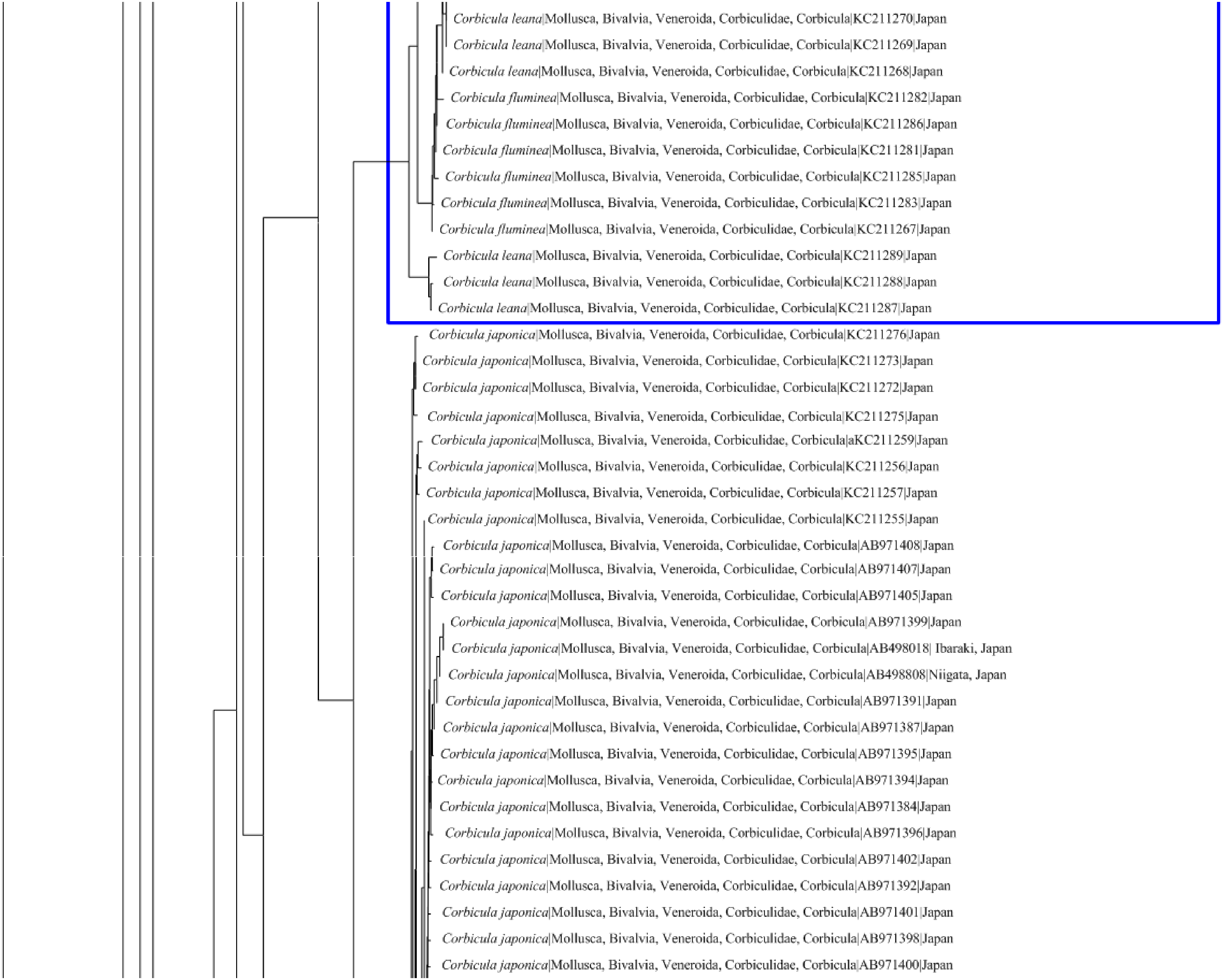
Corbicula leana|Mollusca, Bivalvia, Veneroida, Corbiculidae, Corbicula|KC211270|Japan
Corbicula leana|Mollusca, Bivalvia, Veneroida, Corbiculidae, Corbicula|KC211269|Japan
Corbicula leana|Mollusca, Bivalvia, Veneroida, Corbiculidae, Corbicula|KC211268|Japan
Corbicula fluminea|Mollusca, Bivalvia, Veneroida, Corbiculidae, Corbicula|KC211282|Japan
Corbicula fluminea|Mollusca, Bivalvia, Veneroida, Corbiculidae, Corbicula|KC211286|Japan
Corbicula fluminea|Mollusca, Bivalvia, Veneroida, Corbiculidae, Corbicula|KC211281|Japan
Corbicula fluminea|Mollusca, Bivalvia, Veneroida, Corbiculidae, Corbicula|KC211285|Japan
Corbicula fluminea|Mollusca, Bivalvia, Veneroida, Corbiculidae, Corbicula|KC211283|Japan
Corbicula fluminea|Mollusca, Bivalvia, Veneroida, Corbiculidae, Corbicula|KC211267|Japan
Corbicula leana|Mollusca, Bivalvia, Veneroida, Corbiculidae, Corbicula|KC211289|Japan
Corbicula leana|Mollusca, Bivalvia, Veneroida, Corbiculidae, Corbicula|KC211288|Japan
Corbicula leana|Mollusca, Bivalvia, Veneroida, Corbiculidae, Corbicula|KC211287|Japan
Corbicula japonica|Mollusca, Bivalvia, Veneroida, Corbiculidae, Corbicula|KC211276|Japan
Corbicula japonica|Mollusca, Bivalvia, Veneroida, Corbiculidae, Corbicula|KC211273|Japan
Corbicula japonica|Mollusca, Bivalvia, Veneroida, Corbiculidae, Corbicula|KC211272|Japan
Corbicula japonica|Mollusca, Bivalvia, Veneroida, Corbiculidae, Corbicula|KC211275|Japan
Corbicula japonica|Mollusca, Bivalvia, Veneroida, Corbiculidae, Corbicula|aKC211259|Japan
Corbicula japonica|Mollusca, Bivalvia, Veneroida, Corbiculidae, Corbicula|KC211256|Japan
Corbicula japonica|Mollusca, Bivalvia, Veneroida, Corbiculidae, Corbicula|KC211257|Japan
Corbicula japonica|Mollusca, Bivalvia, Veneroida, Corbiculidae, Corbicula|KC211255|Japan
Corbicula japonica|Mollusca, Bivalvia, Veneroida, Corbiculidae, Corbicula|AB971408|Japan
Corbicula japonica|Mollusca, Bivalvia, Veneroida, Corbiculidae, Corbicula|AB971407|Japan
Corbicula japonica|Mollusca, Bivalvia, Veneroida, Corbiculidae, Corbicula|AB971405|Japan
Corbicula japonica|Mollusca, Bivalvia, Veneroida, Corbiculidae, Corbicula|AB971399|Japan
Corbicula japonica|Mollusca, Bivalvia, Veneroida, Corbiculidae, Corbicula|AB498018| Ibaraki, Japan
Corbicula japonica|Mollusca, Bivalvia, Veneroida, Corbiculidae, Corbicula|AB498808|Niigata, Japan
Corbicula japonica|Mollusca, Bivalvia, Veneroida, Corbiculidae, Corbicula|AB971391|Japan
Corbicula japonica|Mollusca, Bivalvia, Veneroida, Corbiculidae, Corbicula|AB971387|Japan
Corbicula japonica|Mollusca, Bivalvia, Veneroida, Corbiculidae, Corbicula|AB971395|Japan
Corbicula japonica|Mollusca, Bivalvia, Veneroida, Corbiculidae, Corbicula|AB971394|Japan
Corbicula japonica|Mollusca, Bivalvia, Veneroida, Corbiculidae, Corbicula|AB971384|Japan
Corbicula japonica|Mollusca, Bivalvia, Veneroida, Corbiculidae, Corbicula|AB971396|Japan
Corbicula japonica|Mollusca, Bivalvia, Veneroida, Corbiculidae, Corbicula|AB971402|Japan
Corbicula japonica|Mollusca, Bivalvia, Veneroida, Corbiculidae, Corbicula|AB971392|Japan
Corbicula japonica|Mollusca, Bivalvia, Veneroida, Corbiculidae, Corbicula|AB971401|Japan
Corbicula japonica|Mollusca, Bivalvia, Veneroida, Corbiculidae, Corbicula|AB971398|Japan
Corbicula japonica|Mollusca, Bivalvia, Veneroida, Corbiculidae, Corbicula|AB971400|Japan

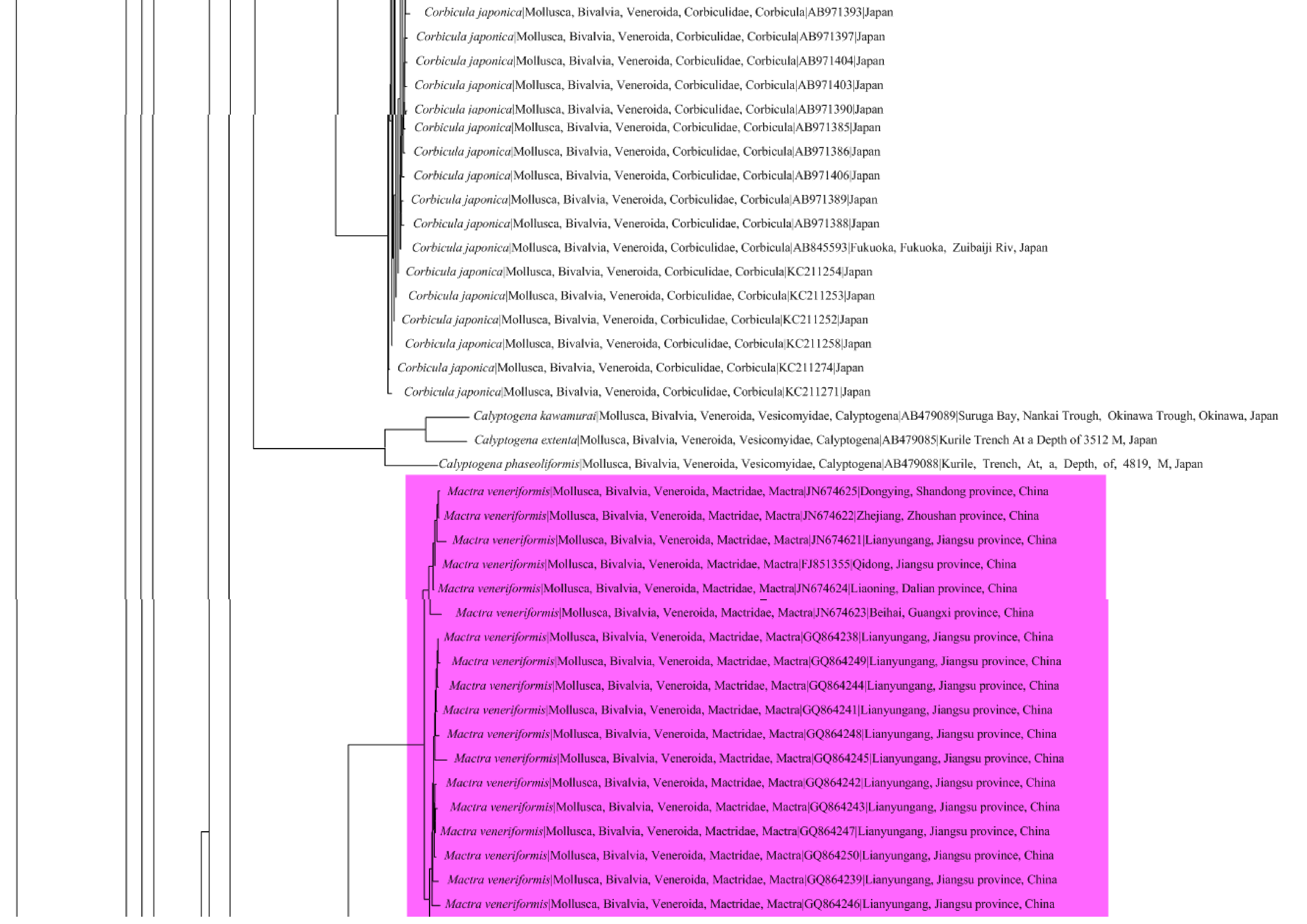
Corbicula japonica|Mollusca, Bivalvia, Veneroida, Corbiculidae, Corbicula|AB971393|Japan
Corbicula japonica|Mollusca, Bivalvia, Veneroida, Corbiculidae, Corbicula|AB971397|Japan
Corbicula japonica|Mollusca, Bivalvia, Veneroida, Corbiculidae, Corbicula|AB971404|Japan
Corbicula japonica|Mollusca, Bivalvia, Veneroida, Corbiculidae, Corbicula|AB971403|Japan
Corbicula japonica|Mollusca, Bivalvia, Veneroida, Corbiculidae, Corbicula|AB971390|Japan
Corbicula japonica|Mollusca, Bivalvia, Veneroida, Corbiculidae, Corbicula|AB971385|Japan
Corbicula japonica|Mollusca, Bivalvia, Veneroida, Corbiculidae, Corbicula|AB971386|Japan
Corbicula japonica|Mollusca, Bivalvia, Veneroida, Corbiculidae, Corbicula|AB971406|Japan
Corbicula japonica|Mollusca, Bivalvia, Veneroida, Corbiculidae, Corbicula|AB971389|Japan
Corbicula japonica|Mollusca, Bivalvia, Veneroida, Corbiculidae, Corbicula|AB971388|Japan
Corbicula japonica|Mollusca, Bivalvia, Veneroida, Corbiculidae, Corbicula|AB845593|Fukuoka, Fukuoka, Zuibaiji Riv, Japan
Corbicula japonica|Mollusca, Bivalvia, Veneroida, Corbiculidae, Corbicula|KC211254|Japan
Corbicula japonica|Mollusca, Bivalvia, Veneroida, Corbiculidae, Corbicula|KC211253|Japan
Corbicula japonica|Mollusca, Bivalvia, Veneroida, Corbiculidae, Corbicula|KC211252|Japan
Corbicula japonica|Mollusca, Bivalvia, Veneroida, Corbiculidae, Corbicula|KC211258|Japan
Corbicula japonica|Mollusca, Bivalvia, Veneroida, Corbiculidae, Corbicula|KC211274|Japan
Corbicula japonica|Mollusca, Bivalvia, Veneroida, Corbiculidae, Corbicula|KC211271|Japan
Calyptogena kawamurai|Mollusca, Bivalvia, Veneroida, Vesicomyidae, Calyptogena|AB479089|Suruga Bay, Nankai Trough, Okinawa Trough, Okinawa, Japan
Calyptogena extenta|Mollusca, Bivalvia, Veneroida, Vesicomyidae, Calyptogena|AB479085|Kurile Trench At a Depth of 3512 M, Japan
Calyptogena phaseoliformis|Mollusca, Bivalvia, Veneroida, Vesicomyidae, Calyptogena|AB479088|Kurile, Trench, At, a, Depth, of, 4819, M, Japan
Mactra veneriformis|Mollusca, Bivalvia, Veneroida, Mactridae, Mactra|JN674625|Dongying, Shandong province, China
Mactra veneriformis|Mollusca, Bivalvia, Veneroida, Mactridae, Mactra|JN674622|Zhejiang, Zhoushan province, China
Mactra veneriformis|Mollusca, Bivalvia, Veneroida, Mactridae, Mactra|JN674621|Lianyungang, Jiangsu province, China
Mactra veneriformis|Mollusca, Bivalvia, Veneroida, Mactridae, Mactra|FJ851355|Qidong, Jiangsu province, China
Mactra veneriformis|Mollusca, Bivalvia, Veneroida, Mactridae, Mactra|JN674624|Liaoning, Dalian province, China
Mactra veneriformis|Mollusca, Bivalvia, Veneroida, Mactridae, Mactra|JN674623|Beihai, Guangxi province, China
Mactra veneriformis|Mollusca, Bivalvia, Veneroida, Mactridae, Mactra|GQ864238|Lianyungang, Jiangsu province, China
Mactra veneriformis|Mollusca, Bivalvia, Veneroida, Mactridae, Mactra|GQ864249|Lianyungang, Jiangsu province, China
Mactra veneriformis|Mollusca, Bivalvia, Veneroida, Mactridae, Mactra|GQ864244|Lianyungang, Jiangsu province, China
Mactra veneriformis|Mollusca, Bivalvia, Veneroida, Mactridae, Mactra|GQ864241|Lianyungang, Jiangsu province, China
Mactra veneriformis|Mollusca, Bivalvia, Veneroida, Mactridae, Mactra|GQ864248|Lianyungang, Jiangsu province, China
Mactra veneriformis|Mollusca, Bivalvia, Veneroida, Mactridae, Mactra|GQ864245|Lianyungang, Jiangsu province, China
Mactra veneriformis|Mollusca, Bivalvia, Veneroida, Mactridae, Mactra|GQ864242|Lianyungang, Jiangsu province, China
Mactra veneriformis|Mollusca, Bivalvia, Veneroida, Mactridae, Mactra|GQ864243|Lianyungang, Jiangsu province, China
Mactra veneriformis|Mollusca, Bivalvia, Veneroida, Mactridae, Mactra|GQ864247|Lianyungang, Jiangsu province, China
Mactra veneriformis|Mollusca, Bivalvia, Veneroida, Mactridae, Mactra|GQ864250|Lianyungang, Jiangsu province, China
Mactra veneriformis|Mollusca, Bivalvia, Veneroida, Mactridae, Mactra|GQ864239|Lianyungang, Jiangsu province, China
Mactra veneriformis|Mollusca, Bivalvia, Veneroida, Mactridae, Mactra|GQ864246|Lianyungang, Jiangsu province, China

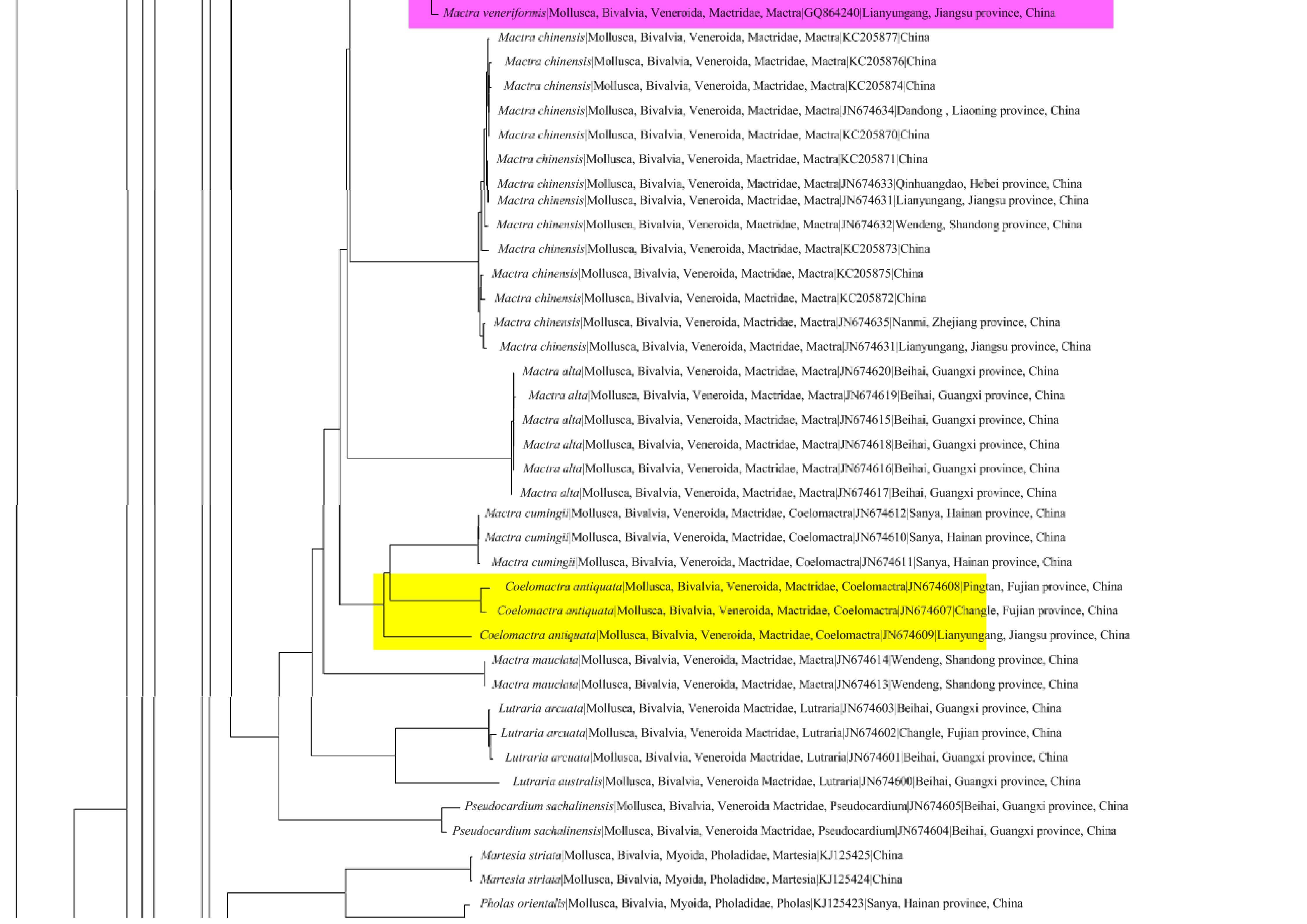
Mactra veneriformis|Mollusca, Bivalvia, Veneroida, Mactridae, Mactra|GQ864240|Lianyungang, Jiangsu province, China
Mactra chinensis|Mollusca, Bivalvia, Veneroida, Mactridae, Mactra|KC205877|China
Mactra chinensis|Mollusca, Bivalvia, Veneroida, Mactridae, Mactra|KC205876|China
Mactra chinensis|Mollusca, Bivalvia, Veneroida, Mactridae, Mactra|KC205874|China
Mactra chinensis|Mollusca, Bivalvia, Veneroida, Mactridae, Mactra|JN674634|Dandong , Liaoning province, China
Mactra chinensis|Mollusca, Bivalvia, Veneroida, Mactridae, Mactra|KC205870|China
Mactra chinensis|Mollusca, Bivalvia, Veneroida, Mactridae, Mactra|KC205871|China
Mactra chinensis|Mollusca, Bivalvia, Veneroida, Mactridae, Mactra|JN674633|Qinhuangdao, Hebei province, China
Mactra chinensis|Mollusca, Bivalvia, Veneroida, Mactridae, Mactra|JN674631|Lianyungang, Jiangsu province, China
Mactra chinensis|Mollusca, Bivalvia, Veneroida, Mactridae, Mactra|JN674632|Wendeng, Shandong province, China
Mactra chinensis|Mollusca, Bivalvia, Veneroida, Mactridae, Mactra|KC205873|China
Mactra chinensis|Mollusca, Bivalvia, Veneroida, Mactridae, Mactra|KC205875|China
Mactra chinensis|Mollusca, Bivalvia, Veneroida, Mactridae, Mactra|KC205872|China
Mactra chinensis|Mollusca, Bivalvia, Veneroida, Mactridae, Mactra|JN674635|Nanmi, Zhejiang province, China
Mactra chinensis|Mollusca, Bivalvia, Veneroida, Mactridae, Mactra|JN674631|Lianyungang, Jiangsu province, China
Mactra alta|Mollusca, Bivalvia, Veneroida, Mactridae, Mactra|JN674620|Beihai, Guangxi province, China
Mactra alta|Mollusca, Bivalvia, Veneroida, Mactridae, Mactra|JN674619|Beihai, Guangxi province, China
Mactra alta|Mollusca, Bivalvia, Veneroida, Mactridae, Mactra|JN674615|Beihai, Guangxi province, China
Mactra alta|Mollusca, Bivalvia, Veneroida, Mactridae, Mactra|JN674618|Beihai, Guangxi province, China
Mactra alta|Mollusca, Bivalvia, Veneroida, Mactridae, Mactra|JN674616|Beihai, Guangxi province, China
Mactra alta|Mollusca, Bivalvia, Veneroida, Mactridae, Mactra|JN674617|Beihai, Guangxi province, China
Mactra cumingii|Mollusca, Bivalvia, Veneroida, Mactridae, Coelomactra|JN674612|Sanya, Hainan province, China
Mactra cumingii|Mollusca, Bivalvia, Veneroida, Mactridae, Coelomactra|JN674610|Sanya, Hainan province, China
Mactra cumingii|Mollusca, Bivalvia, Veneroida, Mactridae, Coelomactra|JN674611|Sanya, Hainan province, China
Coelomactra antiquata|Mollusca, Bivalvia, Veneroida, Mactridae, Coelomactra|JN674608|Pingtan, Fujian province, China
Coelomactra antiquata|Mollusca, Bivalvia, Veneroida, Mactridae, Coelomactra|JN674607|Changle, Fujian province, China
Coelomactra antiquata|Mollusca, Bivalvia, Veneroida, Mactridae, Coelomactra|JN674609|Lianyungang, Jiangsu province, China
Mactra mauclata|Mollusca, Bivalvia, Veneroida, Mactridae, Mactra|JN674614|Wendeng, Shandong province, China
Mactra mauclata|Mollusca, Bivalvia, Veneroida, Mactridae, Mactra|JN674613|Wendeng, Shandong province, China
Lutraria arcuata|Mollusca, Bivalvia, Veneroida Mactridae, Lutraria|JN674603|Beihai, Guangxi province, China
Lutraria arcuata|Mollusca, Bivalvia, Veneroida Mactridae, Lutraria|JN674602|Changle, Fujian province, China
Lutraria arcuata|Mollusca, Bivalvia, Veneroida Mactridae, Lutraria|JN674601|Beihai, Guangxi province, China
Lutraria australis|Mollusca, Bivalvia, Veneroida Mactridae, Lutraria|JN674600|Beihai, Guangxi province, China
Pseudocardium sachalinensis|Mollusca, Bivalvia, Veneroida Mactridae, Pseudocardium|JN674605|Beihai, Guangxi province, China
Pseudocardium sachalinensis|Mollusca, Bivalvia, Veneroida Mactridae, Pseudocardium|JN674604|Beihai, Guangxi province, China
Martesia striata|Mollusca, Bivalvia, Myoida, Pholadidae, Martesia|KJ125425|China
Martesia striata|Mollusca, Bivalvia, Myoida, Pholadidae, Martesia|KJ125424|China
Pholas orientalis|Mollusca, Bivalvia, Myoida, Pholadidae, Pholas|KJ125423|Sanya, Hainan province, China

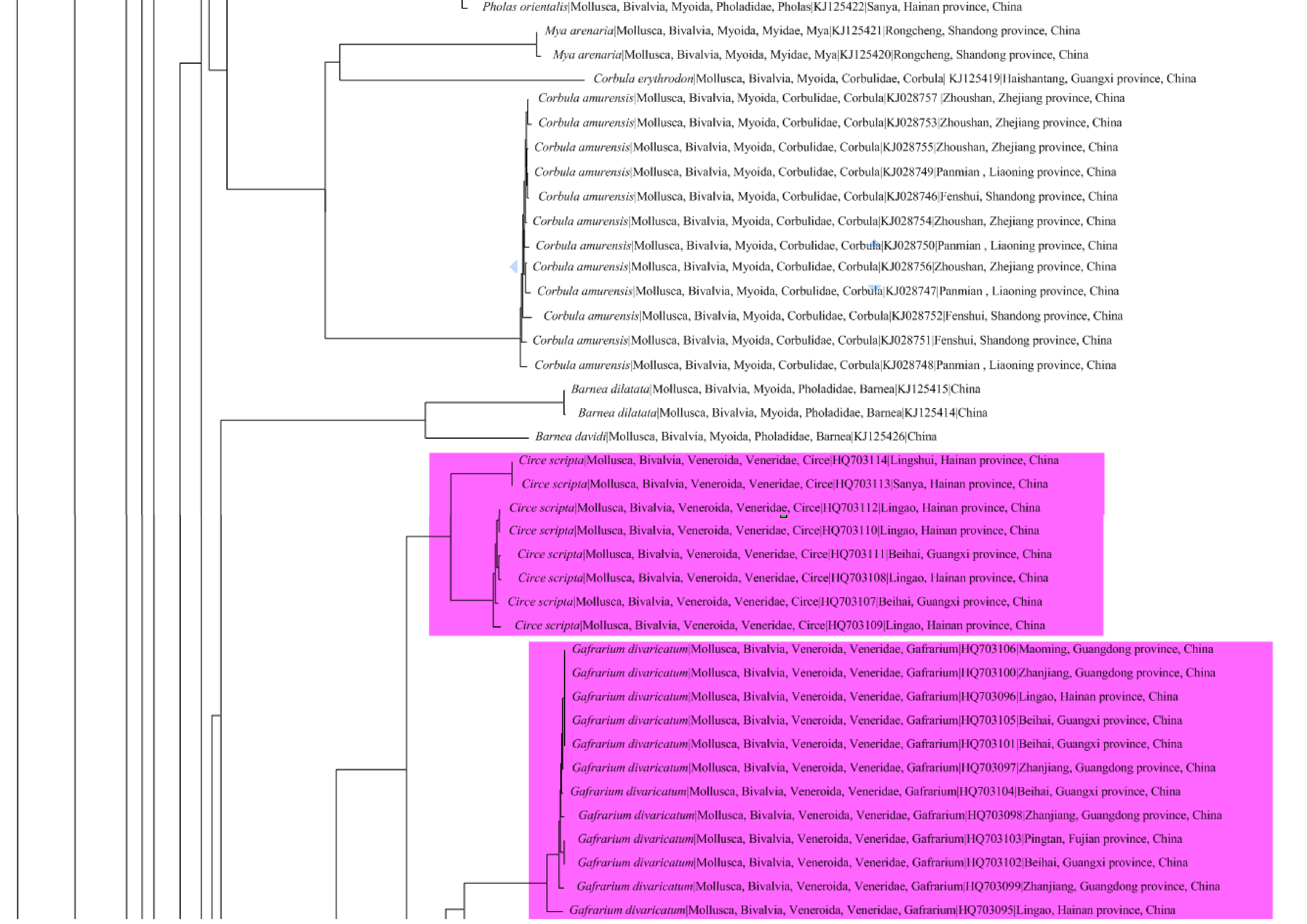
Pholas orientalis|Mollusca, Bivalvia, Myoida, Pholadidae, Pholas|KJ125422|Sanya, Hainan province, China
Mya arenaria|Mollusca, Bivalvia, Myoida, Myidae, Mya|KJ125421|Rongcheng, Shandong province, China
Mya arenaria|Mollusca, Bivalvia, Myoida, Myidae, Mya|KJ125420|Rongcheng, Shandong province, China
Corbula erythrodon|Mollusca, Bivalvia, Myoida, Corbulidae, Corbula| KJ125419|Haishantang, Guangxi province, China
Corbula amurensis|Mollusca, Bivalvia, Myoida, Corbulidae, Corbula|KJ028757 |Zhoushan, Zhejiang province, China
Corbula amurensis|Mollusca, Bivalvia, Myoida, Corbulidae, Corbula|KJ028753|Zhoushan, Zhejiang province, China
Corbula amurensis|Mollusca, Bivalvia, Myoida, Corbulidae, Corbula|KJ028755|Zhoushan, Zhejiang province, China
Corbula amurensis|Mollusca, Bivalvia, Myoida, Corbulidae, Corbula|KJ028749|Panmian , Liaoning province, China
Corbula amurensis|Mollusca, Bivalvia, Myoida, Corbulidae, Corbula|KJ028746|Fenshui, Shandong province, China
Corbula amurensis|Mollusca, Bivalvia, Myoida, Corbulidae, Corbula|KJ028754|Zhoushan, Zhejiang province, China
Corbula amurensis|Mollusca, Bivalvia, Myoida, Corbulidae, Corbula|KJ028750|Panmian , Liaoning province, China
Corbula amurensis|Mollusca, Bivalvia, Myoida, Corbulidae, Corbula|KJ028756|Zhoushan, Zhejiang province, China
Corbula amurensis|Mollusca, Bivalvia, Myoida, Corbulidae, Corbula|KJ028747|Panmian , Liaoning province, China
Corbula amurensis|Mollusca, Bivalvia, Myoida, Corbulidae, Corbula|KJ028752|Fenshui, Shandong province, China
Corbula amurensis|Mollusca, Bivalvia, Myoida, Corbulidae, Corbula|KJ028751|Fenshui, Shandong province, China
Corbula amurensis|Mollusca, Bivalvia, Myoida, Corbulidae, Corbula|KJ028748|Panmian , Liaoning province, China
Barnea dilatata|Mollusca, Bivalvia, Myoida, Pholadidae, Barnea|KJ125415|China
Barnea dilatata|Mollusca, Bivalvia, Myoida, Pholadidae, Barnea|KJ125414|China
Barnea davidi|Mollusca, Bivalvia, Myoida, Pholadidae, Barnea|KJ125426|China
Circe scripta|Mollusca, Bivalvia, Veneroida, Veneridae, Circe|HQ703114|Lingshui, Hainan province, China
Circe scripta|Mollusca, Bivalvia, Veneroida, Veneridae, Circe|HQ703113|Sanya, Hainan province, China
Circe scripta|Mollusca, Bivalvia, Veneroida, Veneridae, Circe|HQ703112|Lingao, Hainan province, China
Circe scripta|Mollusca, Bivalvia, Veneroida, Veneridae, Circe|HQ703110|Lingao, Hainan province, China
Circe scripta|Mollusca, Bivalvia, Veneroida, Veneridae, Circe|HQ703111|Beihai, Guangxi province, China
Circe scripta|Mollusca, Bivalvia, Veneroida, Veneridae, Circe|HQ703108|Lingao, Hainan province, China
Circe scripta|Mollusca, Bivalvia, Veneroida, Veneridae, Circe|HQ703107|Beihai, Guangxi province, China
Circe scripta|Mollusca, Bivalvia, Veneroida, Veneridae, Circe|HQ703109|Lingao, Hainan province, China
Gafrarium divaricatum|Mollusca, Bivalvia, Veneroida, Veneridae, Gafrarium|HQ703106|Maoming, Guangdong province, China
Gafrarium divaricatum|Mollusca, Bivalvia, Veneroida, Veneridae, Gafrarium|HQ703100|Zhanjiang, Guangdong province, China
Gafrarium divaricatum|Mollusca, Bivalvia, Veneroida, Veneridae, Gafrarium|HQ703096|Lingao, Hainan province, China
Gafrarium divaricatum|Mollusca, Bivalvia, Veneroida, Veneridae, Gafrarium|HQ703105|Beihai, Guangxi province, China
Gafrarium divaricatum|Mollusca, Bivalvia, Veneroida, Veneridae, Gafrarium|HQ703101|Beihai, Guangxi province, China
Gafrarium divaricatum|Mollusca, Bivalvia, Veneroida, Veneridae, Gafrarium|HQ703097|Zhanjiang, Guangdong province, China
Gafrarium divaricatum|Mollusca, Bivalvia, Veneroida, Veneridae, Gafrarium|HQ703104|Beihai, Guangxi province, China
Gafrarium divaricatum|Mollusca, Bivalvia, Veneroida, Veneridae, Gafrarium|HQ703098|Zhanjiang, Guangdong province, China
Gafrarium divaricatum|Mollusca, Bivalvia, Veneroida, Veneridae, Gafrarium|HQ703103|Pingtan, Fujian province, China
Gafrarium divaricatum|Mollusca, Bivalvia, Veneroida, Veneridae, Gafrarium|HQ703102|Beihai, Guangxi province, China
Gafrarium divaricatum|Mollusca, Bivalvia, Veneroida, Veneridae, Gafrarium|HQ703099|Zhanjiang, Guangdong province, China
Gafrarium divaricatum|Mollusca, Bivalvia, Veneroida, Veneridae, Gafrarium|HQ703095|Lingao, Hainan province, China

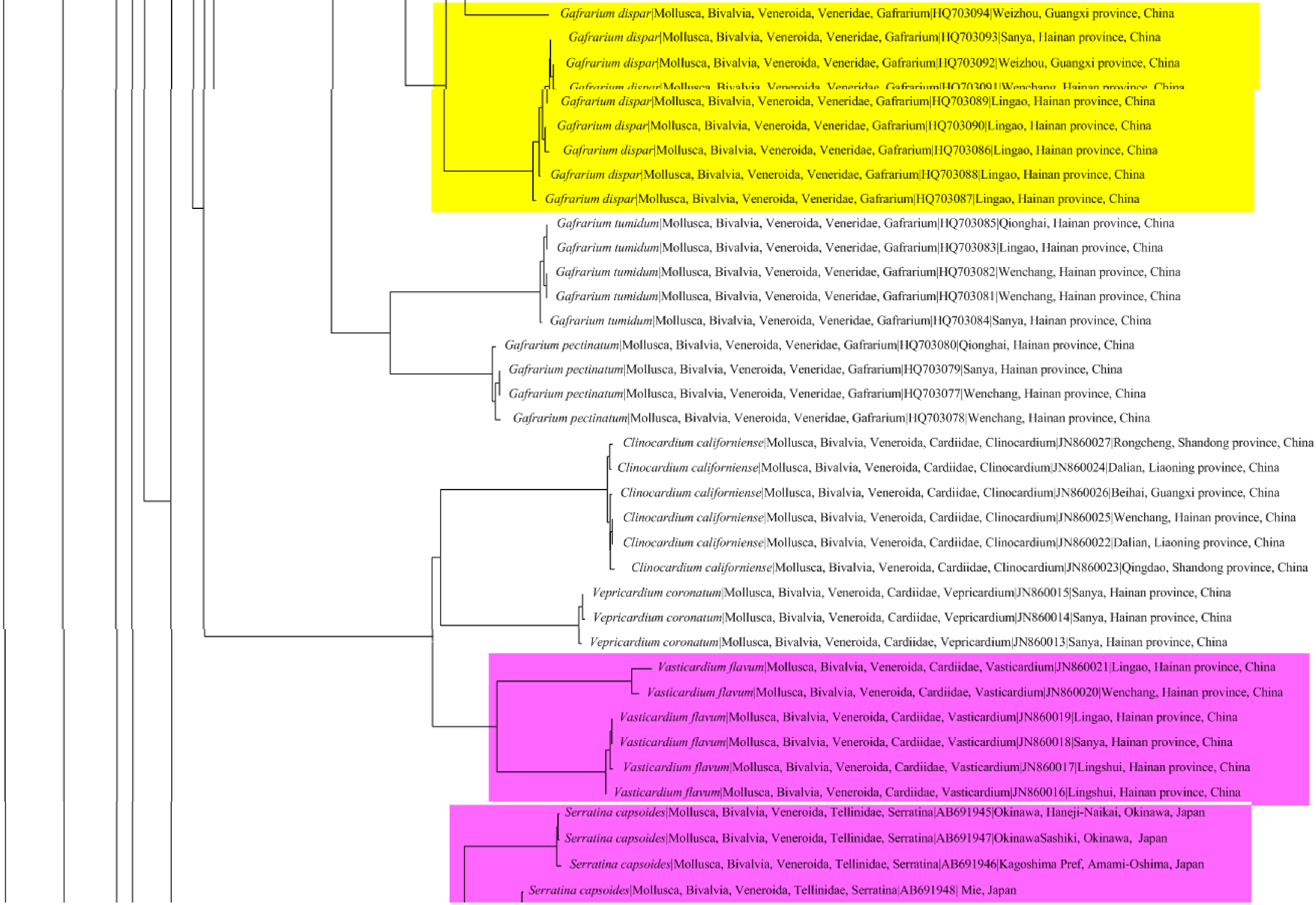

Gafrarium dispar|Mollusca, Bivalvia, Veneroida, Veneridae, Gafrarium|HQ703094|Weizhou, Guangxi province, China
Gafrarium dispar|Mollusca, Bivalvia, Veneroida, Veneridae, Gafrarium|HQ703093|Sanya, Hainan province, China
Gafrarium dispar|Mollusca, Bivalvia, Veneroida, Veneridae, Gafrarium|HQ703092|Weizhou, Guangxi province, China
Gafrarium dispar|Mollusca, Bivalvia, Veneroida, Veneridae, Gafrarium|HQ703091|Wenchang, Hainan province, China
Gafrarium dispar|Mollusca, Bivalvia, Veneroida, Veneridae, Gafrarium|HQ703089|Lingao, Hainan province, China
Gafrarium dispar|Mollusca, Bivalvia, Veneroida, Veneridae, Gafrarium|HQ703090|Lingao, Hainan province, China
Gafrarium dispar|Mollusca, Bivalvia, Veneroida, Veneridae, Gafrarium|HQ703086|Lingao, Hainan province, China
Gafrarium dispar|Mollusca, Bivalvia, Veneroida, Veneridae, Gafrarium|HQ703088|Lingao, Hainan province, China
Gafrarium dispar|Mollusca, Bivalvia, Veneroida, Veneridae, Gafrarium|HQ703087|Lingao, Hainan province, China
Gafrarium tumidum|Mollusca, Bivalvia, Veneroida, Veneridae, Gafrarium|HQ703085|Qionghai, Hainan province, China
Gafrarium tumidum|Mollusca, Bivalvia, Veneroida, Veneridae, Gafrarium|HQ703083|Lingao, Hainan province, China
Gafrarium tumidum|Mollusca, Bivalvia, Veneroida, Veneridae, Gafrarium|HQ703082|Wenchang, Hainan province, China
Gafrarium tumidum|Mollusca, Bivalvia, Veneroida, Veneridae, Gafrarium|HQ703081|Wenchang, Hainan province, China
Gafrarium tumidum|Mollusca, Bivalvia, Veneroida, Veneridae, Gafrarium|HQ703084|Sanya, Hainan province, China
Gafrarium pectinatum|Mollusca, Bivalvia, Veneroida, Veneridae, Gafrarium|HQ703080|Qionghai, Hainan province, China
Gafrarium pectinatum|Mollusca, Bivalvia, Veneroida, Veneridae, Gafrarium|HQ703079|Sanya, Hainan province, China
Gafrarium pectinatum|Mollusca, Bivalvia, Veneroida, Veneridae, Gafrarium|HQ703077|Wenchang, Hainan province, China
Gafrarium pectinatum|Mollusca, Bivalvia, Veneroida, Veneridae, Gafrarium|HQ703078|Wenchang, Hainan province, China
Clinocardium californiense|Mollusca, Bivalvia, Veneroida, Cardiidae, Clinocardium|JN860027|Rongcheng, Shandong province, China
Clinocardium californiense|Mollusca, Bivalvia, Veneroida, Cardiidae, Clinocardium|JN860024|Dalian, Liaoning province, China
Clinocardium californiense|Mollusca, Bivalvia, Veneroida, Cardiidae, Clinocardium|JN860026|Beihai, Guangxi province, China
Clinocardium californiense|Mollusca, Bivalvia, Veneroida, Cardiidae, Clinocardium|JN860025|Wenchang, Hainan province, China
Clinocardium californiense|Mollusca, Bivalvia, Veneroida, Cardiidae, Clinocardium|JN860022|Dalian, Liaoning province, China
Clinocardium californiense|Mollusca, Bivalvia, Veneroida, Cardiidae, Clinocardium|JN860023|Qingdao, Shandong province, China
Vepricardium coronatum|Mollusca, Bivalvia, Veneroida, Cardiidae, Vepricardium|JN860015|Sanya, Hainan province, China
Vepricardium coronatum|Mollusca, Bivalvia, Veneroida, Cardiidae, Vepricardium|JN860014|Sanya, Hainan province, China
Vepricardium coronatum|Mollusca, Bivalvia, Veneroida, Cardiidae, Vepricardium|JN860013|Sanya, Hainan province, China
Vasticardium flavum|Mollusca, Bivalvia, Veneroida, Cardiidae, Vasticardium|JN860021|Lingao, Hainan province, China
Vasticardium flavum|Mollusca, Bivalvia, Veneroida, Cardiidae, Vasticardium|JN860020|Wenchang, Hainan province, China
Vasticardium flavum|Mollusca, Bivalvia, Veneroida, Cardiidae, Vasticardium|JN860019|Lingao, Hainan province, China
Vasticardium flavum|Mollusca, Bivalvia, Veneroida, Cardiidae, Vasticardium|JN860018|Sanya, Hainan province, China
Vasticardium flavum|Mollusca, Bivalvia, Veneroida, Cardiidae, Vasticardium|JN860017|Lingshui, Hainan province, China
Vasticardium flavum|Mollusca, Bivalvia, Veneroida, Cardiidae, Vasticardium|JN860016|Lingshui, Hainan province, China
Serratina capsoides|Mollusca, Bivalvia, Veneroida, Tellinidae, Serratina|AB691945|Okinawa, Haneji-Naikai, Okinawa, Japan
Serratina capsoides|Mollusca, Bivalvia, Veneroida, Tellinidae, Serratina|AB691947|OkinawaSashiki, Okinawa, Japan
Serratina capsoides|Mollusca, Bivalvia, Veneroida, Tellinidae, Serratina|AB691946|Kagoshima Pref, Amami-Oshima, Japan
Serratina capsoides|Mollusca, Bivalvia, Veneroida, Tellinidae, Serratina|AB691948| Mie, Japan

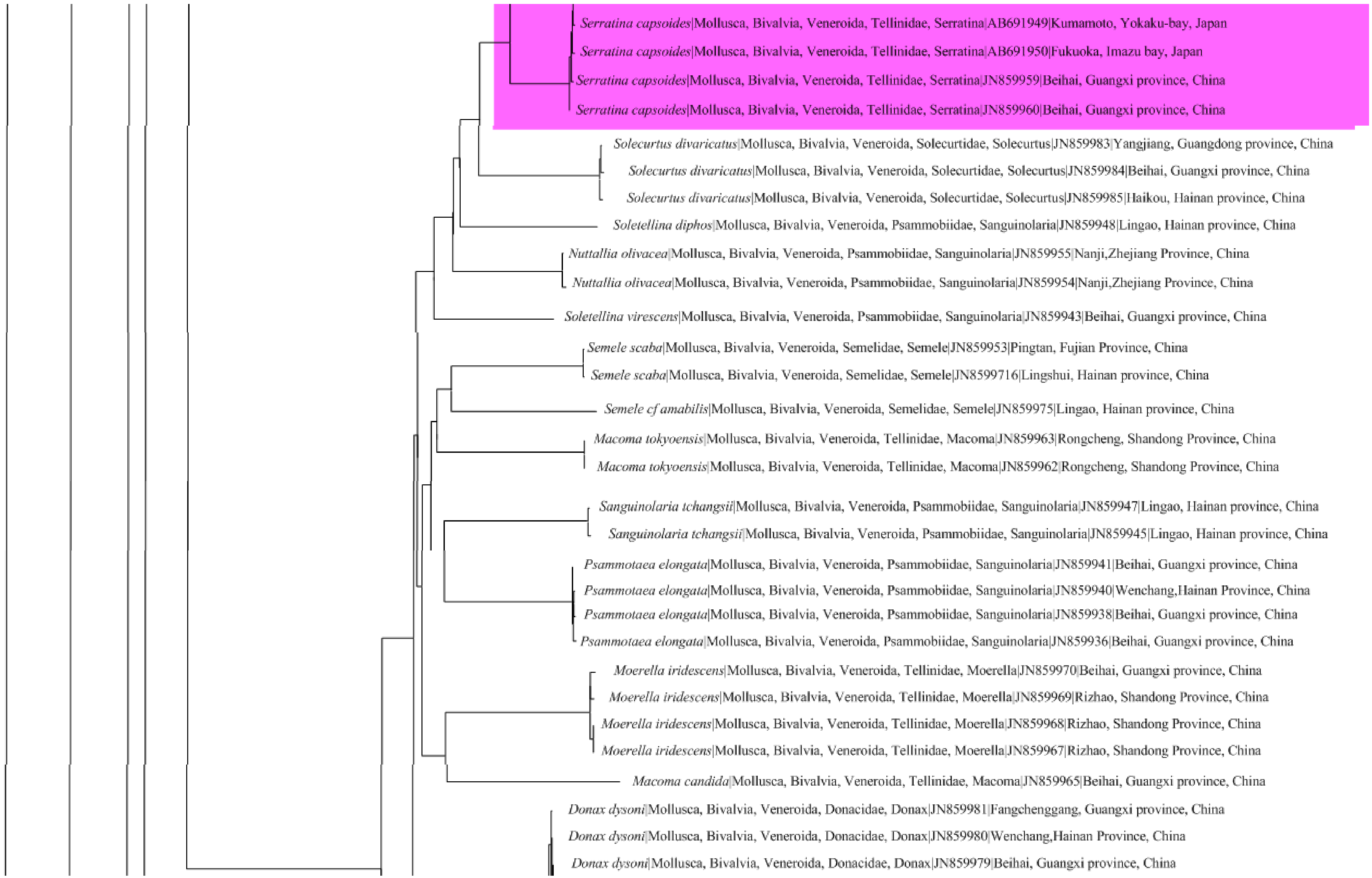
Serratina capsoides|Mollusca, Bivalvia, Veneroida, Tellinidae, Serratina|AB691949|Kumamoto, Yokaku-bay, Japan
Serratina capsoides|Mollusca, Bivalvia, Veneroida, Tellinidae, Serratina|AB691950|Fukuoka, Imazu bay, Japan
Serratina capsoides|Mollusca, Bivalvia, Veneroida, Tellinidae, Serratina|JN859959|Beihai, Guangxi province, China
Serratina capsoides|Mollusca, Bivalvia, Veneroida, Tellinidae, Serratina|JN859960|Beihai, Guangxi province, China
Solecurtus divaricatus|Mollusca, Bivalvia, Veneroida, Solecurtidae, Solecurtus|JN859983|Yangjiang, Guangdong province, China
Solecurtus divaricatus|Mollusca, Bivalvia, Veneroida, Solecurtidae, Solecurtus|JN859984|Beihai, Guangxi province, China
Solecurtus divaricatus|Mollusca, Bivalvia, Veneroida, Solecurtidae, Solecurtus|JN859985|Haikou, Hainan province, China
Soletellina diphos|Mollusca, Bivalvia, Veneroida, Psammobiidae, Sanguinolaria|JN859948|Lingao, Hainan province, China
Nuttallia olivacea|Mollusca, Bivalvia, Veneroida, Psammobiidae, Sanguinolaria|JN859955|Nanji,Zhejiang Province, China
Nuttallia olivacea|Mollusca, Bivalvia, Veneroida, Psammobiidae, Sanguinolaria|JN859954|Nanji,Zhejiang Province, China
Soletellina virescens|Mollusca, Bivalvia, Veneroida, Psammobiidae, Sanguinolaria|JN859943|Beihai, Guangxi province, China
Semele scaba|Mollusca, Bivalvia, Veneroida, Semelidae, Semele|JN859953|Pingtan, Fujian Province, China
Semele scaba|Mollusca, Bivalvia, Veneroida, Semelidae, Semele|JN8599716|Lingshui, Hainan province, China
Semele cf amabilis|Mollusca, Bivalvia, Veneroida, Semelidae, Semele|JN859975|Lingao, Hainan province, China
Macoma tokyoensis|Mollusca, Bivalvia, Veneroida, Tellinidae, Macoma|JN859963|Rongcheng, Shandong Province, China
Macoma tokyoensis|Mollusca, Bivalvia, Veneroida, Tellinidae, Macoma|JN859962|Rongcheng, Shandong Province, China
Sanguinolaria tchangsii|Mollusca, Bivalvia, Veneroida, Psammobiidae, Sanguinolaria|JN859947|Lingao, Hainan province, China
Sanguinolaria tchangsii|Mollusca, Bivalvia, Veneroida, Psammobiidae, Sanguinolaria|JN859945|Lingao, Hainan province, China
Psammotaea elongata|Mollusca, Bivalvia, Veneroida, Psammobiidae, Sanguinolaria|JN859941|Beihai, Guangxi province, China
Psammotaea elongata|Mollusca, Bivalvia, Veneroida, Psammobiidae, Sanguinolaria|JN859940|Wenchang,Hainan Province, China
Psammotaea elongata|Mollusca, Bivalvia, Veneroida, Psammobiidae, Sanguinolaria|JN859938|Beihai, Guangxi province, China
Psammotaea elongata|Mollusca, Bivalvia, Veneroida, Psammobiidae, Sanguinolaria|JN859936|Beihai, Guangxi province, China
Moerella iridescens|Mollusca, Bivalvia, Veneroida, Tellinidae, Moerella|JN859970|Beihai, Guangxi province, China
Moerella iridescens|Mollusca, Bivalvia, Veneroida, Tellinidae, Moerella|JN859969|Rizhao, Shandong Province, China
Moerella iridescens|Mollusca, Bivalvia, Veneroida, Tellinidae, Moerella|JN859968|Rizhao, Shandong Province, China
Moerella iridescens|Mollusca, Bivalvia, Veneroida, Tellinidae, Moerella|JN859967|Rizhao, Shandong Province, China
Macoma candida|Mollusca, Bivalvia, Veneroida, Tellinidae, Macoma|JN859965|Beihai, Guangxi province, China
Donax dysoni|Mollusca, Bivalvia, Veneroida, Donacidae, Donax|JN859981|Fangchenggang, Guangxi province, China
Donax dysoni|Mollusca, Bivalvia, Veneroida, Donacidae, Donax|JN859980|Wenchang,Hainan Province, China
Donax dysoni|Mollusca, Bivalvia, Veneroida, Donacidae, Donax|JN859979|Beihai, Guangxi province, China

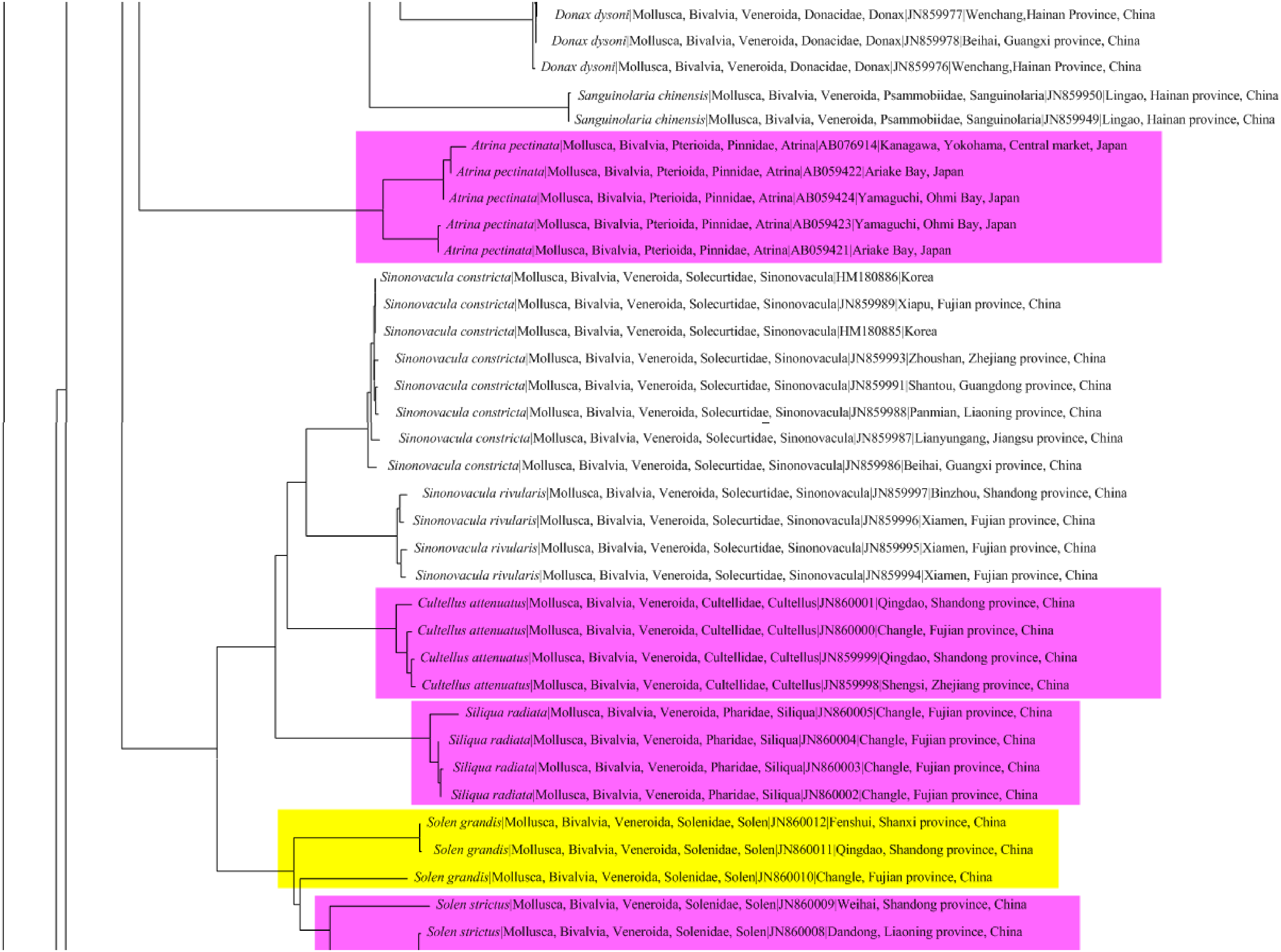
Donax dysoni|Mollusca, Bivalvia, Veneroida, Donacidae, Donax|JN859977|Wenchang,Hainan Province, China
Donax dysoni|Mollusca, Bivalvia, Veneroida, Donacidae, Donax|JN859978|Beihai, Guangxi province, China
Donax dysoni|Mollusca, Bivalvia, Veneroida, Donacidae, Donax|JN859976|Wenchang,Hainan Province, China
Sanguinolaria chinensis|Mollusca, Bivalvia, Veneroida, Psammobiidae, Sanguinolaria|JN859950|Lingao, Hainan province, China
Sanguinolaria chinensis|Mollusca, Bivalvia, Veneroida, Psammobiidae, Sanguinolaria|JN859949|Lingao, Hainan province, China
Atrina pectinata|Mollusca, Bivalvia, Pterioida, Pinnidae, Atrina|AB076914|Kanagawa, Yokohama, Central market, Japan
Atrina pectinata|Mollusca, Bivalvia, Pterioida, Pinnidae, Atrina|AB059422|Ariake Bay, Japan
Atrina pectinata|Mollusca, Bivalvia, Pterioida, Pinnidae, Atrina|AB059424|Yamaguchi, Ohmi Bay, Japan
Atrina pectinata|Mollusca, Bivalvia, Pterioida, Pinnidae, Atrina|AB059423|Yamaguchi, Ohmi Bay, Japan
Atrina pectinata|Mollusca, Bivalvia, Pterioida, Pinnidae, Atrina|AB059421|Ariake Bay, Japan
Sinonovacula constricta|Mollusca, Bivalvia, Veneroida, Solecurtidae, Sinonovacula|HM180886|Korea
Sinonovacula constricta|Mollusca, Bivalvia, Veneroida, Solecurtidae, Sinonovacula|JN859989|Xiapu, Fujian province, China
Sinonovacula constricta|Mollusca, Bivalvia, Veneroida, Solecurtidae, Sinonovacula|HM180885|Korea
Sinonovacula constricta|Mollusca, Bivalvia, Veneroida, Solecurtidae, Sinonovacula|JN859993|Zhoushan, Zhejiang province, China
Sinonovacula constricta|Mollusca, Bivalvia, Veneroida, Solecurtidae, Sinonovacula|JN859991|Shantou, Guangdong province, China
Sinonovacula constricta|Mollusca, Bivalvia, Veneroida, Solecurtidae, Sinonovacula|JN859988|Panmian, Liaoning province, China
Sinonovacula constricta|Mollusca, Bivalvia, Veneroida, Solecurtidae, Sinonovacula|JN859987|Lianyungang, Jiangsu province, China
Sinonovacula constricta|Mollusca, Bivalvia, Veneroida, Solecurtidae, Sinonovacula|JN859986|Beihai, Guangxi province, China
Sinonovacula rivularis|Mollusca, Bivalvia, Veneroida, Solecurtidae, Sinonovacula|JN859997|Binzhou, Shandong province, China
Sinonovacula rivularis|Mollusca, Bivalvia, Veneroida, Solecurtidae, Sinonovacula|JN859996|Xiamen, Fujian province, China
Sinonovacula rivularis|Mollusca, Bivalvia, Veneroida, Solecurtidae, Sinonovacula|JN859995|Xiamen, Fujian province, China
Sinonovacula rivularis|Mollusca, Bivalvia, Veneroida, Solecurtidae, Sinonovacula|JN859994|Xiamen, Fujian province, China
Cultellus attenuatus|Mollusca, Bivalvia, Veneroida, Cultellidae, Cultellus|JN860001|Qingdao, Shandong province, China
Cultellus attenuatus|Mollusca, Bivalvia, Veneroida, Cultellidae, Cultellus|JN860000|Changle, Fujian province, China
Cultellus attenuatus|Mollusca, Bivalvia, Veneroida, Cultellidae, Cultellus|JN859999|Qingdao, Shandong province, China
Cultellus attenuatus|Mollusca, Bivalvia, Veneroida, Cultellidae, Cultellus|JN859998|Shengsi, Zhejiang province, China
Siliqua radiata|Mollusca, Bivalvia, Veneroida, Pharidae, Siliqua|JN860005|Changle, Fujian province, China
Siliqua radiata|Mollusca, Bivalvia, Veneroida, Pharidae, Siliqua|JN860004|Changle, Fujian province, China
Siliqua radiata|Mollusca, Bivalvia, Veneroida, Pharidae, Siliqua|JN860003|Changle, Fujian province, China
Siliqua radiata|Mollusca, Bivalvia, Veneroida, Pharidae, Siliqua|JN860002|Changle, Fujian province, China
Solen grandis|Mollusca, Bivalvia, Veneroida, Solenidae, Solen|JN860012|Fenshui, Shanxi province, China
Solen grandis|Mollusca, Bivalvia, Veneroida, Solenidae, Solen|JN860011|Qingdao, Shandong province, China
Solen grandis|Mollusca, Bivalvia, Veneroida, Solenidae, Solen|JN860010|Changle, Fujian province, China
Solen strictus|Mollusca, Bivalvia, Veneroida, Solenidae, Solen|JN860009|Weihai, Shandong province, China
Solen strictus|Mollusca, Bivalvia, Veneroida, Solenidae, Solen|JN860008|Dandong, Liaoning province, China

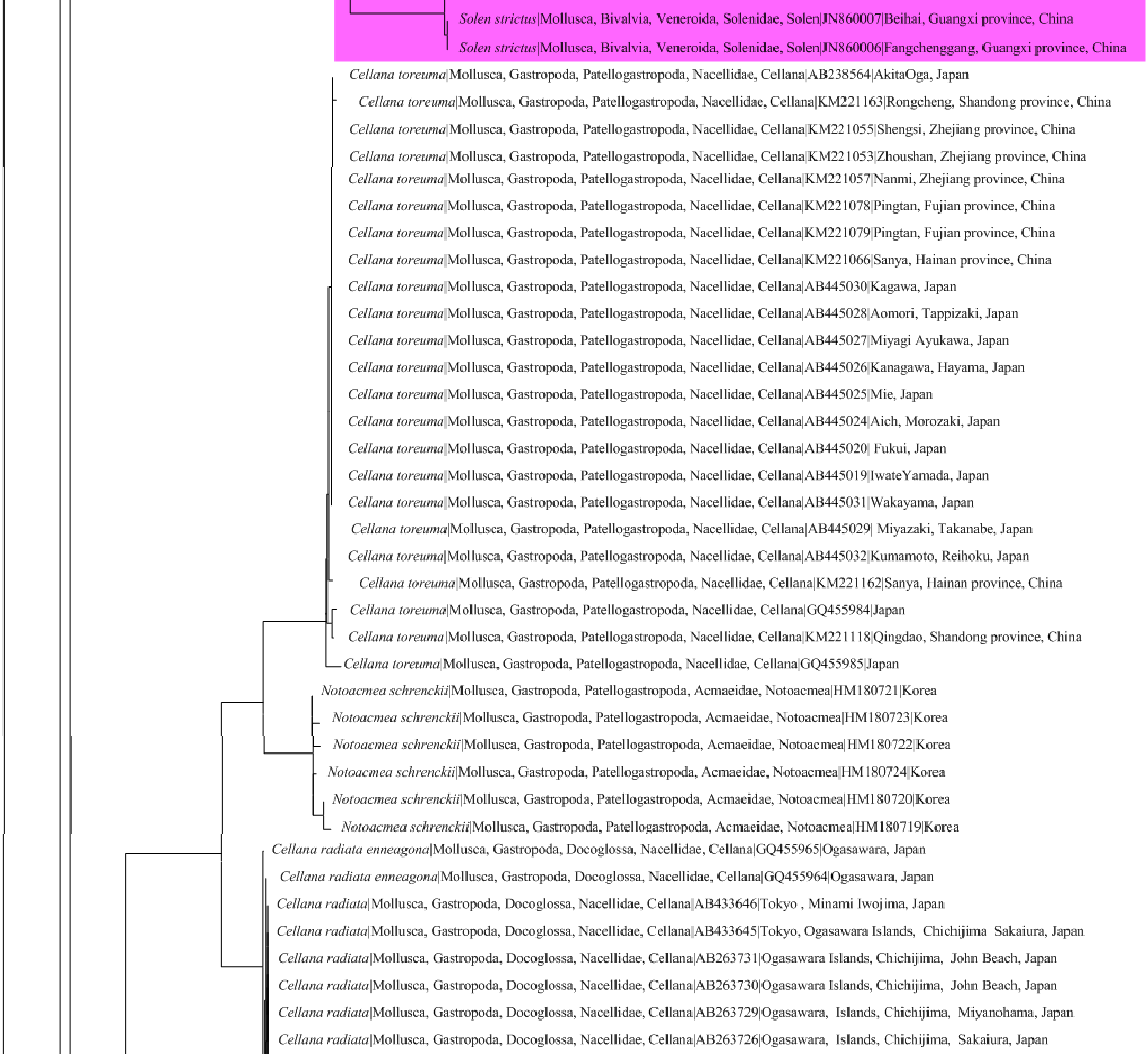
Solen strictus|Mollusca, Bivalvia, Veneroida, Solenidae, Solen|JN860007|Beihai, Guangxi province, China
Solen strictus|Mollusca, Bivalvia, Veneroida, Solenidae, Solen|JN860006|Fangchenggang, Guangxi province, China
Cellana toreuma|Mollusca, Gastropoda, Patellogastropoda, Nacellidae, Cellana|AB238564|AkitaOga, Japan
Cellana toreuma|Mollusca, Gastropoda, Patellogastropoda, Nacellidae, Cellana|KM221163|Rongcheng, Shandong province, China
Cellana toreuma|Mollusca, Gastropoda, Patellogastropoda, Nacellidae, Cellana|KM221055|Shengsi, Zhejiang province, China
Cellana toreuma|Mollusca, Gastropoda, Patellogastropoda, Nacellidae, Cellana|KM221053|Zhoushan, Zhejiang province, China
Cellana toreuma|Mollusca, Gastropoda, Patellogastropoda, Nacellidae, Cellana|KM221057|Nanmi, Zhejiang province, China
Cellana toreuma|Mollusca, Gastropoda, Patellogastropoda, Nacellidae, Cellana|KM221078|Pingtan, Fujian province, China
Cellana toreuma|Mollusca, Gastropoda, Patellogastropoda, Nacellidae, Cellana|KM221079|Pingtan, Fujian province, China
Cellana toreuma|Mollusca, Gastropoda, Patellogastropoda, Nacellidae, Cellana|KM221066|Sanya, Hainan province, China
Cellana toreuma|Mollusca, Gastropoda, Patellogastropoda, Nacellidae, Cellana|AB445030|Kagawa, Japan
Cellana toreuma|Mollusca, Gastropoda, Patellogastropoda, Nacellidae, Cellana|AB445028|Aomori, Tappizaki, Japan
Cellana toreuma|Mollusca, Gastropoda, Patellogastropoda, Nacellidae, Cellana|AB445027|Miyagi Ayukawa, Japan
Cellana toreuma|Mollusca, Gastropoda, Patellogastropoda, Nacellidae, Cellana|AB445026|Kanagawa, Hayama, Japan
Cellana toreuma|Mollusca, Gastropoda, Patellogastropoda, Nacellidae, Cellana|AB445025|Mie, Japan
Cellana toreuma|Mollusca, Gastropoda, Patellogastropoda, Nacellidae, Cellana|AB445024|Aich, Morozaki, Japan
Cellana toreuma|Mollusca, Gastropoda, Patellogastropoda, Nacellidae, Cellana|AB445020| Fukui, Japan
Cellana toreuma|Mollusca, Gastropoda, Patellogastropoda, Nacellidae, Cellana|AB445019|IwateYamada, Japan
Cellana toreuma|Mollusca, Gastropoda, Patellogastropoda, Nacellidae, Cellana|AB445031|Wakayama, Japan
Cellana toreuma|Mollusca, Gastropoda, Patellogastropoda, Nacellidae, Cellana|AB445029| Miyazaki, Takanabe, Japan
Cellana toreuma|Mollusca, Gastropoda, Patellogastropoda, Nacellidae, Cellana|AB445032|Kumamoto, Reihoku, Japan
Cellana toreuma|Mollusca, Gastropoda, Patellogastropoda, Nacellidae, Cellana|KM221162|Sanya, Hainan province, China
Cellana toreuma|Mollusca, Gastropoda, Patellogastropoda, Nacellidae, Cellana|GQ455984|Japan
Cellana toreuma|Mollusca, Gastropoda, Patellogastropoda, Nacellidae, Cellana|KM221118|Qingdao, Shandong province, China
Cellana toreuma|Mollusca, Gastropoda, Patellogastropoda, Nacellidae, Cellana|GQ455985|Japan
Notoacmea schrenckii|Mollusca, Gastropoda, Patellogastropoda, Acmaeidae, Notoacmea|HM180721|Korea
Notoacmea schrenckii|Mollusca, Gastropoda, Patellogastropoda, Acmaeidae, Notoacmea|HM180723|Korea
Notoacmea schrenckii|Mollusca, Gastropoda, Patellogastropoda, Acmaeidae, Notoacmea|HM180722|Korea
Notoacmea schrenckii|Mollusca, Gastropoda, Patellogastropoda, Acmaeidae, Notoacmea|HM180724|Korea
Notoacmea schrenckii|Mollusca, Gastropoda, Patellogastropoda, Acmaeidae, Notoacmea|HM180720|Korea
Notoacmea schrenckii|Mollusca, Gastropoda, Patellogastropoda, Acmaeidae, Notoacmea|HM180719|Korea
Cellana radiata enneagona|Mollusca, Gastropoda, Docoglossa, Nacellidae, Cellana|GQ455965|Ogasawara, Japan
Cellana radiata enneagona|Mollusca, Gastropoda, Docoglossa, Nacellidae, Cellana|GQ455964|Ogasawara, Japan
Cellana radiata|Mollusca, Gastropoda, Docoglossa, Nacellidae, Cellana|AB433646|Tokyo , Minami Iwojima, Japan
Cellana radiata|Mollusca, Gastropoda, Docoglossa, Nacellidae, Cellana|AB433645|Tokyo, Ogasawara Islands, Chichijima Sakaiura, Japan
Cellana radiata|Mollusca, Gastropoda, Docoglossa, Nacellidae, Cellana|AB263731|Ogasawara Islands, Chichijima, John Beach, Japan
Cellana radiata|Mollusca, Gastropoda, Docoglossa, Nacellidae, Cellana|AB263730|Ogasawara Islands, Chichijima, John Beach, Japan
Cellana radiata|Mollusca, Gastropoda, Docoglossa, Nacellidae, Cellana|AB263729|Ogasawara, Islands, Chichijima, Miyanohama, Japan
Cellana radiata|Mollusca, Gastropoda, Docoglossa, Nacellidae, Cellana|AB263726|Ogasawara, Islands, Chichijima, Sakaiura, Japan

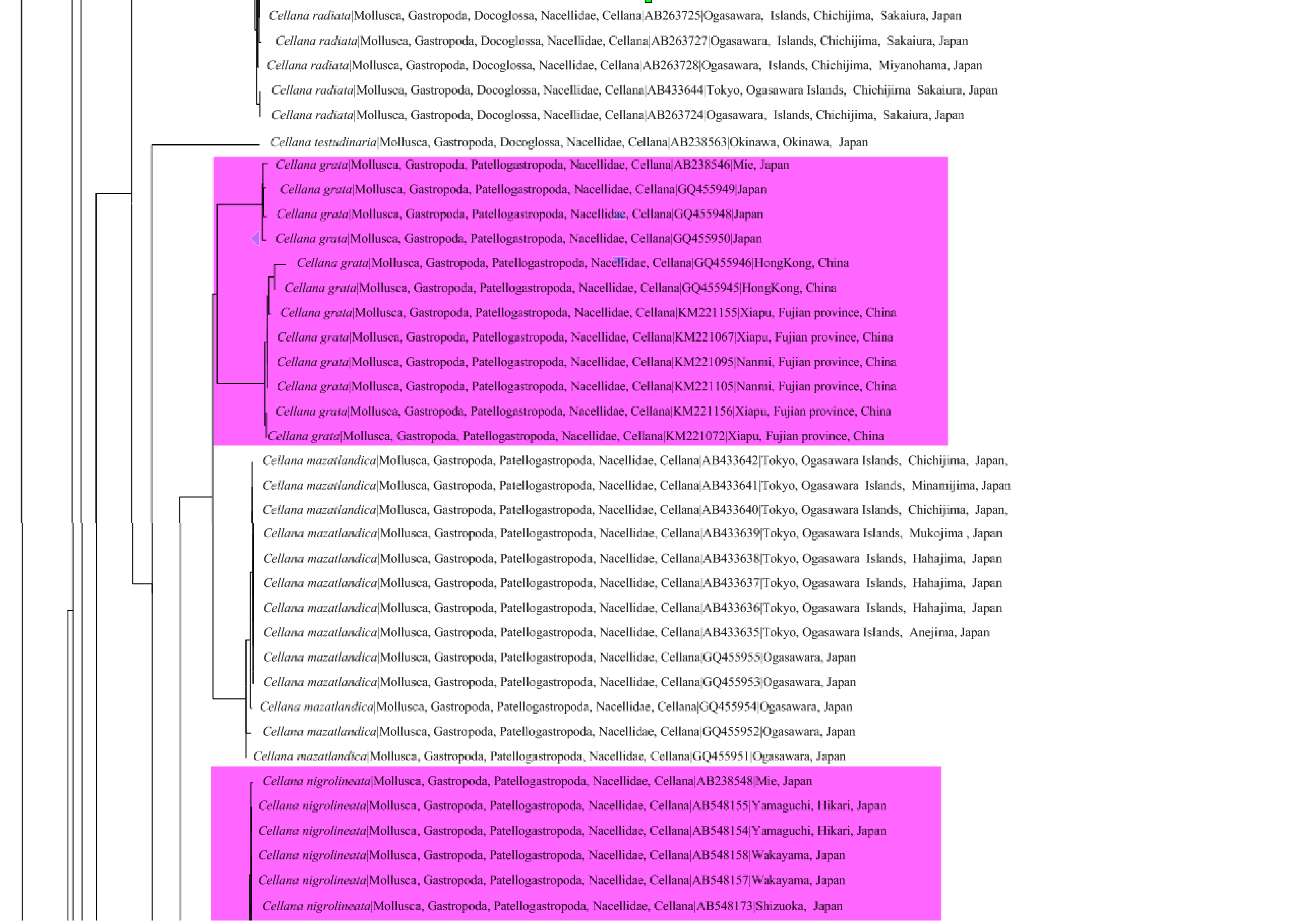
Cellana radiata|Mollusca, Gastropoda, Docoglossa, Nacellidae, Cellana|AB263725|Ogasawara, Islands, Chichijima, Sakaiura, Japan
Cellana radiata|Mollusca, Gastropoda, Docoglossa, Nacellidae, Cellana|AB263727|Ogasawara, Islands, Chichijima, Sakaiura, Japan
Cellana radiata|Mollusca, Gastropoda, Docoglossa, Nacellidae, Cellana|AB263728|Ogasawara, Islands, Chichijima, Miyanohama, Japan
Cellana radiata|Mollusca, Gastropoda, Docoglossa, Nacellidae, Cellana|AB433644|Tokyo, Ogasawara Islands, Chichijima Sakaiura, Japan
Cellana radiata|Mollusca, Gastropoda, Docoglossa, Nacellidae, Cellana|AB263724|Ogasawara, Islands, Chichijima, Sakaiura, Japan
Cellana testudinaria|Mollusca, Gastropoda, Docoglossa, Nacellidae, Cellana|AB238563|Okinawa, Okinawa, Japan
Cellana grata|Mollusca, Gastropoda, Patellogastropoda, Nacellidae, Cellana|AB238546|Mie, Japan
Cellana grata|Mollusca, Gastropoda, Patellogastropoda, Nacellidae, Cellana|GQ455949|Japan
Cellana grata|Mollusca, Gastropoda, Patellogastropoda, Nacellidae, Cellana|GQ455948|Japan
Cellana grata|Mollusca, Gastropoda, Patellogastropoda, Nacellidae, Cellana|GQ455950|Japan
Cellana grata|Mollusca, Gastropoda, Patellogastropoda, Nacellidae, Cellana|GQ455946|HongKong, China
Cellana grata|Mollusca, Gastropoda, Patellogastropoda, Nacellidae, Cellana|GQ455945|HongKong, China
Cellana grata|Mollusca, Gastropoda, Patellogastropoda, Nacellidae, Cellana|KM221155|Xiapu, Fujian province, China
Cellana grata|Mollusca, Gastropoda, Patellogastropoda, Nacellidae, Cellana|KM221067|Xiapu, Fujian province, China
Cellana grata|Mollusca, Gastropoda, Patellogastropoda, Nacellidae, Cellana|KM221095|Nanmi, Fujian province, China
Cellana grata|Mollusca, Gastropoda, Patellogastropoda, Nacellidae, Cellana|KM221105|Nanmi, Fujian province, China
Cellana grata|Mollusca, Gastropoda, Patellogastropoda, Nacellidae, Cellana|KM221156|Xiapu, Fujian province, China
Cellana grata|Mollusca, Gastropoda, Patellogastropoda, Nacellidae, Cellana|KM221072|Xiapu, Fujian province, China
Cellana mazatlandica|Mollusca, Gastropoda, Patellogastropoda, Nacellidae, Cellana|AB433642|Tokyo, Ogasawara Islands, Chichijima, Japan,
Cellana mazatlandica|Mollusca, Gastropoda, Patellogastropoda, Nacellidae, Cellana|AB433641|Tokyo, Ogasawara Islands, Minamijima, Japan
Cellana mazatlandica|Mollusca, Gastropoda, Patellogastropoda, Nacellidae, Cellana|AB433640|Tokyo, Ogasawara Islands, Chichijima, Japan,
Cellana mazatlandica|Mollusca, Gastropoda, Patellogastropoda, Nacellidae, Cellana|AB433639|Tokyo, Ogasawara Islands, Mukojima , Japan
Cellana mazatlandica|Mollusca, Gastropoda, Patellogastropoda, Nacellidae, Cellana|AB433638|Tokyo, Ogasawara Islands, Hahajima, Japan
Cellana mazatlandica|Mollusca, Gastropoda, Patellogastropoda, Nacellidae, Cellana|AB433637|Tokyo, Ogasawara Islands, Hahajima, Japan
Cellana mazatlandica|Mollusca, Gastropoda, Patellogastropoda, Nacellidae, Cellana|AB433636|Tokyo, Ogasawara Islands, Hahajima, Japan
Cellana mazatlandica|Mollusca, Gastropoda, Patellogastropoda, Nacellidae, Cellana|AB433635|Tokyo, Ogasawara Islands, Anejima, Japan
Cellana mazatlandica|Mollusca, Gastropoda, Patellogastropoda, Nacellidae, Cellana|GQ455955|Ogasawara, Japan
Cellana mazatlandica|Mollusca, Gastropoda, Patellogastropoda, Nacellidae, Cellana|GQ455953|Ogasawara, Japan
Cellana mazatlandica|Mollusca, Gastropoda, Patellogastropoda, Nacellidae, Cellana|GQ455954|Ogasawara, Japan
Cellana mazatlandica|Mollusca, Gastropoda, Patellogastropoda, Nacellidae, Cellana|GQ455952|Ogasawara, Japan
Cellana mazatlandica|Mollusca, Gastropoda, Patellogastropoda, Nacellidae, Cellana|GQ455951|Ogasawara, Japan
Cellana nigrolineata|Mollusca, Gastropoda, Patellogastropoda, Nacellidae, Cellana|AB238548|Mie, Japan
Cellana nigrolineata|Mollusca, Gastropoda, Patellogastropoda, Nacellidae, Cellana|AB548155|Yamaguchi, Hikari, Japan
Cellana nigrolineata|Mollusca, Gastropoda, Patellogastropoda, Nacellidae, Cellana|AB548154|Yamaguchi, Hikari, Japan
Cellana nigrolineata|Mollusca, Gastropoda, Patellogastropoda, Nacellidae, Cellana|AB548158|Wakayama, Japan
Cellana nigrolineata|Mollusca, Gastropoda, Patellogastropoda, Nacellidae, Cellana|AB548157|Wakayama, Japan
Cellana nigrolineata|Mollusca, Gastropoda, Patellogastropoda, Nacellidae, Cellana|AB548173|Shizuoka, Japan

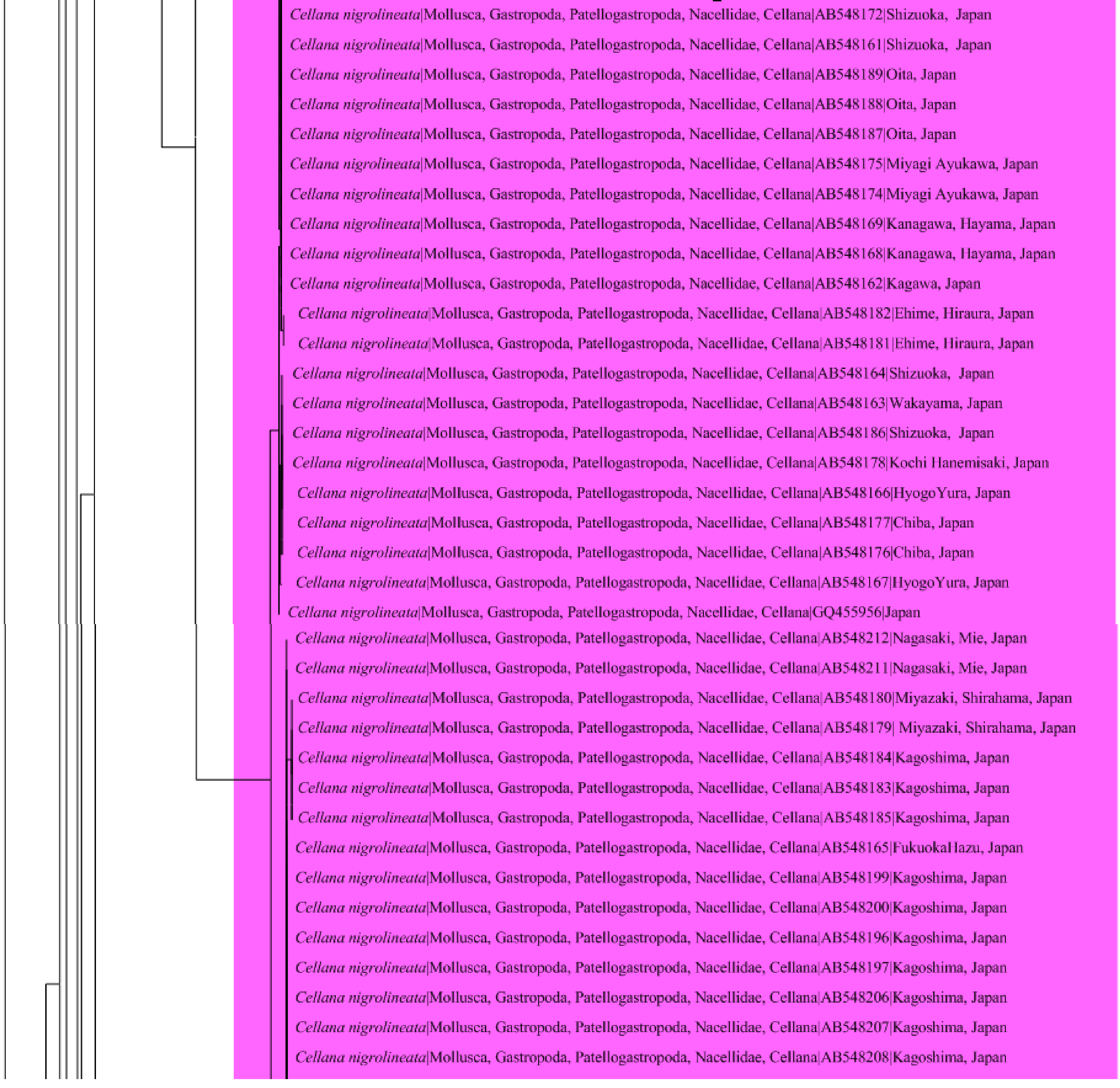
Cellana nigrolineata|Mollusca, Gastropoda, Patellogastropoda, Nacellidae, Cellana|AB548172|Shizuoka, Japan
Cellana nigrolineata|Mollusca, Gastropoda, Patellogastropoda, Nacellidae, Cellana|AB548161|Shizuoka, Japan
Cellana nigrolineata|Mollusca, Gastropoda, Patellogastropoda, Nacellidae, Cellana|AB548189|Oita, Japan
Cellana nigrolineata|Mollusca, Gastropoda, Patellogastropoda, Nacellidae, Cellana|AB548188|Oita, Japan
Cellana nigrolineata|Mollusca, Gastropoda, Patellogastropoda, Nacellidae, Cellana|AB548187|Oita, Japan
Cellana nigrolineata|Mollusca, Gastropoda, Patellogastropoda, Nacellidae, Cellana|AB548175|Miyagi Ayukawa, Japan
Cellana nigrolineata|Mollusca, Gastropoda, Patellogastropoda, Nacellidae, Cellana|AB548174|Miyagi Ayukawa, Japan
Cellana nigrolineata|Mollusca, Gastropoda, Patellogastropoda, Nacellidae, Cellana|AB548169|Kanagawa, Hayama, Japan
Cellana nigrolineata|Mollusca, Gastropoda, Patellogastropoda, Nacellidae, Cellana|AB548168|Kanagawa, Hayama, Japan
Cellana nigrolineata|Mollusca, Gastropoda, Patellogastropoda, Nacellidae, Cellana|AB548162|Kagawa, Japan
Cellana nigrolineata|Mollusca, Gastropoda, Patellogastropoda, Nacellidae, Cellana|AB548182|Ehime, Hiraura, Japan
Cellana nigrolineata|Mollusca, Gastropoda, Patellogastropoda, Nacellidae, Cellana|AB548181|Ehime, Hiraura, Japan
Cellana nigrolineata|Mollusca, Gastropoda, Patellogastropoda, Nacellidae, Cellana|AB548164|Shizuoka, Japan
Cellana nigrolineata|Mollusca, Gastropoda, Patellogastropoda, Nacellidae, Cellana|AB548163|Wakayama, Japan
Cellana nigrolineata|Mollusca, Gastropoda, Patellogastropoda, Nacellidae, Cellana|AB548186|Shizuoka, Japan
Cellana nigrolineata|Mollusca, Gastropoda, Patellogastropoda, Nacellidae, Cellana|AB548178|Kochi Hanemisaki, Japan
Cellana nigrolineata|Mollusca, Gastropoda, Patellogastropoda, Nacellidae, Cellana|AB548166|HyogoYura, Japan
Cellana nigrolineata|Mollusca, Gastropoda, Patellogastropoda, Nacellidae, Cellana|AB548177|Chiba, Japan
Cellana nigrolineata|Mollusca, Gastropoda, Patellogastropoda, Nacellidae, Cellana|AB548176|Chiba, Japan
Cellana nigrolineata|Mollusca, Gastropoda, Patellogastropoda, Nacellidae, Cellana|AB548167|HyogoYura, Japan
Cellana nigrolineata|Mollusca, Gastropoda, Patellogastropoda, Nacellidae, Cellana|GQ455956|Japan
Cellana nigrolineata|Mollusca, Gastropoda, Patellogastropoda, Nacellidae, Cellana|AB548212|Nagasaki, Mie, Japan
Cellana nigrolineata|Mollusca, Gastropoda, Patellogastropoda, Nacellidae, Cellana|AB548211|Nagasaki, Mie, Japan
Cellana nigrolineata|Mollusca, Gastropoda, Patellogastropoda, Nacellidae, Cellana|AB548180|Miyazaki, Shirahama, Japan
Cellana nigrolineata|Mollusca, Gastropoda, Patellogastropoda, Nacellidae, Cellana|AB548179| Miyazaki, Shirahama, Japan
Cellana nigrolineata|Mollusca, Gastropoda, Patellogastropoda, Nacellidae, Cellana|AB548184|Kagoshima, Japan
Cellana nigrolineata|Mollusca, Gastropoda, Patellogastropoda, Nacellidae, Cellana|AB548183|Kagoshima, Japan
Cellana nigrolineata|Mollusca, Gastropoda, Patellogastropoda, Nacellidae, Cellana|AB548185|Kagoshima, Japan
Cellana nigrolineata|Mollusca, Gastropoda, Patellogastropoda, Nacellidae, Cellana|AB548165|FukuokaHazu, Japan
Cellana nigrolineata|Mollusca, Gastropoda, Patellogastropoda, Nacellidae, Cellana|AB548199|Kagoshima, Japan
Cellana nigrolineata|Mollusca, Gastropoda, Patellogastropoda, Nacellidae, Cellana|AB548200|Kagoshima, Japan
Cellana nigrolineata|Mollusca, Gastropoda, Patellogastropoda, Nacellidae, Cellana|AB548196|Kagoshima, Japan
Cellana nigrolineata|Mollusca, Gastropoda, Patellogastropoda, Nacellidae, Cellana|AB548197|Kagoshima, Japan
Cellana nigrolineata|Mollusca, Gastropoda, Patellogastropoda, Nacellidae, Cellana|AB548206|Kagoshima, Japan
Cellana nigrolineata|Mollusca, Gastropoda, Patellogastropoda, Nacellidae, Cellana|AB548207|Kagoshima, Japan
Cellana nigrolineata|Mollusca, Gastropoda, Patellogastropoda, Nacellidae, Cellana|AB548208|Kagoshima, Japan

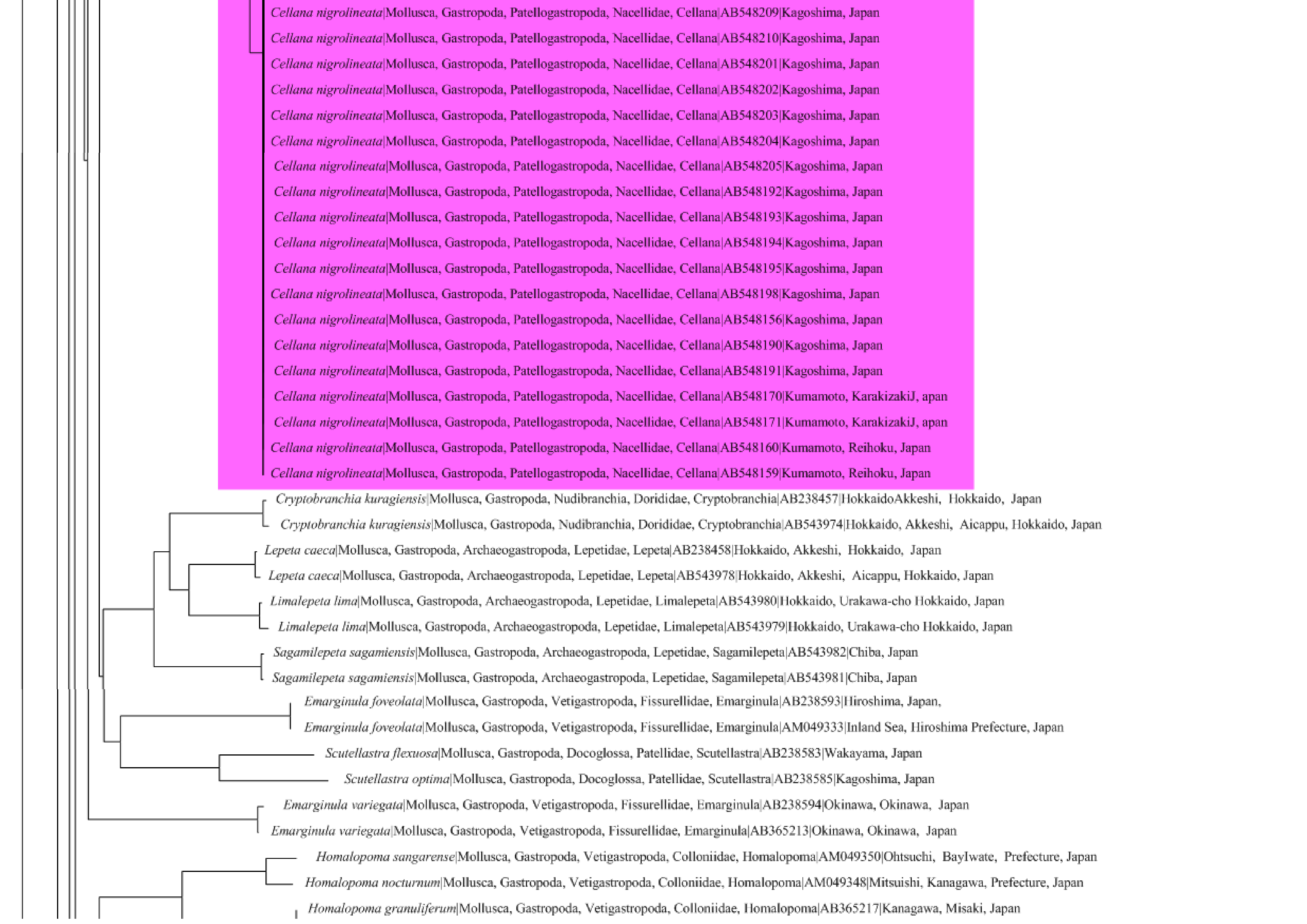
Cellana nigrolineata|Mollusca, Gastropoda, Patellogastropoda, Nacellidae, Cellana|AB548209|Kagoshima, Japan
Cellana nigrolineata|Mollusca, Gastropoda, Patellogastropoda, Nacellidae, Cellana|AB548210|Kagoshima, Japan
Cellana nigrolineata|Mollusca, Gastropoda, Patellogastropoda, Nacellidae, Cellana|AB548201|Kagoshima, Japan
Cellana nigrolineata|Mollusca, Gastropoda, Patellogastropoda, Nacellidae, Cellana|AB548202|Kagoshima, Japan
Cellana nigrolineata|Mollusca, Gastropoda, Patellogastropoda, Nacellidae, Cellana|AB548203|Kagoshima, Japan
Cellana nigrolineata|Mollusca, Gastropoda, Patellogastropoda, Nacellidae, Cellana|AB548204|Kagoshima, Japan
Cellana nigrolineata|Mollusca, Gastropoda, Patellogastropoda, Nacellidae, Cellana|AB548205|Kagoshima, Japan
Cellana nigrolineata|Mollusca, Gastropoda, Patellogastropoda, Nacellidae, Cellana|AB548192|Kagoshima, Japan
Cellana nigrolineata|Mollusca, Gastropoda, Patellogastropoda, Nacellidae, Cellana|AB548193|Kagoshima, Japan
Cellana nigrolineata|Mollusca, Gastropoda, Patellogastropoda, Nacellidae, Cellana|AB548194|Kagoshima, Japan
Cellana nigrolineata|Mollusca, Gastropoda, Patellogastropoda, Nacellidae, Cellana|AB548195|Kagoshima, Japan
Cellana nigrolineata|Mollusca, Gastropoda, Patellogastropoda, Nacellidae, Cellana|AB548198|Kagoshima, Japan
Cellana nigrolineata|Mollusca, Gastropoda, Patellogastropoda, Nacellidae, Cellana|AB548156|Kagoshima, Japan
Cellana nigrolineata|Mollusca, Gastropoda, Patellogastropoda, Nacellidae, Cellana|AB548190|Kagoshima, Japan
Cellana nigrolineata|Mollusca, Gastropoda, Patellogastropoda, Nacellidae, Cellana|AB548191|Kagoshima, Japan
Cellana nigrolineata|Mollusca, Gastropoda, Patellogastropoda, Nacellidae, Cellana|AB548170|Kumamoto, KarakizakiJ, apan
Cellana nigrolineata|Mollusca, Gastropoda, Patellogastropoda, Nacellidae, Cellana|AB548171|Kumamoto, KarakizakiJ, apan
Cellana nigrolineata|Mollusca, Gastropoda, Patellogastropoda, Nacellidae, Cellana|AB548160|Kumamoto, Reihoku, Japan
Cellana nigrolineata|Mollusca, Gastropoda, Patellogastropoda, Nacellidae, Cellana|AB548159|Kumamoto, Reihoku, Japan
Cryptobranchia kuragiensis|Mollusca, Gastropoda, Nudibranchia, Dorididae, Cryptobranchia|AB238457|HokkaidoAkkeshi, Hokkaido, Japan
Cryptobranchia kuragiensis|Mollusca, Gastropoda, Nudibranchia, Dorididae, Cryptobranchia|AB543974|Hokkaido, Akkeshi, Aicappu, Hokkaido, Japan
Lepeta caeca|Mollusca, Gastropoda, Archaeogastropoda, Lepetidae, Lepeta|AB238458|Hokkaido, Akkeshi, Hokkaido, Japan
Lepeta caeca|Mollusca, Gastropoda, Archaeogastropoda, Lepetidae, Lepeta|AB543978|Hokkaido, Akkeshi, Aicappu, Hokkaido, Japan
Limalepeta lima|Mollusca, Gastropoda, Archaeogastropoda, Lepetidae, Limalepeta|AB543980|Hokkaido, Urakawa-cho Hokkaido, Japan
Limalepeta lima|Mollusca, Gastropoda, Archaeogastropoda, Lepetidae, Limalepeta|AB543979|Hokkaido, Urakawa-cho Hokkaido, Japan
Sagamilepeta sagamiensis|Mollusca, Gastropoda, Archaeogastropoda, Lepetidae, Sagamilepeta|AB543982|Chiba, Japan
Sagamilepeta sagamiensis|Mollusca, Gastropoda, Archaeogastropoda, Lepetidae, Sagamilepeta|AB543981|Chiba, Japan
Emarginula foveolata|Mollusca, Gastropoda, Vetigastropoda, Fissurellidae, Emarginula|AB238593|Hiroshima, Japan,
Emarginula foveolata|Mollusca, Gastropoda, Vetigastropoda, Fissurellidae, Emarginula|AM049333|Inland Sea, Hiroshima Prefecture, Japan
Scutellastra flexuosa|Mollusca, Gastropoda, Docoglossa, Patellidae, Scutellastra|AB238583|Wakayama, Japan
Scutellastra optima|Mollusca, Gastropoda, Docoglossa, Patellidae, Scutellastra|AB238585|Kagoshima, Japan
Emarginula variegata|Mollusca, Gastropoda, Vetigastropoda, Fissurellidae, Emarginula|AB238594|Okinawa, Okinawa, Japan
Emarginula variegata|Mollusca, Gastropoda, Vetigastropoda, Fissurellidae, Emarginula|AB365213|Okinawa, Okinawa, Japan
Homalopoma sangarense|Mollusca, Gastropoda, Vetigastropoda, Colloniidae, Homalopoma|AM049350|Ohtsuchi, BayIwate, Prefecture, Japan
Homalopoma nocturnum|Mollusca, Gastropoda, Vetigastropoda, Colloniidae, Homalopoma|AM049348|Mitsuishi, Kanagawa, Prefecture, Japan
Homalopoma granuliferum|Mollusca, Gastropoda, Vetigastropoda, Colloniidae, Homalopoma|AB365217|Kanagawa, Misaki, Japan

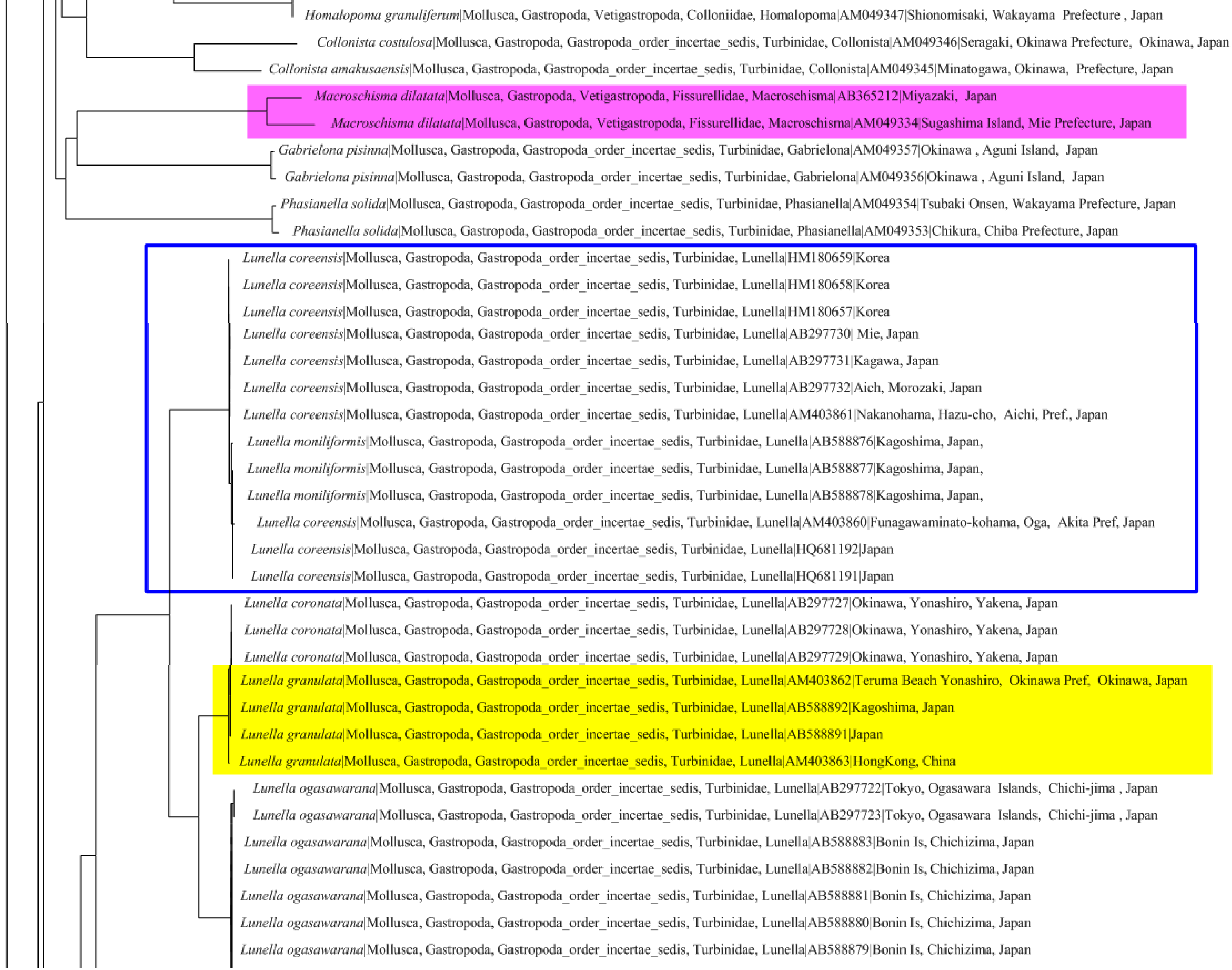

Homalopoma granuliferum|Mollusca, Gastropoda, Vetigastropoda, Colloniidae, Homalopoma|AM049347|Shionomisaki, Wakayama Prefecture , Japan
Collonista costulosa|Mollusca, Gastropoda, Gastropoda_order_incertae_sedis, Turbinidae, Collonista|AM049346|Seragaki, Okinawa Prefecture, Okinawa, Japan
Collonista amakusaensis|Mollusca, Gastropoda, Gastropoda_order_incertae_sedis, Turbinidae, Collonista|AM049345|Minatogawa, Okinawa, Prefecture, Japan
Macroschisma dilatata|Mollusca, Gastropoda, Vetigastropoda, Fissurellidae, Macroschisma|AB365212|Miyazaki, Japan
Macroschisma dilatata|Mollusca, Gastropoda, Vetigastropoda, Fissurellidae, Macroschisma|AM049334|Sugashima Island, Mie Prefecture, Japan
Gabrielona pisinna|Mollusca, Gastropoda, Gastropoda_order_incertae_sedis, Turbinidae, Gabrielona|AM049357|Okinawa , Aguni Island, Japan
Gabrielona pisinna|Mollusca, Gastropoda, Gastropoda_order_incertae_sedis, Turbinidae, Gabrielona|AM049356|Okinawa , Aguni Island, Japan
Phasianella solida|Mollusca, Gastropoda, Gastropoda_order_incertae_sedis, Turbinidae, Phasianella|AM049354|Tsubaki Onsen, Wakayama Prefecture, Japan
Phasianella solida|Mollusca, Gastropoda, Gastropoda_order_incertae_sedis, Turbinidae, Phasianella|AM049353|Chikura, Chiba Prefecture, Japan
Lunella coreensis|Mollusca, Gastropoda, Gastropoda_order_incertae_sedis, Turbinidae, Lunella|HM180659|Korea
Lunella coreensis|Mollusca, Gastropoda, Gastropoda_order_incertae_sedis, Turbinidae, Lunella|HM180658|Korea
Lunella coreensis|Mollusca, Gastropoda, Gastropoda_order_incertae_sedis, Turbinidae, Lunella|HM180657|Korea
Lunella coreensis|Mollusca, Gastropoda, Gastropoda_order_incertae_sedis, Turbinidae, Lunella|AB297730| Mie, Japan
Lunella coreensis|Mollusca, Gastropoda, Gastropoda_order_incertae_sedis, Turbinidae, Lunella|AB297731|Kagawa, Japan
Lunella coreensis|Mollusca, Gastropoda, Gastropoda_order_incertae_sedis, Turbinidae, Lunella|AB297732|Aich, Morozaki, Japan
Lunella coreensis|Mollusca, Gastropoda, Gastropoda_order_incertae_sedis, Turbinidae, Lunella|AM403861|Nakanohama, Hazu-cho, Aichi, Pref., Japan
Lunella moniliformis|Mollusca, Gastropoda, Gastropoda_order_incertae_sedis, Turbinidae, Lunella|AB588876|Kagoshima, Japan,
Lunella moniliformis|Mollusca, Gastropoda, Gastropoda_order_incertae_sedis, Turbinidae, Lunella|AB588877|Kagoshima, Japan,
Lunella moniliformis|Mollusca, Gastropoda, Gastropoda_order_incertae_sedis, Turbinidae, Lunella|AB588878|Kagoshima, Japan,
Lunella coreensis|Mollusca, Gastropoda, Gastropoda_order_incertae_sedis, Turbinidae, Lunella|AM403860|Funagawaminato-kohama, Oga, Akita Pref, Japan
Lunella coreensis|Mollusca, Gastropoda, Gastropoda_order_incertae_sedis, Turbinidae, Lunella|HQ681192|Japan
Lunella coreensis|Mollusca, Gastropoda, Gastropoda_order_incertae_sedis, Turbinidae, Lunella|HQ681191|Japan
Lunella coronata|Mollusca, Gastropoda, Gastropoda_order_incertae_sedis, Turbinidae, Lunella|AB297727|Okinawa, Yonashiro, Yakena, Japan
Lunella coronata|Mollusca, Gastropoda, Gastropoda_order_incertae_sedis, Turbinidae, Lunella|AB297728|Okinawa, Yonashiro, Yakena, Japan
Lunella coronata|Mollusca, Gastropoda, Gastropoda_order_incertae_sedis, Turbinidae, Lunella|AB297729|Okinawa, Yonashiro, Yakena, Japan
Lunella granulata|Mollusca, Gastropoda, Gastropoda_order_incertae_sedis, Turbinidae, Lunella|AM403862|Teruma Beach Yonashiro, Okinawa Pref, Okinawa, Japan
Lunella granulata|Mollusca, Gastropoda, Gastropoda_order_incertae_sedis, Turbinidae, Lunella|AB588892|Kagoshima, Japan
Lunella granulata|Mollusca, Gastropoda, Gastropoda_order_incertae_sedis, Turbinidae, Lunella|AB588891|Japan
Lunella granulata|Mollusca, Gastropoda, Gastropoda_order_incertae_sedis, Turbinidae, Lunella|AM403863|HongKong, China
Lunella ogasawarana|Mollusca, Gastropoda, Gastropoda_order_incertae_sedis, Turbinidae, Lunella|AB297722|Tokyo, Ogasawara Islands, Chichi-jima , Japan
Lunella ogasawarana|Mollusca, Gastropoda, Gastropoda_order_incertae_sedis, Turbinidae, Lunella|AB297723|Tokyo, Ogasawara Islands, Chichi-jima , Japan
Lunella ogasawarana|Mollusca, Gastropoda, Gastropoda_order_incertae_sedis, Turbinidae, Lunella|AB588883|Bonin Is, Chichizima, Japan
Lunella ogasawarana|Mollusca, Gastropoda, Gastropoda_order_incertae_sedis, Turbinidae, Lunella|AB588882|Bonin Is, Chichizima, Japan
Lunella ogasawarana|Mollusca, Gastropoda, Gastropoda_order_incertae_sedis, Turbinidae, Lunella|AB588881|Bonin Is, Chichizima, Japan
Lunella ogasawarana|Mollusca, Gastropoda, Gastropoda_order_incertae_sedis, Turbinidae, Lunella|AB588880|Bonin Is, Chichizima, Japan
Lunella ogasawarana|Mollusca, Gastropoda, Gastropoda_order_incertae_sedis, Turbinidae, Lunella|AB588879|Bonin Is, Chichizima, Japan

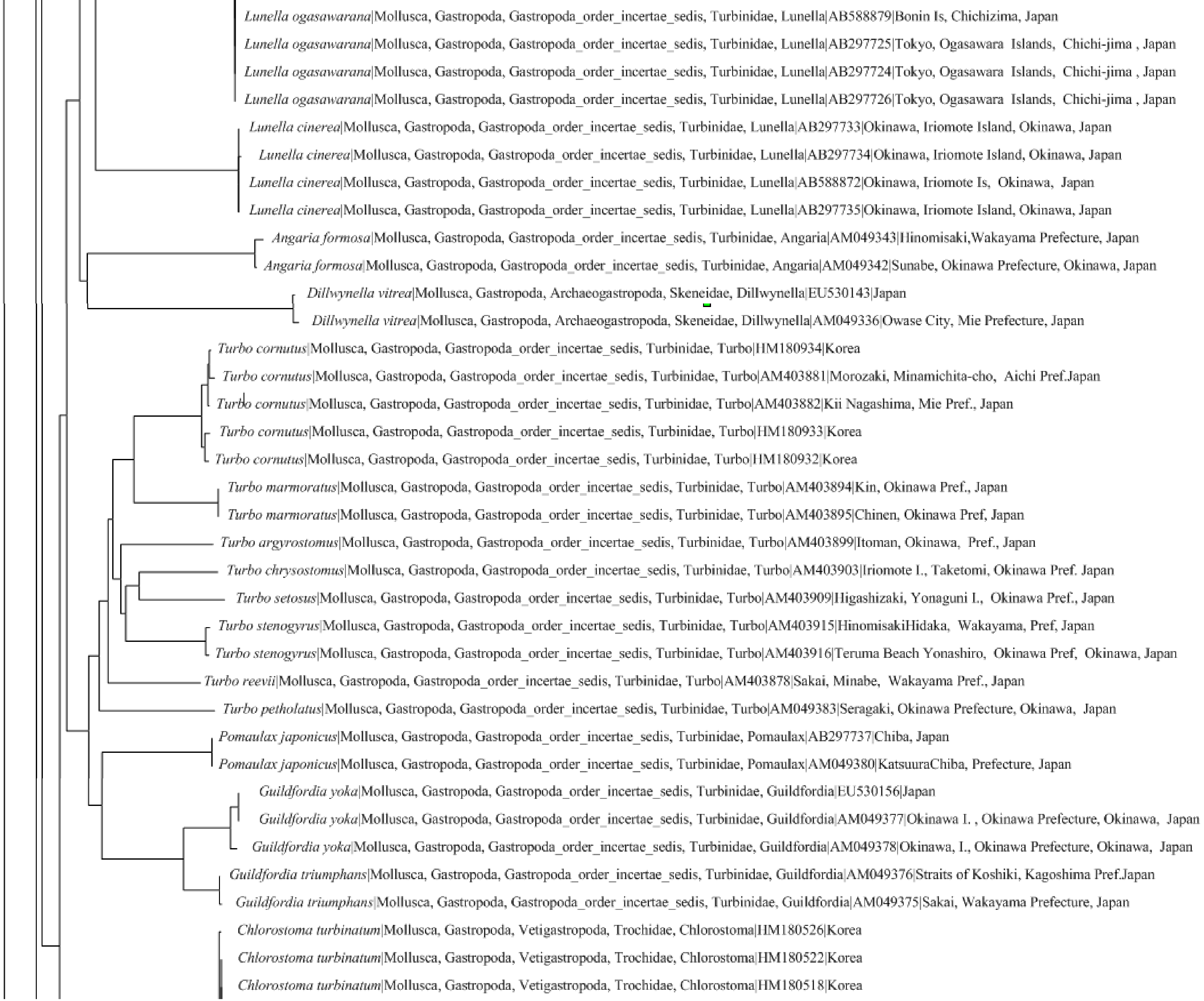
Lunella ogasawarana|Mollusca, Gastropoda, Gastropoda_order_incertae_sedis, Turbinidae, Lunella|AB588879|Bonin Is, Chichizima, Japan
Lunella ogasawarana|Mollusca, Gastropoda, Gastropoda_order_incertae_sedis, Turbinidae, Lunella|AB297725|Tokyo, Ogasawara Islands, Chichi-jima , Japan
Lunella ogasawarana|Mollusca, Gastropoda, Gastropoda_order_incertae_sedis, Turbinidae, Lunella|AB297724|Tokyo, Ogasawara Islands, Chichi-jima , Japan
Lunella ogasawarana|Mollusca, Gastropoda, Gastropoda_order_incertae_sedis, Turbinidae, Lunella|AB297726|Tokyo, Ogasawara Islands, Chichi-jima , Japan
Lunella cinerea|Mollusca, Gastropoda, Gastropoda_order_incertae_sedis, Turbinidae, Lunella|AB297733|Okinawa, Iriomote Island, Okinawa, Japan
Lunella cinerea|Mollusca, Gastropoda, Gastropoda_order_incertae_sedis, Turbinidae, Lunella|AB297734|Okinawa, Iriomote Island, Okinawa, Japan
Lunella cinerea|Mollusca, Gastropoda, Gastropoda_order_incertae_sedis, Turbinidae, Lunella|AB588872|Okinawa, Iriomote Is, Okinawa, Japan
Lunella cinerea|Mollusca, Gastropoda, Gastropoda_order_incertae_sedis, Turbinidae, Lunella|AB297735|Okinawa, Iriomote Island, Okinawa, Japan
Angaria formosa|Mollusca, Gastropoda, Gastropoda_order_incertae_sedis, Turbinidae, Angaria|AM049343|Hinomisaki,Wakayama Prefecture, Japan
Angaria formosa|Mollusca, Gastropoda, Gastropoda_order_incertae_sedis, Turbinidae, Angaria|AM049342|Sunabe, Okinawa Prefecture, Okinawa, Japan
Dillwynella vitrea|Mollusca, Gastropoda, Archaeogastropoda, Skeneidae, Dillwynella|EU530143|Japan
Dillwynella vitrea|Mollusca, Gastropoda, Archaeogastropoda, Skeneidae, Dillwynella|AM049336|Owase City, Mie Prefecture, Japan
Turbo cornutus|Mollusca, Gastropoda, Gastropoda_order_incertae_sedis, Turbinidae, Turbo|HM180934|Korea
Turbo cornutus|Mollusca, Gastropoda, Gastropoda_order_incertae_sedis, Turbinidae, Turbo|AM403881|Morozaki, Minamichita-cho, Aichi Pref.Japan
Turbo cornutus|Mollusca, Gastropoda, Gastropoda_order_incertae_sedis, Turbinidae, Turbo|AM403882|Kii Nagashima, Mie Pref., Japan
Turbo cornutus|Mollusca, Gastropoda, Gastropoda_order_incertae_sedis, Turbinidae, Turbo|HM180933|Korea
Turbo cornutus|Mollusca, Gastropoda, Gastropoda_order_incertae_sedis, Turbinidae, Turbo|HM180932|Korea
Turbo marmoratus|Mollusca, Gastropoda, Gastropoda_order_incertae_sedis, Turbinidae, Turbo|AM403894|Kin, Okinawa Pref., Japan
Turbo marmoratus|Mollusca, Gastropoda, Gastropoda_order_incertae_sedis, Turbinidae, Turbo|AM403895|Chinen, Okinawa Pref, Japan
Turbo argyrostomus|Mollusca, Gastropoda, Gastropoda_order_incertae_sedis, Turbinidae, Turbo|AM403899|Itoman, Okinawa, Pref., Japan
Turbo chrysostomus|Mollusca, Gastropoda, Gastropoda_order_incertae_sedis, Turbinidae, Turbo|AM403903|Iriomote I., Taketomi, Okinawa Pref. Japan
Turbo setosus|Mollusca, Gastropoda, Gastropoda_order_incertae_sedis, Turbinidae, Turbo|AM403909|Higashizaki, Yonaguni I., Okinawa Pref., Japan
Turbo stenogyrus|Mollusca, Gastropoda, Gastropoda_order_incertae_sedis, Turbinidae, Turbo|AM403915|HinomisakiHidaka, Wakayama, Pref, Japan
Turbo stenogyrus|Mollusca, Gastropoda, Gastropoda_order_incertae_sedis, Turbinidae, Turbo|AM403916|Teruma Beach Yonashiro, Okinawa Pref, Okinawa, Japan
Turbo reevii|Mollusca, Gastropoda, Gastropoda_order_incertae_sedis, Turbinidae, Turbo|AM403878|Sakai, Minabe, Wakayama Pref., Japan
Turbo petholatus|Mollusca, Gastropoda, Gastropoda_order_incertae_sedis, Turbinidae, Turbo|AM049383|Seragaki, Okinawa Prefecture, Okinawa, Japan
Pomaulax japonicus|Mollusca, Gastropoda, Gastropoda_order_incertae_sedis, Turbinidae, Pomaulax|AB297737|Chiba, Japan
Pomaulax japonicus|Mollusca, Gastropoda, Gastropoda_order_incertae_sedis, Turbinidae, Pomaulax|AM049380|KatsuuraChiba, Prefecture, Japan
Guildfordia yoka|Mollusca, Gastropoda, Gastropoda_order_incertae_sedis, Turbinidae, Guildfordia|EU530156|Japan
Guildfordia yoka|Mollusca, Gastropoda, Gastropoda_order_incertae_sedis, Turbinidae, Guildfordia|AM049377|Okinawa I. , Okinawa Prefecture, Okinawa, Japan
Guildfordia yoka|Mollusca, Gastropoda, Gastropoda_order_incertae_sedis, Turbinidae, Guildfordia|AM049378|Okinawa, I., Okinawa Prefecture, Okinawa, Japan
Guildfordia triumphans|Mollusca, Gastropoda, Gastropoda_order_incertae_sedis, Turbinidae, Guildfordia|AM049376|Straits of Koshiki, Kagoshima Pref.Japan
Guildfordia triumphans|Mollusca, Gastropoda, Gastropoda_order_incertae_sedis, Turbinidae, Guildfordia|AM049375|Sakai, Wakayama Prefecture, Japan
Chlorostoma turbinatum|Mollusca, Gastropoda, Vetigastropoda, Trochidae, Chlorostoma|HM180526|Korea
Chlorostoma turbinatum|Mollusca, Gastropoda, Vetigastropoda, Trochidae, Chlorostoma|HM180522|Korea
Chlorostoma turbinatum|Mollusca, Gastropoda, Vetigastropoda, Trochidae, Chlorostoma|HM180518|Korea

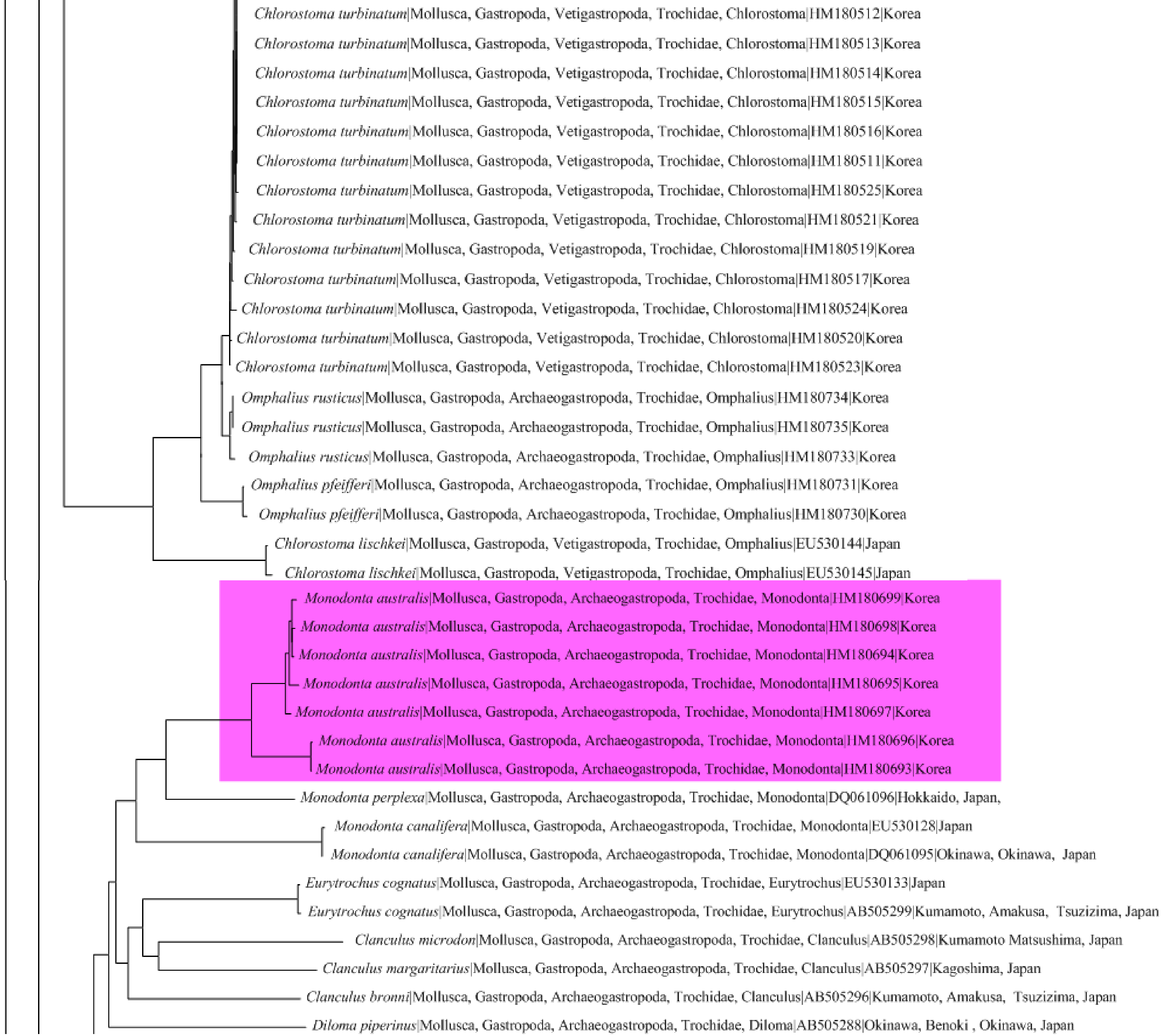

Chlorostoma turbinatum|Mollusca, Gastropoda, Vetigastropoda, Trochidae, Chlorostoma|HM180512|Korea
Chlorostoma turbinatum|Mollusca, Gastropoda, Vetigastropoda, Trochidae, Chlorostoma|HM180513|Korea
Chlorostoma turbinatum|Mollusca, Gastropoda, Vetigastropoda, Trochidae, Chlorostoma|HM180514|Korea
Chlorostoma turbinatum|Mollusca, Gastropoda, Vetigastropoda, Trochidae, Chlorostoma|HM180515|Korea
Chlorostoma turbinatum|Mollusca, Gastropoda, Vetigastropoda, Trochidae, Chlorostoma|HM180516|Korea
Chlorostoma turbinatum|Mollusca, Gastropoda, Vetigastropoda, Trochidae, Chlorostoma|HM180511|Korea
Chlorostoma turbinatum|Mollusca, Gastropoda, Vetigastropoda, Trochidae, Chlorostoma|HM180525|Korea
Chlorostoma turbinatum|Mollusca, Gastropoda, Vetigastropoda, Trochidae, Chlorostoma|HM180521|Korea
Chlorostoma turbinatum|Mollusca, Gastropoda, Vetigastropoda, Trochidae, Chlorostoma|HM180519|Korea
Chlorostoma turbinatum|Mollusca, Gastropoda, Vetigastropoda, Trochidae, Chlorostoma|HM180517|Korea
Chlorostoma turbinatum|Mollusca, Gastropoda, Vetigastropoda, Trochidae, Chlorostoma|HM180524|Korea
Chlorostoma turbinatum|Mollusca, Gastropoda, Vetigastropoda, Trochidae, Chlorostoma|HM180520|Korea
Chlorostoma turbinatum|Mollusca, Gastropoda, Vetigastropoda, Trochidae, Chlorostoma|HM180523|Korea
Omphalius rusticus|Mollusca, Gastropoda, Archaeogastropoda, Trochidae, Omphalius|HM180734|Korea
Omphalius rusticus|Mollusca, Gastropoda, Archaeogastropoda, Trochidae, Omphalius|HM180735|Korea
Omphalius rusticus|Mollusca, Gastropoda, Archaeogastropoda, Trochidae, Omphalius|HM180733|Korea
Omphalius pfeifferi|Mollusca, Gastropoda, Archaeogastropoda, Trochidae, Omphalius|HM180731|Korea
Omphalius pfeifferi|Mollusca, Gastropoda, Archaeogastropoda, Trochidae, Omphalius|HM180730|Korea
Chlorostoma lischkei|Mollusca, Gastropoda, Vetigastropoda, Trochidae, Omphalius|EU530144|Japan
Chlorostoma lischkei|Mollusca, Gastropoda, Vetigastropoda, Trochidae, Omphalius|EU530145|Japan
Monodonta australis|Mollusca, Gastropoda, Archaeogastropoda, Trochidae, Monodonta|HM180699|Korea
Monodonta australis|Mollusca, Gastropoda, Archaeogastropoda, Trochidae, Monodonta|HM180698|Korea
Monodonta australis|Mollusca, Gastropoda, Archaeogastropoda, Trochidae, Monodonta|HM180694|Korea
Monodonta australis|Mollusca, Gastropoda, Archaeogastropoda, Trochidae, Monodonta|HM180695|Korea
Monodonta australis|Mollusca, Gastropoda, Archaeogastropoda, Trochidae, Monodonta|HM180697|Korea
Monodonta australis|Mollusca, Gastropoda, Archaeogastropoda, Trochidae, Monodonta|HM180696|Korea
Monodonta australis|Mollusca, Gastropoda, Archaeogastropoda, Trochidae, Monodonta|HM180693|Korea
Monodonta perplexa|Mollusca, Gastropoda, Archaeogastropoda, Trochidae, Monodonta|DQ061096|Hokkaido, Japan,
Monodonta canalifera|Mollusca, Gastropoda, Archaeogastropoda, Trochidae, Monodonta|EU530128|Japan
Monodonta canalifera|Mollusca, Gastropoda, Archaeogastropoda, Trochidae, Monodonta|DQ061095|Okinawa, Okinawa, Japan
Eurytrochus cognatus|Mollusca, Gastropoda, Archaeogastropoda, Trochidae, Eurytrochus|EU530133|Japan
Eurytrochus cognatus|Mollusca, Gastropoda, Archaeogastropoda, Trochidae, Eurytrochus|AB505299|Kumamoto, Amakusa, Tsuzizima, Japan
Clanculus microdon|Mollusca, Gastropoda, Archaeogastropoda, Trochidae, Clanculus|AB505298|Kumamoto Matsushima, Japan
Clanculus margaritarius|Mollusca, Gastropoda, Archaeogastropoda, Trochidae, Clanculus|AB505297|Kagoshima, Japan
Clanculus bronni|Mollusca, Gastropoda, Archaeogastropoda, Trochidae, Clanculus|AB505296|Kumamoto, Amakusa, Tsuzizima, Japan
Diloma piperinus|Mollusca, Gastropoda, Archaeogastropoda, Trochidae, Diloma|AB505288|Okinawa, Benoki , Okinawa, Japan

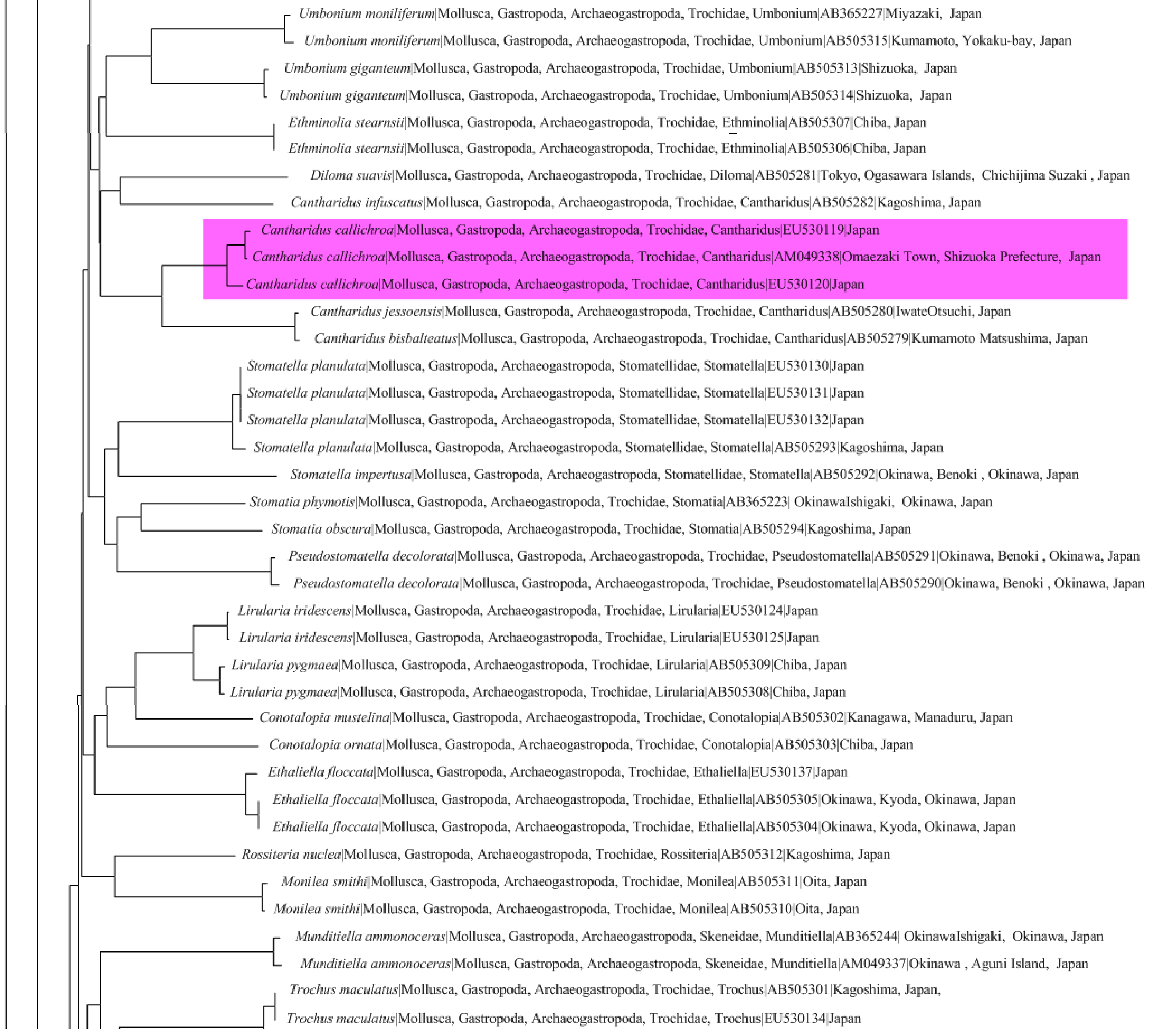

Umbonium moniliferum|Mollusca, Gastropoda, Archaeogastropoda, Trochidae, Umbonium|AB365227|Miyazaki, Japan
Umbonium moniliferum|Mollusca, Gastropoda, Archaeogastropoda, Trochidae, Umbonium|AB505315|Kumamoto, Yokaku-bay, Japan
Umbonium giganteum|Mollusca, Gastropoda, Archaeogastropoda, Trochidae, Umbonium|AB505313|Shizuoka, Japan
Umbonium giganteum|Mollusca, Gastropoda, Archaeogastropoda, Trochidae, Umbonium|AB505314|Shizuoka, Japan
Ethminolia stearnsii|Mollusca, Gastropoda, Archaeogastropoda, Trochidae, Ethminolia|AB505307|Chiba, Japan
Ethminolia stearnsii|Mollusca, Gastropoda, Archaeogastropoda, Trochidae, Ethminolia|AB505306|Chiba, Japan
Diloma suavis|Mollusca, Gastropoda, Archaeogastropoda, Trochidae, Diloma|AB505281|Tokyo, Ogasawara Islands, Chichijima Suzaki , Japan
Cantharidus infuscatus|Mollusca, Gastropoda, Archaeogastropoda, Trochidae, Cantharidus|AB505282|Kagoshima, Japan
Cantharidus callichroa|Mollusca, Gastropoda, Archaeogastropoda, Trochidae, Cantharidus|EU530119|Japan
Cantharidus callichroa|Mollusca, Gastropoda, Archaeogastropoda, Trochidae, Cantharidus|AM049338|Omaezaki Town, Shizuoka Prefecture, Japan
Cantharidus callichroa|Mollusca, Gastropoda, Archaeogastropoda, Trochidae, Cantharidus|EU530120|Japan
Cantharidus jessoensis|Mollusca, Gastropoda, Archaeogastropoda, Trochidae, Cantharidus|AB505280|IwateOtsuchi, Japan
Cantharidus bisbalteatus|Mollusca, Gastropoda, Archaeogastropoda, Trochidae, Cantharidus|AB505279|Kumamoto Matsushima, Japan
Stomatella planulata|Mollusca, Gastropoda, Archaeogastropoda, Stomatellidae, Stomatella|EU530130|Japan
Stomatella planulata|Mollusca, Gastropoda, Archaeogastropoda, Stomatellidae, Stomatella|EU530131|Japan
Stomatella planulata|Mollusca, Gastropoda, Archaeogastropoda, Stomatellidae, Stomatella|EU530132|Japan
Stomatella planulata|Mollusca, Gastropoda, Archaeogastropoda, Stomatellidae, Stomatella|AB505293|Kagoshima, Japan
Stomatella impertusa|Mollusca, Gastropoda, Archaeogastropoda, Stomatellidae, Stomatella|AB505292|Okinawa, Benoki , Okinawa, Japan
Stomatia phymotis|Mollusca, Gastropoda, Archaeogastropoda, Trochidae, Stomatia|AB365223| OkinawaIshigaki, Okinawa, Japan
Stomatia obscura|Mollusca, Gastropoda, Archaeogastropoda, Trochidae, Stomatia|AB505294|Kagoshima, Japan
Pseudostomatella decolorata|Mollusca, Gastropoda, Archaeogastropoda, Trochidae, Pseudostomatella|AB505291|Okinawa, Benoki , Okinawa, Japan
Pseudostomatella decolorata|Mollusca, Gastropoda, Archaeogastropoda, Trochidae, Pseudostomatella|AB505290|Okinawa, Benoki , Okinawa, Japan
Lirularia iridescens|Mollusca, Gastropoda, Archaeogastropoda, Trochidae, Lirularia|EU530124|Japan
Lirularia iridescens|Mollusca, Gastropoda, Archaeogastropoda, Trochidae, Lirularia|EU530125|Japan
Lirularia pygmaea|Mollusca, Gastropoda, Archaeogastropoda, Trochidae, Lirularia|AB505309|Chiba, Japan
Lirularia pygmaea|Mollusca, Gastropoda, Archaeogastropoda, Trochidae, Lirularia|AB505308|Chiba, Japan
Conotalopia mustelina|Mollusca, Gastropoda, Archaeogastropoda, Trochidae, Conotalopia|AB505302|Kanagawa, Manaduru, Japan
Conotalopia ornata|Mollusca, Gastropoda, Archaeogastropoda, Trochidae, Conotalopia|AB505303|Chiba, Japan
Ethaliella floccata|Mollusca, Gastropoda, Archaeogastropoda, Trochidae, Ethaliella|EU530137|Japan
Ethaliella floccata|Mollusca, Gastropoda, Archaeogastropoda, Trochidae, Ethaliella|AB505305|Okinawa, Kyoda, Okinawa, Japan
Ethaliella floccata|Mollusca, Gastropoda, Archaeogastropoda, Trochidae, Ethaliella|AB505304|Okinawa, Kyoda, Okinawa, Japan
Rossiteria nuclea|Mollusca, Gastropoda, Archaeogastropoda, Trochidae, Rossiteria|AB505312|Kagoshima, Japan
Monilea smithi|Mollusca, Gastropoda, Archaeogastropoda, Trochidae, Monilea|AB505311|Oita, Japan
Monilea smithi|Mollusca, Gastropoda, Archaeogastropoda, Trochidae, Monilea|AB505310|Oita, Japan
Munditiella ammonoceras|Mollusca, Gastropoda, Archaeogastropoda, Skeneidae, Munditiella|AB365244| OkinawaIshigaki, Okinawa, Japan
Munditiella ammonoceras|Mollusca, Gastropoda, Archaeogastropoda, Skeneidae, Munditiella|AM049337|Okinawa , Aguni Island, Japan
Trochus maculatus|Mollusca, Gastropoda, Archaeogastropoda, Trochidae, Trochus|AB505301|Kagoshima, Japan,
Trochus maculatus|Mollusca, Gastropoda, Archaeogastropoda, Trochidae, Trochus|EU530134|Japan

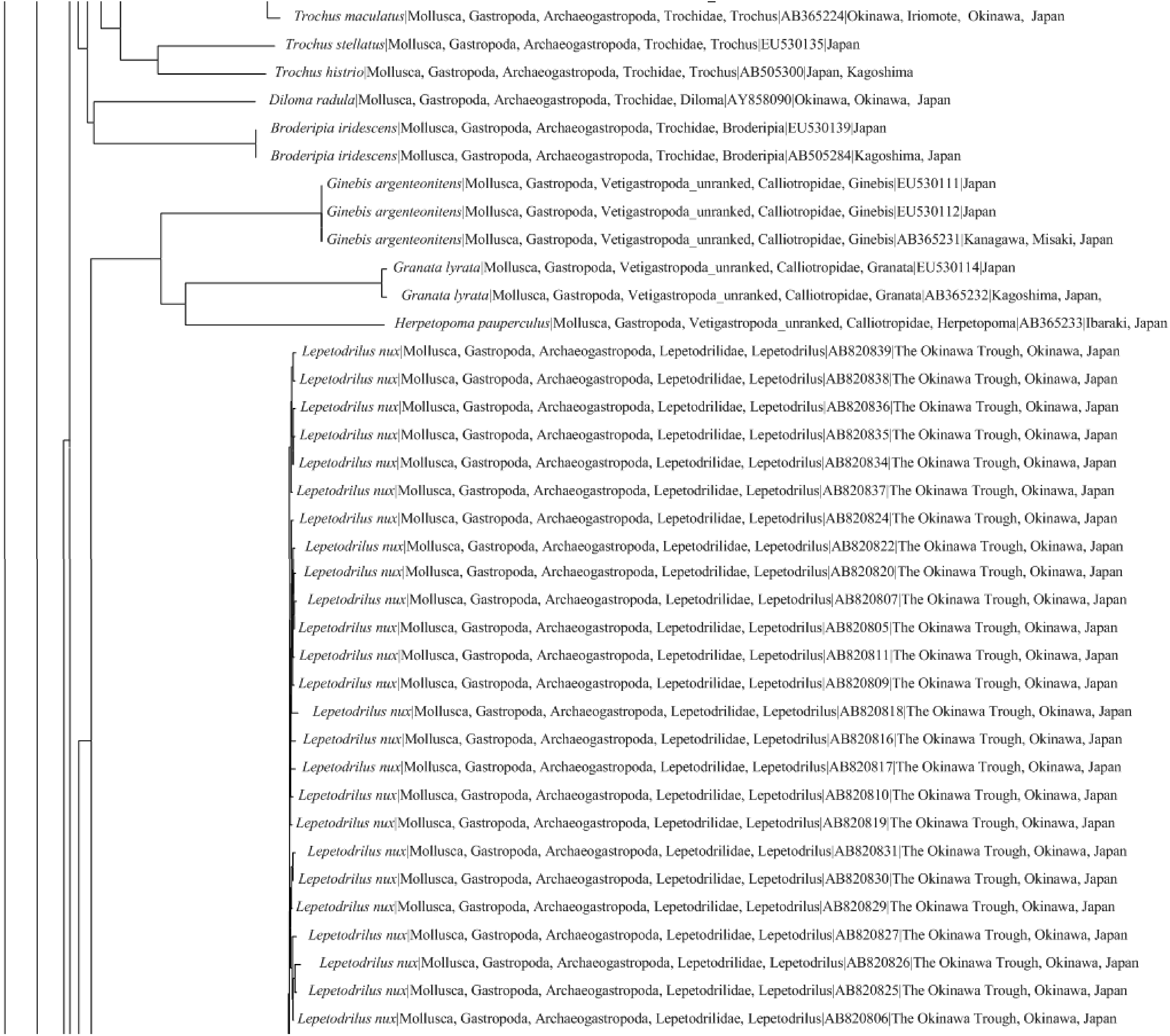
Trochus maculatus|Mollusca, Gastropoda, Archaeogastropoda, Trochidae, Trochus|AB365224|Okinawa, Iriomote, Okinawa, Japan
Trochus stellatus|Mollusca, Gastropoda, Archaeogastropoda, Trochidae, Trochus|EU530135|Japan
Trochus histrio|Mollusca, Gastropoda, Archaeogastropoda, Trochidae, Trochus|AB505300|Japan, Kagoshima
Diloma radula|Mollusca, Gastropoda, Archaeogastropoda, Trochidae, Diloma|AY858090|Okinawa, Okinawa, Japan
Broderipia iridescens|Mollusca, Gastropoda, Archaeogastropoda, Trochidae, Broderipia|EU530139|Japan
Broderipia iridescens|Mollusca, Gastropoda, Archaeogastropoda, Trochidae, Broderipia|AB505284|Kagoshima, Japan
Ginebis argenteonitens|Mollusca, Gastropoda, Vetigastropoda_unranked, Calliotropidae, Ginebis|EU530111|Japan
Ginebis argenteonitens|Mollusca, Gastropoda, Vetigastropoda_unranked, Calliotropidae, Ginebis|EU530112|Japan
Ginebis argenteonitens|Mollusca, Gastropoda, Vetigastropoda_unranked, Calliotropidae, Ginebis|AB365231|Kanagawa, Misaki, Japan
Granata lyrata|Mollusca, Gastropoda, Vetigastropoda_unranked, Calliotropidae, Granata|EU530114|Japan
Granata lyrata|Mollusca, Gastropoda, Vetigastropoda_unranked, Calliotropidae, Granata|AB365232|Kagoshima, Japan,
Herpetopoma pauperculus|Mollusca, Gastropoda, Vetigastropoda_unranked, Calliotropidae, Herpetopoma|AB365233|Ibaraki, Japan
Lepetodrilus nux|Mollusca, Gastropoda, Archaeogastropoda, Lepetodrilidae, Lepetodrilus|AB820839|The Okinawa Trough, Okinawa, Japan
Lepetodrilus nux|Mollusca, Gastropoda, Archaeogastropoda, Lepetodrilidae, Lepetodrilus|AB820838|The Okinawa Trough, Okinawa, Japan
Lepetodrilus nux|Mollusca, Gastropoda, Archaeogastropoda, Lepetodrilidae, Lepetodrilus|AB820836|The Okinawa Trough, Okinawa, Japan
Lepetodrilus nux|Mollusca, Gastropoda, Archaeogastropoda, Lepetodrilidae, Lepetodrilus|AB820835|The Okinawa Trough, Okinawa, Japan
Lepetodrilus nux|Mollusca, Gastropoda, Archaeogastropoda, Lepetodrilidae, Lepetodrilus|AB820834|The Okinawa Trough, Okinawa, Japan
Lepetodrilus nux|Mollusca, Gastropoda, Archaeogastropoda, Lepetodrilidae, Lepetodrilus|AB820837|The Okinawa Trough, Okinawa, Japan
Lepetodrilus nux|Mollusca, Gastropoda, Archaeogastropoda, Lepetodrilidae, Lepetodrilus|AB820824|The Okinawa Trough, Okinawa, Japan
Lepetodrilus nux|Mollusca, Gastropoda, Archaeogastropoda, Lepetodrilidae, Lepetodrilus|AB820822|The Okinawa Trough, Okinawa, Japan
Lepetodrilus nux|Mollusca, Gastropoda, Archaeogastropoda, Lepetodrilidae, Lepetodrilus|AB820820|The Okinawa Trough, Okinawa, Japan
Lepetodrilus nux|Mollusca, Gastropoda, Archaeogastropoda, Lepetodrilidae, Lepetodrilus|AB820807|The Okinawa Trough, Okinawa, Japan
Lepetodrilus nux|Mollusca, Gastropoda, Archaeogastropoda, Lepetodrilidae, Lepetodrilus|AB820805|The Okinawa Trough, Okinawa, Japan
Lepetodrilus nux|Mollusca, Gastropoda, Archaeogastropoda, Lepetodrilidae, Lepetodrilus|AB820811|The Okinawa Trough, Okinawa, Japan
Lepetodrilus nux|Mollusca, Gastropoda, Archaeogastropoda, Lepetodrilidae, Lepetodrilus|AB820809|The Okinawa Trough, Okinawa, Japan
Lepetodrilus nux|Mollusca, Gastropoda, Archaeogastropoda, Lepetodrilidae, Lepetodrilus|AB820818|The Okinawa Trough, Okinawa, Japan
Lepetodrilus nux|Mollusca, Gastropoda, Archaeogastropoda, Lepetodrilidae, Lepetodrilus|AB820816|The Okinawa Trough, Okinawa, Japan
Lepetodrilus nux|Mollusca, Gastropoda, Archaeogastropoda, Lepetodrilidae, Lepetodrilus|AB820817|The Okinawa Trough, Okinawa, Japan
Lepetodrilus nux|Mollusca, Gastropoda, Archaeogastropoda, Lepetodrilidae, Lepetodrilus|AB820810|The Okinawa Trough, Okinawa, Japan
Lepetodrilus nux|Mollusca, Gastropoda, Archaeogastropoda, Lepetodrilidae, Lepetodrilus|AB820819|The Okinawa Trough, Okinawa, Japan
Lepetodrilus nux|Mollusca, Gastropoda, Archaeogastropoda, Lepetodrilidae, Lepetodrilus|AB820831|The Okinawa Trough, Okinawa, Japan
Lepetodrilus nux|Mollusca, Gastropoda, Archaeogastropoda, Lepetodrilidae, Lepetodrilus|AB820830|The Okinawa Trough, Okinawa, Japan
Lepetodrilus nux|Mollusca, Gastropoda, Archaeogastropoda, Lepetodrilidae, Lepetodrilus|AB820829|The Okinawa Trough, Okinawa, Japan
Lepetodrilus nux|Mollusca, Gastropoda, Archaeogastropoda, Lepetodrilidae, Lepetodrilus|AB820827|The Okinawa Trough, Okinawa, Japan
Lepetodrilus nux|Mollusca, Gastropoda, Archaeogastropoda, Lepetodrilidae, Lepetodrilus|AB820826|The Okinawa Trough, Okinawa, Japan
Lepetodrilus nux|Mollusca, Gastropoda, Archaeogastropoda, Lepetodrilidae, Lepetodrilus|AB820825|The Okinawa Trough, Okinawa, Japan
Lepetodrilus nux|Mollusca, Gastropoda, Archaeogastropoda, Lepetodrilidae, Lepetodrilus|AB820806|The Okinawa Trough, Okinawa, Japan

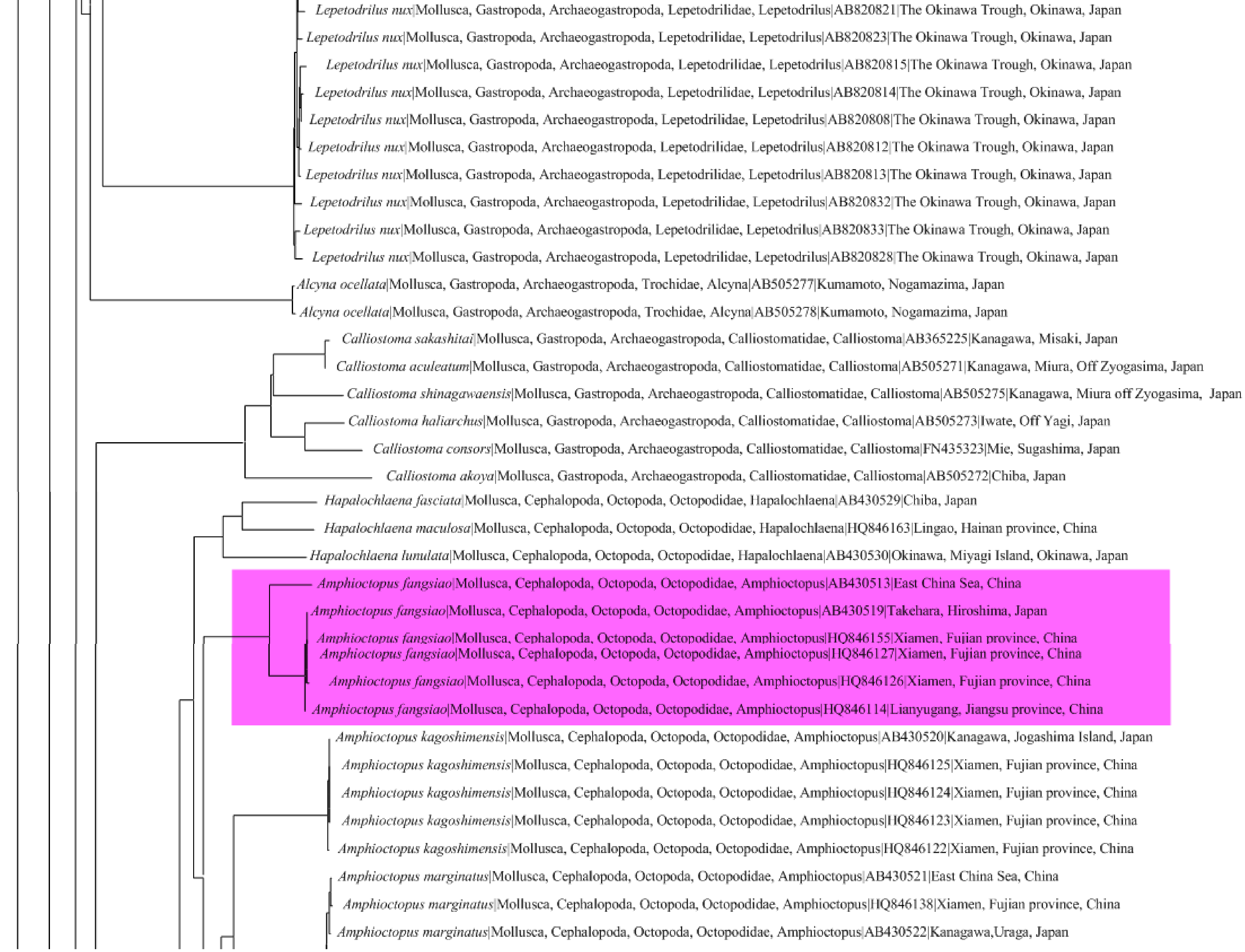

Lepetodrilus nux|Mollusca, Gastropoda, Archaeogastropoda, Lepetodrilidae, Lepetodrilus|AB820821|The Okinawa Trough, Okinawa, Japan
Lepetodrilus nux|Mollusca, Gastropoda, Archaeogastropoda, Lepetodrilidae, Lepetodrilus|AB820823|The Okinawa Trough, Okinawa, Japan
Lepetodrilus nux|Mollusca, Gastropoda, Archaeogastropoda, Lepetodrilidae, Lepetodrilus|AB820815|The Okinawa Trough, Okinawa, Japan
Lepetodrilus nux|Mollusca, Gastropoda, Archaeogastropoda, Lepetodrilidae, Lepetodrilus|AB820814|The Okinawa Trough, Okinawa, Japan
Lepetodrilus nux|Mollusca, Gastropoda, Archaeogastropoda, Lepetodrilidae, Lepetodrilus|AB820808|The Okinawa Trough, Okinawa, Japan
Lepetodrilus nux|Mollusca, Gastropoda, Archaeogastropoda, Lepetodrilidae, Lepetodrilus|AB820812|The Okinawa Trough, Okinawa, Japan
Lepetodrilus nux|Mollusca, Gastropoda, Archaeogastropoda, Lepetodrilidae, Lepetodrilus|AB820813|The Okinawa Trough, Okinawa, Japan
Lepetodrilus nux|Mollusca, Gastropoda, Archaeogastropoda, Lepetodrilidae, Lepetodrilus|AB820832|The Okinawa Trough, Okinawa, Japan
Lepetodrilus nux|Mollusca, Gastropoda, Archaeogastropoda, Lepetodrilidae, Lepetodrilus|AB820833|The Okinawa Trough, Okinawa, Japan
Lepetodrilus nux|Mollusca, Gastropoda, Archaeogastropoda, Lepetodrilidae, Lepetodrilus|AB820828|The Okinawa Trough, Okinawa, Japan
Alcyna ocellata|Mollusca, Gastropoda, Archaeogastropoda, Trochidae, Alcyna|AB505277|Kumamoto, Nogamazima, Japan
Alcyna ocellata|Mollusca, Gastropoda, Archaeogastropoda, Trochidae, Alcyna|AB505278|Kumamoto, Nogamazima, Japan
Calliostoma sakashitai|Mollusca, Gastropoda, Archaeogastropoda, Calliostomatidae, Calliostoma|AB365225|Kanagawa, Misaki, Japan
Calliostoma aculeatum|Mollusca, Gastropoda, Archaeogastropoda, Calliostomatidae, Calliostoma|AB505271|Kanagawa, Miura, Off Zyogasima, Japan
Calliostoma shinagawaensis|Mollusca, Gastropoda, Archaeogastropoda, Calliostomatidae, Calliostoma|AB505275|Kanagawa, Miura off Zyogasima, Japan
Calliostoma haliarchus|Mollusca, Gastropoda, Archaeogastropoda, Calliostomatidae, Calliostoma|AB505273|Iwate, Off Yagi, Japan
Calliostoma consors|Mollusca, Gastropoda, Archaeogastropoda, Calliostomatidae, Calliostoma|FN435323|Mie, Sugashima, Japan
Calliostoma akoya|Mollusca, Gastropoda, Archaeogastropoda, Calliostomatidae, Calliostoma|AB505272|Chiba, Japan
Hapalochlaena fasciata|Mollusca, Cephalopoda, Octopoda, Octopodidae, Hapalochlaena|AB430529|Chiba, Japan
Hapalochlaena maculosa|Mollusca, Cephalopoda, Octopoda, Octopodidae, Hapalochlaena|HQ846163|Lingao, Hainan province, China
Hapalochlaena lunulata|Mollusca, Cephalopoda, Octopoda, Octopodidae, Hapalochlaena|AB430530|Okinawa, Miyagi Island, Okinawa, Japan
Amphioctopus fangsiao|Mollusca, Cephalopoda, Octopoda, Octopodidae, Amphioctopus|AB430513|East China Sea, China
Amphioctopus fangsiao|Mollusca, Cephalopoda, Octopoda, Octopodidae, Amphioctopus|AB430519|Takehara, Hiroshima, Japan
Amphioctopus fangsiao|Mollusca, Cephalopoda, Octopoda, Octopodidae, Amphioctopus|HQ846155|Xiamen, Fujian province, China
Amphioctopus fangsiao|Mollusca, Cephalopoda, Octopoda, Octopodidae, Amphioctopus|HQ846127|Xiamen, Fujian province, China
Amphioctopus fangsiao|Mollusca, Cephalopoda, Octopoda, Octopodidae, Amphioctopus|HQ846126|Xiamen, Fujian province, China
Amphioctopus fangsiao|Mollusca, Cephalopoda, Octopoda, Octopodidae, Amphioctopus|HQ846114|Lianyugang, Jiangsu province, China
Amphioctopus kagoshimensis|Mollusca, Cephalopoda, Octopoda, Octopodidae, Amphioctopus|AB430520|Kanagawa, Jogashima Island, Japan
Amphioctopus kagoshimensis|Mollusca, Cephalopoda, Octopoda, Octopodidae, Amphioctopus|HQ846125|Xiamen, Fujian province, China
Amphioctopus kagoshimensis|Mollusca, Cephalopoda, Octopoda, Octopodidae, Amphioctopus|HQ846124|Xiamen, Fujian province, China
Amphioctopus kagoshimensis|Mollusca, Cephalopoda, Octopoda, Octopodidae, Amphioctopus|HQ846123|Xiamen, Fujian province, China
Amphioctopus kagoshimensis|Mollusca, Cephalopoda, Octopoda, Octopodidae, Amphioctopus|HQ846122|Xiamen, Fujian province, China
Amphioctopus marginatus|Mollusca, Cephalopoda, Octopoda, Octopodidae, Amphioctopus|AB430521|East China Sea, China
Amphioctopus marginatus|Mollusca, Cephalopoda, Octopoda, Octopodidae, Amphioctopus|HQ846138|Xiamen, Fujian province, China
Amphioctopus marginatus|Mollusca, Cephalopoda, Octopoda, Octopodidae, Amphioctopus|AB430522|Kanagawa,Uraga, Japan

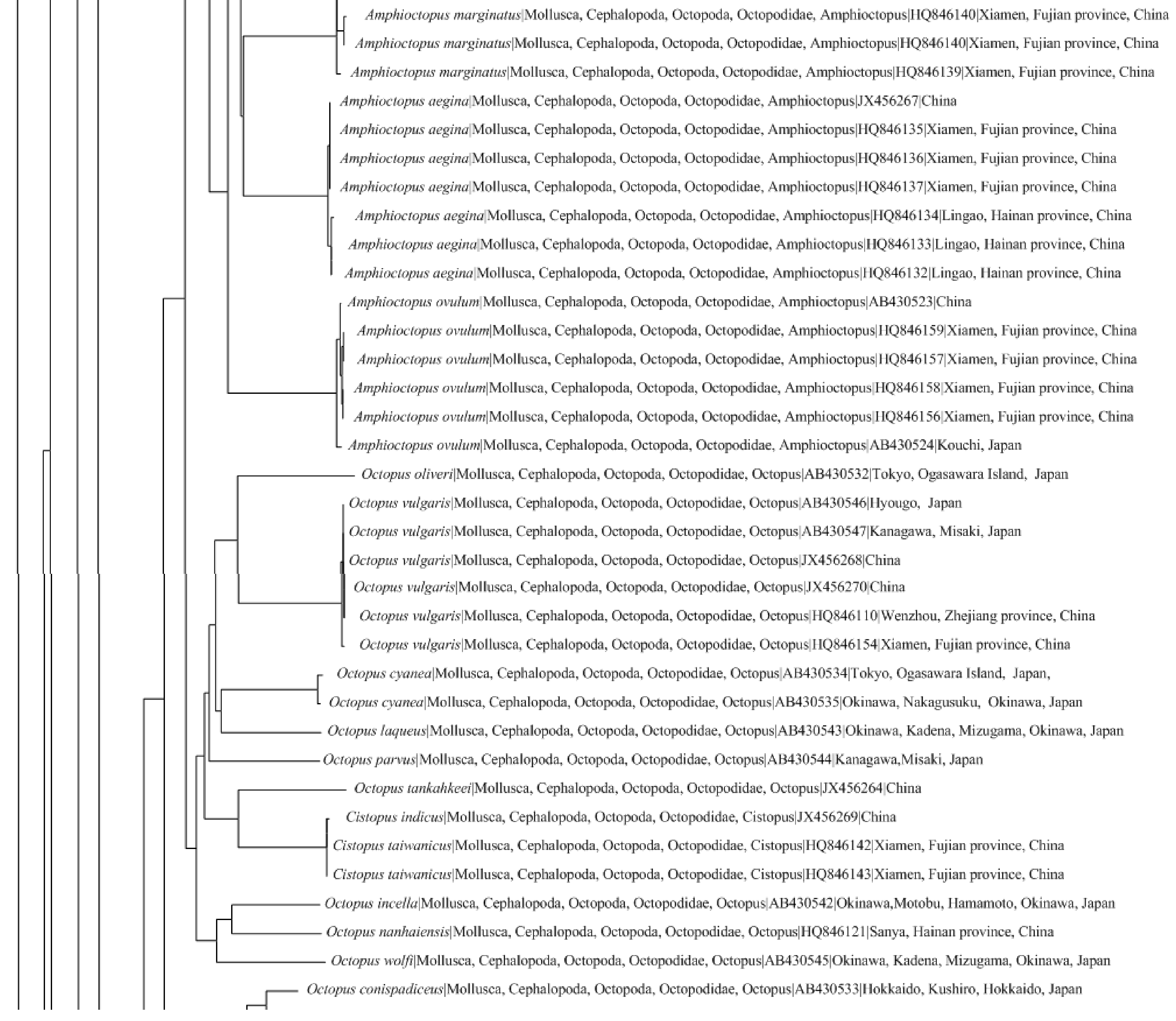
Amphioctopus marginatus|Mollusca, Cephalopoda, Octopoda, Octopodidae, Amphioctopus|HQ846140|Xiamen, Fujian province, China
Amphioctopus marginatus|Mollusca, Cephalopoda, Octopoda, Octopodidae, Amphioctopus|HQ846140|Xiamen, Fujian province, China
Amphioctopus marginatus|Mollusca, Cephalopoda, Octopoda, Octopodidae, Amphioctopus|HQ846139|Xiamen, Fujian province, China
Amphioctopus aegina|Mollusca, Cephalopoda, Octopoda, Octopodidae, Amphioctopus|JX456267|China
Amphioctopus aegina|Mollusca, Cephalopoda, Octopoda, Octopodidae, Amphioctopus|HQ846135|Xiamen, Fujian province, China
Amphioctopus aegina|Mollusca, Cephalopoda, Octopoda, Octopodidae, Amphioctopus|HQ846136|Xiamen, Fujian province, China
Amphioctopus aegina|Mollusca, Cephalopoda, Octopoda, Octopodidae, Amphioctopus|HQ846137|Xiamen, Fujian province, China
Amphioctopus aegina|Mollusca, Cephalopoda, Octopoda, Octopodidae, Amphioctopus|HQ846134|Lingao, Hainan province, China
Amphioctopus aegina|Mollusca, Cephalopoda, Octopoda, Octopodidae, Amphioctopus|HQ846133|Lingao, Hainan province, China
Amphioctopus aegina|Mollusca, Cephalopoda, Octopoda, Octopodidae, Amphioctopus|HQ846132|Lingao, Hainan province, China
Amphioctopus ovulum|Mollusca, Cephalopoda, Octopoda, Octopodidae, Amphioctopus|AB430523|China
Amphioctopus ovulum|Mollusca, Cephalopoda, Octopoda, Octopodidae, Amphioctopus|HQ846159|Xiamen, Fujian province, China
Amphioctopus ovulum|Mollusca, Cephalopoda, Octopoda, Octopodidae, Amphioctopus|HQ846157|Xiamen, Fujian province, China
Amphioctopus ovulum|Mollusca, Cephalopoda, Octopoda, Octopodidae, Amphioctopus|HQ846158|Xiamen, Fujian province, China
Amphioctopus ovulum|Mollusca, Cephalopoda, Octopoda, Octopodidae, Amphioctopus|HQ846156|Xiamen, Fujian province, China
Amphioctopus ovulum|Mollusca, Cephalopoda, Octopoda, Octopodidae, Amphioctopus|AB430524|Kouchi, Japan
Octopus oliveri|Mollusca, Cephalopoda, Octopoda, Octopodidae, Octopus|AB430532|Tokyo, Ogasawara Island, Japan
Octopus vulgaris|Mollusca, Cephalopoda, Octopoda, Octopodidae, Octopus|AB430546|Hyougo, Japan
Octopus vulgaris|Mollusca, Cephalopoda, Octopoda, Octopodidae, Octopus|AB430547|Kanagawa, Misaki, Japan
Octopus vulgaris|Mollusca, Cephalopoda, Octopoda, Octopodidae, Octopus|JX456268|China
Octopus vulgaris|Mollusca, Cephalopoda, Octopoda, Octopodidae, Octopus|JX456270|China
Octopus vulgaris|Mollusca, Cephalopoda, Octopoda, Octopodidae, Octopus|HQ846110|Wenzhou, Zhejiang province, China
Octopus vulgaris|Mollusca, Cephalopoda, Octopoda, Octopodidae, Octopus|HQ846154|Xiamen, Fujian province, China
Octopus cyanea|Mollusca, Cephalopoda, Octopoda, Octopodidae, Octopus|AB430534|Tokyo, Ogasawara Island, Japan,
Octopus cyanea|Mollusca, Cephalopoda, Octopoda, Octopodidae, Octopus|AB430535|Okinawa, Nakagusuku, Okinawa, Japan
Octopus laqueus|Mollusca, Cephalopoda, Octopoda, Octopodidae, Octopus|AB430543|Okinawa, Kadena, Mizugama, Okinawa, Japan
Octopus parvus|Mollusca, Cephalopoda, Octopoda, Octopodidae, Octopus|AB430544|Kanagawa,Misaki, Japan
Octopus tankahkeei|Mollusca, Cephalopoda, Octopoda, Octopodidae, Octopus|JX456264|China
Cistopus indicus|Mollusca, Cephalopoda, Octopoda, Octopodidae, Cistopus|JX456269|China
Cistopus taiwanicus|Mollusca, Cephalopoda, Octopoda, Octopodidae, Cistopus|HQ846142|Xiamen, Fujian province, China
Cistopus taiwanicus|Mollusca, Cephalopoda, Octopoda, Octopodidae, Cistopus|HQ846143|Xiamen, Fujian province, China
Octopus incella|Mollusca, Cephalopoda, Octopoda, Octopodidae, Octopus|AB430542|Okinawa,Motobu, Hamamoto, Okinawa, Japan
Octopus nanhaiensis|Mollusca, Cephalopoda, Octopoda, Octopodidae, Octopus|HQ846121|Sanya, Hainan province, China
Octopus wolfi|Mollusca, Cephalopoda, Octopoda, Octopodidae, Octopus|AB430545|Okinawa, Kadena, Mizugama, Okinawa, Japan
Octopus conispadiceus|Mollusca, Cephalopoda, Octopoda, Octopodidae, Octopus|AB430533|Hokkaido, Kushiro, Hokkaido, Japan

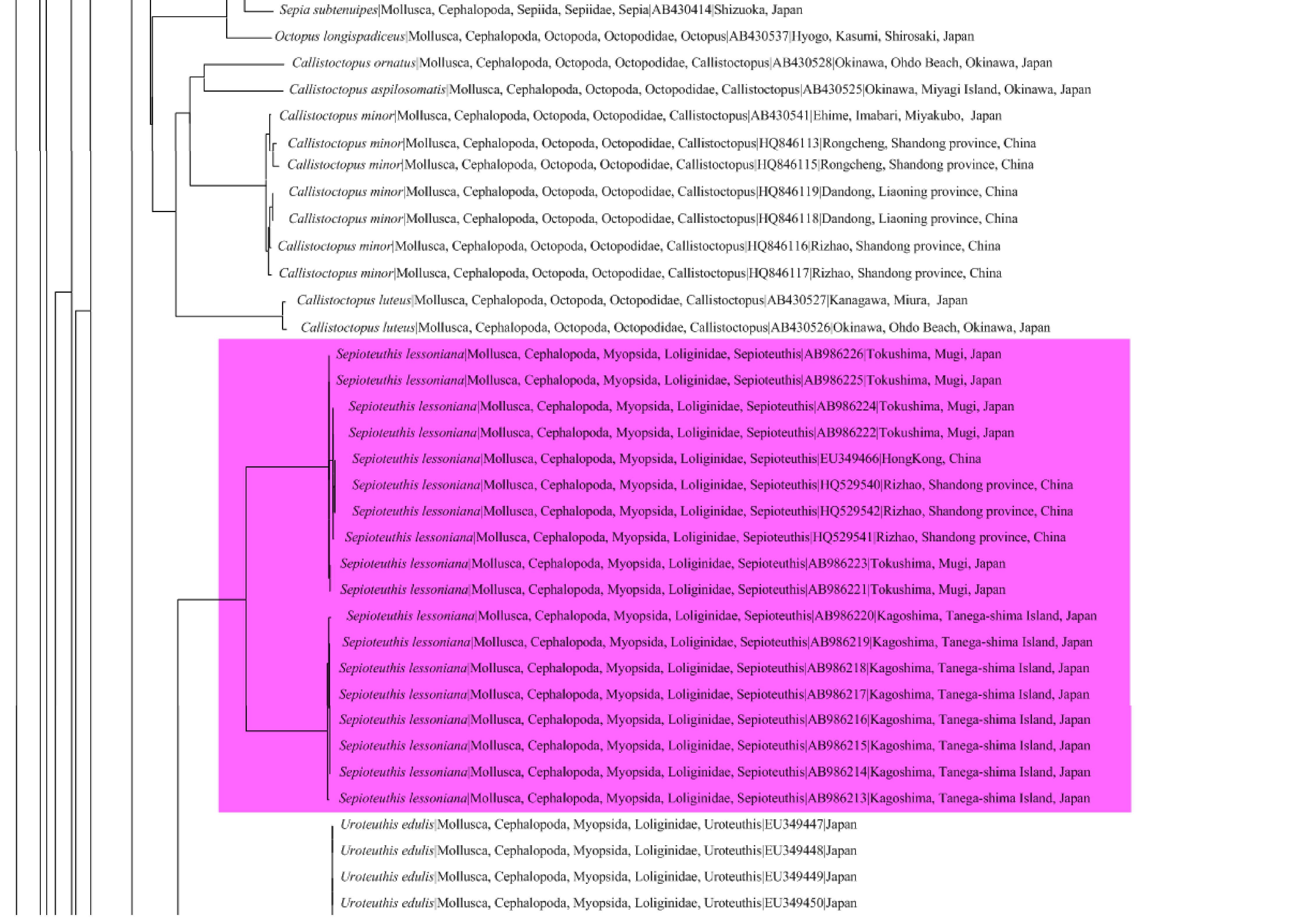

Sepia subtenuipes|Mollusca, Cephalopoda, Sepiida, Sepiidae, Sepia|AB430414|Shizuoka, Japan
Octopus longispadiceus|Mollusca, Cephalopoda, Octopoda, Octopodidae, Octopus|AB430537|Hyogo, Kasumi, Shirosaki, Japan
Callistoctopus ornatus|Mollusca, Cephalopoda, Octopoda, Octopodidae, Callistoctopus|AB430528|Okinawa, Ohdo Beach, Okinawa, Japan
Callistoctopus aspilosomatis|Mollusca, Cephalopoda, Octopoda, Octopodidae, Callistoctopus|AB430525|Okinawa, Miyagi Island, Okinawa, Japan
Callistoctopus minor|Mollusca, Cephalopoda, Octopoda, Octopodidae, Callistoctopus|AB430541|Ehime, Imabari, Miyakubo, Japan
Callistoctopus minor|Mollusca, Cephalopoda, Octopoda, Octopodidae, Callistoctopus|HQ846113|Rongcheng, Shandong province, China
Callistoctopus minor|Mollusca, Cephalopoda, Octopoda, Octopodidae, Callistoctopus|HQ846115|Rongcheng, Shandong province, China
Callistoctopus minor|Mollusca, Cephalopoda, Octopoda, Octopodidae, Callistoctopus|HQ846119|Dandong, Liaoning province, China
Callistoctopus minor|Mollusca, Cephalopoda, Octopoda, Octopodidae, Callistoctopus|HQ846118|Dandong, Liaoning province, China
Callistoctopus minor|Mollusca, Cephalopoda, Octopoda, Octopodidae, Callistoctopus|HQ846116|Rizhao, Shandong province, China
Callistoctopus minor|Mollusca, Cephalopoda, Octopoda, Octopodidae, Callistoctopus|HQ846117|Rizhao, Shandong province, China
Callistoctopus luteus|Mollusca, Cephalopoda, Octopoda, Octopodidae, Callistoctopus|AB430527|Kanagawa, Miura, Japan
Callistoctopus luteus|Mollusca, Cephalopoda, Octopoda, Octopodidae, Callistoctopus|AB430526|Okinawa, Ohdo Beach, Okinawa, Japan
Sepioteuthis lessoniana|Mollusca, Cephalopoda, Myopsida, Loliginidae, Sepioteuthis|AB986226|Tokushima, Mugi, Japan
Sepioteuthis lessoniana|Mollusca, Cephalopoda, Myopsida, Loliginidae, Sepioteuthis|AB986225|Tokushima, Mugi, Japan
Sepioteuthis lessoniana|Mollusca, Cephalopoda, Myopsida, Loliginidae, Sepioteuthis|AB986224|Tokushima, Mugi, Japan
Sepioteuthis lessoniana|Mollusca, Cephalopoda, Myopsida, Loliginidae, Sepioteuthis|AB986222|Tokushima, Mugi, Japan
Sepioteuthis lessoniana|Mollusca, Cephalopoda, Myopsida, Loliginidae, Sepioteuthis|EU349466|HongKong, China
Sepioteuthis lessoniana|Mollusca, Cephalopoda, Myopsida, Loliginidae, Sepioteuthis|HQ529540|Rizhao, Shandong province, China
Sepioteuthis lessoniana|Mollusca, Cephalopoda, Myopsida, Loliginidae, Sepioteuthis|HQ529542|Rizhao, Shandong province, China
Sepioteuthis lessoniana|Mollusca, Cephalopoda, Myopsida, Loliginidae, Sepioteuthis|HQ529541|Rizhao, Shandong province, China
Sepioteuthis lessoniana|Mollusca, Cephalopoda, Myopsida, Loliginidae, Sepioteuthis|AB986223|Tokushima, Mugi, Japan
Sepioteuthis lessoniana|Mollusca, Cephalopoda, Myopsida, Loliginidae, Sepioteuthis|AB986221|Tokushima, Mugi, Japan
Sepioteuthis lessoniana|Mollusca, Cephalopoda, Myopsida, Loliginidae, Sepioteuthis|AB986220|Kagoshima, Tanega-shima Island, Japan
Sepioteuthis lessoniana|Mollusca, Cephalopoda, Myopsida, Loliginidae, Sepioteuthis|AB986219|Kagoshima, Tanega-shima Island, Japan
Sepioteuthis lessoniana|Mollusca, Cephalopoda, Myopsida, Loliginidae, Sepioteuthis|AB986218|Kagoshima, Tanega-shima Island, Japan
Sepioteuthis lessoniana|Mollusca, Cephalopoda, Myopsida, Loliginidae, Sepioteuthis|AB986217|Kagoshima, Tanega-shima Island, Japan
Sepioteuthis lessoniana|Mollusca, Cephalopoda, Myopsida, Loliginidae, Sepioteuthis|AB986216|Kagoshima, Tanega-shima Island, Japan
Sepioteuthis lessoniana|Mollusca, Cephalopoda, Myopsida, Loliginidae, Sepioteuthis|AB986215|Kagoshima, Tanega-shima Island, Japan
Sepioteuthis lessoniana|Mollusca, Cephalopoda, Myopsida, Loliginidae, Sepioteuthis|AB986214|Kagoshima, Tanega-shima Island, Japan
Sepioteuthis lessoniana|Mollusca, Cephalopoda, Myopsida, Loliginidae, Sepioteuthis|AB986213|Kagoshima, Tanega-shima Island, Japan
Uroteuthis edulis|Mollusca, Cephalopoda, Myopsida, Loliginidae, Uroteuthis|EU349447|Japan
Uroteuthis edulis|Mollusca, Cephalopoda, Myopsida, Loliginidae, Uroteuthis|EU349448|Japan
Uroteuthis edulis|Mollusca, Cephalopoda, Myopsida, Loliginidae, Uroteuthis|EU349449|Japan
Uroteuthis edulis|Mollusca, Cephalopoda, Myopsida, Loliginidae, Uroteuthis|EU349450|Japan

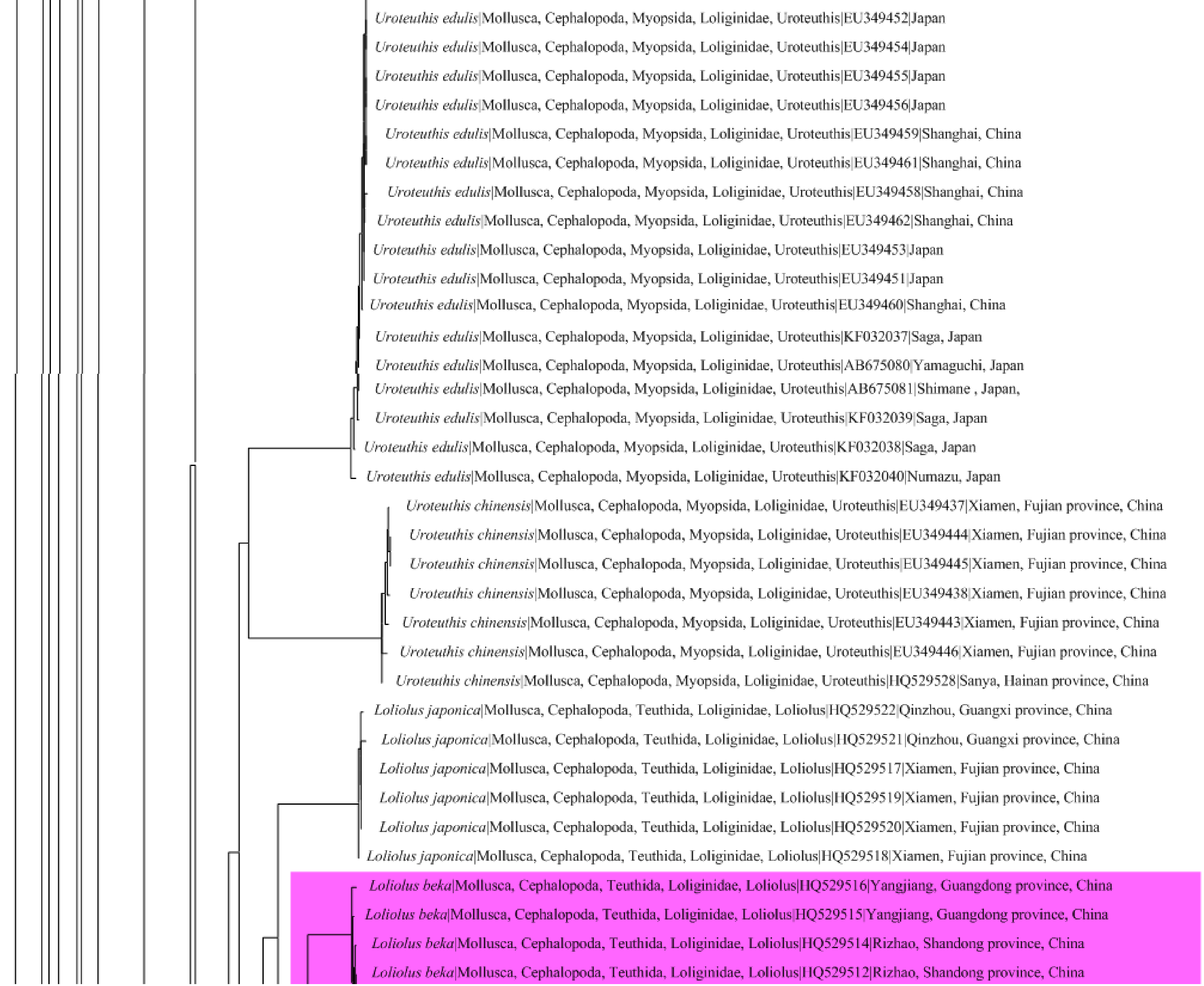
Uroteuthis edulis|Mollusca, Cephalopoda, Myopsida, Loliginidae, Uroteuthis|EU349452|Japan
Uroteuthis edulis|Mollusca, Cephalopoda, Myopsida, Loliginidae, Uroteuthis|EU349454|Japan
Uroteuthis edulis|Mollusca, Cephalopoda, Myopsida, Loliginidae, Uroteuthis|EU349455|Japan
Uroteuthis edulis|Mollusca, Cephalopoda, Myopsida, Loliginidae, Uroteuthis|EU349456|Japan
Uroteuthis edulis|Mollusca, Cephalopoda, Myopsida, Loliginidae, Uroteuthis|EU349459|Shanghai, China
Uroteuthis edulis|Mollusca, Cephalopoda, Myopsida, Loliginidae, Uroteuthis|EU349461|Shanghai, China
Uroteuthis edulis|Mollusca, Cephalopoda, Myopsida, Loliginidae, Uroteuthis|EU349458|Shanghai, China
Uroteuthis edulis|Mollusca, Cephalopoda, Myopsida, Loliginidae, Uroteuthis|EU349462|Shanghai, China
Uroteuthis edulis|Mollusca, Cephalopoda, Myopsida, Loliginidae, Uroteuthis|EU349453|Japan
Uroteuthis edulis|Mollusca, Cephalopoda, Myopsida, Loliginidae, Uroteuthis|EU349451|Japan
Uroteuthis edulis|Mollusca, Cephalopoda, Myopsida, Loliginidae, Uroteuthis|EU349460|Shanghai, China
Uroteuthis edulis|Mollusca, Cephalopoda, Myopsida, Loliginidae, Uroteuthis|KF032037|Saga, Japan
Uroteuthis edulis|Mollusca, Cephalopoda, Myopsida, Loliginidae, Uroteuthis|AB675080|Yamaguchi, Japan
Uroteuthis edulis|Mollusca, Cephalopoda, Myopsida, Loliginidae, Uroteuthis|AB675081|Shimane , Japan,
Uroteuthis edulis|Mollusca, Cephalopoda, Myopsida, Loliginidae, Uroteuthis|KF032039|Saga, Japan
Uroteuthis edulis|Mollusca, Cephalopoda, Myopsida, Loliginidae, Uroteuthis|KF032038|Saga, Japan
Uroteuthis edulis|Mollusca, Cephalopoda, Myopsida, Loliginidae, Uroteuthis|KF032040|Numazu, Japan
Uroteuthis chinensis|Mollusca, Cephalopoda, Myopsida, Loliginidae, Uroteuthis|EU349437|Xiamen, Fujian province, China
Uroteuthis chinensis|Mollusca, Cephalopoda, Myopsida, Loliginidae, Uroteuthis|EU349444|Xiamen, Fujian province, China
Uroteuthis chinensis|Mollusca, Cephalopoda, Myopsida, Loliginidae, Uroteuthis|EU349445|Xiamen, Fujian province, China
Uroteuthis chinensis|Mollusca, Cephalopoda, Myopsida, Loliginidae, Uroteuthis|EU349438|Xiamen, Fujian province, China
Uroteuthis chinensis|Mollusca, Cephalopoda, Myopsida, Loliginidae, Uroteuthis|EU349443|Xiamen, Fujian province, China
Uroteuthis chinensis|Mollusca, Cephalopoda, Myopsida, Loliginidae, Uroteuthis|EU349446|Xiamen, Fujian province, China
Uroteuthis chinensis|Mollusca, Cephalopoda, Myopsida, Loliginidae, Uroteuthis|HQ529528|Sanya, Hainan province, China
Loliolus japonica|Mollusca, Cephalopoda, Teuthida, Loliginidae, Loliolus|HQ529522|Qinzhou, Guangxi province, China
Loliolus japonica|Mollusca, Cephalopoda, Teuthida, Loliginidae, Loliolus|HQ529521|Qinzhou, Guangxi province, China
Loliolus japonica|Mollusca, Cephalopoda, Teuthida, Loliginidae, Loliolus|HQ529517|Xiamen, Fujian province, China
Loliolus japonica|Mollusca, Cephalopoda, Teuthida, Loliginidae, Loliolus|HQ529519|Xiamen, Fujian province, China
Loliolus japonica|Mollusca, Cephalopoda, Teuthida, Loliginidae, Loliolus|HQ529520|Xiamen, Fujian province, China
Loliolus japonica|Mollusca, Cephalopoda, Teuthida, Loliginidae, Loliolus|HQ529518|Xiamen, Fujian province, China
Loliolus beka|Mollusca, Cephalopoda, Teuthida, Loliginidae, Loliolus|HQ529516|Yangjiang, Guangdong province, China
Loliolus beka|Mollusca, Cephalopoda, Teuthida, Loliginidae, Loliolus|HQ529515|Yangjiang, Guangdong province, China
Loliolus beka|Mollusca, Cephalopoda, Teuthida, Loliginidae, Loliolus|HQ529514|Rizhao, Shandong province, China
Loliolus beka|Mollusca, Cephalopoda, Teuthida, Loliginidae, Loliolus|HQ529512|Rizhao, Shandong province, China

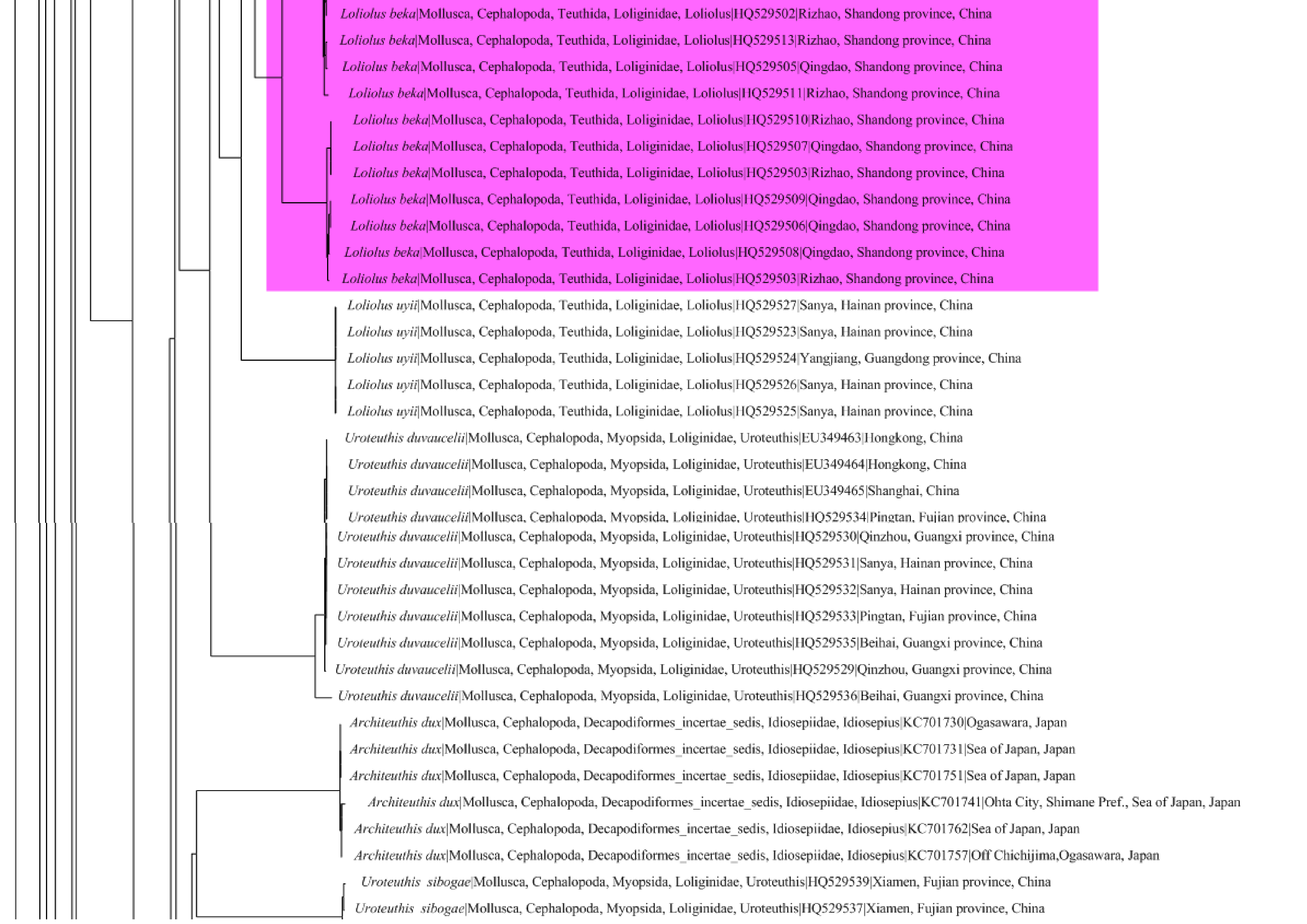

Loliolus beka|Mollusca, Cephalopoda, Teuthida, Loliginidae, Loliolus|HQ529502|Rizhao, Shandong province, China
Loliolus beka|Mollusca, Cephalopoda, Teuthida, Loliginidae, Loliolus|HQ529513|Rizhao, Shandong province, China
Loliolus beka|Mollusca, Cephalopoda, Teuthida, Loliginidae, Loliolus|HQ529505|Qingdao, Shandong province, China
Loliolus beka|Mollusca, Cephalopoda, Teuthida, Loliginidae, Loliolus|HQ529511|Rizhao, Shandong province, China
Loliolus beka|Mollusca, Cephalopoda, Teuthida, Loliginidae, Loliolus|HQ529510|Rizhao, Shandong province, China
Loliolus beka|Mollusca, Cephalopoda, Teuthida, Loliginidae, Loliolus|HQ529507|Qingdao, Shandong province, China
Loliolus beka|Mollusca, Cephalopoda, Teuthida, Loliginidae, Loliolus|HQ529503|Rizhao, Shandong province, China
Loliolus beka|Mollusca, Cephalopoda, Teuthida, Loliginidae, Loliolus|HQ529509|Qingdao, Shandong province, China
Loliolus beka|Mollusca, Cephalopoda, Teuthida, Loliginidae, Loliolus|HQ529506|Qingdao, Shandong province, China
Loliolus beka|Mollusca, Cephalopoda, Teuthida, Loliginidae, Loliolus|HQ529508|Qingdao, Shandong province, China
Loliolus beka|Mollusca, Cephalopoda, Teuthida, Loliginidae, Loliolus|HQ529503|Rizhao, Shandong province, China
Loliolus uyii|Mollusca, Cephalopoda, Teuthida, Loliginidae, Loliolus|HQ529527|Sanya, Hainan province, China
Loliolus uyii|Mollusca, Cephalopoda, Teuthida, Loliginidae, Loliolus|HQ529523|Sanya, Hainan province, China
Loliolus uyii|Mollusca, Cephalopoda, Teuthida, Loliginidae, Loliolus|HQ529524|Yangjiang, Guangdong province, China
Loliolus uyii|Mollusca, Cephalopoda, Teuthida, Loliginidae, Loliolus|HQ529526|Sanya, Hainan province, China
Loliolus uyii|Mollusca, Cephalopoda, Teuthida, Loliginidae, Loliolus|HQ529525|Sanya, Hainan province, China
Uroteuthis duvaucelii|Mollusca, Cephalopoda, Myopsida, Loliginidae, Uroteuthis|EU349463|Hongkong, China
Uroteuthis duvaucelii|Mollusca, Cephalopoda, Myopsida, Loliginidae, Uroteuthis|EU349464|Hongkong, China
Uroteuthis duvaucelii|Mollusca, Cephalopoda, Myopsida, Loliginidae, Uroteuthis|EU349465|Shanghai, China
Uroteuthis duvaucelii|Mollusca, Cephalopoda, Myopsida, Loliginidae, Uroteuthis|HQ529534|Pingtan, Fujian province, China
Uroteuthis duvaucelii|Mollusca, Cephalopoda, Myopsida, Loliginidae, Uroteuthis|HQ529530|Qinzhou, Guangxi province, China
Uroteuthis duvaucelii|Mollusca, Cephalopoda, Myopsida, Loliginidae, Uroteuthis|HQ529531|Sanya, Hainan province, China
Uroteuthis duvaucelii|Mollusca, Cephalopoda, Myopsida, Loliginidae, Uroteuthis|HQ529532|Sanya, Hainan province, China
Uroteuthis duvaucelii|Mollusca, Cephalopoda, Myopsida, Loliginidae, Uroteuthis|HQ529533|Pingtan, Fujian province, China
Uroteuthis duvaucelii|Mollusca, Cephalopoda, Myopsida, Loliginidae, Uroteuthis|HQ529535|Beihai, Guangxi province, China
Uroteuthis duvaucelii|Mollusca, Cephalopoda, Myopsida, Loliginidae, Uroteuthis|HQ529529|Qinzhou, Guangxi province, China
Uroteuthis duvaucelii|Mollusca, Cephalopoda, Myopsida, Loliginidae, Uroteuthis|HQ529536|Beihai, Guangxi province, China
Architeuthis dux|Mollusca, Cephalopoda, Decapodiformes_incertae_sedis, Idiosepiidae, Idiosepius|KC701730|Ogasawara, Japan
Architeuthis dux|Mollusca, Cephalopoda, Decapodiformes_incertae_sedis, Idiosepiidae, Idiosepius|KC701731|Sea of Japan, Japan
Architeuthis dux|Mollusca, Cephalopoda, Decapodiformes_incertae_sedis, Idiosepiidae, Idiosepius|KC701751|Sea of Japan, Japan
Architeuthis dux|Mollusca, Cephalopoda, Decapodiformes_incertae_sedis, Idiosepiidae, Idiosepius|KC701741|Ohta City, Shimane Pref., Sea of Japan, Japan
Architeuthis dux|Mollusca, Cephalopoda, Decapodiformes_incertae_sedis, Idiosepiidae, Idiosepius|KC701762|Sea of Japan, Japan
Architeuthis dux|Mollusca, Cephalopoda, Decapodiformes_incertae_sedis, Idiosepiidae, Idiosepius|KC701757|Off Chichijima,Ogasawara, Japan
Uroteuthis sibogae|Mollusca, Cephalopoda, Myopsida, Loliginidae, Uroteuthis|HQ529539|Xiamen, Fujian province, China
Uroteuthis sibogae|Mollusca, Cephalopoda, Myopsida, Loliginidae, Uroteuthis|HQ529537|Xiamen, Fujian province, China

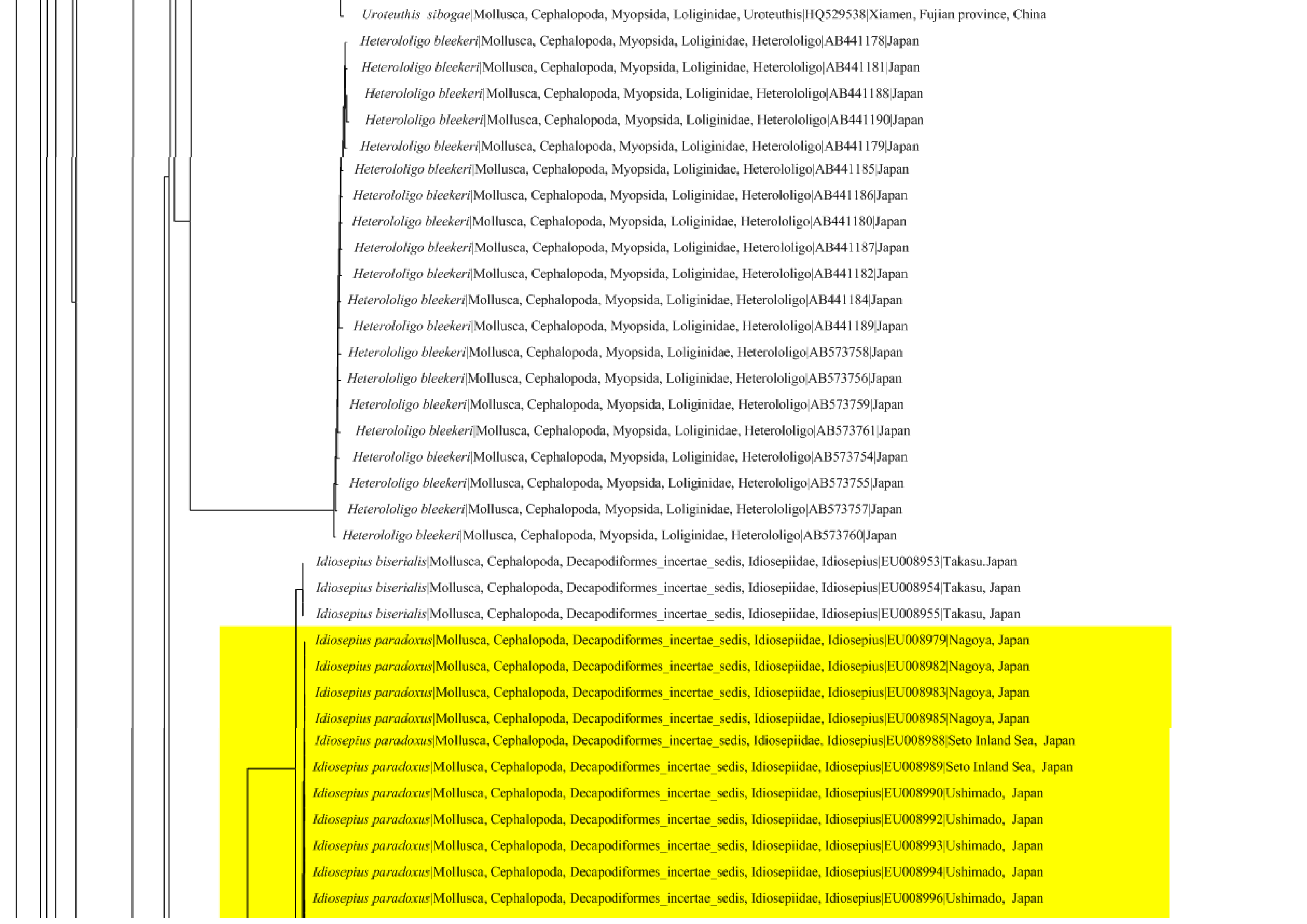

Uroteuthis sibogae|Mollusca, Cephalopoda, Myopsida, Loliginidae, Uroteuthis|HQ529538|Xiamen, Fujian province, China
Heterololigo bleekeri|Mollusca, Cephalopoda, Myopsida, Loliginidae, Heterololigo|AB441178|Japan
Heterololigo bleekeri|Mollusca, Cephalopoda, Myopsida, Loliginidae, Heterololigo|AB441181|Japan
Heterololigo bleekeri|Mollusca, Cephalopoda, Myopsida, Loliginidae, Heterololigo|AB441188|Japan
Heterololigo bleekeri|Mollusca, Cephalopoda, Myopsida, Loliginidae, Heterololigo|AB441190|Japan
Heterololigo bleekeri|Mollusca, Cephalopoda, Myopsida, Loliginidae, Heterololigo|AB441179|Japan
Heterololigo bleekeri|Mollusca, Cephalopoda, Myopsida, Loliginidae, Heterololigo|AB441185|Japan
Heterololigo bleekeri|Mollusca, Cephalopoda, Myopsida, Loliginidae, Heterololigo|AB441186|Japan
Heterololigo bleekeri|Mollusca, Cephalopoda, Myopsida, Loliginidae, Heterololigo|AB441180|Japan
Heterololigo bleekeri|Mollusca, Cephalopoda, Myopsida, Loliginidae, Heterololigo|AB441187|Japan
Heterololigo bleekeri|Mollusca, Cephalopoda, Myopsida, Loliginidae, Heterololigo|AB441182|Japan
Heterololigo bleekeri|Mollusca, Cephalopoda, Myopsida, Loliginidae, Heterololigo|AB441184|Japan
Heterololigo bleekeri|Mollusca, Cephalopoda, Myopsida, Loliginidae, Heterololigo|AB441189|Japan
Heterololigo bleekeri|Mollusca, Cephalopoda, Myopsida, Loliginidae, Heterololigo|AB573758|Japan
Heterololigo bleekeri|Mollusca, Cephalopoda, Myopsida, Loliginidae, Heterololigo|AB573756|Japan
Heterololigo bleekeri|Mollusca, Cephalopoda, Myopsida, Loliginidae, Heterololigo|AB573759|Japan
Heterololigo bleekeri|Mollusca, Cephalopoda, Myopsida, Loliginidae, Heterololigo|AB573761|Japan
Heterololigo bleekeri|Mollusca, Cephalopoda, Myopsida, Loliginidae, Heterololigo|AB573754|Japan
Heterololigo bleekeri|Mollusca, Cephalopoda, Myopsida, Loliginidae, Heterololigo|AB573755|Japan
Heterololigo bleekeri|Mollusca, Cephalopoda, Myopsida, Loliginidae, Heterololigo|AB573757|Japan
Heterololigo bleekeri|Mollusca, Cephalopoda, Myopsida, Loliginidae, Heterololigo|AB573760|Japan
Idiosepius biserialis|Mollusca, Cephalopoda, Decapodiformes_incertae_sedis, Idiosepiidae, Idiosepius|EU008953|Takasu.Japan
Idiosepius biserialis|Mollusca, Cephalopoda, Decapodiformes_incertae_sedis, Idiosepiidae, Idiosepius|EU008954|Takasu, Japan
Idiosepius biserialis|Mollusca, Cephalopoda, Decapodiformes_incertae_sedis, Idiosepiidae, Idiosepius|EU008955|Takasu, Japan
Idiosepius paradoxus|Mollusca, Cephalopoda, Decapodiformes_incertae_sedis, Idiosepiidae, Idiosepius|EU008979|Nagoya, Japan
Idiosepius paradoxus|Mollusca, Cephalopoda, Decapodiformes_incertae_sedis, Idiosepiidae, Idiosepius|EU008982|Nagoya, Japan
Idiosepius paradoxus|Mollusca, Cephalopoda, Decapodiformes_incertae_sedis, Idiosepiidae, Idiosepius|EU008983|Nagoya, Japan
Idiosepius paradoxus|Mollusca, Cephalopoda, Decapodiformes_incertae_sedis, Idiosepiidae, Idiosepius|EU008985|Nagoya, Japan
Idiosepius paradoxus|Mollusca, Cephalopoda, Decapodiformes_incertae_sedis, Idiosepiidae, Idiosepius|EU008988|Seto Inland Sea, Japan
Idiosepius paradoxus|Mollusca, Cephalopoda, Decapodiformes_incertae_sedis, Idiosepiidae, Idiosepius|EU008989|Seto Inland Sea, Japan
Idiosepius paradoxus|Mollusca, Cephalopoda, Decapodiformes_incertae_sedis, Idiosepiidae, Idiosepius|EU008990|Ushimado, Japan
Idiosepius paradoxus|Mollusca, Cephalopoda, Decapodiformes_incertae_sedis, Idiosepiidae, Idiosepius|EU008992|Ushimado, Japan
Idiosepius paradoxus|Mollusca, Cephalopoda, Decapodiformes_incertae_sedis, Idiosepiidae, Idiosepius|EU008993|Ushimado, Japan
Idiosepius paradoxus|Mollusca, Cephalopoda, Decapodiformes_incertae_sedis, Idiosepiidae, Idiosepius|EU008994|Ushimado, Japan
Idiosepius paradoxus|Mollusca, Cephalopoda, Decapodiformes_incertae_sedis, Idiosepiidae, Idiosepius|EU008996|Ushimado, Japan

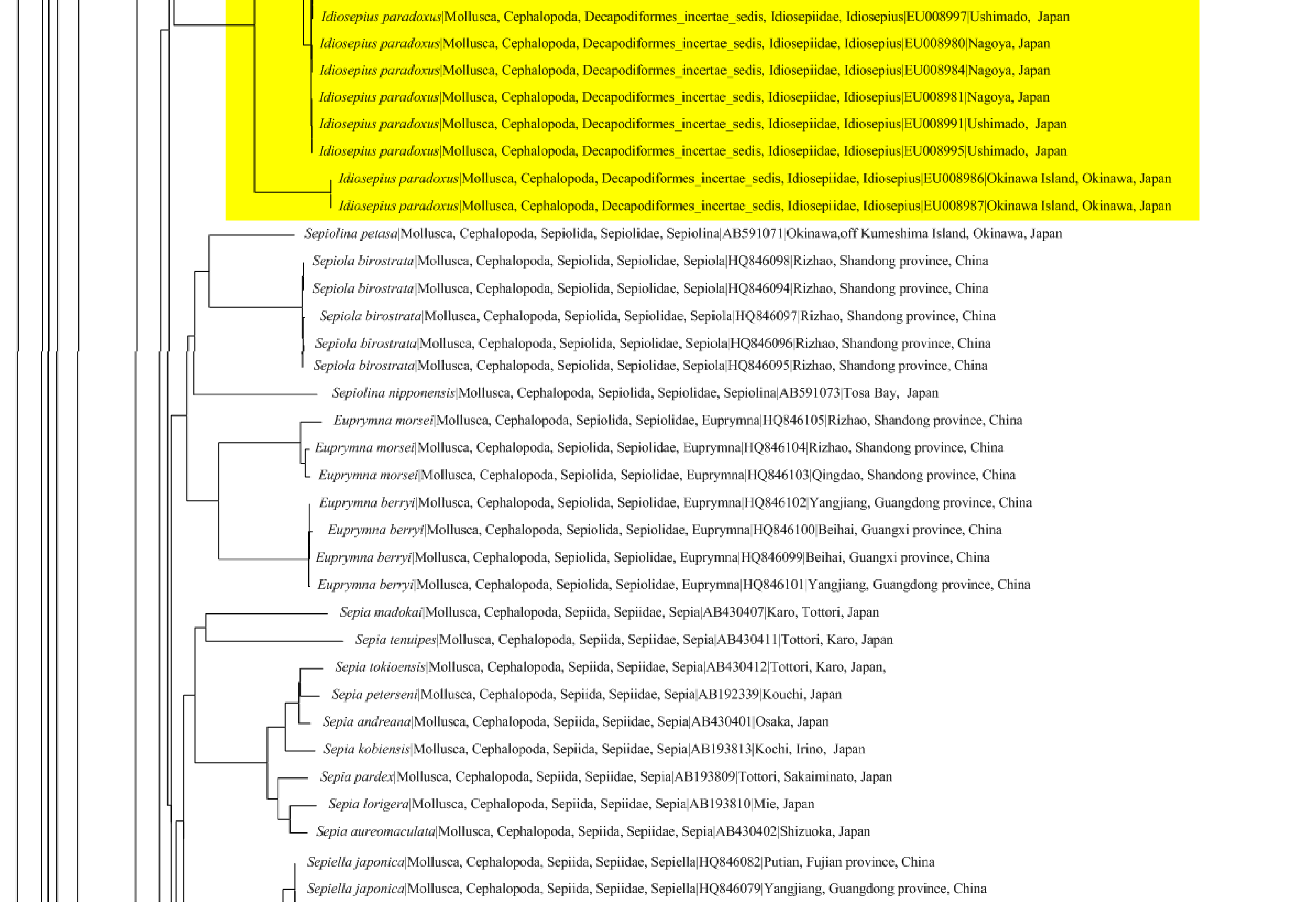
Idiosepius paradoxus|Mollusca, Cephalopoda, Decapodiformes_incertae_sedis, Idiosepiidae, Idiosepius|EU008997|Ushimado, Japan
Idiosepius paradoxus|Mollusca, Cephalopoda, Decapodiformes_incertae_sedis, Idiosepiidae, Idiosepius|EU008980|Nagoya, Japan
Idiosepius paradoxus|Mollusca, Cephalopoda, Decapodiformes_incertae_sedis, Idiosepiidae, Idiosepius|EU008984|Nagoya, Japan
Idiosepius paradoxus|Mollusca, Cephalopoda, Decapodiformes_incertae_sedis, Idiosepiidae, Idiosepius|EU008981|Nagoya, Japan
Idiosepius paradoxus|Mollusca, Cephalopoda, Decapodiformes_incertae_sedis, Idiosepiidae, Idiosepius|EU008991|Ushimado, Japan
Idiosepius paradoxus|Mollusca, Cephalopoda, Decapodiformes_incertae_sedis, Idiosepiidae, Idiosepius|EU008995|Ushimado, Japan
Idiosepius paradoxus|Mollusca, Cephalopoda, Decapodiformes_incertae_sedis, Idiosepiidae, Idiosepius|EU008986|Okinawa Island, Okinawa, Japan
Idiosepius paradoxus|Mollusca, Cephalopoda, Decapodiformes_incertae_sedis, Idiosepiidae, Idiosepius|EU008987|Okinawa Island, Okinawa, Japan
Sepiolina petasa|Mollusca, Cephalopoda, Sepiolida, Sepiolidae, Sepiolina|AB591071|Okinawa,off Kumeshima Island, Okinawa, Japan
Sepiola birostrata|Mollusca, Cephalopoda, Sepiolida, Sepiolidae, Sepiola|HQ846098|Rizhao, Shandong province, China
Sepiola birostrata|Mollusca, Cephalopoda, Sepiolida, Sepiolidae, Sepiola|HQ846094|Rizhao, Shandong province, China
Sepiola birostrata|Mollusca, Cephalopoda, Sepiolida, Sepiolidae, Sepiola|HQ846097|Rizhao, Shandong province, China
Sepiola birostrata|Mollusca, Cephalopoda, Sepiolida, Sepiolidae, Sepiola|HQ846096|Rizhao, Shandong province, China
Sepiola birostrata|Mollusca, Cephalopoda, Sepiolida, Sepiolidae, Sepiola|HQ846095|Rizhao, Shandong province, China
Sepiolina nipponensis|Mollusca, Cephalopoda, Sepiolida, Sepiolidae, Sepiolina|AB591073|Tosa Bay, Japan
Euprymna morsei|Mollusca, Cephalopoda, Sepiolida, Sepiolidae, Euprymna|HQ846105|Rizhao, Shandong province, China
Euprymna morsei|Mollusca, Cephalopoda, Sepiolida, Sepiolidae, Euprymna|HQ846104|Rizhao, Shandong province, China
Euprymna morsei|Mollusca, Cephalopoda, Sepiolida, Sepiolidae, Euprymna|HQ846103|Qingdao, Shandong province, China
Euprymna berryi|Mollusca, Cephalopoda, Sepiolida, Sepiolidae, Euprymna|HQ846102|Yangjiang, Guangdong province, China
Euprymna berryi|Mollusca, Cephalopoda, Sepiolida, Sepiolidae, Euprymna|HQ846100|Beihai, Guangxi province, China
Euprymna berryi|Mollusca, Cephalopoda, Sepiolida, Sepiolidae, Euprymna|HQ846099|Beihai, Guangxi province, China
Euprymna berryi|Mollusca, Cephalopoda, Sepiolida, Sepiolidae, Euprymna|HQ846101|Yangjiang, Guangdong province, China
Sepia madokai|Mollusca, Cephalopoda, Sepiida, Sepiidae, Sepia|AB430407|Karo, Tottori, Japan
Sepia tenuipes|Mollusca, Cephalopoda, Sepiida, Sepiidae, Sepia|AB430411|Tottori, Karo, Japan
Sepia tokioensis|Mollusca, Cephalopoda, Sepiida, Sepiidae, Sepia|AB430412|Tottori, Karo, Japan,
Sepia peterseni|Mollusca, Cephalopoda, Sepiida, Sepiidae, Sepia|AB192339|Kouchi, Japan
Sepia andreana|Mollusca, Cephalopoda, Sepiida, Sepiidae, Sepia|AB430401|Osaka, Japan
Sepia kobiensis|Mollusca, Cephalopoda, Sepiida, Sepiidae, Sepia|AB193813|Kochi, Irino, Japan
Sepia pardex|Mollusca, Cephalopoda, Sepiida, Sepiidae, Sepia|AB193809|Tottori, Sakaiminato, Japan
Sepia lorigera|Mollusca, Cephalopoda, Sepiida, Sepiidae, Sepia|AB193810|Mie, Japan
Sepia aureomaculata|Mollusca, Cephalopoda, Sepiida, Sepiidae, Sepia|AB430402|Shizuoka, Japan
Sepiella japonica|Mollusca, Cephalopoda, Sepiida, Sepiidae, Sepiella|HQ846082|Putian, Fujian province, China
Sepiella japonica|Mollusca, Cephalopoda, Sepiida, Sepiidae, Sepiella|HQ846079|Yangjiang, Guangdong province, China

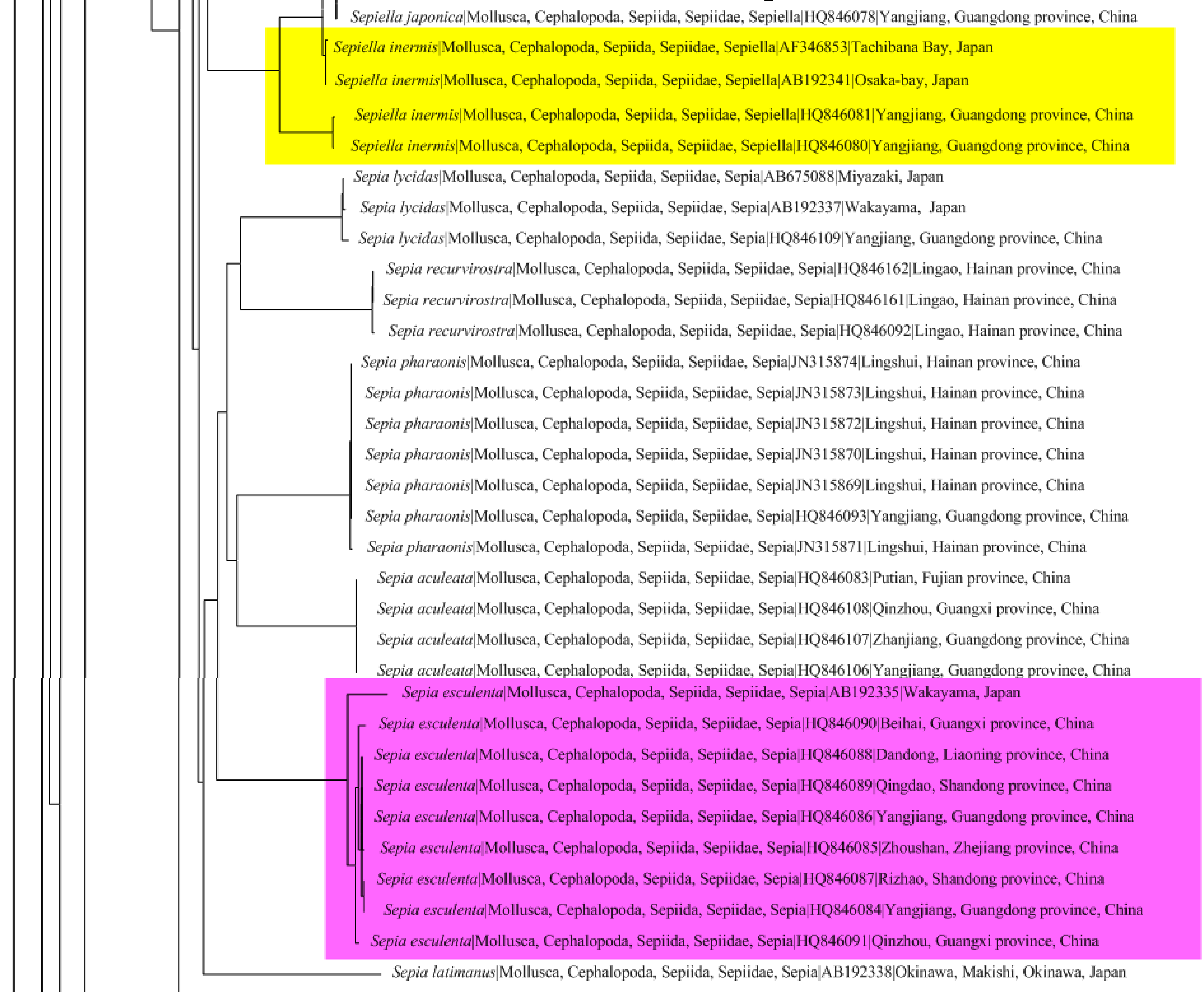

Sepiella japonica|Mollusca, Cephalopoda, Sepiida, Sepiidae, Sepiella|HQ846078|Yangjiang, Guangdong province, China
Sepiella inermis|Mollusca, Cephalopoda, Sepiida, Sepiidae, Sepiella|AF346853|Tachibana Bay, Japan
Sepiella inermis|Mollusca, Cephalopoda, Sepiida, Sepiidae, Sepiella|AB192341|Osaka-bay, Japan
Sepiella inermis|Mollusca, Cephalopoda, Sepiida, Sepiidae, Sepiella|HQ846081|Yangjiang, Guangdong province, China
Sepiella inermis|Mollusca, Cephalopoda, Sepiida, Sepiidae, Sepiella|HQ846080|Yangjiang, Guangdong province, China
Sepia lycidas|Mollusca, Cephalopoda, Sepiida, Sepiidae, Sepia|AB675088|Miyazaki, Japan
Sepia lycidas|Mollusca, Cephalopoda, Sepiida, Sepiidae, Sepia|AB192337|Wakayama, Japan
Sepia lycidas|Mollusca, Cephalopoda, Sepiida, Sepiidae, Sepia|HQ846109|Yangjiang, Guangdong province, China
Sepia recurvirostra|Mollusca, Cephalopoda, Sepiida, Sepiidae, Sepia|HQ846162|Lingao, Hainan province, China
Sepia recurvirostra|Mollusca, Cephalopoda, Sepiida, Sepiidae, Sepia|HQ846161|Lingao, Hainan province, China
Sepia recurvirostra|Mollusca, Cephalopoda, Sepiida, Sepiidae, Sepia|HQ846092|Lingao, Hainan province, China
Sepia pharaonis|Mollusca, Cephalopoda, Sepiida, Sepiidae, Sepia|JN315874|Lingshui, Hainan province, China
Sepia pharaonis|Mollusca, Cephalopoda, Sepiida, Sepiidae, Sepia|JN315873|Lingshui, Hainan province, China
Sepia pharaonis|Mollusca, Cephalopoda, Sepiida, Sepiidae, Sepia|JN315872|Lingshui, Hainan province, China
Sepia pharaonis|Mollusca, Cephalopoda, Sepiida, Sepiidae, Sepia|JN315870|Lingshui, Hainan province, China
Sepia pharaonis|Mollusca, Cephalopoda, Sepiida, Sepiidae, Sepia|JN315869|Lingshui, Hainan province, China
Sepia pharaonis|Mollusca, Cephalopoda, Sepiida, Sepiidae, Sepia|HQ846093|Yangjiang, Guangdong province, China
Sepia pharaonis|Mollusca, Cephalopoda, Sepiida, Sepiidae, Sepia|JN315871|Lingshui, Hainan province, China
Sepia aculeata|Mollusca, Cephalopoda, Sepiida, Sepiidae, Sepia|HQ846083|Putian, Fujian province, China
Sepia aculeata|Mollusca, Cephalopoda, Sepiida, Sepiidae, Sepia|HQ846108|Qinzhou, Guangxi province, China
Sepia aculeata|Mollusca, Cephalopoda, Sepiida, Sepiidae, Sepia|HQ846107|Zhanjiang, Guangdong province, China
Sepia aculeata|Mollusca, Cephalopoda, Sepiida, Sepiidae, Sepia|HQ846106|Yangjiang, Guangdong province, China
Sepia esculenta|Mollusca, Cephalopoda, Sepiida, Sepiidae, Sepia|AB192335|Wakayama, Japan
Sepia esculenta|Mollusca, Cephalopoda, Sepiida, Sepiidae, Sepia|HQ846090|Beihai, Guangxi province, China
Sepia esculenta|Mollusca, Cephalopoda, Sepiida, Sepiidae, Sepia|HQ846088|Dandong, Liaoning province, China
Sepia esculenta|Mollusca, Cephalopoda, Sepiida, Sepiidae, Sepia|HQ846089|Qingdao, Shandong province, China
Sepia esculenta|Mollusca, Cephalopoda, Sepiida, Sepiidae, Sepia|HQ846086|Yangjiang, Guangdong province, China
Sepia esculenta|Mollusca, Cephalopoda, Sepiida, Sepiidae, Sepia|HQ846085|Zhoushan, Zhejiang province, China
Sepia esculenta|Mollusca, Cephalopoda, Sepiida, Sepiidae, Sepia|HQ846087|Rizhao, Shandong province, China
Sepia esculenta|Mollusca, Cephalopoda, Sepiida, Sepiidae, Sepia|HQ846084|Yangjiang, Guangdong province, China
Sepia esculenta|Mollusca, Cephalopoda, Sepiida, Sepiidae, Sepia|HQ846091|Qinzhou, Guangxi province, China
Sepia latimanus|Mollusca, Cephalopoda, Sepiida, Sepiidae, Sepia|AB192338|Okinawa, Makishi, Okinawa, Japan

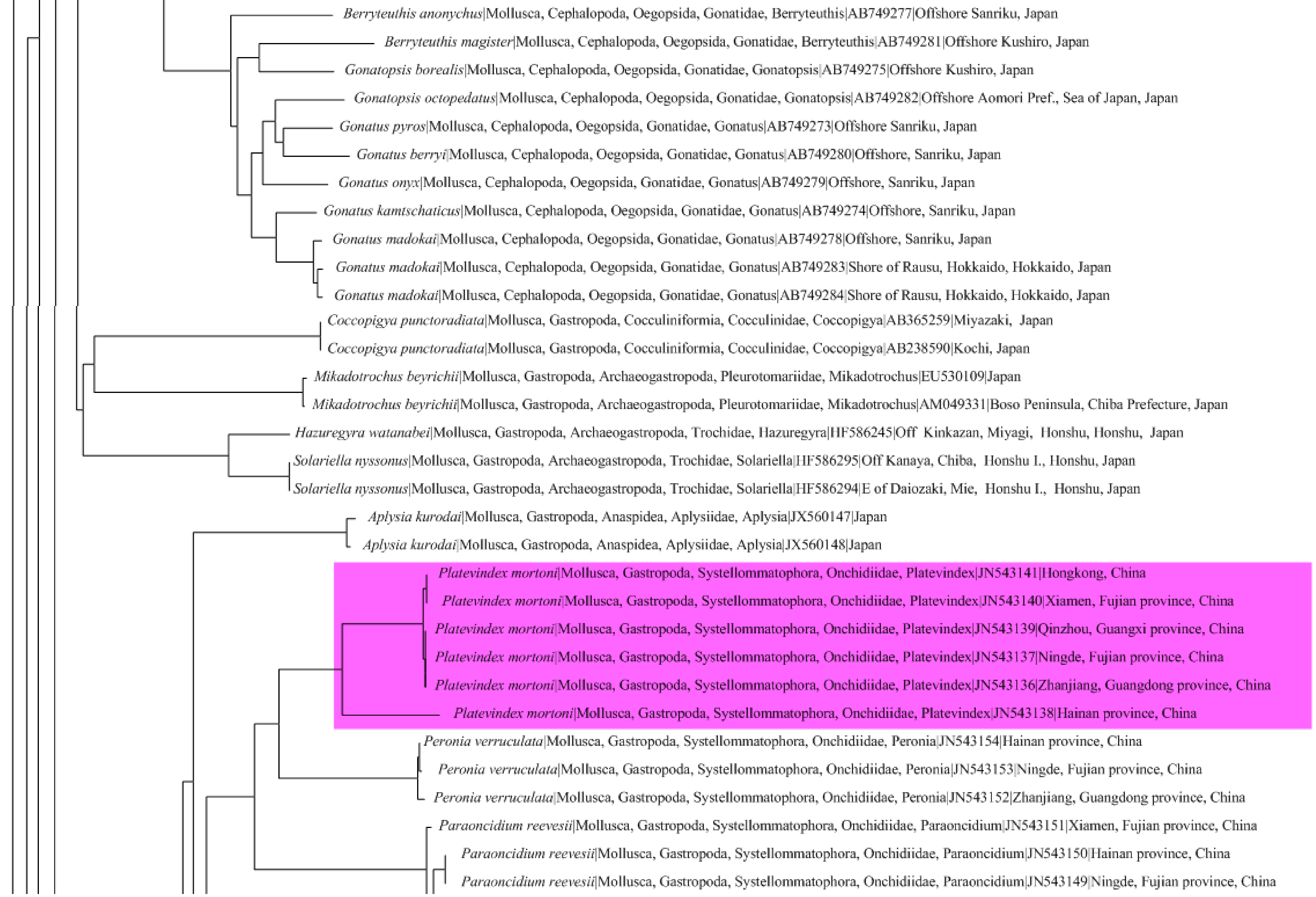

Berryteuthis anonychus|Mollusca, Cephalopoda, Oegopsida, Gonatidae, Berryteuthis|AB749277|Offshore Sanriku, Japan
Berryteuthis magister|Mollusca, Cephalopoda, Oegopsida, Gonatidae, Berryteuthis|AB749281|Offshore Kushiro, Japan
Gonatopsis borealis|Mollusca, Cephalopoda, Oegopsida, Gonatidae, Gonatopsis|AB749275|Offshore Kushiro, Japan
Gonatopsis octopedatus|Mollusca, Cephalopoda, Oegopsida, Gonatidae, Gonatopsis|AB749282|Offshore Aomori Pref., Sea of Japan, Japan
Gonatus pyros|Mollusca, Cephalopoda, Oegopsida, Gonatidae, Gonatus|AB749273|Offshore Sanriku, Japan
Gonatus berryi|Mollusca, Cephalopoda, Oegopsida, Gonatidae, Gonatus|AB749280|Offshore, Sanriku, Japan
Gonatus onyx|Mollusca, Cephalopoda, Oegopsida, Gonatidae, Gonatus|AB749279|Offshore, Sanriku, Japan
Gonatus kamtschaticus|Mollusca, Cephalopoda, Oegopsida, Gonatidae, Gonatus|AB749274|Offshore, Sanriku, Japan
Gonatus madokai|Mollusca, Cephalopoda, Oegopsida, Gonatidae, Gonatus|AB749278|Offshore, Sanriku, Japan
Gonatus madokai|Mollusca, Cephalopoda, Oegopsida, Gonatidae, Gonatus|AB749283|Shore of Rausu, Hokkaido, Hokkaido, Japan
Gonatus madokai|Mollusca, Cephalopoda, Oegopsida, Gonatidae, Gonatus|AB749284|Shore of Rausu, Hokkaido, Hokkaido, Japan
Coccopigya punctoradiata|Mollusca, Gastropoda, Cocculiniformia, Cocculinidae, Coccopigya|AB365259|Miyazaki, Japan
Coccopigya punctoradiata|Mollusca, Gastropoda, Cocculiniformia, Cocculinidae, Coccopigya|AB238590|Kochi, Japan
Mikadotrochus beyrichii|Mollusca, Gastropoda, Archaeogastropoda, Pleurotomariidae, Mikadotrochus|EU530109|Japan
Mikadotrochus beyrichii|Mollusca, Gastropoda, Archaeogastropoda, Pleurotomariidae, Mikadotrochus|AM049331|Boso Peninsula, Chiba Prefecture, Japan
Hazuregyra watanabei|Mollusca, Gastropoda, Archaeogastropoda, Trochidae, Hazuregyra|HF586245|Off Kinkazan, Miyagi, Honshu, Honshu, Japan
Solariella nyssonus|Mollusca, Gastropoda, Archaeogastropoda, Trochidae, Solariella|HF586295|Off Kanaya, Chiba, Honshu I., Honshu, Japan
Solariella nyssonus|Mollusca, Gastropoda, Archaeogastropoda, Trochidae, Solariella|HF586294|E of Daiozaki, Mie, Honshu I., Honshu, Japan
Aplysia kurodai|Mollusca, Gastropoda, Anaspidea, Aplysiidae, Aplysia|JX560147|Japan
Aplysia kurodai|Mollusca, Gastropoda, Anaspidea, Aplysiidae, Aplysia|JX560148|Japan
Platevindex mortoni|Mollusca, Gastropoda, Systellommatophora, Onchidiidae, Platevindex|JN543141|Hongkong, China
Platevindex mortoni|Mollusca, Gastropoda, Systellommatophora, Onchidiidae, Platevindex|JN543140|Xiamen, Fujian province, China
Platevindex mortoni|Mollusca, Gastropoda, Systellommatophora, Onchidiidae, Platevindex|JN543139|Qinzhou, Guangxi province, China
Platevindex mortoni|Mollusca, Gastropoda, Systellommatophora, Onchidiidae, Platevindex|JN543137|Ningde, Fujian province, China
Platevindex mortoni|Mollusca, Gastropoda, Systellommatophora, Onchidiidae, Platevindex|JN543136|Zhanjiang, Guangdong province, China
Platevindex mortoni|Mollusca, Gastropoda, Systellommatophora, Onchidiidae, Platevindex|JN543138|Hainan province, China
Peronia verruculata|Mollusca, Gastropoda, Systellommatophora, Onchidiidae, Peronia|JN543154|Hainan province, China
Peronia verruculata|Mollusca, Gastropoda, Systellommatophora, Onchidiidae, Peronia|JN543153|Ningde, Fujian province, China
Peronia verruculata|Mollusca, Gastropoda, Systellommatophora, Onchidiidae, Peronia|JN543152|Zhanjiang, Guangdong province, China
Paraoncidium reevesii|Mollusca, Gastropoda, Systellommatophora, Onchidiidae, Paraoncidium|JN543151|Xiamen, Fujian province, China
Paraoncidium reevesii|Mollusca, Gastropoda, Systellommatophora, Onchidiidae, Paraoncidium|JN543150|Hainan province, China
Paraoncidium reevesii|Mollusca, Gastropoda, Systellommatophora, Onchidiidae, Paraoncidium|JN543149|Ningde, Fujian province, China

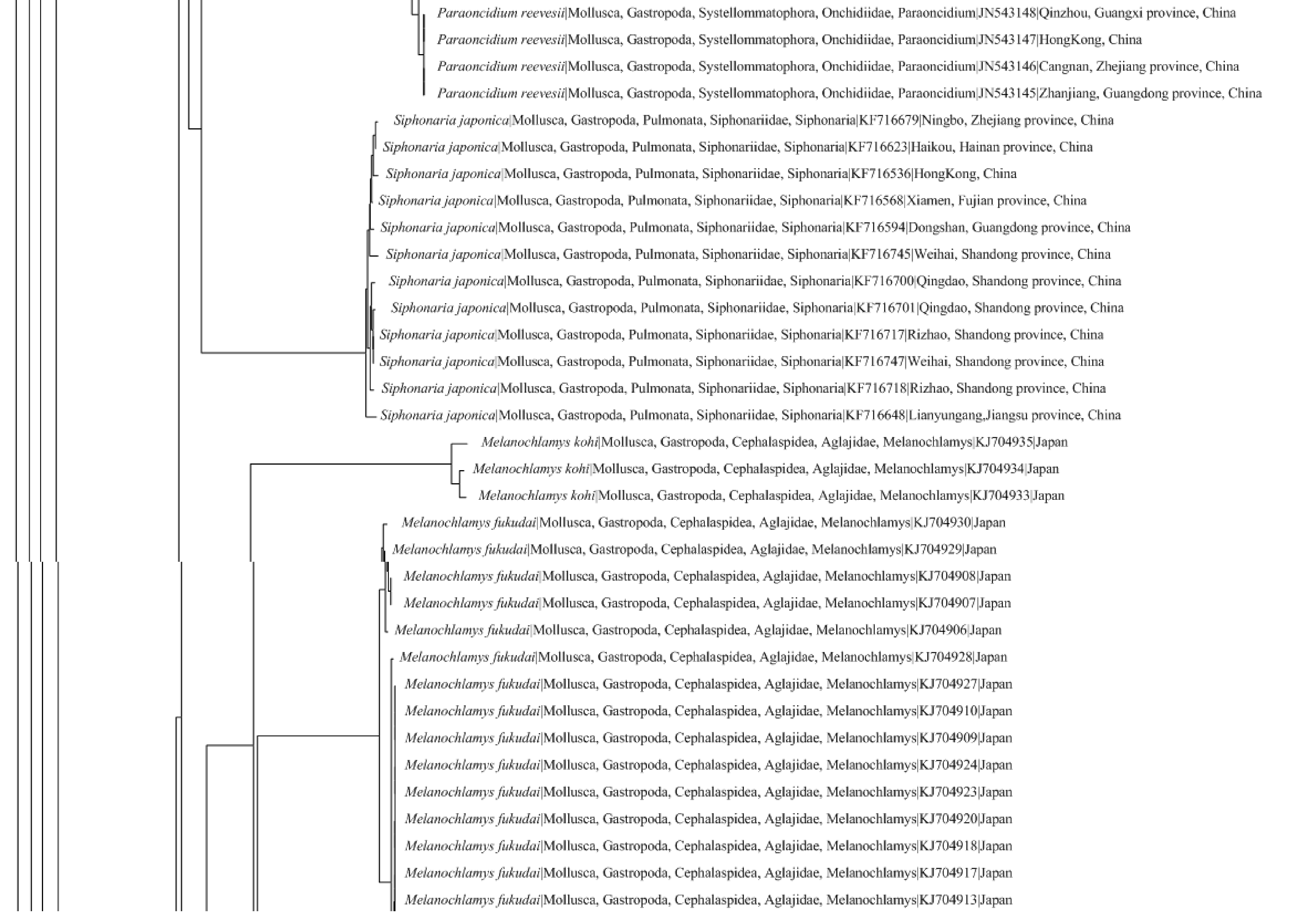
Paraoncidium reevesii|Mollusca, Gastropoda, Systellommatophora, Onchidiidae, Paraoncidium|JN543148|Qinzhou, Guangxi province, China
Paraoncidium reevesii|Mollusca, Gastropoda, Systellommatophora, Onchidiidae, Paraoncidium|JN543147|HongKong, China
Paraoncidium reevesii|Mollusca, Gastropoda, Systellommatophora, Onchidiidae, Paraoncidium|JN543146|Cangnan, Zhejiang province, China
Paraoncidium reevesii|Mollusca, Gastropoda, Systellommatophora, Onchidiidae, Paraoncidium|JN543145|Zhanjiang, Guangdong province, China
Siphonaria japonica|Mollusca, Gastropoda, Pulmonata, Siphonariidae, Siphonaria|KF716679|Ningbo, Zhejiang province, China
Siphonaria japonica|Mollusca, Gastropoda, Pulmonata, Siphonariidae, Siphonaria|KF716623|Haikou, Hainan province, China
Siphonaria japonica|Mollusca, Gastropoda, Pulmonata, Siphonariidae, Siphonaria|KF716536|HongKong, China
Siphonaria japonica|Mollusca, Gastropoda, Pulmonata, Siphonariidae, Siphonaria|KF716568|Xiamen, Fujian province, China
Siphonaria japonica|Mollusca, Gastropoda, Pulmonata, Siphonariidae, Siphonaria|KF716594|Dongshan, Guangdong province, China
Siphonaria japonica|Mollusca, Gastropoda, Pulmonata, Siphonariidae, Siphonaria|KF716745|Weihai, Shandong province, China
Siphonaria japonica|Mollusca, Gastropoda, Pulmonata, Siphonariidae, Siphonaria|KF716700|Qingdao, Shandong province, China
Siphonaria japonica|Mollusca, Gastropoda, Pulmonata, Siphonariidae, Siphonaria|KF716701|Qingdao, Shandong province, China
Siphonaria japonica|Mollusca, Gastropoda, Pulmonata, Siphonariidae, Siphonaria|KF716717|Rizhao, Shandong province, China
Siphonaria japonica|Mollusca, Gastropoda, Pulmonata, Siphonariidae, Siphonaria|KF716747|Weihai, Shandong province, China
Siphonaria japonica|Mollusca, Gastropoda, Pulmonata, Siphonariidae, Siphonaria|KF716718|Rizhao, Shandong province, China
Siphonaria japonica|Mollusca, Gastropoda, Pulmonata, Siphonariidae, Siphonaria|KF716648|Lianyungang,Jiangsu province, China
Melanochlamys kohi|Mollusca, Gastropoda, Cephalaspidea, Aglajidae, Melanochlamys|KJ704935|Japan
Melanochlamys kohi|Mollusca, Gastropoda, Cephalaspidea, Aglajidae, Melanochlamys|KJ704934|Japan
Melanochlamys kohi|Mollusca, Gastropoda, Cephalaspidea, Aglajidae, Melanochlamys|KJ704933|Japan
Melanochlamys fukudai|Mollusca, Gastropoda, Cephalaspidea, Aglajidae, Melanochlamys|KJ704930|Japan
Melanochlamys fukudai|Mollusca, Gastropoda, Cephalaspidea, Aglajidae, Melanochlamys|KJ704929|Japan
Melanochlamys fukudai|Mollusca, Gastropoda, Cephalaspidea, Aglajidae, Melanochlamys|KJ704908|Japan
Melanochlamys fukudai|Mollusca, Gastropoda, Cephalaspidea, Aglajidae, Melanochlamys|KJ704907|Japan
Melanochlamys fukudai|Mollusca, Gastropoda, Cephalaspidea, Aglajidae, Melanochlamys|KJ704906|Japan
Melanochlamys fukudai|Mollusca, Gastropoda, Cephalaspidea, Aglajidae, Melanochlamys|KJ704928|Japan
Melanochlamys fukudai|Mollusca, Gastropoda, Cephalaspidea, Aglajidae, Melanochlamys|KJ704927|Japan
Melanochlamys fukudai|Mollusca, Gastropoda, Cephalaspidea, Aglajidae, Melanochlamys|KJ704910|Japan
Melanochlamys fukudai|Mollusca, Gastropoda, Cephalaspidea, Aglajidae, Melanochlamys|KJ704909|Japan
Melanochlamys fukudai|Mollusca, Gastropoda, Cephalaspidea, Aglajidae, Melanochlamys|KJ704924|Japan
Melanochlamys fukudai|Mollusca, Gastropoda, Cephalaspidea, Aglajidae, Melanochlamys|KJ704923|Japan
Melanochlamys fukudai|Mollusca, Gastropoda, Cephalaspidea, Aglajidae, Melanochlamys|KJ704920|Japan
Melanochlamys fukudai|Mollusca, Gastropoda, Cephalaspidea, Aglajidae, Melanochlamys|KJ704918|Japan
Melanochlamys fukudai|Mollusca, Gastropoda, Cephalaspidea, Aglajidae, Melanochlamys|KJ704917|Japan
Melanochlamys fukudai|Mollusca, Gastropoda, Cephalaspidea, Aglajidae, Melanochlamys|KJ704913|Japan

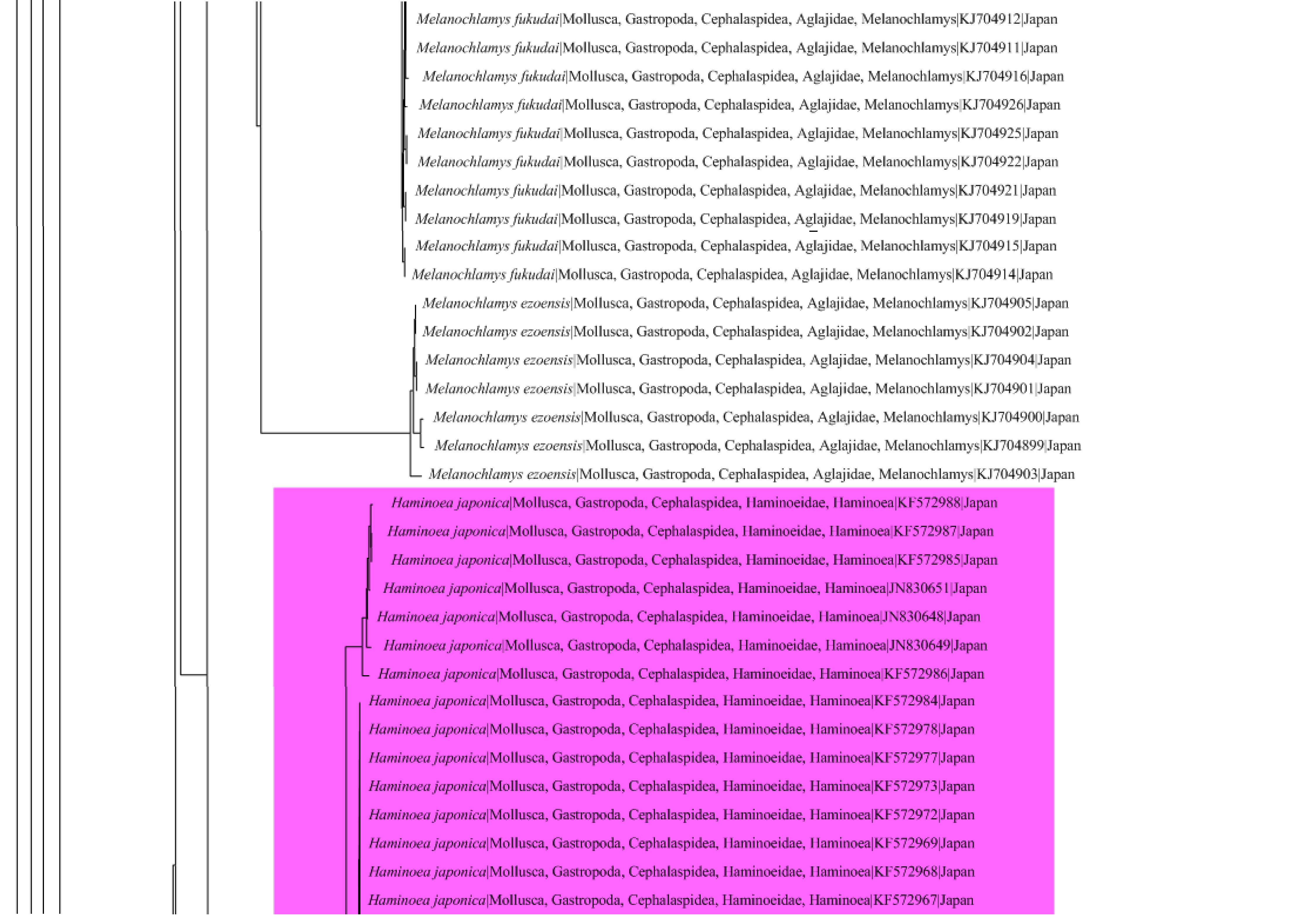
Melanochlamys fukudai|Mollusca, Gastropoda, Cephalaspidea, Aglajidae, Melanochlamys|KJ704912|Japan
Melanochlamys fukudai|Mollusca, Gastropoda, Cephalaspidea, Aglajidae, Melanochlamys|KJ704911|Japan
Melanochlamys fukudai|Mollusca, Gastropoda, Cephalaspidea, Aglajidae, Melanochlamys|KJ704916|Japan
Melanochlamys fukudai|Mollusca, Gastropoda, Cephalaspidea, Aglajidae, Melanochlamys|KJ704926|Japan
Melanochlamys fukudai|Mollusca, Gastropoda, Cephalaspidea, Aglajidae, Melanochlamys|KJ704925|Japan
Melanochlamys fukudai|Mollusca, Gastropoda, Cephalaspidea, Aglajidae, Melanochlamys|KJ704922|Japan
Melanochlamys fukudai|Mollusca, Gastropoda, Cephalaspidea, Aglajidae, Melanochlamys|KJ704921|Japan
Melanochlamys fukudai|Mollusca, Gastropoda, Cephalaspidea, Aglajidae, Melanochlamys|KJ704919|Japan
Melanochlamys fukudai|Mollusca, Gastropoda, Cephalaspidea, Aglajidae, Melanochlamys|KJ704915|Japan
Melanochlamys fukudai|Mollusca, Gastropoda, Cephalaspidea, Aglajidae, Melanochlamys|KJ704914|Japan
Melanochlamys ezoensis|Mollusca, Gastropoda, Cephalaspidea, Aglajidae, Melanochlamys|KJ704905|Japan
Melanochlamys ezoensis|Mollusca, Gastropoda, Cephalaspidea, Aglajidae, Melanochlamys|KJ704902|Japan
Melanochlamys ezoensis|Mollusca, Gastropoda, Cephalaspidea, Aglajidae, Melanochlamys|KJ704904|Japan
Melanochlamys ezoensis|Mollusca, Gastropoda, Cephalaspidea, Aglajidae, Melanochlamys|KJ704901|Japan
Melanochlamys ezoensis|Mollusca, Gastropoda, Cephalaspidea, Aglajidae, Melanochlamys|KJ704900|Japan
Melanochlamys ezoensis|Mollusca, Gastropoda, Cephalaspidea, Aglajidae, Melanochlamys|KJ704899|Japan
Melanochlamys ezoensis|Mollusca, Gastropoda, Cephalaspidea, Aglajidae, Melanochlamys|KJ704903|Japan
Haminoea japonica|Mollusca, Gastropoda, Cephalaspidea, Haminoeidae, Haminoea|KF572988|Japan
Haminoea japonica|Mollusca, Gastropoda, Cephalaspidea, Haminoeidae, Haminoea|KF572987|Japan
Haminoea japonica|Mollusca, Gastropoda, Cephalaspidea, Haminoeidae, Haminoea|KF572985|Japan
Haminoea japonica|Mollusca, Gastropoda, Cephalaspidea, Haminoeidae, Haminoea|JN830651|Japan
Haminoea japonica|Mollusca, Gastropoda, Cephalaspidea, Haminoeidae, Haminoea|JN830648|Japan
Haminoea japonica|Mollusca, Gastropoda, Cephalaspidea, Haminoeidae, Haminoea|JN830649|Japan
Haminoea japonica|Mollusca, Gastropoda, Cephalaspidea, Haminoeidae, Haminoea|KF572986|Japan
Haminoea japonica|Mollusca, Gastropoda, Cephalaspidea, Haminoeidae, Haminoea|KF572984|Japan
Haminoea japonica|Mollusca, Gastropoda, Cephalaspidea, Haminoeidae, Haminoea|KF572978|Japan
Haminoea japonica|Mollusca, Gastropoda, Cephalaspidea, Haminoeidae, Haminoea|KF572977|Japan
Haminoea japonica|Mollusca, Gastropoda, Cephalaspidea, Haminoeidae, Haminoea|KF572973|Japan
Haminoea japonica|Mollusca, Gastropoda, Cephalaspidea, Haminoeidae, Haminoea|KF572972|Japan
Haminoea japonica|Mollusca, Gastropoda, Cephalaspidea, Haminoeidae, Haminoea|KF572969|Japan
Haminoea japonica|Mollusca, Gastropoda, Cephalaspidea, Haminoeidae, Haminoea|KF572968|Japan
Haminoea japonica|Mollusca, Gastropoda, Cephalaspidea, Haminoeidae, Haminoea|KF572967|Japan

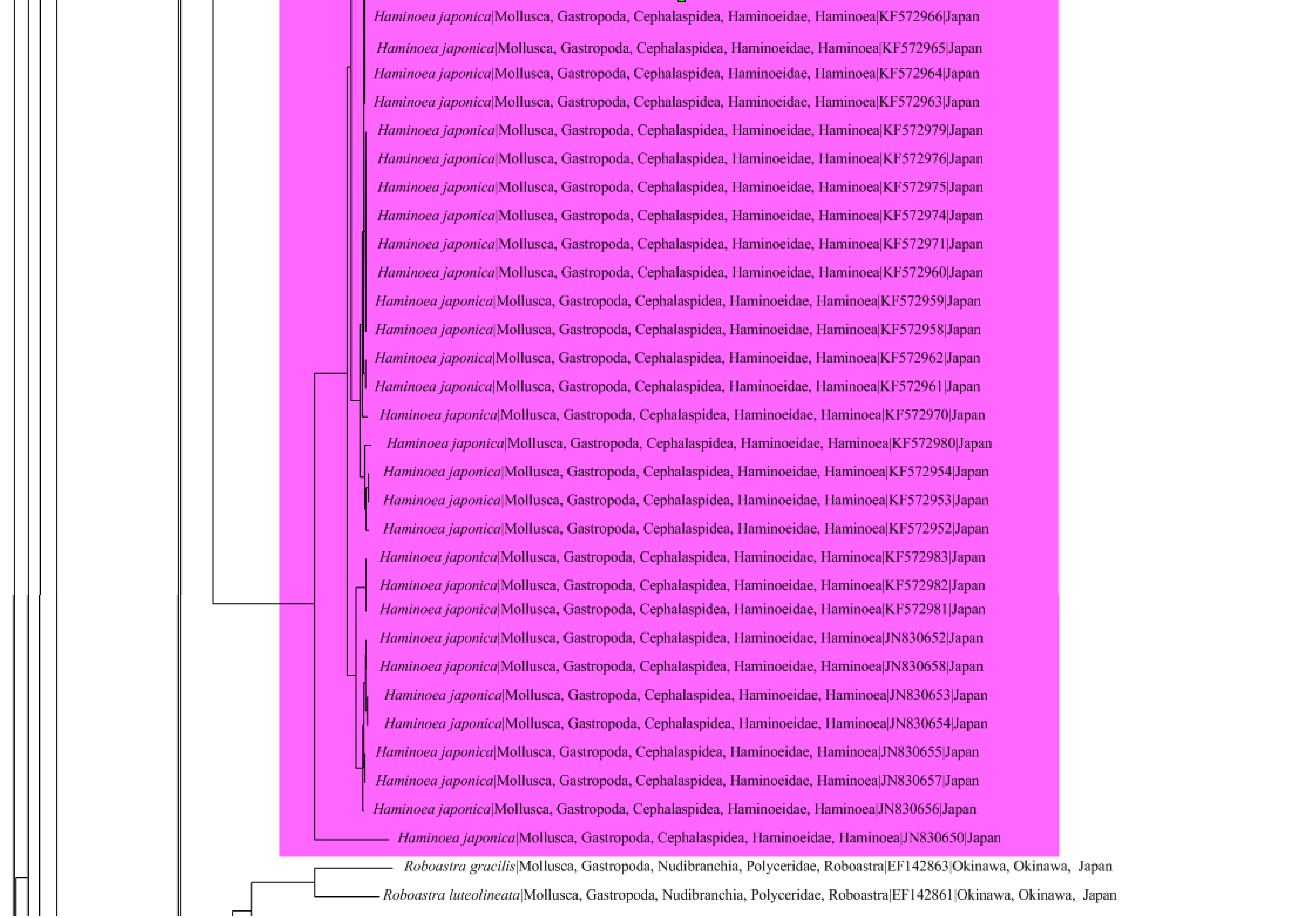

Haminoea japonica|Mollusca, Gastropoda, Cephalaspidea, Haminoeidae, Haminoea|KF572966|Japan
Haminoea japonica|Mollusca, Gastropoda, Cephalaspidea, Haminoeidae, Haminoea|KF572965|Japan
Haminoea japonica|Mollusca, Gastropoda, Cephalaspidea, Haminoeidae, Haminoea|KF572964|Japan
Haminoea japonica|Mollusca, Gastropoda, Cephalaspidea, Haminoeidae, Haminoea|KF572963|Japan
Haminoea japonica|Mollusca, Gastropoda, Cephalaspidea, Haminoeidae, Haminoea|KF572979|Japan
Haminoea japonica|Mollusca, Gastropoda, Cephalaspidea, Haminoeidae, Haminoea|KF572976|Japan
Haminoea japonica|Mollusca, Gastropoda, Cephalaspidea, Haminoeidae, Haminoea|KF572975|Japan
Haminoea japonica|Mollusca, Gastropoda, Cephalaspidea, Haminoeidae, Haminoea|KF572974|Japan
Haminoea japonica|Mollusca, Gastropoda, Cephalaspidea, Haminoeidae, Haminoea|KF572971|Japan
Haminoea japonica|Mollusca, Gastropoda, Cephalaspidea, Haminoeidae, Haminoea|KF572960|Japan
Haminoea japonica|Mollusca, Gastropoda, Cephalaspidea, Haminoeidae, Haminoea|KF572959|Japan
Haminoea japonica|Mollusca, Gastropoda, Cephalaspidea, Haminoeidae, Haminoea|KF572958|Japan
Haminoea japonica|Mollusca, Gastropoda, Cephalaspidea, Haminoeidae, Haminoea|KF572962|Japan
Haminoea japonica|Mollusca, Gastropoda, Cephalaspidea, Haminoeidae, Haminoea|KF572961|Japan
Haminoea japonica|Mollusca, Gastropoda, Cephalaspidea, Haminoeidae, Haminoea|KF572970|Japan
Haminoea japonica|Mollusca, Gastropoda, Cephalaspidea, Haminoeidae, Haminoea|KF572980|Japan
Haminoea japonica|Mollusca, Gastropoda, Cephalaspidea, Haminoeidae, Haminoea|KF572954|Japan
Haminoea japonica|Mollusca, Gastropoda, Cephalaspidea, Haminoeidae, Haminoea|KF572953|Japan
Haminoea japonica|Mollusca, Gastropoda, Cephalaspidea, Haminoeidae, Haminoea|KF572952|Japan
Haminoea japonica|Mollusca, Gastropoda, Cephalaspidea, Haminoeidae, Haminoea|KF572983|Japan
Haminoea japonica|Mollusca, Gastropoda, Cephalaspidea, Haminoeidae, Haminoea|KF572982|Japan
Haminoea japonica|Mollusca, Gastropoda, Cephalaspidea, Haminoeidae, Haminoea|KF572981|Japan
Haminoea japonica|Mollusca, Gastropoda, Cephalaspidea, Haminoeidae, Haminoea|JN830652|Japan
Haminoea japonica|Mollusca, Gastropoda, Cephalaspidea, Haminoeidae, Haminoea|JN830658|Japan
Haminoea japonica|Mollusca, Gastropoda, Cephalaspidea, Haminoeidae, Haminoea|JN830653|Japan
Haminoea japonica|Mollusca, Gastropoda, Cephalaspidea, Haminoeidae, Haminoea|JN830654|Japan
Haminoea japonica|Mollusca, Gastropoda, Cephalaspidea, Haminoeidae, Haminoea|JN830655|Japan
Haminoea japonica|Mollusca, Gastropoda, Cephalaspidea, Haminoeidae, Haminoea|JN830657|Japan
Haminoea japonica|Mollusca, Gastropoda, Cephalaspidea, Haminoeidae, Haminoea|JN830656|Japan
Haminoea japonica|Mollusca, Gastropoda, Cephalaspidea, Haminoeidae, Haminoea|JN830650|Japan
Roboastra gracilis|Mollusca, Gastropoda, Nudibranchia, Polyceridae, Roboastra|EF142863|Okinawa, Okinawa, Japan
Roboastra luteolineata|Mollusca, Gastropoda, Nudibranchia, Polyceridae, Roboastra|EF142861|Okinawa, Okinawa, Japan

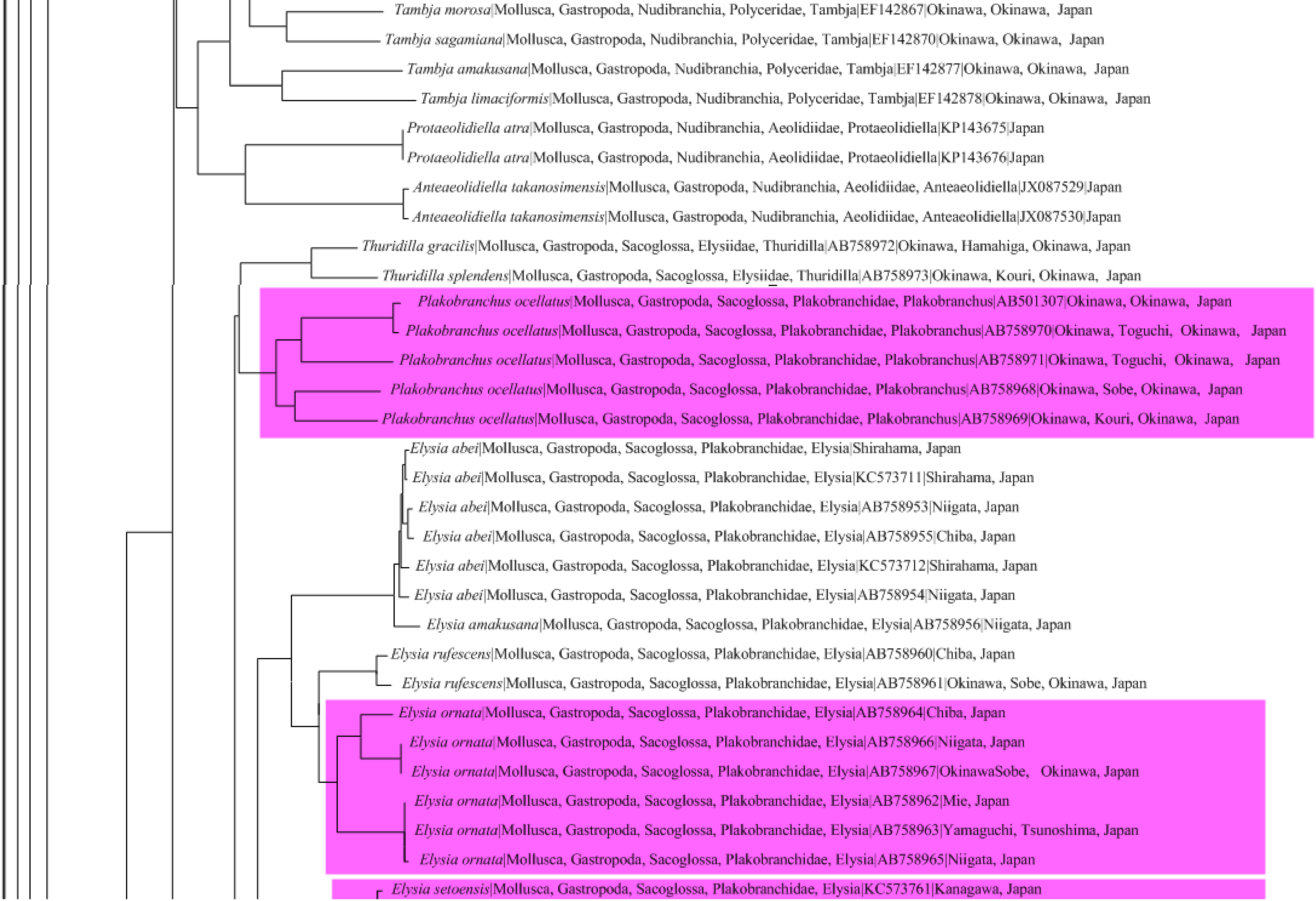

Tambja morosa|Mollusca, Gastropoda, Nudibranchia, Polyceridae, Tambja|EF142867|Okinawa, Okinawa, Japan
Tambja sagamiana|Mollusca, Gastropoda, Nudibranchia, Polyceridae, Tambja|EF142870|Okinawa, Okinawa, Japan
Tambja amakusana|Mollusca, Gastropoda, Nudibranchia, Polyceridae, Tambja|EF142877|Okinawa, Okinawa, Japan
Tambja limaciformis|Mollusca, Gastropoda, Nudibranchia, Polyceridae, Tambja|EF142878|Okinawa, Okinawa, Japan
Protaeolidiella atra|Mollusca, Gastropoda, Nudibranchia, Aeolidiidae, Protaeolidiella|KP143675|Japan
Protaeolidiella atra|Mollusca, Gastropoda, Nudibranchia, Aeolidiidae, Protaeolidiella|KP143676|Japan
Anteaeolidiella takanosimensis|Mollusca, Gastropoda, Nudibranchia, Aeolidiidae, Anteaeolidiella|JX087529|Japan
Anteaeolidiella takanosimensis|Mollusca, Gastropoda, Nudibranchia, Aeolidiidae, Anteaeolidiella|JX087530|Japan
Thuridilla gracilis|Mollusca, Gastropoda, Sacoglossa, Elysiidae, Thuridilla|AB758972|Okinawa, Hamahiga, Okinawa, Japan
Thuridilla splendens|Mollusca, Gastropoda, Sacoglossa, Elysiidae, Thuridilla|AB758973|Okinawa, Kouri, Okinawa, Japan
Plakobranchus ocellatus|Mollusca, Gastropoda, Sacoglossa, Plakobranchidae, Plakobranchus|AB501307|Okinawa, Okinawa, Japan
Plakobranchus ocellatus|Mollusca, Gastropoda, Sacoglossa, Plakobranchidae, Plakobranchus|AB758970|Okinawa, Toguchi, Okinawa, Japan
Plakobranchus ocellatus|Mollusca, Gastropoda, Sacoglossa, Plakobranchidae, Plakobranchus|AB758971|Okinawa, Toguchi, Okinawa, Japan
Plakobranchus ocellatus|Mollusca, Gastropoda, Sacoglossa, Plakobranchidae, Plakobranchus|AB758968|Okinawa, Sobe, Okinawa, Japan
Plakobranchus ocellatus|Mollusca, Gastropoda, Sacoglossa, Plakobranchidae, Plakobranchus|AB758969|Okinawa, Kouri, Okinawa, Japan
Elysia abei|Mollusca, Gastropoda, Sacoglossa, Plakobranchidae, Elysia|Shirahama, Japan
Elysia abei|Mollusca, Gastropoda, Sacoglossa, Plakobranchidae, Elysia|KC573711|Shirahama, Japan
Elysia abei|Mollusca, Gastropoda, Sacoglossa, Plakobranchidae, Elysia|AB758953|Niigata, Japan
Elysia abei|Mollusca, Gastropoda, Sacoglossa, Plakobranchidae, Elysia|AB758955|Chiba, Japan
Elysia abei|Mollusca, Gastropoda, Sacoglossa, Plakobranchidae, Elysia|KC573712|Shirahama, Japan
Elysia abei|Mollusca, Gastropoda, Sacoglossa, Plakobranchidae, Elysia|AB758954|Niigata, Japan
Elysia amakusana|Mollusca, Gastropoda, Sacoglossa, Plakobranchidae, Elysia|AB758956|Niigata, Japan
Elysia rufescens|Mollusca, Gastropoda, Sacoglossa, Plakobranchidae, Elysia|AB758960|Chiba, Japan
Elysia rufescens|Mollusca, Gastropoda, Sacoglossa, Plakobranchidae, Elysia|AB758961|Okinawa, Sobe, Okinawa, Japan
Elysia ornata|Mollusca, Gastropoda, Sacoglossa, Plakobranchidae, Elysia|AB758964|Chiba, Japan
Elysia ornata|Mollusca, Gastropoda, Sacoglossa, Plakobranchidae, Elysia|AB758966|Niigata, Japan
Elysia ornata|Mollusca, Gastropoda, Sacoglossa, Plakobranchidae, Elysia|AB758967|OkinawaSobe, Okinawa, Japan
Elysia ornata|Mollusca, Gastropoda, Sacoglossa, Plakobranchidae, Elysia|AB758962|Mie, Japan
Elysia ornata|Mollusca, Gastropoda, Sacoglossa, Plakobranchidae, Elysia|AB758963|Yamaguchi, Tsunoshima, Japan
Elysia ornata|Mollusca, Gastropoda, Sacoglossa, Plakobranchidae, Elysia|AB758965|Niigata, Japan
Elysia setoensis|Mollusca, Gastropoda, Sacoglossa, Plakobranchidae, Elysia|KC573761|Kanagawa, Japan

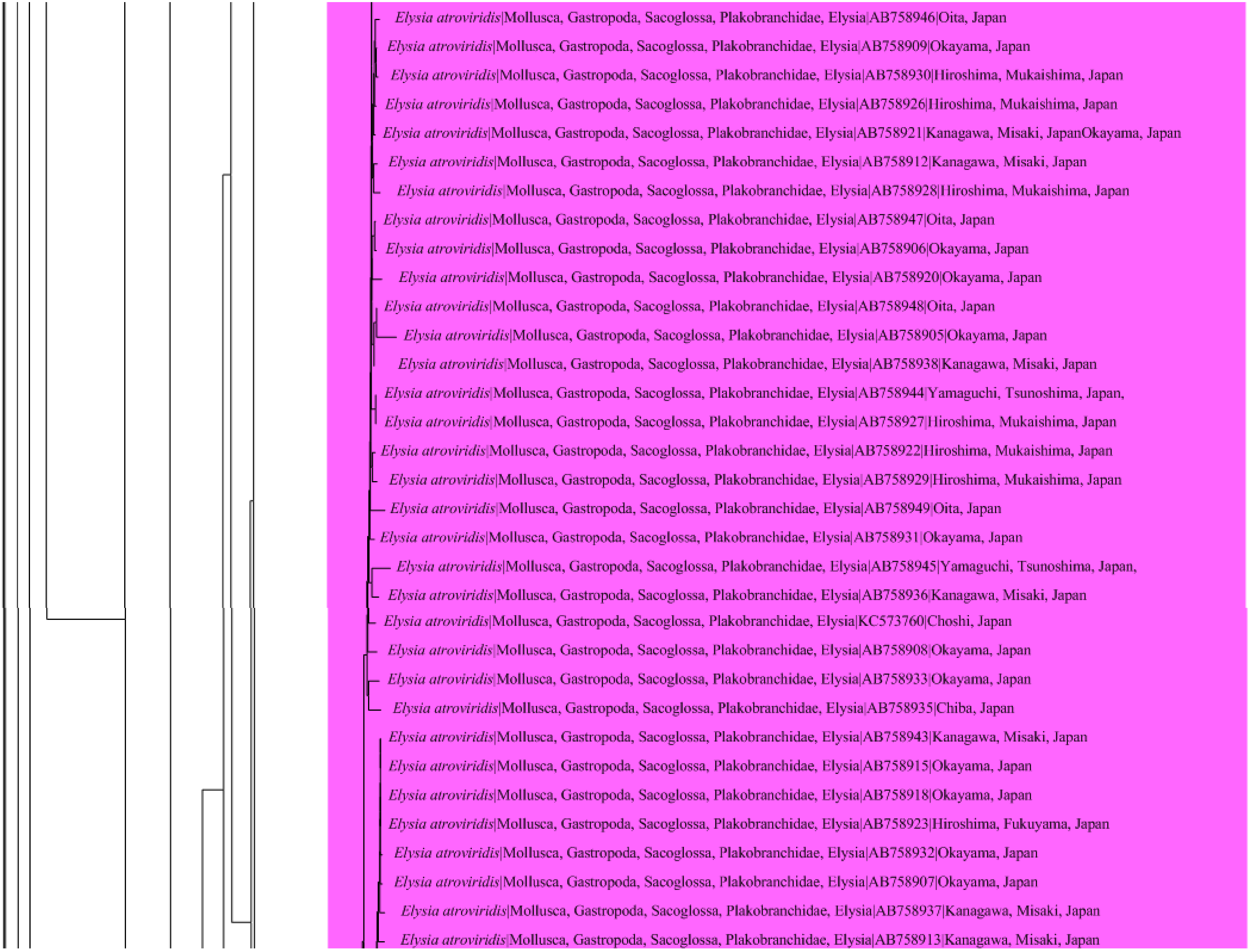

Elysia atroviridis|Mollusca, Gastropoda, Sacoglossa, Plakobranchidae, Elysia|AB758946|Oita, Japan
Elysia atroviridis|Mollusca, Gastropoda, Sacoglossa, Plakobranchidae, Elysia|AB758909|Okayama, Japan
Elysia atroviridis|Mollusca, Gastropoda, Sacoglossa, Plakobranchidae, Elysia|AB758930|Hiroshima, Mukaishima, Japan
Elysia atroviridis|Mollusca, Gastropoda, Sacoglossa, Plakobranchidae, Elysia|AB758926|Hiroshima, Mukaishima, Japan
Elysia atroviridis|Mollusca, Gastropoda, Sacoglossa, Plakobranchidae, Elysia|AB758921|Kanagawa, Misaki, JapanOkayama, Japan
Elysia atroviridis|Mollusca, Gastropoda, Sacoglossa, Plakobranchidae, Elysia|AB758912|Kanagawa, Misaki, Japan
Elysia atroviridis|Mollusca, Gastropoda, Sacoglossa, Plakobranchidae, Elysia|AB758928|Hiroshima, Mukaishima, Japan
Elysia atroviridis|Mollusca, Gastropoda, Sacoglossa, Plakobranchidae, Elysia|AB758947|Oita, Japan
Elysia atroviridis|Mollusca, Gastropoda, Sacoglossa, Plakobranchidae, Elysia|AB758906|Okayama, Japan
Elysia atroviridis|Mollusca, Gastropoda, Sacoglossa, Plakobranchidae, Elysia|AB758920|Okayama, Japan
Elysia atroviridis|Mollusca, Gastropoda, Sacoglossa, Plakobranchidae, Elysia|AB758948|Oita, Japan
Elysia atroviridis|Mollusca, Gastropoda, Sacoglossa, Plakobranchidae, Elysia|AB758905|Okayama, Japan
Elysia atroviridis|Mollusca, Gastropoda, Sacoglossa, Plakobranchidae, Elysia|AB758938|Kanagawa, Misaki, Japan
Elysia atroviridis|Mollusca, Gastropoda, Sacoglossa, Plakobranchidae, Elysia|AB758944|Yamaguchi, Tsunoshima, Japan,
Elysia atroviridis|Mollusca, Gastropoda, Sacoglossa, Plakobranchidae, Elysia|AB758927|Hiroshima, Mukaishima, Japan
Elysia atroviridis|Mollusca, Gastropoda, Sacoglossa, Plakobranchidae, Elysia|AB758922|Hiroshima, Mukaishima, Japan
Elysia atroviridis|Mollusca, Gastropoda, Sacoglossa, Plakobranchidae, Elysia|AB758929|Hiroshima, Mukaishima, Japan
Elysia atroviridis|Mollusca, Gastropoda, Sacoglossa, Plakobranchidae, Elysia|AB758949|Oita, Japan
Elysia atroviridis|Mollusca, Gastropoda, Sacoglossa, Plakobranchidae, Elysia|AB758931|Okayama, Japan
Elysia atroviridis|Mollusca, Gastropoda, Sacoglossa, Plakobranchidae, Elysia|AB758945|Yamaguchi, Tsunoshima, Japan,
Elysia atroviridis|Mollusca, Gastropoda, Sacoglossa, Plakobranchidae, Elysia|AB758936|Kanagawa, Misaki, Japan
Elysia atroviridis|Mollusca, Gastropoda, Sacoglossa, Plakobranchidae, Elysia|KC573760|Choshi, Japan
Elysia atroviridis|Mollusca, Gastropoda, Sacoglossa, Plakobranchidae, Elysia|AB758908|Okayama, Japan
Elysia atroviridis|Mollusca, Gastropoda, Sacoglossa, Plakobranchidae, Elysia|AB758933|Okayama, Japan
Elysia atroviridis|Mollusca, Gastropoda, Sacoglossa, Plakobranchidae, Elysia|AB758935|Chiba, Japan
Elysia atroviridis|Mollusca, Gastropoda, Sacoglossa, Plakobranchidae, Elysia|AB758943|Kanagawa, Misaki, Japan
Elysia atroviridis|Mollusca, Gastropoda, Sacoglossa, Plakobranchidae, Elysia|AB758915|Okayama, Japan
Elysia atroviridis|Mollusca, Gastropoda, Sacoglossa, Plakobranchidae, Elysia|AB758918|Okayama, Japan
Elysia atroviridis|Mollusca, Gastropoda, Sacoglossa, Plakobranchidae, Elysia|AB758923|Hiroshima, Fukuyama, Japan
Elysia atroviridis|Mollusca, Gastropoda, Sacoglossa, Plakobranchidae, Elysia|AB758932|Okayama, Japan
Elysia atroviridis|Mollusca, Gastropoda, Sacoglossa, Plakobranchidae, Elysia|AB758907|Okayama, Japan
Elysia atroviridis|Mollusca, Gastropoda, Sacoglossa, Plakobranchidae, Elysia|AB758937|Kanagawa, Misaki, Japan
Elysia atroviridis|Mollusca, Gastropoda, Sacoglossa, Plakobranchidae, Elysia|AB758913|Kanagawa, Misaki, Japan

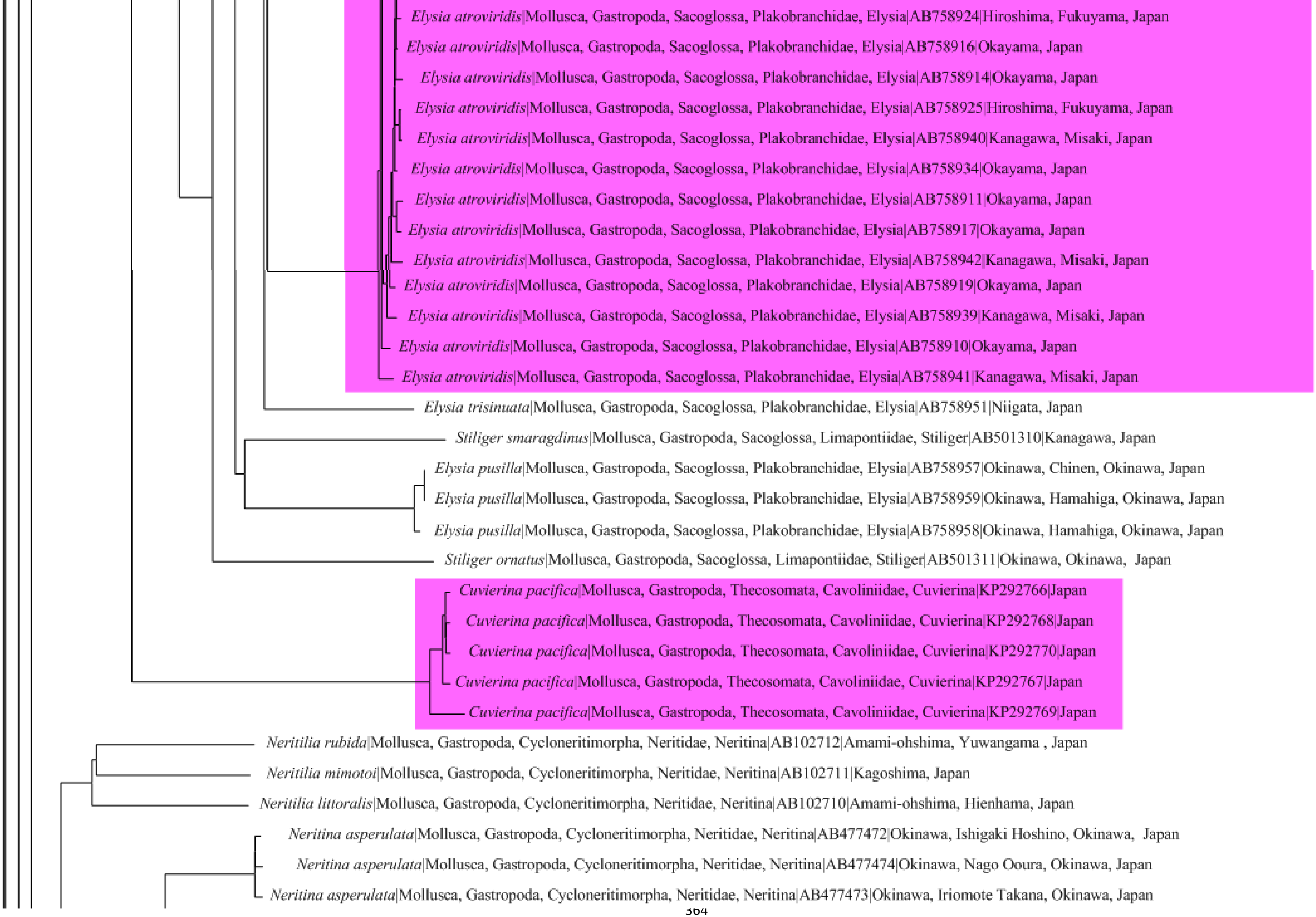

Elysia atroviridis|Mollusca, Gastropoda, Sacoglossa, Plakobranchidae, Elysia|AB758924|Hiroshima, Fukuyama, Japan
Elysia atroviridis|Mollusca, Gastropoda, Sacoglossa, Plakobranchidae, Elysia|AB758916|Okayama, Japan
Elysia atroviridis|Mollusca, Gastropoda, Sacoglossa, Plakobranchidae, Elysia|AB758914|Okayama, Japan
Elysia atroviridis|Mollusca, Gastropoda, Sacoglossa, Plakobranchidae, Elysia|AB758925|Hiroshima, Fukuyama, Japan
Elysia atroviridis|Mollusca, Gastropoda, Sacoglossa, Plakobranchidae, Elysia|AB758940|Kanagawa, Misaki, Japan
Elysia atroviridis|Mollusca, Gastropoda, Sacoglossa, Plakobranchidae, Elysia|AB758934|Okayama, Japan
Elysia atroviridis|Mollusca, Gastropoda, Sacoglossa, Plakobranchidae, Elysia|AB758911|Okayama, Japan
Elysia atroviridis|Mollusca, Gastropoda, Sacoglossa, Plakobranchidae, Elysia|AB758917|Okayama, Japan
Elysia atroviridis|Mollusca, Gastropoda, Sacoglossa, Plakobranchidae, Elysia|AB758942|Kanagawa, Misaki, Japan
Elysia atroviridis|Mollusca, Gastropoda, Sacoglossa, Plakobranchidae, Elysia|AB758919|Okayama, Japan
Elysia atroviridis|Mollusca, Gastropoda, Sacoglossa, Plakobranchidae, Elysia|AB758939|Kanagawa, Misaki, Japan
Elysia atroviridis|Mollusca, Gastropoda, Sacoglossa, Plakobranchidae, Elysia|AB758910|Okayama, Japan
Elysia atroviridis|Mollusca, Gastropoda, Sacoglossa, Plakobranchidae, Elysia|AB758941|Kanagawa, Misaki, Japan
Elysia trisinuata|Mollusca, Gastropoda, Sacoglossa, Plakobranchidae, Elysia|AB758951|Niigata, Japan
Stiliger smaragdinus|Mollusca, Gastropoda, Sacoglossa, Limapontiidae, Stiliger|AB501310|Kanagawa, Japan
Elysia pusilla|Mollusca, Gastropoda, Sacoglossa, Plakobranchidae, Elysia|AB758957|Okinawa, Chinen, Okinawa, Japan
Elysia pusilla|Mollusca, Gastropoda, Sacoglossa, Plakobranchidae, Elysia|AB758959|Okinawa, Hamahiga, Okinawa, Japan
Elysia pusilla|Mollusca, Gastropoda, Sacoglossa, Plakobranchidae, Elysia|AB758958|Okinawa, Hamahiga, Okinawa, Japan
Stiliger ornatus|Mollusca, Gastropoda, Sacoglossa, Limapontiidae, Stiliger|AB501311|Okinawa, Okinawa, Japan
Cuvierina pacifica|Mollusca, Gastropoda, Thecosomata, Cavoliniidae, Cuvierina|KP292766|Japan
Cuvierina pacifica|Mollusca, Gastropoda, Thecosomata, Cavoliniidae, Cuvierina|KP292768|Japan
Cuvierina pacifica|Mollusca, Gastropoda, Thecosomata, Cavoliniidae, Cuvierina|KP292770|Japan
Cuvierina pacifica|Mollusca, Gastropoda, Thecosomata, Cavoliniidae, Cuvierina|KP292767|Japan
Cuvierina pacifica|Mollusca, Gastropoda, Thecosomata, Cavoliniidae, Cuvierina|KP292769|Japan
Neritilia rubida|Mollusca, Gastropoda, Cycloneritimorpha, Neritidae, Neritina|AB102712|Amami-ohshima, Yuwangama , Japan
Neritilia mimotoi|Mollusca, Gastropoda, Cycloneritimorpha, Neritidae, Neritina|AB102711|Kagoshima, Japan
Neritilia littoralis|Mollusca, Gastropoda, Cycloneritimorpha, Neritidae, Neritina|AB102710|Amami-ohshima, Hienhama, Japan
Neritina asperulata|Mollusca, Gastropoda, Cycloneritimorpha, Neritidae, Neritina|AB477472|Okinawa, Ishigaki Hoshino, Okinawa, Japan
Neritina asperulata|Mollusca, Gastropoda, Cycloneritimorpha, Neritidae, Neritina|AB477474|Okinawa, Nago Ooura, Okinawa, Japan
Neritina asperulata|Mollusca, Gastropoda, Cycloneritimorpha, Neritidae, Neritina|AB477473|Okinawa, Iriomote Takana, Okinawa, Japan

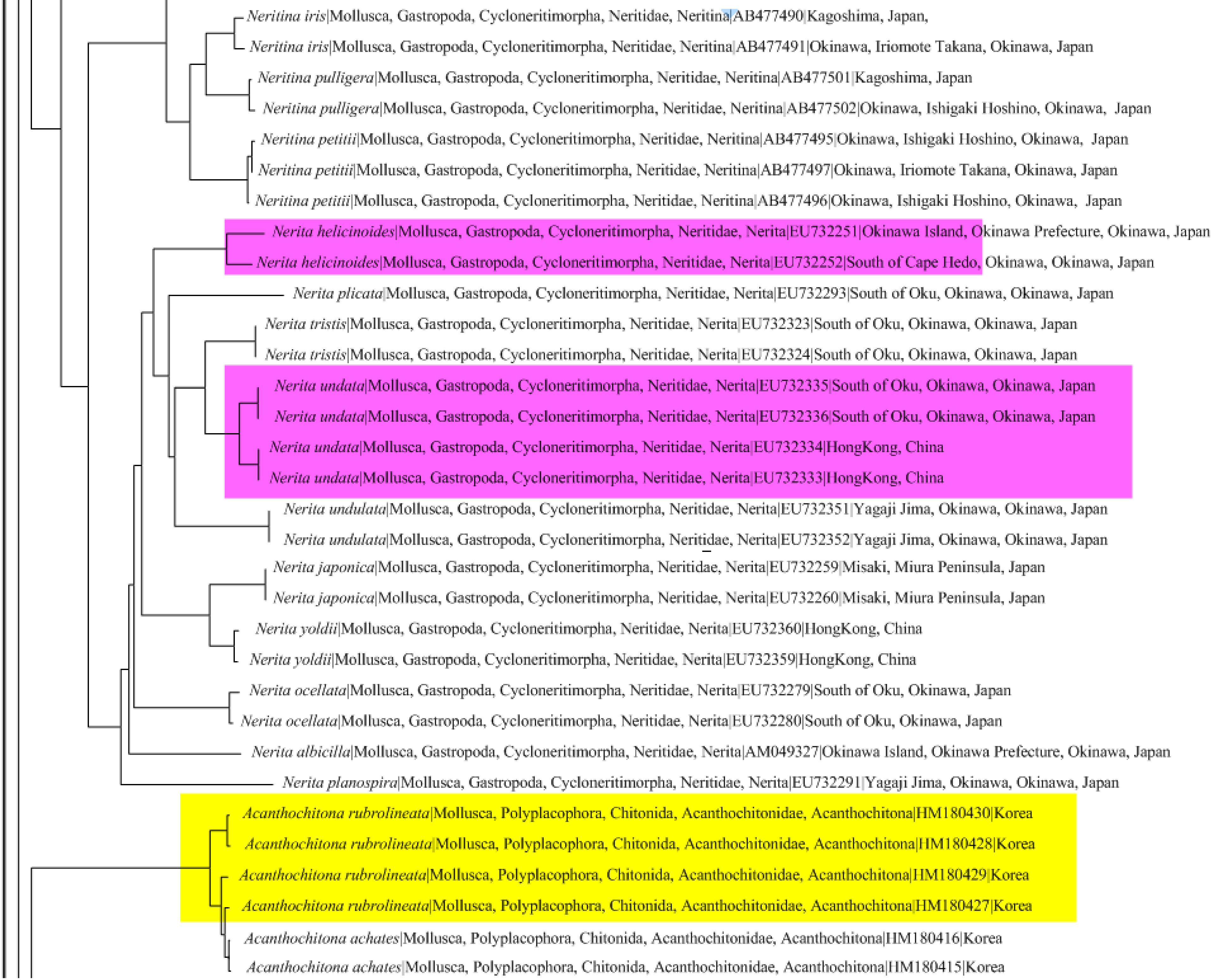
Neritina iris|Mollusca, Gastropoda, Cycloneritimorpha, Neritidae, Neritina|AB477490|Kagoshima, Japan,
Neritina iris|Mollusca, Gastropoda, Cycloneritimorpha, Neritidae, Neritina|AB477491|Okinawa, Iriomote Takana, Okinawa, Japan
Neritina pulligera|Mollusca, Gastropoda, Cycloneritimorpha, Neritidae, Neritina|AB477501|Kagoshima, Japan
Neritina pulligera|Mollusca, Gastropoda, Cycloneritimorpha, Neritidae, Neritina|AB477502|Okinawa, Ishigaki Hoshino, Okinawa, Japan
Neritina petitii|Mollusca, Gastropoda, Cycloneritimorpha, Neritidae, Neritina|AB477495|Okinawa, Ishigaki Hoshino, Okinawa, Japan
Neritina petitii|Mollusca, Gastropoda, Cycloneritimorpha, Neritidae, Neritina|AB477497|Okinawa, Iriomote Takana, Okinawa, Japan
Neritina petitii|Mollusca, Gastropoda, Cycloneritimorpha, Neritidae, Neritina|AB477496|Okinawa, Ishigaki Hoshino, Okinawa, Japan
Nerita helicinoides|Mollusca, Gastropoda, Cycloneritimorpha, Neritidae, Nerita|EU732251|Okinawa Island, Okinawa Prefecture, Okinawa, Japan
Nerita helicinoides|Mollusca, Gastropoda, Cycloneritimorpha, Neritidae, Nerita|EU732252|South of Cape Hedo, Okinawa, Okinawa, Japan
Nerita plicata|Mollusca, Gastropoda, Cycloneritimorpha, Neritidae, Nerita|EU732293|South of Oku, Okinawa, Okinawa, Japan
Nerita tristis|Mollusca, Gastropoda, Cycloneritimorpha, Neritidae, Nerita|EU732323|South of Oku, Okinawa, Okinawa, Japan
Nerita tristis|Mollusca, Gastropoda, Cycloneritimorpha, Neritidae, Nerita|EU732324|South of Oku, Okinawa, Okinawa, Japan
Nerita undata|Mollusca, Gastropoda, Cycloneritimorpha, Neritidae, Nerita|EU732335|South of Oku, Okinawa, Okinawa, Japan
Nerita undata|Mollusca, Gastropoda, Cycloneritimorpha, Neritidae, Nerita|EU732336|South of Oku, Okinawa, Okinawa, Japan
Nerita undata|Mollusca, Gastropoda, Cycloneritimorpha, Neritidae, Nerita|EU732334|HongKong, China
Nerita undata|Mollusca, Gastropoda, Cycloneritimorpha, Neritidae, Nerita|EU732333|HongKong, China
Nerita undulata|Mollusca, Gastropoda, Cycloneritimorpha, Neritidae, Nerita|EU732351|Yagaji Jima, Okinawa, Okinawa, Japan
Nerita undulata|Mollusca, Gastropoda, Cycloneritimorpha, Neritidae, Nerita|EU732352|Yagaji Jima, Okinawa, Okinawa, Japan
Nerita japonica|Mollusca, Gastropoda, Cycloneritimorpha, Neritidae, Nerita|EU732259|Misaki, Miura Peninsula, Japan
Nerita japonica|Mollusca, Gastropoda, Cycloneritimorpha, Neritidae, Nerita|EU732260|Misaki, Miura Peninsula, Japan
Nerita yoldii|Mollusca, Gastropoda, Cycloneritimorpha, Neritidae, Nerita|EU732360|HongKong, China
Nerita yoldii|Mollusca, Gastropoda, Cycloneritimorpha, Neritidae, Nerita|EU732359|HongKong, China
Nerita ocellata|Mollusca, Gastropoda, Cycloneritimorpha, Neritidae, Nerita|EU732279|South of Oku, Okinawa, Japan
Nerita ocellata|Mollusca, Gastropoda, Cycloneritimorpha, Neritidae, Nerita|EU732280|South of Oku, Okinawa, Japan
Nerita albicilla|Mollusca, Gastropoda, Cycloneritimorpha, Neritidae, Nerita|AM049327|Okinawa Island, Okinawa Prefecture, Okinawa, Japan
Nerita planospira|Mollusca, Gastropoda, Cycloneritimorpha, Neritidae, Nerita|EU732291|Yagaji Jima, Okinawa, Okinawa, Japan
Acanthochitona rubrolineata|Mollusca, Polyplacophora, Chitonida, Acanthochitonidae, Acanthochitona|HM180430|Korea
Acanthochitona rubrolineata|Mollusca, Polyplacophora, Chitonida, Acanthochitonidae, Acanthochitona|HM180428|Korea
Acanthochitona rubrolineata|Mollusca, Polyplacophora, Chitonida, Acanthochitonidae, Acanthochitona|HM180429|Korea
Acanthochitona rubrolineata|Mollusca, Polyplacophora, Chitonida, Acanthochitonidae, Acanthochitona|HM180427|Korea
Acanthochitona achates|Mollusca, Polyplacophora, Chitonida, Acanthochitonidae, Acanthochitona|HM180416|Korea
Acanthochitona achates|Mollusca, Polyplacophora, Chitonida, Acanthochitonidae, Acanthochitona|HM180415|Korea

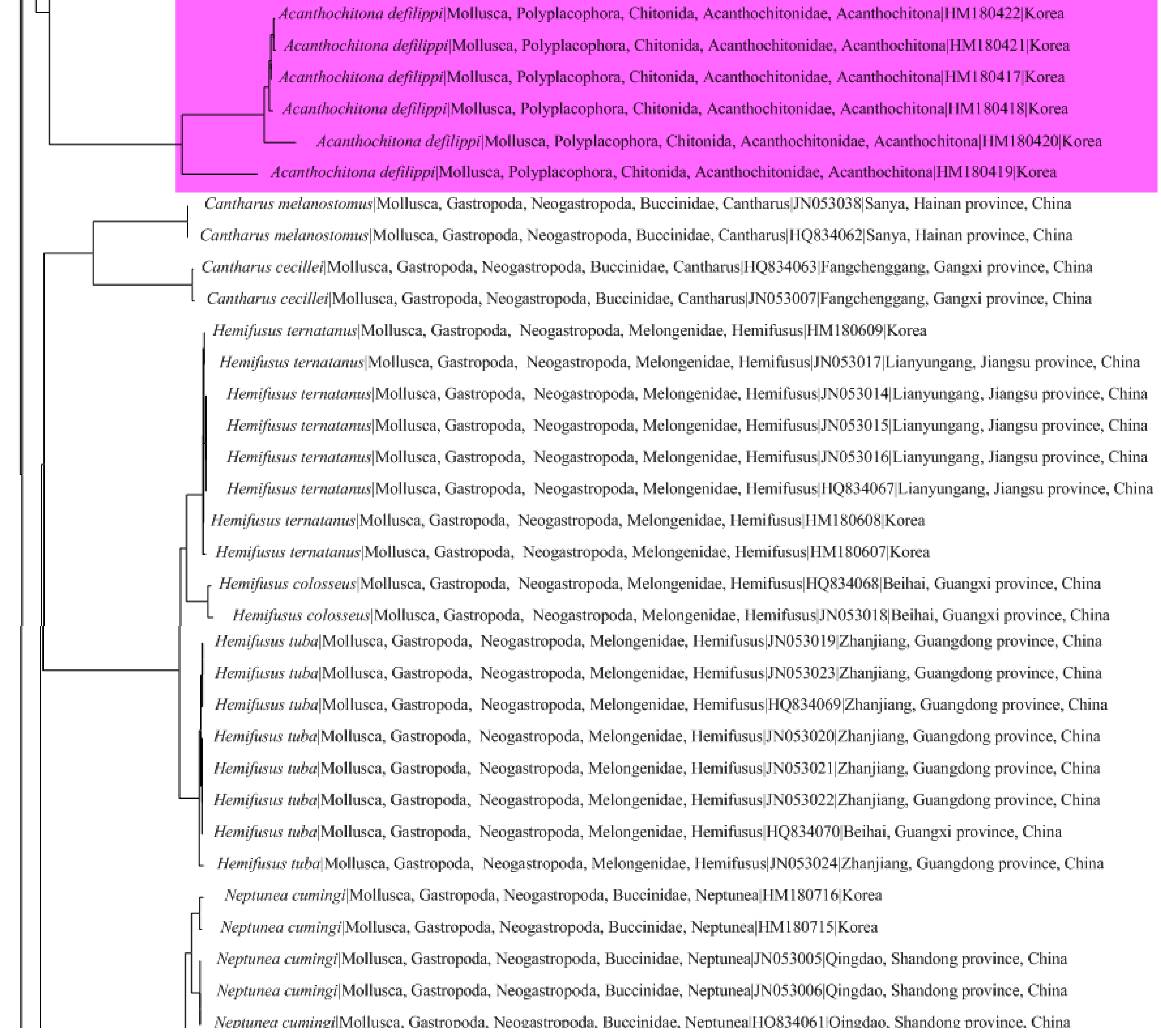
Acanthochitona defilippi|Mollusca, Polyplacophora, Chitonida, Acanthochitonidae, Acanthochitona|HM180422|Korea
Acanthochitona defilippi|Mollusca, Polyplacophora, Chitonida, Acanthochitonidae, Acanthochitona|HM180421|Korea
Acanthochitona defilippi|Mollusca, Polyplacophora, Chitonida, Acanthochitonidae, Acanthochitona|HM180417|Korea
Acanthochitona defilippi|Mollusca, Polyplacophora, Chitonida, Acanthochitonidae, Acanthochitona|HM180418|Korea
Acanthochitona defilippi|Mollusca, Polyplacophora, Chitonida, Acanthochitonidae, Acanthochitona|HM180420|Korea
Acanthochitona defilippi|Mollusca, Polyplacophora, Chitonida, Acanthochitonidae, Acanthochitona|HM180419|Korea
Cantharus melanostomus|Mollusca, Gastropoda, Neogastropoda, Buccinidae, Cantharus|JN053038|Sanya, Hainan province, China
Cantharus melanostomus|Mollusca, Gastropoda, Neogastropoda, Buccinidae, Cantharus|HQ834062|Sanya, Hainan province, China
Cantharus cecillei|Mollusca, Gastropoda, Neogastropoda, Buccinidae, Cantharus|HQ834063|Fangchenggang, Gangxi province, China
Cantharus cecillei|Mollusca, Gastropoda, Neogastropoda, Buccinidae, Cantharus|JN053007|Fangchenggang, Gangxi province, China
Hemifusus ternatanus|Mollusca, Gastropoda, Neogastropoda, Melongenidae, Hemifusus|HM180609|Korea
Hemifusus ternatanus|Mollusca, Gastropoda, Neogastropoda, Melongenidae, Hemifusus|JN053017|Lianyungang, Jiangsu province, China
Hemifusus ternatanus|Mollusca, Gastropoda, Neogastropoda, Melongenidae, Hemifusus|JN053014|Lianyungang, Jiangsu province, China
Hemifusus ternatanus|Mollusca, Gastropoda, Neogastropoda, Melongenidae, Hemifusus|JN053015|Lianyungang, Jiangsu province, China
Hemifusus ternatanus|Mollusca, Gastropoda, Neogastropoda, Melongenidae, Hemifusus|JN053016|Lianyungang, Jiangsu province, China
Hemifusus ternatanus|Mollusca, Gastropoda, Neogastropoda, Melongenidae, Hemifusus|HQ834067|Lianyungang, Jiangsu province, China
Hemifusus ternatanus|Mollusca, Gastropoda, Neogastropoda, Melongenidae, Hemifusus|HM180608|Korea
Hemifusus ternatanus|Mollusca, Gastropoda, Neogastropoda, Melongenidae, Hemifusus|HM180607|Korea
Hemifusus colosseus|Mollusca, Gastropoda, Neogastropoda, Melongenidae, Hemifusus|HQ834068|Beihai, Guangxi province, China
Hemifusus colosseus|Mollusca, Gastropoda, Neogastropoda, Melongenidae, Hemifusus|JN053018|Beihai, Guangxi province, China
Hemifusus tuba|Mollusca, Gastropoda, Neogastropoda, Melongenidae, Hemifusus|JN053019|Zhanjiang, Guangdong province, China
Hemifusus tuba|Mollusca, Gastropoda, Neogastropoda, Melongenidae, Hemifusus|JN053023|Zhanjiang, Guangdong province, China
Hemifusus tuba|Mollusca, Gastropoda, Neogastropoda, Melongenidae, Hemifusus|HQ834069|Zhanjiang, Guangdong province, China
Hemifusus tuba|Mollusca, Gastropoda, Neogastropoda, Melongenidae, Hemifusus|JN053020|Zhanjiang, Guangdong province, China
Hemifusus tuba|Mollusca, Gastropoda, Neogastropoda, Melongenidae, Hemifusus|JN053021|Zhanjiang, Guangdong province, China
Hemifusus tuba|Mollusca, Gastropoda, Neogastropoda, Melongenidae, Hemifusus|JN053022|Zhanjiang, Guangdong province, China
Hemifusus tuba|Mollusca, Gastropoda, Neogastropoda, Melongenidae, Hemifusus|HQ834070|Beihai, Guangxi province, China
Hemifusus tuba|Mollusca, Gastropoda, Neogastropoda, Melongenidae, Hemifusus|JN053024|Zhanjiang, Guangdong province, China
Neptunea cumingi|Mollusca, Gastropoda, Neogastropoda, Buccinidae, Neptunea|HM180716|Korea
Neptunea cumingi|Mollusca, Gastropoda, Neogastropoda, Buccinidae, Neptunea|HM180715|Korea
Neptunea cumingi|Mollusca, Gastropoda, Neogastropoda, Buccinidae, Neptunea|JN053005|Qingdao, Shandong province, China
Neptunea cumingi|Mollusca, Gastropoda, Neogastropoda, Buccinidae, Neptunea|JN053006|Qingdao, Shandong province, China
Neptunea cumingi|Mollusca, Gastropoda, Neogastropoda, Buccinidae, Neptunea|HQ834061|Qingdao, Shandong province, China

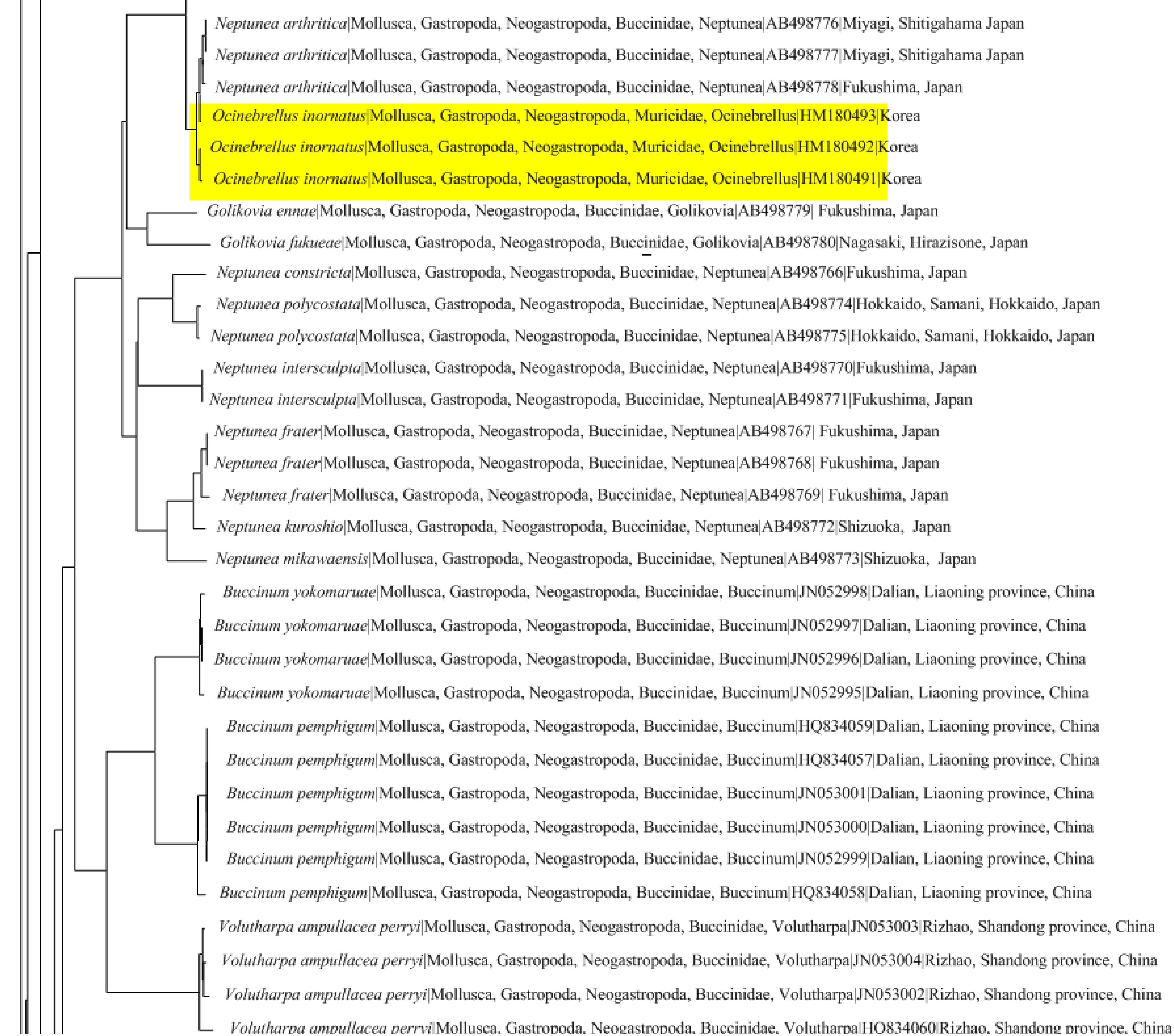

Neptunea arthritica|Mollusca, Gastropoda, Neogastropoda, Buccinidae, Neptunea|AB498776|Miyagi, Shitigahama Japan
Neptunea arthritica|Mollusca, Gastropoda, Neogastropoda, Buccinidae, Neptunea|AB498777|Miyagi, Shitigahama Japan
Neptunea arthritica|Mollusca, Gastropoda, Neogastropoda, Buccinidae, Neptunea|AB498778|Fukushima, Japan
Ocinebrellus inornatus|Mollusca, Gastropoda, Neogastropoda, Muricidae, Ocinebrellus|HM180493|Korea
Ocinebrellus inornatus|Mollusca, Gastropoda, Neogastropoda, Muricidae, Ocinebrellus|HM180492|Korea
Ocinebrellus inornatus|Mollusca, Gastropoda, Neogastropoda, Muricidae, Ocinebrellus|HM180491|Korea
Golikovia ennae|Mollusca, Gastropoda, Neogastropoda, Buccinidae, Golikovia|AB498779| Fukushima, Japan
Golikovia fukueae|Mollusca, Gastropoda, Neogastropoda, Buccinidae, Golikovia|AB498780|Nagasaki, Hirazisone, Japan
Neptunea constricta|Mollusca, Gastropoda, Neogastropoda, Buccinidae, Neptunea|AB498766|Fukushima, Japan
Neptunea polycostata|Mollusca, Gastropoda, Neogastropoda, Buccinidae, Neptunea|AB498774|Hokkaido, Samani, Hokkaido, Japan
Neptunea polycostata|Mollusca, Gastropoda, Neogastropoda, Buccinidae, Neptunea|AB498775|Hokkaido, Samani, Hokkaido, Japan
Neptunea intersculpta|Mollusca, Gastropoda, Neogastropoda, Buccinidae, Neptunea|AB498770|Fukushima, Japan
Neptunea intersculpta|Mollusca, Gastropoda, Neogastropoda, Buccinidae, Neptunea|AB498771|Fukushima, Japan
Neptunea frater|Mollusca, Gastropoda, Neogastropoda, Buccinidae, Neptunea|AB498767| Fukushima, Japan
Neptunea frater|Mollusca, Gastropoda, Neogastropoda, Buccinidae, Neptunea|AB498768| Fukushima, Japan
Neptunea frater|Mollusca, Gastropoda, Neogastropoda, Buccinidae, Neptunea|AB498769| Fukushima, Japan
Neptunea kuroshio|Mollusca, Gastropoda, Neogastropoda, Buccinidae, Neptunea|AB498772|Shizuoka, Japan
Neptunea mikawaensis|Mollusca, Gastropoda, Neogastropoda, Buccinidae, Neptunea|AB498773|Shizuoka, Japan
Buccinum yokomaruae|Mollusca, Gastropoda, Neogastropoda, Buccinidae, Buccinum|JN052998|Dalian, Liaoning province, China
Buccinum yokomaruae|Mollusca, Gastropoda, Neogastropoda, Buccinidae, Buccinum|JN052997|Dalian, Liaoning province, China
Buccinum yokomaruae|Mollusca, Gastropoda, Neogastropoda, Buccinidae, Buccinum|JN052996|Dalian, Liaoning province, China
Buccinum yokomaruae|Mollusca, Gastropoda, Neogastropoda, Buccinidae, Buccinum|JN052995|Dalian, Liaoning province, China
Buccinum pemphigum|Mollusca, Gastropoda, Neogastropoda, Buccinidae, Buccinum|HQ834059|Dalian, Liaoning province, China
Buccinum pemphigum|Mollusca, Gastropoda, Neogastropoda, Buccinidae, Buccinum|HQ834057|Dalian, Liaoning province, China
Buccinum pemphigum|Mollusca, Gastropoda, Neogastropoda, Buccinidae, Buccinum|JN053001|Dalian, Liaoning province, China
Buccinum pemphigum|Mollusca, Gastropoda, Neogastropoda, Buccinidae, Buccinum|JN053000|Dalian, Liaoning province, China
Buccinum pemphigum|Mollusca, Gastropoda, Neogastropoda, Buccinidae, Buccinum|JN052999|Dalian, Liaoning province, China
Buccinum pemphigum|Mollusca, Gastropoda, Neogastropoda, Buccinidae, Buccinum|HQ834058|Dalian, Liaoning province, China
Volutharpa ampullacea perryi|Mollusca, Gastropoda, Neogastropoda, Buccinidae, Volutharpa|JN053003|Rizhao, Shandong province, China
Volutharpa ampullacea perryi|Mollusca, Gastropoda, Neogastropoda, Buccinidae, Volutharpa|JN053004|Rizhao, Shandong province, China
Volutharpa ampullacea perryi|Mollusca, Gastropoda, Neogastropoda, Buccinidae, Volutharpa|JN053002|Rizhao, Shandong province, China
Volutharpa ampullacea perryi|Mollusca, Gastropoda, Neogastropoda, Buccinidae, Volutharpa|HQ834060|Rizhao, Shandong province, China

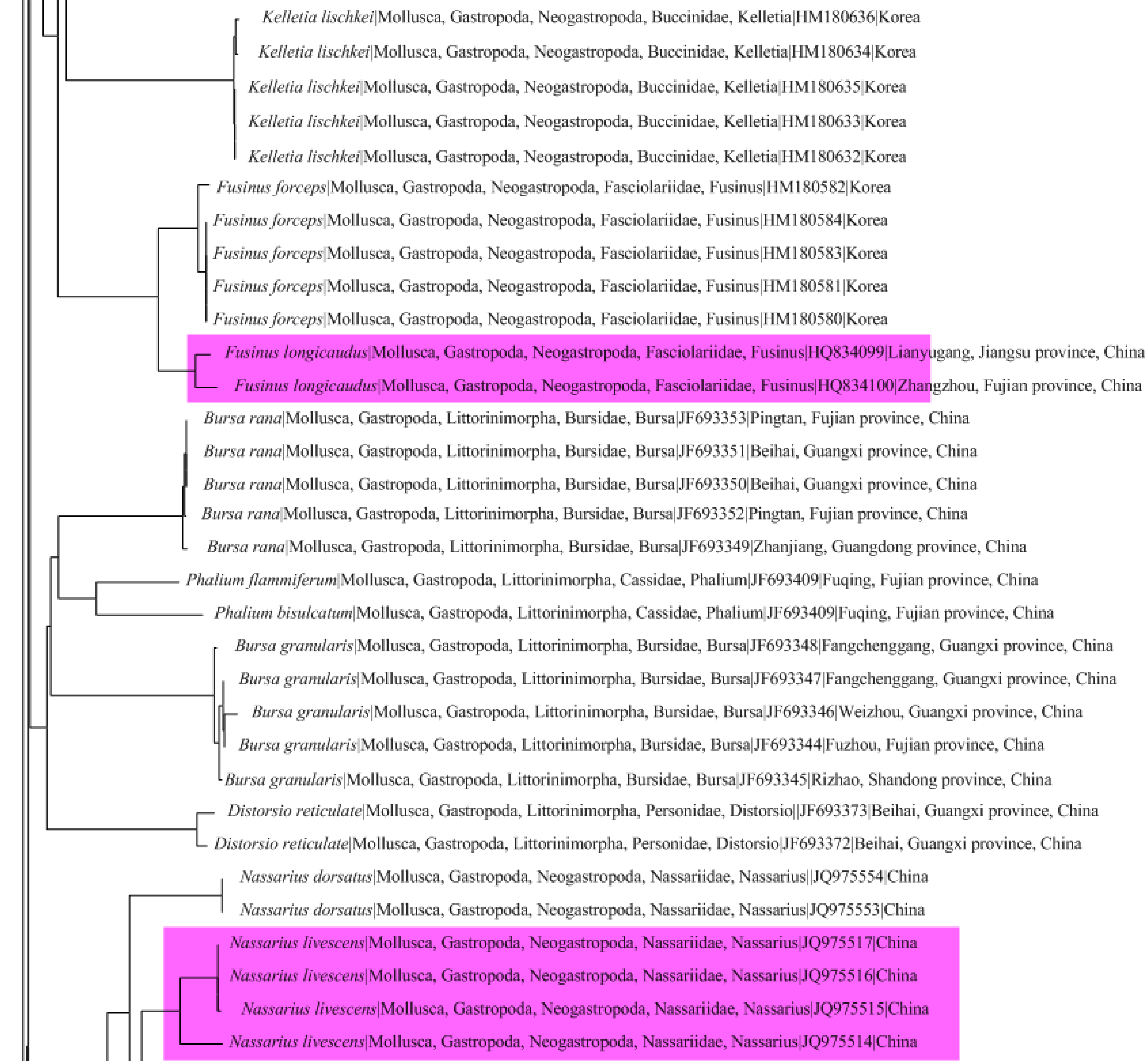
Kelletia lischkei|Mollusca, Gastropoda, Neogastropoda, Buccinidae, Kelletia|HM180636|Korea
Kelletia lischkei|Mollusca, Gastropoda, Neogastropoda, Buccinidae, Kelletia|HM180634|Korea
Kelletia lischkei|Mollusca, Gastropoda, Neogastropoda, Buccinidae, Kelletia|HM180635|Korea
Kelletia lischkei|Mollusca, Gastropoda, Neogastropoda, Buccinidae, Kelletia|HM180633|Korea
Kelletia lischkei|Mollusca, Gastropoda, Neogastropoda, Buccinidae, Kelletia|HM180632|Korea
Fusinus forceps|Mollusca, Gastropoda, Neogastropoda, Fasciolariidae, Fusinus|HM180582|Korea
Fusinus forceps|Mollusca, Gastropoda, Neogastropoda, Fasciolariidae, Fusinus|HM180584|Korea
Fusinus forceps|Mollusca, Gastropoda, Neogastropoda, Fasciolariidae, Fusinus|HM180583|Korea
Fusinus forceps|Mollusca, Gastropoda, Neogastropoda, Fasciolariidae, Fusinus|HM180581|Korea
Fusinus forceps|Mollusca, Gastropoda, Neogastropoda, Fasciolariidae, Fusinus|HM180580|Korea
Fusinus longicaudus|Mollusca, Gastropoda, Neogastropoda, Fasciolariidae, Fusinus|HQ834099|Lianyugang, Jiangsu province, China
Fusinus longicaudus|Mollusca, Gastropoda, Neogastropoda, Fasciolariidae, Fusinus|HQ834100|Zhangzhou, Fujian province, China
Bursa rana|Mollusca, Gastropoda, Littorinimorpha, Bursidae, Bursa|JF693353|Pingtan, Fujian province, China
Bursa rana|Mollusca, Gastropoda, Littorinimorpha, Bursidae, Bursa|JF693351|Beihai, Guangxi province, China
Bursa rana|Mollusca, Gastropoda, Littorinimorpha, Bursidae, Bursa|JF693350|Beihai, Guangxi province, China
Bursa rana|Mollusca, Gastropoda, Littorinimorpha, Bursidae, Bursa|JF693352|Pingtan, Fujian province, China
Bursa rana|Mollusca, Gastropoda, Littorinimorpha, Bursidae, Bursa|JF693349|Zhanjiang, Guangdong province, China
Phalium flammiferum|Mollusca, Gastropoda, Littorinimorpha, Cassidae, Phalium|JF693409|Fuqing, Fujian province, China
Phalium bisulcatum|Mollusca, Gastropoda, Littorinimorpha, Cassidae, Phalium|JF693409|Fuqing, Fujian province, China
Bursa granularis|Mollusca, Gastropoda, Littorinimorpha, Bursidae, Bursa|JF693348|Fangchenggang, Guangxi province, China
Bursa granularis|Mollusca, Gastropoda, Littorinimorpha, Bursidae, Bursa|JF693347|Fangchenggang, Guangxi province, China
Bursa granularis|Mollusca, Gastropoda, Littorinimorpha, Bursidae, Bursa|JF693346|Weizhou, Guangxi province, China
Bursa granularis|Mollusca, Gastropoda, Littorinimorpha, Bursidae, Bursa|JF693344|Fuzhou, Fujian province, China
Bursa granularis|Mollusca, Gastropoda, Littorinimorpha, Bursidae, Bursa|JF693345|Rizhao, Shandong province, China
Distorsio reticulate|Mollusca, Gastropoda, Littorinimorpha, Personidae, Distorsio||JF693373|Beihai, Guangxi province, China
Distorsio reticulate|Mollusca, Gastropoda, Littorinimorpha, Personidae, Distorsio|JF693372|Beihai, Guangxi province, China
Nassarius dorsatus|Mollusca, Gastropoda, Neogastropoda, Nassariidae, Nassarius||JQ975554|China
Nassarius dorsatus|Mollusca, Gastropoda, Neogastropoda, Nassariidae, Nassarius|JQ975553|China
Nassarius livescens|Mollusca, Gastropoda, Neogastropoda, Nassariidae, Nassarius|JQ975517|China
Nassarius livescens|Mollusca, Gastropoda, Neogastropoda, Nassariidae, Nassarius|JQ975516|China
Nassarius livescens|Mollusca, Gastropoda, Neogastropoda, Nassariidae, Nassarius|JQ975515|China
Nassarius livescens|Mollusca, Gastropoda, Neogastropoda, Nassariidae, Nassarius|JQ975514|China

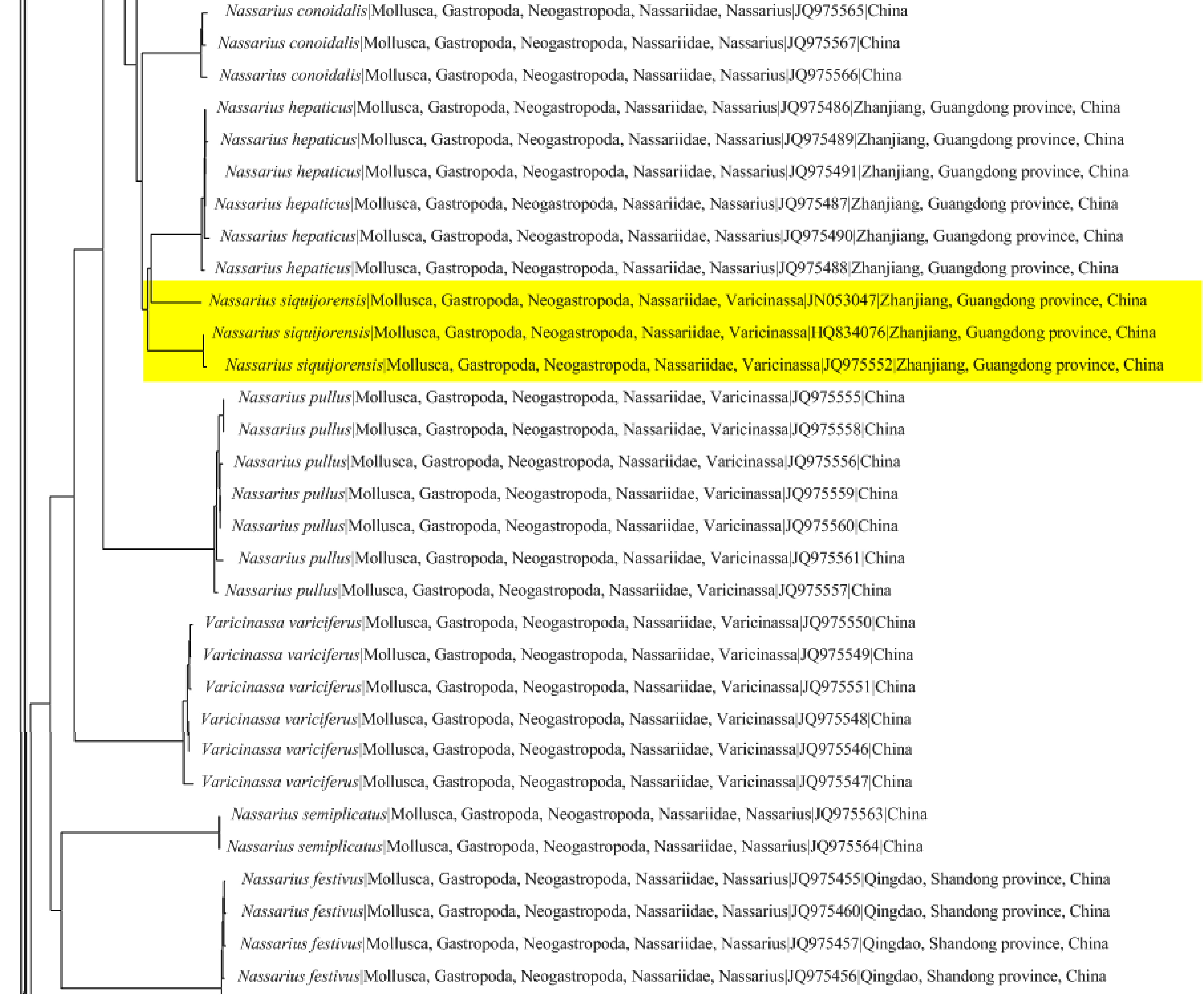
Nassarius conoidalis|Mollusca, Gastropoda, Neogastropoda, Nassariidae, Nassarius|JQ975565|China
Nassarius conoidalis|Mollusca, Gastropoda, Neogastropoda, Nassariidae, Nassarius|JQ975567|China
Nassarius conoidalis|Mollusca, Gastropoda, Neogastropoda, Nassariidae, Nassarius|JQ975566|China
Nassarius hepaticus|Mollusca, Gastropoda, Neogastropoda, Nassariidae, Nassarius|JQ975486|Zhanjiang, Guangdong province, China
Nassarius hepaticus|Mollusca, Gastropoda, Neogastropoda, Nassariidae, Nassarius|JQ975489|Zhanjiang, Guangdong province, China
Nassarius hepaticus|Mollusca, Gastropoda, Neogastropoda, Nassariidae, Nassarius|JQ975491|Zhanjiang, Guangdong province, China
Nassarius hepaticus|Mollusca, Gastropoda, Neogastropoda, Nassariidae, Nassarius|JQ975487|Zhanjiang, Guangdong province, China
Nassarius hepaticus|Mollusca, Gastropoda, Neogastropoda, Nassariidae, Nassarius|JQ975490|Zhanjiang, Guangdong province, China
Nassarius hepaticus|Mollusca, Gastropoda, Neogastropoda, Nassariidae, Nassarius|JQ975488|Zhanjiang, Guangdong province, China
Nassarius siquijorensis|Mollusca, Gastropoda, Neogastropoda, Nassariidae, Varicinassa|JN053047|Zhanjiang, Guangdong province, China
Nassarius siquijorensis|Mollusca, Gastropoda, Neogastropoda, Nassariidae, Varicinassa|HQ834076|Zhanjiang, Guangdong province, China
Nassarius siquijorensis|Mollusca, Gastropoda, Neogastropoda, Nassariidae, Varicinassa|JQ975552|Zhanjiang, Guangdong province, China
Nassarius pullus|Mollusca, Gastropoda, Neogastropoda, Nassariidae, Varicinassa|JQ975555|China
Nassarius pullus|Mollusca, Gastropoda, Neogastropoda, Nassariidae, Varicinassa|JQ975558|China
Nassarius pullus|Mollusca, Gastropoda, Neogastropoda, Nassariidae, Varicinassa|JQ975556|China
Nassarius pullus|Mollusca, Gastropoda, Neogastropoda, Nassariidae, Varicinassa|JQ975559|China
Nassarius pullus|Mollusca, Gastropoda, Neogastropoda, Nassariidae, Varicinassa|JQ975560|China
Nassarius pullus|Mollusca, Gastropoda, Neogastropoda, Nassariidae, Varicinassa|JQ975561|China
Nassarius pullus|Mollusca, Gastropoda, Neogastropoda, Nassariidae, Varicinassa|JQ975557|China
Varicinassa variciferus|Mollusca, Gastropoda, Neogastropoda, Nassariidae, Varicinassa|JQ975550|China
Varicinassa variciferus|Mollusca, Gastropoda, Neogastropoda, Nassariidae, Varicinassa|JQ975549|China
Varicinassa variciferus|Mollusca, Gastropoda, Neogastropoda, Nassariidae, Varicinassa|JQ975551|China
Varicinassa variciferus|Mollusca, Gastropoda, Neogastropoda, Nassariidae, Varicinassa|JQ975548|China
Varicinassa variciferus|Mollusca, Gastropoda, Neogastropoda, Nassariidae, Varicinassa|JQ975546|China
Varicinassa variciferus|Mollusca, Gastropoda, Neogastropoda, Nassariidae, Varicinassa|JQ975547|China
Nassarius semiplicatus|Mollusca, Gastropoda, Neogastropoda, Nassariidae, Nassarius|JQ975563|China
Nassarius semiplicatus|Mollusca, Gastropoda, Neogastropoda, Nassariidae, Nassarius|JQ975564|China
Nassarius festivus|Mollusca, Gastropoda, Neogastropoda, Nassariidae, Nassarius|JQ975455|Qingdao, Shandong province, China
Nassarius festivus|Mollusca, Gastropoda, Neogastropoda, Nassariidae, Nassarius|JQ975460|Qingdao, Shandong province, China
Nassarius festivus|Mollusca, Gastropoda, Neogastropoda, Nassariidae, Nassarius|JQ975457|Qingdao, Shandong province, China
Nassarius festivus|Mollusca, Gastropoda, Neogastropoda, Nassariidae, Nassarius|JQ975456|Qingdao, Shandong province, China

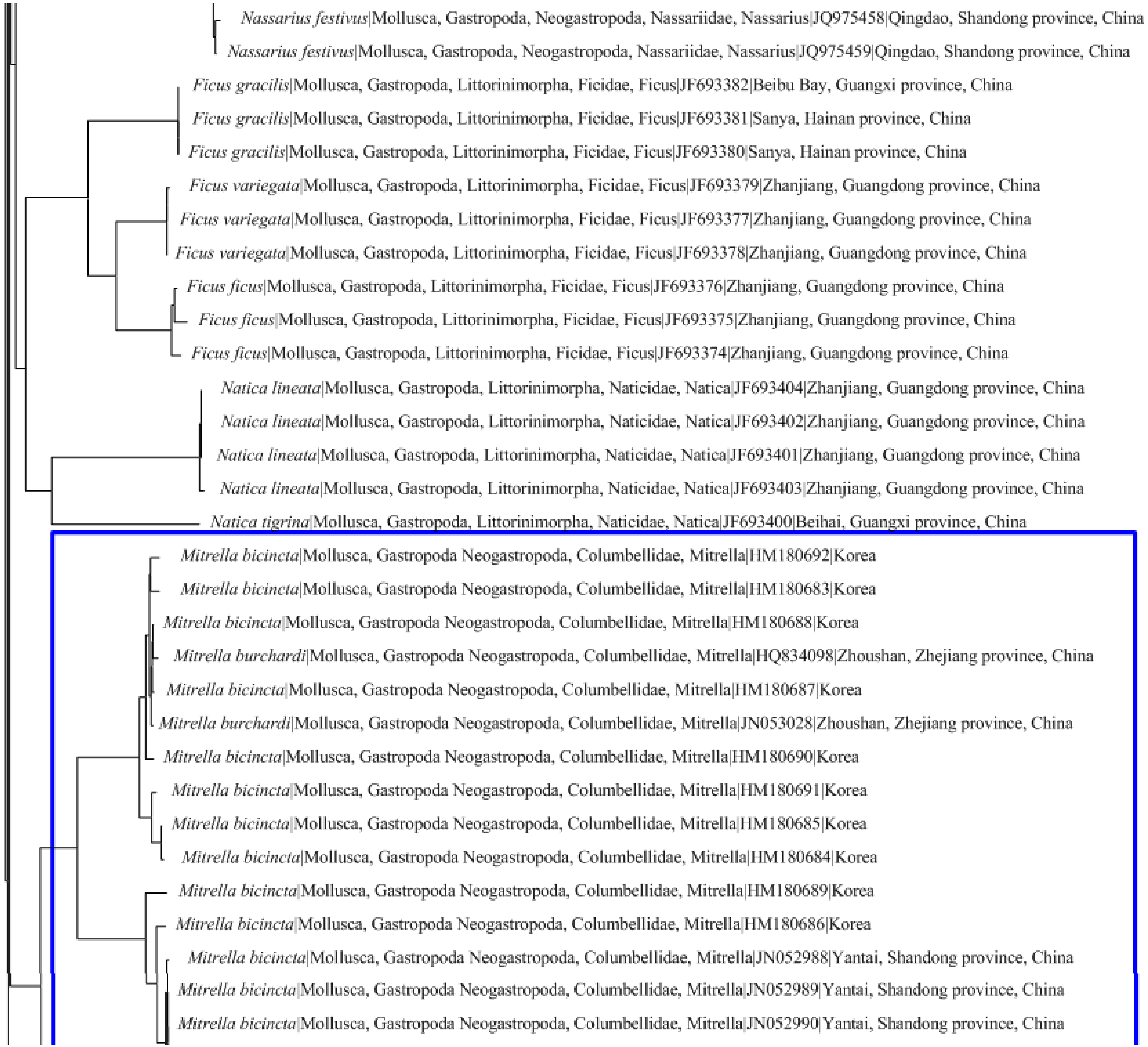

Nassarius festivus|Mollusca, Gastropoda, Neogastropoda, Nassariidae, Nassarius|JQ975458|Qingdao, Shandong province, China
Nassarius festivus|Mollusca, Gastropoda, Neogastropoda, Nassariidae, Nassarius|JQ975459|Qingdao, Shandong province, China
Ficus gracilis|Mollusca, Gastropoda, Littorinimorpha, Ficidae, Ficus|JF693382|Beibu Bay, Guangxi province, China
Ficus gracilis|Mollusca, Gastropoda, Littorinimorpha, Ficidae, Ficus|JF693381|Sanya, Hainan province, China
Ficus gracilis|Mollusca, Gastropoda, Littorinimorpha, Ficidae, Ficus|JF693380|Sanya, Hainan province, China
Ficus variegata|Mollusca, Gastropoda, Littorinimorpha, Ficidae, Ficus|JF693379|Zhanjiang, Guangdong province, China
Ficus variegata|Mollusca, Gastropoda, Littorinimorpha, Ficidae, Ficus|JF693377|Zhanjiang, Guangdong province, China
Ficus variegata|Mollusca, Gastropoda, Littorinimorpha, Ficidae, Ficus|JF693378|Zhanjiang, Guangdong province, China
Ficus ficus|Mollusca, Gastropoda, Littorinimorpha, Ficidae, Ficus|JF693376|Zhanjiang, Guangdong province, China
Ficus ficus|Mollusca, Gastropoda, Littorinimorpha, Ficidae, Ficus|JF693375|Zhanjiang, Guangdong province, China
Ficus ficus|Mollusca, Gastropoda, Littorinimorpha, Ficidae, Ficus|JF693374|Zhanjiang, Guangdong province, China
Natica lineata|Mollusca, Gastropoda, Littorinimorpha, Naticidae, Natica|JF693404|Zhanjiang, Guangdong province, China
Natica lineata|Mollusca, Gastropoda, Littorinimorpha, Naticidae, Natica|JF693402|Zhanjiang, Guangdong province, China
Natica lineata|Mollusca, Gastropoda, Littorinimorpha, Naticidae, Natica|JF693401|Zhanjiang, Guangdong province, China
Natica lineata|Mollusca, Gastropoda, Littorinimorpha, Naticidae, Natica|JF693403|Zhanjiang, Guangdong province, China
Natica tigrina|Mollusca, Gastropoda, Littorinimorpha, Naticidae, Natica|JF693400|Beihai, Guangxi province, China
Mitrella bicincta|Mollusca, Gastropoda Neogastropoda, Columbellidae, Mitrella|HM180692|Korea
Mitrella bicincta|Mollusca, Gastropoda Neogastropoda, Columbellidae, Mitrella|HM180683|Korea
Mitrella bicincta|Mollusca, Gastropoda Neogastropoda, Columbellidae, Mitrella|HM180688|Korea
Mitrella burchardi|Mollusca, Gastropoda Neogastropoda, Columbellidae, Mitrella|HQ834098|Zhoushan, Zhejiang province, China
Mitrella bicincta|Mollusca, Gastropoda Neogastropoda, Columbellidae, Mitrella|HM180687|Korea
Mitrella burchardi|Mollusca, Gastropoda Neogastropoda, Columbellidae, Mitrella|JN053028|Zhoushan, Zhejiang province, China
Mitrella bicincta|Mollusca, Gastropoda Neogastropoda, Columbellidae, Mitrella|HM180690|Korea
Mitrella bicincta|Mollusca, Gastropoda Neogastropoda, Columbellidae, Mitrella|HM180691|Korea
Mitrella bicincta|Mollusca, Gastropoda Neogastropoda, Columbellidae, Mitrella|HM180685|Korea
Mitrella bicincta|Mollusca, Gastropoda Neogastropoda, Columbellidae, Mitrella|HM180684|Korea
Mitrella bicincta|Mollusca, Gastropoda Neogastropoda, Columbellidae, Mitrella|HM180689|Korea
Mitrella bicincta|Mollusca, Gastropoda Neogastropoda, Columbellidae, Mitrella|HM180686|Korea
Mitrella bicincta|Mollusca, Gastropoda Neogastropoda, Columbellidae, Mitrella|JN052988|Yantai, Shandong province, China
Mitrella bicincta|Mollusca, Gastropoda Neogastropoda, Columbellidae, Mitrella|JN052989|Yantai, Shandong province, China
Mitrella bicincta|Mollusca, Gastropoda Neogastropoda, Columbellidae, Mitrella|JN052990|Yantai, Shandong province, China

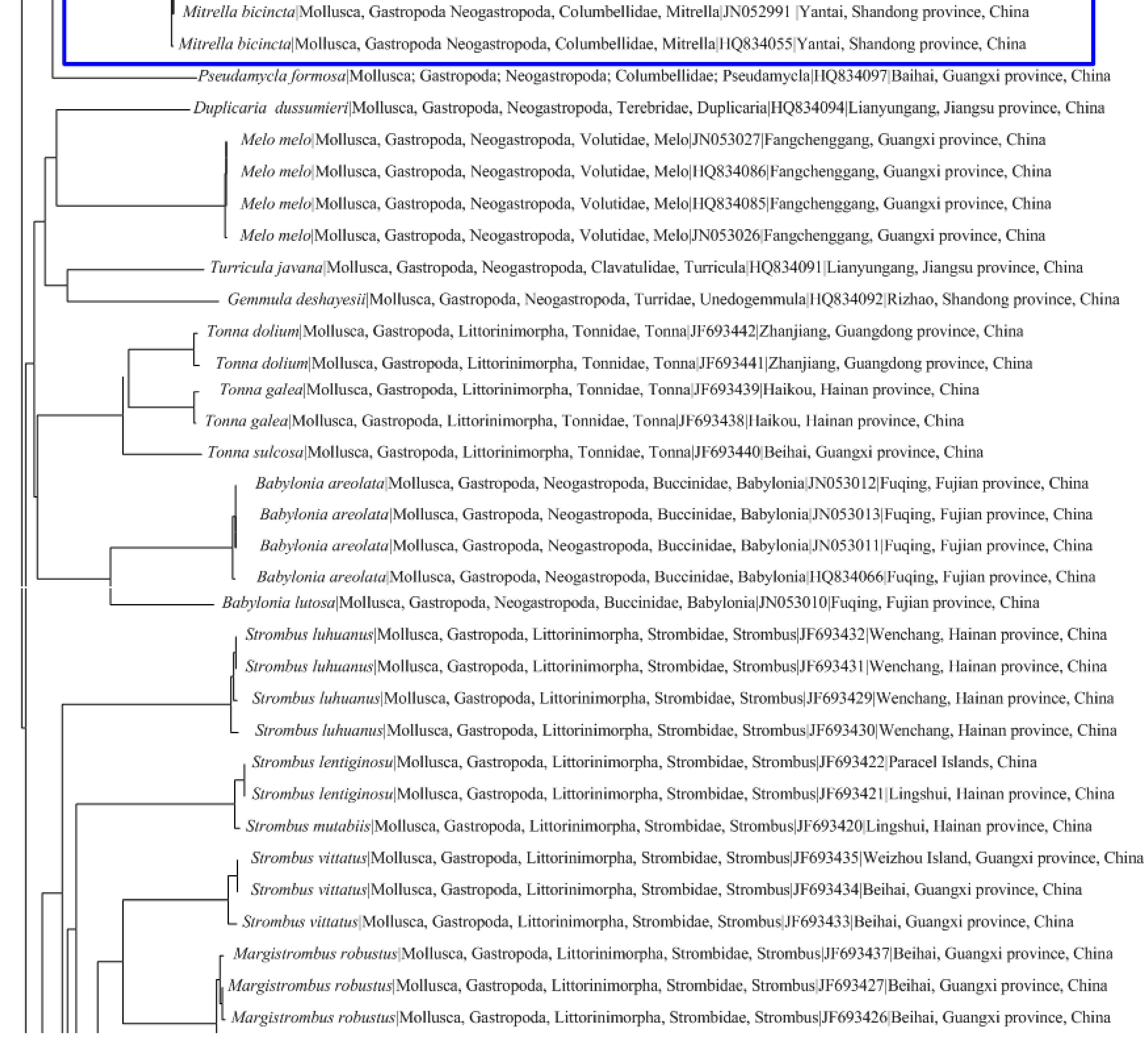
Mitrella bicincta|Mollusca, Gastropoda Neogastropoda, Columbellidae, Mitrella|JN052991 |Yantai, Shandong province, China
Mitrella bicincta|Mollusca, Gastropoda Neogastropoda, Columbellidae, Mitrella|HQ834055|Yantai, Shandong province, China
Pseudamycla formosa|Mollusca; Gastropoda; Neogastropoda; Columbellidae; Pseudamycla|HQ834097|Baihai, Guangxi province, China
Duplicaria dussumieri|Mollusca, Gastropoda, Neogastropoda, Terebridae, Duplicaria|HQ834094|Lianyungang, Jiangsu province, China
Melo melo|Mollusca, Gastropoda, Neogastropoda, Volutidae, Melo|JN053027|Fangchenggang, Guangxi province, China
Melo melo|Mollusca, Gastropoda, Neogastropoda, Volutidae, Melo|HQ834086|Fangchenggang, Guangxi province, China
Melo melo|Mollusca, Gastropoda, Neogastropoda, Volutidae, Melo|HQ834085|Fangchenggang, Guangxi province, China
Melo melo|Mollusca, Gastropoda, Neogastropoda, Volutidae, Melo|JN053026|Fangchenggang, Guangxi province, China
Turricula javana|Mollusca, Gastropoda, Neogastropoda, Clavatulidae, Turricula|HQ834091|Lianyungang, Jiangsu province, China
Gemmula deshayesii|Mollusca, Gastropoda, Neogastropoda, Turridae, Unedogemmula|HQ834092|Rizhao, Shandong province, China
Tonna dolium|Mollusca, Gastropoda, Littorinimorpha, Tonnidae, Tonna|JF693442|Zhanjiang, Guangdong province, China
Tonna dolium|Mollusca, Gastropoda, Littorinimorpha, Tonnidae, Tonna|JF693441|Zhanjiang, Guangdong province, China
Tonna galea|Mollusca, Gastropoda, Littorinimorpha, Tonnidae, Tonna|JF693439|Haikou, Hainan province, China
Tonna galea|Mollusca, Gastropoda, Littorinimorpha, Tonnidae, Tonna|JF693438|Haikou, Hainan province, China
Tonna sulcosa|Mollusca, Gastropoda, Littorinimorpha, Tonnidae, Tonna|JF693440|Beihai, Guangxi province, China
Babylonia areolata|Mollusca, Gastropoda, Neogastropoda, Buccinidae, Babylonia|JN053012|Fuqing, Fujian province, China
Babylonia areolata|Mollusca, Gastropoda, Neogastropoda, Buccinidae, Babylonia|JN053013|Fuqing, Fujian province, China
Babylonia areolata|Mollusca, Gastropoda, Neogastropoda, Buccinidae, Babylonia|JN053011|Fuqing, Fujian province, China
Babylonia areolata|Mollusca, Gastropoda, Neogastropoda, Buccinidae, Babylonia|HQ834066|Fuqing, Fujian province, China
Babylonia lutosa|Mollusca, Gastropoda, Neogastropoda, Buccinidae, Babylonia|JN053010|Fuqing, Fujian province, China
Strombus luhuanus|Mollusca, Gastropoda, Littorinimorpha, Strombidae, Strombus|JF693432|Wenchang, Hainan province, China
Strombus luhuanus|Mollusca, Gastropoda, Littorinimorpha, Strombidae, Strombus|JF693431|Wenchang, Hainan province, China
Strombus luhuanus|Mollusca, Gastropoda, Littorinimorpha, Strombidae, Strombus|JF693429|Wenchang, Hainan province, China
Strombus luhuanus|Mollusca, Gastropoda, Littorinimorpha, Strombidae, Strombus|JF693430|Wenchang, Hainan province, China
Strombus lentiginosu|Mollusca, Gastropoda, Littorinimorpha, Strombidae, Strombus|JF693422|Paracel Islands, China
Strombus lentiginosu|Mollusca, Gastropoda, Littorinimorpha, Strombidae, Strombus|JF693421|Lingshui, Hainan province, China
Strombus mutabiis|Mollusca, Gastropoda, Littorinimorpha, Strombidae, Strombus|JF693420|Lingshui, Hainan province, China
Strombus vittatus|Mollusca, Gastropoda, Littorinimorpha, Strombidae, Strombus|JF693435|Weizhou Island, Guangxi province, China
Strombus vittatus|Mollusca, Gastropoda, Littorinimorpha, Strombidae, Strombus|JF693434|Beihai, Guangxi province, China
Strombus vittatus|Mollusca, Gastropoda, Littorinimorpha, Strombidae, Strombus|JF693433|Beihai, Guangxi province, China
Margistrombus robustus|Mollusca, Gastropoda, Littorinimorpha, Strombidae, Strombus|JF693437|Beihai, Guangxi province, China
Margistrombus robustus|Mollusca, Gastropoda, Littorinimorpha, Strombidae, Strombus|JF693427|Beihai, Guangxi province, China
Margistrombus robustus|Mollusca, Gastropoda, Littorinimorpha, Strombidae, Strombus|JF693426|Beihai, Guangxi province, China

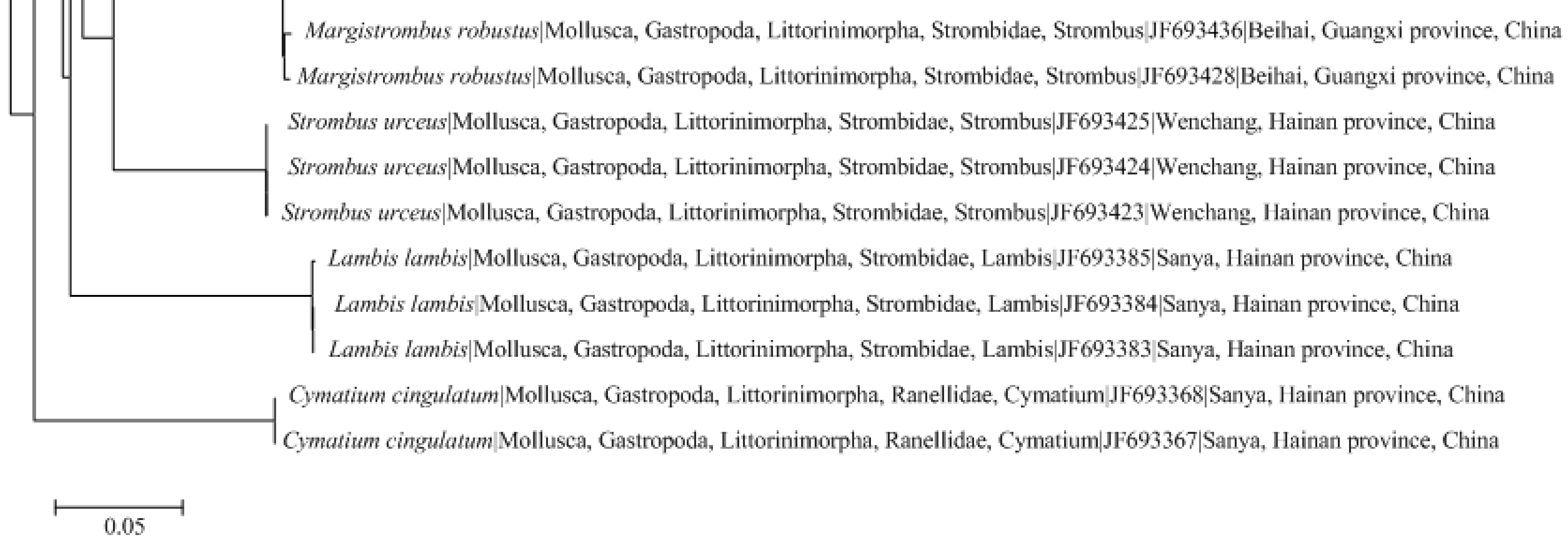
Margistrombus robustus|Mollusca, Gastropoda, Littorinimorpha, Strombidae, Strombus|JF693436|Beihai, Guangxi province, China
Margistrombus robustus|Mollusca, Gastropoda, Littorinimorpha, Strombidae, Strombus|JF693428|Beihai, Guangxi province, China
Strombus urceus|Mollusca, Gastropoda, Littorinimorpha, Strombidae, Strombus|JF693425|Wenchang, Hainan province, China
Strombus urceus|Mollusca, Gastropoda, Littorinimorpha, Strombidae, Strombus|JF693424|Wenchang, Hainan province, China
Strombus urceus|Mollusca, Gastropoda, Littorinimorpha, Strombidae, Strombus|JF693423|Wenchang, Hainan province, China
Lambis lambis|Mollusca, Gastropoda, Littorinimorpha, Strombidae, Lambis|JF693385|Sanya, Hainan province, China
Lambis lambis|Mollusca, Gastropoda, Littorinimorpha, Strombidae, Lambis|JF693384|Sanya, Hainan province, China
Lambis lambis|Mollusca, Gastropoda, Littorinimorpha, Strombidae, Lambis|JF693383|Sanya, Hainan province, China
Cymatium cingulatum|Mollusca, Gastropoda, Littorinimorpha, Ranellidae, Cymatium|JF693368|Sanya, Hainan province, China
Cymatium cingulatum|Mollusca, Gastropoda, Littorinimorpha, Ranellidae, Cymatium|JF693367|Sanya, Hainan province, China
0.05
